# Copper-Mediated Nitrogen-Atom Migration into Saturated Carbocycles

**DOI:** 10.1021/jacs.6c15884

**Published:** 2026-10-07

**Authors:** Michael J. Zeiler, Matthieu F. Maciejewski, Cade A. MacAllister, Tehshik P. Yoon

**Affiliations:** Department of Chemistry, University of Wisconsin−Madison, 1101 University Avenue, Madison, Wisconsin 53706, United States

## Abstract

The conversion of carbocyclic rings into medicinally significant heterocycles represents a promising emerging strategy for molecular remodeling of pharmaceutical lead compounds. The selective activation and functionalization of C­(sp^3^)–C­(sp^3^) bonds, however, remains a formidable challenge for generalizing these methods to saturated rings. We report an operationally straightforward, one-step method for the formal insertion of nitrogen atoms into the skeletal cores of saturated carbocycles. This method leverages the underexplored ability of nitrogen-centered radical intermediates to trigger deconstructive β-scission events, and we have discovered a Cu­(II) system that both generates nitrogen-centered radicals directly from sulfonamides and mediates the oxidative cyclization of the ring-opened organoradical intermediates. Given its broad functional group tolerance, scalability, and capacity for late-stage scaffold-hopping, this strategy establishes nitrogen-centered radical-mediated β-scission as a viable, general tool for the synthesis of nitrogen heterocycles by skeletal editing.

## Graphical abstract

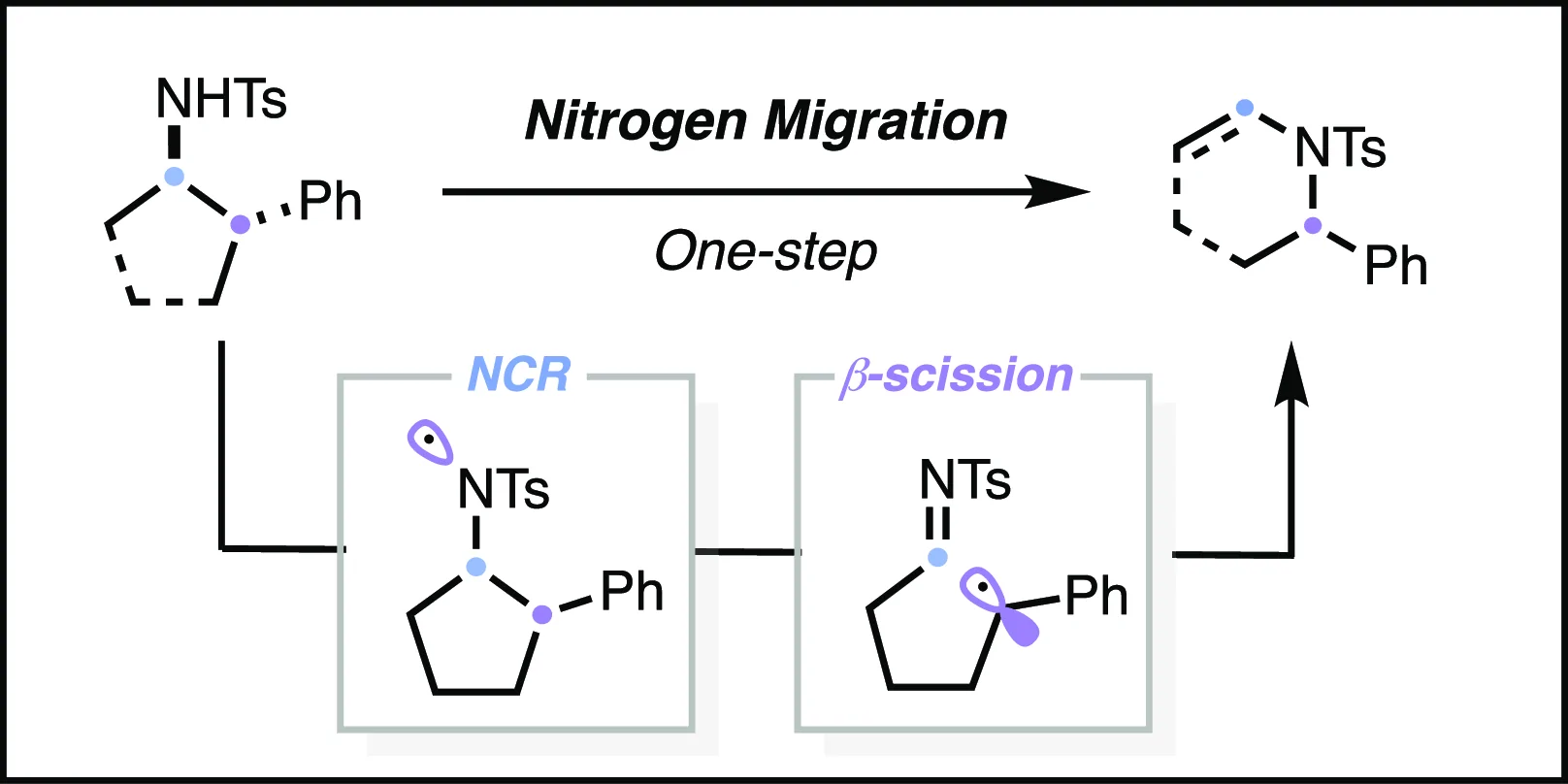

Strategies that enable the conversion of one core skeletal ring into another have emerged as an exciting new capability in synthetic chemistry.^1,2^ Reactions that can transform carbocycles into medicinally significant heterocycles are particularly valuable, but a central challenge has been the difficulty of selectively activating the relatively inert C­(sp^3^)–C­(sp^3^) bonds of saturated carbocycles. While some strategies exploit the strained bonds of small ring systems,^3,4^ the most common industrial methods for reformation of unstrained hydrocarbon substrates require highly energetic conditions that do not tolerate highly functionalized complex molecules (Figure 1A). One attractive alternative leverages the high reactivity of oxygen-centered radicals, which can undergo facile β-scission of adjacent C­(sp^3^)–C­(sp^3^) bonds^5–19^ and have been utilized in the development of challenging single-atom carbocycle insertions.^20–22^ The Knowles group recently deployed this approach in a method for *exo*-to-*endo* nitrogen migration in cyclic 1,2-amino alcohols (Figure 1B).^21^ This two-step protocol involves generation of an alkoxy radical via a proton-coupled electron transfer (PCET) step followed by subsequent intramolecular reductive amination to assemble the *N*-heterocycle. Our group explored a complementary strategy for oxidative conversion of cycloalkanols into cyclic ethers (Figure 1C).^22^ In this strategy, a Cu­(II)-centered ligand-to-metal charge transfer (LMCT) initiates alkoxy radical mediated ring opening to a linear aldehydic intermediate, and subsequent Cu­(II)-mediated oxidative cyclization results in formal migration of the oxygen atom into the ring.

**Figure 1:**
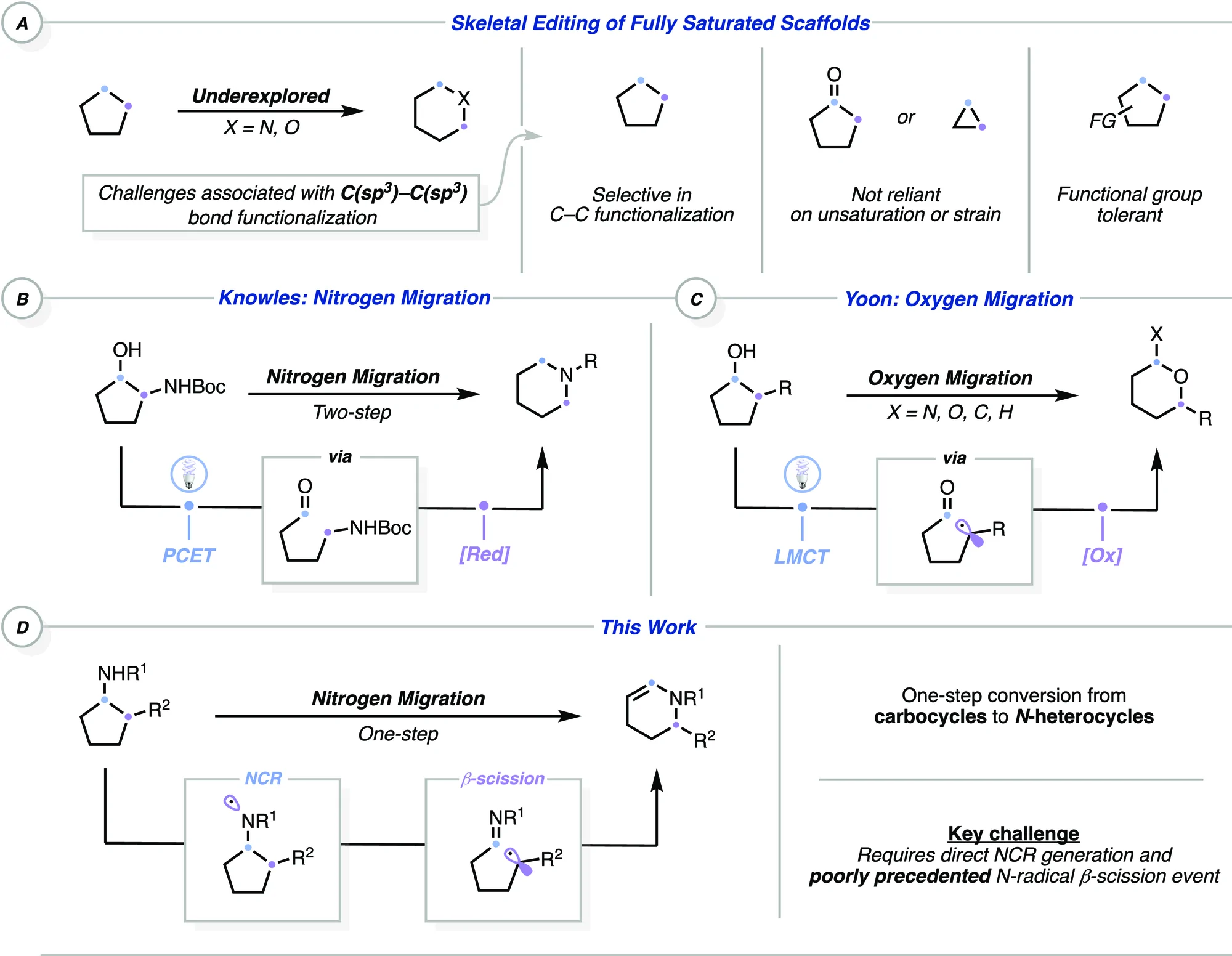
Background and summary of our approach. (A) Skeletal editing challenges of fully saturated scaffolds. (B) Nitrogen migration using alcohols by Knowles. (C) Our prior oxygen migration work. (D) This work: Nitrogen migration using sulfonamide functional groups.

We wondered if we might develop an analogous process that would leverage β-scission of nitrogen-centered radicals (NCRs) in a one-step skeletal rearrangement of saturated *N*-cycloalkylamines into synthetically valuable nitrogen heterocycles (Figure 1D).^23^ Although this idea is conceptually appealing, there is much less precedent for several key steps in this sequence. First, the direct generation of NCRs from native nitrogen functionalities has relatively limited precedent. Knowles and Lei have respectively reported photochemical^24,25^ and electrochemical^26^ generation of NCRs by PCET. Shi, West and Ritter have developed photochemical generation of NCRs by LMCT activation of Fe­(III)-azido^27,28^ and Cu­(II)-sulfoximide^29^ complexes. Chemler has proposed that Cu­(II) salts can mediate the thermal oxidation of easily oxidized *N*-aryl sulfonamides.^30,31^ Second, and perhaps more fundamentally, it was not obvious that NCR-initiated β-scission of relatively unstrained saturated carbocycles would be feasible. Radicals localized on nitrogen are generally more stable than those on highly electronegative oxygen atoms, and the formation of C=N double bonds provides less driving force than highly stable C=O bonds;^32^ correspondingly, previously documented examples of β-scission from NCRs involve either the cleavage of highly strained small rings or the generation of stable nitriles from iminyl radicals.^33–38^ Herein, we report a simple protocol for the oxidative migration of nitrogen atoms into the adjacent C­(sp^3^)–C­(sp^3^) bond of a saturated carbocycle, demonstrating the utility of NCR β-scission as a strategic tool for the design of new synthetically useful skeletal editing methods.

Our investigations commenced by studying the reaction of sulfonamide **1** under conditions previously optimized for the analogous transformation of cycloalkanols into oxygen heterocycles. We were pleased to observe the formation of enamine **2**, albeit in only 29% yield (Figure 2A, entry 1). Exploration of base identity revealed sterically encumbered pyridyl bases were uniquely effective, with 2,6-di-*tert*-butylpyridine (2,6-DTBPy) increasing the yield to 70% (entry 2). Reactivity could be further improved by doubling the concentration of each reagent, which afforded a near quantitative yield (entry 3). Other copper­(II) salts afforded no product (entry 4). Surprisingly, a control reaction conducted without irradiation produced **2** in high yield (Figure 2B). The thermal nitrogen-atom insertion process seemed to be dependent on base identity, and specifically on the use of sterically bulky 2,6-disubstituted pyridines: experiments employing pyridine itself produce **2** only under photochemical conditions.

**Figure 2:**
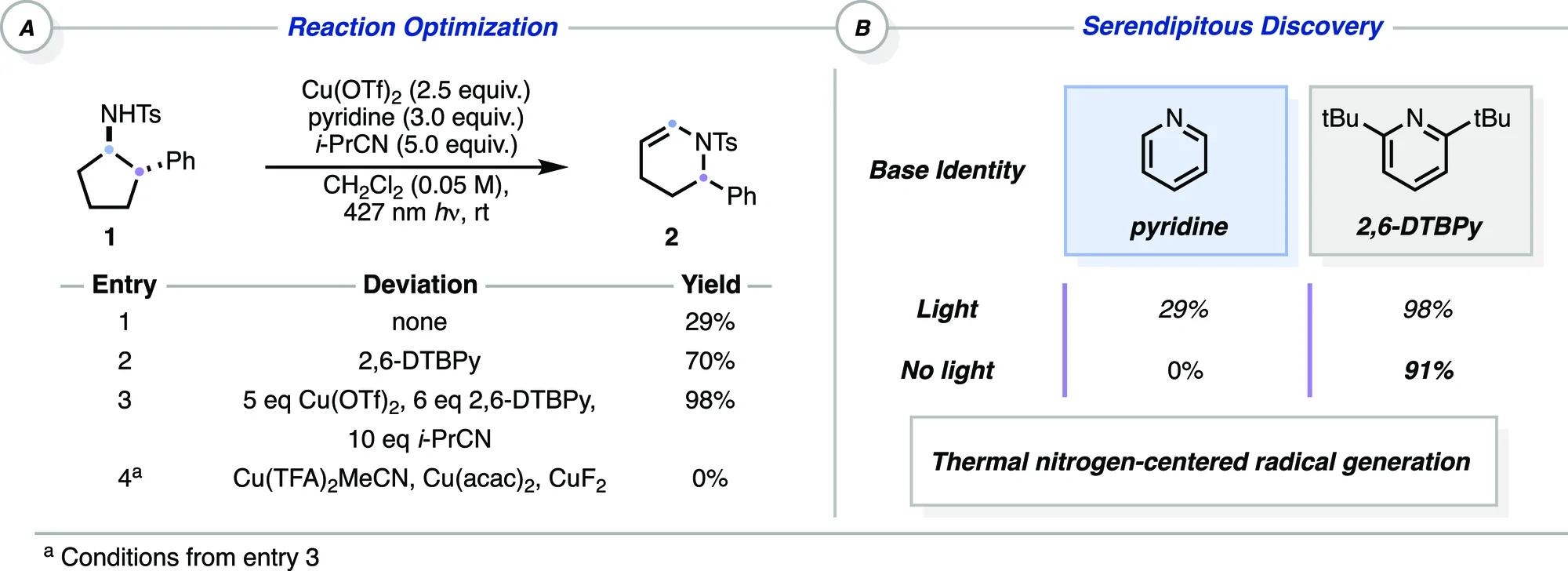
(A) Reaction optimization. (B) Discovery of thermal NCR generation with 2,6-DTBPy.

The conditions required for this nitrogen-insertion transformation are operationally simple. The reaction is a one-step process that operates directly on the sulfonamide substrate without oxidative prefunctionalization, it avoids strong chemical oxidants, and it proceeds at ambient temperatures without the application of external stimuli such as light or electricity (Figure 3A). The reaction appears general for a range of electronically varied sulfonamide protecting groups (Figure 3B, **3**–**6**). Other protecting groups, including carbamates and benzamides, however, did not furnish the insertion product (see SI). Both *trans* and *cis* diastereomers of sulfonamide **1** afforded similar yields of **2** (Figure 3C). Modification of the arene substituent revealed that high yields are obtained with electron-rich (**7**, **8**) and electron-poor arenes (**9**, **10**), although the reaction appeared somewhat more successful with electron-rich substrates. Consistent with the mild oxidative conditions, the reaction tolerated multiple functional groups common in drug discovery, including carbamates, amides, and esters (**11**–**13**). Additionally, substrates containing medicinally valuable benzofuran, indazole, indole, and deactivated pyridine heterocycles were all smoothly converted to their corresponding insertion products (**14**–**17**), suggesting that this reaction could be an enabling transformation for analogue synthesis. Six-membered rings (**18**), however, proved unreactive (Figure 3D). Similarly, the parent unsubstituted *N*-cyclopentyl benzenesulfonamide (**19**) did not undergo insertion; however, introduction of vinyl substituents β to the sulfonamide restored reactivity, affording **20** and **21** in 91% and 73% yield, respectively (Figure 3E). Unlike photochemical reactions, this thermally induced insertion reaction is readily scaled: a gram-scale reaction (2.5 mmol) afforded 82% yield of **22** even when the concentrations of Cu­(OTf)_2_ and 2,6-DTBPy were halved. Finally, to investigate the ability of this insertion reaction to enable scaffold-hopping between bioactive targets, we prepared sulfonamide **23** featuring the elaborated cyclopentylamino nucleus of a class of compounds investigated by Merck as NK1 antagonists (Figure 3F).^39^ Subjecting this compound to the optimized conditions for nitrogen insertion followed by acid-catalyzed hydride reduction of enamine **24** results in the facile production of piperidine **25**, featuring the core substitution pattern of an NK1 antagonist class with up to 4-fold greater binding affinity.^40^

**Figure 3:**
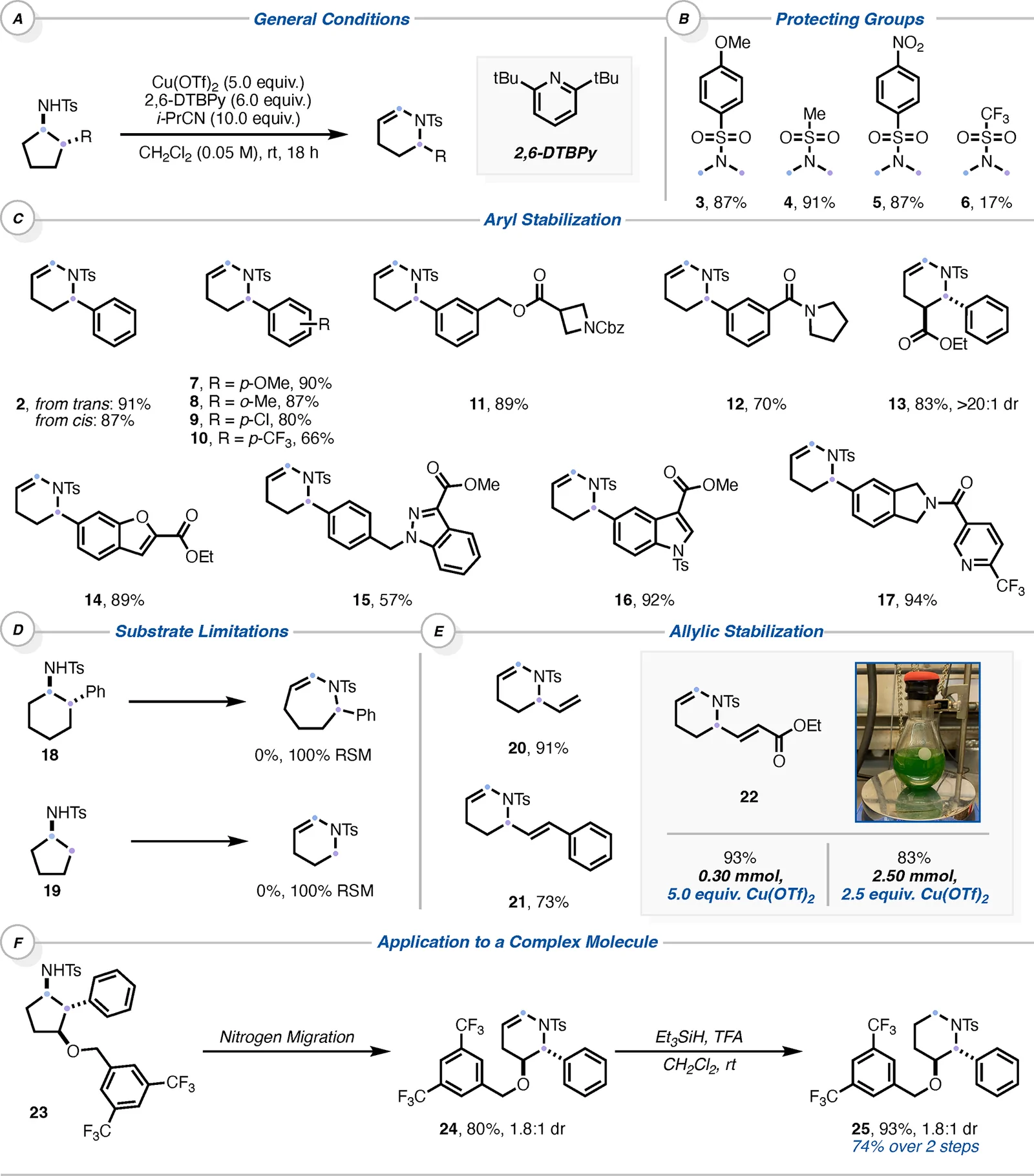
(A) General conditions for nitrogen migration. (B) Protecting group scope. (C) Aryl stabilization scope. (D) Substrate limitations. (E) Allylic stabilization scope. (F) Application of nitrogen migration for interconversion of NK1 inhibitor scaffolds.

The design of this nitrogen-insertion reaction was premised on the ability of NCRs to trigger a key β-scission event, in analogy to the oxygen-insertion method we recently reported. While β-scission of unstrained rings from oxygen-centered radicals has substantial precedent, similar reactions of NCRs have not been previously reported. To test whether such a radical ring-opening event might be feasible, we first prepared *N*-chlorosulfonamide **26** and subjected it to conditions previously developed for Mn-catalyzed NCR generation by halogen-atom abstraction (Figure 4A).^41^ This experiment resulted in the formation of the ring-opened benzylic chloride **27** in 49% yield, presumably via the halogen-atom chain-transfer process invoked by Zhang. We conclude that β-scission of an unstrained ring can indeed be initiated by generation of an NCR, that this process does not depend on the presence of a Cu salt, and that cyclization to afford the nitrogen-insertion product does not occur when the NCR is generated by metalloradical-catalyzed halogen-atom transfer.

**Figure 4:**
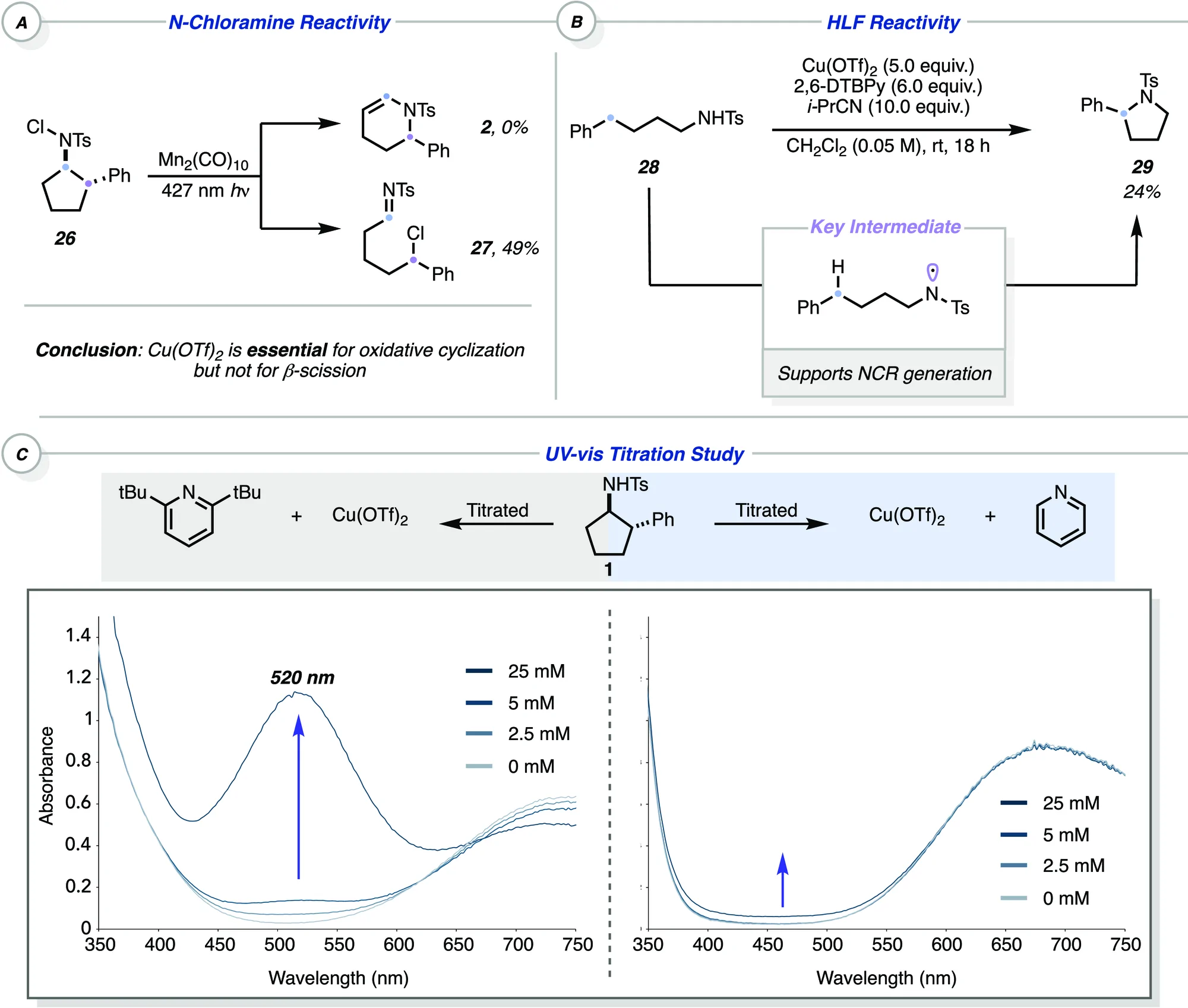
(A) *N*-Chloramine reactivity study. (B) HLF reactivity study. (C) UV–vis titration study with Cu­(OTf)_2_ (25 mM), pyridyl base (30 mM), and increasing amounts of **1**. The UV–vis study was conducted in MeCN for improved solubility.

To further examine whether the conditions optimized for the nitrogen-insertion reaction result in the formation of an NCR, we designed a linear sulfonamide substrate (**28**) that we expected would be less prone to undergo β-scission. We reasoned that this substrate could instead be induced to undergo a different canonical reaction known to be mediated by the NCR intermediate. In particular, the NCR would be poised to undergo an intramolecular 1,5-HAT process; subsequent oxidative coupling of the translocated radical would then result in formation of a pyrrolidine in a formal Hofmann–Löffler–Freytag (HLF) reaction.^42^ Indeed, this experiment resulted in the formation of pyrrolidine **29** in 24% yield, consistent with the ability of the Cu­(OTf)_2_/2,6-DTBPy system to produce NCRs from sulfonamides (Figure 4B).

Chemler has previously reported that inner-sphere oxidation of sulfonamides by Cu­(II) can produce sulfonamidyl radicals.^30,31^ However, these previous reports were limited to the generation of resonance-stabilized *N*-aryl sulfonamidyl radicals, and generally at elevated temperatures. The ability to produce higher-energy *N*-alkylsulfonamidyl radicals at ambient temperature, therefore, offers a significantly expanded synthetic capability. The role of the 2,6-DTBPy base appears critical, as pyridine proved to be ineffective in this application. We conducted UV–vis titration studies to rationalize this effect (Figure 4C). Addition of sulfonamide **1** into an acetonitrile solution of Cu­(OTf)_2_ and 2,6-DTBPy resulted in a new feature centered at 520 nm, which we assigned to the coordination of **1** to Cu­(II). In contrast, no new feature was observed when **1** was titrated into a solution of Cu­(OTf)_2_ and pyridine. To further support the binding requirement, the *N*-methylated analog of **1** was subjected to the reaction conditions to afford only returned **1** (see SI). We hypothesize that the formation of a coordinatively saturated Cu­(II) pyridine complex is disfavored using a 2,6-DTBPy base, facilitating association with and subsequent inner-sphere oxidation of sulfonamide **1**.

The scope of NCR-triggered β-scission events has not previously been the subject of systematic investigation; however, relatively simple thermochemical DFT calculations are effective in rationalizing the empirically determined scope of the nitrogen insertion reaction (Scheme 1A). All structures were optimized and minima confirmed on the potential energy surface using B3LYP with the 6-31­(d) basis set, and a polarizable continuum model for dichloromethane was used. All calculations were performed with Gaussian 16.^43^ The β-scission of simple *N*-cyclopentyl benzenesulfonamidyl radical **30** was calculated to be highly endergonic (+12.0 kcal/mol), consistent with the failure of the corresponding sulfonamide **19** to undergo nitrogen insertion. This may be due to the diminished electronegativity of nitrogen compared to oxygen, the lower thermodynamic stability of a C=N bond compared to a C=O bond, and the formation of a high-energy primary carbon-centered radical. Incorporating an aryl substituent capable of stabilizing the organoradical intermediate, however, rendered the β-scission event exergonic (**31**, −3.88 kcal/mol). Benzylic stabilization is not sufficient to promote the β-scission of a six-membered ring (**32**, +5.98 kcal/mol). On the other hand, both aryl-substituted and unsubstituted cyclobutyl sulfonamides (**33** and **34**, respectively) were exergonic in their scission event (−15.1 and −6.23 kcal/mol, respectively) due to relief of their associated ring strain.

**Scheme 1:**
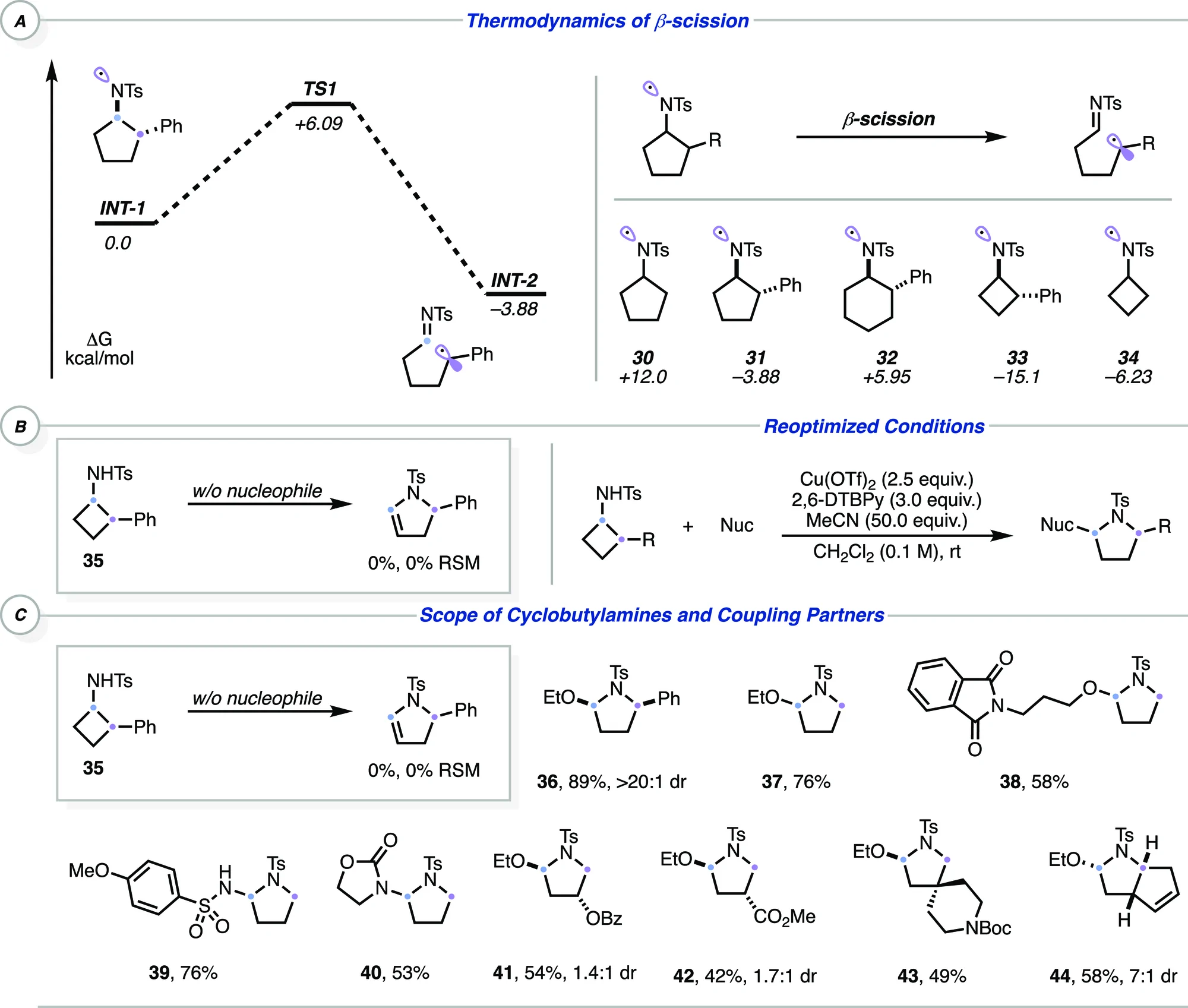
(A) Computational Study on the *N*-Radical β-Scission Process for Various Cycloamines. (B) General Conditions for the Cyclobutyl Amine Scope. (C) Scope of Cyclobutylamines with Various Coupling Partners

These calculations suggested that synthetically readily accessible *N*-cyclobutyl sulfonamides might be excellent substrates for nitrogen insertion (Scheme 1B). However, while 2-phenylcyclobutyl sulfonamide **35** was completely consumed upon treatment with Cu­(OTf)_2_/2,6-DTBPy, the corresponding dihydropyrrole was not detected (Scheme 1C). We reasoned that the increased strain of the five-membered enesulfamide renders it unstable toward the oxidizing conditions of this reaction. Thus, we wondered whether we could instead intercept the iminium intermediate with an exogenous nucleophile to furnish a 2,5-substituted pyrrolidine. Gratifyingly, upon addition of ethanol, we observed efficient trapping of the iminium to afford pyrrolidine **36** in 89% yield and with >20:1 diastereoselectivity. Along with 2-phenylcyclobutylamines, our computations also suggested that the β-scission should occur for unstabilized cyclobutyl-sulfonamides. Indeed, with minimal modification to the conditions, we were able to form pyrrolidines using alcohol, sulfonamide, and carbamate nucleophiles without the need for radical stabilization (**37**–**40**). A brief exploration of the scope showed that the reaction was similarly tolerant of functionality (**41**, **42**) and could be used to access interesting spirocyclic and fused ring systems (**43**, **44**), furthering the synthetic potential of this nitrogen migration strategy.

In summary, we have developed an operationally simple, one-step protocol for the oxidative insertion of nitrogen atoms into saturated carbocyclic rings. This method takes advantage of the underexplored ability of NCRs to trigger a wide range of β-scission reactions, including those of unstrained rings, the feasibility of which can be readily assessed using simple thermochemical calculations. A variety of mechanistic experiments are consistent with the hypothesis that a Cu­(OTf)_2_/2,6-DTBPy system is a uniquely potent strategy for the generation of sulfonamidyl radicals directly from sulfonamides without the need for oxidative prefunctionalization of the nitrogen, strong chemical oxidants, or external photochemical stimulus. Ultimately, this work establishes the strategic utility of NCR-mediated β-scission as a powerful tool for skeletal editing. Given the exceptional functional group tolerance, scalability, and demonstrated capacity for late-stage scaffold-hopping of bioactive targets, we anticipate that this platform will find broad utility in medicinal chemistry and complex molecule synthesis.

## Supplementary Material



## Notes

General experimental details, synthesis of sulfonamides, nitrogen migration conditions, UV–vis experiments, computational data, additional mechanistic experiments, and characterization of new compounds (PDF)
