# Supplementary material for: Copper-Mediated Nitrogen-Atom Migration into Saturated Carbocycles
Source: J Am Chem Soc. 2026 Oct 7;148(41):44005–11. doi: 10.1021/jacs.6c15884 (PMC13693389; doi:10.1021/jacs.6c15884)
Supplement: Supplementary file 1 [file ja6c15884_si_001.pdf]

Supplementary Information

Copper-mediated Nitrogen-atom Migration into Saturated Carbocycles

Michael J. Zeiler, Matthieu F. Maciejewski, Cade A. MacAllister, Tehshik P. Yoon*

Department of Chemistry, University of Wisconsin–Madison, 1101 University Avenue, Madison, Wisconsin 53706 USA.

correspondence to: tyoon@chem.wisc.edu (T. P. Y.)

Table of Contents:

**A.**	**General Information**	**5**
**B.**	**Select optimization results**	**6**
**C.**	**General Procedures for sulfonamide preparation**	**9**
**D.**	**Preparation of cyclopentyl and cyclohexyl sulfonamides**	**12**
**E.**	**Preparation of cyclobutyl sulfonamides**	**31**
**F.**	**Experimental procedure, isolation, and characterization for enamine products**	**36**
**G.**	**Experimental procedure, isolation, and characterization for pyrrolidine products**	**44**
**H.**	***N*-Chloramine reactivity study**	**47**
**I.**	**Hofmann-Löffler-Freytag reactivity study**	**48**
**J.**	**UV-Vis studies**	**49**
**K.**	**Nitrogen protecting groups**	**58**
**L.**	**Other mechanistic experiments**	**60**
**M.**	**Unsuccessful Substrates**	**64**
**N.**	**Computational Analysis**	**65**
**O.**	**NMR Spectra**	**86**
**P.**	**References and Notes**	**168**

List of Tables:

Table S1. Pyridyl base screen with and without light

Table S2. Metal salt screen

Table S3. Nitrile screen

Table S4. Solvent screen

Table S5. Air and moisture sensitivity screen

Table S6. Solutions prepared for UV-Vis analysis of the reaction components

Table S7. Solutions prepared for UV-Vis titration of Cu/2,6-di-tertbutylpyridine with **1**

Table S8. Solutions prepared for UV-Vis titration of Cu/2,6-di-tertbutylpyridine with **1**

Table S9. Solutions prepared for UV-Vis titration of Cu/2,6-di-tertbutylpyridine with **S45**

Table S10. Calculated free energies for reaction intermediates and transition states

List of Figures:

Figure S1. UV-vis of reaction components in MeCN

Figure S2. UV-vis titration of **1** into a $Cu(OTf)_2$/2,6-di-tertbutylpyridine solution

Figure S3. UV-vis titration of **1** into a $Cu(OTf)_2$/pyridine solution

Figure S4. UV-vis titration of **S45** into a $Cu(OTf)_2$/2,6-di-tertbutylpyridine solution

Figure S5. Unsuccessful substrates for the nitrogen migration.

Figure S6. Structures for DFT Analysis

A. General Information

Reagent Preparation: Dichloromethane, diethyl ether, toluene, tetrahydrofuran and acetonitrile were dried by elution through alumina as described by Grubbs.[1] $Cu(OTf)_2$ was purchased from Oakwood Chemical and stored in glovebox. All pyridyl bases and $^{i}PrCN$ were distilled over CaH_2. Anhydrous EtOH was purchased from Sigma Aldrich and stored under an inert atmosphere. All other chemicals were purchased from commercial suppliers and used as received unless otherwise stated. 40 W PR160L 427 nm LEDs from Kessil Lights were used as light source. Flash chromatography was performed with Sigma Aldrich 60 Å silica gel (230–400 mesh) and thin layer chromatography (TLC) was performed utilizing pre-coated silica gel F254 plates from SiliCycle Inc. containing a fluorescent indicator. Visualization of plates were accomplished with 254 nm UV lamp or staining with p-anisaldehyde or $KMnO_4$.

Product Characterization: All NMR spectra were obtained using a Bruker Avance-400, Avance-500 or Avance-600 spectrometer, and the spectra were internally referenced according to $CDCl_3$ chemical shifts (7.26 ppm for 1H NMR and 77.16 ppm for 13C NMR). The spectrometers used for this work are funded by the NSF (CHE1048642), NIH (1S10 OD020022-1) and a generous gift from Paul J. and Margaret M. Bender. Diastereomer ratios for reactions were determined by ^{1}H NMR analysis of reaction mixtures vs. a 1-methylnapthalene internal standard or dibromomethane internal standard. NMR data are reported as follows: chemical shift, multiplicity (s = singlet, d = doublet, t = triplet, q = quartet, p = pentet, sext = sextet, sept = septet, m = multiplet), coupling constant(s) in Hz, integration. NMR spectra were obtained at 298 K unless otherwise noted. Mass spectrometry was performed with a Thermo Q Exactive™ Plus (electrospray ionization, time-of-flight analyzer or electron impact) and is funded by the NIH (1S10 OD020022-1). Melting points were obtained using a Stanford Research Systems DigiMelt apparatus. UV–Vis absorption spectra were acquired using a Varian Cary® 50 UV–visible spectrophotometer with a spectrophotometer.

B. Select Optimization Results

All optimization reactions were run according to this procedure unless otherwise indicated:

A flame dried 4-mL vial charged with sulfonamide **1** (0.05 mmol) and a stirbar was brought into the glovebox. $Cu(OTf)_2$ (2.5–5.0 equiv) was added to the vial followed by DCM (1.0 mL), *i*PrCN (5.0–10.0 equiv) and 2,6-di-*t*Bu-pyridine (3.0–6.0 equiv). The vial was then sealed with a Teflon cap, removed from the glovebox and stirred at room temperature or irradiated with a 40 W PR160L 427 nm Kessil Lamp for 18 hours. 1-methylnaphthalene (10 µL) was added to the reaction as an internal standard. The reaction was quenched by addition of 1 mL of saturated ammonium chloride and diluted with 2 mL of EtOAc. The organic layer was separated, then passed through a 0.5 cm plug of silica and Na_2SO_4, eluting with EtOAc, and concentrated to provide a residue. This residue was diluted in $CDCl_3$ and submitted for NMR analysis.

Table S1. Pyridyl base screen with and without light

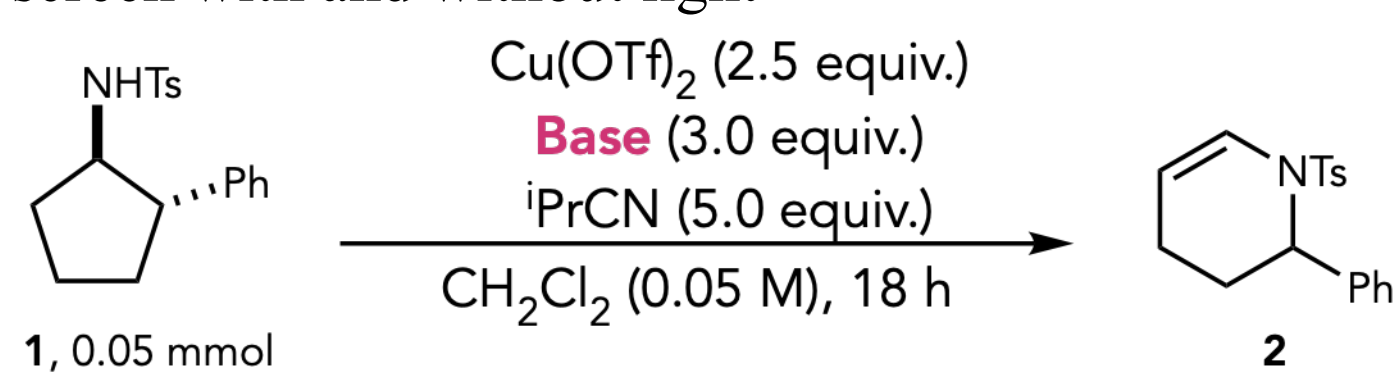

Entry	Base	PDT	RSM
**427 nm light**			
1	Pyridine	29%	67%
2	2,6-lutidine	56%	24%
3	2,4,6-collidine	60%	27%
4	2,6-DTBPy	70%	26%
5	none	0%	49%
**no light, rt**			
6	Pyridine	0%	96%
7	2,6-lutidine	60%	30%
8	2,4,6-collidine	64%	21%
9	2,6-DTBPy	71%	28%
10	4-Me-2,6-DTBPy	85%	14%
11	none	0%	66%

Table S2. Metal salt screen

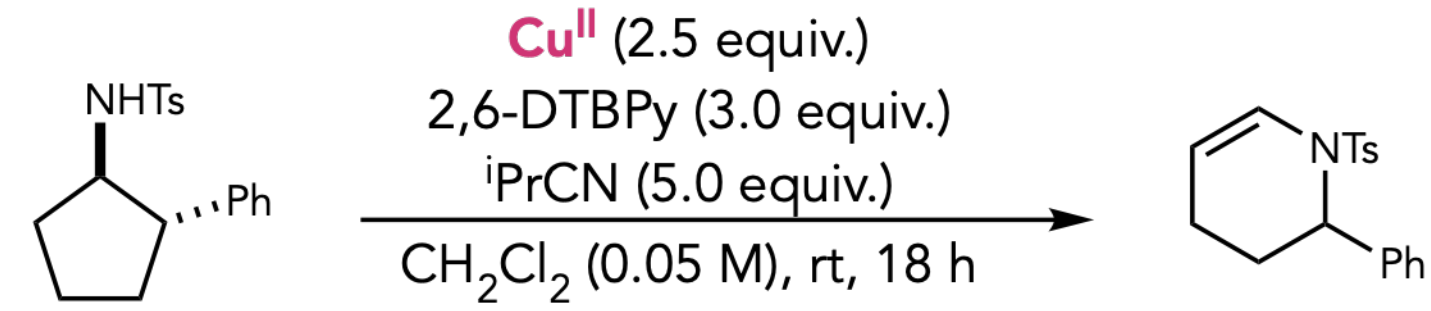

1, 0.05 mmol → **2**

Entry	Cu(II) Salts	PDT	RSM
1	$Cu(OTf)_2$	64%	37%
2	$Cu(TFA)_2MeCN$	0	>99%
3	$Cu(acac)_2$	0	>99%
4	CuF_2	0	>99%
5	$CuBr_2$	0	>99%
6	$Cu(OAc)_2$	0	>99%
7	$CuSO_4$	0	>99%
8	$Fe(OTf)_3$	trace	90%

Table S3. Nitrile screen

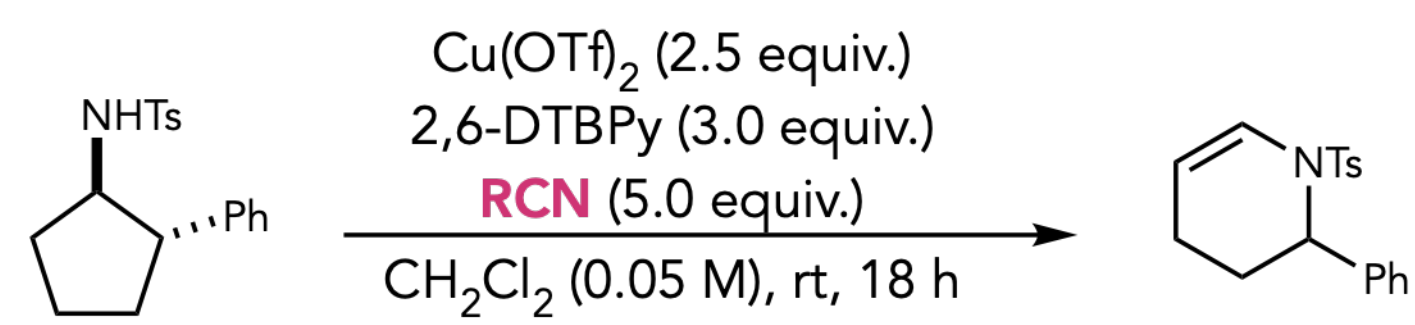

1, 0.05 mmol → **2**

Entry	RCN	PDT	RSM
1	MeCN	64%	37%
2	^{i}PrCN	45%	51%
3	PhCN	52%	49%
4	none	0%	72%

Table S4. Solvent screen

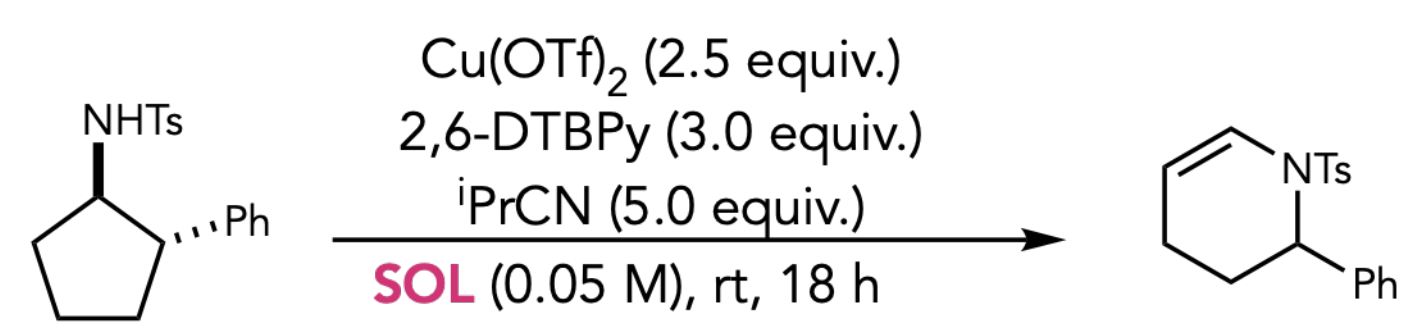

1, 0.05 mmol → **2**

Entry	SOL	PDT	RSM
1	DCM	64%	37%
2	THF	0%	>99%
3	Toluene	0%	>99%
	MeCN	53%	15%

Table S5. Air and moisture sensitivity screen

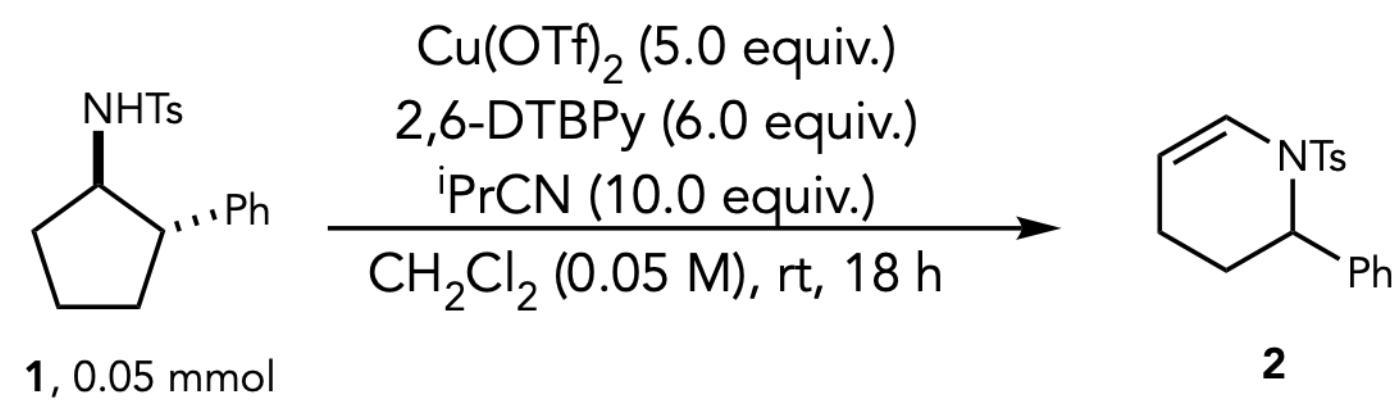

Entry	Setup	PDT
1	non-flame dried vial, open to air, non distilled base and nitrile	58%
2	Schlenk technique, outside of glove box	83%
3	standard procedure in glove box	91%

C. General procedures for sulfonamide preparation

General Procedure A: *Aziridination*

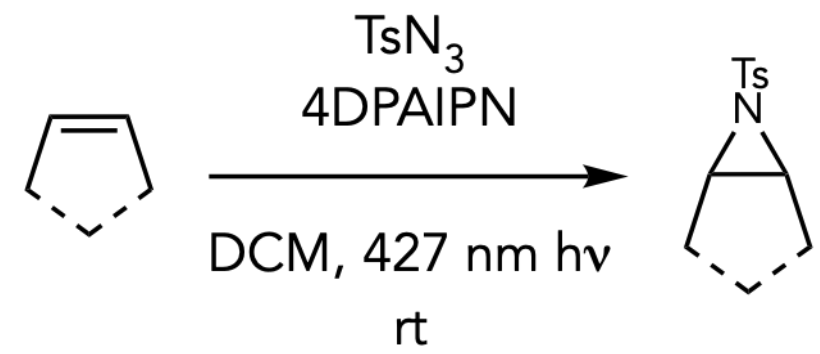

Adapted from a known literature procedure.[2] A flame dried round bottom flask was charged with 4DPAIPN (585 mg, 2.5 mol%, 0.734 mmol) and TsN_3 (8.1 g, 1.4 equiv, 41.10 mmol) and the flask was sealed then evacuated and backfilled with N_2 three times. Freshly distilled DCM (60 mL) was added followed by freshly distilled cyclopentene (2 g, 1 equiv, 30 mmol). The reaction was placed under a nitrogen atmosphere via Schlenk line (Note: This is necessary to allow for the safe release of nitrogen generated during reaction!). The reaction was placed in a water bath to help maintain temperature then irradiated with two 427 nm Kessil lamps for 6 hours (Nitrogen generation will begin immediately after lamps are turned on!). After 6 hours the reaction was concentrated then purified over silica with 0-20% acetone in hexanes to give the product as a light-yellow oil (5.68 g, 81%).

General Procedure B: *Aziridine Opening*

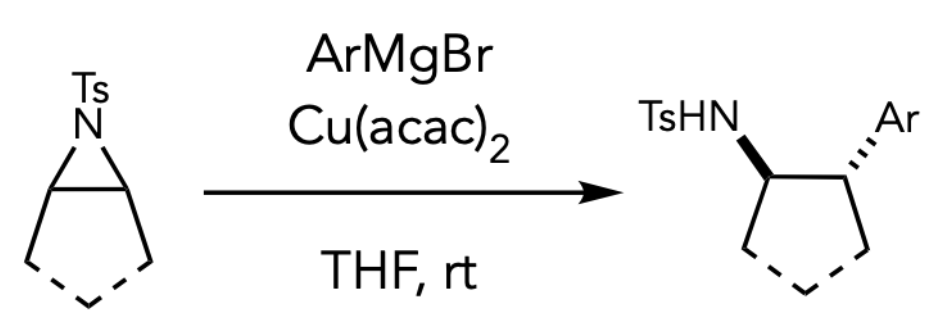

Grignard reagent was prepared by addition of magnesium powder (128 mg, 5 equiv, 5.27 mmol) to a flame dried three-neck round bottom fitted with a reflux condenser. Anhydrous THF (5 mL) was added followed by 2-3 drops of dibromomethane. The appropriate aryl bromide (5 equiv, 5.27 mmol) was added and the reaction was heated to reflux (70 °C) for one hour then cooled to room temperature.

To a separate flame dried vial was added cyclopentyl-aziridine (250 mg, 1 equiv, 1.0 mmol) and $Cu(acac)_2$ (42 mg, 15 mol%, 0.158 mmol) followed by anhydrous THF (5 mL). The cooled Grignard solution was then cannulated over to the solution containing aziridine and copper (color changes from blue to usually a dark purple) at 0 °C. The reaction was then left to warm to room temperature and stir overnight. The reaction was quenched by pouring into a solution of saturated ammonium chloride. The reaction was then extracted twice with EtOAc, and the combined organic layers were dried, concentrated and purified over silica with 0-20% ethyl acetate in hexanes to give the desired products.

General Procedure C: *Alpha-Arylation of Cyclopentenones*

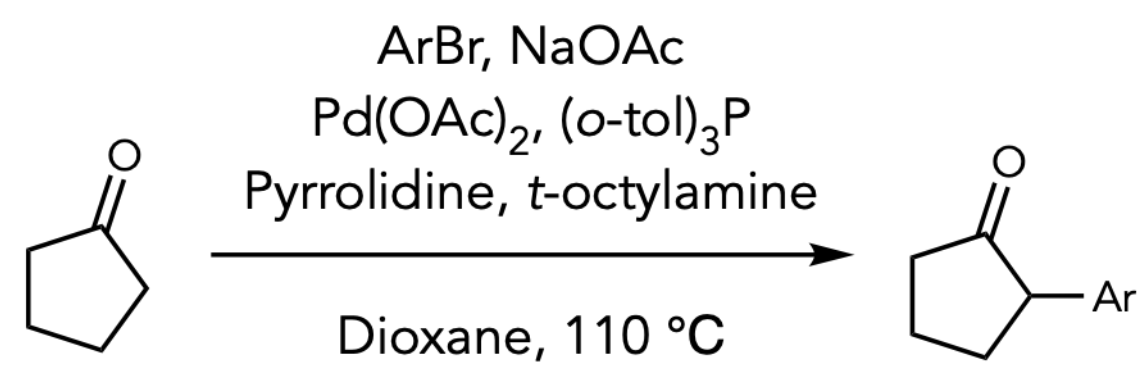

In a glove box, tri-*o*-tolyl-phosphine (362 mg, 0.2 equiv, 1.189 mmol), $Pd(OAc)_2$ (133 mg, 0.1 equiv, 0.594 mmol), sodium acetate (487 mg, 1 equiv, 5.9 mmol) and the appropriate aryl bromide (1 equiv, 5.944 mmol) were combined in a 40 mL vial and brought out of the glovebox. Anhydrous dioxane (25 mL) was added followed by freshly distilled cyclopentenone (0.526 mL, 1 equiv, 5.94 mmol), pyrrolidine (0.146 mL, 0.3 equiv, 1.78 mmol) and *tert*-octylamine (0.288 mL, 0.3 equiv, 1.78 mmol). The reaction was then heated to 110 °C overnight with stirring. The reaction was diluted with EtOAc and passed through a pad of silica, eluting with EtOAc, then concentrated. The resulting reside was purified over silica with 0-50% EtOAc in hexanes to give the arylated cyclopentanone product.

General Procedure D: *Reductive Amination Route*

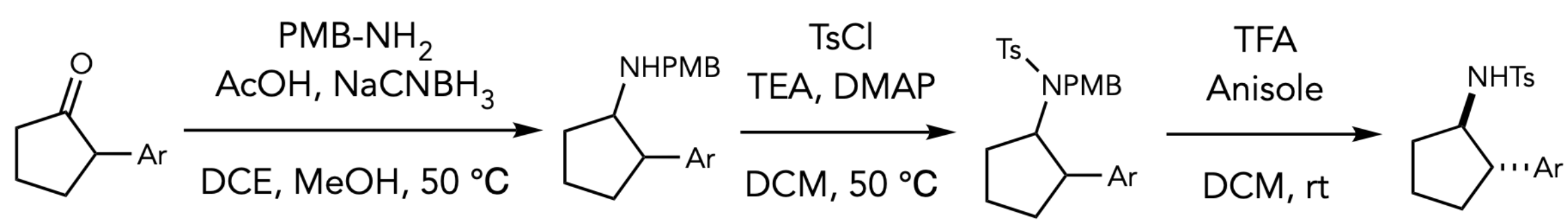

A vial was charged with the appropriate 2-aryl cyclopentanone (1 equiv, 1.5 mmol) followed by dichloroethane (4.2 mL) and anhydrous MeOH (3 mL). AcOH (0.255 mL, 3 equiv, 4.45 mmol) was added followed by *p*-methoxybenzylamine (407 mg, 2 equiv, 2.97 mmol) and the reaction was stirred at rt for 15 minutes. Next $NaCNBH_3$ (187 mg, 2 equiv, 2.97 mmol) was added and the reaction was heated to 50 °C overnight with stirring. After full consumption of ketone by TLC, the reaction was concentrated then poured into a 2 M potassium carbonate solution, then extracted three times with EtOAc. The combined organic layers were dried, concentrated and passed through a plug of silica, eluting with EtOAc, to afford the crude amine diastereomers.

The crude mixture was dissolved in THF (10 mL) then TEA (0.84 mL, 4 equiv, 6.0 mmol) and DMAP (60 mg, 0.2 equiv, 0.3 mmol) were added followed by TsCl (570 mg, 2 equiv, 3.0 mmol) and stirred at 50 °C overnight. The reaction was then poured into 1 M HCl and extracted three times with EtOAc. The combined organic layers were dried, passed through a plug of silica, eluting with EtOAc, and concentrated to afford the crude tosyl protected amines.

Finally, the crude tosyl protected amines were dissolved in DCM (10 mL) followed by addition of TFA (5.7 mL, 50 equiv, 75 mmol) and anisole (0.5 mL, 3 equiv, 4.5 mmol) and stirred for 3 hours with monitoring by TLC. When complete, the reaction was quenched by addition of saturated sodium bicarbonate then extracted 3 times with DCM. The organic layers were combined, dried, concentrated and purified over silica with 0-40% EtOAc in hexanes to isolate the desired trans diastereomers (consistently the more polar of the two diastereomers).

General Procedure E: *Sulfonylation*

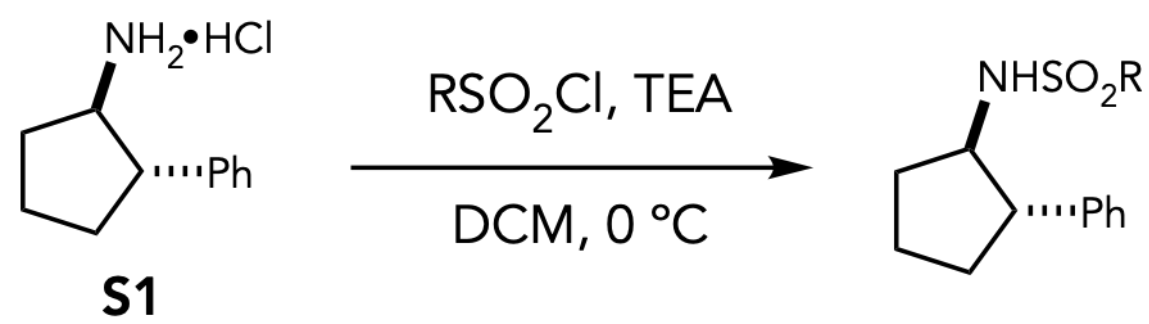

A flame dried 8 mL vial was charged with amine hydrochloride **S1** (100 mg, 1 equiv, 0.51 mmol), DCM (1.0 mL, 0.5 M) and triethylamine (211 µL, 3 equiv, 1.52 mmol). The flask was cooled to 0 ºC and the sulfonyl chloride (1.1 equiv, 0.56 mmol) was added. The contents gradually warmed to room temperature and stirred overnight. The reaction was quenched with H_2O (10 mL), extracted with DCM (2 x 10 mL), dried over $MgSO_4$, filtered, and concentrated. The crude sulfonamide was purified over silica with ethyl acetate and hexanes to afford the desired products.

D. Preparation of cyclopentyl and cyclohexyl sulfonamides

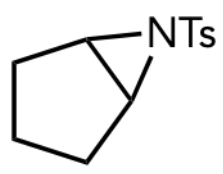

6-tosyl-6-azabicyclo[3.1.0]hexane (S2): Prepared according to **General Procedure A**. **^{1}H NMR** (400 MHz, $CDCl_3$) δ 7.85 – 7.77 (m, 2H), 7.32 (d, *J* = 8.0 Hz, 2H), 3.33 (d, *J* = 1.6 Hz, 2H), 2.44 (s, 3H), 1.99 – 1.87 (m, 2H), 1.69 – 1.51 (m, 3H), 1.49 – 1.31 (m, 1H). NMR values matched previously reported spectra.[3]

(±)-4-methyl-*N*-(trans-2-phenylcyclopentyl)-benzenesulfonamide (1): Prepared according to

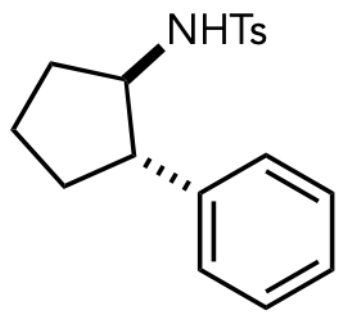

General Procedure B to give a white solid (245 mg, 78%). **^{1}H NMR** (500 MHz, $CDCl_3$) δ 7.52 – 7.46 (m, 2H), 7.22 – 7.10 (m, 5H), 7.00 (m, 2H), 4.49 (d, *J* = 6.0 Hz, 1H), 3.48 (m, 1H), 2.75 (m, 1H), 2.41 (s, 3H), 2.19 (m, 1H), 2.08 (m, 1H), 1.78 (m, 2H), 1.69 – 1.52 (m, 2H). NMR values matched previously reported spectra.[4]

*Synthesis of (cis-**1**):*

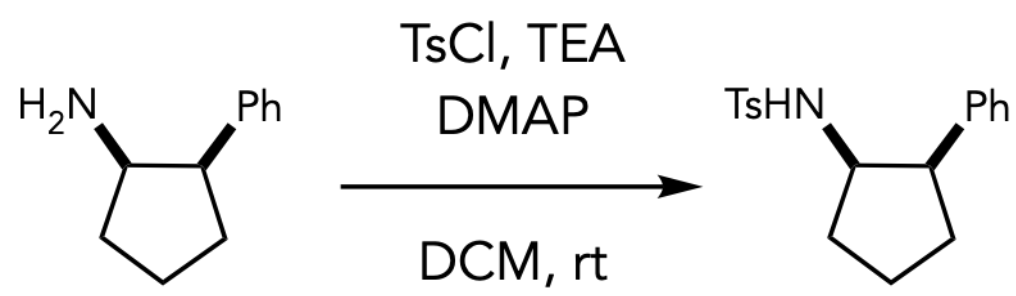

(±)-4-methyl-*N*-(cis-2-phenylcyclopentyl)-benzenesulfonamide (*cis*-1): Cis-2-

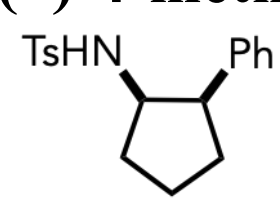

phenylcyclopentylamine (100 mg, 0.62 mmol, 1 equiv) was dissolved in DCM (3 mL). DMAP (8 mg, 0.1 equiv, 0.062 mmol), TEA (0.173 mL, 2 equiv, 1.24 mmol) and TsCl (177 mg, 1.5 equiv, 0.93 mmol) were added and the reaction was stirred for 3 hours at room temperature. After complete, reaction was concentrated under reduced pressure and directly columned over silica with 0-40% EtOAc in hexanes to give the desired product as a colorless oil (160 mg, 81%). **^{1}H NMR** (500 MHz, $CDCl_3$) δ 7.55 – 7.49 (m, 2H), 7.28 – 7.19 (m, 5H), 6.99 – 6.91 (m, 2H), 3.96 (d, *J* = 6.1 Hz, 1H), 3.77 – 3.69 (m, 1H), 3.20 – 3.13 (m, 1H), 2.44 (s, 3H), 2.10 – 2.00 (m, 1H), 1.98 – 1.86 (m, 4H), 1.75 – 1.64 (m, 1H). **^{13}C NMR** (126 MHz, $CDCl_3$) δ 143.2, 139.3, 137.3, 129.6, 128.8, 128.4, 127.2, 127.1, 57.9, 48.2, 32.6, 28.3, 21.7, 21.6. **HRMS** (ESI) calculated for $[C_{18}H_{20}NO_2S]^-$ $(M-H)^-$ requires *m/z* 314.1220, found 314.1223.

Synthesis of (±)-(1S,2R)-2-phenylcyclopentan-1-amine hydrochloride:

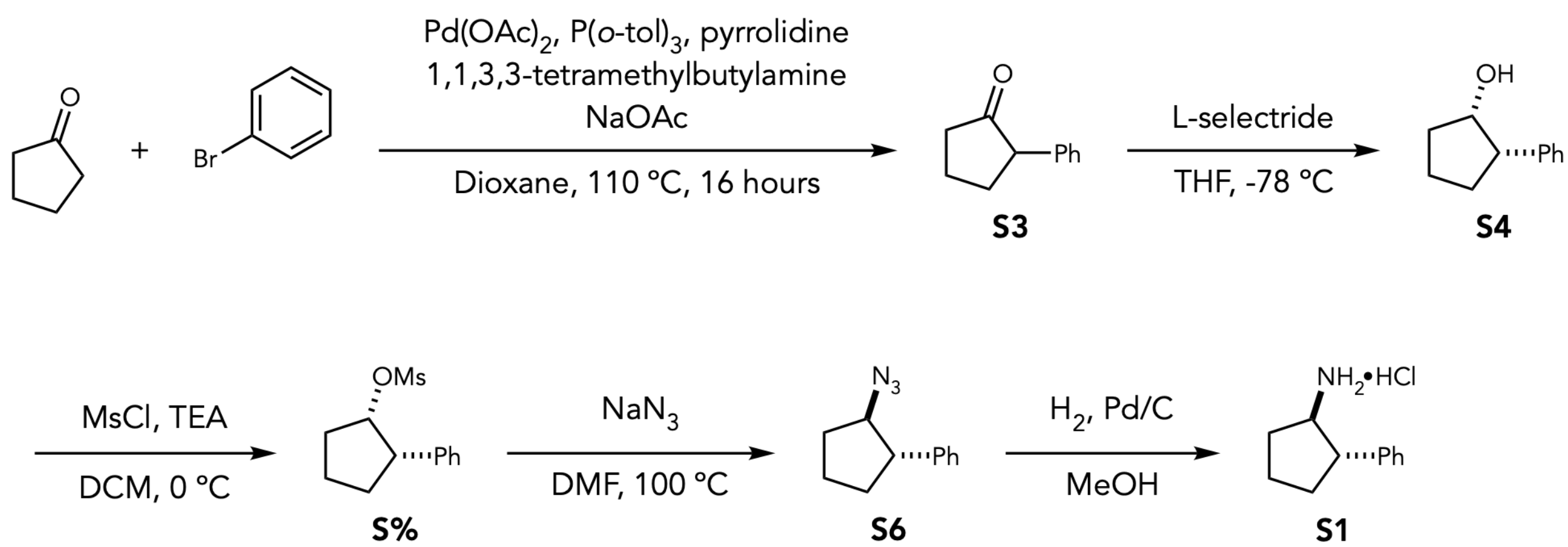

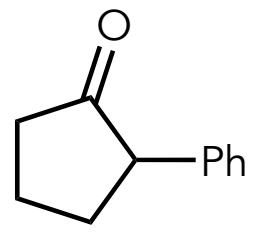

2-phenylcyclopentan-1-one (S3): Adapted from a published protocol[1]. A 500 mL 3-neck flame-dried round bottom flask was charged with Pd(OAc)2 (936 mg, 10 mol%, 4.16 mmol), P(*o*-tol)3 (2.53 g, 20 mol%, 8.32 mmol), and NaOAc (3.41 g, 1 equiv, 41.6 mmol) and the flask was sealed then evacuated and backfilled with N_2 three times. Bromobenzene (5.7 mL, 1.3 equiv, 54.1 mmol) was syringed in, followed by 1,4 dioxane (208 mL, 0.2 M), cyclopentanone (3.5 g, 1 equiv, 41.6 mmol), pyrrolidine (1.03 mL, 30 mol%, 12.5 mmol), and 1,1,3,3-tetramethylbutylamine (2.01 mL, 30 mol%, 12.5 mmol). The flask then refluxed at 110 ºC and stirred overnight, before cooled to room temperature. The mixture was filtered through a silica plug with ethyl acetate. The solvent was removed in *vacuo* then purified over silica with 0-15% ethyl acetate in hexanes to afford the product (**S3**) as a yellow oil (3.78 g, 57%). **^{1}H NMR** (500 MHz, CDCl3) δ 7.36 – 7.30 (m, 2H), 7.28 – 7.22 (m, 1H), 7.21 – 7.16 (m, 2H), 3.36 – 3.27 (m, 1H), 2.56 – 2.42 (m, 2H), 2.36 – 2.22 (m, 1H), 2.21 – 2.06 (m, 2H), 1.93 (dtdd, J = 10.8, 8.7, 7.2, 5.5 Hz, 1H). NMR values matched previous reported spectra.[5]

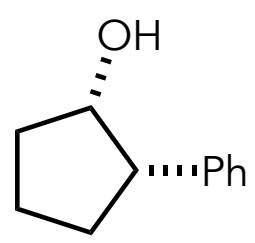

(±)-(1*S*,2*S*)-2-phenylcyclopentan-1-ol (S4): A 500 mL 3-neck flame-dried round bottom flask was charged with **S3** (3.78 g, 1 equiv, 23.6 mmol) and the flask was sealed then evacuated and backfilled with N_2 three times. THF (236 mL, 0.1 M) was added and the flask was cooled to -78 ºC. A 1 M solution of L-selectride (55 mL, 2.3 equiv, 55 mmol) was added dropwise via dropping funnel. The reaction was allowed to warm to room temperature and stir overnight. The reaction was cooled to 0 ºC and quenched slowly with 2.5 M NaOH (30 mL) and 30% H_2O_2 (30 mL). The reaction was warmed to room temperature and extracted with diethyl ether (3 x 100 mL), washed with H_2O (2 x 150 mL), then dried with $MgSO_4$, filtered, and concentrated. The crude *cis* alcohol was purified over silica with 0-30% DCM in hexanes to afford the pure alcohol **S4** as a yellow oil (2.68 g, 70%). **^{1}H NMR** (400 MHz, CDCl3) δ 7.41 – 7.29 (m, 4H), 7.28 – 7.21 (m, 1H), 4.29 (t, J = 4.1 Hz, 1H), 3.05 (ddd, J = 11.6, 7.3, 4.2 Hz, 1H), 2.18 – 1.90 (m, 4H), 1.89 – 1.78 (m, 1H), 1.78 – 1.64 (m, 1H), 1.35 – 1.07 (m, 1H). NMR values matched previous reported spectra.[6]

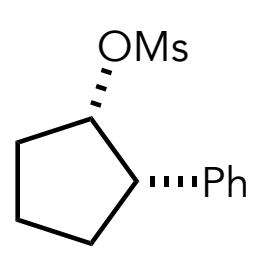

(±)-(1*S*,2*S*)-2-phenylcyclopentyl methanesulfonate (S5): To a 250 mL flame-dried round bottom flask was charged alcohol **S4** (2.63 g, 1 equiv, 16.2 mmol) and DCM (65 mL, 0.25 M). The flask was cooled to 0 ºC then triethylamine (6.78 mL, 3 equiv, 48.6 mmol) and mesyl chloride (1.90 mL, 1.5 equiv, 24.3 mmol) were added. The contents were gradually allowed to warm to room temperature. After 2 hours, the reaction was quenched with a 10% $NaHCO_3$ solution (40 mL), extracted with DCM (3 x 40 mL), dried with $MgSO_4$, filtered, and concentrated. The solvent was removed in *vacuo* then purified over silica with 0-15% ethyl acetate in hexanes to afford the product (**S5**) as a clear yellow liquid (2.69 g, 69%). **^{1}H NMR** (400 MHz, CDCl3) δ 7.40 – 7.32 (m, 4H), 7.32 – 7.25 (m, 1H), 5.15 (td, J = 4.0, 2.1 Hz, 1H), 3.22 (ddd, J = 12.1, 7.6, 4.3 Hz, 1H), 2.31 – 2.18 (m, 6H), 2.18 – 2.03 (m, 2H), 1.91 – 1.77 (m, 1H). **^{13}C NMR** (101 MHz, CDCl3) δ 138.4, 128.7, 128.4, 127.1, 88.3, 50.7, 37.3, 33.9, 27.7, 22.0. **HRMS** (ESI) calculated for $[C_{12}H_{20}NO_3S]^+$ $(M+NH_4)^+$ requires *m/z* 258.1158, found 258.1159.

(±)-((1*S*,2*R*)-2-azidocyclopentyl)benzene (S6): To a 250 mL flame-dried round bottom flask was sequentially charged mesylate **S5** (2.69 g, 1 equiv, 11.2 mmol), DMF (53 mL, 0.2 M), and sodium azide (2.91 g, 4 equiv, 44.8 mmol) and the contents were warmed to 100 ºC. After 3 hours, the reaction mixture was diluted with ethyl acetate (100 mL) and

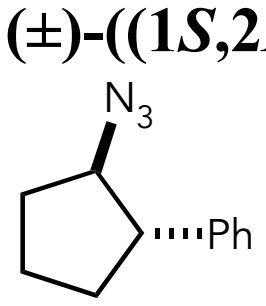

the organic layer was washed with H_2O (2 x 50 mL), brine (50 mL), then dried over $MgSO_4$, filtered, and concentrated. The crude azide was purified over silica with 10% DCM in pentanes to afford the pure azide (**S6**) as a clear and colorless liquid (1.22 g, 58%).**^{1}H NMR** (500 MHz, CDCl3) δ 7.32 (t, J = 7.6 Hz, 2H), 7.28 – 7.20 (m, 3H), 3.79 (q, J = 7.5 Hz, 1H), 2.99 (dt, J = 9.6, 8.1 Hz, 1H), 2.23 – 2.10 (m, 2H), 1.90 – 1.72 (m, 4H). NMR values matched previous reported spectra.[7]

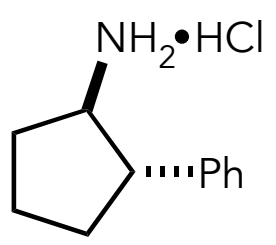

(±)-(1S,2R)-2-phenylcyclopentan-1-amine hydrochloride (S1): To a 100 mL flame-dried pear-shaped flask was charged azide **S6** (1.22 g, 1 equiv, 6.52 mmol) and Pd/C (1.39 g, 0.1 equiv, 0.65 mmol, 5% w/w) and the flask was sealed then evacuated and backfilled with N_2 three times. MeOH (32 mL, 0.2 M) was added via syringe. The reaction was sparged with H_2 for 1 minute, and then the N_2 line was removed, and the reaction was kept under a positive pressure of H_2 overnight. The reaction was filtered through a pad of celite and concentrated to ~1 mL MeOH. Concentrated HCl (37% w/w) was added to form the amine hydrochloride (**S1**). MeOH was added to azeotrope the remaining H_2O to afford the desired product as an orange solid (850 mg, 66%). **^{1}H NMR** (500 MHz, $CDCl_3$) δ 7.33 – 7.28 (m, 2H), 7.28 – 7.19 (m, 3H), 3.48 (q, *J* = 7.2 Hz, 1H), 3.18 (q, *J* = 8.3 Hz, 1H), 2.24 (ddt, *J* = 13.5, 10.1, 4.8 Hz, 2H), 2.04 – 1.75 (m, 4H). **^{13}C NMR** (126 MHz, $CDCl_3$) δ 141.0, 128.9, 127.4, 127.2, 59.5, 51.2, 32.9, 31.2, 22.7. **HRMS** (ESI) calculated for $[C_{11}H_{16}N]^+$ $(M+H)^+$ requires *m/z* 162.1127, found 162.1127. **Melting Point**: 141-143 ºC.

(±)-4-methoxy-*N*-((1*R*,2*S*)-2-phenylcyclopentyl)benzenesulfonamide (S7): Prepared according to **General Procedure E** and purified over silica with 0-30% ethyl acetate in hexanes to afford product **S7** as a white solid (135 mg, 81%). **^{1}H NMR** (500 MHz, $CDCl_3$) δ 7.55 – 7.48 (m, 2H), 7.22 – 7.12 (m, 3H), 7.03 – 6.96 (m, 2H), 6.82 – 6.76 (m, 2H), 4.43 (d, *J* = 6.0 Hz, 1H), 3.85 (s, 3H), 3.44 (dt, *J* = 15.8, 8.1 Hz, 1H), 2.73 (td, *J* = 10.0, 8.2 Hz, 1H), 2.24 – 2.14 (m, 1H), 2.12 – 2.01 (m, 1H), 1.82 – 1.72 (m, 2H), 1.68 – 1.56 (m, 2H). **^{13}C NMR** (126 MHz, $CDCl_3$) δ 162.6, 141.5, 131.6, 129.1, 128.6, 127.3, 126.7, 114.0, 61.3, 55.6, 52.5, 33.3, 32.2, 22.1. **HRMS** (ESI) calculated for $[C_{18}H_{20}NO_3S]^-$ $(M-H)^-$ requires *m/z* 330.1169, found 330.1167. **Melting Point**: 117-119 ºC.

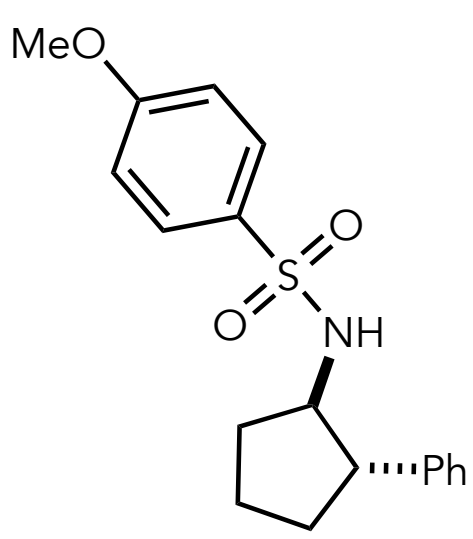

(±)-*N*-((1*R*,2*S*)-2-phenylcyclopentyl)methanesulfonamide (S8): Prepared according to **General Procedure E** on 0.556 mmol scale and purified over silica with 10-30% ethyl acetate in hexanes to afford product **S8** as a clear and colorless liquid (89 mg, 74%). **^{1}H NMR** (500 MHz, $CDCl_3$) δ 7.40 – 7.32 (m, 2H), 7.31 – 7.26 (m, 3H), 4.33 (d, *J* = 7.6 Hz, 1H), 3.77 (p, *J* = 9.7 Hz, 1H), 2.77 (td, *J* = 10.0, 8.1 Hz, 1H), 2.43 (s, 3H), 2.42 – 2.31 (m, 1H), 2.23 – 2.13 (m, 1H), 1.93 – 1.77 (m, 3H), 1.72 – 1.60 (m, 1H). **^{13}C NMR** (126 MHz, $CDCl_3$) δ 142.0, 128.9, 127.6, 127.2, 62.7, 52.9, 41.1, 33.9, 32.0, 21.8. **HRMS** (ESI) calculated for $[C_{12}H_{16}NO_2S]^-$ $(M-H)^-$ requires *m/z* 238.0907, found 238.0907.

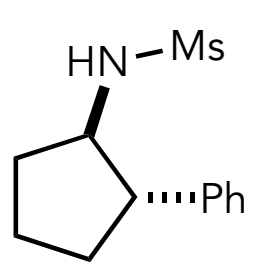

(±)-4-nitro-*N*-((1*R*,2*S*)-2-phenylcyclopentyl)benzenesulfonamide (S9): Prepared according to **General Procedure E** and purified over silica with 0-30% ethyl acetate in hexanes to afford product **S9** as an off-white, tan solid (140 mg, 80%). **^{1}H NMR** (500 MHz, $CDCl_3$) δ 8.10 – 8.05 (m, 2H), 7.70 – 7.62 (m, 2H), 7.20 – 7.09 (m, 3H), 7.00 – 6.94 (m, 2H), 4.71 (d, *J* = 7.0 Hz, 1H), 3.59 (p, *J* = 8.3 Hz, 1H), 2.70 (td, *J* = 10.2, 8.2 Hz, 1H), 2.37 – 2.27 (m, 1H), 2.11 (ddt, *J* = 13.2, 8.4, 6.5 Hz, 1H), 1.89 – 1.78 (m, 2H), 1.75 – 1.59

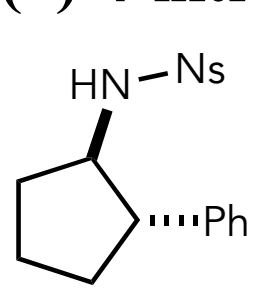

(m, 2H). **^{13}C NMR** (126 MHz, $CDCl_3$) δ 149.6, 146.0, 141.3, 128.7, 127.9, 127.2, 126.9, 123.9, 62.4, 52.8, 33.9, 32.3, 22.0. **HRMS** (ESI) calculated for $[C_{17}H_{17}N_2O_4S]^-$ $(M-H)^-$ requires *m/z* 345.0915, found 345.0918. **Melting Point**: 169-171 ºC.

(±)-1,1,1-trifluoro-*N*-((1*R*,2*S*)-2-phenylcyclopentyl)methanesulfonamide (S10): A flame dried 8 mL vial was charged with amine hydrochloride **S1** (197 mg, 1 equiv, 0.700 mmol), DCM (2.8 mL, 0.25 M) and triethylamine (244 µL, 2.5 equiv, 1.75 mmol). The flask was cooled to -78 ºC, and trifluoromethanesulfonic anhydride (118 uL, 1 equiv, 0.700 mmol) was added dropwise. The reaction was stirred vigorously at -78 ºC for 30 min and allowed to gradually warm up to room temperature. The reaction was then quenched with 5 mL H_2O, extracted with DCM (3 x 5 mL), dried over $MgSO_4$, filtered, and concentrated. The crude sulfonamide was purified over silica with 0-15% ethyl acetate in hexanes to afford product **S10** as a light-yellow oil (157 mg, 77%). **^{1}H NMR** (400 MHz, $CDCl_3$) δ 7.34 (tt, *J* = 6.9, 1.0 Hz, 2H), 7.30 – 7.18 (m, 3H), 4.95 (s, 1H), 3.90 (q, *J* = 8.5 Hz, 1H), 2.90 – 2.79 (m, 1H), 2.40 – 2.27 (m, 1H), 2.24 – 2.11 (m, 1H), 1.93 – 1.63 (m, 4H). **^{13}C NMR** (101 MHz, $CDCl_3$) δ 140.5, 128.8, 127.3, 127.2, 119.4 (q, *J* = 321.0 Hz), 63.2, 52.9, 33.4, 32.0, 21.8. **^{19}F NMR** (377 MHz, $CDCl_3$) δ -77.6. **HRMS** (ESI) calculated for $[C_{12}H_{13}F_3NO_2S]^-$ $(M-H)^-$ requires *m/z* 292.0625, found 292.0624.

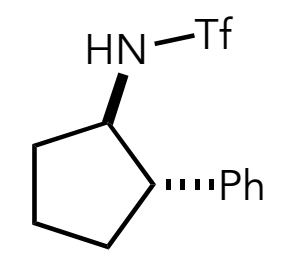

(±)-4-methyl-*N*-(trans-2-(4-methoxyphenyl)cyclopentyl)-benzenesulfonamide (S11): Prepared according to **General Procedure B** to give a white solid (300 mg, 82%). **^{1}H NMR** (400 MHz, CDCl3) δ 7.51 – 7.44 (m, 2H), 7.13 (d, *J* = 8.1 Hz, 2H), 6.89 (d, *J* = 8.7 Hz, 2H), 6.74 – 6.66 (m, 2H), 4.41 (m, 1H), 3.79 (d, *J* = 1.4 Hz, 3H), 3.37 (m, 1H), 2.67 (q, *J* = 9.5 Hz, 1H), 2.40 (s, 3H), 2.16 (m, 1H), 2.02 (m, 1H), 1.74 (m, 2H), 1.57 (m, 2H, overlapping with residual water). **^{13}C NMR** (126 MHz, $CDCl_3$) δ 158.5, 143.0, 137.1, 133.3, 129.5, 128.2, 127.1, 114.1, 61.5, 55.3, 51.8, 33.1, 32.2, 22.0, 21.6. **HRMS** (ESI) calculated for $[C_{19}H_{22}NO_3S]^-$ $(M-H)^-$ requires 344.1326, found 344.1325. **Melting Point**: 125-127 °C.

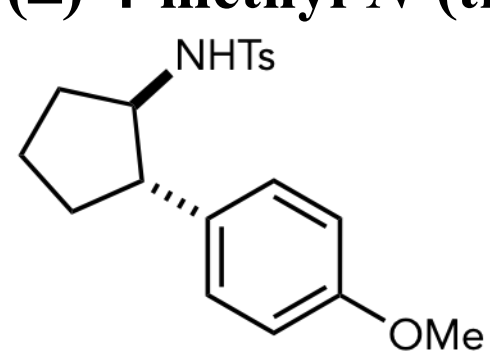

(±)-4-methyl-*N*-(trans-2-(2-methylphenyl)cyclopentyl)-benzenesulfonamide (S12): Prepared according to **General Procedure B** to give product as a while solid (320 mg, 92%). **^{1}H NMR** (500 MHz, $CDCl_3$) δ 8.33 – 8.27 (m, 2H), 7.92 (d, *J* = 8.0 Hz, 2H), 7.85 – 7.78 (m, 2H), 7.74 (m, 1H), 7.58 (d, *J* = 7.7 Hz, 1H), 5.38 (d, *J* = 6.1 Hz, 1H), 4.44 – 4.34 (m, 1H), 3.81 (q, *J* = 9.1 Hz, 1H), 3.17 (s, 3H), 2.99 (s, 3H), 2.90 (m, 1H), 2.85 (m, 1H), 2.60 – 2.45 (m, 2H), 2.34 (m, 1H), 2.21 (m, 1H). **^{13}C NMR** (126 MHz, $CDCl_3$) δ 143.1, 139.8, 137.2, 136.5, 130.4, 129.5, 127.1, 126.4, 126.3, 125.2, 60.3, 47.9, 33.1, 31.7, 22.1, 21.6, 19.8. **HRMS** (ESI) calculated for $[C_{19}H_{22}NO_2S]^-$ $(M-H)^-$ requires 328.1377, found 328.1377. **Melting Point**: 109-111 °C.

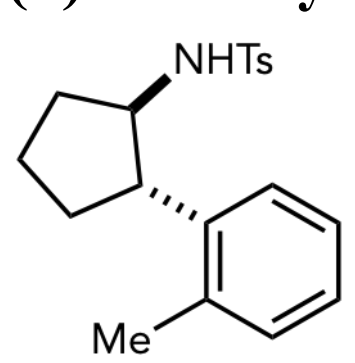

(±)-4-methyl-*N*-(trans-2-(4-chlorophenyl)cyclopentyl)-benzenesulfonamide (S13): Prepared according to **General Procedure B** to give white solid (320 mg, 86%). **^{1}H NMR** (500 MHz, $CDCl_3$) δ 7.43 (d, *J* = 8.3 Hz, 2H), 7.34 (d, *J* = 8.0 Hz, 2H), 7.07 (dd, *J* = 14.2, 8.0 Hz, 4H), 5.01 (d, *J* = 7.7 Hz, 1H), 3.60 – 3.49 (m, 1H), 2.79 (td, *J* = 9.9, 8.3 Hz, 1H), 2.37 (s, 3H), 2.23 – 2.13 (m, 1H), 2.12 – 2.02 (m, 1H), 1.82 – 1.73 (m, 2H), 1.66 – 1.50 (m, 2H). NMR values matched previously reported spectra.[4]

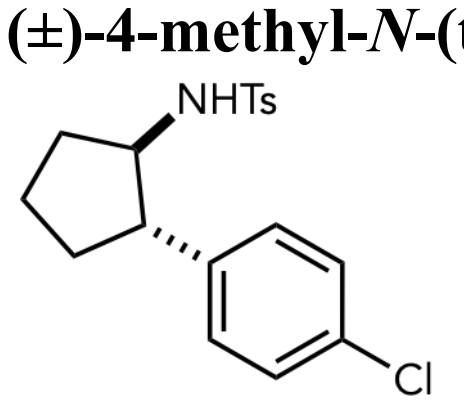

(±)-4-methyl-*N*-(trans-2-(4-trifluoromethylphenyl)cyclopentyl)-benzenesulfonamide (S14):

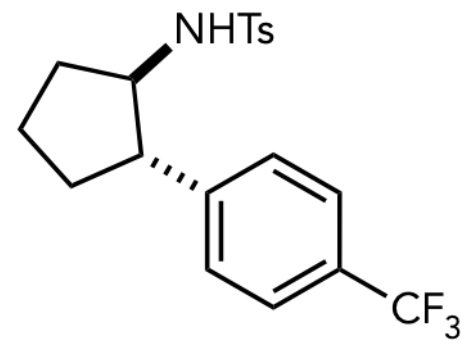

Prepared according to **General Procedure B** to give a white solid (254 mg, 63%). **^{1}H NMR** (500 MHz, $CDCl_3$) δ 7.46 – 7.40 (m, 2H), 7.34 (d, *J* = 8.0 Hz, 2H), 7.07 (m, 4H), 5.01 (d, *J* = 7.7 Hz, 1H), 3.55 (dq, *J* = 9.5, 7.8 Hz, 1H), 2.79 (td, *J* = 9.9, 8.3 Hz, 1H), 2.37 (s, 3H), 2.18 (dtd, *J* = 13.2, 7.5, 5.8 Hz, 1H), 2.12 – 2.02 (m, 1H), 1.82 – 1.73 (m, 2H), 1.66 – 1.50 (m, 2H). **^{13}C NMR** (126 MHz, $CDCl_3$) δ 146.2, 143.2, 137.2, 129.4, 128.9 (q, *J* = 32.4 Hz), 127.7, 126.9, 125.3 (q, *J* = 3.9 Hz), 124.4 (q, *J* = 271.7 Hz), 61.7, 52.2, 33.8, 32.4, 22.2, 21.4. **^{19}F NMR** (377 MHz, $CDCl_3$) δ -62.40. **HRMS** (ESI) calculated for $[C_{19}H_{24}F_3N_2O_2S]^+$ $(M+NH4)^+$ requires 401.1505, found 401.1498. **Melting Point**: 128-130 °C.

*Synthesis of (±)-1-benzyl 3-(3-((trans)-2-((4-methylphenyl)sulfonamido)cyclopentyl)benzyl) azetidine-1,3-dicarboxylate (***S17***):*

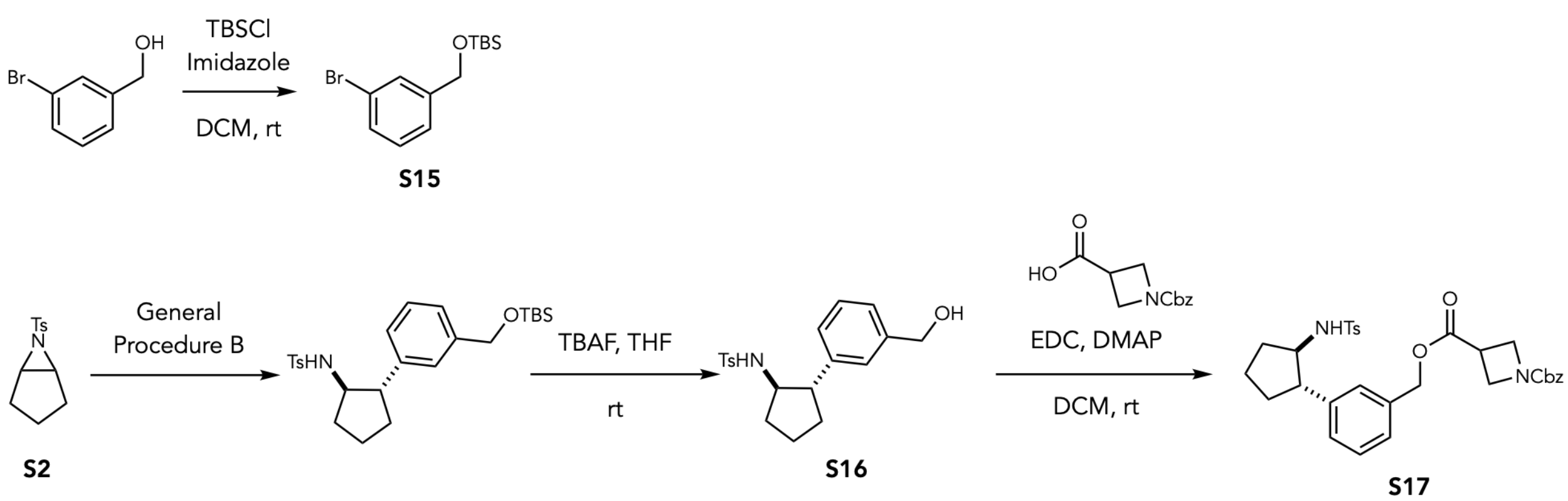

((3-bromobenzyl)oxy)(*tert*-butyl)dimethylsilane (S15): 3-bromo benzyl alcohol (3 g, 1 equiv, 16 mmol) was dissolved in DCM (80 mL) followed by addition of imidazole (2.2 g, 2 equiv, 32 mmol) and TBSCl (3.6 g, 1.5 equiv, 24 mmol) and the reaction was stirred for one hour. The reaction was then poured into saturated ammonium chloride and extracted with DCM twice. The combined organic layers were dried, concentrated and purified over silica with 0-10% EtOAc in hexanes to give the product as a colorless oil (4.5 g, 93%). **^{1}H NMR** (400 MHz, $CDCl_3$) δ 7.37 (s, 1H), 7.26 (m, 1H), 7.17 – 7.04 (m, 2H), 4.60 (s, 2H), 0.84 (s, 9H), 0.00 (s, 6H). NMR values matched previously reported spectra.[8]

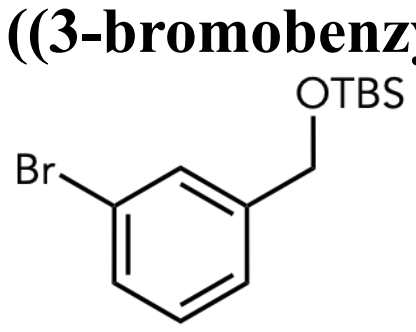

(±)-4-methyl-*N*-(trans-2-(3-hydroxymethylphenyl)cyclopentyl)-benzenesulfonamide (S16):

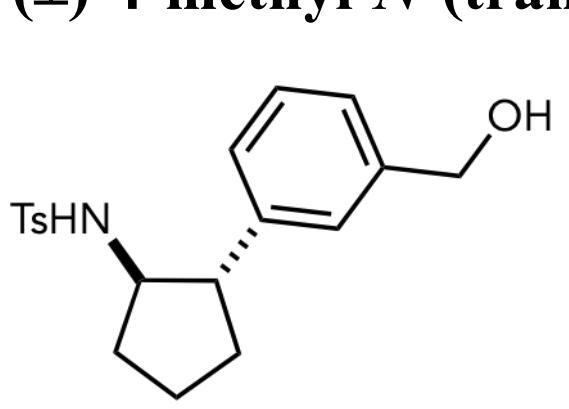

Prepared by a modification to **General Procedure B**. Aziridine opening was accomplished according to **General Procedure B** however before purification the residue (assuming quantitative yield) was dissolved in THF (10 mL) and TBAF (1.47 g, 5 equiv, 5.27 mmol) was added and the reaction was stirred overnight. In the morning the reaction was poured into saturated ammonium chloride and extracted three times with ethyl acetate. The combined organic layers were dried, concentrated and purified over silica with 0-50% EtOAc in hexanes to give the product as a colorless oil (321 mg, 88%). **^{1}H NMR** (500 MHz, $CDCl_3$) δ 7.52 – 7.46 (m, 2H), 7.20 – 7.10 (m, 4H), 7.03 (s, 1H), 6.93 (m, 1H), 4.77 (br s, 1H), 4.57 (s, 2H),

3.48 (q, J = 8.6 Hz, 1H), 2.75 (td, J = 10.0, 8.1 Hz, 1H), 2.39 (s, 3H), 2.09 (m, 2H), 1.82 – 1.70 (m, 3H), 1.58 (m, 2H). **^{13}C NMR** (126 MHz, $CDCl_3$) δ 143.0, 142.0, 141.3, 137.2, 129.5, 128.8, 127.1, 126.6, 126.0, 125.4, 65.2, 61.4, 52.4, 33.3, 32.3, 22.1, 21.5. **HRMS** (ESI) calculated for $[C_{19}H_{27}N_2O_3S]^+$ $(M+NH_4)^+$ requires *m/z* 363.1737, found 363.1731.

(±)-1-benzyl 3-(3-((trans)-2-((4-methylphenyl)sulfonamido)cyclopentyl)benzyl) azetidine-1,3-dicarboxylate (S17):

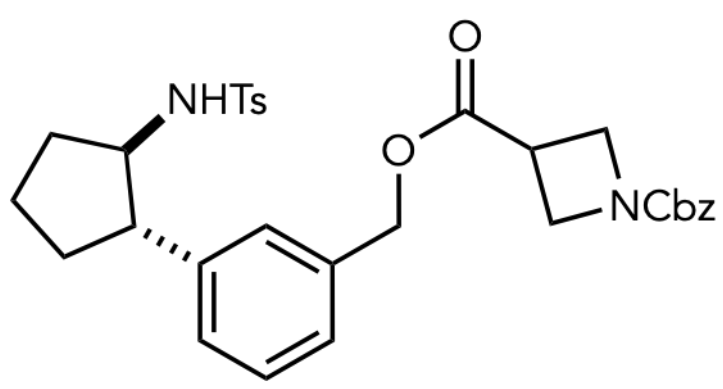

Azetidine carboxylic acid (141 mg, 1.2 equiv, 0.6 mmol), DMAP (73 mg, 1.2 equiv, 0.6 mmol) and EDC (133 mg, 1.5 equiv, 0.75 mmol) were dissolved in DCM (2.5 mL). After 15 minutes, alcohol **S16** (173 mg, 1 equiv, 0.5 mmol) was added and the reaction was stirred overnight at room temperature. The reaction was then poured in 1M HCl and extracted three times with ethyl acetate and the combined organic layers were washed with saturated sodium bicarbonate, dried, concentrated and purified over silica with 0-40% EtOAc in hexane to give the product as a colorless oil (153 mg, 54%). **^{1}H NMR** (400 MHz, $CDCl_3$) δ 7.46 – 7.39 (m, 2H), 7.33 – 7.22 (m, 5H), 7.15 – 6.97 (m, 4H), 6.92 (m, 2H), 5.02 (s, 2H), 4.99 (d, J = 3.7 Hz, 2H), 4.52 (s, 1H), 4.16 – 4.03 (m, 4H), 3.40 (m, 2H), 2.74 – 2.63 (m, 1H), 2.32 (s, 3H), 2.13 – 1.93 (m, 2H), 1.74 – 1.62 (m, 2H), 1.60 – 1.36 (m, 2H). **^{13}C NMR** (126 MHz, $CDCl_3$) δ 172.1, 156.3, 143.1, 142.3, 137.4, 136.5, 135.7, 130.0, 129.5, 129.0, 128.6, 128.2, 128.1, 127.4, 127.1, 126.6, 66.9, 66.9, 61.3, 52.4, 51.9, 33.2, 32.6, 32.3, 22.1, 21.6. **HRMS** (ESI) calculated for $[C_{31}H_{33}N_2O_6S]^-$ $(M-H)^-$ requires *m/z* 561.2065, found 561.2064.

*Synthesis of (±)-4-methyl-N-((trans)-2-(3-(pyrrolidine-1-carbonyl)phenyl)cyclopentyl) benzenesulfonamide (***S18***):*

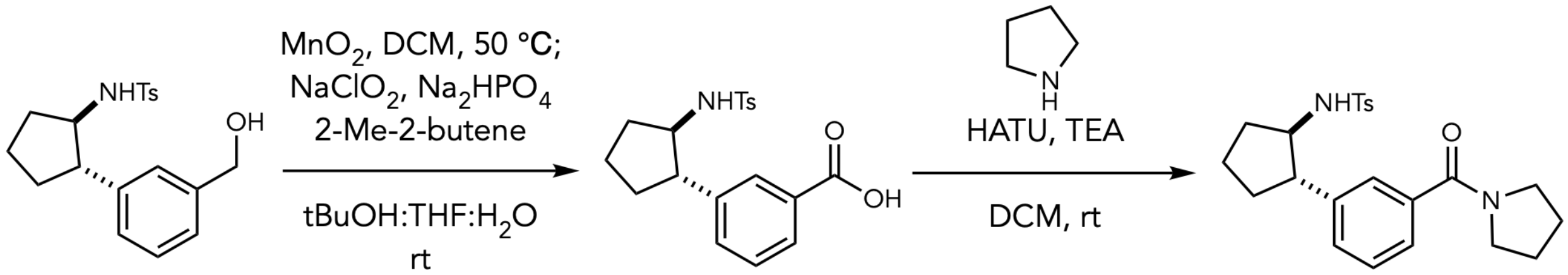

S16 **S18**

(±)-4-methyl-*N*-((trans)-2-(3-(pyrrolidine-1-carbonyl)phenyl)cyclopentyl) benzenesulfonamide (S18):

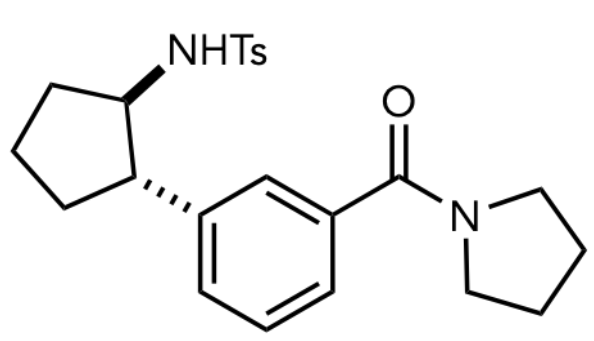

Alcohol **S16** (242 mg, 1 equiv, 0.7 mmol) was dissolved in DCM (10 mL) and activated MnO_2 (487 mg, 8 equiv, 5.6 mmol) was added. The vial was sealed and heated to 50 °C overnight. The reaction was then cooled to room temp before being filtered over a pad of celite and concentrated to afford the crude benzaldehyde. This aldehyde (assuming quantitative yield) was dissolved in a 1:1 mixture of THF:*t*BuOH (12 mL) and water (4 mL) followed by addition of NaH_2PO_4 (420 mg, 5 equiv, 3.5 mmol) and 2-Me-2-butene (982 mg, 20 equiv, 14.0 mmol) and stirred until the base was fully dissolved. Next, sodium chlorite (222 mg, 3.5 equiv, 2.45 mmol) was added and the light-yellow mixture was stirred until complete by TLC (~3 hours) then diluted with EtOAc. The layers were separated, and the aqueous layer was extracted twice more with EtOAc. The combined organic layers were dried, concentrated and the crude carboxylic acid (250 mg) was used directly in the next step.

The crude carboxylic acid (250 mg, 1 equiv, 0.7 mmol) was dissolved in DCM (2 mL) followed by the addition of HATU (317 mg, 1.2 equiv, 0.84 mmol) and TEA (0.194 mL, 2 equiv, 1.4 mmol). After 15 minutes, pyrrolidine (0.086 mL, 1.5 equiv, 1.04 mmol) was added and the reaction was stirred overnight at room temperature. The reaction was poured into 1M HCl and extracted three times with EtOAc. The combined organic layers were washed with saturated sodium bicarbonate, dried and concentrated. The residue was purified over silica with 0-50% EtOAc in hexanes to provide the desired amide as a sticky, colorless residue (230 mg, 80%). **^{1}H NMR** (500 MHz, $CDCl_3$) δ 7.47 (d, J = 7.9 Hz, 2H), 7.26 (d, J = 7.6 Hz, 1H), 7.20 (m, 1H), 7.11 (m, 3H), 7.00 (d, J = 7.8 Hz, 1H), 4.78 (s, 1H), 3.58 (br t, J = 7.0 Hz, 2 H), 3.48 (p, J = 7.9 Hz, 1H), 3.35 – 3.26 (m, 2H), 2.75 (m, 1H), 2.32 (s, 3H), 2.04 – 1.93 (m, 2H), 1.88 (m, 2H), 1.79 (m, 2H), 1.74 – 1.61 (m, 2H), 1.55 (m, 1H), 1.42 (m, 1H). **^{13}C NMR** (126 MHz, $CDCl_3$) δ 169.7, 143.2, 142.1, 137.6, 137.5, 129.6, 128.7, 128.5, 127.0, 126.3, 125.4, 61.4, 52.3, 49.7, 46.3, 33.2, 32.2, 26.5, 24.5, 22.3, 21.6. **HRMS** (ESI) calculated for $[C_{23}H_{27}N_2O_3S]^-$ $(M-H)^-$ requires *m/z* 411.1748, found 411.1747.

Synthesis of (±)-Ethyl (trans-trans)-3-((4-methylphenyl)sulfonamido)-2-phenylcyclopentane-1-carboxylate (**S22**)*:*

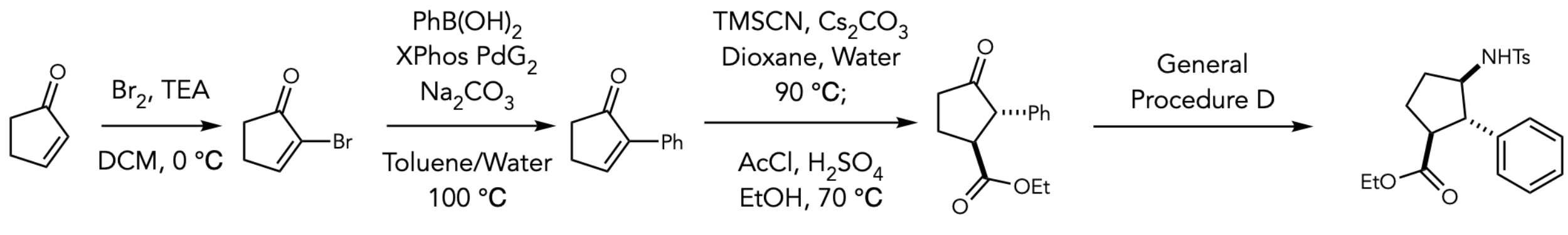

2-bromo-cyclopentone (S19): Cyclopentenone (3 g, 1 equiv, 36 mmol) was dissolved in DCM (30 mL) and cooled to 0 °C. A solution of bromine (1.88 mL, 1 equiv, 36 mmol) in DCM (30 mL) was added slowly over ten minutes then the reaction was stirred for 5 minutes. Next a solution of TEA (7.6 mL, 1.5 equiv, 55 mmol) in DCM (10 mL) was added and the reaction was allowed to warm to room temperature and stirred for 3 hours. The reaction was filtered over a pad of celite and concentrated. The residue was purified over silica with 10% EtOAc in hexanes to provide the product as a colorless oil (4.7 g, 80%). **^{1}H NMR** (400 MHz, $CDCl_3$) δ 7.73 (m, 1H), 2.65 (m, 2H), 2.46 (m, 2H). NMR values matched previous reported spectra.[9]

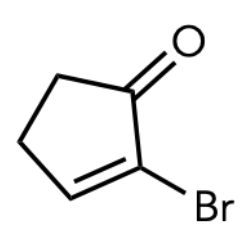

2-phenyl-cyclopentenone (S20): 2-bromo-cyclopentenone **S19** (3.32 g, 1 equiv, 20 mmol), XPhos PdG2 (400 mg, 2.5 mol%, 0.516 mmol), phenylboronic acid (3.77 g, 1.5 equiv, 31 mmol) and Na_2CO_3 (4.37 g, 2 equiv, 41 mmol) were added to a 250 mL round bottom flask. The flask was sealed and evacuated then backfilled with nitrogen. This was repeated two more times. Toluene (50 mL) and water (50 mL) were added, and the reaction was sparged with nitrogen for 5 minutes then heated to 100 °C overnight. The reaction was filtered over celite, and the layers were separated. The organic layer was washed with EtOAc, and the combined organic layers were dried, concentrated and purified over silica with 10% EtOAc in hexanes to give the product as a colorless oil (2.1 g, 64%). **^{1}H NMR** (400 MHz, $CDCl_3$) δ 7.83 (t, J = 2.9 Hz, 1H), 7.72 – 7.65 (m, 2H), 7.42 – 7.29 (m, 3H), 2.72 (m, 2H), 2.61 (m, 2H). NMR values matched previously reported spectra.[10]

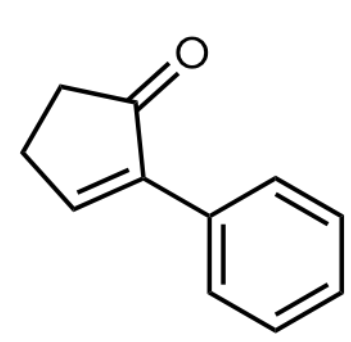

(±)-Ethyl (trans)-3-oxo-2-phenylcyclopentane-1-carboxylate (S21): 2-phenyl-cyclopentenone

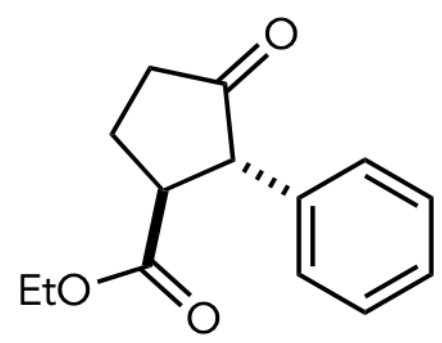

S20 (2.1 g, 1 equiv, 13 mmol) and Cs_2CO_3 (43 mg, 1 mol%, 0.13 mmol) were dissolved in dioxane (24 mL) followed by addition of TMSCN (2.5 mL, 1.5 equiv, 20 mmol) and water (1 mL, 4 equiv, 53 mmol) and the reaction was heated to 90 °C for one hour. After cooling to room temperature, water (5 mL) was added to quench the reaction and was extracted three times with EtOAc (all workup procedure must be conducted in well-ventilated hood, reaction generates toxic HCN gas). The combined organic layers were dried and concentrated to provide the crude trans-3-cyano-2-phenyl-cyclopentanone.

To a separate flask at 0 °C was added EtOH (15 mL), AcCl (6.1 mL) and H_2SO_4 (2.3 mL). After 10 minutes a solution of the crude cyclopentanone in EtOH (9 mL) was added and the reaction was sealed and heated to 70 °C overnight. After cooling to room temperature, the reaction was concentrated to ~10 mL under reduced pressure and quenched with saturated sodium bicarbonate. The suspension was extracted three times with EtOAc, and the combined organic layers were dried, concentrated and purified over silica with 0-20% EtOAc in hexanes to give the product as a light-yellow oil (1.3 g, 43%). **^{1}H NMR** (400 MHz, $CDCl_3$) δ 7.39–7.30 (m, 2H), 7.30–7.24 (m, 1H), 7.19–7.08 (m, 2H), 4.14 (q, J = 7.2 Hz, 2H), 3.72 (d, J = 11.2 Hz, 1H), 3.29–3.19 (m, 1H), 2.69–2.60 (m, 1H), 2.51–2.34 (m, 2H), 2.18–2.03 (m, 1H), 1.2 (t, J = 7.2 Hz, 3H). NMR values matched previously reported spectra.[11]

(±)-Ethyl (trans-trans)-3-((4-methylphenyl)sulfonamido)-2-phenylcyclopentane-1-carboxylate (S22):

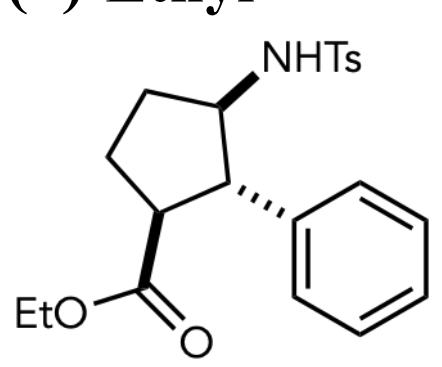

Prepared from the corresponding ketone **S21** (4.3 mmol scale) according to **General Procedure D** to give desired product as a colorless residue (450 mg, 33%). **^{1}H NMR** (500 MHz, $CDCl_3$) δ 7.50 – 7.44 (m, 2H), 7.22 – 7.09 (m, 5H), 7.00 – 6.92 (m, 2H), 4.83 (d, *J* = 7.0 Hz, 1H), 4.04 (q, *J* = 7.1 Hz, 2H), 3.64 (tt, *J* = 8.9, 7.1 Hz, 1H), 3.10 (t, *J* = 9.2 Hz, 1H), 2.81 (td, *J* = 9.4, 7.1 Hz, 1H), 2.39 (s, 3H), 2.26 – 2.16 (m, 1H), 2.15 – 1.94 (m, 2H), 1.72 (ddt, *J* = 13.0, 9.7, 8.0 Hz, 1H), 1.13 (t, *J* = 7.1 Hz, 3H). **^{13}C NMR** (126 MHz, $CDCl_3$) δ 175.1, 143.1, 139.6, 137.3, 129.5, 128.7, 127.5, 127.1, 127.0, 61.1, 60.9, 55.3, 49.5, 32.6, 27.1, 21.5, 14.1. **HRMS** (ESI) calculated for $[C_{21}H_{24}NO_4S]^-$ $(M-H)^-$ requires *m/z* 386.1432, found 386.1431. **Melting Point**: 129-131 °C.

(±)-Ethyl 5-(2-oxocyclopentyl)benzofuran-2-carboxylate (S23): Prepared according to **General**

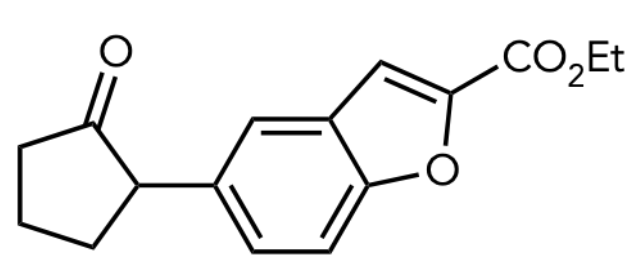

Procedure C on a 3.56 mmol scale to provide arylation product as sticky residue (766 mg, 78%). **^{1}H NMR** (500 MHz, $CDCl_3$) δ 7.55 (d, *J* = 8.7 Hz, 1H), 7.49 (m, 2H), 7.30 – 7.24 (m, 1H), 4.44 (q, *J* = 7.1 Hz, 2H), 3.43 (dd, *J* = 11.5, 8.2 Hz, 1H), 2.62 – 2.46 (m, 2H), 2.32 (m, 1H), 2.26 – 2.09 (m, 2H), 2.03 – 1.90 (m, 1H), 1.43 (t, *J* = 7.1 Hz, 3H). **^{13}C NMR** (126 MHz, $CDCl_3$) δ 217.9, 159.6, 154.9, 146.2, 134.1, 128.0, 127.3, 122.1, 113.7, 112.5, 61.6, 55.2, 38.4, 32.1, 20.9, 14.4. **HRMS** (ESI) for $[C_{16}H_{17}O_4]^+$ $(M+H)^+$ requires *m/z* 273.1121, found 273.1117.

(±)-Ethyl 5-((trans)-2-((4-methylphenyl)sulfonamido)cyclopentyl)benzofuran-2-carboxylate

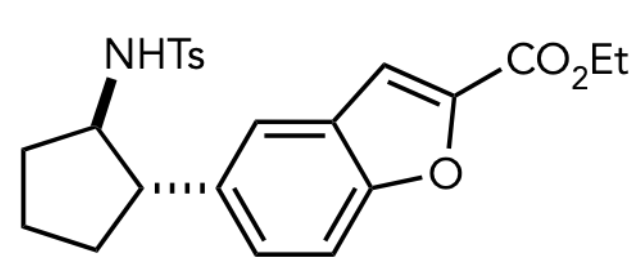

(S24): Prepared according to **General Procedure D** on a 2.57 mmol scale to provide sulfonamide product as a white solid (354 mg, 31%). **^{1}H NMR** (500 MHz, $CDCl_3$) δ 7.34 – 7.24 (m, 4H), 7.15 (d, *J* = 1.8 Hz, 1H), 7.00 (dd, *J* = 8.7, 1.8 Hz, 1H), 6.88 (d, *J* = 8.0 Hz, 2H), 4.55 (d, *J*

= 6.9 Hz, 1H), 4.40 (q, *J* = 7.1 Hz, 2H), 3.45 (tt, *J* = 9.4, 7.4 Hz, 1H), 2.74 (td, *J* = 10.1, 8.2 Hz, 1H), 2.23 (s, 3H), 2.20 – 2.12 (m, 1H), 2.09 – 1.98 (m, 1H), 1.73 (m, 2H), 1.65 – 1.47 (m, 2H), 1.38 (t, *J* = 7.1 Hz, 3H). **^{13}C NMR** (126 MHz, $CDCl_3$) δ 159.6, 154.8, 146.0, 143.0, 137.3, 137.2, 129.3, 127.2, 127.1, 127.0, 121.1, 113.6, 112.4, 62.0, 61.7, 52.4, 33.6, 32.6, 22.1, 21.4, 14.4. **HRMS** (ESI) calculated for $[C_{23}H_{26}NO_5S]^+$ $(M+H)^+$ requires *m/z* 428.1526, found 428.1523. **Melting Point**: 121-123 °C.

Synthesis of (±)-Methyl-1-(4-((trans)-2-((4-methylphenyl)sulfonamido)cyclopentyl)benzyl)-1H-indazole-3-carboxylate (**S27**)*:*

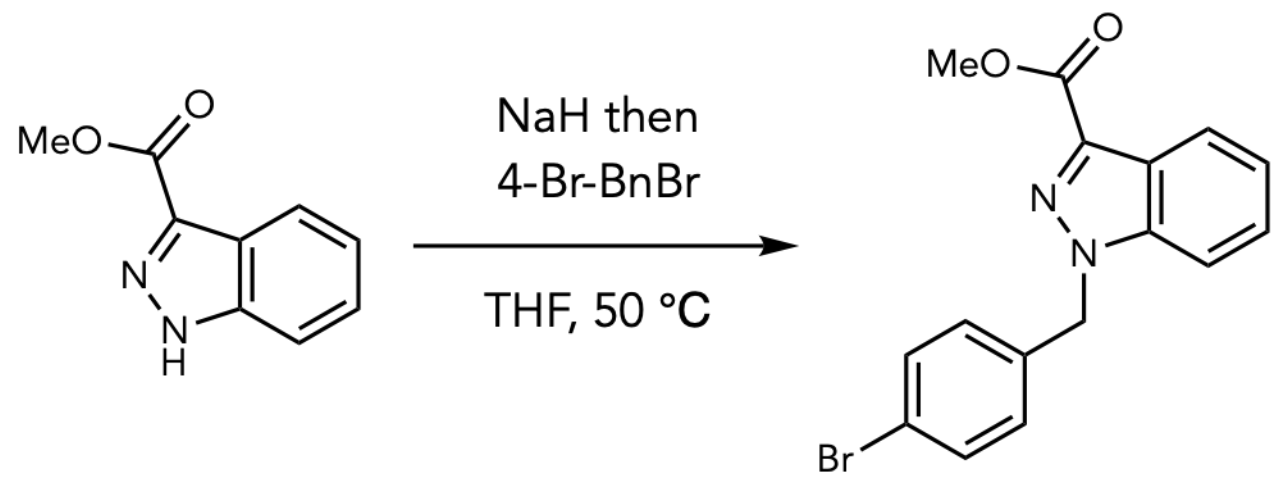

S25

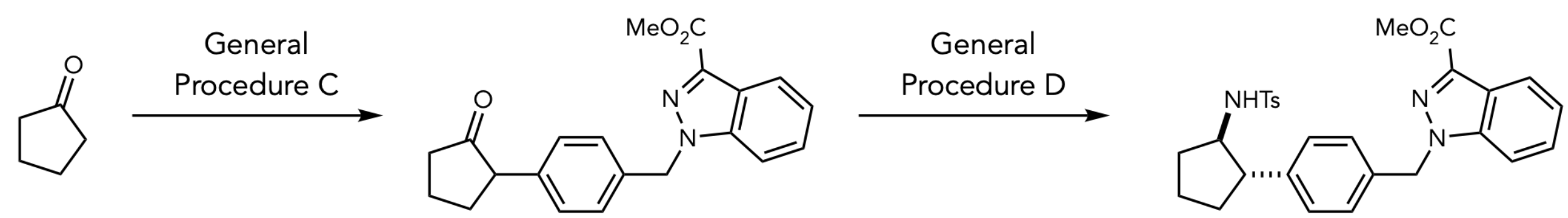

S26 **S27**

Methyl 1-(4-bromobenzyl)-1*H*-indazole-3-carboxylate (S25): To a solution of 1H-indazole-3-methylcarboxylate (2 g, 1 equiv, 11.35 mmol) in THF (48 mL) was added NaH (500 mg of 60% by weight, 1.1 equiv, 12.5 mmol). After 10 minutes, 4-bromo-benzylbromide (3.4 g, 1.2 equiv, 13.62 mmol) was added and the reaction was heated to 50 °C until complete by TLC. The reaction was cooled and poured into saturated ammonium chloride and extracted three times with EtOAc. The combined organic layers were dried, concentrated and purified over silica with 0-20% EtOAc in hexanes to give the product as a colorless solid (2.8 g, 72%). **^{1}H NMR** (500 MHz, $CDCl_3$) δ 8.25 (d, *J* = 7.8 Hz, 1H), 7.42 (d, *J* = 8.5 Hz, 2H), 7.39 (d, *J* = 7.5 Hz, 1H), 7.36 – 7.29 (m, 2H), 7.09 (d, *J* = 8.4 Hz, 2H), 5.65 (s, 2H), 4.06 (s, 3H). **^{13}C NMR** (126 MHz, $CDCl_3$) δ 162.98, 140.50, 135.29, 134.66, 132.04, 128.90, 127.27, 124.13, 123.42, 122.42, 122.19, 109.81, 53.40, 52.14. **HRMS** (ESI) calculated for $[C_{16}H_{14}BrN_2O_2]^+$ $(M+NH_4)^+$ requires *m/z* 345.0233, found 345.0229. **Melting Point**: 172 – 174 °C.

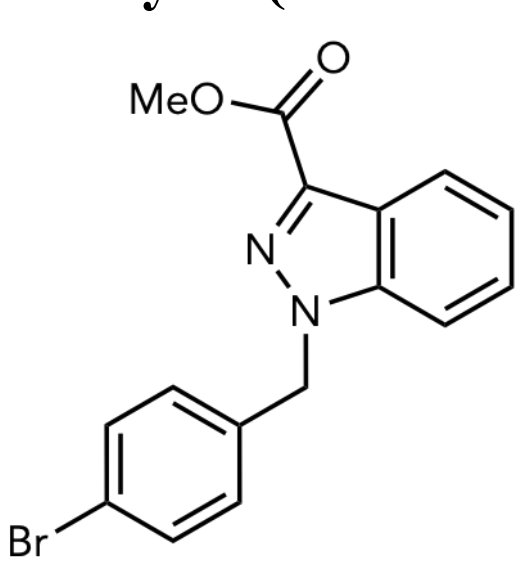

(±)-Methyl 1-(4-(2-oxocyclopentyl)benzyl)-1*H*-indazole-3-carboxylate (S26): Prepared according to **General Procedure C** on a 3.56 mmol scale to provide arylation product as a sticky residue (900 mg, 72%). **^{1}H NMR** (500 MHz, $CDCl_3$) δ 8.23 (dt, *J* = 8.2, 1.1 Hz, 1H), 7.39 – 7.35 (m, 2H), 7.30 (m, 1H), 7.23 – 7.18 (m, 2H), 7.17 – 7.08 (m, 2H), 5.67 (s, 2H), 4.05 (s, 3H), 3.31 – 3.23 (m, 1H), 2.48 – 2.40 (m, 2H), 2.25 (m, 1H), 2.17 – 1.99 (m, 2H), 1.97 – 1.83 (m, 1H). **^{13}C NMR** (126 MHz, $CDCl_3$) δ 217.7, 163.1, 140.6, 138.4, 135.0, 134.3, 128.7, 127.6, 127.1, 124.2, 123.3, 122.3, 110.2, 54.9, 53.8, 52.1, 38.4, 31.6, 20.8. **HRMS** (ESI) calculated for $[C_{21}H_{20}N_2O_3]^+$ $(M+H)^+$ requires *m/z* 349.1547, found 349.1544.

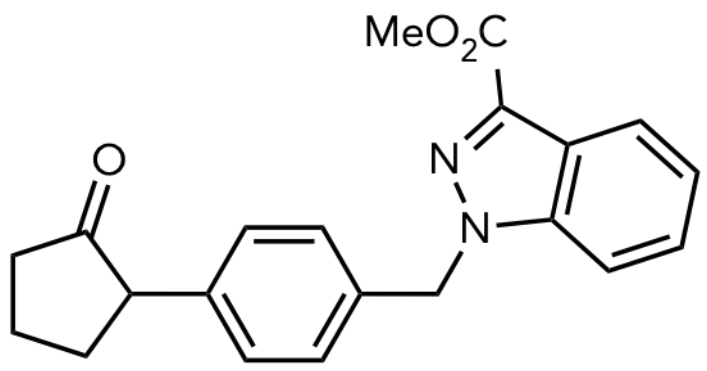

(±)-Methyl-1-(4-((trans)-2-((4-methylphenyl)sulfonamido)cyclopentyl)benzyl)-1*H*-indazole-3-carboxylate (S27): Prepared according to **General Procedure D** on a 2.55 mmol scale to provide desired sulfonamide as a white solid (284 mg, 22%). **^{1}H NMR** (500 MHz, $CDCl_3$) δ 8.19 (dt, *J* = 8.2, 1.0 Hz, 1H), 7.38 – 7.29 (m, 4H), 7.26 (m, 1H), 7.00 (d, *J* = 7.9 Hz, 2H), 6.92 – 6.82 (m, 4H), 5.65 – 5.53 (m, 2H), 4.32 (d, *J* = 6.1 Hz, 1H), 3.99 (s, 3H), 3.39 – 3.29 (m, 1H), 2.65 (m, 1H), 2.23 (s, 3H), 2.04 (m, 1H), 1.95 (m, 1H), 1.65 (m, 2H), 1.51 – 1.39 (m, 2H). **^{13}C NMR** (126 MHz, $CDCl_3$) δ 163.1, 143.2, 141.6, 140.6, 137.0, 135.1, 134.2, 129.5, 127.8, 127.7, 127.3, 127.1, 124.2, 123.4, 122.4, 110.1, 61.0, 53.9, 52.2, 52.2, 33.1, 32.4, 22.1, 21.5. **HRMS** (ESI) calculated for $[C_{28}H_{30}N_3O_4S]^+$ $(M+H)^+$ requires *m/z* 504.1952, found 504.1946. **Melting Point**: 82-84 °C.

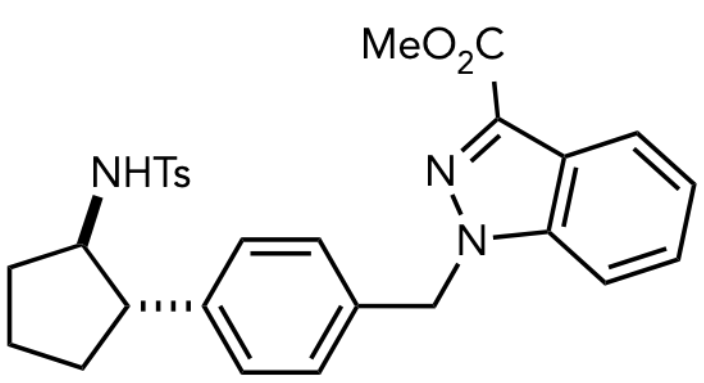

Synthesis of (±)-Methyl 5-((trans)-2-((4-methylphenyl)sulfonamido)cyclopentyl)-1-tosyl-1H-indole-3-carboxylate (**S30**)*:*

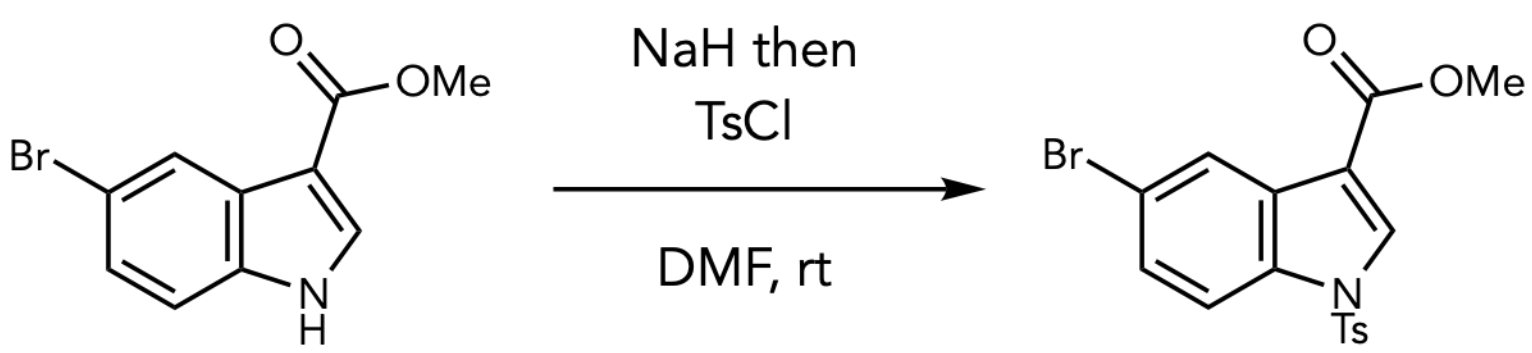

S28

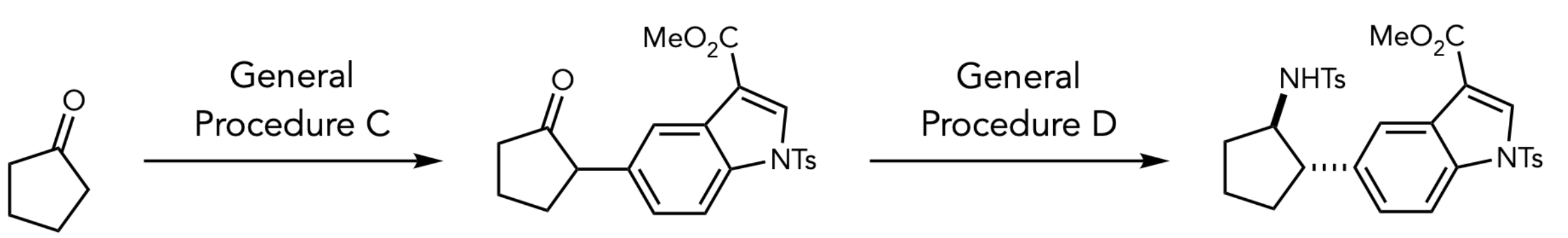

S29 **S30**

Methyl 5-bromo-1-tosyl-1*H*-indole-3-carboxylate (S28): Methyl-5-bromo-indole-3-carboxylate (2 g, 1 equiv, 7.9 mmol) was dissolved in DMF (20 mL) followed by portion wise addition of NaH (470 mg of 60% by weight, 1.5 equiv, 12 mmol). After 15 minutes, TsCl (2.3 g, 1.5 equiv, 12 mmol) was added and the reaction was stirred at room temperature overnight. The reaction was poured into saturated ammonium chloride and extracted three times with EtOAc. The combined organic layers were dried, concentrated and purified over silica

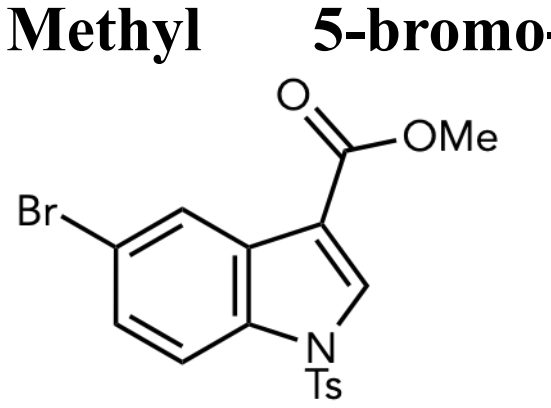

with 0-100% of a 9:1 EtOAc:MeOH mixture in hexanes to provide the protected indole as a white solid (2.5 g, 88%). **^{1}H NMR** (400 MHz, DMSO-d6) δ 8.49 (s, 1H), 8.14 (d, J= 2.0 Hz, 1H), 8.05 (d, J= 1.6 Hz, 1H), 8.03 (s, 1H), 7.94 (d, J= 8.9 Hz, 1H), 7.58 (dd, J= 8.9, 2.0 Hz, 1H), 7.43 (d, J= 8.1 Hz, 2H), 3.86 (s, 3H), 2.34 (s, 3H). NMR value matched previously reported spectra.[12]

(±)-Methyl 5-(2-oxocyclopentyl)-1-tosyl-1*H*-indole-3-carboxylate (S29): Prepared according to **General Procedure C** on 2.378 mmol scale to provide the arylated product as a colorless foam (686 mg, 70%).

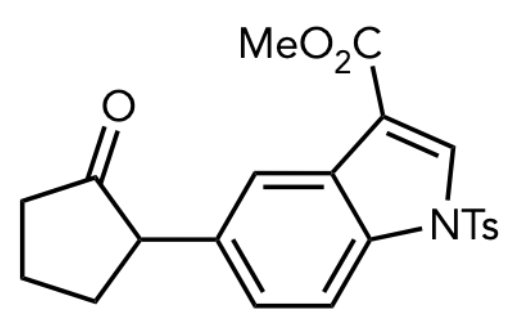

^{1}H NMR (500 MHz, $CDCl_3$) δ 8.17 (s, 1H), 7.87 (d, *J* = 1.8 Hz, 1H), 7.83 (d, *J* = 8.6 Hz, 1H), 7.80 – 7.70 (m, 2H), 7.24 – 7.15 (m, 2H), 7.11 (dd, *J* = 8.6, 1.8 Hz, 1H), 3.84 (s, 3H), 3.39 – 3.31 (m, 1H), 2.51 – 2.37 (m, 2H), 2.29 (s, 3H), 2.27 – 2.20 (m, 1H), 2.17 – 2.03 (m, 2H), 1.94 – 1.82 (m, 1H). **^{13}C NMR** (126 MHz, $CDCl_3$) δ 218.2, 164.1, 145.9, 135.0, 134.7, 133.9, 132.3, 130.3, 128.2, 127.3, 126.0, 121.5, 113.6, 113.3, 55.7, 51.7, 38.4, 32.3, 21.7, 20.9. **HRMS** (ESI) calculated for $[C_{22}H_{22}NO_5S]^+$ $(M+H)^+$ requires *m/z* 429.1479, found 429.1475.

(±)-Methyl 5-((trans)-2-((4-methylphenyl)sulfonamido)cyclopentyl)-1-tosyl-1*H*-indole-3-carboxylate (S30): Prepared according to **General Procedure D** on a 1.48 mmol scale to afford the desired sulfonamide as a white solid (236 mg, 31%).

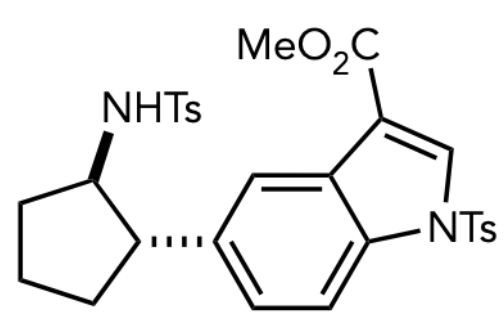

^{1}H NMR (500 MHz, $CDCl_3$) δ 8.22 (s, 1H), 7.87 – 7.81 (m, 2H), 7.74 (d, *J* = 8.6 Hz, 1H), 7.69 (d, *J* = 1.7 Hz, 1H), 7.34 – 7.24 (m, 4H), 7.03 (dd, *J* = 8.6, 1.8 Hz, 1H), 6.77 (d, *J* = 8.0 Hz, 2H), 4.76 – 4.69 (m, 1H), 3.91 (s, 3H), 3.53 (M, 1H), 2.80 (m, 1H), 2.35 (s, 3H), 2.23 (s, 3H), 2.21 – 2.15 (m, 1H), 2.04 (m, 1H), 1.82 – 1.71 (m, 2H), 1.68 (m, 1H), 1.57 (m, 1H). **^{13}C NMR** (126 MHz, $CDCl_3$) δ 164.0, 146.0, 142.8, 138.1, 137.1, 134.7, 133.8, 132.1, 130.3, 129.0, 128.0, 127.2, 126.8, 124.9, 120.6, 113.4, 113.2, 62.2, 52.6, 51.6, 33.5, 32.5, 22.1, 21.7, 21.4. **HRMS** (ESI) calculated for $[C_{29}H_{29}N_2O_6S_2]^-$ $(M-H)^-$ requires *m/z* 565.1473, found 565.1479. **Melting Point**: 101-103 °C.

Synthesis of (±)-4-methyl-N-((trans)-2-(2-(6-(trifluoromethyl)nicotinoyl)isoindolin-5-yl) cyclopentyl)benzenesulfonamide **(S33)**:

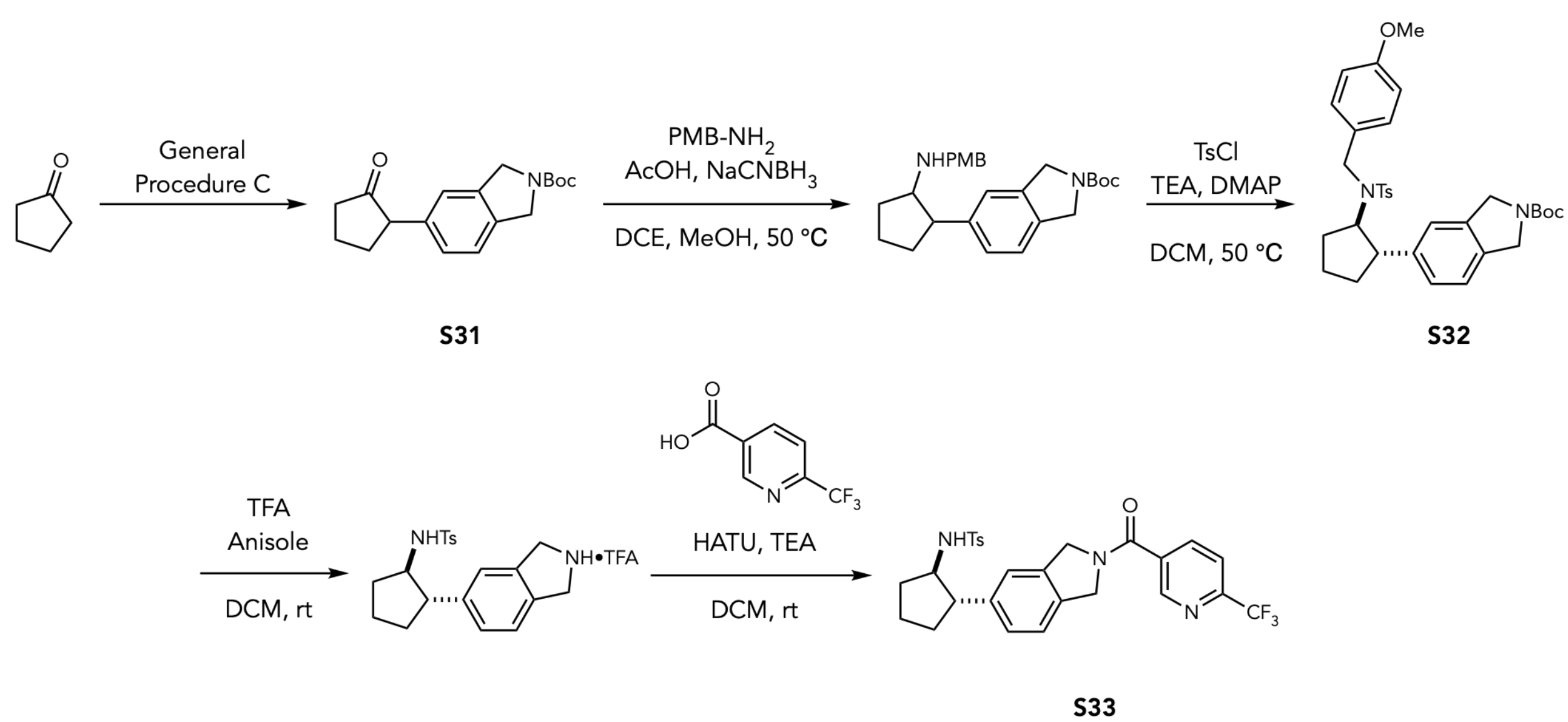

(±)-*tert*-butyl 5-(2-oxocyclopentyl)isoindoline-2-carboxylate (S31): Prepared according to **General Procedure C** on a 5.94 mmol scale to give product as a colorless foam (1.4 g, 78%). **^{1}H NMR** (500 MHz, $CDCl_3$) δ 7.20 (dd, *J* = 23.7, 7.8 Hz, 1H), 7.11 – 7.03 (m, 2H), 4.68 – 4.59 (m, 4H), 3.32 (dd, *J* = 11.5, 8.4 Hz, 1H), 2.56 – 2.42 (m, 2H), 2.29 (m, 1H), 2.22 – 2.03 (m, 2H), 1.94 (m, 1H), 1.51 (s, 9H). **^{13}C NMR** (126 MHz, $CDCl_3$, compound exists as mixture of rotamers, peaks listed are those for the major rotamer) δ 218.0, 154.6, 137.9, 137.5, 136.1, 127.5, 122.8, 122.5, 79.7, 55.4, 52.2, 52.0, 38.4, 32.0, 28.6, 20.9. **HRMS** (ESI) calculated for $[C_{18}H_{23}NaNO_3]^+$ $(M+Na)^+$ requires 324.1570, found 324.1564.

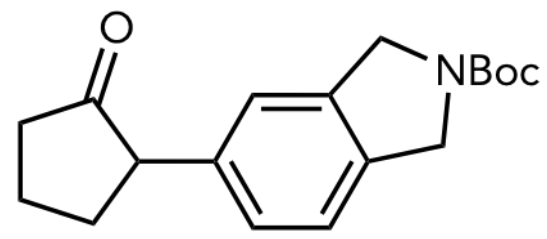

(±)-*tert*-butyl-5-((trans)-2-((N-(4-methoxybenzyl)-4-methylphenyl)sulfonamido)cyclopentyl)isoindoline-2-carboxylate (S32): Prepared according to the first 2 steps of **General Procedure D** (4.645 mmol scale). Following the tosyl protection step, rather than moving directly to the PMB deprotection, the crude product was purified over silica with 0-30% EtOAc in hexanes to afford the trans diastereomer as a fluffy white solid (988 mg, 36%). **^{1}H NMR** (500 MHz, $CDCl_3$, mixture of rotamers) δ 7.45 (m, 2H), 7.09 (m, 4H), 6.95 (m, 1H), 6.81 – 6.67 (m, 3H), 6.49 (m, 1H), 4.54 (d, *J* = 22.6 Hz, 2H), 4.43 – 4.34 (m, 3H), 4.28 – 4.18 (m, 1H), 4.12 (m, 1H), 3.72 (s, 3H), 2.95 – 2.84 (m, 1H), 2.32 (d, *J* = 2.5 Hz, 3H), 1.84 (m, 1H), 1.74 (m, 1H), 1.65 – 1.32 (m, 13H). **^{13}C NMR** (126 MHz, $CDCl_3$, mixture of rotamers, peaks given are for the major rotamer) δ 159.0, 154.6, 142.9, 141.8, 137.9, 137.3, 134.9, 130.4, 129.4, 129.4, 127.3, 126.7, 122.5, 121.5, 113.7, 79.7, 65.2, 55.3, 52.2, 51.9, 48.2, 47.5, 33.8, 28.6, 28.6, 22.3, 21.5. **HRMS** (ESI) calculated for $[C_{33}H_{41}N_2O_5S]^+$ $(M+H)^+$ requires *m/z* 577.2731, found 577.2728. **Melting Point**: 164-166 °C.

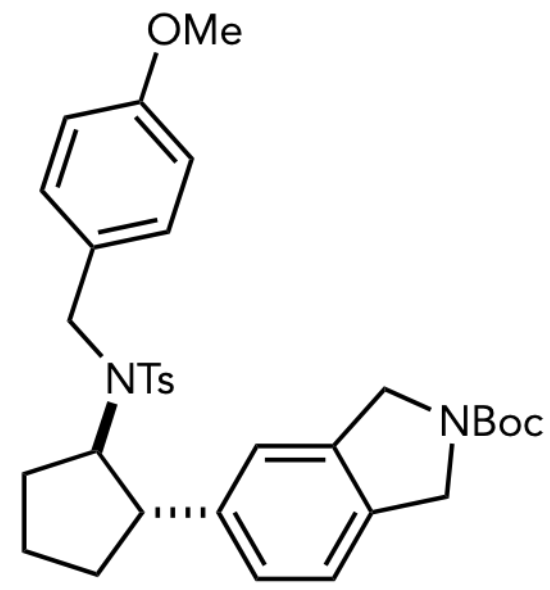

(±)-4-methyl-*N*-((trans)-2-(2-(6-(trifluoromethyl)nicotinoyl)isoindolin-5-yl)cyclopentyl)benzenesulfonamide (S33): PMB-protected sulfonamide **S32** (500 mg, 1 equiv, 0.867 mmol) was dissolved in DCM (5 mL). Anisole (281 mg, 3 equiv, 2.6 mmol) and TFA (4.9 g, 50 equiv, 43 mmol) were added and the reaction was stirred at room temperature for 3 hours before being concentrated under reduced pressure. Residual TFA was removed by azeotroping with toluene three times to provide the crude TFA salt as a tan solid.

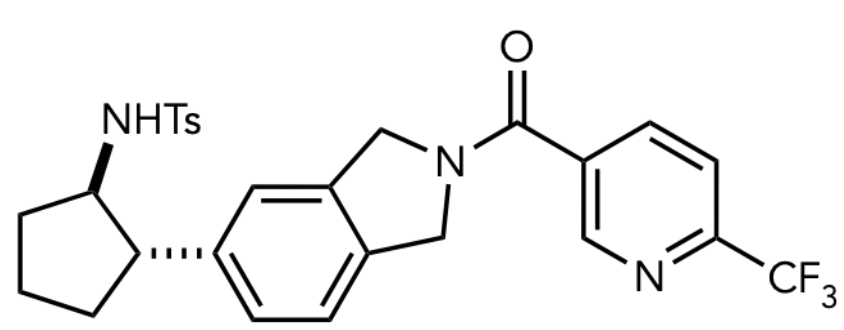

Assuming quantitative yield, in a separate flask 2-trifluoromethyl-pyridine-5-carboxylic acid was dissolved in DMF (4 mL) followed by addition of HATU (389 mg, 1.2 equiv, 1.02 mmol) and TEA (0.238 mL, 2 equiv, 1.7 mmol). After 15 minutes, the crude TFA salt was dissolved in a minimal amount of DMF and added to the carboxylic acid flask and stirred at room temperature overnight. The reaction was poured into 1M HCl and extracted three times with EtOAc. The combined organic layers were dried, concentrated and purified over silica with 0-50% EtOAc in hexanes to provide the product as an off-white solid (359 mg, 79%). **^{1}H NMR** (500 MHz, $CDCl_3$, mixture of rotamers) δ 8.95 (s, 1H), 8.13 – 8.07 (m, 1H), 7.81 (d, *J* = 8.0 Hz, 1H), 7.58 – 7.50 (m, 2H), 7.16 (m, 2H), 7.05 – 6.98 (m, 2H), 6.91 (d, *J* = 44.1 Hz, 1H), 4.98 (s, 1H), 4.94 – 4.83 (m, 1H), 4.81 – 4.62 (m, 3H), 3.60 – 3.47 (m, 1H), 2.78 (m, 1H), 2.38 (d, *J* = 22.2 Hz, 3H (rotameric -C*H$_3$*), 2.13 – 1.97 (m, 2H), 1.80 – 1.66 (m, 2H), 1.66 – 1.38 (m, 2H). **^{13}C NMR** (126 MHz, $CDCl_3$, mixture of rotamers, shifts provided correspond to the major rotamer) δ 166.3, 149.3 (q, *J* = 35.1 Hz), 148.1, 143.1, 142.3, 137.6, 136.4, 136.0, 135.1, 134.0, 129.4, 127.3, 127.1, 122.6, 121.7,

121.1 (q, J = 274.2 Hz) 120.5 (m, J = 2.6 Hz), 61.3, 54.7, 52.6, 52.3, 33.1, 32.5, 22.1, 21.5. ^{19}F NMR (377 MHz, $CDCl_3$) δ -68.15. **^{19}F NMR** (377 MHz, $CDCl_3$) δ -68.15. **HRMS** (ESI) calculated for $[C_{27}H_{25}F_3N_3O_3S]^-$ $(M-H)^-$ requires *m/z* 528.1574, found 528.1571. **Melting Point**: 102-104 °C.

Synthesis of allylic substrates **(S36–S38)**:

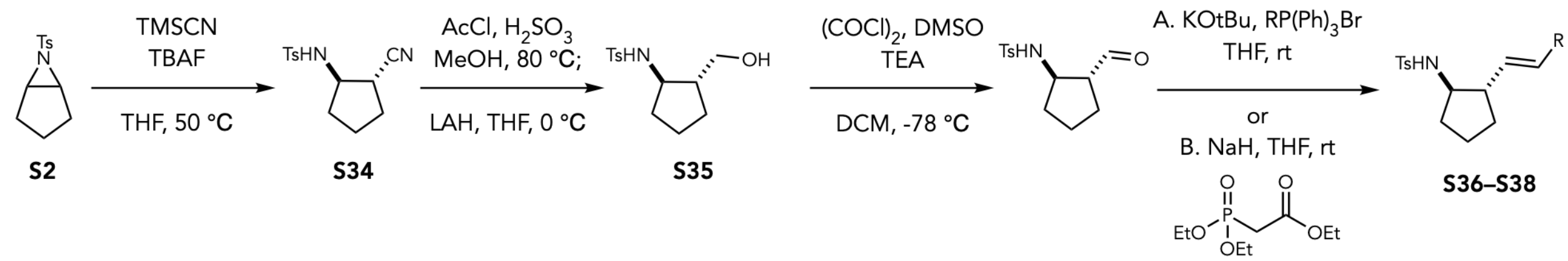

(±)-*N*-((trans)-2-cyanocyclopentyl)-4-methylbenzenesulfonamide (S34): 6-tosyl-6-azabicyclo[3.1.0]hexane **S2** (1 g, 1 equiv, 4.2 mmol) was dissolved in THF (35 mL). TMSCN (0.53 mL, 1 equiv, 4.2 mmol) and TBAF (0.84 mL of 1M solution, 0.2 equiv, 0.84 mmol) were added and the vial was heated to 50 °C overnight. The reaction was then cooled and concentrated under reduced pressure. The residue was then purified over silica with 40% EtOAc in hexane to afford the product as a colorless oil (835 mg, 75%). **^{1}H NMR** (400 MHz, $CDCl_3$) δ 7.85 (d, J = 8.18 Hz, 2H) 7.35 (d, J = 8.03 Hz, 2H), 5.45 (d, J = 6.57 Hz, 1H), 3.90−3.70 (m, 1H), 3.00−2.75 (m, 1H), 2.45 (s, 3H), 2.30-1.60 (m, 5H), 1.60−1.40 (m, 1H). NMR values matched previously reported spectra.[13]

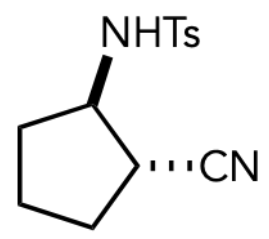

(±)-*N*-((trans)-2-(hydroxymethyl)cyclopentyl)-4-methylbenzenesulfonamide (S35): Nitrile **S34** (1.8 g, 1 equiv, 6.8mmol) was added to a solution of AcCl (7.3 mL, 15 equiv, 100 mmol) and H_2SO_4 (1.8 mL, 5 equiv, 34 mmol) in MeOH (25 mL). A reflux condenser was fitted, and the reaction was refluxed at 80 °C overnight. The next day, the reaction was cooled and poured into EtOAc (~400 mL) and quenched by addition of saturated sodium bicarbonate. The layers were separated, and the aqueous layer was extracted two more times with EtOAc. The combined organic layers were dried and concentrated to provide the crude methyl ester, used directly in the next step.

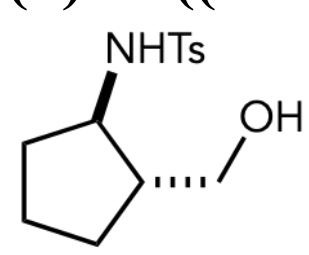

Assuming quantitative yield, the residue was dissolved in THF (50 mL) and cooled to 0 °C. LAH (8.4 mL of 2M solution, 2.5 equiv, 17 mmol) was added slowly. After stirring for 30 minutes, TLC showed complete consumption of starting material, and the reaction was carefully quenched by dropwise addition of saturated ammonium chloride. The suspension was poured into EtOAc, and the layers were separated. The aqueous layer was extracted twice more with EtOAc, and the combined organic layers were dried, concentrated and purified over silica with 0-50% EtOAc in hexanes to afford the alcohol as a sticky residue (1.2 g, 66%). **^{1}H NMR** (400 MHz, $CDCl_3$) δ 7.77 (d, J = 8.2 Hz, 2H), 7.31 (d, J = 7.9 Hz, 2H), 5.05 (d, J = 6.8 Hz, 1H), 3.70 (dd, J = 10.9, 4.8 Hz, 1H), 3.50 (dd, J = 11.0, 6.8 Hz, 1H), 3.27 (p, J = 7.8 Hz, 1H), 2.43 (s, 3H), 2.08 (br s, 1H), 1.99 – 1.86 (m, 1H), 1.77 (m, 2H), 1.62 (m, 1H), 1.56 – 1.45 (m, 1H), 1.38 (m, 1H), 1.32 – 1.22 (m, 1H). NMR values matched previously reported spectra.[13]

(±)-4-methyl-*N*-((trans)-2-vinylcyclopentyl)benzenesulfonamide (S36): To a vial containing

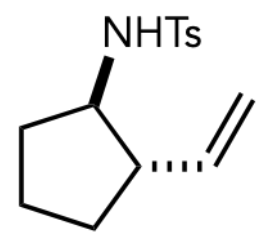

oxalyl chloride (0.211 mL, 1.3 equiv, 2.41 mmol) in DCM (10 mL) was added DMSO (0.395 mL, 3 equiv, 5.57 mmol) at -78 °C. After 10 minutes a solution of alcohol (500 mg, 1 equiv, 1.86 mmol) in DCM (5 mL) was added and the reaction was stirred at -78 °C for one hour. Next, TEA (1 mL, 4 equiv, 7.43 mmol) was added and the reaction was stirred at -78 °C for 30 minutes before being allowed to warm to room temperature. Once warmed to room temperature, the reaction was poured into 1M HCl and diluted with DCM (~400 mL). The layers were separated, and the organic layer was washed with saturated sodium bicarbonate then dried and concentrated to provide the crude aldehyde.

In a separate flask was add methyltriphenylphosphonium bromide (1 g, 1.5 equiv, 2.8 mmol) followed by addition of THF (7 mL). KOtBu (313 mg, 1.5 equiv, 2.8 mmol was added and the yellow solution was stirred at room temperature for 10 minutes. Next a solution of the crude aldehyde in THF (4 mL) was added and the reaction was stirred at room temperature with monitoring by TLC. When complete (~1 hour), the reaction was poured into saturated ammonium chloride and extracted three times with EtOAc. The combined organic layers were dried, concentrated and purified over silica with 0-20% EtOAc in hexanes to provide the desired product as a colorless oil (376 mg, 76%). **^{1}H NMR** (500 MHz, $CDCl_3$) δ 7.77 – 7.71 (m, 2H), 7.31 – 7.26 (m, 2H), 5.50 (ddd, *J* = 17.1, 10.2, 8.0 Hz, 1H), 5.01 – 4.89 (m, 2H), 4.54 – 4.44 (m, 1H), 3.13 (m, 1H), 2.43 (s, 3H), 2.19 (p, *J* = 8.6 Hz, 1H), 2.02 – 1.91 (m, 1H), 1.82 (m, 1H), 1.71 – 1.52 (m, 2H), 1.37 (m, 2H). **^{13}C NMR** (126 MHz, $CDCl_3$) δ 143.4, 139.1, 137.6, 129.6, 127.4, 116.1, 59.6, 51.1, 32.8, 29.6, 21.7, 21.6. **HRMS** (ESI) calculated for $[C_{14}H_{18}NO_2S]^-$ $(M-H)^-$ requires *m/z* 264.1064, found 264.1062.

(±)-4-methyl-*N*-((trans)-2-((*E*)-styryl)cyclopentyl)benzenesulfonamide (S37): To a vial

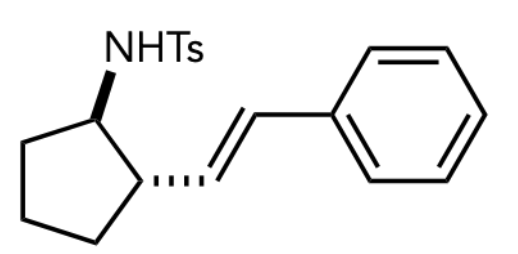

containing oxalyl chloride (0.211 mL, 1.3 equiv, 2.41 mmol) in DCM (10 mL) was added DMSO (0.395 mL, 3 equiv, 5.57 mmol) at -78 °C. After 10 minutes a solution of alcohol (500 mg, 1 equiv, 1.86 mmol) in DCM (5 mL) was added and the reaction was stirred at -78 °C for one hour. Next, TEA (1 mL, 4 equiv, 7.43 mmol) was added and the reaction was stirred at -78 °C for 30 minutes before being allowed to warm to room temperature. Once warmed to room temperature, the reaction was poured into 1M HCl and diluted with DCM (~400 mL). The layers were separated, and the organic layer was washed with saturated sodium bicarbonate then dried and concentrated to provide the crude aldehyde.

In a separate flask was add benzyltriphenylphosphonium bromide (4.0 g, 5 equiv, 9.3 mmol) followed by addition of THF (10 mL). KO*t*Bu (835 mg, 4.0 equiv, 7.44) mmol was added and the yellow solution was stirred at room temperature for 10 minutes. Next a solution of the crude aldehyde in THF (10 mL) was added and the reaction was stirred at room temperature with monitoring by TLC. When complete (~1 hour), the reaction was poured into saturated ammonium chloride and extracted three times with EtOAc. The combined organic layers were dried, concentrated and purified over silica with 0-20% EtOAc in hexanes to provide the desired *E*-alkene as a colorless oil (589 mg, 65%) with a small (<6%) impurity of the *Z*-alkene. **^{1}H NMR** (500 MHz, $CDCl_3$) δ 7.74 – 7.71 (m, 2H), 7.30 (m, 2H), 7.25 – 7.22 (m, 1H), 7.21 – 7.18 (m, 2H), 7.13 (d, *J* = 8.0 Hz, 2H), 6.29 (d, *J* = 15.8 Hz, 1H), 5.77 (dd, *J* = 15.8, 8.4 Hz, 1H), 4.87 (d, *J* = 6.7 Hz, 1H), 3.26 (tt, *J* = 8.7, 7.1 Hz, 1H), 2.40 – 2.35 (m, 1H), 2.33 (s, 3H), 2.10 (m, 1H), 1.91 (m, 1H), 1.79 – 1.60 (m, 2H), 1.57 – 1.38 (m, 2H). **^{13}C NMR** (126 MHz, $CDCl_3$) δ 143.2, 137.5, 137.1, 131.2,

130.8, 129.6, 129.6, 128.5, 127.3, 126.2, 60.0, 50.4, 33.2, 30.2, 21.8, 21.5. **HRMS** (ESI) for $[C_{20}H_{22}NO_2S]^-$ $(M\text{-}H)^-$ requires *m/z* 340.1377, found 340.1378.

(±)-Ethyl (*E*)-3-((trans)-2-((4-methylphenyl)sulfonamido)cyclopentyl)acrylate (S38): To a

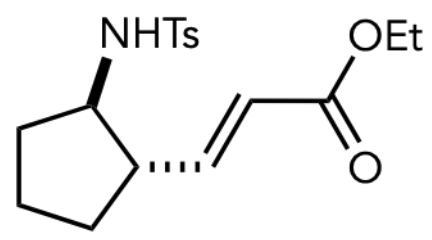

vial containing oxalyl chloride (0.211 mL, 1.3 equiv, 2.41 mmol) in DCM (10 mL) was added DMSO (0.395 mL, 3 equiv, 5.57 mmol) at -78 °C. After 10 minutes a solution of alcohol (500 mg, 1 equiv, 1.86 mmol) in DCM (5 mL) was added and the reaction was stirred at -78 °C for one hour. Next, TEA (1 mL, 4 equiv, 7.43 mmol) was added and the reaction was stirred at -78 °C for 30 minutes before being allowed to warm to room temperature. Once warmed to room temperature, the reaction was poured into 1M HCl and diluted with DCM (~400 mL). The layers were separated, and the organic layer was washed with saturated sodium bicarbonate then dried and concentrated to provide the crude aldehyde.

In a separate flame dried flask was added NaH (90 mg of 60% by weight, 1.2 equiv, 2.23 mmol) followed by THF (15 mL) then triethyl phosphonoacetate (0.554 mL, 1.5 equiv, 2.79 mmol). After stirring at room temperature for 15 minutes, a solution of the crude aldehyde in THF (5 mL) was added and the reaction was stirred at room temperature with monitoring by TLC. When complete (~1 hour) the reaction was poured into saturated ammonium chloride and extracted three times with EtOAc. The combined organic layers were dried, concentrated and purified over silica with 0-20% EtOAc in hexanes to afford the desired ester as a white solid (573 mg, 91%). **^{1}H NMR** (400 MHz, $CDCl_3$) δ 7.77 – 7.69 (m, 2H), 7.33 – 7.24 (m, 2H), 6.63 (dd, *J* = 15.6, 8.4 Hz, 1H), 5.68 (dd, *J* = 15.6, 1.1 Hz, 1H), 4.73 (d, *J* = 7.5 Hz, 1H), 4.16 (q, *J* = 7.1 Hz, 2H), 3.35 (m, *J* = 7.8 Hz, 1H), 2.42 (m, 4H), 1.92 (m, 2H), 1.75 – 1.56 (m, 2H), 1.50 – 1.35 (m, 2H), 1.29 (t, *J* = 7.1 Hz, 3H). **^{13}C NMR** (126 MHz, $CDCl_3$) δ 166.1, 148.6, 143.6, 137.6, 129.8, 127.3, 122.1, 60.3, 59.6, 49.4, 33.3, 29.8, 22.1, 21.6, 14.3. **HRMS** (ESI) calculated for $[C_{17}H_{22}NO_4S]^-$ $(M\text{-}H)^-$ requires *m/z* 336.1275, found 336.1278. **Melting Point**: 82-84 °C.

Synthesis of enantiopure **(S40):**

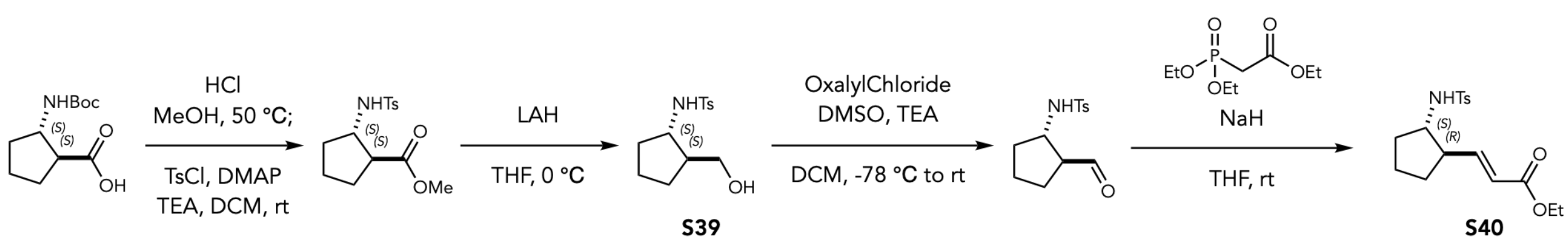

(-)-*N*-((1*S*,2*S*)-2-(hydroxymethyl)cyclopentyl)-4-methylbenzenesulfonamide (S39): (1*S*,2*S*)-

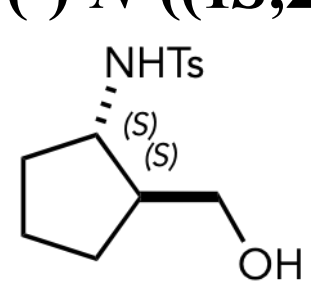

2-((*tert*-butoxycarbonyl)amino)cyclopentane-1-carboxylic acid (1 g, 1 equiv, 4.3 mmol) was dissolved in MeOH (16 mL) followed by addition of concentrated HCl (1.8 mL). The reaction was then stirred and heated at 50 °C overnight. In the morning the reaction was concentrated and residual HCl was removed by azeotroping with toluene three times to afford the HCl salt of the methyl ester as a white solid. The was directly dissolved in DCM (16 mL) followed by addition of DMAP (106 mg, 0.2 equiv, 0.86 mmol), TEA (1.82 mL, 3 equiv, 13 mmol) and TsCl (1.25 g, 1.5 equiv, 6.5 mmol) and stirred overnight at room temperature. In the morning, the reaction was concentrated under reduced pressure and directly purified over silica with 0-40% EtOAc in hexanes to give the corresponding tosyl-protected amine (1.17 g, 90%, 2 steps).

This methyl ester (1168 mg, 1 equiv, 3.928 mmol) was reduced according to the same procedure used for the racemic material to afford the alcohol as a sticky residue (987 mg, 93%). NMR values matched the racemic material. $[a]_D^{22}$ –14.4° (c 0.8, $CHCl_3$).

(-)-Ethyl (*E*)-3-((1*R*,2*S*)-2-((4-methylphenyl)sulfonamido)cyclopentyl)acrylate (S40):

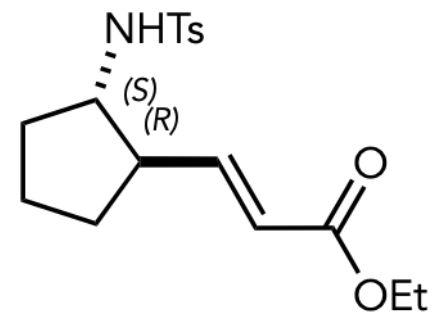

Prepared identically as the racemic material on 3.83 mmol scale to provide the enantiopure material as a white solid (1.05 g, 81%). NMR values matched racemic material. >99% ee [Daicel CHIRALPAK OD-H, 5% to 40% *i*PrOH, 30 min, 1 mL/min, t_1 = 11.55 min, t_2 = 12.97 min] $[a]_D^{22}$ –11.6° (c 1.0, $CHCl_3$).

Synthesis of (±)-N-((trans, trans)-3-((3,5-bis(trifluoromethyl)benzyl)oxy)-2-phenylcyclopentyl)-4-methylbenzenesulfonamide (**S43**)*:*

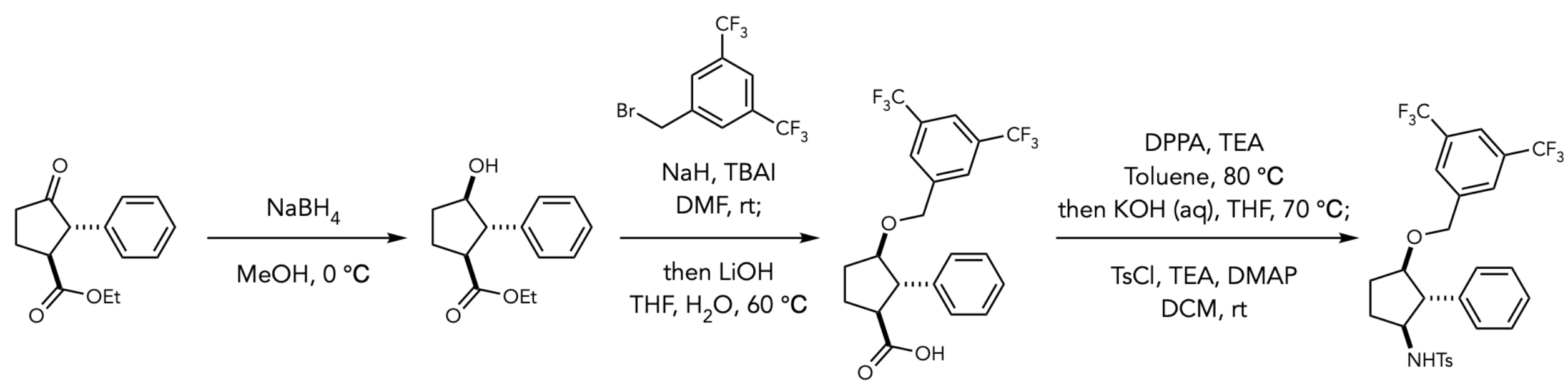

S21 **S41** **S42** **23**

(±)-Ethyl (trans, trans)-3-hydroxy-2-phenylcyclopentane-1-carboxylate (S41):

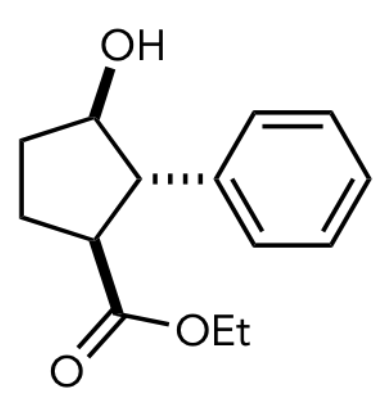

To a solution of ketone **S21** (1.0 g, 1 equiv, 4.31 mmol) in MeOH (18 mL) was added $NaBH_4$ (195 mg, 1.2 equiv, 5.17 mmol) at 0 °C. The reaction was stirred for one hour before being quenched with 1 mL saturated ammonium chloride and concentrated under vacuum. The reside was partitioned between water and EtOAc and the organic layer was separated. The aqueous layer was extracted twice more with EtOAc, and the combined organic layers were dried, concentrated and purified over silica with 0-40% EtOAc in hexanes to provide the product as a sticky residue (743 mg, 73%). **^{1}H NMR** (500 MHz, $CDCl_3$) δ 7.37 – 7.29 (m, 2H), 7.28 – 7.21 (m, 3H), 4.23 (m, 1H), 4.07 (q, *J* = 7.1 Hz, 2H), 3.24 (dd, *J* = 9.5, 7.4 Hz, 1H), 3.00 – 2.89 (m, 1H), 2.21 – 2.08 (m, 3H), 1.97 (d, *J* = 4.6 Hz, 1H), 1.90 – 1.80 (m, 1H), 1.16 (t, *J* = 7.1 Hz, 3H). **^{13}C NMR** (126 MHz, $CDCl_3$) δ 175.4, 141.1, 128.8, 127.7, 127.0, 79.9, 60.7, 57.7, 49.4, 33.2, 26.5, 14.2. **HRMS** (ESI) calculated for $[C_{14}H_{19}O_3]^+$ $(M+H)^+$ requires *m/z* 235.1329, found 235.1325.

(±)- (trans, trans)-3-((3,5-bis(trifluoromethyl)benzyl)oxy)-2-phenylcyclopentane-1-carboxylic acid (S42): To a vial of NaH (190 mg of 60% by weight, 1.5 equiv, 4.75 mmol) on ice was added a solution of alcohol **S41** (743 mg, 1 equiv, 3.17 mmol) and tetrabutylammonium iodide (586 mg, 0.5 equiv, 1.59 mmol) in DMF/THF (2:1, 15 mL). After stirring for 20 minutes on ice, 3,5-bistrifluoromethyl-benzylbromide (1.95 g, 2 equiv, 6.34 mmol) was added and the reaction was stirred for four hours at room temperature. The reaction was poured into saturated ammonium chloride and extracted three times with EtOAc. Combined organic layers were dried, concentrated and purified over silica with 0-15% EtOAc in hexanes to give the product as a yellow oil (714 mg, 49%).

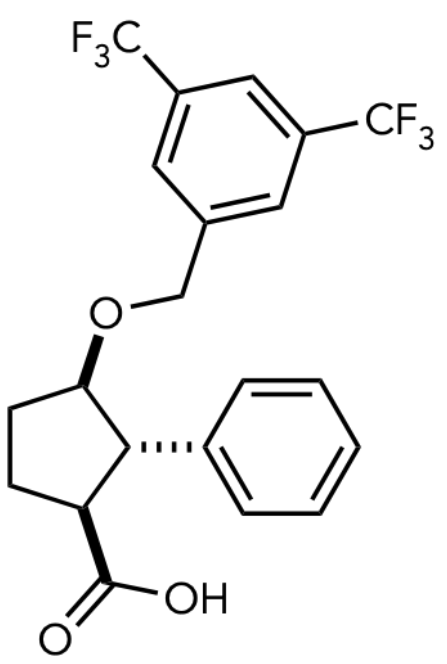

This oil (714 mg, 1 equiv, 1.55 mol) was dissolved in THF (6 mL) followed by addition of LiOH (7.75 mL of 1M aqueous solution). The reaction was heated to 60 °C and stirred overnight. In the morning the reaction was acidified to pH 2 by addition of concentrated HCl and extracted 3 times with EtOAc. The combined organic layers were dried and concentrated to provide the acid as a sticky residue (650 mg, 97%). **^{1}H NMR** (500 MHz, $CDCl_3$) δ 7.66 (s, 1H), 7.53 (s, 2H), 7.27 – 7.21 (m, 2H), 7.20 – 7.14 (m, 4H), 4.46 – 4.36 (m, 2H), 3.99 – 3.91 (m, 1H), 3.41 (dd, J = 9.7, 6.6 Hz, 1H), 2.93 – 2.84 (m, 1H), 2.17 – 1.99 (m, 3H), 1.96 – 1.86 (m, 1H). **^{13}C NMR** (126 MHz, $CDCl_3$) δ 179.7, 141.6, 141.2, 131.6 (q, J = 33.2 Hz), 128.9, 127.5, 127.2, 126.6, 123.4 (q, J = 273.8 Hz), 121.4 (m), 87.4, 70.0, 55.3, 50.2, 31.6, 27.6. **^{19}F NMR** (377 MHz, $CDCl_3$) δ -62.89. **HRMS** (ESI) calculated for $[C_{21}H_{17}F_6O_3]^-$ $(M-H)^-$ requires m/z 431.1087, found 431.1090.

(±)-*N*-((trans, trans)-3-((3,5-bis(trifluoromethyl)benzyl)oxy)-2-phenylcyclopentyl)-4-methylbenzenesulfonamide (23): To a solution of carboxylic acid **S42** (400 mg, 1 equiv, 0.925 mmol) in anhydrous toluene (6 mL) was added diphenylphosphoryl azide (0.24 mL, 1.2 equiv, 1.11 mmol) and TEA (0.193 mL, 1.5 equiv, 1.39 mmol) and the vial was sealed and heated to 80 °C for 16 hours. After cooling to room temperature, the reaction was concentrated and resuspended in THF (7 mL). KOH (3 mL of 5M aqueous solution) was added, and the reaction was heated to 70 °C for 16 hours. After cooling to room temperature, the mixture was extracted three times with EtOAc, and the combined organic layers were dried and concentrated to provide the crude amine product. This was immediately dissolved in DCM (7 mL) followed by DMAP (22 mg, 0.2 equiv, 0.185 mmol), TEA (0.387 mL, 3 equiv, 2.78 mmol) and TsCl (353 mg, 2 equiv, 1.85 mmol) and stirred at room temperature overnight. In the morning the reaction was poured into 1M HCl and extracted three times with EtOAc. The combined organic layers were washed with saturated sodium bicarbonate, dried and concentrated. The residue was purified over silica with 0-20% EtOAc in hexanes to provide the desired product as a sticky foam (170 mg, 33%). **^{1}H NMR** (500 MHz, $CDCl_3$) δ 7.73 (s, 1H), 7.56 (s, 2H), 7.50 – 7.44 (m, 2H), 7.25 – 7.15 (m, 3H), 7.11 (d, J = 8.0 Hz, 2H), 6.99 – 6.93 (m, 2H), 4.58 (d, J = 7.0 Hz, 1H), 4.46 (d, J = 12.9 Hz, 1H), 4.39 (d, J = 12.9 Hz, 1H), 3.89 (m, 1H), 3.60 (m, 1H), 2.88 (dd, J = 9.2, 6.2 Hz, 1H), 2.39 (s, 3H), 2.26 – 2.16 (m, 1H), 2.11 – 1.98 (m, 1H), 1.96 – 1.75 (m, 2H). **^{13}C NMR** (126 MHz, $CDCl_3$) δ 143.2, 140.9, 139.4, 137.2, 131.8 (q, J = 33.3 Hz), 129.6, 128.9, 127.4, 127.3, 127.2, 127.1, 123.3 (q, J = 272.8 Hz), 121.5 (m), 85.2, 69.9, 59.6, 59.0, 31.6, 29.6, 21.6. **^{19}F NMR** (377 MHz, $CDCl_3$) δ -62.91. **HRMS** (ESI) calculated for $[C_{27}H_{24}F_6NO_3S]^-$ $(M-H)^-$ requires m/z 556.1387, found 556.1387.

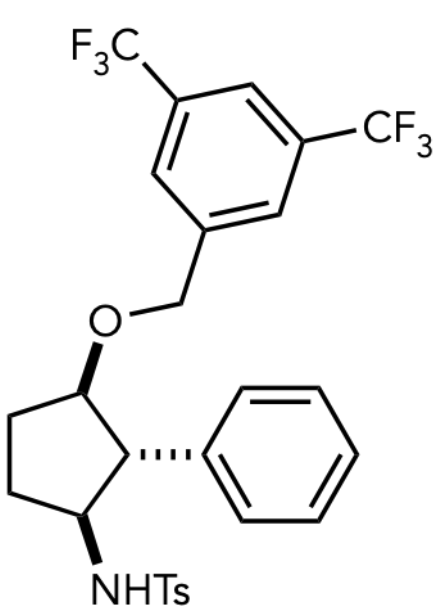

***N*-cyclopentyl-4-methylbenzenesulfonamide (19):** Prepared according to a modified **General Procedure E** using cyclopentanamine (1.0 g, 1 equiv, 10.0 mmol) and triethylamine (3.0 mL, 2 equiv, 20.0 mmol) and was purified over silica with 0-20% ethyl acetate in hexanes to afford product **19** as a white solid (2.08 g, 87%). **^{1}H NMR** (500 MHz, $CDCl_3$) δ 7.77 (d, *J* = 8.0 Hz, 2H), 7.30 (d, *J* = 7.9 Hz, 2H), 4.61 (d, *J* = 7.2 Hz, 1H), 3.58 (h, *J* = 6.8 Hz, 1H), 2.43 (s, 3H), 1.82 – 1.71 (m, 2H), 1.63 – 1.59 (m, 2H), 1.54 – 1.42 (m, 2H), 1.42 – 1.30 (m, 2H). NMR values matched previously reported spectra.[14]

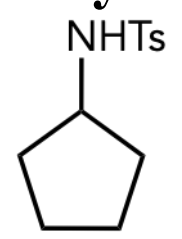

7-tosyl-7-azabicyclo[4.1.0]heptane (S43): Prepared according to **General Procedure A** on 2.43 mmol scale to give product as white solid (599 mg, 97%). **^{1}H NMR** (400 MHz, $CDCl_3$): δ 7.81 (d, *J* = 8.0Hz, 2H), 7.32(d, *J* = 8.0Hz, 2H), 2.96(s, 2H), 2.43(s, 3H), 1.77 – 1.80 (m, 4H), 1.39 – 1.44 (m, 2H), 1.22 – 1.25(m, 2H). NMR values matched previously reported spectra.[3]

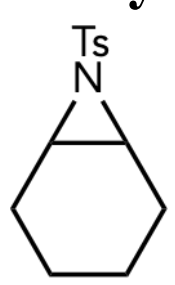

(±)-4-methyl-*N*-((trans)-2-phenylcyclohexyl)benzenesulfonamide (18): Prepared according to **General Procedure B** on 2.38 mmol scale to give product as white solid (476 mg, 61%). **^{1}H NMR** (500 MHz, $CDCl_3$) δ 7.35 – 7.29 (m, 2H), 7.18 – 6.99 (m, 5H), 6.88 – 6.82 (m, 2H), 4.25 (d, *J* = 4.9 Hz, 1H), 3.08 (tt, *J* = 10.7, 4.7 Hz, 1H), 2.42 (m, 4H), 2.33 (m), 1.81 (m, 2H), 1.75 – 1.68 (m, 1H), 1.48 – 1.24 (m, 4H). **^{13}C NMR** (126 MHz, $CDCl_3$) δ 142.7, 142.3, 137.0, 129.5, 128.8, 127.4, 127.0, 126.8, 57.5, 50.5, 35.1, 34.7, 25.9, 25.1, 21.5. **HRMS** (ESI) calculated for $[C_{19}H_{22}NO_2S]^-$ $(M\text{-}H)^-$ requires *m/z* 328.1377, found 328.1379. **Melting Point**: 165-167 °C.

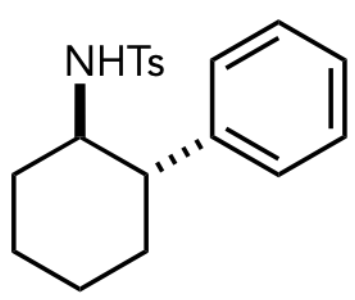

N-chloro-4-methyl-N-((trans)-2-phenylcyclopentyl)benzenesulfonamide (26): To a suspension of NaH (60% by weight, 95 mg, 1.5 equiv, 2.38 mmol) in THF (2.5 mL) was added a solution of sulfonamide **1** (500 mg, 1 equiv, 1.59 mmol) in THF (2.5 mL) at 0 °C. After 20 min, NCS (233 mg, 1.1 equiv, 1.74 mmol) in THF (5 mL) was added and the reaction was maintained at 0 °C and stirred for one hour. Following this, the reaction was poured into water and extracted three times with ether. The organic layer was dried, concentrated and purified over silica with 0-15% EtOAc in hexane to provide **26** as a white solid (464 mg, 83%). **^{1}H NMR** (500 MHz, $CDCl_3$) δ 7.72 – 7.66 (m, 2H), 7.37 – 7.30 (m, 2H), 7.30 – 7.23 (m, 5H), 4.65 (m, 1H), 3.22 (dt, *J* = 10.2, 8.1 Hz, 1H), 2.45 (s, 3H), 2.11 (m, 1H), 1.85 – 1.64 (m, 5H). **^{13}C NMR** (126 MHz, $CDCl_3$) δ 145.0, 141.8, 133.0, 129.6, 129.2, 128.6, 127.5, 126.7, 69.4, 48.5, 32.2, 26.9, 23.5, 21.7. **HRMS** (ESI) calculated for $(C_{18}H_{21}ClNO_2S)^+$ $(M+H)^+$ requires *m/z* 350.0976, found 350.0972. **Melting Point**: 103 – 104 °C.

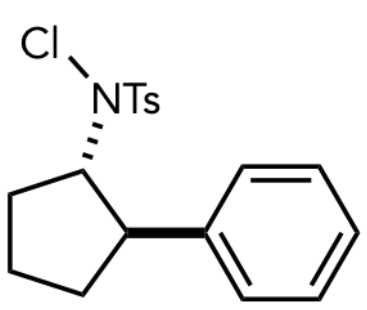

1-methyl-6-tosyl-6-azabicyclo[3.1.0]hexane (S44) Prepared according to modified **General Procedure A** on 24.3 mmol scale to provide product as an off-white solid (2.9 g, 47%). Following column purification, a small impurity remained that could not be separate by chromatography. Instead, the material was dissolved in a minimal amount of MTBE at room temperature then placed in an ice bath and stirred slowly for 10 min. Next, heptane was added (approximately 5x the volume of MTBE) and the reaction was stirred in an ice bath for 20 min. Next the reaction was filtered. The solid was collected and dried to give pure product. **^{1}H NMR** (400 MHz, DMSO-d_6) δ 1.15-1.25 (m, 1H), 1.46-1.70 (m, 4H), 1.75 (s, 3H), 1.88-1.93 (m, 1H), 2.50 (s, 3H), 3.34-3.35 (m, 1H), 7.40-7.42 (m, 2H), 7.74-7.76 (m, 2H). NMR values matched previously reported spectra.[3]

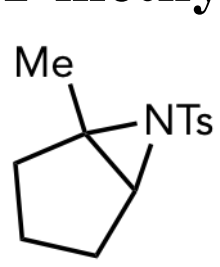

(±)-4-methyl-*N*-((1*S*,2*R*)-1-methyl-2-phenylcyclopentyl)benzenesulfonamide (S45): Prepared according to **General Procedure B** on 2.0 mmol scale to provide product as a white solid (520 mg, 79%). **^{1}H NMR** (500 MHz, $CDCl_3$) δ 7.78 – 7.71 (m, 2H), 7.35 – 7.28 (m, 2H), 7.28 – 7.21 (m, 5H), 4.57 (s, 1H), 3.20 (dd, *J* = 10.5, 8.3 Hz, 1H), 2.41 (s, 3H), 2.09 – 1.88 (m, 2H), 1.83 – 1.69 (m, 3H), 0.90 (s, 3H). **^{13}C NMR** (126 MHz, $CDCl_3$) δ 143.0, 140.4, 139.3, 129.6, 128.9, 128.4, 127.1, 127.0, 65.8, 55.8, 37.4, 27.5, 22.4, 21.6, 20.7. **HRMS** (ESI) calculated for $[C_{19}H_{24}NO_2S]^+$ $(M+H)^+$ requires *m/z* 330.1522, found 330.1516. **Melting Point**: 145-150 °C.

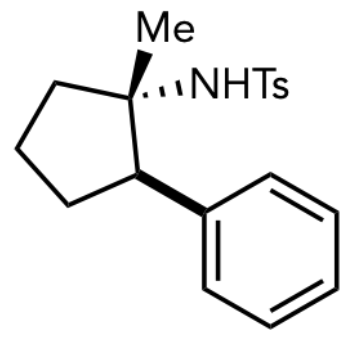

***N*,4-dimethyl-*N*-((1S,2R)-2-phenylcyclopentyl)benzenesulfonamide (S46):** To a flame- dried 40 mL vial under N_2 atmosphere was added sulfonamide **1** and K_2CO_3 (415 mg, 2 equiv, 3.00 mmol). The contents were diluted in DMF (18.8 mL) and methyl iodide (282 µL, 3.0 equiv, 4.50 mmol) was subsequently added. The reaction was allowed to stir at room temperature overnight. The reaction was quenched with NH_4Cl and extracted three times with ethyl acetate. The combined organic layers were washed with brine and 5% (w/v) Na_2SO_3, successively. The organic layer was dried with $MgSO_4$, filtered, concentrated, and purified over silica with 0-30% EtOAc in hexane to provide a clear and colorless oil (363 mg, 74%). **^{1}H NMR** (500 MHz, $CDCl_3$) δ 7.35 (d, *J* = 8.5 Hz, 2H), 7.25 – 7.13 (m, 5H), 7.07 (d, *J* = 7.9 Hz, 2H), 4.51 – 4.42 (m, 1H), 2.94 (td, *J* = 10.2, 7.8 Hz, 1H), 2.76 (s, 3H), 2.36 (s, 3H), 2.10 – 2.01 (m, 1H), 1.86 – 1.55 (m, 5H). **^{13}C NMR** (126 MHz, $CDCl_3$) δ 142.57, 142.16, 136.73, 129.32, 128.52, 127.44, 127.14, 126.41, 64.34, 47.72, 32.69, 28.20, 26.75, 22.20, 21.43. **HRMS** (ESI) calculated for $[C_{19}H_{24}NO_2S]^+$ $(M+H)^+$ requires *m/z* 330.1522, found 330. 1519. **Melting Point**: 75 – 79 °C.

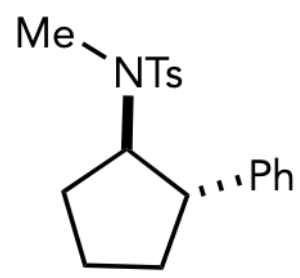

E. Preparation of cyclobutyl sulfonamides

Synthesis of 4-methyl-N-(2-phenylcyclobutyl)benzenesulfonamide **(S49):**

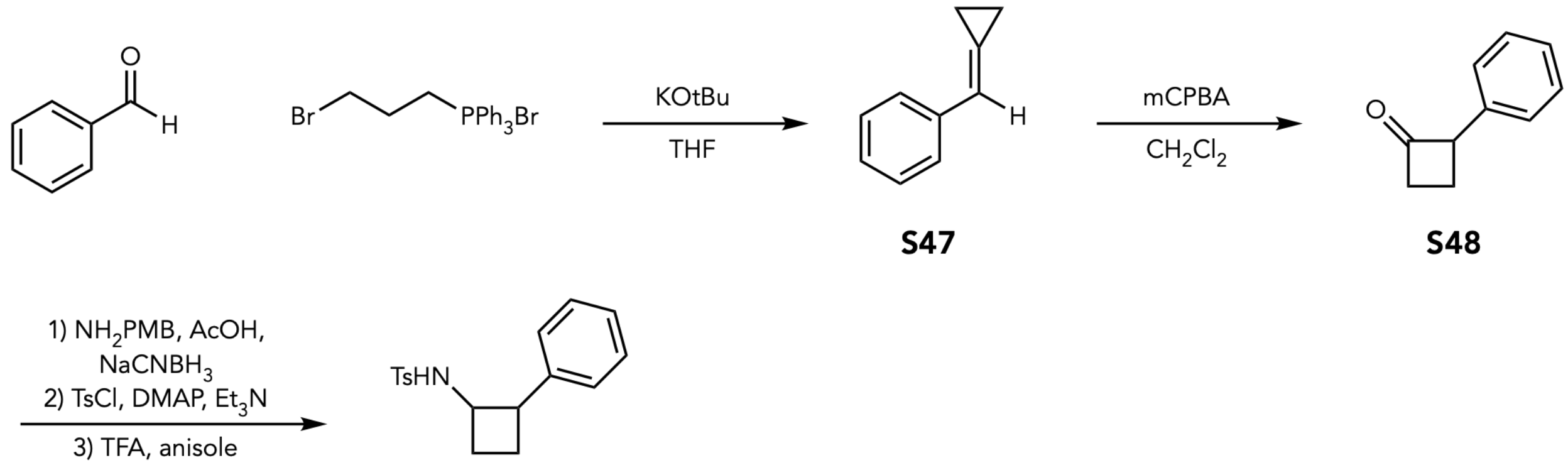

(cyclopropylidenemethyl)benzene (S47): To a dried three-necked flask under N_2 atmosphere was added 1-bromopropyl-3-triphenylphosphonium bromide (6.96 g, 15.0 mmol) and tetrahydrofuran (30 mL, 0.33 M). A solution of KOtBu (3.37 g, 30.0 mmol) in tetrahydrofuran (30 mL, 0.33 M) was subsequently added. The reaction was refluxed for 90 minutes. Benzaldehyde (1.02 mL, 10.0 mmol) was then added in one portion, and the reaction was allowed to reflux for another 2 hours. The flask was cooled to room temperature, and then pentanes was added to the solution. The precipitate was filtered, and the filtrate was concentrated under reduced pressure. The residue was purified by flash column chromatography on regular silica with 100% pentanes to furnish the product as a colorless oil (1.23 g, 95% yield). **^{1}H NMR** (500 MHz, $CDCl_3$) δ (ppm) = 7.57 – 7.50 (m, 2H), 7.34 (dd, J = 8.5, 7.0 Hz, 2H), 7.24 – 7.19 (m, 1H), 6.76 (p, J = 2.1 Hz, 1H), 1.44 (ddd, J = 9.8, 5.7, 2.3 Hz, 2H), 1.22 – 1.15 (m, 2H). Characterization data were consistent with reported literature values.[15]

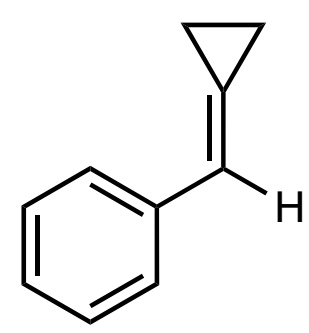

2-phenylcyclobutan-1-one (S48): To a flask was added (cyclopropylidenemethyl)benzene **S47** (1.23 g, 9.45 mmol) and CH_2Cl_2 (63 mL, 0.15 M). The reaction mixture was stirred until (cyclopropylidenemethyl)benzene dissolved and then cooled to 0 °C. A separate solution of mCPBA (2.56 g, 10.4 mmol) in CH_2Cl_2 (23.6 mL, 0.4 M) was prepared and added dropwise over 10 minutes. The reaction was stirred at 0 °C and monitored by TLC. Upon completion, the reaction was quenched with an aqueous Na_2SO_3 solution and extracted with CH_2Cl_2. The organic layer was washed with $NaHCO_3$ 3x and then dried over Na_2SO_4 and concentrated under reduced pressure. The crude material was then purified by flash column chromatography on regular silica with 10:90 Hexanes:Et_2O to furnish the product as a colorless oil (810 mg, 59% yield). **^{1}H NMR** (500 MHz, $CDCl_3$) δ (ppm) = 7.38 – 7.30 (m, 2H), 7.27 – 7.21 (m, 3H), 4.55 (ddt, J = 10.8, 8.2, 2.7 Hz, 1H), 3.23 (dddd, J = 17.6, 10.5, 8.2, 2.5 Hz, 1H), 3.04 (dddd, J = 17.6, 9.8, 5.0, 2.6 Hz, 1H), 2.55 (dtd, J = 11.2, 10.5, 5.0 Hz, 1H), 2.25 (ddt, J = 11.2, 9.8, 8.2 Hz, 1H). Characterization data were consistent with reported literature values.[15]

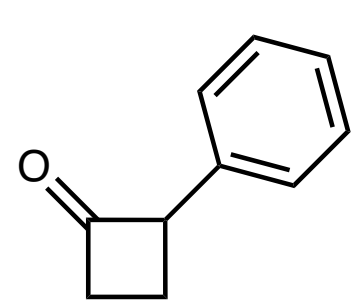

4-methyl-*N*-(2-phenylcyclobutyl)benzenesulfonamide (35): 1) To a dry flask under a N_2 atmosphere was added a magnetic stir bar, 2-phenylcyclobutan-1-one (810 mg, 5.54 mmol), dichloroethane (16.8 mL, 0.33 M), and (4-methoxyphenyl)methanamine (1.45 mL, 11.1 mmol). The reaction was allowed to stir for 30 minutes, and then sodium cyanoborohydride (696 mg, 11.1 mmol) was added portion wise. The reaction was allowed to stir overnight at room temperature. Upon completion, the reaction was quenched with an aqueous solution Na_2CO_3 until bubbling stops, then transferred to a separatory funnel and extracted with EtOAc. The organic layer was dried over Na_2SO_4 and then concentrated under reduced pressure. The crude material was passed through a silica plug with 98:2 EA:Et_3N and carried forward without further purification. 2) The partially purified material (1.39 g, 5.2 mmol) was added to a flask and diluted with CH_2Cl_2 (20.8 mL, 0.25 M), followed by the addition of DMAP (159 mg, 25 mol%) and Et_3N (7.25 mL, 52.0 mmol). Tosyl chloride was then added portion wise (4.96 g, 26.0 mmol) over 10 minutes. The reaction was stirred overnight at room temperature. Upon completion, the reaction was diluted with EtOAc and washed with aqueous $NaHCO_3$ 5x. The organic layer was collected, dried over Na_2SO4, and concentrated under reduced pressure. The crude material was partially purified by flash column chromatography on regular silica with an eluent of 2:98 Toluene:EA and then carried forward without further purification. 3) Trifluoroacetic acid (6.95 mL, 90.3 mmol) was added in a single portion to a solution of *N*-(4-methoxybenzyl)-4-methyl-*N*-(2-phenylcyclobutyl)benzenesulfonamide (761 mg, 1.81 mmol) in CH_2Cl_2 (18.1 mL, 0.10 M). Anisole (0.589 mL, 5.42 mmol) was then added to the solution. The reaction was monitored by TLC and, upon completion, diluted with EtOAc and quenched slowly with $NaHCO_3$. The organic phase was washed once more with $NaHCO_3$ and then with brine. The organic phase was collected, dried over Na_2SO_4, and concentrated under reduced pressure. The crude mixture was purified by flash column chromatography on regular silica with a gradient of 0–10% EtOAc:Hexanes to furnish separable diastereomers (265 mg, 49% yield, 1.6 d.r.) as tan solids. Major-isomer (*trans*): **^{1}H NMR** (500 MHz, $CDCl_3$) δ 7.64 – 7.58 (m, 2H), 7.35 – 7.21 (m, 5H), 6.99 – 6.92 (m, 2H), 4.27 – 4.19 (m, 1H), 4.19 – 4.12 (m, 1H), 3.69 (dq, *J* = 9.4, 4.5 Hz, 1H), 2.44 (s, 2H), 2.18 – 2.03 (m, 3H). **^{13}C NMR** (126 MHz, $CDCl_3$) δ 143.3, 138.1, 137.8, 129.6, 128.9, 128.1, 127.1, 127.0, 50.3, 46.0, 30.4, 21.6, 20.8. Minor-isomer (*cis*): **^{1}H NMR** (500 MHz, $CDCl_3$) δ 4.97 (d, *J* = 9.1 Hz, 1H), 3.88 (p, *J* = 8.4 Hz, 1H), 3.21 (q, *J* = 9.2 Hz, 1H), 2.37 (s, 3H), 2.16 – 2.05 (m, 2H), 1.76 – 1.60 (m, 2H). **^{13}C NMR** (126 MHz, $CDCl_3$) δ 143.3, 141.8, 138.0, 129.7, 128.4, 127.1, 126.6 (2C), 54.5, 49.9, 28.6, 22.7, 21.6. **HRMS** (ESI) calculated for $[C_{17}H_{23}N_2O_2S]^+$ $(M+H)^+$ requires *m/z* 319.1475, found 319.1472. **Melting Point (*cis*)**: 141 – 143 °C.

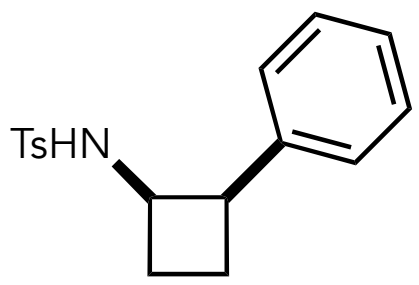

N-cyclobutyl-4-methylbenzenesulfonamide (S49): To an oven-dried flask equipped with a magnetic stir bar was added cyclobutanamine (745 mL, 10.00 mmol, 1.0 equiv) and K_2CO_3 (3.46 g, 25.0 mmol, 2.5 equiv). The reaction components were diluted in CH_3Cl (12.05 mL, 0.83 M) and H_2O (12.05 mL, 0.83 M). TsCl (1.91 g, 10.0 mmol, 1.0 equiv) was added portion wise, and the reaction was allowed to stir overnight at room temperature. Upon completion, the reaction was diluted in EtOAc, transferred to a separatory funnel, and then washed with $NaHCO_3$ (2x) and brine. The organic layer was collected, dried over Na_2SO_4, and concentrated under reduced pressure to afford the product as a white solid without further purification (2.01 g, 89% yield). **^{1}H NMR** (500 MHz, $CDCl_3$) δ 7.74 (d, *J* = 8.4 Hz, 1H), 7.29 (d, *J* = 8.0 Hz, 1H), 4.62 (d, *J* = 9.0 Hz, 1H), 3.84 – 3.72 (m, 1H), 2.42 (s, 2H), 2.17 – 2.07 (m, 1H), 1.81 – 1.69 (m, 1H), 1.67 – 1.56 (m, 1H). Characterization data were consistent with reported literature values.[16]

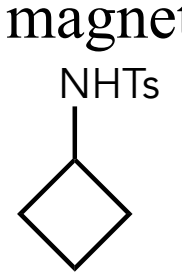

3-((4-methylphenyl)sulfonamido)cyclobutyl benzoate (S50): To an oven-dried flask equipped with a magnetic stir bar was added N-(3-hydroxycyclobutyl)-4-methylbenzenesulfonamide (241 mg, 1.00 mmol, 1.0 equiv), and the flask was cooled to 0 °C. The material was diluted in CH_2Cl_2 (10 mL, 0.1 M), and then Et_3N (0.279 mL, 2.00 mmol, 2.0 equiv) was added, followed by dropwise addition of benzoyl chloride (0.138 mL, 1.20 mmol, 1.2 equiv). The solution was stirred overnight and then diluted in EtOAc. The solution was transferred to a separatory funnel and then washed with $NaHCO_3$ and brine. The organic layer was collected, dried over Na_2SO_4, and concentrated under reduced pressure. The crude material was purified via flash column chromatography (15–60% EtOAc:Hexanes) to afford the product as a white solid (315 mg, 1.4:1 d.r., 91% yield). *Cis-isomer (major)* **^{1}H NMR** (500 MHz, $CDCl_3$) δ 7.97 (dd, *J* = 8.3, 1.4 Hz, 2H), 7.75 (d, *J* = 8.3 Hz, 2H), 7.55 (ddt, *J* = 8.7, 7.1, 1.3 Hz, 1H), 7.45 – 7.39 (m, 2H), 7.31 (d, *J* = 7.8 Hz, 2H), 4.84 (tt, *J* = 7.7, 6.8 Hz, 1H), 4.76 (d, *J* = 8.8 Hz, 1H), 3.58 (qt, *J* = 8.6, 7.2 Hz, 1H), 2.83 – 2.73 (m, 2H), 2.43 (s, 3H), 2.04 – 1.94 (m, 2H). **^{13}C NMR** (126 MHz, $CDCl_3$) δ 165.8, 143.8, 137.4, 133.2, 129.9, 129.7, 129.6, 128.4, 127.1, 63.0, 41.0, 39.3, 21.6. *Trans-isomer (minor)* **^{1}H NMR** (500 MHz, $CDCl_3$) δ 8.00 (dd, *J* = 8.3, 1.4 Hz, 2H), 7.75 (d, *J* = 8.3 Hz, 2H), 7.60 – 7.53 (m, 1H), 7.44 (t, *J* = 7.8 Hz, 2H), 7.31 (d, *J* = 7.9 Hz, 2H), 5.23 (ttd, *J* = 7.1, 3.6, 0.8 Hz, 1H), 4.97 (d, *J* = 7.8 Hz, 1H), 4.18 – 4.05 (m, 1H), 2.51 – 2.44 (m, 2H), 2.43 (s, 3H), 2.40 – 2.29 (m, 2H). **^{13}C NMR** (126 MHz, $CDCl_3$) δ 166.2, 143.7, 137.4, 133.2, 129.9, 129.9, 129.6, 128.4, 127.1, 67.7, 45.4, 37.8, 21.6. **HRMS** (ESI) calculated for $[C_{18}H_{18}NO_4S]^-$ $[M–H]^-$ requires m/z 344.0962, found 344.0967. **Melting Point**: 110–114 °C.

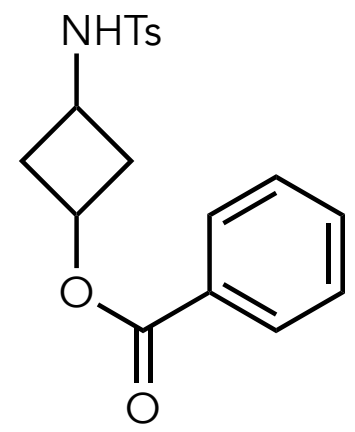

***cis*-methyl 3-((4-methylphenyl)sulfonamido)cyclobutane-1-carboxylate (S51)**: To an oven-dried flask charged with a magnetic stir bar was added *cis*-methyl-3-aminocyclobutane-1-carboxylate hydrochloride (432 mg, 2.61 mmol, 1.00 equiv) and DMAP (79.7 mg, 0.653 mmol, 25 mol%). The reaction components were diluted in CH_2Cl_2 (5.22 mL, 0.5 M), and then Et_3N (0.909 mL, 6.53 mmol, 2.5 equiv) was added. TsCl (746 mg, 3.92 mmol, 1.5 equiv) was then added portionwise, and the reaction was allowed to stir at room temperature overnight. Upon completion, the reaction was diluted in EtOAc, transferred to a separatory funnel, washed with $NaHCO_3$ (2x), washed with brine, and then the organic layer was collected, dried with Na_2SO_4, and concentrated under reduced pressure. The crude mixture was purified via flash column chromatography (25–100% EtOAc:Hexanes) to afford the product as a white solid (169.7 mg, 23% yield). **^{1}H NMR** (500 MHz, $CDCl_3$) δ 7.73 (d, *J* = 8.4 Hz, 2H), 7.29 (d, *J* = 7.8 Hz, 2H), 4.81 (d, *J* = 9.7 Hz, 1H), 3.80 (dtt, *J* = 9.7, 8.8, 7.6 Hz, 1H), 3.64 (s, 3H), 2.69 (tt, *J* = 9.4, 7.9 Hz, 1H), 2.47 – 2.35 (m, 5H), 2.00 (qd, *J* = 9.4, 2.8 Hz, 2H). **^{13}C NMR** (126 MHz, $CDCl_3$) δ 174.7, 143.6, 137.9, 129.8, 127.0, 52.0, 44.5, 34.8, 31.4, 21.6. **HRMS** (ESI) calculated for $[C_{13}H_{16}NO_4S]^-$ $[M–H]^-$ requires m/z 282.0806, found 282.0810. **Melting Point**: 123–126 °C.

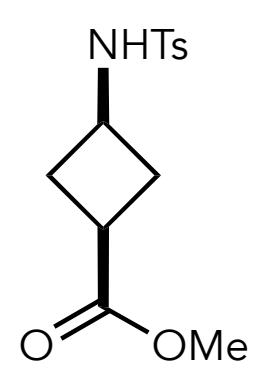

***tert*-butyl 2-((4-methylphenyl)sulfonamido)-7-azaspiro[3.5]nonane-7-carboxylate (S52):** To an oven-dried flask equipped with a magnetic stir bar was added tert-butyl 2-amino-7-azaspiro[3.5]nonane-7-carboxylate (500 mg, 2.08 mmol, 1.0 equiv) and DMAP (63.5 mg, 0.520 mmol, 25 mol%). The reaction components were diluted in CH_2Cl_2 (4.16 mL, 0.5 M), and then Et_3N was added (0.580 mL, 4.16 mmol, 2.0 equiv). TsCl (436 mg, 2.29 mmol, 1.1 equiv) was added portion wise, and the reaction was allowed to stir overnight at room temperature. Upon completion, the reaction was diluted in EtOAc, transferred to a separatory funnel, washed with $NaHCO_3$ (2x), washed with NH_4Cl (2x), and brine.

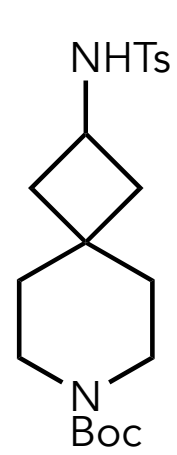

The organic layer was collected, dried over Na_2SO_4, and concentrated under reduced pressure to afford the product as a white solid without further purification (706 mg, 86% yield). **^{1}H NMR** (500 MHz, $CDCl_3$) δ 7.73 (d, *J* = 8.3 Hz, 2H), 7.30 (d, *J* = 8.1 Hz, 2H), 4.68 (d, *J* = 8.4 Hz, 1H), 3.79 (q, *J* = 8.1 Hz, 1H), 3.31 – 3.24 (m, 2H), 3.24 – 3.17 (m, 2H), 2.43 (s, 3H), 2.12 (ddd, *J* = 12.2, 6.3, 2.3 Hz, 2H), 1.57 – 1.49 (m, 2H), 1.48 – 1.37 (m, 13H). **^{13}C NMR** (126 MHz, $CDCl_3$) δ 155.0, 143.7, 137.8, 129.9, 127.2, 79.6, 43.7, 40.9, 39.4, 35.6, 32.7, 28.6, 21.7. **HRMS** (ESI) calculated for $[C_{20}H_{29}N_2O_4S]^-$ $[M–H]^-$ requires m/z 393.1854, found 393.1863. **Melting Point:** 195–197 °C.

Synthesis Procedure for N-((1S,5R)-bicyclo[3.2.0]hept-2-en-6-yl)-4-methylbenzenesulfonamide (**S53**):

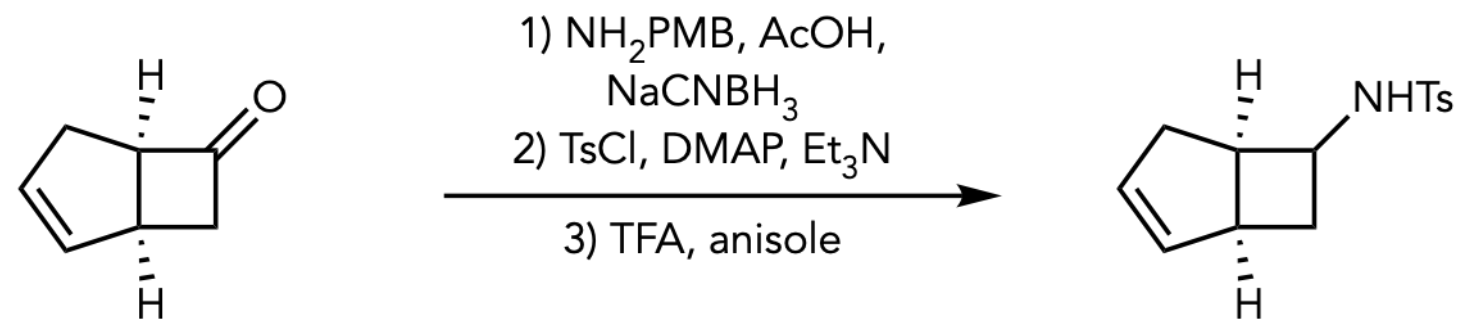

S53

***N*-((1*S*,5*R*)-bicyclo[3.2.0]hept-2-en-6-yl)-4-methylbenzenesulfonamide (S53)**: To a dry flask under a N_2 atmosphere was added a magnetic stir bar, (1S,5R)-bicyclo[3.2.0]hept-2-en-6-one (1.081 g, 10.0 mmol), dichloroethane (30.0 mL, 0.33 M), and (4-methoxyphenyl)methanamine (2.61 mL, 20.0 mmol). The reaction was allowed to stir for 30 minutes, and then sodium cyanoborohydride (1.26 g, 20.0 mmol) was added portion wise. The reaction was allowed to stir overnight at room temperature. Upon completion, the reaction was quenched with an aqueous solution Na_2CO_3 until bubbling stops, then transferred to a separatory funnel and extracted with EtOAc. The organic layer was dried over Na_2SO_4 and then concentrated under reduced pressure. The crude material was passed through a silica plug with 98:2 EA:Et_3N and carried forward without further purification. 2) The partially purified material (459 mg, 2.0 mmol) was added to a flask and diluted with CH_2Cl_2 (10.0 mL, 0.2 M), followed by the addition of DMAP (61.1 mg, 25 mol%) and Et_3N (1.39 mL, 10.0 mmol). Tosyl chloride was then added portion wise (1.53 g, 8.00 mmol) over 10 minutes. The reaction was stirred overnight at room temperature. Upon completion, the reaction was diluted with EtOAc and washed with aqueous $NaHCO_3$ 5x. The organic layer was collected, dried over Na_2SO4, and concentrated under reduced pressure. The crude material was carried forward without further purification. 3) Trifluoroacetic acid (4.19 mL, 54.8 mmol) was added in a single portion to a solution of N-((1S,5R,6R)-bicyclo[3.2.0]hept-2-en-6-yl)-N-(4-methoxybenzyl)-4-methylbenzenesulfonamide (420 mg, 1.10 mmol) in CH_2Cl_2 (5.48 mL, 0.20 M). Anisole (0.357 mL, 3.29 mmol) was then added to the solution. The reaction was monitored by TLC and, upon completion, diluted with EtOAc and quenched slowly with $NaHCO_3$. The organic phase was washed once more with $NaHCO_3$ and then with brine. The organic phase was collected, dried over Na_2SO_4, and concentrated under reduced pressure. The crude mixture was purified by flash column chromatography on regular silica with a gradient of 0–25% EtOAc:Hexanes to furnish a mixture of diastereomers (200 mg, 69% yield, 10:1 d.r.). **^{1}H NMR** (500 MHz, $CDCl_3$) only major diastereomer is reported δ 7.74 (d, *J* = 8.1 Hz, 2H), 7.29 (d, *J* = 7.9 Hz, 2H), 5.68 (m, 2H), 4.81 (d, *J* = 9.0 Hz, 1H), 3.49 (qd, *J* = 8.1, 5.6 Hz, 1H), 3.03 (d, *J* = 8.7 Hz, 1H), 2.68 (q, *J* = 7.0 Hz, 1H), 2.42 (s, 3H), 2.37 (ddd, *J* = 17.1, 7.6, 2.0 Hz, 1H), 2.17 – 2.11 (m, 1H), 2.06 (ddd, *J* = 11.2, 8.2, 2.5 Hz, 1H), 1.94 (dt, *J* = 12.0, 8.7 Hz, 1H). **^{13}C NMR** (126 MHz, $CDCl_3$) δ 143.5, 138.0, 133.5, 131.1, 129.8, 127.3, 54.0, 46.9, 40.3, 38.6, 35.9, 21.7. **HRMS** (ESI)

calculated for $[C_{14}H_{18}NO_2S]^+$ $[M+H]^+$ requires m/z 264.1053, found 264.1054. **Melting Point:** 92–94 °C.

F. Experimental procedure, isolation, and characterization for enamine products

General Procedure F: *Isolation Scale N-Insertion Conditions*

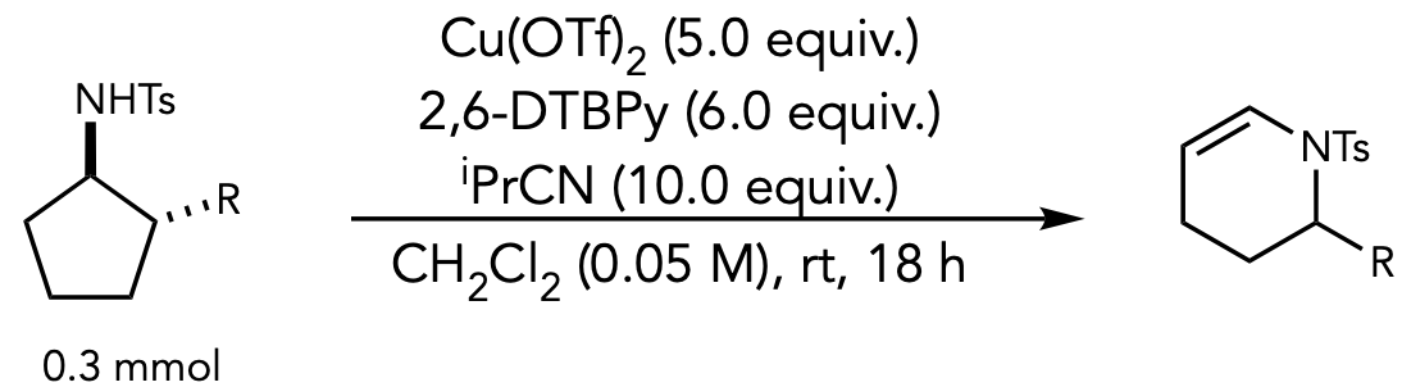

A flame dried 20-mL vial charged with sulfonamide (0.3 mmol) and a stirbar was brought into the glovebox. $Cu(OTf)_2$ (543 mg, 5 equiv, 1.5 mmol) was added to the vial followed by DCM (5.5 mL), *i*PrCN (0.269 mL, 10 equiv, 3.0 mmol) and 2,6-di-*t*Bu-pyridine (0.404 mL, 6 equiv, 1.8 mmol). The vial was then sealed with a Teflon cap, removed from the glovebox and stirred at room temperature for 18 hours. The reaction was quenched by addition of 4 mL of saturated ammonium chloride and diluted with 10 mL of EtOAc. The organic layer was separated, and the aqueous layer was extracted once more with EtOAc. The combined organic layers were washed once with saturated ammonium chloride (~2 mL) then passed through a 0.5 cm plug of silica, eluting with EtOAc, and concentrated to provide a residue. This residue was purified over silica with either 0-20% or 0-40% EtOAc in hexanes to provide the desired products.

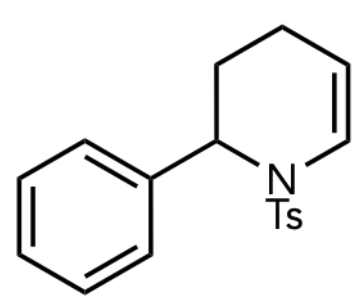

(±)-2-phenyl-1-tosyl-1,2,3,4-tetrahydropyridine (2): Prepared according to **General Procedure F** to give product as a white solid (85 mg, 91% from trans, 82 mg, 87% from cis). **^{1}H NMR** ($CDCl_3$, 500MHz) δ 7.65 (2H, d, J = 8.5 Hz), 7.27-7.21 (7H, m), 6.92 (1H, d, J = 8.0Hz), 5.19 (1H, s), 5.06 (1H, t, J = 7.0 Hz), 2.43 (3H, s), 1.93 (1H, br d, J = 13.5 Hz), 1.80 (1H, dt, J = 18.0, 5.0 Hz), 1.66-1.59 (1H, m), 1.45 (1H, tt, J = 12.5, 5.0 Hz). NMR values matched previously reported spectra.[17]

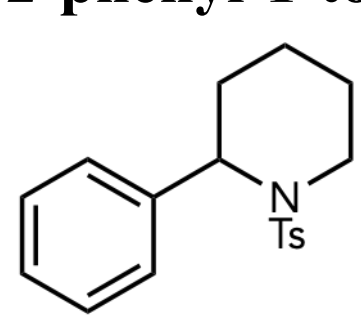

2-phenyl-1-tosylpiperidine (S54): To a vial charged with enamide **2** (94 mg, 1 equiv, 0.30 mmol) was added DCM (4.0 mL), TFA (92 µL, 4 equiv, 1.20 mmol) and Et_3SiH (192 µL, 4 equiv, 1.20 mmol) and the reaction was stirred at room temperature overnight. The reaction was concentrated under reduced pressure and purified over silica with 0-15% EtOAc in hexanes to afford product as a white solid (84 mg, 89%). **^{1}H NMR** (400 MHz, $CDCl_3$) δ 7.76 (d, *J* = 8.3 Hz, 2H), 7.37 – 7.27 (m, 6H), 7.26 – 7.20 (m, 1H), 5.27 (d, *J* = 5.2 Hz, 1H), 3.84 (d, *J* = 14.2 Hz, 1H), 3.01 (ddd, *J* = 14.3, 12.6, 3.1 Hz, 1H), 2.44 (s, 3H), 2.21 (d, *J* = 14.2 Hz, 1H), 1.72 – 1.59 (m, 1H), 1.51 – 1.22 (m, 4H). NMR values matched previously reported spectra.[18]

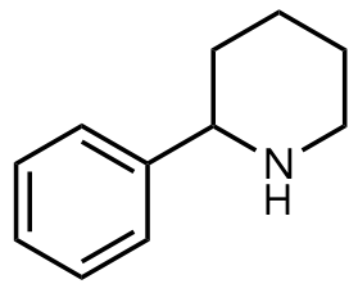

2-phenylpiperidine (S55): To a vial with tosylpiperidine **S54** (47.3 mg, 1equiv, 0.15 mmol) was added PhOH (42.3 mg, 3 equiv, 0.45 mmol) and 48% aqueous HBr (485 uL) and the reaction was stirred for 2 hours at 130 ºC. The acidic mixture was washed with diethyl ether and then made alkaline using 30% sodium hydroxide solution. The crude amine was extracted with diethyl ether and then dried over Na_2SO_4 and concentrated. The residue was purified over silica with either 0-10% MeOH in DCM to provide the amine as a clear and colorless oil (10.2 mg, 42%). **^{1}H NMR** (400 MHz, $CDCl_3$) δ 7.43 – 7.30 (m, 5H), 3.65 – 3.58 (m, 1H), 3.23 – 3.16 (m, 1H), 2.83 (td, *J* = 11.6, 3.0 Hz, 1H), 2.43 (br, 1H), 1.92 – 1.86 (m, 1H), 1.83 – 1.75 (m, 2H), 1.71 – 1.62 (m, 1H), 1.61 – 1.44 (m, 3H). NMR values matched previously reported spectra.[19]

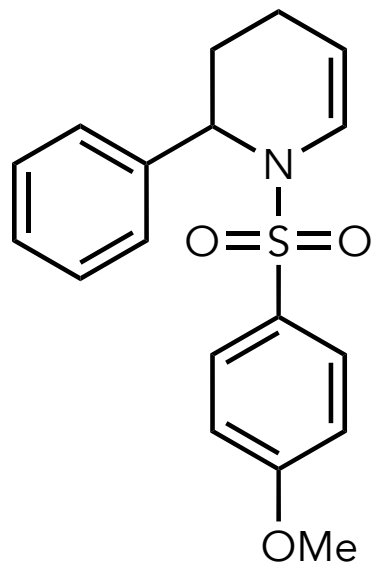

(±)-1-((4-methoxyphenyl)sulfonyl)-2-phenyl-1,2,3,4-tetrahydropyridine (3): Prepared according to **General Procedure F** to give product as a white solid (87 mg, 88%). **^{1}H NMR** (500 MHz, $CDCl_3$) δ 7.70 (d, *J* = 8.9 Hz, 2H), 7.28 – 7.25 (m, 2H), 7.22 (td, *J* = 6.6, 1.7 Hz, 3H), 6.96 – 6.89 (m, 3H), 5.19 (t, *J* = 3.7 Hz, 1H), 5.05 (ddt, *J* = 7.8, 5.8, 1.7 Hz, 1H), 3.88 (s, 3H), 1.98 – 1.90 (m, 1H), 1.86 – 1.76 (m, 1H), 1.65 (dddt, *J* = 17.6, 14.7, 5.1, 2.2 Hz, 1H), 1.49 (tt, *J* = 12.8, 4.8 Hz, 1H). **^{13}C NMR** (126 MHz, $CDCl_3$) δ 162.86, 140.27, 131.02, 129.07, 128.31, 126.97, 125.91, 124.63, 114.13, 108.34, 55.85, 55.60, 26.31, 16.90. **HRMS** (ESI) calculated for $[C_{18}H_{20}NO_3S]^+$ $(M+H)^+$ requires *m/z* 330.1158, found 330.1153. **Melting Point**: 96-98 ºC.

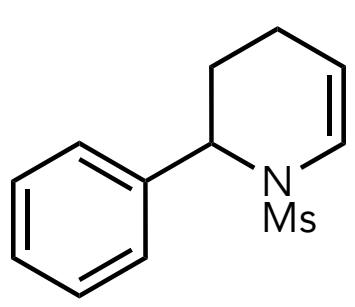

(±)-1-(methylsulfonyl)-2-phenyl-1,2,3,4-tetrahydropyridine (4): Prepared according to **General Procedure F** to give product as a clear and colorless oil (65 mg, 91%). **^{1}H NMR** (500 MHz, $CDCl_3$) δ 7.39 – 7.25 (m, 5H), 6.79 (ddt, *J* = 8.4, 2.6, 1.1 Hz, 1H), 5.31 (t, *J* = 3.9 Hz, 1H), 5.09 (tq, *J* = 5.9, 2.0 Hz, 1H), 2.80 (s, 3H), 2.17 – 2.08 (m, 1H), 2.07 – 1.94 (m, 2H), 1.86 – 1.74 (m, 1H). **^{13}C NMR** (126 MHz, $CDCl_3$) δ 140.67, 128.61, 127.41, 125.97, 124.20, 107.33, 55.98, 39.90, 27.40, 16.84. **HRMS** (ESI) calculated for $[C_{12}H_{16}NO_2S]^+$ $(M+H)^+$ requires *m/z* 238.0896, found 238.0893.

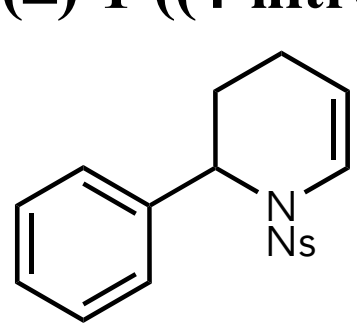

(±)-1-((4-nitrophenyl)sulfonyl)-2-phenyl-1,2,3,4-tetrahydropyridine (5): Prepared according to **General Procedure F** to give product as a pale yellow solid (90 mg, 87%). **^{1}H NMR** (500 MHz, $CDCl_3$) δ 8.18 – 8.12 (m, 2H), 7.80 – 7.73 (m, 2H), 7.12 (dp, *J* = 5.9, 2.0 Hz, 3H), 7.07 – 7.00 (m, 2H), 6.84 (ddt, *J* = 8.4, 2.4, 1.2 Hz, 1H), 5.17 (t, *J* = 3.7 Hz, 1H), 5.07 (ddt, *J* = 7.9, 5.8, 1.7 Hz, 1H), 1.93 – 1.85 (m, 1H), 1.80 (dt, *J* = 17.4, 4.9 Hz, 1H), 1.64 (dddt, *J* = 17.4, 12.4, 4.7, 2.3 Hz, 1H), 1.54 (tt, *J* = 12.7, 4.7 Hz, 1H). **^{13}C NMR** (126 MHz, $CDCl_3$) δ 149.87, 144.90, 139.49, 128.43, 128.07, 127.42, 125.91, 124.11, 123.93, 109.71, 56.53, 26.78, 16.75. **HRMS** (ESI) calculated for $[C_{17}H_{17}N_2O_4S]^+$ $(M+H)^+$ requires *m/z* 345.0904, found 345.0900. **Melting Point**: 176-178 ºC.

(±)-2-phenyl-1-((trifluoromethyl)sulfonyl)-1,2,3,4-tetrahydropyridine (6): Prepared according to general **General Procedure F** to give product as a clear and colorless oil (15 mg, 17%). **^{1}H NMR** (500 MHz, $CDCl_3$) δ 7.28 (dd, *J* = 8.1, 6.7 Hz, 2H), 7.25 – 7.19 (m, 1H), 7.17 (d, *J* = 8.5 Hz, 2H), 6.66 – 6.60 (m, 1H), 5.28 (t, *J* = 3.5 Hz, 1H), 5.18 (ddd, *J* = 8.1, 6.0, 1.7 Hz, 1H), 2.18 – 2.08 (m, 1H), 2.02 (tt, *J* = 13.4, 4.7 Hz, 1H), 1.92 (dt, *J* = 18.2, 5.6 Hz, 1H), 1.66 (td, *J* = 12.5, 7.3 Hz, 1H). **^{13}C NMR** (126 MHz, $CDCl_3$) δ 139.16, 128.69, 127.64, 125.26, 122.94, 120.0 (q, *J* = 324.5 Hz), 111.70, 58.11, 27.18, 16.71. **^{19}F NMR** (377 MHz, $CDCl_3$) δ -75.3. **HRMS** (ESI) calculated for $[C_{12}H_{16}F_3N_2O_2S]^+$ $(M+NH_4)^+$ requires *m/z* 309.0879, found 309.0876.

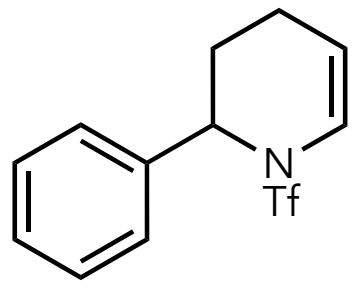

(±)-2-(4-methoxyphenyl)-1-tosyl-1,2,3,4-tetrahydropyridine (7): Prepared according to **General Procedure F** to give product as a white solid (93 mg, 90%). **^{1}H NMR** (500 MHz, $CDCl_3$) δ 7.66 – 7.60 (m, 2H), 7.24 (d, *J* = 8.1 Hz, 2H), 7.15 – 7.08 (m, 2H), 6.88 (m, 1H), 6.83 – 6.75 (m, 2H), 5.11 (t, *J* = 3.6 Hz, 1H), 5.02 (ddt, *J* = 9.3, 5.9, 1.7 Hz, 1H), 3.77 (s, 3H), 2.41 (s, 3H), 1.87 (m, 1H), 1.83 – 1.71 (m, 1H), 1.64 (m, 1H), 1.41 (m, 1H). **^{13}C NMR** (126 MHz, $CDCl_3$) δ 158.7, 143.4, 136.4, 132.5, 129.6, 127.1, 127.0, 124.6, 113.8, 108.5, 55.5, 55.3, 26.3, 21.6, 17.0. **HRMS** (ESI) calculated for $[C_{19}H_{22}NO_3S]^+$ $(M+H)^+$ requires *m/z* 344.1315, found 344.1311. **Melting Point**: 125-127 °C.

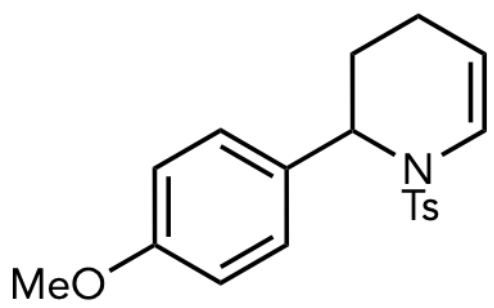

(±)-2-(*o*-tolyl)-1-tosyl-1,2,3,4-tetrahydropyridine (8): Prepared according to **General Procedure F** to provide product as a white solid (85 mg, 87%). **^{1}H NMR** (500 MHz, $CDCl_3$) δ 7.57 – 7.52 (d, *J* = 8.0 Hz, 2H), 7.18 (d, *J* = 8.0 Hz, 2H), 7.12 – 7.04 (m, 2H), 7.03 – 6.89 (m, 3H), 5.38 – 5.33 (m, 1H), 5.10 – 5.03 (m, 1H), 2.38 (s, 3H), 2.36 (s, 3H), 1.89 – 1.81 (m, 1H), 1.72 (m, 2H), 1.60 – 1.50 (m, 1H). **^{13}C NMR** (126 MHz, $CDCl_3$) δ 143.4, 139.0, 136.5, 133.3, 130.4, 129.5, 127.0, 126.9, 126.4, 125.9, 125.5, 106.9, 53.1, 25.0, 21.6, 19.1, 16.7. **HRMS** (ESI) calculated for $[C_{19}H_{21}NO_2SNa]^+$ $(M+Na)^+$ requires *m/z* 350.1185, found 350.1180. **Melting Point**: 129-131 °C

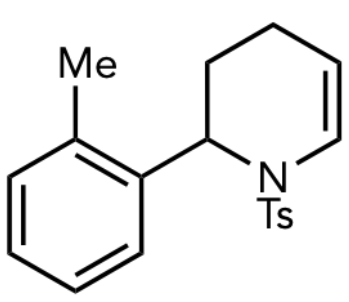

(±)-2-(4-chlorophenyl)-1-tosyl-1,2,3,4-tetrahydropyridine (9): Prepared according to **General Procedure F** to give product as a white solid (84 mg, 80%). **^{1}H NMR** (500 MHz, $CDCl_3$) δ 7.66 – 7.60 (m, 2H), 7.29 – 7.19 (m, 5H), 7.17 – 7.10 (m, 2H), 6.89 (ddt, *J* = 8.4, 2.4, 1.2 Hz, 1H), 5.13 (t, *J* = 3.6 Hz, 1H), 5.04 (ddt, *J* = 7.7, 5.8, 1.7 Hz, 1H), 2.42 (s, 3H), 1.88 (m, 1H), 1.83 – 1.74 (m, 1H), 1.58 (m, 1H), 1.44 (m, 1H). **^{13}C NMR** (126 MHz, $CDCl_3$) δ 143.7, 138.9, 136.2, 132.9, 129.7, 128.5, 127.4, 127.0, 124.5, 108.5, 55.4, 26.2, 21.6, 16.8. **HRMS** (ESI) calculated for $[C_{18}H_{19}ClNO_2S]^+$ $(M+H)^+$ requires *m/z* 348.0819, found 348.0816. **Melting Point**: 119-121 °C

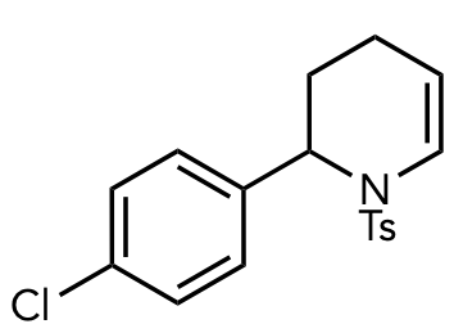

(±)-1-tosyl-2-(4-(trifluoromethyl)phenyl)-1,2,3,4-tetrahydropyridine (10): Prepared according to **General Procedure F** to give product as a white solid (75 mg, 66%). **^{1}H NMR** (500 MHz, $CDCl_3$) δ 7.65 – 7.58 (m, 2H), 7.49 (d, *J* = 8.2 Hz, 2H), 7.30 (d, *J* = 8.0 Hz, 2H), 7.25 – 7.22 (m, 2H), 6.93 (d, *J* = 8.3 Hz, 1H), 5.21 (br s, 1H), 5.05 (m, 1H), 2.40 (s, 3H), 1.96 – 1.87 (m, 1H), 1.87 – 1.76 (m, 1H), 1.63 – 1.46 (m, 2H). **^{13}C NMR** (126 MHz, $CDCl_3$) δ 144.4 (q, *J* = 1.5 Hz), 143.8, 136.1, 129.8, 129.4 (q, *J* = 32.4 Hz), 127.0, 126.4, 125.4 (q, *J* = 3.8 Hz), 124.6, 124.1 (q, *J* = 271.9 Hz), 108.4, 55.7, 26.2, 21.6, 16.8. **^{19}F NMR** (377 MHz, $CDCl_3$) δ -62.54. **HRMS** (ESI) calculated for $[C_{19}H_{19}F_3NO_2S]^+$ $(M+H)^+$ requires *m/z* 344.1315, found 344.1311. **Melting Point**: 94-96 °C

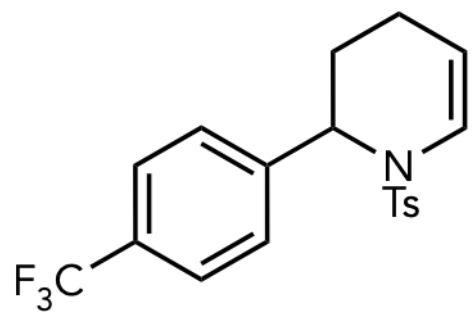

(±)-1-benzyl 3-(3-(1-tosyl-1,2,3,4-tetrahydropyridin-2-yl)benzyl) azetidine-1,3-dicarboxylate (11): Prepared according to **General Procedure F** to give product as a residue (120 mg, 89%). **^{1}H NMR** (500 MHz, $CDCl_3$) δ 7.57 – 7.54 (m, 2H), 7.26 (m, 3H), 7.25 – 7.19 (m, 1H), 7.19 – 7.15 (m, 3H), 7.13 – 7.08 (m, 2H), 7.06 (m, 1H), 6.81 (m, 1H), 5.07 (br s, 1H), 5.02 (m, 4H), 4.99 – 4.91 (m, 1H), 4.11 (dd, *J* = 7.9, 1.9 Hz, 4H), 3.35 (p, *J* = 7.5 Hz, 1H), 2.33 (s, 3H), 1.81 (m, 1H), 1.68 (m, 1H), 1.49 (m, 1H), 1.33 (m, 1H). **^{13}C NMR** (126 MHz, $CDCl_3$) δ 172.1, 156.3, 143.6, 141.0, 136.5, 136.2, 135.6, 129.7, 128.8, 128.6, 128.2, 128.1, 127.0, 126.9, 126.2, 125.8, 124.6, 108.7, 67.0, 66.9, 55.8, 51.8, 32.6, 26.2, 21.6, 16.9. **HRMS** (ESI) calculated for $[C_{31}H_{33}N_2O_6S]^+$ $(M+H)^+$ requires *m/z* 561.2054, found 561.2050.

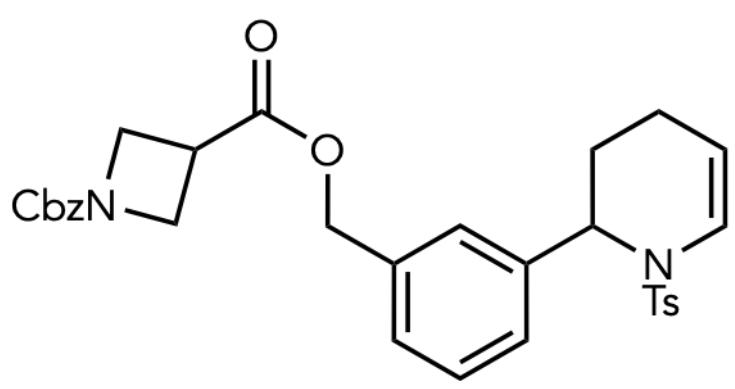

(±)-pyrrolidin-1-yl(3-(1-tosyl-1,2,3,4-tetrahydropyridin-2-yl)phenyl)methanone (12): Prepared according to **General Procedure F** to give product as a sticky residue (86 mg, 70%). **^{1}H NMR** (500 MHz, $CDCl_3$) δ 7.62 – 7.56 (m, 2H), 7.35 – 7.29 (m, 2H), 7.27 – 7.18 (m, 4H), 6.82 (ddt, *J* = 8.3, 2.3, 1.2 Hz, 1H), 5.09 (br s, 1H), 5.01 – 4.93 (m, 1H), 3.56 (t, *J* = 7.0 Hz, 2H), 3.37 – 3.25 (m, 2H), 2.35 (s, 3H), 1.94 – 1.74 (m, 5H), 1.73 – 1.64 (m, 1H), 1.50 (m, 1H), 1.32 (m, 1H). **^{13}C NMR** (126 MHz, $CDCl_3$) δ 169.5, 143.7, 140.3, 137.3, 136.1, 129.7, 128.4, 127.3, 127.0, 126.0, 124.7, 124.4, 108.9, 55.7, 49.6, 46.2, 26.5, 25.9, 24.4, 21.6, 16.9. **HRMS** (ESI) calculated for $[C_{23}H_{27}N_2O_3S]^+$ $(M+H)^+$ requires *m/z* 411.1737, found 411.1734.

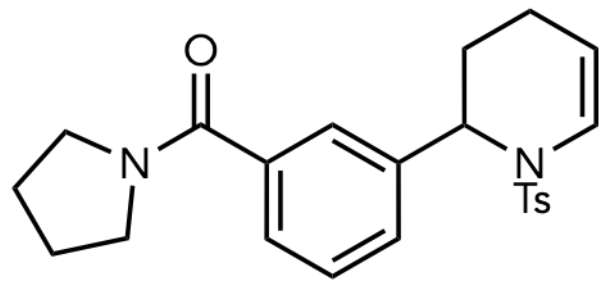

(±)-ethyl (2*S*,3*S*)-2-phenyl-1-tosyl-1,2,3,4-tetrahydropyridine-3-carboxylate (13): Prepared according to **General Procedure F** to give product as a sticky residue (96 mg, 83%, single diastereomer). **^{1}H NMR** (500 MHz, $CDCl_3$) δ 7.59 – 7.54 (m, 2H), 7.28 – 7.13 (m, 7H), 6.91 (d, *J* = 8.5 Hz, 1H), 5.71 (s, 1H), 5.02 – 4.94 (m, 1H), 3.94 (dq, *J* = 10.8, 7.2 Hz, 1H), 3.76 (dq, *J* = 10.8, 7.2 Hz, 1H), 2.88 (m, 1H), 2.37 (m, 4H), 1.83 (ddt, *J* = 17.7, 6.0, 2.4 Hz, 1H), 1.16 (t, *J* = 7.1 Hz, 3H). **^{13}C NMR** (126 MHz, $CDCl_3$) δ 171.4, 143.5, 140.5, 137.2, 129.5, 128.7, 127.5, 127.3, 125.9, 124.6, 104.3,

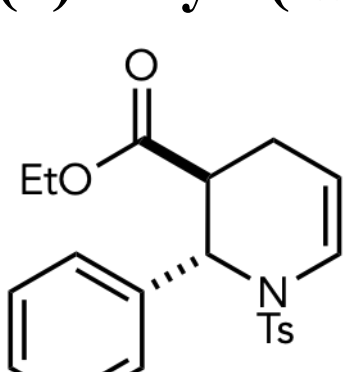

61.2, 57.7, 43.9, 21.6, 18.7, 14.2. **HRMS** (ESI) calculated for $[C_{21}H_{27}N_2O_4S]^+$ $(M+NH_4)^+$ requires *m/z* 403.1686, found 403.1679.

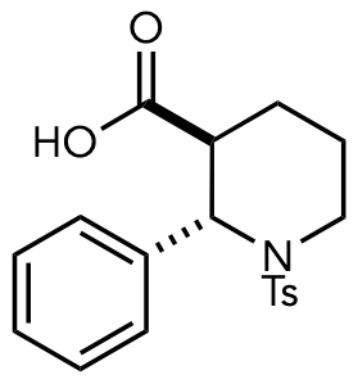

Stereochemical assignment was made by transformation to a known compound with known stereochemistry. Enamide (40 mg, 1 equiv) was dissolved in MeOH (2 mL) followed by addition of Pd/C (22 mg of 10%/weight basis) and reaction was fitted with a hydrogen balloon and stirred overnight. In the morning, the reaction was filtered over celite and concentrated. The residue was dissolved in THF (2 mL) and LiOH (0.31 mL of 1M solution) was added and heated to reflux overnight. The following morning the reaction was acidified with 1M HCl and extracted with EtOAc. The organic layers were dried and concentrated to provide the desired compound (30 mg, 80%, 2 steps). NMR values matched previously reported spectra.[20]

(±)-ethyl 5-(1-tosyl-1,2,3,4-tetrahydropyridin-2-yl)benzofuran-2-carboxylate (14): Prepared according to **General Procedure F** to give product as white solid (114 mg, 89%). **^{1}H NMR** (500 MHz, $CDCl_3$) δ 7.64 – 7.59 (m, 2H), 7.51 – 7.44 (m, 2H), 7.42 (s, 1H), 7.27 (m, 1H), 7.22 (d, *J* = 8.0 Hz, 2H), 6.95 (dt, *J* = 8.4, 1.4 Hz, 1H), 5.27 (br s, 1H), 5.10 – 5.03 (m, 1H), 4.45 (q, *J* = 7.1 Hz, 2H), 2.38 (s, 3H), 1.98 – 1.91 (m, 1H), 1.85 – 1.76 (m, 1H), 1.60 (m, 1H), 1.50 (m, 1H), 1.43 (t, *J* = 7.1 Hz, 3H). **^{13}C NMR** (126 MHz, CDCl3) δ 159.6, 154.9, 146.2, 143.7, 136.3, 136.1, 129.7, 127.1, 127.0, 125.8, 124.7, 120.3, 113.9, 112.3, 108.5, 61.6, 55.9, 26.7, 21.6, 16.9, 14.4. **HRMS** (ESI) calculated for $[C_{23}H_{24}NO_5S]^+$ $(M+H)^+$ requires 426.1370, found 426.1365. **Melting Point**: 124-126 °C.

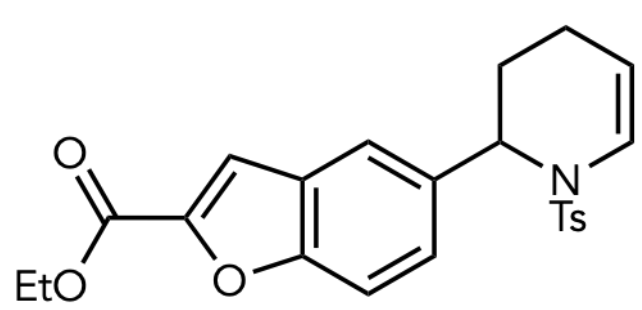

(±)-methyl 1-(4-(1-tosyl-1,2,3,4-tetrahydropyridin-2-yl)benzyl)-1*H*-indazole-3-carboxylate (15): Prepared according to **General Procedure F** to give product as a residue (86 mg, 57%). **^{1}H NMR** (500 MHz, $CDCl_3$) δ 8.24 (m, 1H), 7.61 – 7.54 (m, 2H), 7.43 – 7.34 (m, 2H), 7.31 (m, 1H), 7.16 (d, *J* = 8.0 Hz, 2H), 7.12 (m, 4H), 6.84 (d, *J* = 8.4, 2.4, 1.2 Hz, 1H), 5.65 (s, 2H), 5.11 (br s, 1H), 4.99 (ddt, *J* = 7.4 Hz, 1H), 4.05 (s, 3H), 2.34 (s, 3H), 1.84 (m, 1H), 1.78 – 1.68 (m, 1H), 1.52 (m, 1H), 1.39 (m, 1H). **^{13}C NMR** (126 MHz, $CDCl_3$) δ 163.1, 143.5, 140.6, 140.4, 136.1, 135.0, 134.4, 129.6, 127.3, 127.1, 126.9, 126.4, 124.4, 124.1, 123.3, 122.3, 110.1, 108.5, 55.6, 53.7, 52.1, 26.1, 21.5, 16.8. **HRMS** (ESI) calculated for $[C_{28}H_{31}N_4O_4S]^+$ $(M+NH_4)^+$ requires *m/z* 519.2060, found 519.2056.

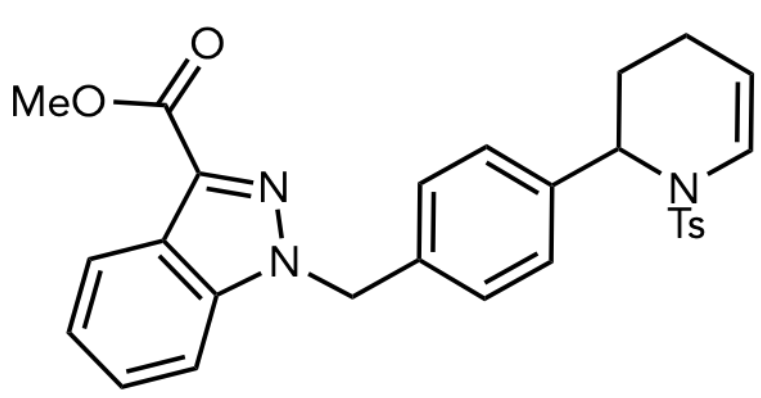

(±)-methyl 1-tosyl-5-(1-tosyl-1,2,3,4-tetrahydropyridin-2-yl)-1*H*-indole-3-carboxylate (16): Prepared according to **General Procedure F** to give product as a white solid (156 mg, 92%). **^{1}H NMR** (500 MHz, $CDCl_3$) δ 8.22 (s, 1H), 7.88 (s, 1H), 7.82 (d, *J* = 8.4 Hz, 3H), 7.59 – 7.53 (m, 2H), 7.32 – 7.22 (m, 3H), 7.12 (d, *J* = 8.0 Hz, 2H), 6.98 – 6.92 (m, 1H), 5.23 (br s, 1H), 5.07 (ddt, *J* = 7.4, 5.9, 1.7 Hz, 1H), 3.90 (s, 3H), 2.38 (s, 3H), 2.33 (s, 3H), 1.95 – 1.87 (m, 1H), 1.78 (m, 1H), 1.61 (m, 1H), 1.49 (m, 1H). **^{13}C NMR** (126 MHz, $CDCl_3$) δ

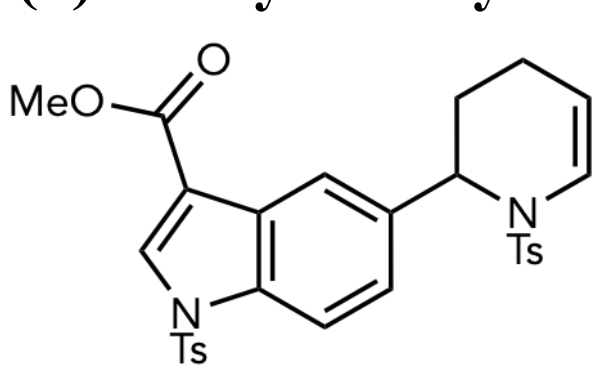

164.0, 146.0, 143.5, 137.0, 136.3, 134.7, 134.0, 132.3, 130.3, 129.6, 127.9, 127.3, 127.0, 124.8, 123.9, 119.4, 113.3, 113.2, 108.5, 56.1, 51.6, 26.8, 21.7, 21.5, 16.9. **HRMS** (ESI) calculated for $[C_{29}H_{29}N_2O_6S_2]^+$ $(M+H)^+$ requires *m/z* 565.1462, found 565.1454. **Melting Point**: 110-120 °C.

(±)-(5-(1-tosyl-1,2,3,4-tetrahydropyridin-2-yl)isoindolin-2-yl)(6-(trifluoromethyl)pyridin-3-yl)methanone (17): Prepared according to **General Procedure F** to give product as an off-white solid (149 mg, 94%). **^{1}H NMR** (500 MHz, $CDCl_3$, mixture of rotamers) δ 8.87 (d, *J* = 2.1 Hz, 1H), 8.01 (m, 1H), 7.73 (d, *J* = 8.0 Hz, 1H), 7.58 (m, 2H), 7.24 – 7.20 (m, 2H), 7.15 – 6.97 (m, 3H), 6.82 (dd, *J* = 26.9, 8.3 Hz, 1H), 5.07 (m, 1H), 5.01 – 4.84 (m, 3H), 4.67 (m, 2H), 2.35 (d, *J* = 8.0 Hz, 3H), 1.82 (m, 1H), 1.72 (m, 1H), 1.49 (m, 1H), 1.34 (m, 1H). **^{13}C NMR** (126 MHz, $CDCl_3$, mixture of rotamers, peaks shown correspond to the major rotamer) δ 166.3, 149.3 (q, *J* = 34.8 Hz), 148.1, 143.6, 140.7, 136.3, 136.1, 136.0, 134.6, 129.7, 126.9, 126.5, 125.7, 124.5, 123.0, 121.2 (q, *J* = 274.4 Hz), 120.5 (m), 120.2, 108.5, 55.7, 54.9, 52.5, 26.2, 21.5, 16.8. **^{19}F NMR** (377 MHz, $CDCl_3$) δ -68.16. **HRMS** (ESI) calculated for $[C_{27}H_{25}F_3N_3O_3S]^+$ $(M+H)^+$ requires *m/z* 528.1563, found 528.1555. **Melting Point**: 101-103 °C.

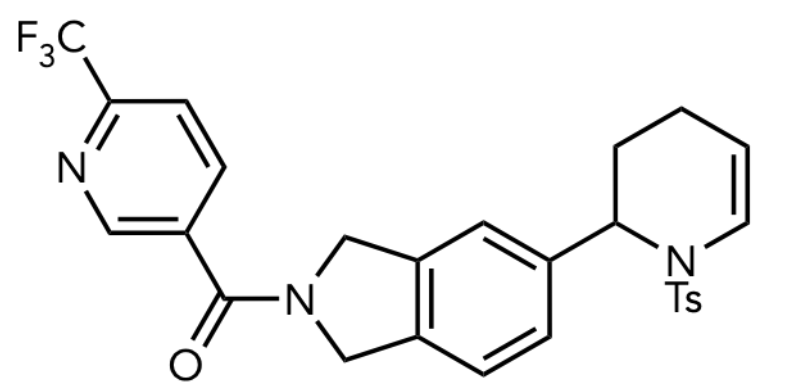

(±)-1-tosyl-2-vinyl-1,2,3,4-tetrahydropyridine (20): Prepared according to **General Procedure F** to provide product as a colorless oil (72 mg, 91%). **^{1}H NMR** (500 MHz, $CDCl_3$) δ 7.70 – 7.65 (d, *J* = 8.0 Hz, 2H), 7.29 (d, *J* = 8.0 Hz, 2H), 6.68 (d, *J* = 8.3 Hz, 1H), 5.65 (ddd, *J* = 17.1, 10.4, 4.7 Hz, 1H), 5.23 (dt, *J* = 17.1, 1.5 Hz, 1H), 5.15 (dt, *J* = 10.4, 1.5 Hz, 1H), 5.02 – 4.94 (m, 1H), 4.54 (m, 1H), 2.41 (s, 3H), 1.94 – 1.75 (m, 2H), 1.68 – 1.59 (m, 1H), 1.27 – 1.13 (m, 1H). **^{13}C NMR** (126 MHz, $CDCl_3$) δ 143.5, 136.4, 135.0, 129.7, 127.1, 123.6, 116.4, 108.4, 54.6, 24.5, 21.6, 17.4. **HRMS** (ESI) calculated for $[C_{14}H_{18}NO_2S]^+$ $(M+H)^+$ requires *m/z* 264.1053, found 264.1049.

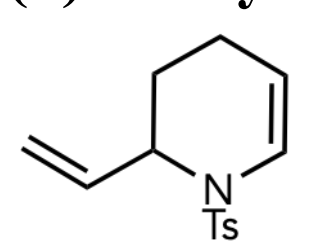

(±)-(*E*)-2-styryl-1-tosyl-1,2,3,4-tetrahydropyridine (21): Prepared according to **General Procedure F** to provide product as a colorless oil (74 mg, 73%). **^{1}H NMR** (500 MHz, $CDCl_3$) δ 7.64 – 7.58 (m, 2H), 7.24 – 7.10 (m, 7H), 6.66 (m, 1H), 6.40 (dd, *J* = 15.9, 1.6 Hz, 1H), 5.86 (dd, *J* = 15.9, 5.8 Hz, 1H), 4.95 (m, 1H), 4.67 (m, 1H), 2.29 (s, 3H), 1.93 – 1.83 (m, 1H), 1.82 – 1.75 (m, 1H), 1.68 (m, 1H), 1.37 – 1.26 (m, 1H). **^{13}C NMR** (126 MHz, $CDCl_3$) δ 143.5, 136.6, 136.6, 131.6, 129.7, 128.5, 127.6, 127.2, 126.6, 126.3, 123.8, 108.3, 54.5, 25.7, 21.6, 17.6. **HRMS** (ESI) calculated for $[C_{20}H_{22}NO_2S]^+$ $(M+H)^+$ requires *m/z* 340.1366, found 340.1362.

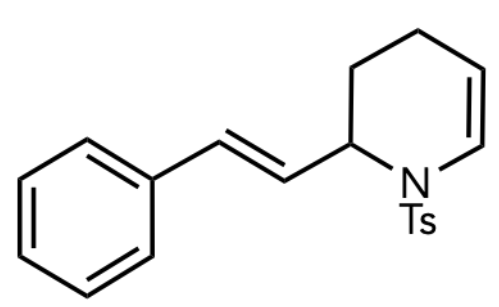

(±)-ethyl (*E*)-3-(1-tosyl-1,2,3,4-tetrahydropyridin-2-yl)acrylate (22): Prepared according to **General Procedure F** to provide product as a colorless oil (94 mg, 93%). Gram-scale modification to general procedure F (with following modifications: 2.5 equiv $Cu(OTf)_2$, 3.0 equiv 2,6-di-*t*Bu-py, 10 equiv *i*PrCN) provided 823 mg of product (83%). **^{1}H NMR** (500 MHz, $CDCl_3$) δ 7.71 – 7.64 (m, 2H), 7.30 (d, *J* = 8.1 Hz, 2H), 6.73 – 6.65 (m, 2H), 5.96 (dd, *J* = 15.5, 1.9 Hz, 1H), 5.02 (m, 1H), 4.71 – 4.65 (m, 1H), 4.17 (m, 2H), 2.42 (s, 3H), 1.88 – 1.80 (m, 2H), 1.75 – 1.68 (m, 1H),

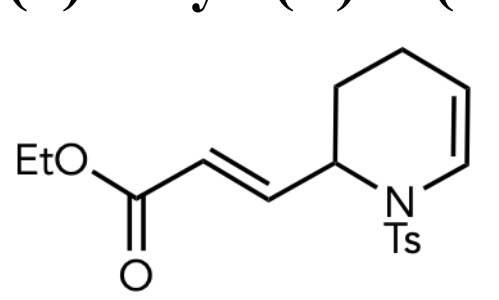

1.28 (t, J = 7.1 Hz, 3H), 1.25 – 1.17 (m, 1H). **^{13}C NMR** (126 MHz, $CDCl_3$) δ 166.1, 144.2, 143.9, 136.0, 129.9, 127.1, 123.4, 123.1, 108.8, 60.6, 53.5, 24.1, 21.6, 17.6, 14.3. **HRMS** (ESI) calculated for $[C_{17}H_{22}NO_4S]^+$ $(M+H)^+$ requires m/z 336.1264, found 336.1259.

(±)-(2*S*,3*R*)-3-((3,5-bis(trifluoromethyl)benzyl)oxy)-2-phenyl-1-tosyl-1,2,3,4-tetrahydropyridine and (±)-(2*S*,3*S*)-3-((3,5-bis(trifluoromethyl)benzyl)oxy)-2-phenyl-1-tosyl-1,2,3,4-tetrahydropyridine (24): Prepared according to **General Procedure F** (0.15 mmol scale) to provide product as a sticky residue (67 mg, 80%).

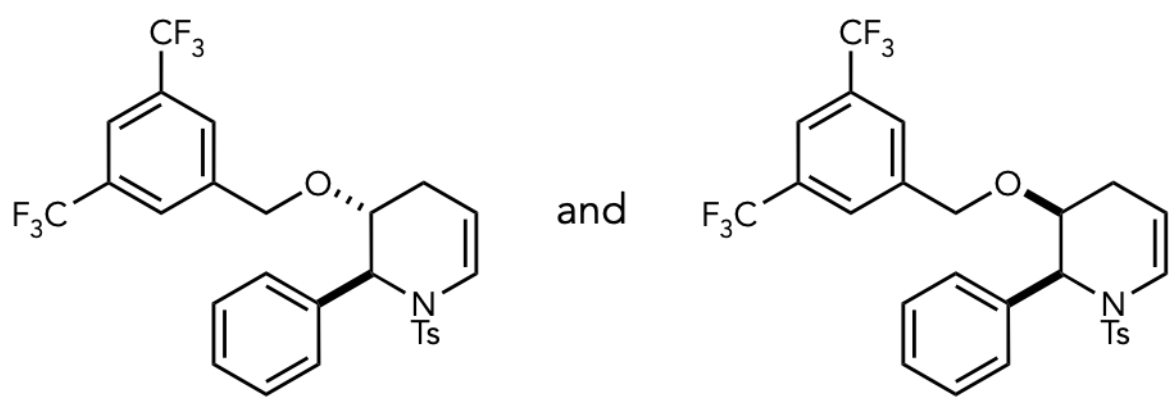

1.8:1 trans:cis

^{1}H NMR (500 MHz, $CDCl_3$, minor diastereomer signals denoted by *) δ 7.80* (s, 1H, p-CH), 7.80 (s, 1H, p-CH), 7.68* (s, 2H, o-CH), 7.67 (d, J = 8.4 Hz, 2H), 7.60 (s, 2H, o-CH), 7.52* (d, J = 8.4 Hz, 2H), 7.34-7.22 (m, 5H), 7.34-7.17* (m, 5H), 7.15* (d, J = 8.0 Hz, 2H), 7.08 (m, 1H), 7.04 (d, J = 8.0 Hz, 2H), 6.90* (m, 1H), 5.36* (d, J = 5.0 Hz, 1H), 5.28 (s, 1H), 4.96* (m, 1H), 4.92 (m, 1H), 4.74* (d, J = 12.8 Hz, 1H), 4.60* (d, J = 12.7 Hz, 1H), 4.43 (s, 2H), 3.87 (m, 1H), 3.63* (m, 1H), 2.37* (s, 3H), 2.30-2.23* (m, 1H), 2.19 (s, 3H), 2.12-2.06 (m, 1H), 1.96-1.89* (m, 1H), 1.93-1.86 (m, 1H). **^{13}C NMR** (125 MHz, $CDCl_3$, minor diastereomer signals denoted by *) δ 143.7*, 143.3, 141.0, 140.8*, 138.5, 137.0, 136.3*, 136.0*, 131.7* (q, J = 33.3 Hz), 131.6 (q, J = 33.3 Hz), 129.6*, 129.3, 128.8, 128.0*, 128.0*, 127.8*, 127.8, 127.1, 127.0*, 127.0 (m), 127.0* (m), 125.9, 125.3*, 124.9, 123.3 (q, J = 272.7Hz), 123.3* (q, J = 272.7 Hz), 121.5 (m), 121.5* (m, overlapped), 103.7*, 102.4, 74.8, 74.6*, 69.1*, 69.0, 59.0, 58.1*, 24.4*, 22.5, 21.5*, 21.3. **^{19}F NMR** (377 MHz, $CDCl_3$) δ -62.76, -62.87. **HRMS** (ESI) calculated for $[C_{27}H_{24}F_6NO_3S]^+$ $(M+H)^+$ requires m/z 556.1376, found 556.1371.

(±)-(2*S*,3*R*)-3-((3,5-bis(trifluoromethyl)benzyl)oxy)-2-phenyl-1-tosylpiperidine and (±)-(2*S*,3*S*)-3-((3,5-bis(trifluoromethyl)benzyl)oxy)-2-phenyl-1-tosylpiperidine (25): To a vial charged with enamide (60 mg, 1 equiv, 0.11 mmol) was added DCM (1.5 mL), TFA (33 μL, 4 equiv, 0.42 mmol) and Et_3SiH (69 μL, 4 equiv, 0.43 mmol) and the reaction was stirred at room temperature. When complete by TLC, reaction was concentrated under reduced pressure and purified over silica with 0-20% EtOAc in hexanes to afford product as a colorless residue (56 mg, 93%).

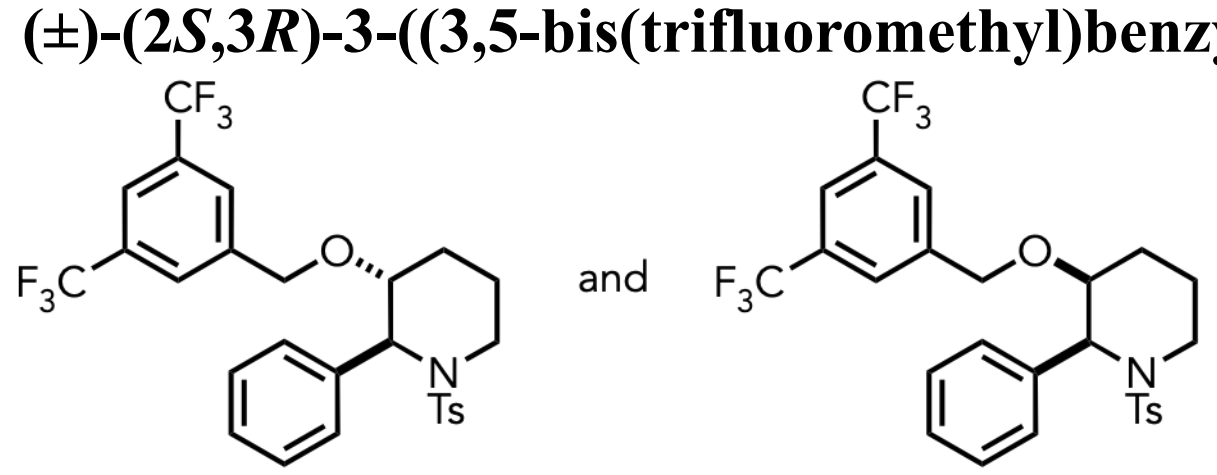

1.8:1 trans:cis

^{1}H NMR (500 MHz, $CDCl_3$, minor diastereomer peaks denoted by *) δ 7.76 (s, 1H), 7.72 (s, 2H), 7.70* (s, 1H), 7.64 (d, J = 8.0 Hz, 2H), 7.57* (s, 2H), 7.50* (d, J = 8.0 Hz, 2H), 7.42* (m, 2H), 7.31 – 7.22* (m, 3H, overlapped), 7.27-7.22 (m, 2H, overlapped), 7.20- 7.14 (m, 3H), 7.10* (d, J = 8.0 Hz, 2H), 6.98 (d, J = 8.0 Hz, 2H), 5.44* (m, 1H), 5.43 (br s, 1H), 4.72 (d, J = 12.5 Hz, 1H), 4.64* (d, J = 12.5 Hz, 1H), 4.56* (d, J = 12.4 Hz, 1H), 4.52 (d, J = 12.5 Hz, 1H), 4.05 (q, J = 2.9 Hz, 1H), 3.75 – 3.61* (m, 1H, overlapped), 3.75 – 3.61 (m, 1H, overlapped), 3.75 – 3.61* (m, 1H, overlapped), 3.13 (td, J = 13.3, 3.1 Hz, 1H), 2.87* (td, J = 13.4,

3.5 Hz, 1H), 2.31* (s, 3H), 2.21 (s, 3H), 1.87-1.21 (m, 4H, overlapped with minor diastereomer), 1.87-1.21* (m, 4H, overlapped with major diastereomer). **^{13}C NMR** (126 MHz, $CDCl_3$, minor diastereomer peaks denoted by *) δ 143.3*, 142.9, 141.1, 140.9*, 138.2, 137.8*, 137.4, 136.4*, 131.8 (q, J = 33.2 Hz), 131.7* (q, J = 33.2 Hz), 129.6*, 129.2, 129.0*, 128.9, 128.4, 127.6*, 127.4, 127.4*, 127.3 (m), 127.2* (m), 127.0*, 126.9, 123.4 (q, J = 272.6 Hz), 123.4* (q, J = 272.6 Hz), 121.6 (m), 121.6* (m), 78.2*, 75.9, 69.4, 69.2*, 58.2, 57.4*, 41.7, 41.0*, 25.6*, 24.2*, 24.0, 21.5*, 21.4, 18.8. **^{19}F NMR** (377 MHz, $CDCl_3$) δ -62.79, -62.89. **HRMS** (ESI) calculated for $[C_{27}H_{26}F_6NO_3S]^+$ $(M+H)^+$ requires m/z 558.1532, found 558.1527.

G. Experimental procedure, isolation, and characterization for pyrrolidine products

General Procedure G: *Cyclobutylamine to Pyrrolidine*

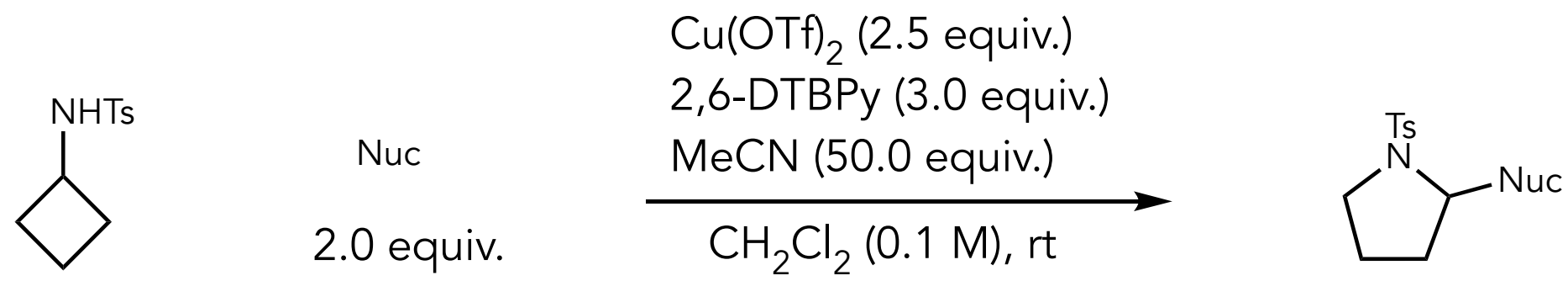

An oven-dried 8 mL vial charged with a magnetic stir bar and cyclobutylamine substrate (1.0 equiv, 0.30 mmol) was brought into a nitrogen-filled glovebox. $Cu(OTf)_2$ (2.5 equiv, 271 mg, 0.750 mmol) was added to the vial, followed by 2,6-di-tert-butylpyridine (3.0 equiv, 195 mL, 0.900 mmol), anhydrous MeCN (50.0 equiv, 783 mL, 15.0 mmol), and CH_2Cl_2 (3.0 mL, 0.1 M). The nucleophile (2.0 equiv, 0.600 mmol) was then added in a single portion. The vial was capped, brought out of the glovebox, and allowed to stir for 1–24 hours at room temperature. Upon completion of the reaction, the solution was diluted with EtOAc, washed 1x with aqueous NH_4Cl, washed 1x with brine, dried over Na_2SO_4, and concentrated under reduced pressure. The crude mixture was then purified using flash column chromatography.

(±)-*cis*-2-ethoxy-5-phenyl-1-tosylpyrrolidine (36): Prepared according to **General Procedure G** with **35** (90.4 mg, 0.300 mmol) as the substrate. The reaction was stirred for 5 h, and the crude material was purified by flash column chromatography on silica gel (0–20 EtOAc:Hexanes). **36** was afforded as a colorless oil (92.5 mg, 89% yield, >20:1 dr). **^{1}H NMR** (500 MHz, $CDCl_3$) δ 7.57 (d, *J* = 8.4 Hz, 2H), 7.30 – 7.23 (m, 4H), 7.20 (m, 3H), 5.38 (d, *J* = 4.9 Hz, 1H), 4.50 (dd, *J* = 9.6, 7.4 Hz, 1H), 3.98 (dq, *J* = 9.4, 7.1 Hz, 1H), 3.64 (dq, *J* = 9.4, 6.9 Hz, 1H), 2.38 (s, 3H), 2.27 – 2.18 (m, 1H), 2.09 (tdd, *J* = 12.9, 9.7, 6.4 Hz, 1H), 1.91 (dd, *J* = 12.6, 6.2 Hz, 1H), 1.38 (tdd, *J* = 12.9, 7.6, 5.1 Hz, 1H), 1.28 (t, *J* = 7.0 Hz, 3H). **^{13}C NMR** (126 MHz, $CDCl_3$) δ 143.5, 142.9, 136.0, 129.6, 128.4, 127.6, 127.2, 127.0, 91.2, 64.9, 62.9, 35.0, 33.1, 21.6, 15.1. The spectral data matched those previously reported.[21]

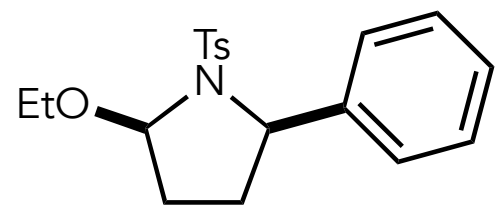

2-ethoxy-1-tosylpyrrolidine (37): Prepared according to **General Procedure G** with **S49** (67.6 mg, 0.300 mmol) as the substrate. The reaction was stirred for 5 h, and the crude material was purified by flash column chromatography on silica gel (0–20% EtOAc:Hexanes). **37** was afforded as a white solid (61.0 mg, 76% yield). **^{1}H NMR** (500 MHz, $CDCl_3$) δ 7.72 (d, *J* = 8.3 Hz, 2H), 7.30 (d, *J* = 7.9 Hz, 2H), 5.21 (d, *J* = 5.1 Hz, 1H), 3.81 (dq, *J* = 9.6, 7.1 Hz, 1H), 3.55 (dq, *J* = 9.6, 7.0 Hz, 1H), 3.41 (ddd, *J* = 9.8, 8.5, 2.6 Hz, 1H), 3.15 (td, *J* = 9.7, 7.5 Hz, 1H), 2.42 (s, 3H), 2.03 (dddd, *J* = 15.7, 12.2, 7.9, 5.0 Hz, 1H), 1.85 (ddd, *J* = 12.9, 6.7, 1.9 Hz, 1H), 1.75 (dtt, *J* = 12.3, 7.7, 2.4 Hz, 1H), 1.41 (tdd, *J* = 12.6, 7.8, 5.2 Hz, 1H), 1.19 (t, *J* = 7.0 Hz, 3H). **^{13}C NMR** (126 MHz, $CDCl_3$) δ 143.5, 136.2, 129.8, 127.6, 90.3, 63.3, 47.4, 33.1, 23.4, 21.7, 15.2. **HRMS** (ESI) calculated for $[C_{13}H_{19}NO_3Na]^+$ $[M+Na]^+$ requires m/z 292.0978, found 292.0978. **Melting Point:** 82–83 °C.

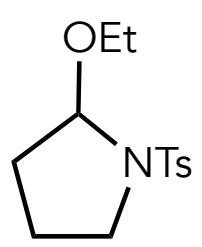

2-(3-((1-tosylpyrrolidin-2-yl)oxy)propyl)isoindoline-1,3-dione (38): Prepared according to **General Procedure G** with **S49** (67.6 mg, 0.300 mmol) as the substrate. The reaction was stirred for 5 h, and the crude material was purified by flash column chromatography on silica gel (15–35% EtOAc:Hexanes). **38** was afforded as a white foam (74.6 mg, 58% yield). **^{1}H NMR** (500 MHz, $CDCl_3$) δ 7.83 (dd, *J* = 5.4, 3.0 Hz, 2H), 7.72 – 7.67 (m, 4H), 7.29 (d, *J* = 8.0 Hz, 2H), 5.16 (d, *J* = 5.1 Hz, 1H), 3.85 – 3.80 (m, 1H), 3.80 – 3.72 (m, 2H), 3.57 – 3.49 (m, 1H), 3.38 (ddd, *J* = 10.6, 8.5, 2.5 Hz, 1H), 3.10 (td, *J* = 9.7, 7.5 Hz, 1H), 2.41 (s, 3H), 2.02 – 1.90 (m, 3H), 1.77 (ddd, *J* = 12.8, 6.8, 1.9 Hz, 1H), 1.73 – 1.65 (m, 1H), 1.33 (tdd, *J* = 12.4, 7.6, 5.0 Hz, 1H). **^{13}C NMR** (126 MHz, $CDCl_3$) δ 168.0, 143.5, 136.0, 134.0, 132.4, 129.8, 127.5, 123.3, 90.6, 65.1, 47.5, 35.7, 32.9, 28.5, 23.3, 21.6. **HRMS** (ESI) calculated for $[C_{22}H_{24}N_2O_5SNa]^+$ $[M+Na]^+$ requires m/z 451.1298, found 451.1289.

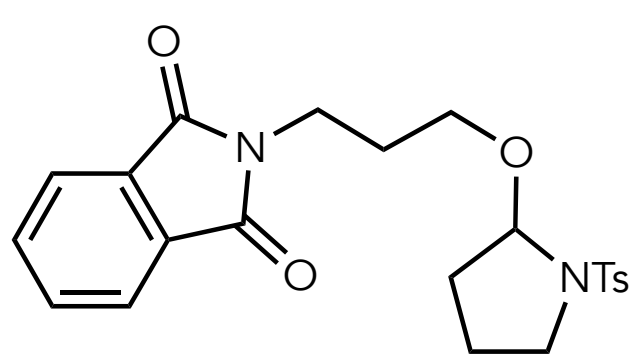

4-methoxy-*N*-(1-tosylpyrrolidin-2-yl)benzenesulfonamide (39): Prepared according to **General Procedure G** with **S49** (67.6 mg, 0.300 mmol) as the substrate. The reaction was stirred for 1 h, and the crude material was purified by flash column chromatography on silica gel (15–20% Acetone:Toluene). **39** was afforded as a white solid (93.6 mg, 76% yield). **^{1}H NMR** (500 MHz, $CDCl_3$) δ 7.88 (d, *J* = 8.9 Hz, 2H), 7.30 (d, *J* = 8.0 Hz, 2H), 7.20 (d, *J* = 8.0 Hz, 2H), 7.07 (d, *J* = 9.0 Hz, 2H), 4.94 (d, *J* = 2.8 Hz, 1H), 4.54 (ddd, *J* = 6.1, 3.0, 1.6 Hz, 1H), 3.92 (s, 3H), 3.53 (ddd, *J* = 9.7, 7.4, 2.6 Hz, 1H), 2.99 (td, *J* = 9.6, 6.5 Hz, 1H), 2.41 (s, 3H), 2.24 (ddt, *J* = 13.1, 6.3, 2.2 Hz, 1H), 2.06 – 1.91 (m, 1H), 1.78 – 1.68 (m, 1H), 1.61 (ddt, *J* = 13.1, 11.3, 6.6 Hz, 1H). **^{13}C NMR** (126 MHz, $CDCl_3$) δ 163.4, 144.2, 134.0, 131.2, 130.1, 129.9,

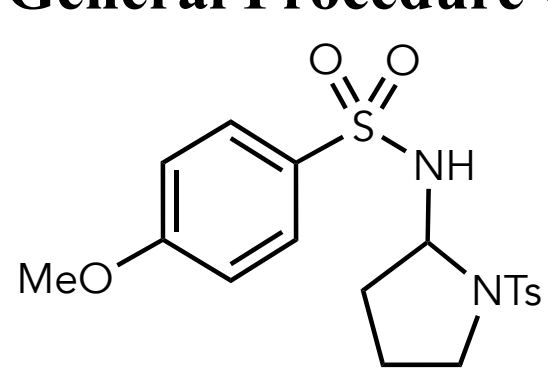

127.4, 114.3, 69.8, 55.9, 48.9, 34.2, 22.8, 21.7. **HRMS** (ESI) calculated for $[C_{18}H_{21}N_2O_5S_2]^-$ [M–H]$^-$ requires m/z 409.0897, found 409.0900. **Melting Point:** 114–116 °C.

3-(1-tosylpyrrolidin-2-yl)oxazolidin-2-one (40): Prepared according to **General Procedure G** with **S49** (67.6 mg, 0.300 mmol) as the substrate. The reaction was stirred for 1 h, and the crude

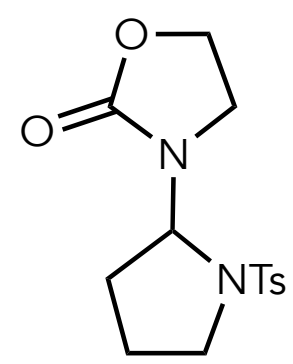

material was purified by flash column chromatography on silica gel (60–80% EtOAc:Hexanes). **40** was afforded as a white solid (54.6 mg, 53% yield). **^{1}H NMR** (500 MHz, $CDCl_3$) δ 7.85 (d, *J* = 8.4 Hz, 2H), 7.35 (d, *J* = 7.9 Hz, 2H), 5.37 (dd, *J* = 7.8, 4.7 Hz, 1H), 4.43 – 4.35 (m, 1H), 4.38 – 4.28 (m, 1H), 3.86 (dt, *J* = 9.5, 7.7 Hz, 1H), 3.69 – 3.63 (m, 1H), 3.48 (ddd, *J* = 9.1, 8.0, 6.0 Hz, 1H), 3.16 (dt, *J* = 10.0, 7.2 Hz, 1H), 2.44 (s, 3H), 2.04 – 1.79 (m, 3H), 1.68 – 1.57 (m, 1H). **^{13}C NMR** (126 MHz, $CDCl_3$) δ 158.0, 144.2, 133.6, 130.0, 128.2, 68.9, 62.5, 49.2, 39.8, 31.4, 23.0, 21.7. **HRMS** (ESI) calculated for $[C_{14}H_{19}N_2O_4S]^+$ [M+H]$^+$ requires m/z 311.1060, found 311.1055. **Melting Point:** 160–161 °C.

(±)-5-ethoxy-1-tosylpyrrolidin-3-yl benzoate (41): Prepared according to **General Procedure G**

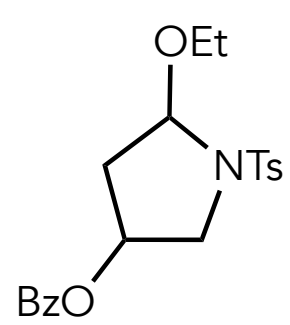

with **S50** (104.0 mg, 0.300 mmol) as the substrate. The reaction was stirred for 24 h, and the crude material was purified by flash column chromatography on silica gel (10–35% EtOAc:Hexanes). **41** was isolated as a mixture of diastereomers and afforded a colorless oil (63.6 mg, 54% yield, 1.4:1 d.r.). The *trans*-isomer was assigned as the major isomer, and peaks overlapping with the minor isomer were denoted with an asterisk. **^{1}H NMR** (500 MHz, $CDCl_3$) δ 8.00 – 7.97* (m, 1.4H), 7.82 (dd, *J* = 8.3, 1.4 Hz, 2H), 7.78 – 7.71* (m, 3.4H), 7.56* (ddq, *J* = 9.6, 5.3, 1.9 Hz, 1.7H), 7.41* (td, *J* = 7.8, 3.8 Hz, 3.4H), 7.31* (d, *J* = 7.9 Hz, 1.4H), 7.21 (d, *J* = 7.9 Hz, 2H), 5.48 (qd, *J* = 6.1, 3.4 Hz, 1H), 5.43 (dd, *J* = 5.9, 1.8 Hz, 1H), 5.37 (d, *J* = 5.6 Hz, 0.7H), 5.31 (tt, *J* = 9.1, 3.5 Hz, 0.7H), 3.89 – 3.74* (m, 3.4H), 3.61 – 3.52* (m, 3.4H), 2.43 (s, 2.1H), 2.39 (ddd, *J* = 12.4, 6.8, 1.8 Hz, 1H), 2.32 (s, 3H), 2.18 (d, *J* = 15.6 Hz, 0.7H), 2.04 (dt, *J* = 13.9, 5.8 Hz, 1H), 1.95 (dt, *J* = 14.6, 6.1 Hz, 0.7H), 1.20* (m, 5.1H). **^{13}C NMR** (126 MHz, $CDCl_3$) δ 166.1, 166.0, 143.8, 143.6, 136.4, 135.8, 133.3, 133.2, 129.8, 129.8, 129.7, 129.6, 128.3, 127.4, 89.7, 89.6, 73.6, 72.7, 63.6, 63.5, 53.1, 51.8, 39.4, 38.9, 21.5, 21.5, 15.0, 15.0. **HRMS** (ESI) calculated for $[C_{20}H_{23}NO_5SNa]^+$ [M+Na]$^+$ requires m/z 412.1189, found 412.1189.

(±)-methyl 5-ethoxy-1-tosylpyrrolidine-3-carboxylate (42): Prepared according to **General Procedure G** with **S51** (85.0 mg, 0.300 mmol) as the substrate. The reaction was stirred for 24 h,

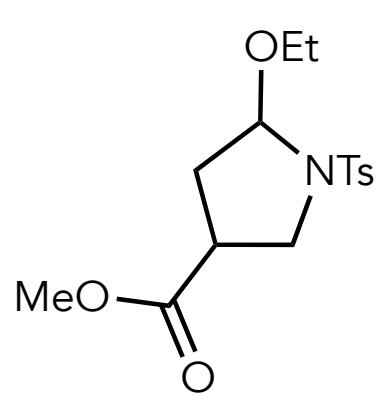

and the crude material was purified by flash column chromatography on silica gel (0–25% Acetone:Hexanes). **42** was isolated as a mixture of separable diastereomers and afforded a colorless oil (41.4 mg, 42% yield, 1.7:1 d.r.). *Trans isomer (major)* **^{1}H NMR** (500 MHz, $CDCl_3$) δ 7.72 (d, *J* = 8.4 Hz, 2H), 7.31 (d, *J* = 7.9 Hz, 2H), 5.22 (d, *J* = 4.9 Hz, 1H), 3.80 (dq, *J* = 9.6, 7.1 Hz, 1H), 3.67 – 3.58 (m, 4H), 3.53 (dq, *J* = 9.5, 7.0 Hz, 1H), 3.44 – 3.33 (m, 2H), 2.42 (s, 3H), 2.09 (dd, *J* = 12.6, 5.9 Hz, 1H), 1.61 (dt, *J* = 12.3, 4.9 Hz, 1H), 1.18 (t, *J* = 7.1 Hz, 3H). **^{13}C NMR** (126 MHz, $CDCl_3$) δ 172.9, 143.9, 135.6, 129.9, 127.6, 90.0, 63.4, 52.3, 49.0, 41.6, 36.5, 21.7, 15.1. *Cis-isomer (minor)* **^{1}H NMR** (500 MHz, $CDCl_3$) δ 7.73 (d, *J* = 8.4 Hz, 2H), 7.30 (d, *J* = 8.6 Hz, 2H), 5.26 (dd, *J* = 5.2, 1.5 Hz, 1H), 3.83 – 3.76 (m, 1H), 3.74 – 3.66 (m, 1H), 3.64 (s, 3H), 3.53 – 3.43 (m, 2H), 2.82 (dddd, *J* = 9.1, 7.8, 5.1, 4.0 Hz, 1H), 2.43 (s, 3H), 2.30 (ddd, *J* = 13.4, 4.0, 1.5 Hz, 1H), 1.88 (ddd, *J* = 13.9, 9.2, 5.3 Hz, 1H), 1.11 (t, *J* = 7.0 Hz, 3H). **^{13}C NMR** (126 MHz, $CDCl_3$) δ 172.6, 143.8, 136.4, 129.9, 127.5, 89.6, 63.0, 52.3, 48.2, 42.0, 36.1, 21.7, 15.0. **HRMS** (ESI) calculated for $[C_{15}H_{21}NO_5SNa]^+$ [M+Na]$^+$ requires m/z 350.1033, found 350.1032.

***tert*-butyl 3-ethoxy-2-tosyl-2,8-diazaspiro[4.5]decane-8-carboxylate (43):** Prepared according to **General Procedure G** with **S52** (118 mg, 0.300 mmol) as the substrate. The reaction was stirred for 24 h, and the crude material was purified by flash column chromatography on silica gel (10–35% Acetone:Hexanes). **43** was afforded as a white foam (64.4 mg, 49% yield). **^{1}H NMR** (500 MHz, $CDCl_3$) δ 7.67 (d, *J* = 8.3 Hz, 2H), 7.23 (d, *J* = 7.9 Hz, 2H), 5.19 (d, *J* = 5.8 Hz, 1H), 3.66 (m, 1H), 3.42 (dq, *J* = 9.5, 7.0 Hz, 1H), 3.35 (dt, *J* = 13.7, 5.5 Hz, 1H), 3.25 (dt, *J* = 13.8, 5.5 Hz, 1H), 3.22 – 3.06 (m, 4H), 2.36 (s, 3H), 1.78 (dd, *J* = 13.4, 1.5 Hz, 1H), 1.59 (dd, *J* = 6.5, 5.0 Hz, 2H), 1.55 – 1.47 (m, 1H), 1.36 (s, 9H), 1.20 – 1.14 (m, 2H), 1.08 (t, *J* = 7.0 Hz, 3H). **^{13}C NMR** (126 MHz, $CDCl_3$) δ 154.8, 143.6, 136.5, 129.7, 127.4, 90.6, 79.6, 63.7, 56.8, 44.1, 41.1, 36.6, 35.9, 28.5, 21.6, 15.2. **HRMS** (ESI) calculated for $[C_{22}H_{34}N_2O_5SNa]^+$ $[M+Na]^+$ requires m/z 461.2081, found 461.2074.

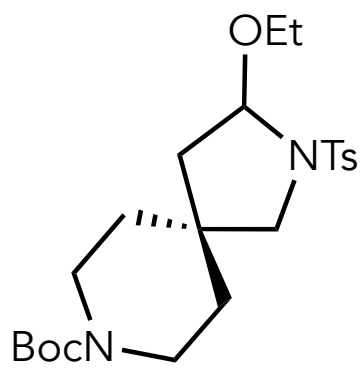

2-ethoxy-1-tosyl-1,2,3,3a,6,6a-hexahydrocyclopenta[*b*]pyrrole (44): Prepared according to **General Procedure G** with **S53** (85.0 mg, 0.300 mmol) as the substrate. The reaction was stirred for 5 h, and the crude material was purified by flash column chromatography on silica gel (0–20% EtOAc:Hexanes). **44** was isolated as an inseparable mixture of diastereomers and afforded a colorless oil (52.3 mg, 58% yield, 7.7:1 d.r.). **^{1}H NMR** (500 MHz, $CDCl_3$) Only major diastereomer (*endo*) reported δ 7.71 (d, *J* = 8.3 Hz, 2H), 7.30 (d, *J* = 8.5 Hz, 2H), 5.62 – 5.54 (m, 2H), 5.25 (d, *J* = 5.6 Hz, 1H), 4.27 (td, *J* = 7.4, 1.7 Hz, 1H), 3.73 (dq, *J* = 9.9, 7.1 Hz, 1H), 3.50 (dq, *J* = 9.9, 7.0 Hz, 1H), 3.22 – 3.16 (m, 1H), 2.80 – 2.69 (m, 1H), 2.62 (d, *J* = 17.1 Hz, 1H), 2.43 (s, 3H), 1.87 (d, *J* = 13.3 Hz, 1H), 1.49 (ddd, *J* = 13.3, 9.2, 5.7 Hz, 1H), 1.13 (t, *J* = 7.0 Hz, 3H). **^{13}C NMR** (126 MHz, $CDCl_3$) δ 143.4, 136.7, 132.8, 129.7, 128.0, 127.2, 91.0, 62.8, 62.6, 49.8, 41.9, 37.4, 21.5, 14.9. **^{1}H NMR** (500 MHz, $CDCl_3$) δ 7.71 (d, *J* = 8.3 Hz, 2H), 7.30 (d, *J* = 8.5 Hz, 2H), 5.62 – 5.54 (m, 2H), 5.25 (d, *J* = 5.6 Hz, 1H), 4.27 (td, *J* = 7.4, 1.7 Hz, 1H), 3.73 (dq, *J* = 9.9, 7.1 Hz, 1H), 3.50 (dq, *J* = 9.9, 7.0 Hz, 1H), 3.22 – 3.16 (m, 1H), 2.80 – 2.69 (m, 1H), 2.62 (d, *J* = 17.1 Hz, 1H), 2.43 (s, 3H), 1.87 (d, *J* = 13.3 Hz, 1H), 1.49 (ddd, *J* = 13.3, 9.2, 5.7 Hz, 1H), 1.13 (t, *J* = 7.0 Hz, 3H). **^{13}C NMR** (126 MHz, $CDCl_3$) δ 143.4, 136.7, 132.8, 129.7, 128.0, 127.2, 91.0, 62.8, 62.6, 49.8, 41.9, 37.4, 21.5, 14.9. **HRMS** (ESI) calculated for $[C_{16}H_{21}NO_3SNa]^+$ $[M+Na]^+$ requires m/z 330.1134, found 330.1136.

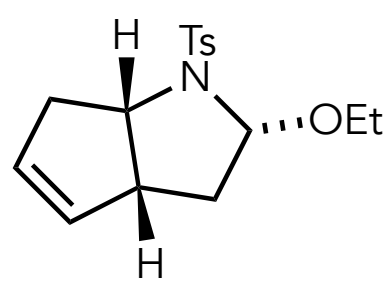

H. N-Chloramine reactivity study

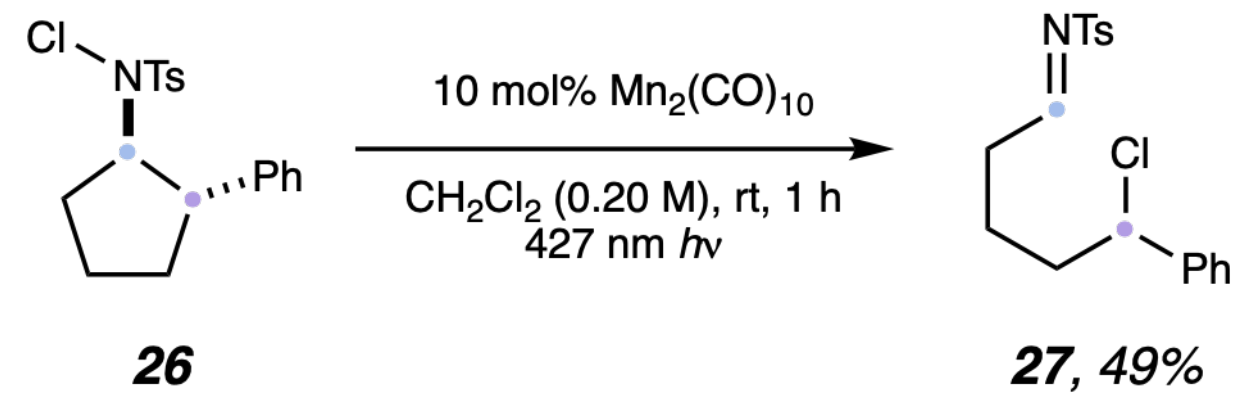

Followed a modified procedure by Zhang.[22] *N*-Chloro sulfonamide **26** (70.0 mg, 0.20 mmol, 1.0 equiv) and $Mn_2(CO)_{10}$ (7.80 mg, 0.02 mmol, 0.10 equiv) were place in a 4 mL flame-dried vial under N_2. Then CD_2Cl_2 (1.0 mL) was added with a syringe. The reaction mixture was stirred and irradiated with a 40 W PR160L 427 nm Kessil Lamp. After one hour the reaction mixture was filtered through a celite plug which was rinsed with CD_2Cl_2 (1.0 mL), then CH_2Br_2 (28.1 mg, 0.16 mmol) was added as an internal standard. The reaction was analyzed without further purification. The yield of **27** was calculated by ^{1}H NMR. **^{1}H NMR yield**: 49%. **^{1}H NMR** (500 MHz, CD_2Cl_2) δ 8.53 (t, J = 4.0 Hz, 1H), 7.76 (d, J = 7.8 Hz, 2H), 7.38 – 7.31 (m, 7H), 4.87 – 4.80 (m, 1H), 2.55 (td, J = 7.4, 4.0 Hz, 2H), 2.44 (s, 3H), 2.12 (qd, J = 9.1, 4.6 Hz, 1H), 2.08 – 1.99 (m, 1H), 1.83 (ddt, J = 13.7, 8.6, 4.6 Hz, 1H), 1.70 – 1.61 (m, 1H). **^{13}C NMR** (126 MHz, CD_2Cl_2) δ 178.01, 145.43, 141.81, 134.94, 130.26, 129.07, 128.80, 128.44, 127.26, 39.35, 35.53, 22.40, 21.79, 19.86. **HRMS** (ESI) calculated for $[C_{18}H_{21}ClNO_2S]^+$ $[M+H]^+$ requires m/z 350.0976, found 350.0969.

I. Hofmann-Löffler-Freytag reactivity study

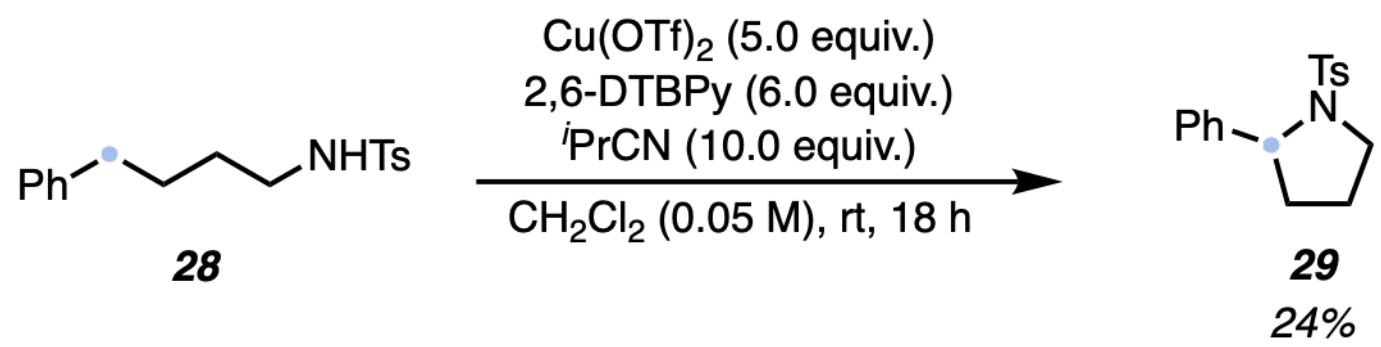

4-methyl-*N*-(4-phenylbutyl)benzenesulfonamide (28): Prepared according to a modified

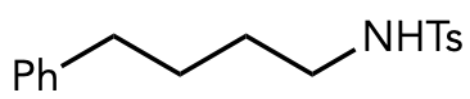

General Procedure E using 4-phenylbutan-1-amine (250 mg, 1 equiv, 1.68 mmol) and triethylamine (281 μL, 1.2 equiv, 2.01 mmol) and was purified over silica with 0-20% ethyl acetate in hexanes to afford **28** as a white solid (460 mg, 91%). **^{1}H NMR** (500 MHz, CDCl3) δ 7.75 (d, J = 8.3 Hz, 2H), 7.33 – 7.26 (m, 4H), 7.23 – 7.17 (m, 1H), 7.15 – 7.11 (m, 2H), 4.37 (t, J = 6.2 Hz, 1H), 2.98 (q, J = 6.7 Hz, 2H), 2.58 (t, J = 7.5 Hz, 2H), 1.68 – 1.56 (m, 3H), 1.55 – 1.47 (m, 2H). NMR values matched previously reported spectra.[23]

2-phenyl-1-tosylpyrrolidine (29): Prepared according to **General Procedure F** using

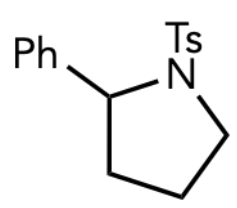

sulfonamide **28** and purified over silica with 0-20% ethyl acetate in hexanes to afford **29** as a white solid (21.7 mg, 24%). **^{1}H NMR** (500 MHz, CDCl3) δ 7.70 – 7.64 (m, 2H), 7.32 – 7.26 (m, 6H), 7.25 – 7.18 (m, 1H), 4.79 (dd, J = 8.0, 3.7 Hz, 1H), 3.61 (ddd, J = 10.2, 7.2, 4.7 Hz, 1H), 3.43 (dt, J = 10.2, 7.3 Hz, 1H), 2.42 (s, 3H), 2.06 – 1.94 (m, 1H), 1.92 – 1.77 (m, 2H), 1.72 – 1.62 (m, 1H). NMR values matched previously reported spectra.[Error! Bookmark not defined.]

J. UV-vis studies

I. UV-vis study of reaction components in MeCN

Objective: Study the absorption spectra in MeCN.

Operational Notes and Procedure: Three stock solutions of $Cu(OTf)_2$, 2,6-di-tertbutylpyridine and **1** were prepared in anhydrous MeCN. MeCN was used rather than CH_2Cl_2 because of improved solubility. These solutions were prepared in a glove box and used to produce mixtures of all reaction components across separate quartz cuvettes for UV-Vis analysis. Volumetric glassware and syringes were used to ensure accurate measurements.

Solution A:
$Cu(OTf)_2$ – (362 mg, 1.00 mmol)
MeCN – (10.0 mL)

Solution B:
2,6-di-tertbutylpyridine – (216 µL, 0.96 mmol)
MeCN – (10.0 mL)

Solution C:
1 – (63 mg, 0.20 mmol)
MeCN – (10.0 mL)

Upon preparing the solution, aliquots were separately added into dried 2 mL volumetric flasks in the desired ratio (**Table S6**). Once the aliquots were added, the volumetric flasks were diluted to 2 mL.

Table S6. Solutions prepared for UV-Vis analysis of the reaction components

Cuvette	[$Cu(OTf)_2$] mM	[2,6-di-tBu-py] mM	[1] mM
1	25	0	0
2	25	30	0
3	25	30	5
4	25	0	5

UV-Vis measurements for each solution were collected 3 times after a blank subtraction. The results are summarized in **Fig. S1** below.

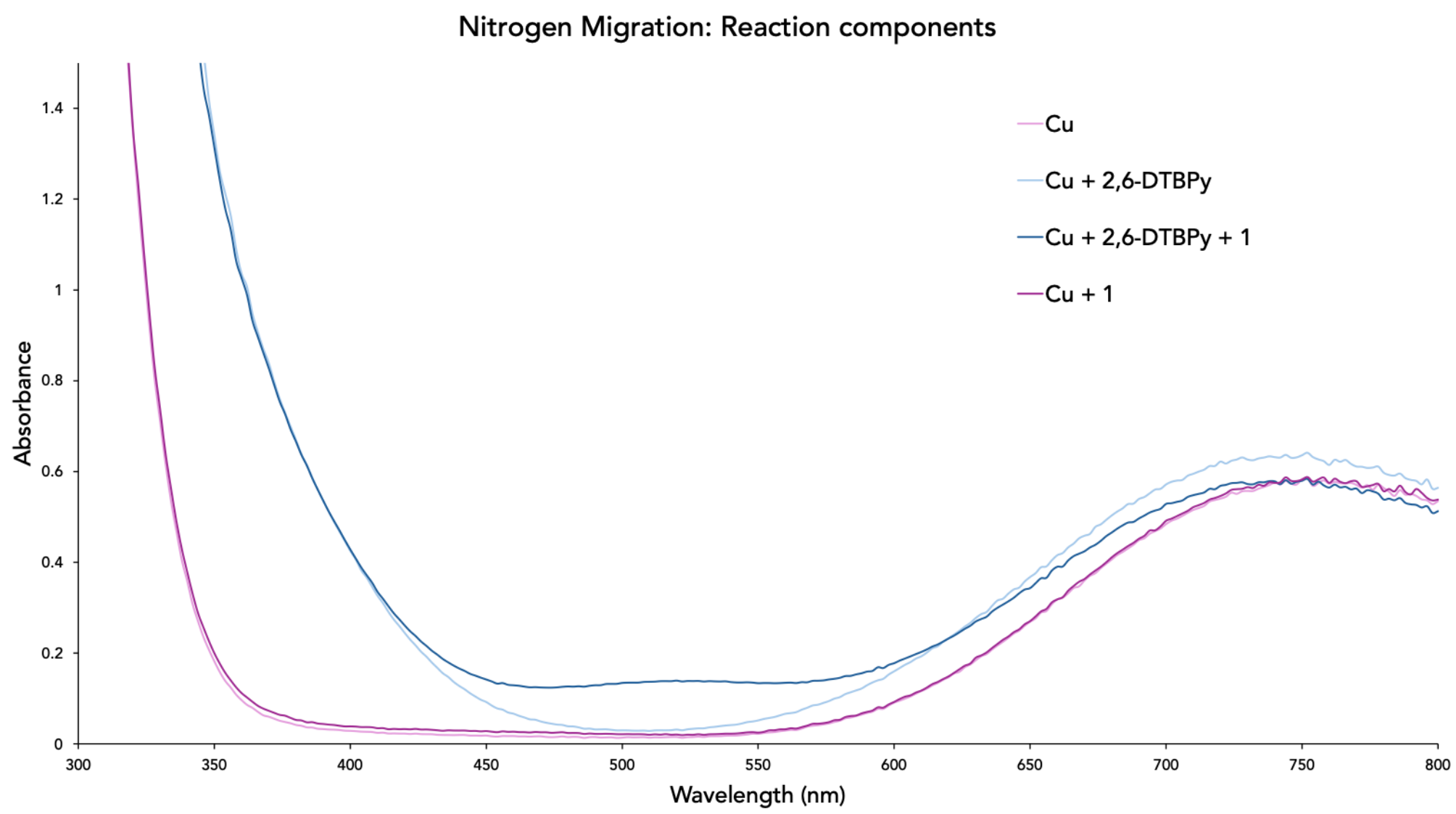

Figure S1. UV-vis of reaction components in MeCN

II. UV-vis titration study of Cu(II)/2,6-di-tertbutylpyridine with 1

Objective: To gain insight into whether **1** binds to the Cu/2,6-di-tertbutylpyridine complex.

Operational Notes and Procedure: Four stock solutions of $Cu(OTf)_2$, 2,6-di-tertbutylpyridine and **1** were prepared in anhydrous MeCN. MeCN was used rather than CH_2Cl_2 because of improved solubility. These solutions were prepared in a glove box and used to produce mixtures of Cu/2,6-di-tertbutylpyridine and **1** at the desired ratios across separate quartz cuvettes for UV-Vis analysis. Volumetric glassware and syringes were used to ensure accurate measurements.

Solution A:
$Cu(OTf)_2$ – (362 mg, 1.00 mmol)
MeCN – (10.0 mL, 100 mM)

Solution B:
2,6-di-tertbutylpyridine – (216 µL, 0.96 mmol)
MeCN – (10.0 mL, 96 mM)

Solution C:
1 – (63 mg, 0.20 mmol)
MeCN – (10.0 mL, 20 mM)

Solution D:
1 – (63 mg, 0.20 mmol)
MeCN – (2.0 mL, 100 mM)

Upon preparing the solution, aliquots were separately added into dried 2 mL volumetric flasks in the desired ratio (**Table S7**). Once the aliquots were added, the volumetric flasks were diluted to 2 mL.

Table S7. Solutions prepared for UV-Vis titration of Cu/2,6-di-tertbutylpyridine with **1**

Cuvette	[$Cu(OTf)_2$] mM	[2,6-di-tBu-py] mM	[1] mM	Ratio [$Cu(OTf)_2$] : [1]
1	25	30	0	1 : 0
2	25	30	2.5	1 : 0.1
3	25	30	5.0	1 : 0.2
4	25	30	25	1 : 1

UV-Vis measurements for each solution were collected 3 times after a blank subtraction. The results are summarized in **Fig. S2** below. As sulfonamide **1** is titrated into the $Cu(OTf)_2$/2,6-DTBPy solution in MeCN a new feature appears with a new feature centered at 520 nm.

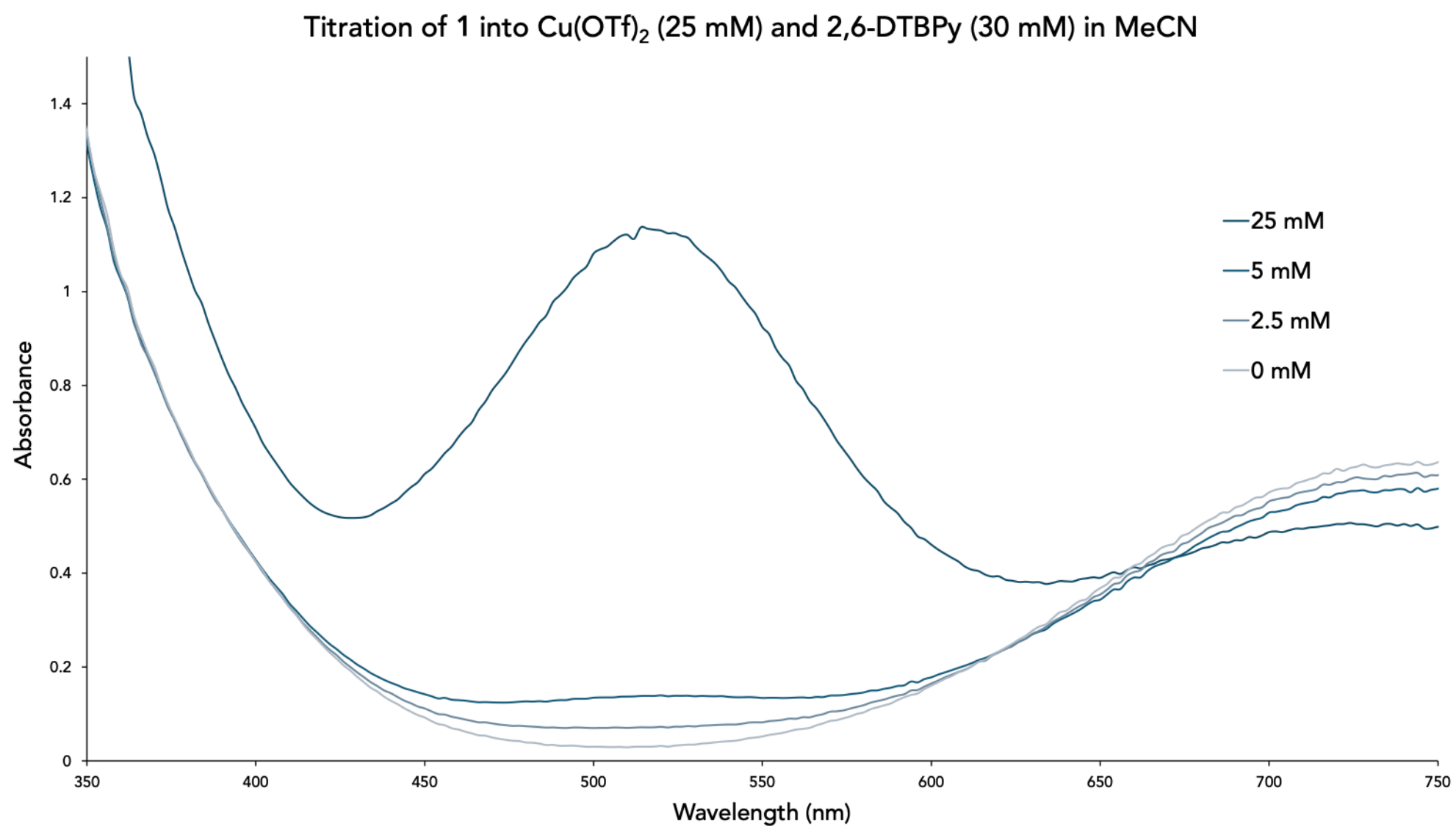

Figure S2. UV-vis titration of **1** into a $Cu(OTf)_2$/2,6-di-tertbutylpyridine solution

III. UV-vis titration study of Cu(II)/pyridine with 1

Objective: To gain insight into whether **1** binds to the Cu/pyridine complex.

Operational Notes and Procedure: Four stock solutions of $Cu(OTf)_2$, pyridine and **1** were prepared in anhydrous MeCN. MeCN was used rather than CH_2Cl_2 because of improved solubility. These solutions were prepared in a glove box and used to produce mixtures of Cu/pyridine and **1** at the desired ratios across separate quartz cuvettes for UV-Vis analysis. Volumetric glassware and syringes were used to ensure accurate measurements.

Solution A:
$Cu(OTf)_2$ – (362 mg, 1.00 mmol)
MeCN – (10.0 mL, 100 mM)

Solution B:
pyridine – (24.3 µL, 0.30 mmol)
MeCN – (2.0 mL, 150 mM)

Solution C:
1 – (63 mg, 0.20 mmol)
MeCN – (10.0 mL, 20 mM)

Solution D:
1 – (63 mg, 0.20 mmol)
MeCN – (2.0 mL, 100 mM)

Upon preparing the solution, aliquots were separately added into dried 2 mL volumetric flasks in the desired ratio (**Table S8**). Once the aliquots were added, the volumetric flasks were diluted to 2 mL.

Table S8. Solutions prepared for UV-Vis titration of Cu/2,6-di-tertbutylpyridine with **1**

Cuvette	$[Cu(OTf)_2]$ mM	[pyridine] mM	[1] mM	Ratio $[Cu(OTf)_2]$: [1]
1	25	30	0	1 : 0
2	25	30	2.5	1 : 0.1
3	25	30	5.0	1 : 0.2
4	25	30	25	1 : 1

UV-Vis measurements for each solution were collected 3 times after a blank subtraction. The results are summarized in **Fig. S3** below. As sulfonamide **1** is titrated into the $Cu(OTf)_2$/Pyridine solution in MeCN a very slight increase in absorbance occurs.

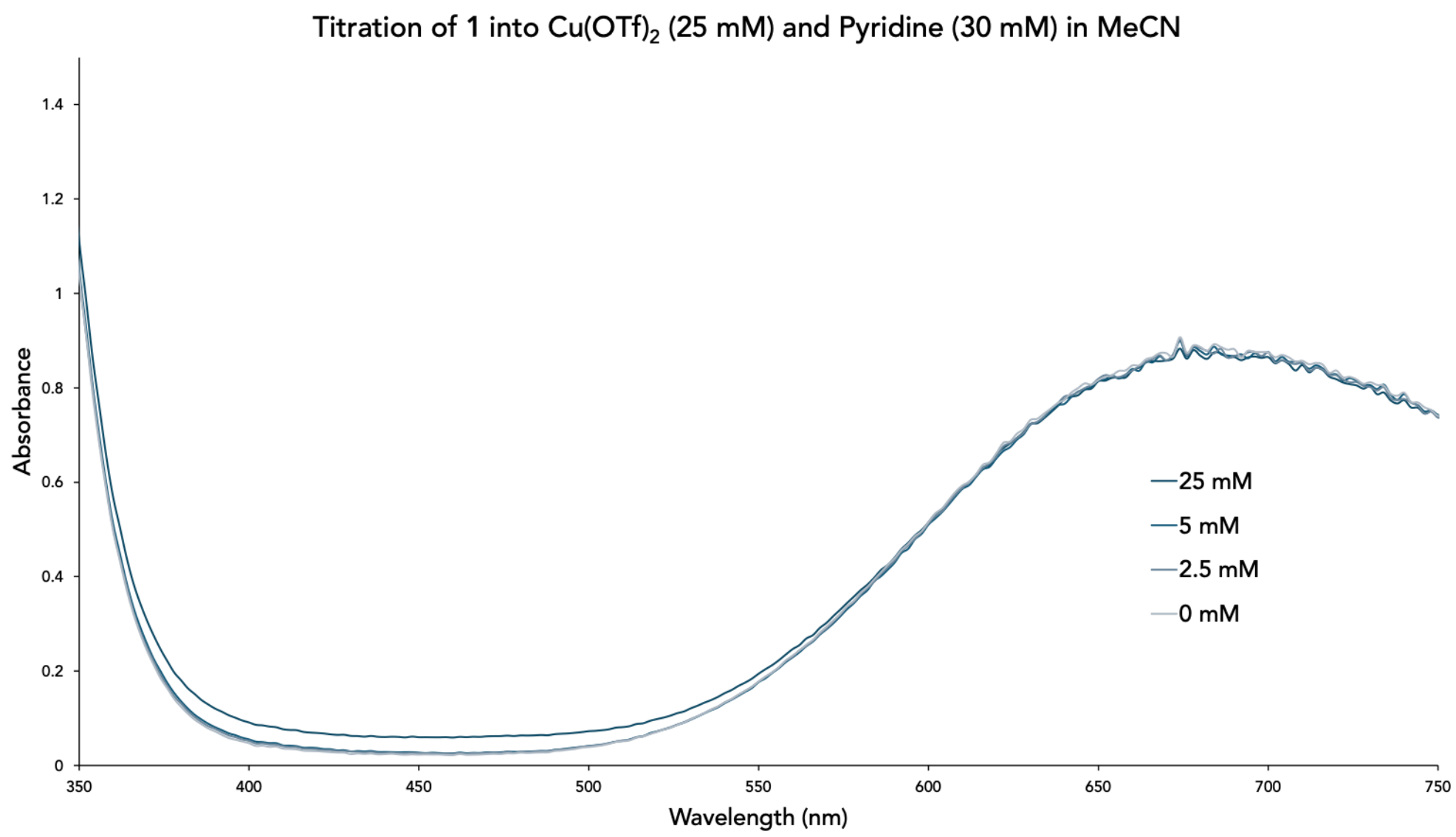

Figure S3. UV-vis titration of **1** into a $Cu(OTf)_2$/pyridine solution

I. UV-Vis titration study of Cu(II)/2,6-di-tertbutylpyridine with S45

Objective: To gain insight into whether **S45** binds to the Cu/2,6-di-tertbutylpyridine complex.

Operational Notes and Procedure: Three stock solutions of $Cu(OTf)_2$, 2,6-di-tertbutylpyridine and **S45** were prepared in anhydrous MeCN. MeCN was used rather than CH_2Cl_2 because of improved solubility. These solutions were prepared in a glove box and used to produce mixtures of Cu/2,6-di-tertbutylpyridine and **S45** at the desired ratios across separate quartz cuvettes for UV-Vis analysis. Volumetric glassware and syringes were used to ensure accurate measurements.

Solution A:
$Cu(OTf)_2$ – (362 mg, 1.00 mmol)
MeCN – (10.0 mL, 100 mM)

Solution B:
2,6-di-tertbutylpyridine – (216 µL, 0.96 mmol)
MeCN – (10.0 mL, 96 mM)

Solution C:
S45 – (82.4 mg, 0.25 mmol)
MeCN – (10.0 L, 25 mM)

Upon preparing the solution, aliquots were separately added into dried 2 mL volumetric flasks in the desired ratio (**Table S9**). Once the aliquots were added, the volumetric flasks were diluted to 2 mL.

Table S9. Solutions prepared for UV-Vis titration of Cu/2,6-di-tertbutylpyridine with **S45**

Cuvette	$[Cu(OTf)_2]$ mM	[2,6-di-tBu-py] mM	[?] mM	Ratio $[Cu(OTf)_2]$: [?]
1	25	30	0	1 : 0
2	25	30	2.5	1 : 0.1
3	25	30	5.0	1 : 0.2
4	25	30	10	1 : 0.4

UV-Vis measurements for each solution were collected 3 times after a blank subtraction. The results are summarized in **Fig. S4** below. As sulfonamide **S45** is titrated into the $Cu(OTf)_2$/2,6-DTBPy solution in MeCN a bathochromic shift was observed.

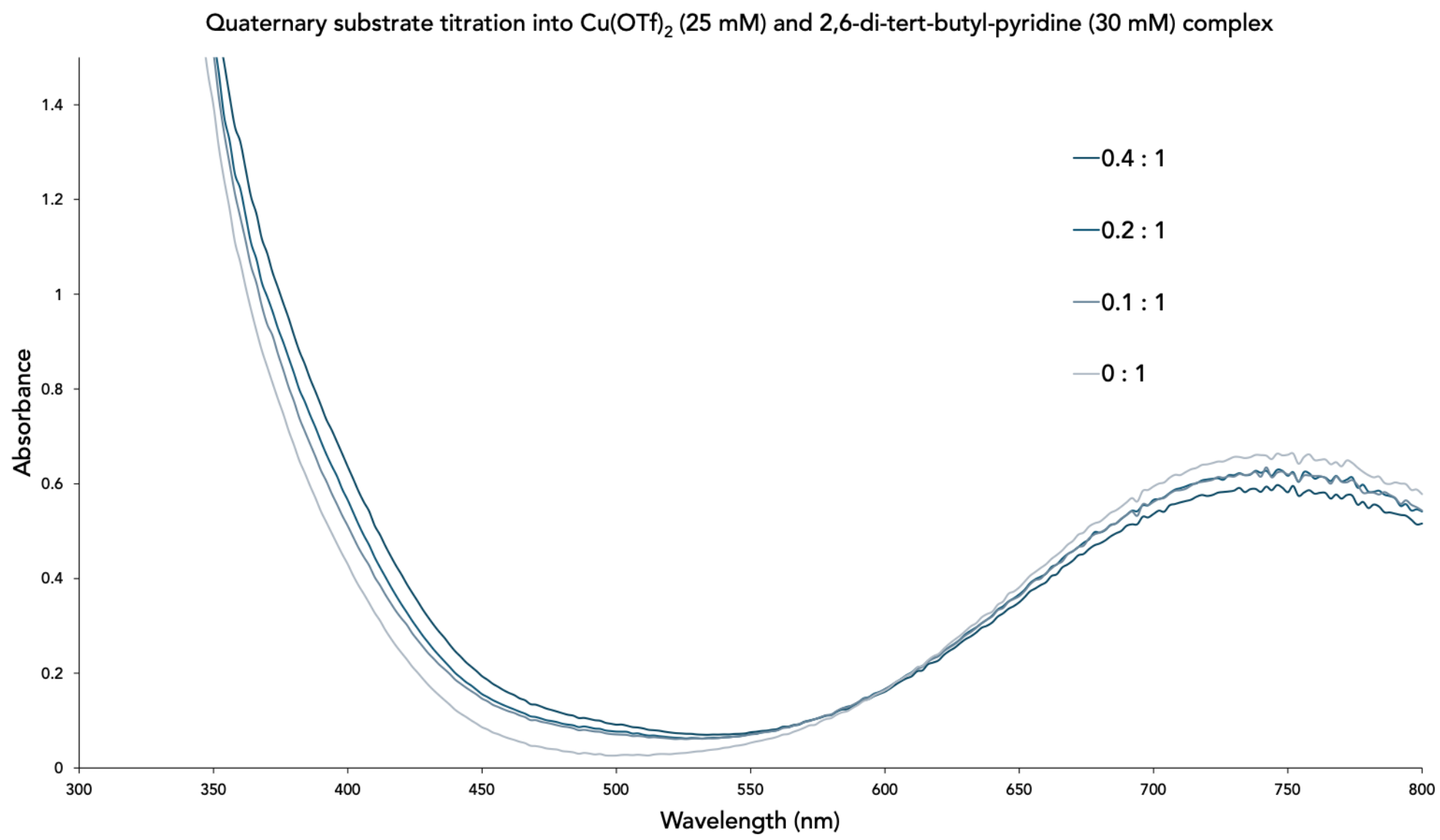

Figure S4. UV-vis titration of **S45** into a $Cu(OTf)_2$/2,6-di-tertbutylpyridine solution

K. Nitrogen protecting groups

benzyl ((1*R*,2*S*)-2-phenylcyclopentyl)carbamate (S56): A flame dried 8 mL vial was charged

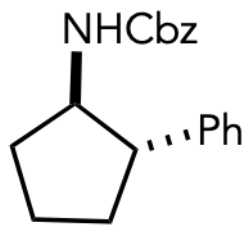

with amine hydrochloride **S1** (100 mg, 1 equiv, 0.51 mmol), DCM (1.0 mL, 0.5 M) and triethylamine (211 µL, 3 equiv, 1.52 mmol). The flask was cooled to 0 ºC and Cbz-Cl (95 mg, 1.1 equiv, 0.56 mmol) was added. The contents gradually warmed to room temperature and stirred overnight. The reaction was quenched with H_2O (10 mL), extracted with DCM (2 x 10 mL), dried over $MgSO_4$, filtered, and concentrated. The crude residue was purified over silica with 0-30% ethyl acetate and hexanes to afford a white solid (60 mg, 40%). **^{1}H NMR** (500 MHz, $CDCl_3$) δ 7.36 – 7.23 (m, 10H), 5.04 (s, 2H), 4.74 (br s, 1H), 4.08 (tp, 1H), 2.83 (d, *J* = 10.3 Hz, 1H), 2.33 (s, 1H), 2.17 (dtd, *J* = 12.4, 7.7, 4.7 Hz, 1H), 1.90 – 1.71 (m, 3H), 1.65 – 1.54 (m, 1H). **^{13}C NMR** (126 MHz, $CDCl_3$) δ 155.94, 142.43, 136.55, 128.55, 128.48, 128.03, 127.36, 126.55, 66.56, 58.87, 52.18, 32.83, 29.72, 22.14. **HRMS** (ESI) calculated for $[C_{19}H_{21}NO_2Na]^+$ $(M+Na)^+$ requires *m/z* 318.1465, found 318.1457. **Melting Point**: 76 – 78 °C.

Benzyl carbamate trial:

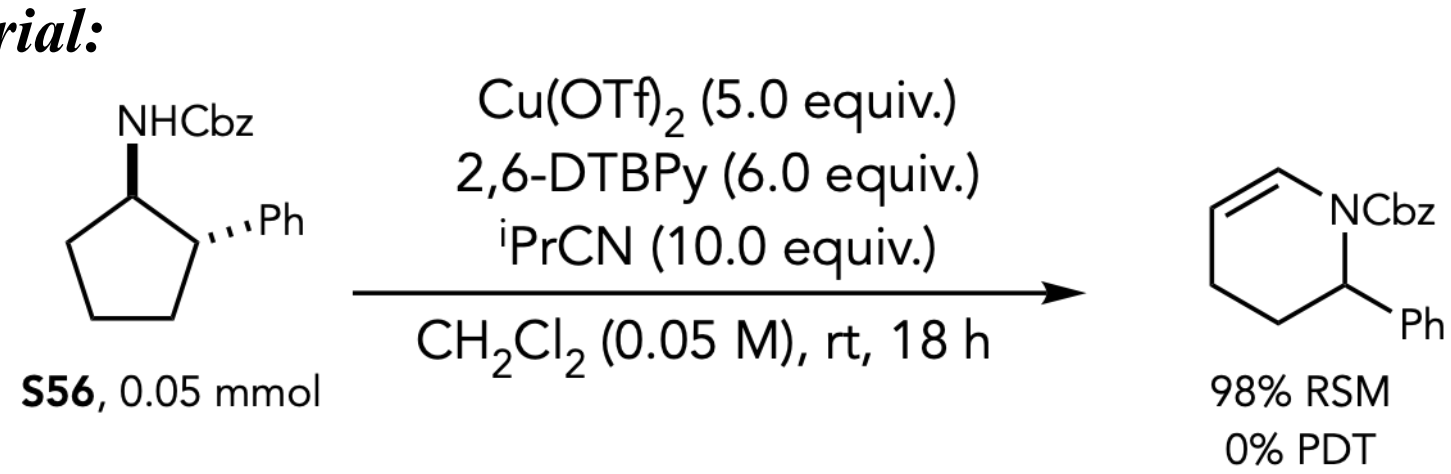

Tested according to **General Procedure F** with **(S56)** (14.8 mg, 1 equiv, 0.05 mmol). After reaction completion 1-methyl naphthalene (10 µL), was added to the reaction mixture for ^{1}H NMR analysis.

***N*-((1*R*,2*S*)-2-phenylcyclopentyl)benzamide (S57):** A flame dried 8 mL vial was charged with

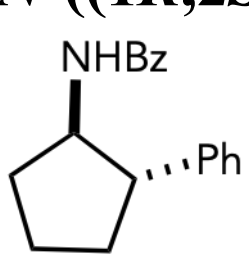

amine hydrochloride **S1** (100 mg, 1 equiv, 0.51 mmol), DCM (1.0 mL, 0.5 M) and triethylamine (211 µL, 3 equiv, 1.52 mmol). The flask was cooled to 0 ºC and the benzoyl chloride (1.1 equiv, 0.770 mmol) was added. The contents gradually warmed to room temperature and stirred overnight. The reaction was quenched with H_2O (10 mL), extracted with DCM (2 x 10 mL), dried over $MgSO_4$, filtered, and concentrated. The crude residue was purified over silica with 0-30% ethyl acetate and hexanes to afford a white solid (102 mg, 76%). **^{1}H NMR** (500 MHz, $CDCl_3$) δ 7.72 – 7.66 (m, 2H), 7.51 – 7.44 (m, 1H), 7.44 – 7.38 (m, 2H), 7.32 (d, *J* = 4.9 Hz, 4H), 7.27 – 7.19 (m, 1H), 6.07 (d, *J* = 7.4 Hz, 1H), 4.52 (dq, *J* = 9.7, 7.9 Hz, 1H), 3.01 (td, *J* = 9.9, 8.0 Hz, 1H), 2.48 (dtd, *J* = 13.4, 7.8, 5.6 Hz, 1H), 2.25 (dtd, *J* = 12.5, 7.6, 4.1 Hz, 1H), 1.98 – 1.77 (m, 3H), 1.71 – 1.60 (m, 1H). **^{13}C NMR** (126 MHz, $CDCl_3$) δ 167.36, 142.29, 134.81, 131.30, 128.64, 128.50, 127.28, 126.81, 126.61, 57.44, 52.19, 33.08, 32.78, 22.35. **HRMS** (ESI) calculated for $[C_{18}H_{19}NONa]^+$ $(M+Na)^+$ requires *m/z* 288.1359, found 288.1353. **Melting Point**: 162 – 164 °C.

Benzamide trial:

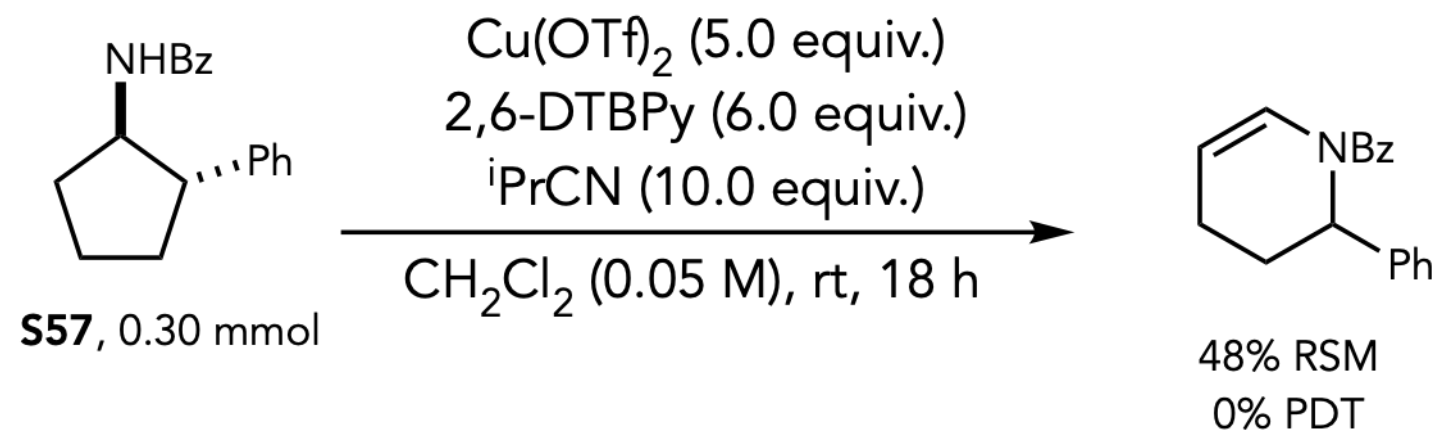

Tested according to **General Procedure F** with **(S57)** (79.6 mg, 1 equiv, 0.30 mmol). After reaction completion 1-methyl naphthalene (30 μL), was added to the reaction mixture for ^{1}H NMR analysis.

L. Other mechanistic experiments

N-methyl trial:

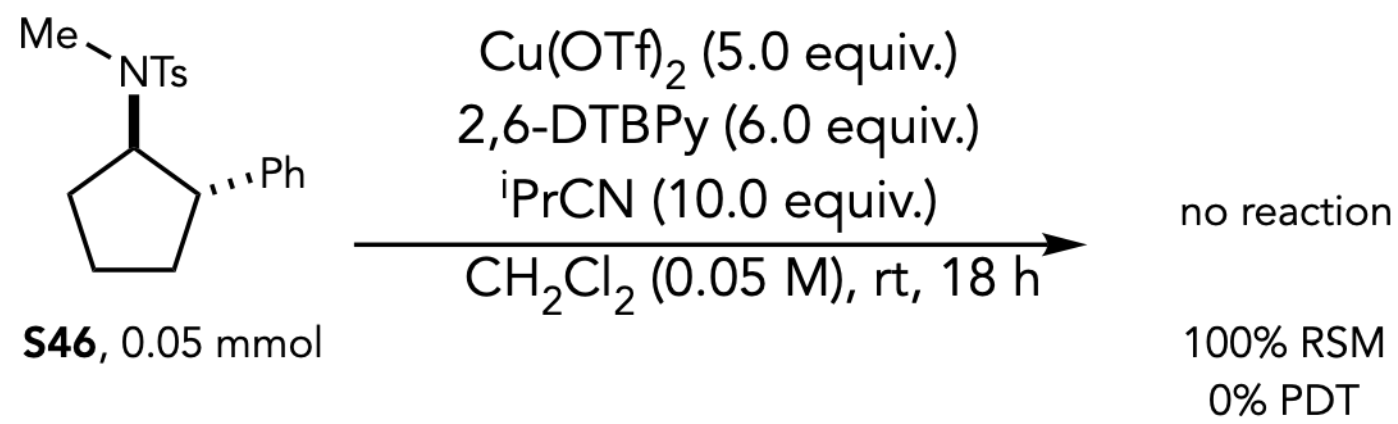

Tested according to **General Procedure F** with **(S46)** (16.5 mg, 1 equiv, 0.05 mmol). After reaction completion 1-methyl naphthalene (10 µL), was added to the reaction mixture for ^{1}H NMR analysis.

Oxygen Migration using 2,6-DTBPy:

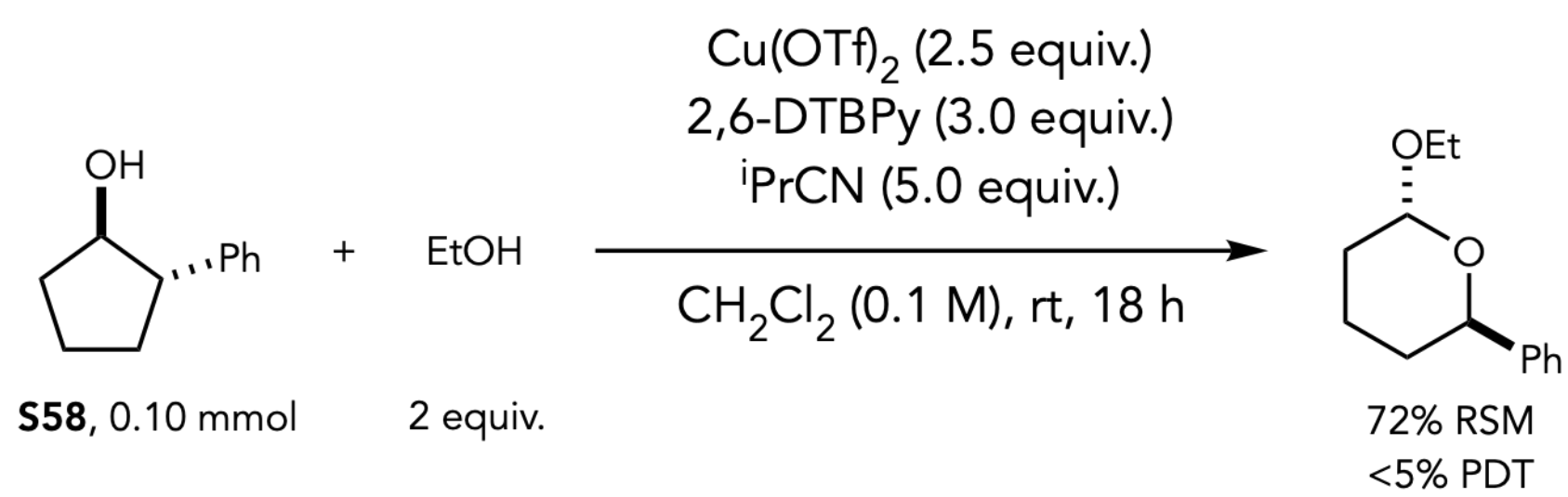

Adapted from a known literature procedure.[24] A 4 mL oven-dried vial equipped with a stir bar was brought into a nitrogen-filled glovebox. The vial was charged with **S58** (16.2 mg, 0.10 mmol, 1.0 equiv), $Cu(OTf)_2$ (90.4 mg, 0.25 mmol, 2.5 equiv), iPrCN (44.9 µL, 0.50 mmol, 5.0 equiv), and 2,6-di-*t*Bu-pyridine (67.4 µL, 0.30 mmol, 3.0 equiv). The reaction components were diluted with anhydrous CH_2Cl_2 (1 mL, 0.1 M), and ethanol (11.7 µL, 0.20 mmol, 2.0 equiv) was added. The vial was capped and brought out of the glovebox, then stirred at room temperature for 18 h. Upon completion, the reaction was diluted with EtOAc, and the organic layer was washed with NH_4Cl before being dried by Na_2SO_4 and concentrated under reduced pressure. Yields are determined by ^{1}H NMR analysis using 1-methylnapthalene (10 µL) as an internal standard.

S58 was prepared according to a known procedure.[24]

This observation seems consistent with the mechanism we propose for N-centered radical formation. Because the alcohol is more electronegative than the sulfonamide, its thermal oxidation is substantially more challenging and does not occur without the input of light energy.

***β-scission reversibility experiment*:**

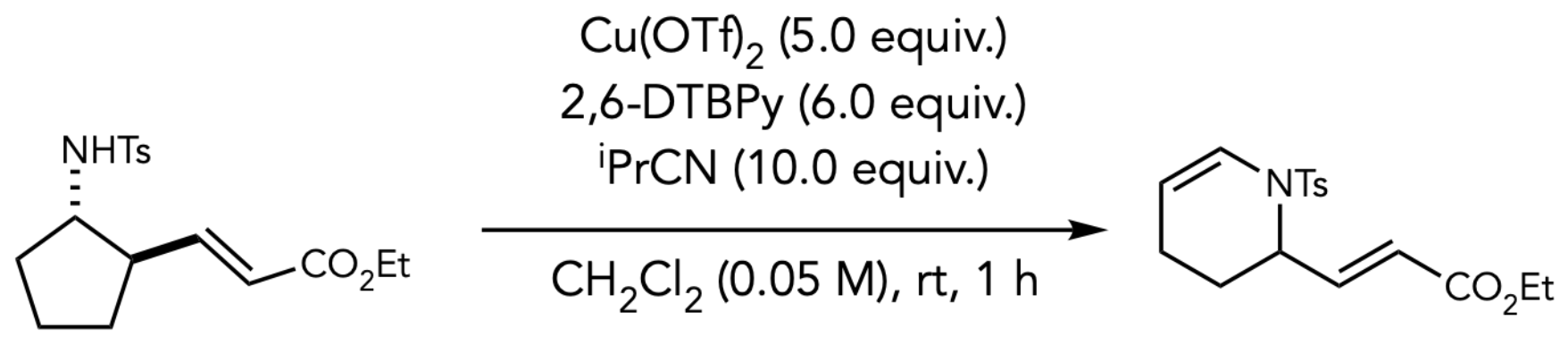

S40, 0.15 mmol
>99% ee

22, 48% (0% ee)
50% RSM (**S40**) (>99% ee)

Tested according to **General Procedure F** with **(S40)** (16.5 mg, 1 equiv, 0.05 mmol). After 1 h 1-methyl naphthalene (10 μL), was added to the reaction mixture for ^{1}H NMR analysis. NMR values matched racemic material.

HPLC Data:

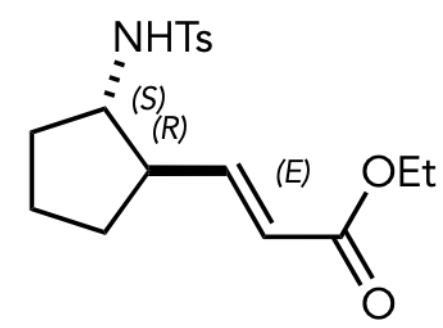

Method Conditions:

HPLC, Daicel CHIRALPAK OD-H

10.00 mL, 5% to 40% iPrOH/Hexanes, 30 minutes, 1 mL/min, 224 nm

Racemic Chromatogram (Compound **S38**)

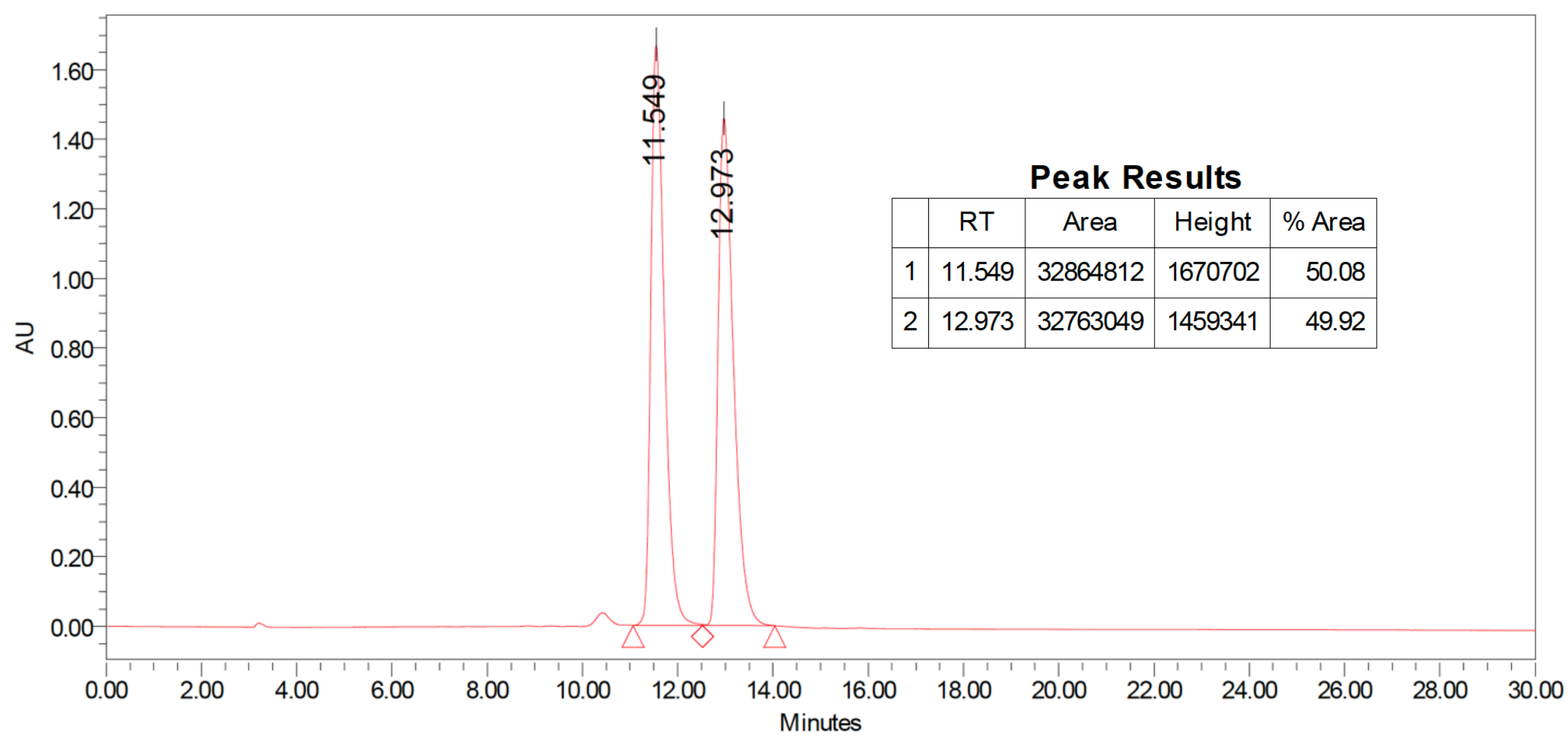

	RT	Area	Height	% Area
1	11.549	32864812	1670702	50.08
2	12.973	32763049	1459341	49.92

Scalemic Chromatogram (Compound **S40**)

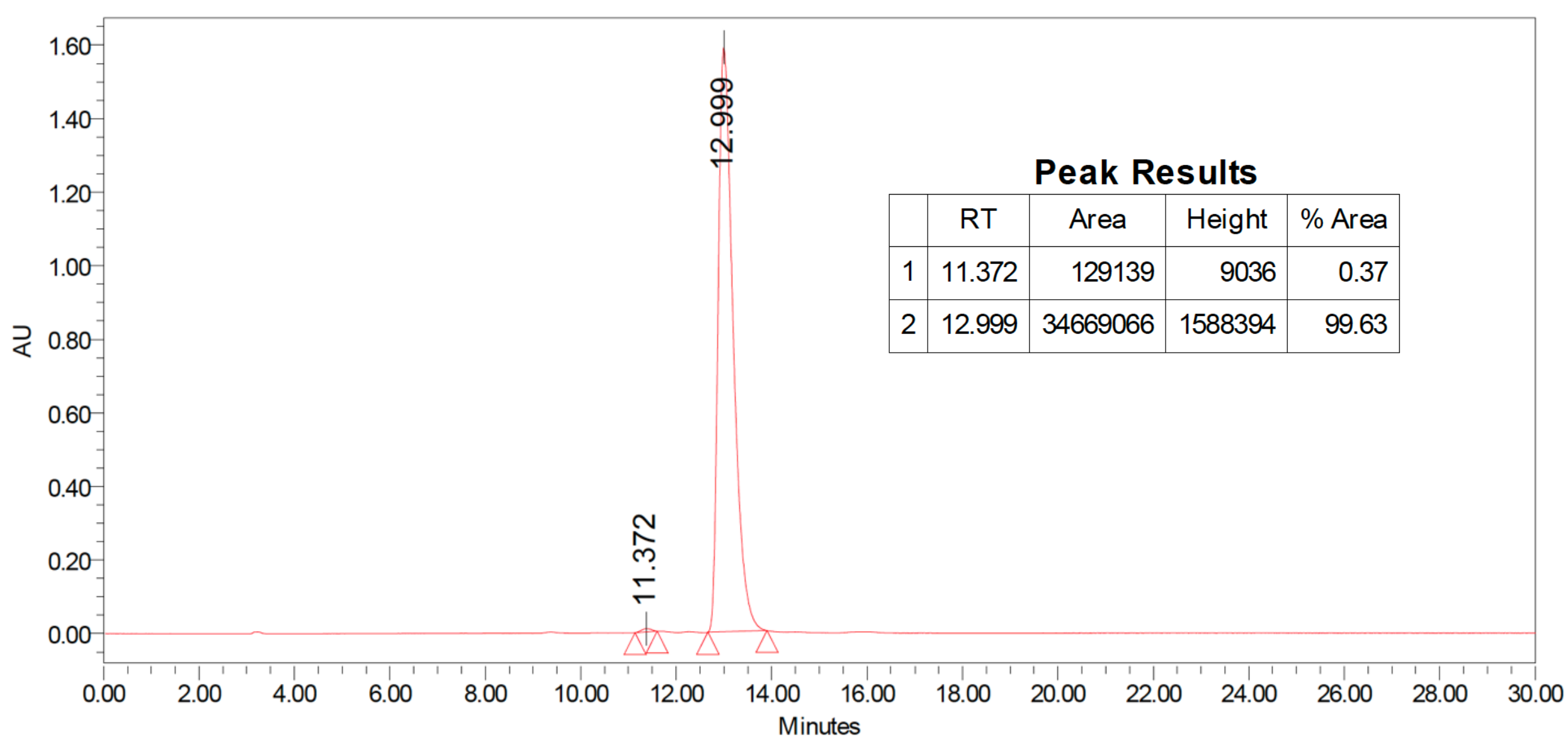

	RT	Area	Height	% Area
1	11.372	129139	9036	0.37
2	12.999	34669066	1588394	99.63

Chromatogram of Material Recovered from Reaction Conditions

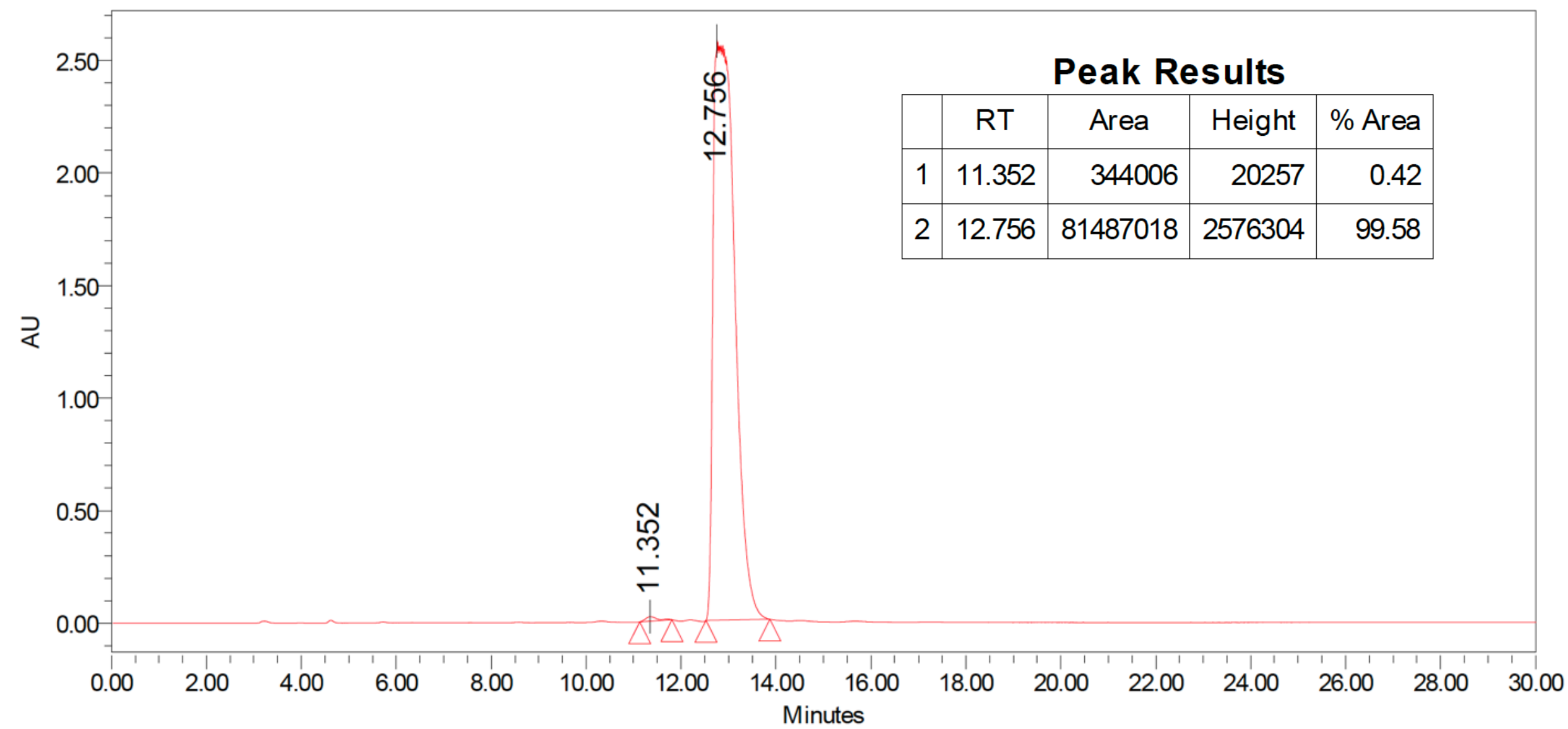

	RT	Area	Height	% Area
1	11.352	344006	20257	0.42
2	12.756	81487018	2576304	99.58

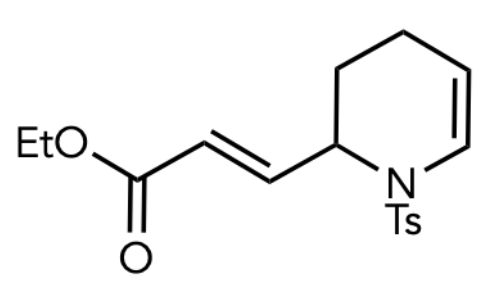

Method Conditions:

HPLC, Daicel CHIRALPAK OD-H

10.00 mL, 5% to 40% iPrOH/Hexanes, 30 minutes, 1 mL/min, 224 nm

Chromatogram of Product (**22**) Derived from Enantiopure Starting Material

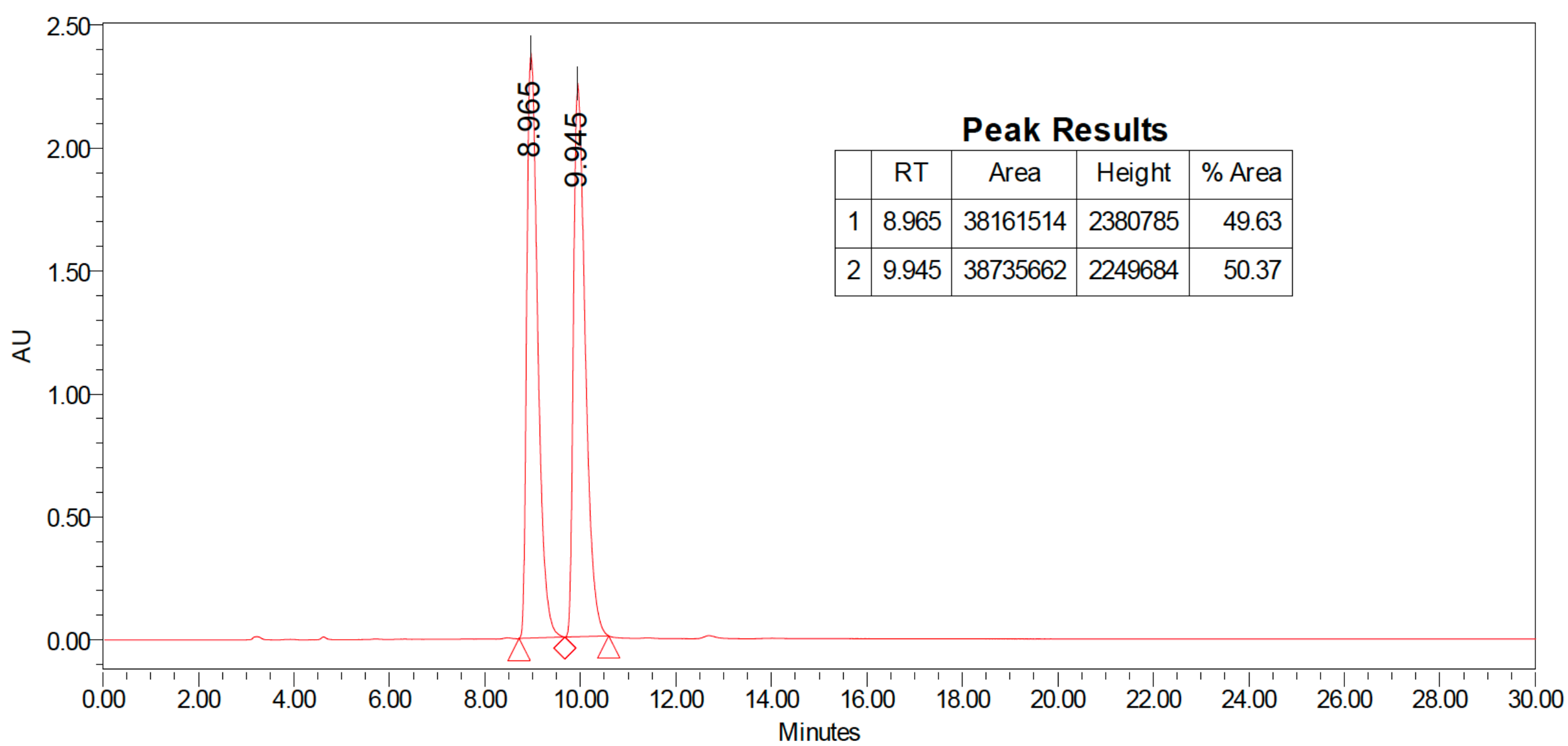

	RT	Area	Height	% Area
1	8.965	38161514	2380785	49.63
2	9.945	38735662	2249684	50.37

M. Unsuccessful Substrates

Below is a list of unsuccessful substrates tested for the nitrogen migration reaction following **General Procedure F**.

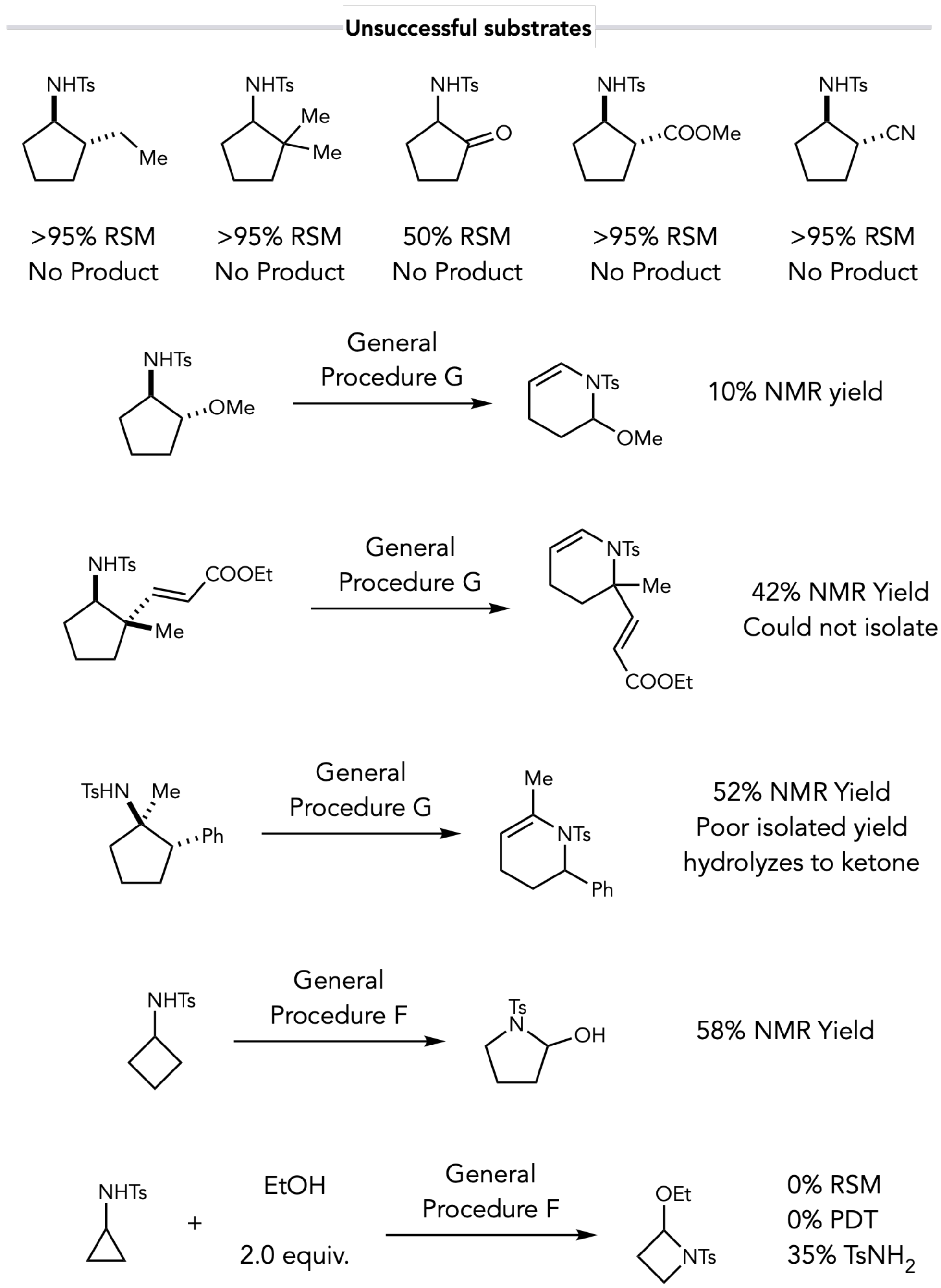

Figure S5. Unsuccessful substrates for the nitrogen migration.

N. Computational Analysis

Computational Methods

All structures were determined with Density Functional Theory (DFT) using B3LYP functional in conjunction with 6-31G(d) basis set. The nature of the stationary points was verified with harmonic vibrational frequency calculations. All calculations used the Polarizable Continuum Model (PCM) to model dichloromethane as the reaction solvent. All calculations were preformed using Gaussian 16.[25] The degree of spin contamination was verified to be 0.75 for the doublet radicals as explained by Cramer.[26]

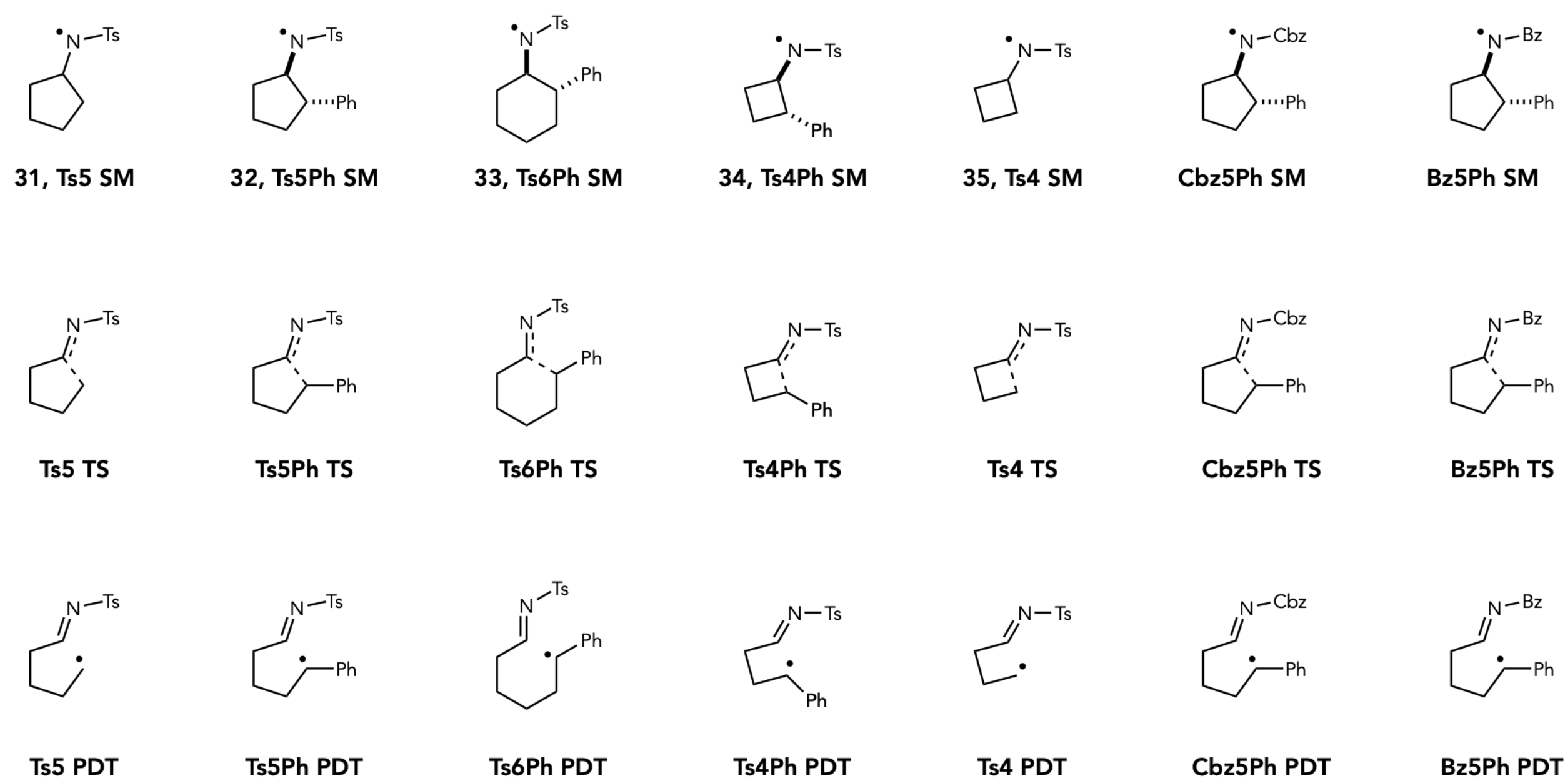

Figure S6. Structures for DFT Analysis

Below is a summary of the energies of the structures in kcal/mol.

Table S9. Calculated free energies for reaction intermediates and transition states.

Structure	Free energy	ΔG	E_A
Ts5H SM	–671558.797		
Ts5H TS	–671544.1801	+12.00	+14.62
Ts5H PDT	–671546.7987		
Ts5Ph SM	–816545.502		
Ts5Ph TS	–816539.415	–3.88	+6.09
Ts5Ph PDT	–816549.3811		
Ts6Ph SM	–841219.9744		
Ts6Ph TS	–841205.8292	+5.95	+14.16
Ts6Ph PDT	–841214.0256		
Ts4Ph SM	–791860.3164		
Ts4Ph TS	–791859.4051	–15.10	+0.91
Ts4Ph PDT	–791875.4201		
Ts4H SM	–646870.3379		
Ts4H TS	–646862.6187	–6.23	+7.72
Ts4H PDT	–646876.5634		
Cbz5Ph SM	–590632.2463		
Cbz5Ph TS	–590626.242	–3.40	+6.00
Cbz5Ph PDT	–590635.6474		
Bz5Ph SM	–518759.3779		
Bz5Ph TS	–518752.5892	–2.34	+6.79
Bz5Ph PDT	–518761.7226		

All energies in kcal/mol

Cartesian Coordinates

Below contains the cartesian coordinates (xyz format) for all intermediates and transition state structures involved in each reaction mechanism. All the reported structures are calculated at the PCM (CH_2Cl_2) B3LYP-6-31G(d).

Ts5H SM

C	0.00000000	0.00000000	0.00000000
C	-0.00019800	1.55108000	0.13348700
C	1.48263200	1.98496400	0.08158400
C	2.16969200	0.84685600	-0.71691800
C	1.48238700	-0.41703500	-0.13681700
H	1.63938700	-1.29095300	-0.77531700
H	1.92482600	-0.63046800	0.84285300
H	1.92161000	0.94448700	-1.78157000
N	3.59040500	0.82271000	-0.44931100
S	4.54807300	0.94443400	-1.83889600
O	4.32424300	2.27344000	-2.43977200
O	4.32365000	-0.25875900	-2.66297600
C	6.19788300	0.88569000	-1.17014500
C	6.83459100	2.07531600	-0.80949700
C	8.12148300	2.01665800	-0.27910500
C	8.78156700	0.79052100	-0.10652700
C	8.11540400	-0.38700000	-0.47929300
C	6.82937000	-0.35013800	-1.01266100
H	6.32420800	-1.26365700	-1.30718400
H	8.61117400	-1.34573700	-0.35305800
C	10.1878810	0.74106900	0.43902800
H	10.9220280	0.78887000	-0.37609000
H	10.3880840	1.58381400	1.10810700
H	10.3705480	-0.18826700	0.98738000
H	8.62193500	2.93922300	0.00261900
H	6.33448500	3.02807900	-0.94685400
H	1.92170500	2.01403500	1.08514000
H	1.64260500	2.96101500	-0.38527400
H	-0.47322400	1.88993700	1.06036100
H	-0.55625600	2.00524100	-0.69319900
H	-0.46480800	-0.49252100	0.85969300
H	-0.56364300	-0.30713200	-0.88718400

Ts5H TS

$\nu_{min} = -301.00$ cm^{-1}

C	0.00000000	0.00000000	0.00000000
C	-0.31622000	1.27586500	0.80631800
C	-1.82542400	1.52781800	0.69009100

C	-2.59796000	0.27567000	1.03315700
C	-0.93075200	-1.08880000	0.43418700
H	-0.77348900	-1.54682500	1.40853400
H	-1.42505300	-1.71952800	-0.29848400
H	-2.51177000	-0.09990400	2.05688100
N	-3.63872600	-0.05444700	0.29479500
S	-4.55364900	-1.36867500	0.85965600
O	-4.28000500	-1.67974500	2.27886300
O	-4.39501500	-2.44690100	-0.13554500
C	-6.21158600	-0.71794000	0.73391900
C	-6.83208500	-0.65779700	-0.51722800
C	-8.12144200	-0.14088100	-0.60824000
C	-8.80510100	0.31283700	0.53132900
C	-8.15776000	0.23838200	1.77298400
C	-6.86584400	-0.27358200	1.88397100
H	-6.37182500	-0.33382600	2.84759700
H	-8.67119400	0.58420700	2.66628800
C	-10.2144420	0.84222200	0.42288800
H	-10.3683990	1.37781800	-0.51945900
H	-10.9422300	0.02072700	0.45224000
H	-10.4522130	1.51998500	1.24842500
H	-8.60735600	-0.09093900	-1.57920500
H	-6.31606100	-1.01630500	-1.40151900
H	-2.09090000	1.83659100	-0.32672800
H	-2.14335100	2.32568500	1.37489900
H	0.25713200	2.13500900	0.44241500
H	-0.04295600	1.12328500	1.85849300
H	-0.12131000	0.19935600	-1.07070700
H	1.04592800	-0.30069800	0.16177800

Ts5H PDT

C	0.00000000	0.00000000	0.00000000
C	0.25789400	-1.06912900	1.00809300
H	0.22431600	-0.83989200	2.07038600
H	0.64372500	-2.04025300	0.71541200
C	-1.18814900	0.91809500	0.35028000
C	-2.53243100	0.15939000	0.34800700
C	-3.66258000	1.03126000	0.78664900
N	-4.66373700	1.26484100	0.02003800
S	-5.85173200	2.34352700	0.65196800
O	-5.96893200	3.41979100	-0.34456800
O	-5.58124500	2.69737300	2.05887200
C	-7.31506900	1.32626800	0.58698100
C	-7.82361200	0.77686600	1.76477600
H	-7.33435800	0.97275100	2.71257100
C	-8.96801700	-0.01644000	1.70009600

C	-9.60903400	-0.26835300	0.47866900
C	-10.8642160	-1.10425600	0.42075200
H	-10.9037300	-1.70233000	-0.49539200
H	-10.9354810	-1.77982700	1.27846600
H	-11.7568790	-0.46526600	0.42991000
C	-9.07155300	0.29632300	-0.68952800
C	-7.93193800	1.09410500	-0.64598400
H	-7.53069100	1.53537000	-1.55211400
H	-9.55445200	0.11076000	-1.64528700
H	-9.36962400	-0.44516000	2.61432300
H	-3.59768500	1.47254400	1.78849000
H	-2.74678600	-0.25046600	-0.64421200
H	-2.45965300	-0.68105700	1.05409500
H	-1.23602900	1.74437800	-0.36886200
H	-1.02079100	1.36673000	1.33868200
H	-0.15727200	-0.44423300	-0.99204200
H	0.89241500	0.64433700	-0.10417900

Ts5Ph SM

C	0.00000000	0.00000000	0.00000000
C	-1.13881200	0.98979700	0.26256000
C	-1.91650500	0.32023300	1.40333100
C	-1.88384300	-1.20313100	1.09307200
C	-0.67577300	-1.39399200	0.04352900
C	0.23624700	-2.55716300	0.35837300
C	0.36427100	-3.62081900	-0.54608500
C	1.22046900	-4.69166100	-0.28009800
C	1.96128900	-4.71899200	0.90237100
C	1.83902300	-3.66846500	1.81665000
C	0.98526200	-2.59977400	1.54632800
H	0.90190000	-1.79103200	2.26866100
H	2.40929100	-3.68188600	2.74169900
H	2.62665800	-5.55186400	1.11289700
H	1.30489300	-5.50460800	-0.99633000
H	-0.21692500	-3.61110700	-1.46440600
H	-1.13866100	-1.57204000	-0.93204600
H	-1.69140000	-1.80223000	1.98829600
N	-3.11275800	-1.58494100	0.44444600
S	-3.63089700	-3.18238800	0.71046100
O	-3.24698900	-3.67967000	2.04445300
O	-3.20859200	-3.94065900	-0.48443100
C	-5.39967700	-2.97251200	0.67100300
C	-6.11690800	-2.96857900	1.86869200
C	-7.50029600	-2.80205900	1.82665800
C	-8.17533000	-2.64020200	0.60855900

C -7.42593300 -2.64741100 -0.58007600
C -6.04510200 -2.81398800 -0.55991900
H -5.47594900 -2.82814500 -1.48328100
H -7.93296800 -2.52352300 -1.53333300
C -9.67580800 -2.48638500 0.56728500
H -10.0728000 -2.16767400 1.53552900
H -9.98078100 -1.75587200 -0.18935300
H -10.1568620 -3.43889000 0.30958300
H -8.06362400 -2.79926100 2.75584300
H -5.60017200 -3.09886700 2.81316000
H -2.94202300 0.68423000 1.51317700
H -1.40289600 0.48296300 2.35866300
H -1.77440100 1.08064300 -0.62733200
H -0.78642200 1.99344100 0.52272200
H 0.51096000 0.15707600 -0.95578100
H 0.75363200 0.07790200 0.79314200

Ts5Ph TS

ν_{min} = –387.45 cm^{-1}

C 0.00000000 0.00000000 0.00000000
C 1.15840400 0.88994600 -0.47138500
C 1.75557000 0.15878800 -1.67844400
C 2.01468500 -1.29113600 -1.28149700
C 0.48050100 -1.43766000 0.06124100
C -0.36873200 -2.56540200 -0.32677700
C -0.17076100 -3.82080000 0.29218400
C -0.96015700 -4.91699500 -0.04127500
C -1.95994700 -4.79296600 -1.01121300
C -2.15967400 -3.56269400 -1.64746200
C -1.37479700 -2.46328500 -1.31369300
H -1.54327100 -1.51971000 -1.82329700
H -2.93096900 -3.46232800 -2.40604900
H -2.57634500 -5.64832800 -1.27308400
H -0.79602000 -5.87016200 0.45341500
H 0.61597700 -3.92271100 1.03411800
H 1.09558000 -1.64976600 0.93304000
H 1.68439200 -2.07125900 -1.97092100
N 3.13989400 -1.49833700 -0.57071500
S 3.62645600 -3.09947900 -0.38706800
O 3.14133300 -3.97249200 -1.47738200
O 3.32274700 -3.49493900 1.00632200
C 5.39799800 -2.93491200 -0.54596200
C 6.02015200 -3.32245400 -1.73311200
C 7.40445700 -3.19537400 -1.84611800
C 8.17467400 -2.68291300 -0.79318200

C	7.52141500	-2.30604000	0.39212300
C	6.14159100	-2.42838900	0.52461000
H	5.64791700	-2.14801700	1.44907400
H	8.10364700	-1.91780700	1.22391300
C	9.66945200	-2.52298300	-0.92937300
H	10.0650180	-3.13346200	-1.74652600
H	9.93252200	-1.47807000	-1.13937300
H	10.1864090	-2.80593900	-0.00622800
H	7.89314500	-3.50361600	-2.76659600
H	5.42966600	-3.72676500	-2.54804400
H	2.68847800	0.61747100	-2.02258200
H	1.04549100	0.17204800	-2.51385300
H	1.91609200	0.97435800	0.31668100
H	0.82258600	1.90165700	-0.72191000
H	-0.35978200	0.29916100	0.99359800
H	-0.84344600	0.10312100	-0.69010900

Ts5Ph PDT

C	0.00000000	0.00000000	0.00000000
C	-1.17021600	0.93705100	-0.02435500
C	-2.20756400	0.99267900	0.93985000
C	-3.25345800	1.95466000	0.80252100
C	-4.28726000	2.03587000	1.72270000
C	-4.32895400	1.16539500	2.82241500
C	-3.31463400	0.21105100	2.98266100
C	-2.27475400	0.12046400	2.06709100
H	-1.50165100	-0.62762600	2.21368300
H	-3.34003000	-0.46667100	3.83223500
H	-5.13929100	1.23020300	3.54278400
H	-5.06922500	2.77910600	1.58976200
H	-3.22837500	2.63288400	-0.04728700
H	-1.21688700	1.65964700	-0.83654900
C	1.19908600	0.49152800	0.85250800
C	1.79957100	1.81101100	0.32673500
C	2.92470800	2.28718500	1.18661000
N	4.09578800	2.48689600	0.70425900
S	5.27033100	3.07972100	1.81979700
O	4.75265600	3.10479600	3.20138900
O	5.77786600	4.32588100	1.22477800
C	6.52773400	1.82070100	1.70026400
C	6.65497600	0.87662400	2.72058700
H	5.99845300	0.91487900	3.58291600
C	7.63943600	-0.10409200	2.61334800
C	8.49604200	-0.15506700	1.50395400
C	9.57835900	-1.20258300	1.41019500
H	9.77205800	-1.48483400	0.37051500

H	10.5214250	-0.82451500	1.82615000
H	9.31235000	-2.10429200	1.97016400
C	8.34166800	0.80716200	0.49306100
C	7.36674300	1.79681000	0.58249800
H	7.26241100	2.54255900	-0.19837600
H	8.99588400	0.78219800	-0.37434900
H	7.74423600	-0.84069400	3.40546400
H	2.71129600	2.44898700	2.24993600
H	2.14700900	1.70214700	-0.70582200
H	1.01930600	2.58613400	0.34147500
H	1.97291200	-0.28609700	0.85309900
H	0.87540800	0.62432200	1.89242900
H	0.35116600	-0.16328600	-1.02690500
H	-0.29904500	-0.98326200	0.38503200

Ts6Ph SM

C	0.00000000	0.00000000	0.00000000
C	-0.59575400	1.25491500	-0.64934400
C	0.11966800	2.52276100	-0.16444700
C	1.64582500	2.46054500	-0.40019000
C	2.23237900	1.18946700	0.28094400
C	1.51362600	-0.09325700	-0.23449400
H	1.93748600	-0.96416200	0.27493700
H	1.72703100	-0.20431300	-1.30491200
H	2.08831400	1.25601700	1.36704200
N	3.63670900	1.09044600	-0.07858900
S	4.66982000	0.56845300	1.16320200
O	4.32875500	1.19638900	2.45251200
O	4.68947200	-0.90595200	1.07092800
C	6.23815300	1.21010400	0.61588900
C	7.02739900	0.44794700	-0.24943500
C	8.24796600	0.96321700	-0.67707600
C	8.69226900	2.22612600	-0.25406100
C	7.87617200	2.96772800	0.61300800
C	6.65207300	2.47008100	1.05447500
H	6.02813200	3.04809900	1.72743600
H	8.20317600	3.94801200	0.94897600
C	10.0306200	2.76000200	-0.70243500
H	10.0606960	3.85288300	-0.65895900
H	10.8348670	2.38293300	-0.05721500
H	10.2625090	2.44744800	-1.72565000
H	8.86725100	0.37488400	-1.34893500
H	6.69394300	-0.53182000	-0.57387000
H	1.81758400	2.34064900	-1.47815000
C	2.34931100	3.73498400	0.04276100

C	2.35789900	4.14147600	1.38644000
C	2.99498900	5.32130500	1.77426100
C	3.63410100	6.12160300	0.82349900
C	3.63171800	5.73018500	-0.51636700
C	2.99542900	4.54711100	-0.89926900
H	3.00097700	4.24816900	-1.94496200
H	4.12590000	6.34324500	-1.26558400
H	4.12884300	7.04081800	1.12546000
H	2.99071500	5.61590800	2.82044900
H	1.86726800	3.53281000	2.14156800
H	-0.07792000	2.66933700	0.90642400
H	-0.27639800	3.40733800	-0.67768900
H	-0.50265200	1.17909800	-1.74212100
H	-1.66776800	1.32408800	-0.42840800
H	-0.20201900	0.01289800	1.08008300
H	-0.47706400	-0.90341900	-0.39941500

Ts6Ph TS

$\nu_{min} = -353.49\ cm^{-1}$

C	0.00000000	0.00000000	0.00000000
C	-0.23195200	1.40990000	-0.54840200
C	0.70101000	2.44212500	0.09207100
C	2.16633300	2.20916900	-0.20727700
C	2.48293200	0.29068200	0.63338400
C	1.44297700	-0.48444000	-0.16691200
H	1.51692700	-1.52301800	0.18459900
H	1.73673600	-0.50076100	-1.22377900
H	2.17999300	0.61802100	1.63373400
N	3.75002100	-0.07289700	0.41867600
S	4.79883400	0.05574900	1.72479000
O	4.40516500	1.09124000	2.70322700
O	4.97742000	-1.32030200	2.23958800
C	6.32601900	0.55419500	0.94239600
C	6.79989300	-0.13976700	-0.17719600
C	8.02800900	0.21656500	-0.72464300
C	8.80427600	1.24584300	-0.16449600
C	8.30795900	1.91720100	0.95981600
C	7.07342600	1.58042600	1.51704400
H	6.68787500	2.11294900	2.37916500
H	8.89165500	2.71800900	1.40615100
C	10.1436360	1.60524700	-0.76121300
H	10.5603600	2.50424300	-0.29789100
H	10.8662040	0.79114200	-0.62324800
H	10.0633220	1.78248200	-1.83994700
H	8.39424200	-0.31451400	-1.59970900

H	6.21202900	-0.93780500	-0.61774100
H	2.36444500	1.85028600	-1.21716000
C	3.16491900	3.15603900	0.27437000
C	2.96382600	3.95316500	1.42322700
C	3.90919700	4.89772800	1.80901600
C	5.07915500	5.07186500	1.06134800
C	5.30093500	4.28476000	-0.07302000
C	4.35995200	3.33428800	-0.45664600
H	4.53361200	2.72634700	-1.34065100
H	6.20799400	4.41270000	-0.65666200
H	5.81239800	5.81508300	1.36197500
H	3.73447600	5.50392100	2.69354400
H	2.06134900	3.83355200	2.01395300
H	0.52546100	2.47668000	1.17561600
H	0.44696400	3.44700800	-0.28073500
H	-0.08173500	1.41247700	-1.63721500
H	-1.27262400	1.70857000	-0.37497600
H	-0.26863100	-0.02740200	1.06562600
H	-0.66744900	-0.70887700	-0.50453600

Ts6Ph PDT

C	0.00000000	0.00000000	0.00000000
C	-0.25283500	-0.75192400	-1.33465600
H	-0.08375000	-1.82486000	-1.16875100
H	0.49580000	-0.42430300	-2.06687200
C	-1.64493800	-0.54311400	-1.95016200
C	-2.79353200	-1.08872100	-1.06735500
C	-4.10721500	-0.95375600	-1.77909900
N	-5.11065000	-0.17946800	-1.57331400
S	-5.05673000	0.91841400	-0.24517600
O	-3.91967700	1.84598300	-0.40125600
O	-5.19799200	0.15765900	1.00956300
C	-6.56510700	1.82888800	-0.53216100
C	-7.76855000	1.34169400	-0.01679100
C	-8.93876300	2.06245900	-0.24016500
C	-8.92601500	3.26177800	-0.96994700
C	-7.70063900	3.72966100	-1.46572400
C	-6.51828200	3.02231300	-1.25346700
H	-5.57250600	3.39606700	-1.63043300
H	-7.66867300	4.66308800	-2.02125700
C	-10.2063070	4.01855100	-1.22746800
H	-10.0114790	5.07259200	-1.44681600
H	-10.7410320	3.59621600	-2.08825200
H	-10.8823260	3.96355300	-0.36798700
H	-9.87696600	1.69121900	0.16382600
H	-7.78564200	0.42251100	0.55895900

H -4.23196900 -1.60907600 -2.64778100
H -2.81164400 -0.58987000 -0.09733600
H -2.62807600 -2.16065800 -0.89135700
H -1.67720600 -1.04105000 -2.92757300
H -1.81556400 0.52407100 -2.13154300
C -0.17232200 1.48660800 -0.09084300
H -1.18000200 1.87606600 0.04026700
C 0.84407600 2.42707700 -0.39053900
C 2.20430800 2.06629800 -0.62893600
C 3.16282300 3.02906600 -0.91575700
C 2.81897900 4.38678000 -0.97903500
C 1.48850300 4.76971300 -0.74965400
C 0.52304000 3.81697300 -0.46325300
H -0.50580900 4.12204700 -0.28682900
H 1.21106200 5.81980400 -0.79667500
H 3.57450600 5.13421800 -1.20364500
H 4.19102600 2.72367600 -1.09357800
H 2.49695000 1.02150000 -0.58861200
H -0.68324200 -0.39118100 0.76530300
H 1.01080100 -0.25638000 0.34016400

Ts4Ph SM
S 0.00000000 0.00000000 0.00000000
N -1.17845400 -0.48378300 1.10105500
C -1.63848500 0.39141100 2.10062200
C -2.36110300 -0.26336000 3.30459500
C -3.46839400 0.81208200 3.23384800
C -3.08083800 1.11437900 1.76504700
C -3.06220200 2.49730100 1.19647800
C -3.38326100 2.69674300 -0.15726900
C -3.35944300 3.97320300 -0.71966400
C -3.01213000 5.07667400 0.06395800
C -2.68764200 4.89313100 1.41119900
C -2.70922300 3.61597200 1.97056800
H -2.44953100 3.49256900 3.01828900
H -2.41813800 5.74642400 2.02761100
H -2.99586900 6.07227800 -0.37074500
H -3.61526500 4.10615200 -1.76721300
H -3.65115000 1.84133600 -0.77173400
H -3.60468000 0.43060300 1.09302300
H -4.49993100 0.48623000 3.39080100
H -3.26203100 1.65399200 3.90002000
H -2.72862800 -1.25496400 3.02729600
H -1.77392900 -0.33562900 4.22398200
H -0.93428700 1.17337100 2.40682700
O -0.72945800 0.44637900 -1.20201500

O	0.90985400	-1.15255200	-0.10455600
C	0.94610400	1.38281500	0.62903100
C	2.04678000	1.13591400	1.45569400
C	2.78456300	2.21298800	1.94019400
C	2.44422300	3.53411400	1.60979900
C	1.33381100	3.75115900	0.78062800
C	0.58052600	2.68752300	0.28742400
H	-0.27325000	2.86587500	-0.35690300
H	1.05283000	4.76684700	0.51566800
C	3.27105900	4.69220300	2.11235800
H	2.66710100	5.59871700	2.21876700
H	3.73008100	4.46595300	3.07979700
H	4.08395900	4.92149600	1.41098800
H	3.64178400	2.02480600	2.58115400
H	2.32642300	0.11765500	1.70399900

Ts4Ph TS

ν_{min} = –383.41 cm^{-1}

S	0.00000000	0.00000000	0.00000000
N	-1.07271700	-0.54934100	1.16694000
C	-1.53156000	0.28813400	2.13254900
C	-2.31760200	-0.32040300	3.30263700
C	-3.45425700	0.71713800	3.22594300
C	-3.17066100	1.05787500	1.76065700
C	-3.14884200	2.41524600	1.19977700
C	-3.33039900	2.58879300	-0.18927900
C	-3.30729500	3.85817200	-0.76055600
C	-3.09745500	4.98404600	0.04259100
C	-2.90947900	4.82940700	1.42020300
C	-2.93083200	3.56059900	1.99327800
H	-2.78152600	3.46055300	3.06387300
H	-2.74766800	5.70103600	2.04808800
H	-3.08126700	5.97534800	-0.40145600
H	-3.45547100	3.97152100	-1.83067400
H	-3.48311700	1.71494000	-0.81559200
H	-3.59162600	0.32245300	1.07555600
H	-4.46656800	0.34498700	3.41085900
H	-3.27124300	1.56371300	3.89223600
H	-2.65797100	-1.32329500	3.03139200
H	-1.75850600	-0.37079800	4.24221700
H	-0.93029200	1.16474500	2.39823700
O	-0.79647400	0.48454200	-1.14486900
O	0.93180700	-1.11986300	-0.21822000
C	0.95657000	1.37860200	0.63349900
C	2.08858000	1.12194900	1.41479700

C	2.83144300	2.19195300	1.90527200
C	2.46538500	3.51922300	1.62737800
C	1.32549800	3.74704100	0.84429800
C	0.56725900	2.68805600	0.34487500
H	-0.30883900	2.87428700	-0.26658400
H	1.02499900	4.76683100	0.61912900
C	3.29753200	4.66844700	2.14205500
H	2.74077900	5.60980800	2.11543000
H	3.62830600	4.49181200	3.17111400
H	4.19992600	4.80011300	1.53111900
H	3.71331500	1.99484700	2.50943900
H	2.38784100	0.10005700	1.62266900

Ts4Ph PDT

S	0.00000000	0.00000000	0.00000000
N	-0.04877900	1.60372100	-0.62928300
C	-1.17477000	2.22071900	-0.66773200
C	-2.55703100	1.78451900	-0.28098700
C	-3.39018400	1.42330200	-1.55695100
C	-4.81879000	1.11659100	-1.23224700
C	-5.32820100	-0.14986100	-0.85357400
C	-6.72082300	-0.29614500	-0.57591100
C	-7.26036000	-1.52065700	-0.21341600
C	-6.43938200	-2.65342800	-0.10629800
C	-5.06693900	-2.53581100	-0.36695000
C	-4.51384600	-1.31586200	-0.73346500
H	-3.44564200	-1.25003400	-0.91658200
H	-4.42421900	-3.40811800	-0.27925600
H	-6.86296000	-3.61218500	0.17885100
H	-8.32525000	-1.60131900	-0.01067800
H	-7.36305700	0.57773900	-0.65746300
H	-5.51690500	1.95132800	-1.24299100
H	-3.34939100	2.26970800	-2.25288300
H	-2.90023200	0.57645900	-2.04831500
H	-2.54252400	0.93483900	0.40374100
H	-3.04089500	2.62548100	0.22949800
H	-1.10744300	3.22704800	-1.09425800
O	-0.84701400	-0.88363700	-0.82521100
O	-0.21139600	0.04541600	1.45791500
C	1.70833700	-0.41002800	-0.31461000
C	2.67568100	-0.09144700	0.64053000
C	4.00544300	-0.42231300	0.38998800
C	4.38297400	-1.06545300	-0.79871900
C	3.38621500	-1.38092800	-1.73428100
C	2.05072900	-1.05802600	-1.50264800
H	1.28399900	-1.31577200	-2.22513100

H 3.65722600 -1.89161700 -2.65450100
C 5.83051100 -1.39403500 -1.07237000
H 5.92684200 -2.28711400 -1.69771700
H 6.32232400 -0.56872100 -1.60377500
H 6.38537600 -1.56047800 -0.14384400
H 4.76158800 -0.18264200 1.13282200
H 2.39067900 0.39409300 1.56769700

Ts4H SM

S 0.00000000 0.00000000 0.00000000
N 1.30788500 -0.97989500 -0.43302600
C 1.13591800 -2.38963800 -0.52947500
C 1.41862500 -3.20538100 0.81016800
C 2.17562000 -4.24163700 -0.05482500
C 2.29784000 -3.18134200 -1.17993400
H 2.14595200 -3.50394800 -2.21370900
H 3.22969900 -2.61325700 -1.10805600
H 3.10597000 -4.63725600 0.35999400
H 1.53172400 -5.07722500 -0.34466700
H 2.08500300 -2.63763700 1.46361300
H 0.52989500 -3.51718900 1.36383100
H 0.15676000 -2.68825100 -0.92330300
O -0.04577400 1.04300700 -1.03750900
O 0.25392600 0.38200900 1.40037900
C -1.54082000 -0.90677600 -0.06265300
C -2.02829700 -1.51805600 1.09547300
C -3.23301400 -2.21619600 1.03590200
C -3.95535900 -2.31858200 -0.16211300
C -3.44362300 -1.68948500 -1.30828100
C -2.24336500 -0.98486200 -1.26963800
H -1.86457300 -0.48754100 -2.15614000
H -3.99687900 -1.74490800 -2.24195300
C -5.24518100 -3.09922300 -0.22515900
H -5.06521300 -4.11624300 -0.59734900
H -5.70861200 -3.18767200 0.76188600
H -5.96347000 -2.62849100 -0.90412200
H -3.62151600 -2.68229900 1.93730300
H -1.48515500 -1.42881900 2.03009600

Ts4H TS

ν_{min} = –468.59 cm^{-1}

S 0.00000000 0.00000000 0.00000000
N -1.27170900 -0.92621200 -0.60796900
C -1.15281200 -2.24359700 -0.69810700

C -2.27927500 -3.06687500 -1.31618400
C -2.41222400 -4.10937300 -0.18356900
C -1.83971800 -3.24323800 0.91770300
H -1.00307000 -3.57651600 1.52709200
H -2.49798700 -2.51840300 1.38982700
H -3.43661900 -4.45074100 -0.00116100
H -1.78123300 -4.98349500 -0.36759300
H -3.17417000 -2.44473900 -1.40022500
H -2.02885400 -3.47070100 -2.30379000
H -0.16492000 -2.71652100 -0.69826900
O -0.30272600 0.26591500 1.41818300
O 0.12491900 1.14010600 -0.92292600
C 1.53212900 -0.92907900 -0.07212600
C 2.26615700 -0.95184500 -1.26217500
C 3.45531300 -1.67495700 -1.31175100
C 3.92816300 -2.37485900 -0.19042700
C 3.17080500 -2.33654500 0.98952700
C 1.97700200 -1.62011300 1.05777100
H 1.40264700 -1.58414400 1.97731200
H 3.52108500 -2.87113400 1.86857800
C 5.23594000 -3.12603400 -0.24633300
H 5.42626400 -3.52326800 -1.24836300
H 6.07463100 -2.46419900 0.00631800
H 5.25080300 -3.95773700 0.46474200
H 4.02899400 -1.69258400 -2.23467600
H 1.91656600 -0.40267000 -2.13009500

Ts4H PDT

C 0.00000000 0.00000000 0.00000000
C 1.04817300 -1.02991200 -0.25561400
H 0.85553500 -1.85813000 -0.93145400
H 2.06839900 -0.87413000 0.07943500
C -1.42618500 -0.60214500 0.03221100
C -2.46168100 0.43682300 0.32340700
N -3.40612800 0.68570400 -0.50605700
S -4.48789400 1.95137000 -0.04788200
O -4.46832400 2.90305100 -1.16937400
O -4.20995000 2.44332500 1.31512900
C -6.04683900 1.08559400 -0.04018000
C -6.67292300 0.79467900 -1.25572500
H -6.22313700 1.10510800 -2.19274400
C -7.88881100 0.11724100 -1.24177900
C -8.48937700 -0.27432400 -0.03431300
C -9.79432200 -1.03230100 -0.03536000
H -9.61801300 -2.10816000 -0.16497200
H -10.3351720 -0.89985200 0.90649300

H	-10.4431470	-0.70916400	-0.85568700
C	-7.84054900	0.03931800	1.16882500
C	-6.62115300	0.71440400	1.17667900
H	-6.12732000	0.96144300	2.11006500
H	-8.29706200	-0.24405100	2.11339000
H	-8.38360900	-0.10628300	-2.18317900
H	-2.37631100	0.98160500	1.27133100
H	-1.65836500	-1.09671000	-0.91597200
H	-1.47225900	-1.35585100	0.83071200
H	0.02628300	0.78040600	-0.78207800
H	0.20463800	0.52329500	0.94370300

Cbz5Ph SM

C	0.00000000	0.00000000	0.00000000
C	-0.16741200	1.25623300	-0.90264700
C	0.03462200	0.76863300	-2.35440300
C	0.84914600	-0.53766800	-2.24820200
C	0.24529700	-1.20909100	-0.93548900
H	0.99778000	-1.88318900	-0.51837000
C	-0.96921200	-2.04318000	-1.30203300
C	-0.77711300	-3.37314900	-1.71354800
C	-1.85567700	-4.17836100	-2.07678900
C	-3.15608900	-3.66700700	-2.03812600
C	-3.36282900	-2.34699100	-1.63488000
C	-2.27936500	-1.54227700	-1.27271000
H	-2.46544900	-0.52047400	-0.95483900
H	-4.36962000	-1.93919500	-1.59874500
H	-3.99938300	-4.29288300	-2.31710900
H	-1.68221600	-5.20580500	-2.38577500
H	0.23332100	-3.77383300	-1.75003300
H	0.72148500	-1.20214700	-3.10978500
N	2.22888800	-0.23170200	-1.99044100
C	3.15471600	-1.10996200	-2.52864400
O	4.28336900	-0.45761500	-2.85682900
C	5.37598200	-1.26169000	-3.39693600
C	6.29739800	-1.74675400	-2.30575700
C	6.11701200	-3.00811700	-1.72176300
C	6.96924900	-3.44533600	-0.70643000
C	8.00914600	-2.62470200	-0.26243700
C	8.19545400	-1.36535800	-0.83833500
C	7.34390900	-0.93084700	-1.85492900
H	7.49373200	0.04738100	-2.30584500
H	9.00514500	-0.72477900	-0.49962200
H	8.67358600	-2.96615000	0.52672700
H	6.82260800	-4.42666600	-0.26339900

H 5.30248500 -3.64027900 -2.06247000
H 4.95132100 -2.09099800 -3.96490400
H 5.88977500 -0.57761400 -4.07448800
O 2.98789200 -2.32117000 -2.62450200
H 0.52852800 1.50570200 -2.99444200
H -0.92472500 0.51860300 -2.82107800
H 0.58189500 2.00990400 -0.64297900
H -1.14796700 1.72838600 -0.77983500
H 0.86772700 0.12242000 0.65419100
H -0.86351500 -0.17052800 0.64975000

Cbz5Ph TS

ν_{min} = –365.75 cm^{-1}

C 0.00000000 0.00000000 0.00000000
C -0.05816200 -1.49474800 -0.34339000
C -0.24045300 -1.55063000 -1.86468400
C -1.39665700 -0.64111900 -2.25377900
C -1.14919200 0.70391300 -0.69146100
H -2.11148900 0.51922700 -0.21853700
C -1.03257900 2.04305700 -1.26154600
C -2.17603200 2.87308500 -1.31878700
C -2.10614200 4.15732100 -1.84857600
C -0.89480800 4.64504700 -2.34943400
C 0.24506400 3.83364000 -2.31643100
C 0.17956500 2.55061100 -1.78269100
H 1.07393300 1.93536800 -1.77282300
H 1.18763700 4.20522400 -2.70885800
H -0.83888400 5.64740100 -2.76452400
H -2.99596700 4.78019100 -1.87267100
H -3.12140700 2.49715900 -0.93788000
H -1.23435800 0.07436900 -3.06396200
N -2.63182500 -1.13100800 -2.07336500
C -3.64620400 -0.43070500 -2.71387200
O -4.83105400 -1.03698900 -2.45122900
C -6.01656600 -0.42229900 -3.01873100
C -6.59837200 0.62333900 -2.09638000
C -6.29583600 1.98145100 -2.26655100
C -6.82702400 2.93950900 -1.40030500
C -7.66440200 2.54957800 -0.35211300
C -7.97075300 1.19791500 -0.17449700
C -7.44114900 0.24260900 -1.04333600
H -7.68563300 -0.80827500 -0.90611600
H -8.62522600 0.88894200 0.63613800
H -8.07945600 3.29532500 0.32063400
H -6.59009200 3.99015700 -1.54597700
H -5.63731200 2.28058000 -3.07632200

H -5.76533100 0.00093600 -3.99282000
H -6.70979500 -1.25608700 -3.14979600
O -3.54476300 0.57015500 -3.41623800
H -0.44469900 -2.56845500 -2.21467000
H 0.67216300 -1.20091100 -2.36219900
H -0.92311000 -1.95977200 0.14443300
H 0.83963100 -2.02682900 -0.01107100
H -0.07710400 0.16677900 1.08324600
H 0.96319600 0.41157400 -0.31883500

Cbz5Ph PDT

C 0.00000000 0.00000000 0.00000000
C -0.27995500 -1.23476200 -0.79986900
C -0.99580300 -2.38488800 -0.38890800
C -1.56915100 -2.54098600 0.90910300
C -2.25985500 -3.69463600 1.25448400
C -2.41488900 -4.74159100 0.33492000
C -1.86369900 -4.61415500 -0.94922600
C -1.17237500 -3.46688900 -1.30562200
H -0.74863700 -3.37689500 -2.30320300
H -1.97865400 -5.41894800 -1.67090100
H -2.95689100 -5.64075900 0.61329800
H -2.68568400 -3.78385900 2.25079700
H -1.46879600 -1.74542000 1.64066400
H 0.08907500 -1.22918500 -1.82553500
C 1.45859800 0.48957500 -0.12320400
C 2.47288500 -0.50394900 0.48325900
C 3.86910800 0.02219000 0.41677800
N 4.80442000 -0.59103400 -0.20589300
C 6.05886400 0.05694000 -0.25879300
O 7.12015700 -0.76350600 -0.16903400
C 6.92798700 -2.19343000 0.05838700
C 8.28631100 -2.84107000 0.04128200
C 8.87432400 -3.21920900 -1.17302900
C 10.1417890 -3.80198500 -1.19562700
C 10.8345670 -4.01448700 -0.00059300
C 10.2557410 -3.64188500 1.21474800
C 8.98821400 -3.05701200 1.23348100
H 8.53850300 -2.76799100 2.18046100
H 10.7891660 -3.80776800 2.14668100
H 11.8200740 -4.47193500 -0.01677900
H 10.5867410 -4.09367600 -2.14299900
H 8.33478200 -3.05721600 -2.10316900
H 6.28042800 -2.58749600 -0.72760100
H 6.43198200 -2.33430100 1.02239200
O 6.22780800 1.24843900 -0.43524100

H	4.06514500	0.97871800	0.91998100
H	2.21717100	-0.66330500	1.54153900
H	2.42285400	-1.47204300	-0.02423800
H	1.55084400	1.46156600	0.37726700
H	1.70087100	0.65472000	-1.18089400
H	-0.24214300	-0.14378900	1.05922800
H	-0.65113700	0.81796300	-0.35135600

Bz5Ph SM

C	0.00000000	0.00000000	0.00000000
C	0.36225000	0.85097000	-1.25096000
C	0.70871500	-0.15853300	-2.36839000
C	1.07735700	-1.47494600	-1.64878900
C	0.06210700	-1.48711700	-0.42431500
H	0.48956200	-2.10480500	0.36979200
C	-1.24574800	-2.12789800	-0.85372700
C	-1.36115100	-3.52770200	-0.80144100
C	-2.53274100	-4.16995300	-1.19937700
C	-3.61921500	-3.42208700	-1.66248000
C	-3.51878400	-2.03144200	-1.72380800
C	-2.34230300	-1.39102600	-1.32564300
H	-2.29010900	-0.30731500	-1.37558900
H	-4.35761800	-1.43939900	-2.08033900
H	-4.53527000	-3.91915400	-1.96986500
H	-2.59977900	-5.25331900	-1.14423200
H	-0.51632200	-4.11382200	-0.44722600
H	0.93919800	-2.36529800	-2.27223700
N	2.40995700	-1.38704900	-1.12638800
C	3.16632300	-2.54517500	-1.10375000
O	2.63353700	-3.65350500	-0.95164500
C	4.64266800	-2.36864000	-1.18331600
C	5.45330700	-3.51057600	-1.27590900
C	6.83607300	-3.38144600	-1.37299500
C	7.42069100	-2.11015200	-1.37873600
C	6.61879100	-0.96926500	-1.28677400
C	5.23368300	-1.09526900	-1.18854500
H	4.60447900	-0.21549900	-1.10599400
H	7.07320900	0.01737700	-1.28705500
H	8.50001400	-2.01011300	-1.45404500
H	7.45990800	-4.26778700	-1.44541900
H	4.98239200	-4.48830700	-1.27257500
H	1.51123500	0.18380000	-3.02850300
H	-0.16592200	-0.36428300	-2.99551000
H	1.21899700	1.49617900	-1.03436000
H	-0.45865000	1.50801200	-1.55768200

H 0.73364000 0.17076300 0.79284200
H -0.97915900 0.25561400 0.41585200

Bz5Ph TS

ν_{min} = –354.98 cm^{-1}

C 0.00000000 0.00000000 0.00000000
C 0.98061200 0.99457700 -0.63908000
C 1.30164100 0.41425000 -2.02073900
C 1.75020900 -1.02804500 -1.84749500
C 0.52112900 -1.40927200 -0.19216400
H 1.33004900 -1.67302500 0.48506600
C -0.34536500 -2.53255400 -0.52863100
C 0.02195600 -3.83665700 -0.12080700
C -0.78181700 -4.93365800 -0.41012900
C -1.96925300 -4.76360000 -1.12991200
C -2.34262700 -3.48441700 -1.55811000
C -1.54425200 -2.38392600 -1.26433900
H -1.85082400 -1.40184700 -1.61035400
H -3.26100700 -3.34710400 -2.12219900
H -2.59761300 -5.61995900 -1.35789000
H -0.48397500 -5.92381900 -0.07672400
H 0.94865700 -3.97358200 0.42784000
H 1.31849700 -1.78655300 -2.50584300
N 2.99389000 -1.21789200 -1.38910700
C 3.50040500 -2.51162100 -1.46973600
O 2.82548100 -3.51167500 -1.74686100
C 4.96246000 -2.63068900 -1.16837300
C 5.57407300 -3.88977300 -1.27659100
C 6.93293400 -4.04008000 -1.01116400
C 7.69840000 -2.93164000 -0.63346800
C 7.09782200 -1.67470900 -0.52301900
C 5.73682300 -1.52312600 -0.78850800
H 5.26003000 -0.55355700 -0.70263100
H 7.69016600 -0.81264800 -0.22835100
H 8.75874900 -3.04837400 -0.42576100
H 7.39772900 -5.01846600 -1.09793000
H 4.96595000 -4.73891600 -1.57113200
H 2.09006400 0.98126300 -2.52854500
H 0.40815200 0.44178900 -2.65604900
H 1.90132600 1.04984100 -0.04669100
H 0.55812700 2.00346400 -0.69715900
H -0.12068900 0.19314300 1.07475200
H -0.98764500 0.11793400 -0.45714400

Bz5Ph PDT

C 0.00000000 0.00000000 0.00000000

C	-0.50607800	1.32764300	-0.47481800
C	-1.70166900	1.58342600	-1.18882800
C	-2.03495800	2.92597400	-1.54633600
C	-3.19384400	3.22346700	-2.24626900
C	-4.07866700	2.20157000	-2.62290400
C	-3.77873600	0.87492800	-2.28291300
C	-2.62073100	0.56481600	-1.58259500
H	-2.41665100	-0.47057900	-1.32852800
H	-4.45989600	0.07720700	-2.56854000
H	-4.98669300	2.43575900	-3.17102700
H	-3.41681400	4.25582400	-2.50367500
H	-1.35503700	3.72460500	-1.25864900
H	0.09731600	2.19389500	-0.20348700
C	1.52964600	-0.14887900	-0.12324600
C	2.01566800	-0.14392900	-1.58836100
C	3.49095800	-0.36432200	-1.68832100
N	4.28944100	0.51253700	-2.16497600
C	5.65586400	0.16660400	-2.28363100
O	6.02038300	-0.91690400	-2.72746900
C	6.61482200	1.23745700	-1.89129300
C	6.18141500	2.50845700	-1.48294700
C	7.11176300	3.47590400	-1.10432300
C	8.47767300	3.18269900	-1.13279500
C	8.91547900	1.91818500	-1.54145700
C	7.98898900	0.95048900	-1.91924000
H	8.30983300	-0.03598200	-2.23760700
H	9.97748600	1.69071900	-1.56404800
H	9.20118300	3.93834300	-0.83909900
H	6.77209900	4.45904300	-0.79103900
H	5.12012800	2.73081600	-1.47333000
H	3.87390800	-1.32370900	-1.31209000
H	1.51321900	-0.96108500	-2.12760700
H	1.75301200	0.79683700	-2.08199700
H	1.83707200	-1.08364600	0.36219400
H	2.02159900	0.66639800	0.42325600
H	-0.48561300	-0.82586000	-0.53360000
H	-0.26162400	-0.13475800	1.06357200

O. NMR Spectra

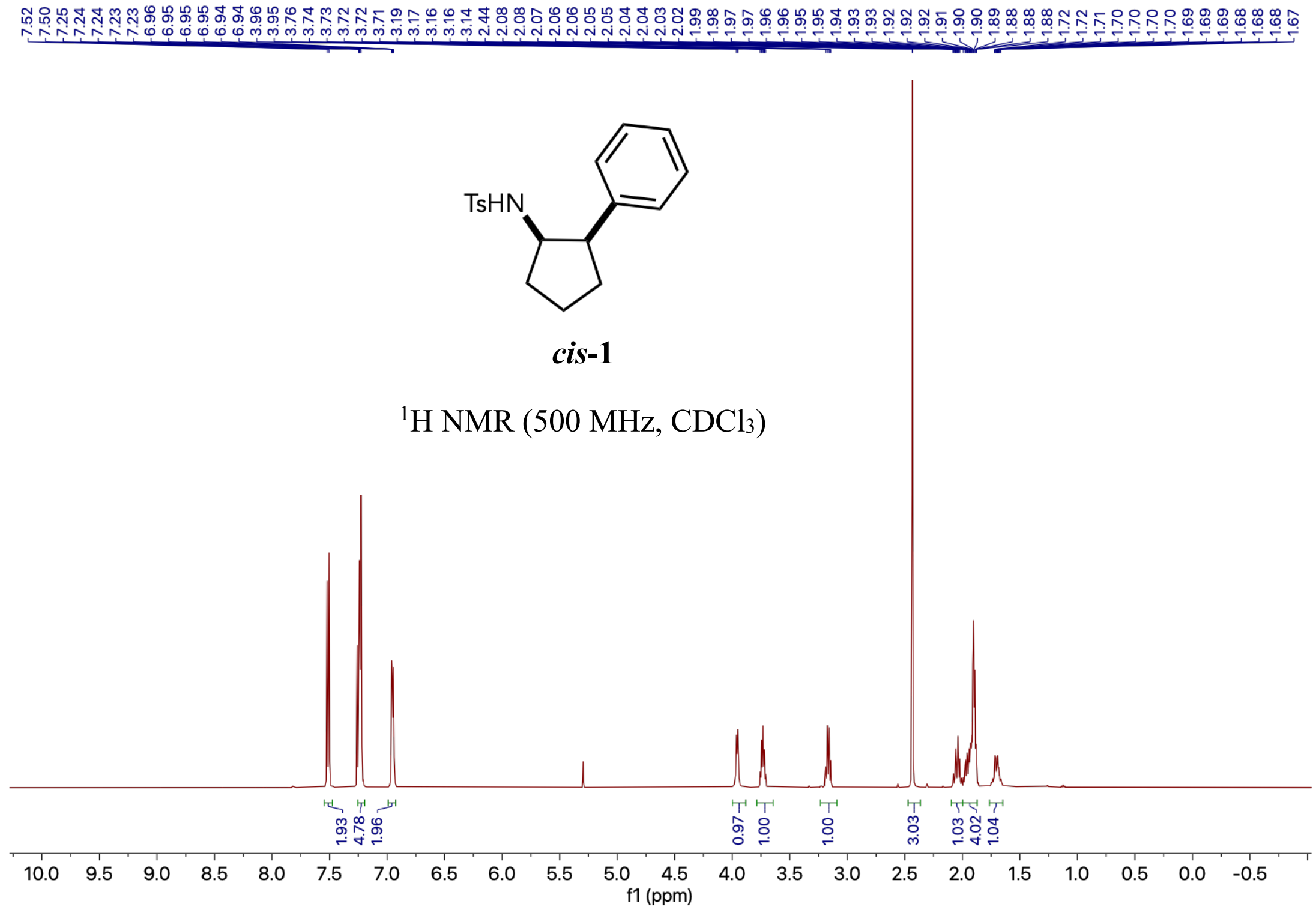

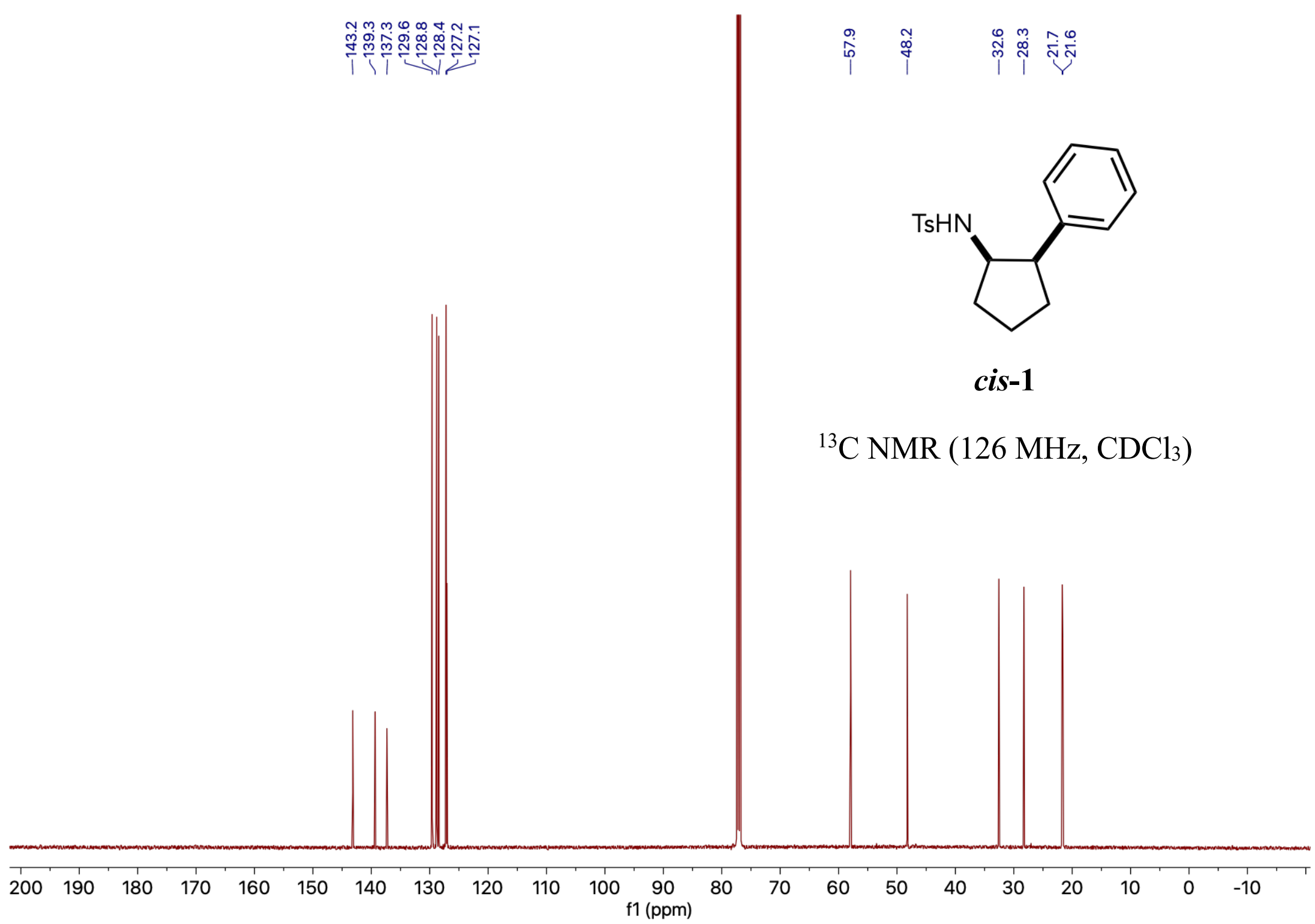

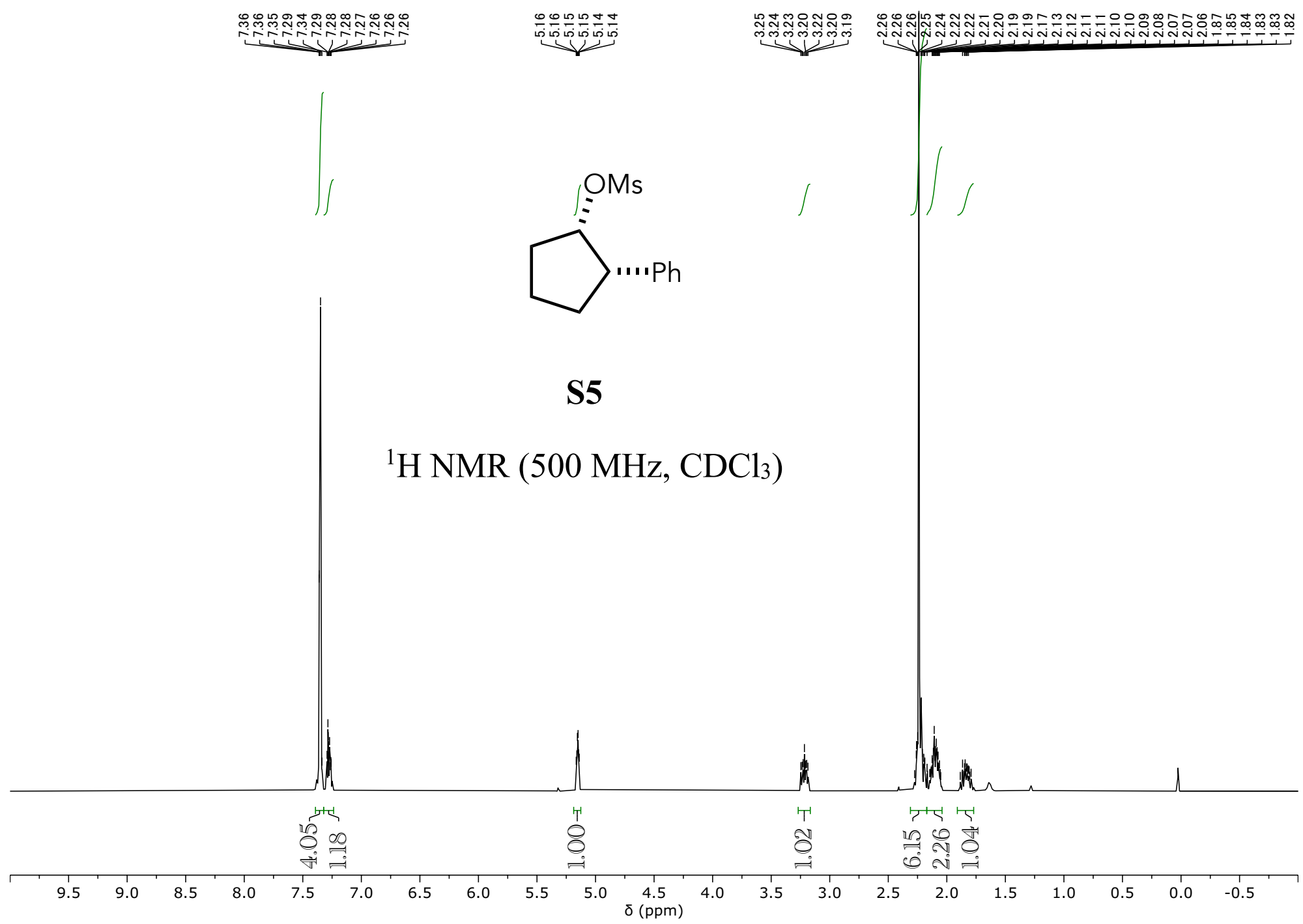
OMs
Ph
S5
^{1}H NMR (500 MHz, $CDCl_3$)
4.05
1.18
1.00
1.02
6.15
2.26
1.04
9.5 9.0 8.5 8.0 7.5 7.0 6.5 6.0 5.5 5.0 4.5 4.0 3.5 3.0 2.5 2.0 1.5 1.0 0.5 0.0 -0.5
δ (ppm)

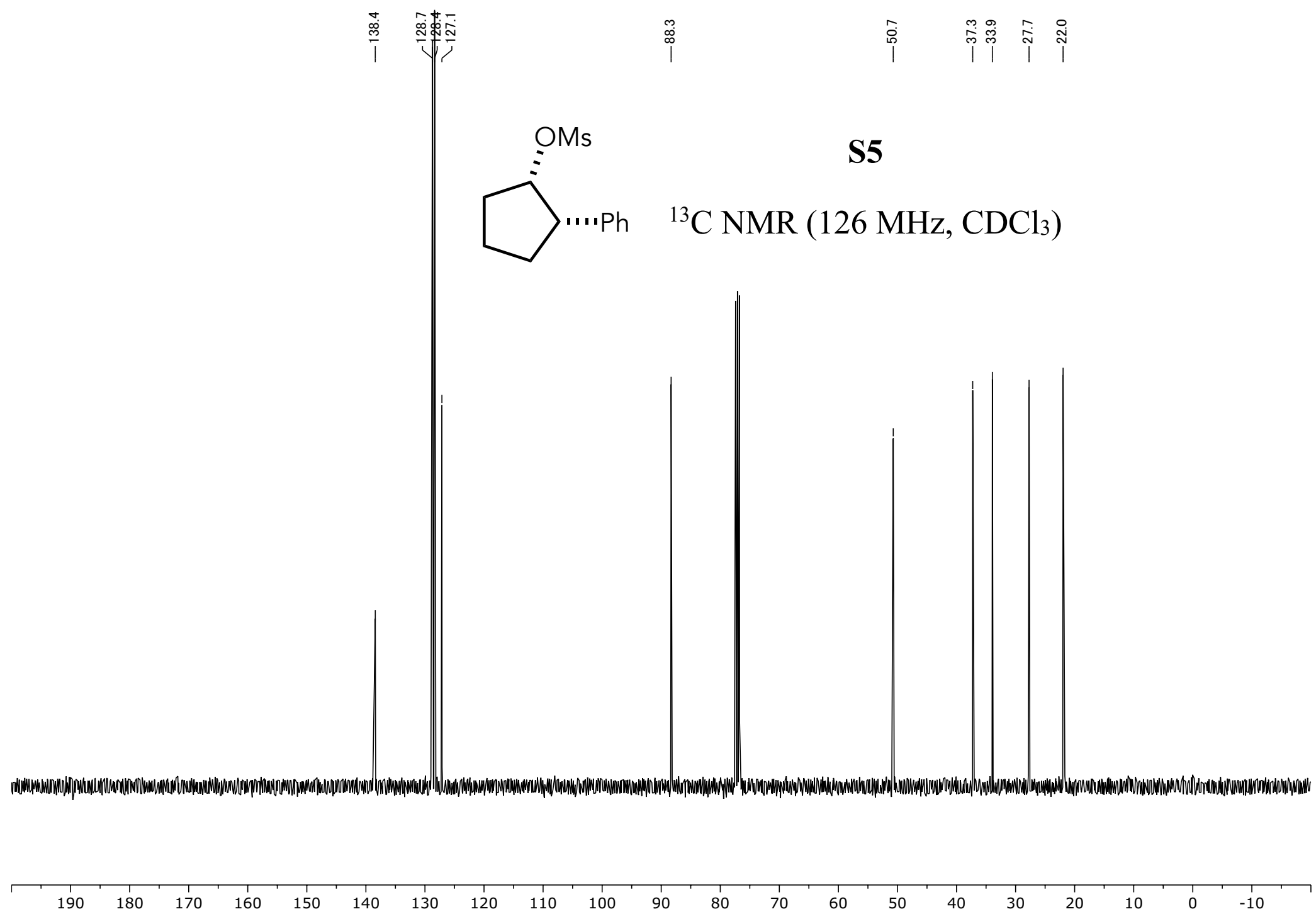
138.4
128.7
127.1
88.3
50.7
37.3
33.9
27.7
22.0
OMs
Ph
S5
^{13}C NMR (126 MHz, $CDCl_3$)
190 180 170 160 150 140 130 120 110 100 90 80 70 60 50 40 30 20 10 0 -10
δ (ppm)

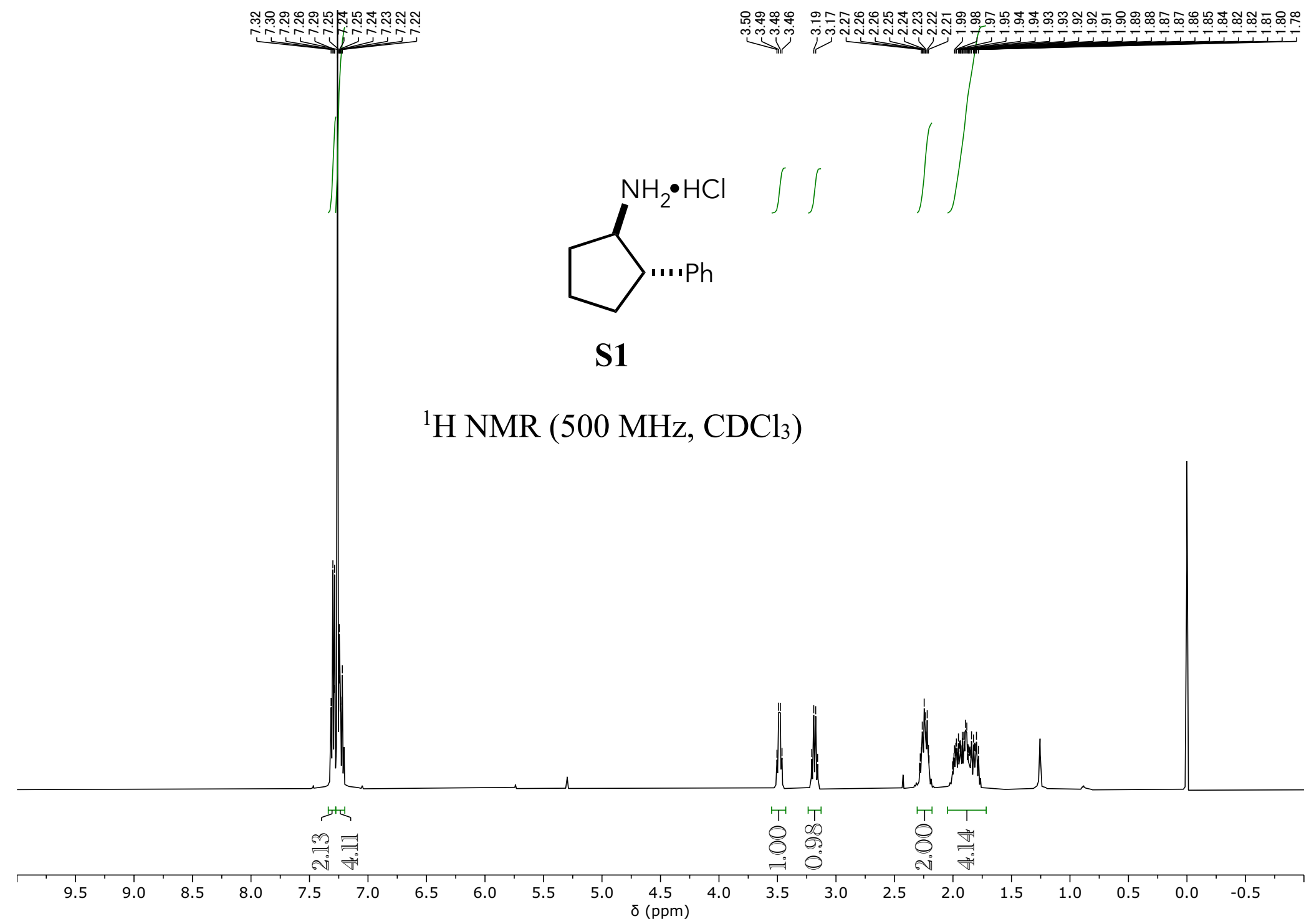
NH2•HCl
Ph
S1
1H NMR (500 MHz, CDCl3)
2.13
4.11
1.00
0.98
2.00
4.14
δ (ppm)

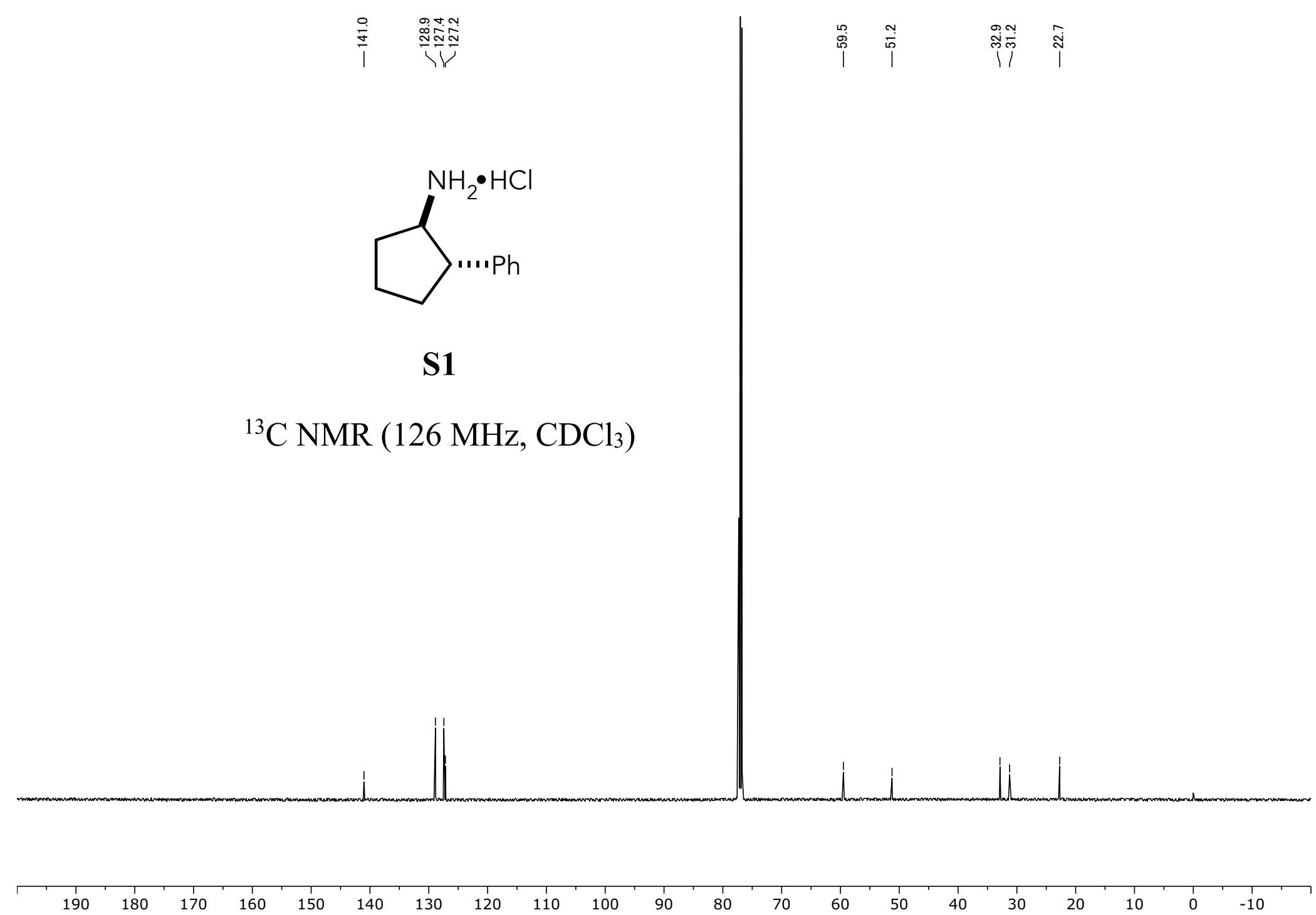
141.0
128.9
127.4
127.2
59.5
51.2
32.9
31.2
22.7
NH2•HCl
Ph
S1
13C NMR (126 MHz, CDCl3)
δ (ppm)

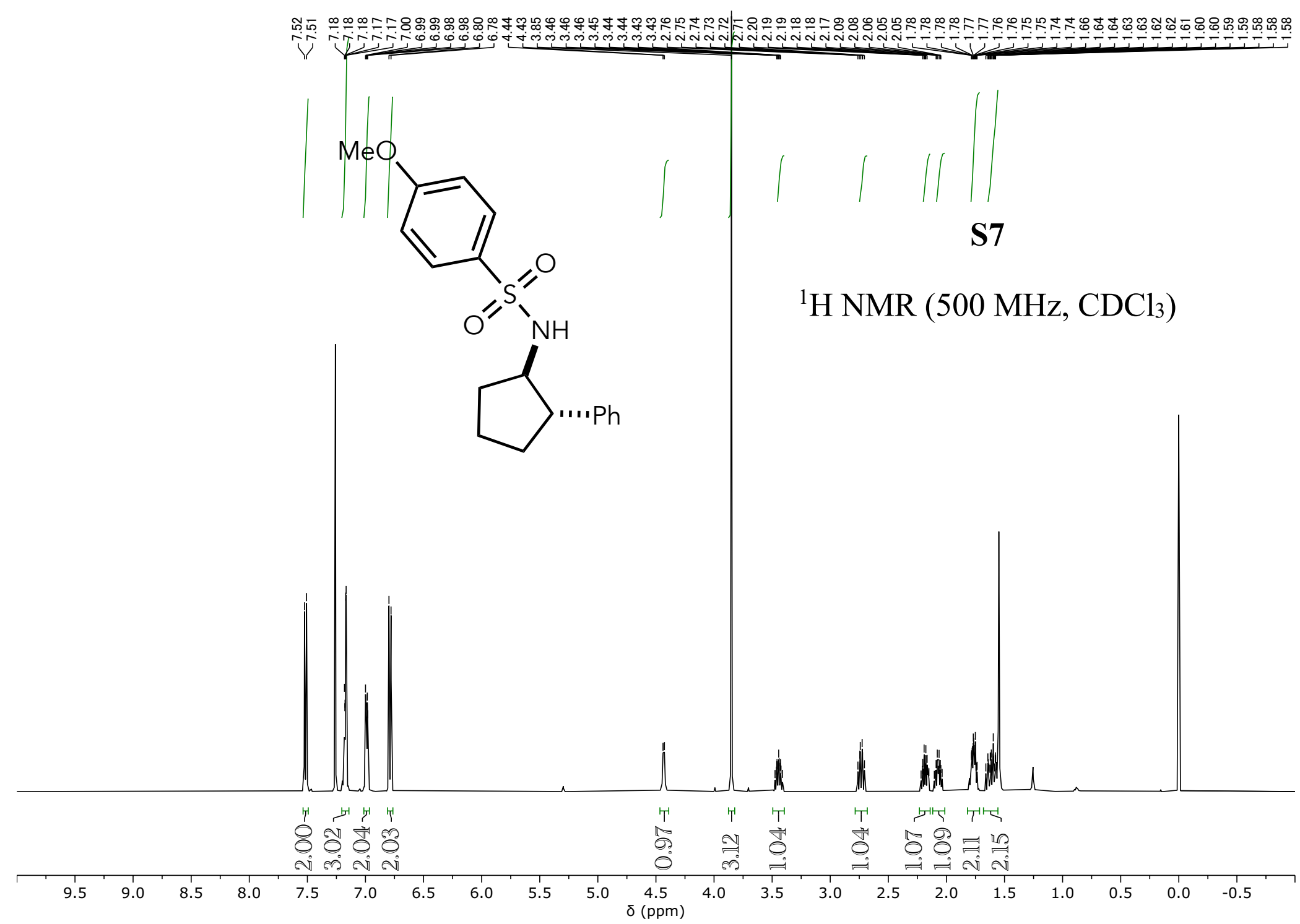

S7

^{1}H NMR (500 MHz, $CDCl_3$)

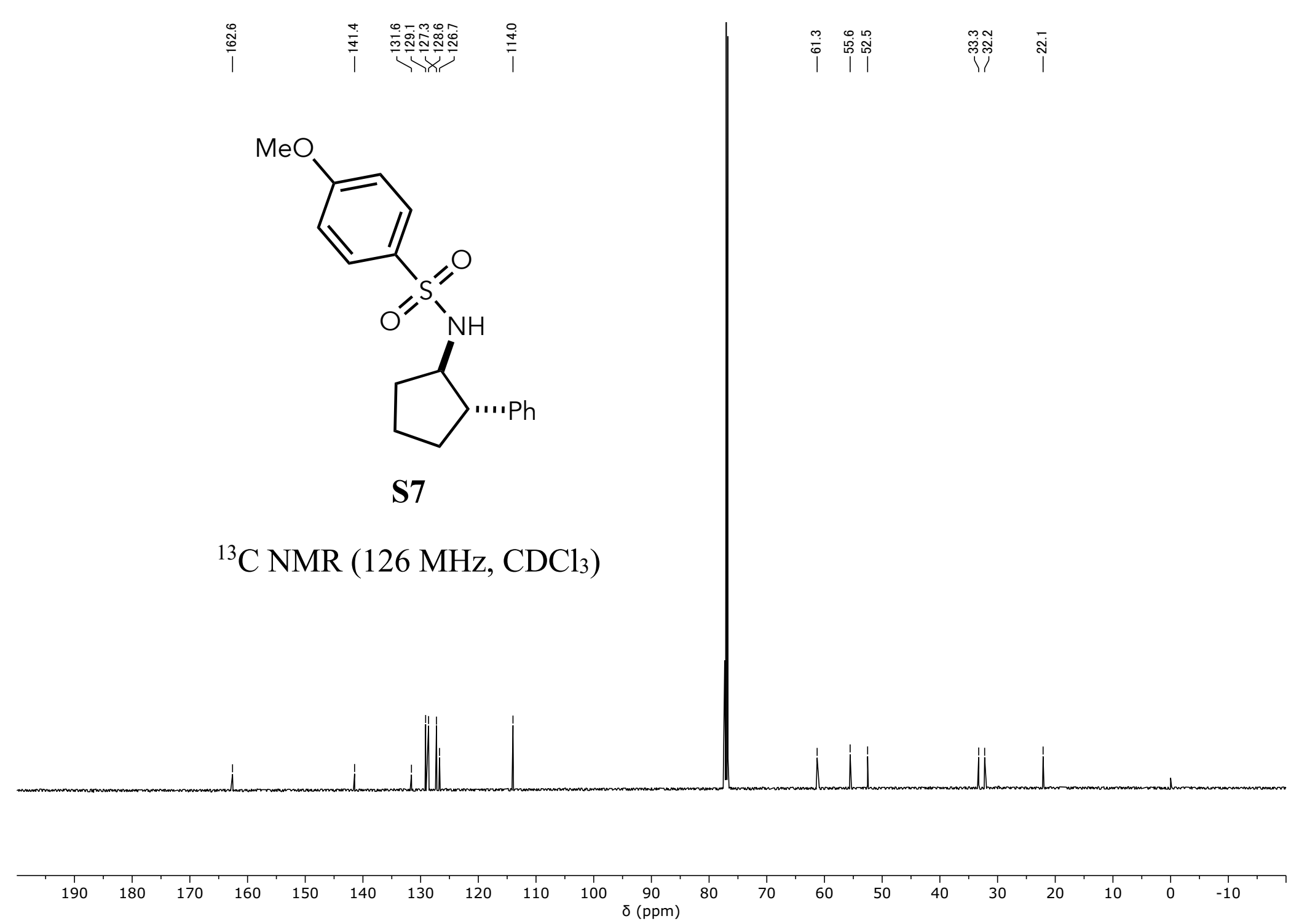

S7

^{13}C NMR (126 MHz, $CDCl_3$)

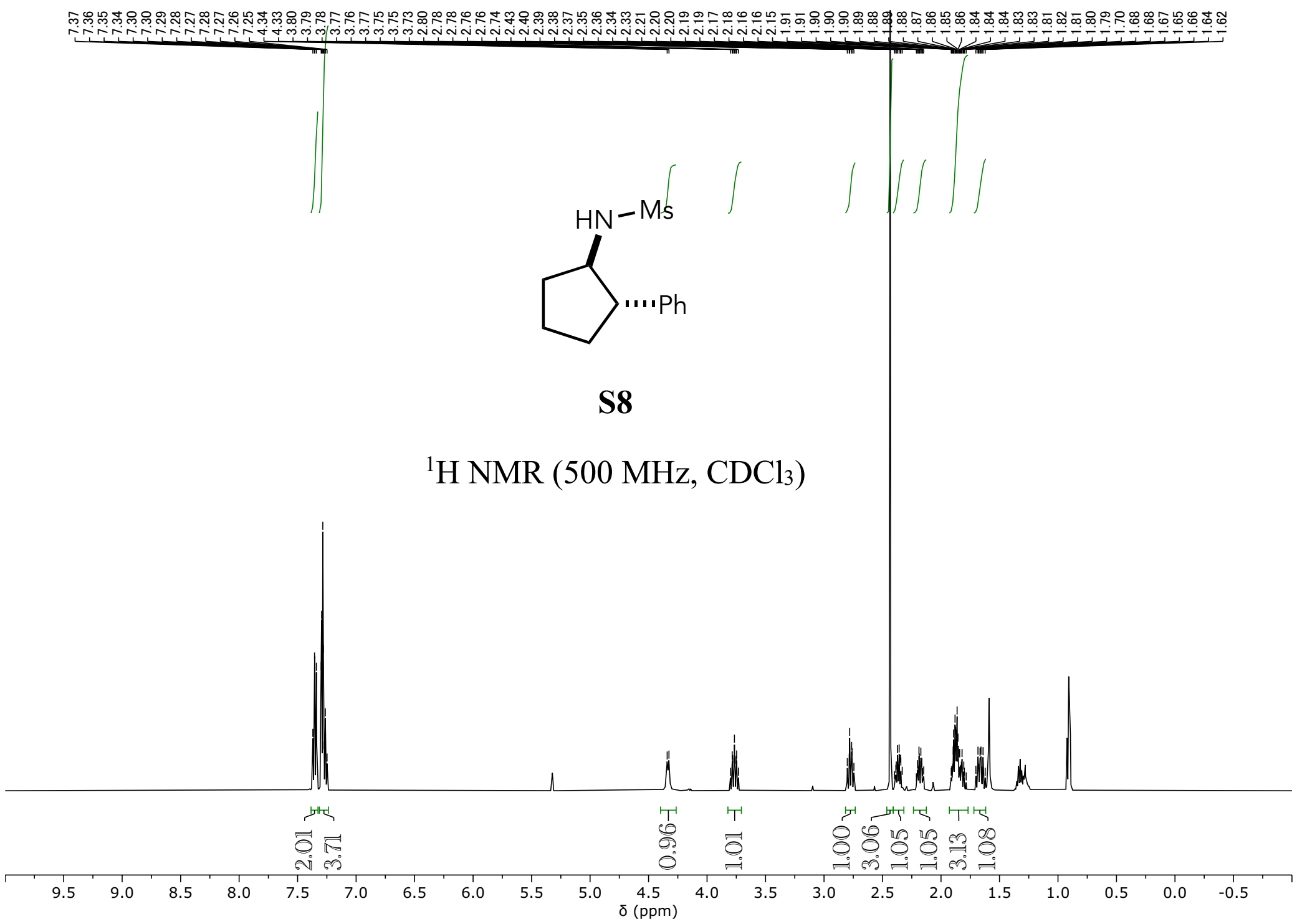

S8

^{1}H NMR (500 MHz, $CDCl_3$)

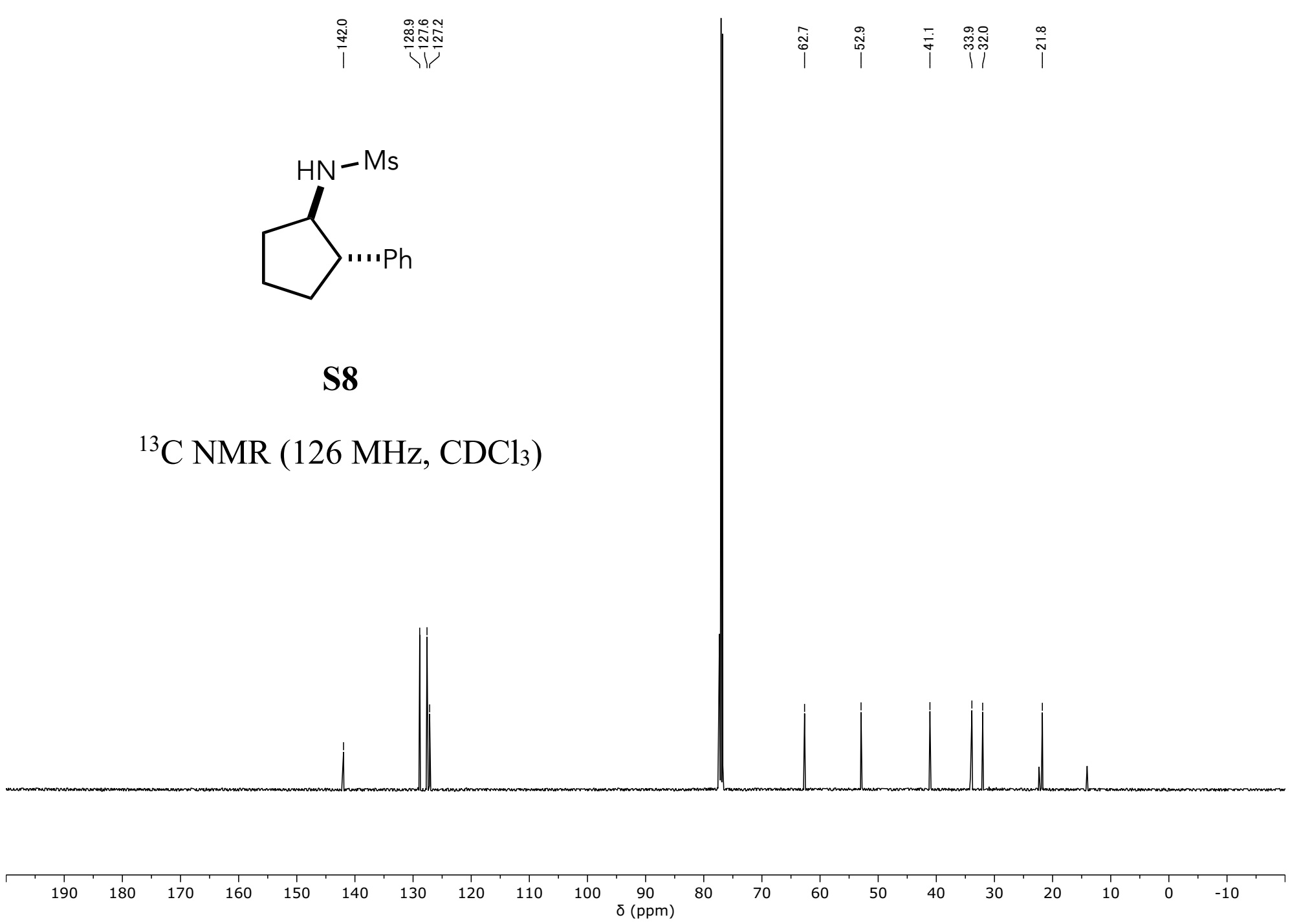

S8

^{13}C NMR (126 MHz, $CDCl_3$)

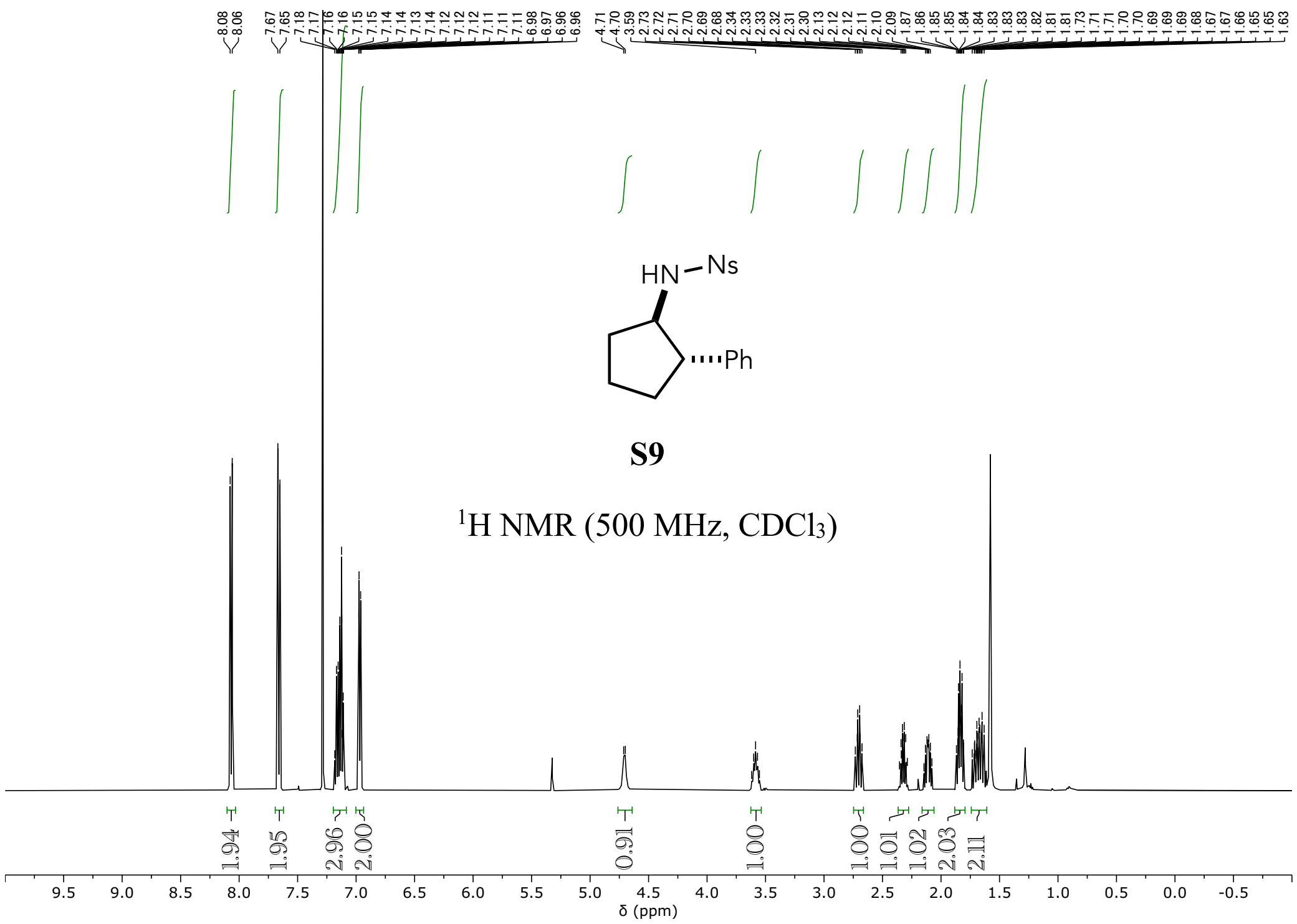

S9

^{1}H NMR (500 MHz, $CDCl_3$)

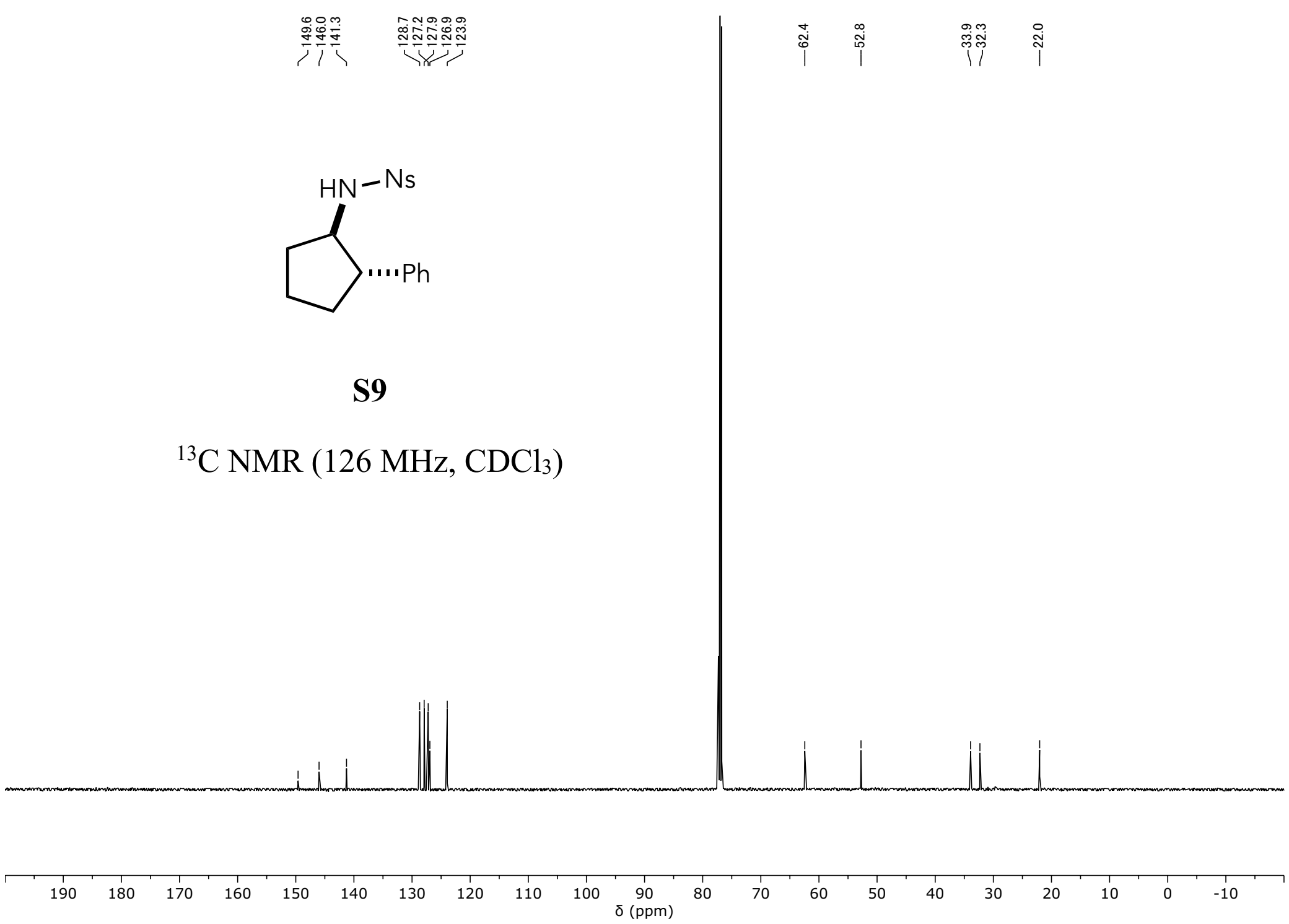

S9

^{13}C NMR (126 MHz, $CDCl_3$)

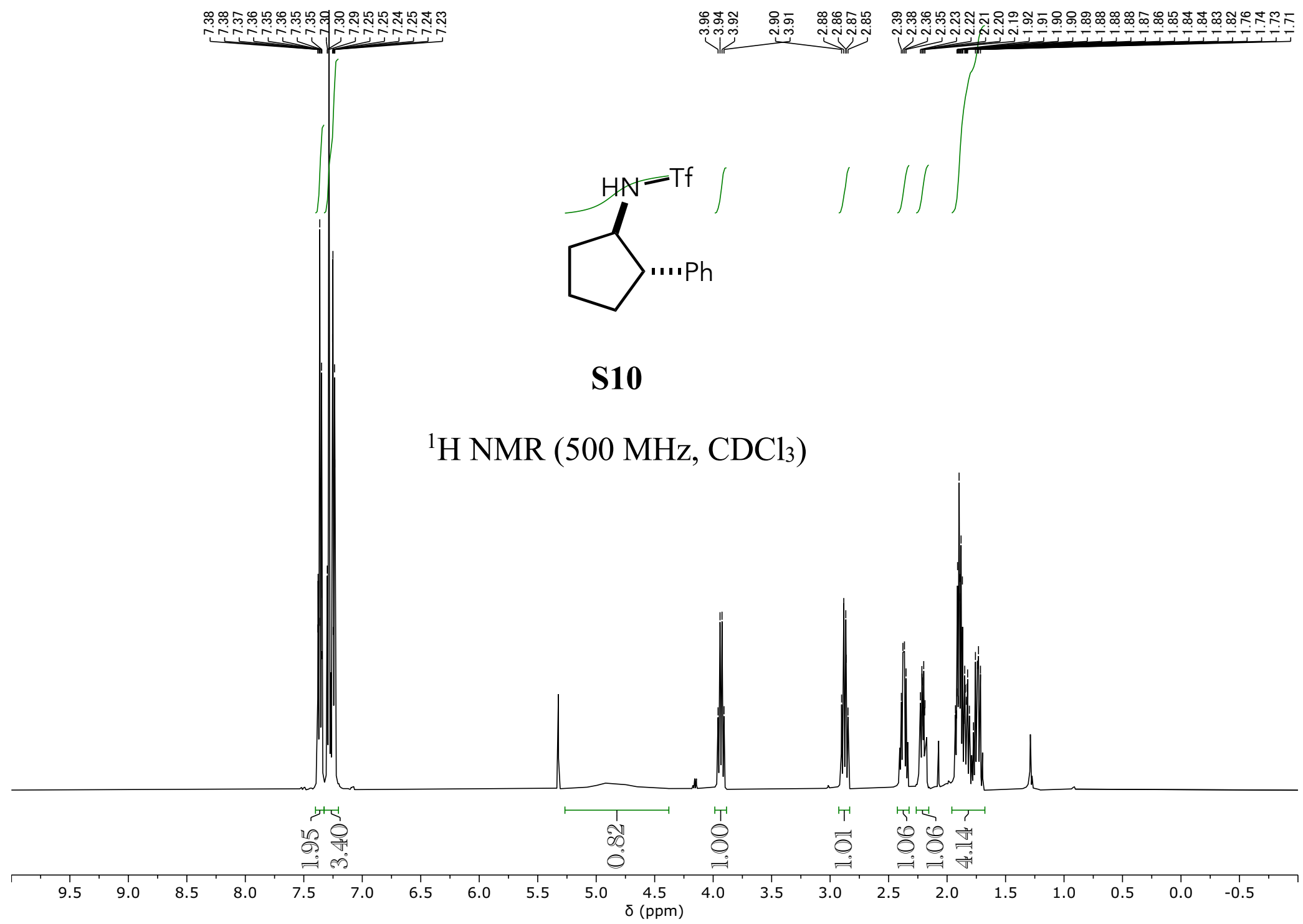
HN—Tf
Ph
S10
1H NMR (500 MHz, CDCl3)
δ (ppm)

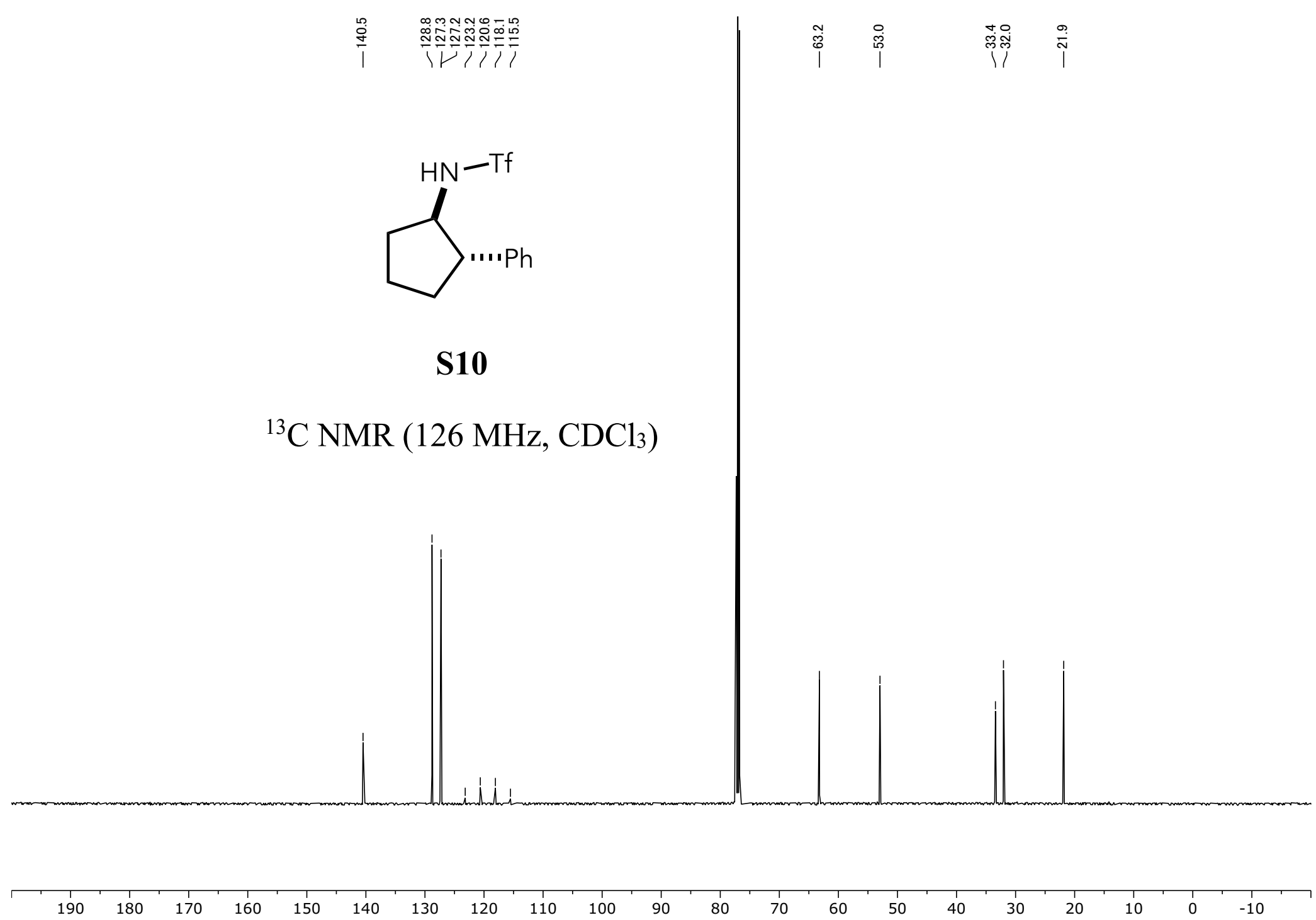
HN—Tf
Ph
S10
13C NMR (126 MHz, CDCl3)
δ (ppm)

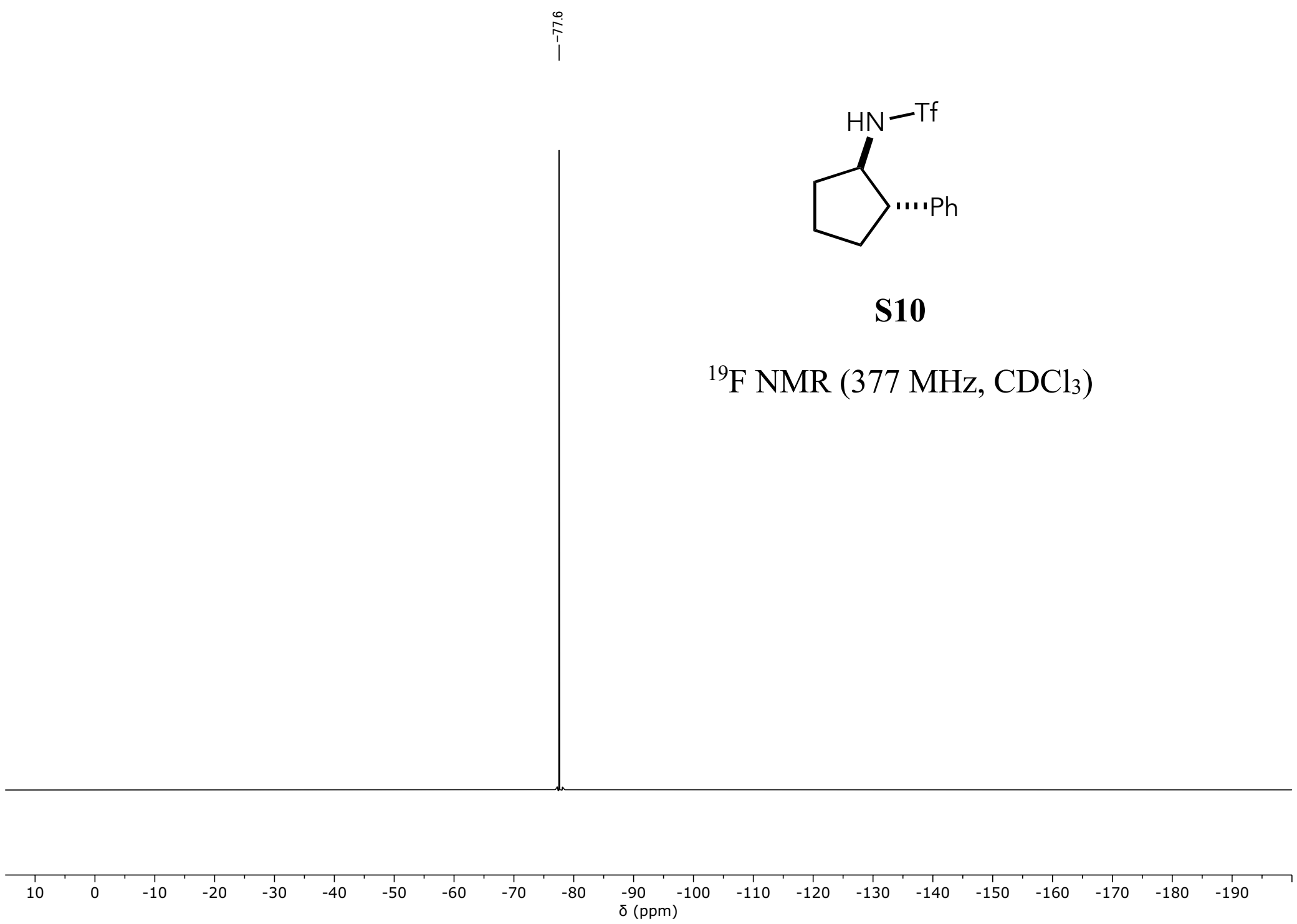

S10

^{19}F NMR (377 MHz, $CDCl_3$)

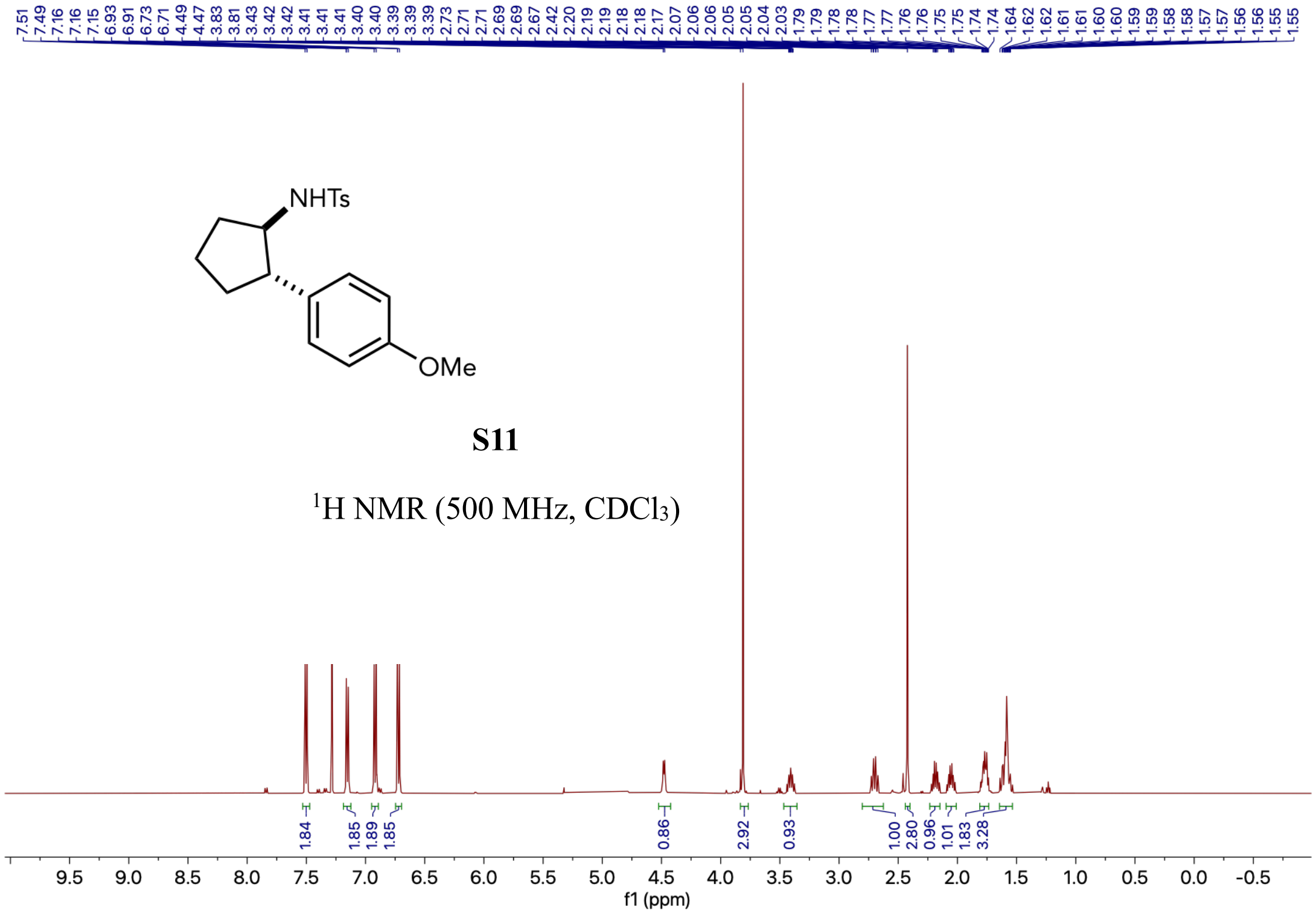
NHTs
OMe
S11
^{1}H NMR (500 MHz, $CDCl_3$)
f1 (ppm)

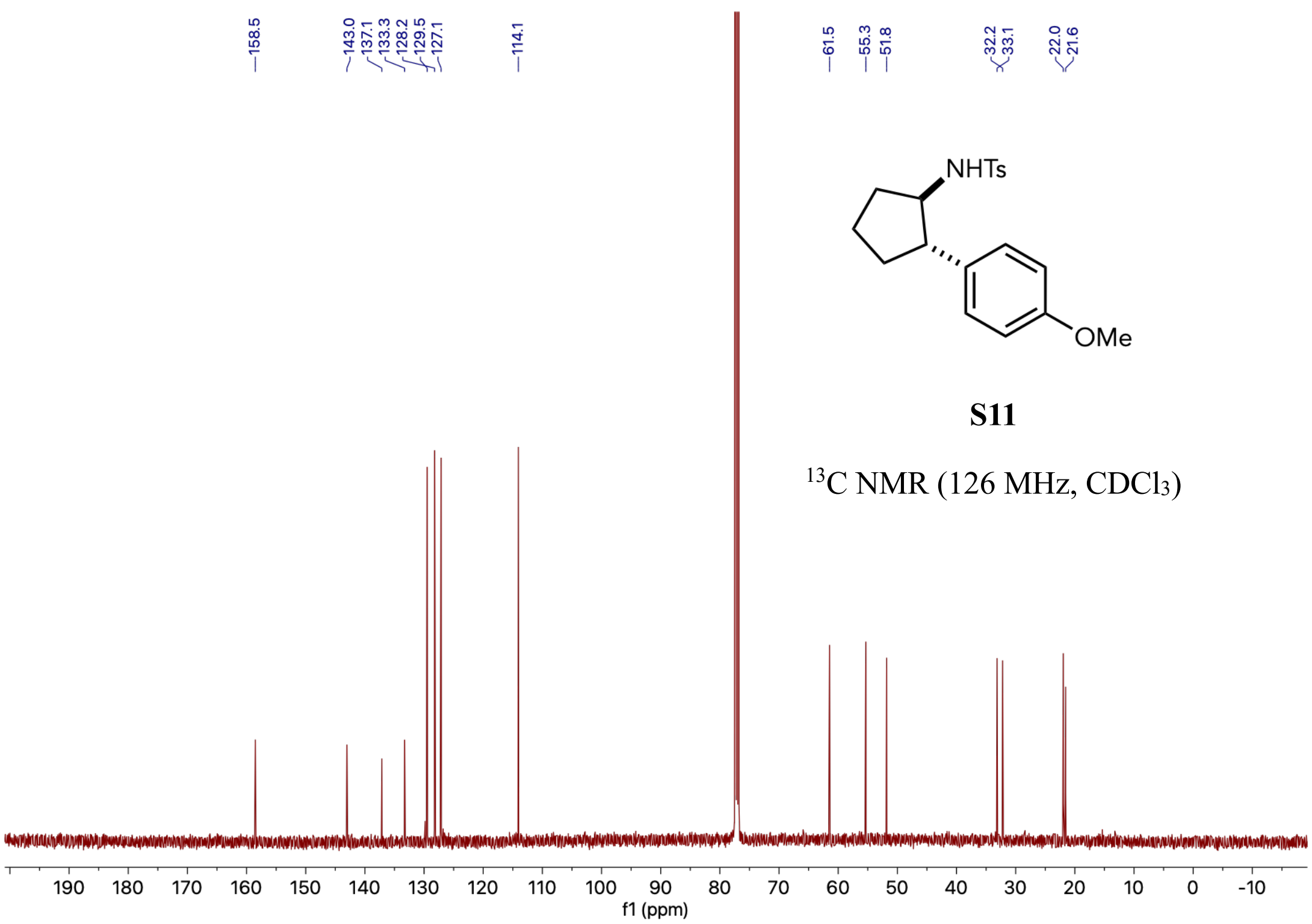
NHTs
OMe
S11
^{13}C NMR (126 MHz, $CDCl_3$)
f1 (ppm)

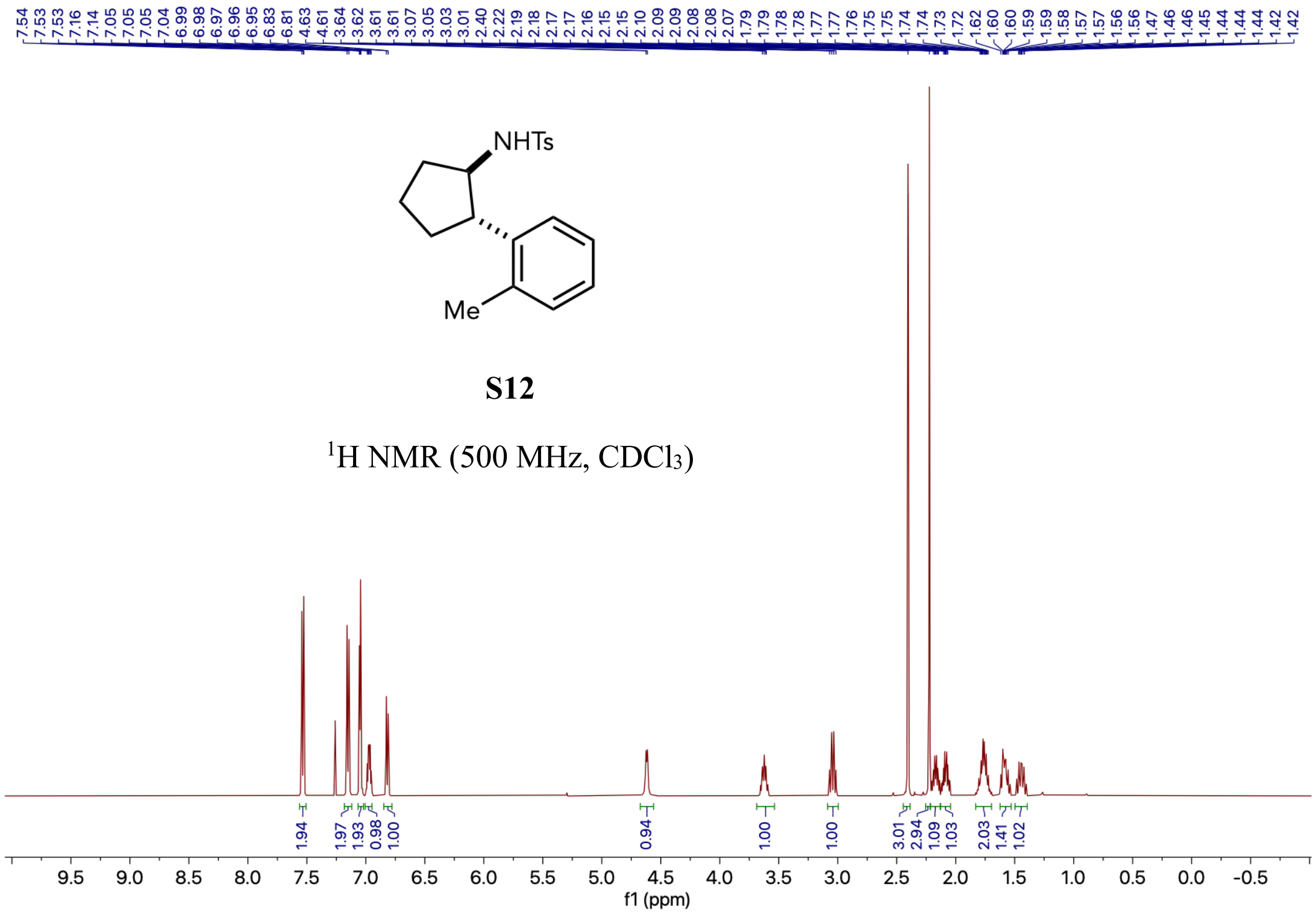
7.54 7.53 7.53 7.16 7.14 7.05 7.05 7.05 7.04 6.99 6.98 6.97 6.96 6.95 6.83 6.81 4.63 4.61 3.64 3.62 3.61 3.61 3.07 3.05 3.03 3.01 2.40 2.22 2.19 2.18 2.17 2.17 2.16 2.15 2.15 2.10 2.09 2.09 2.08 2.07 1.79 1.79 1.78 1.78 1.77 1.77 1.76 1.75 1.75 1.74 1.74 1.73 1.72 1.62 1.60 1.60 1.59 1.59 1.58 1.57 1.56 1.47 1.46 1.45 1.44 1.44 1.44 1.42 1.42
NHTs
Me
S12
1H NMR (500 MHz, CDCl3)
1.94 1.97 1.93 0.98 1.00 0.94 1.00 1.00 3.01 2.94 1.09 1.03 2.03 1.41 1.02
f1 (ppm)

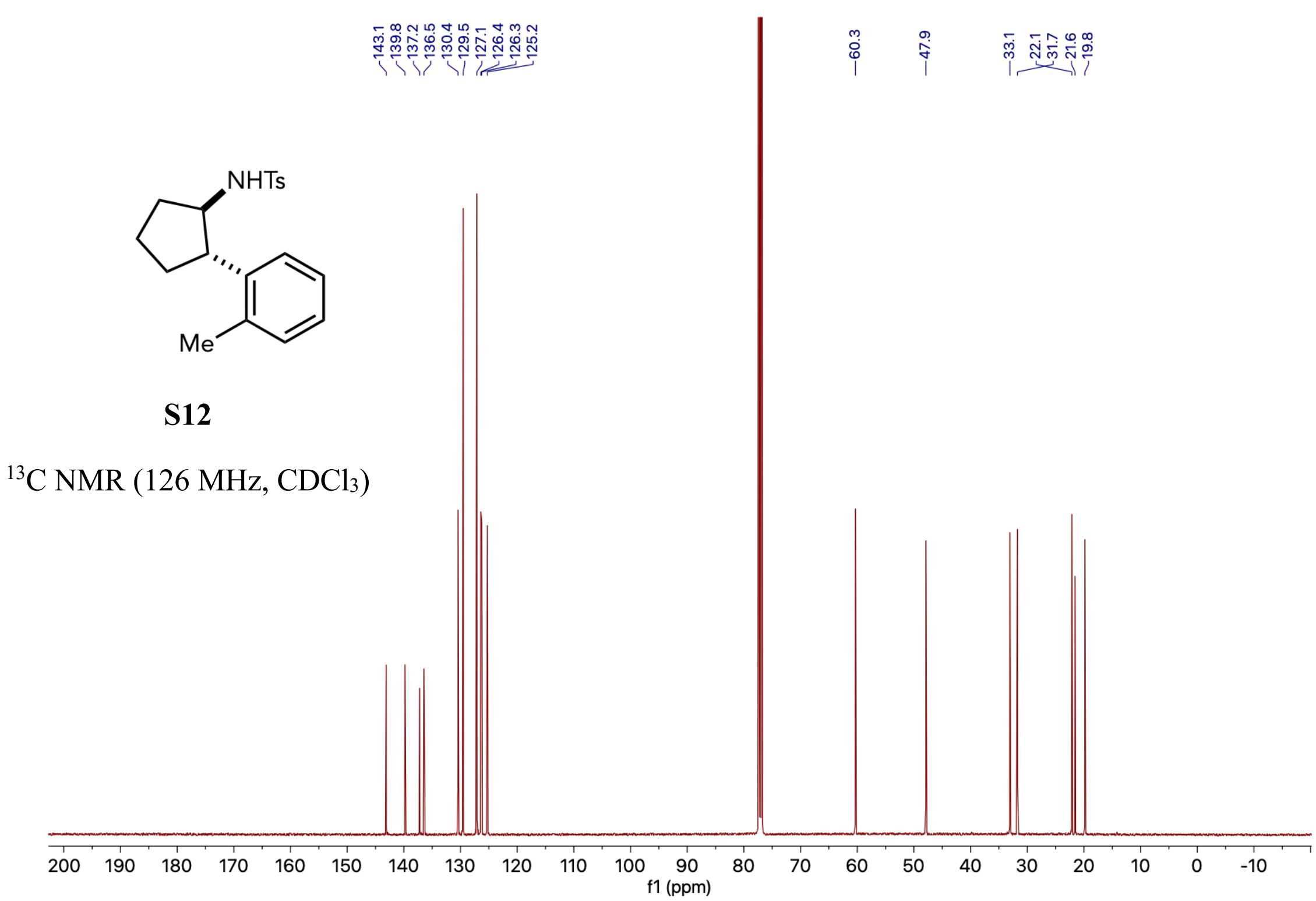
143.1 139.8 137.2 136.5 130.4 129.5 127.1 126.4 126.3 125.2
60.3
47.9
33.1 22.1 31.7 21.6 19.8
NHTs
Me
S12
13C NMR (126 MHz, CDCl3)
f1 (ppm)

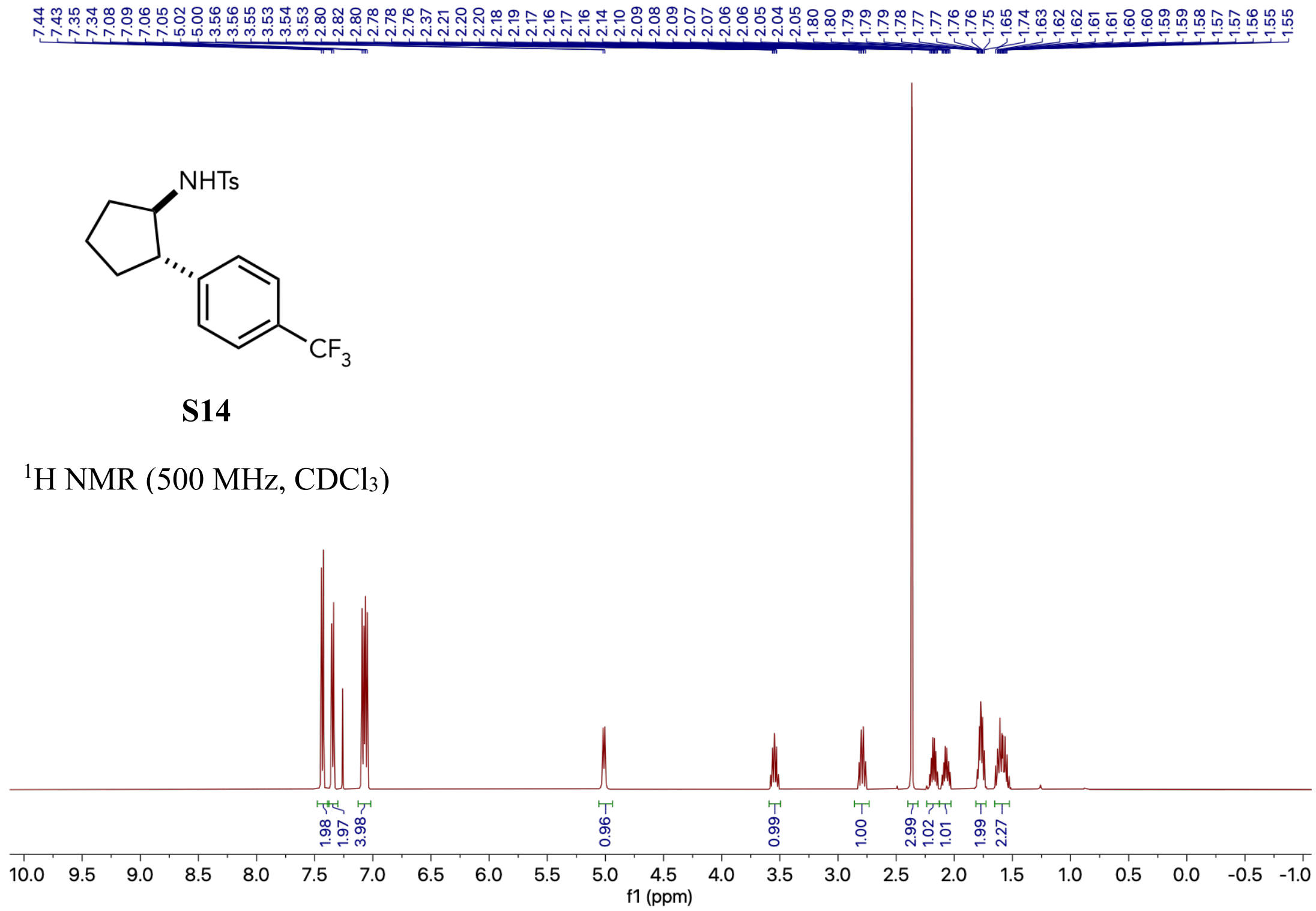

S14

^{1}H NMR (500 MHz, CDCl$_3$)

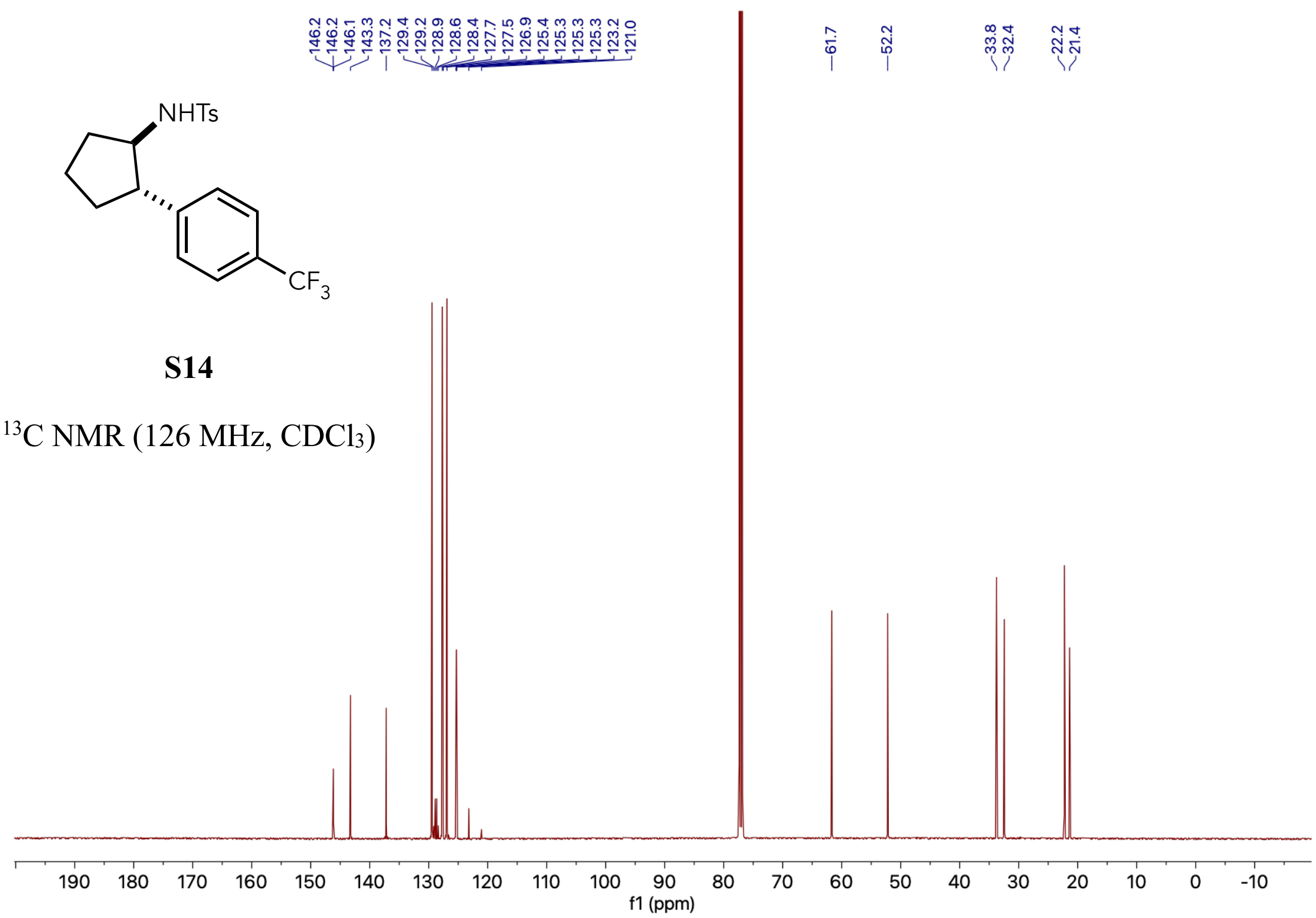

S14

^{13}C NMR (126 MHz, CDCl$_3$)

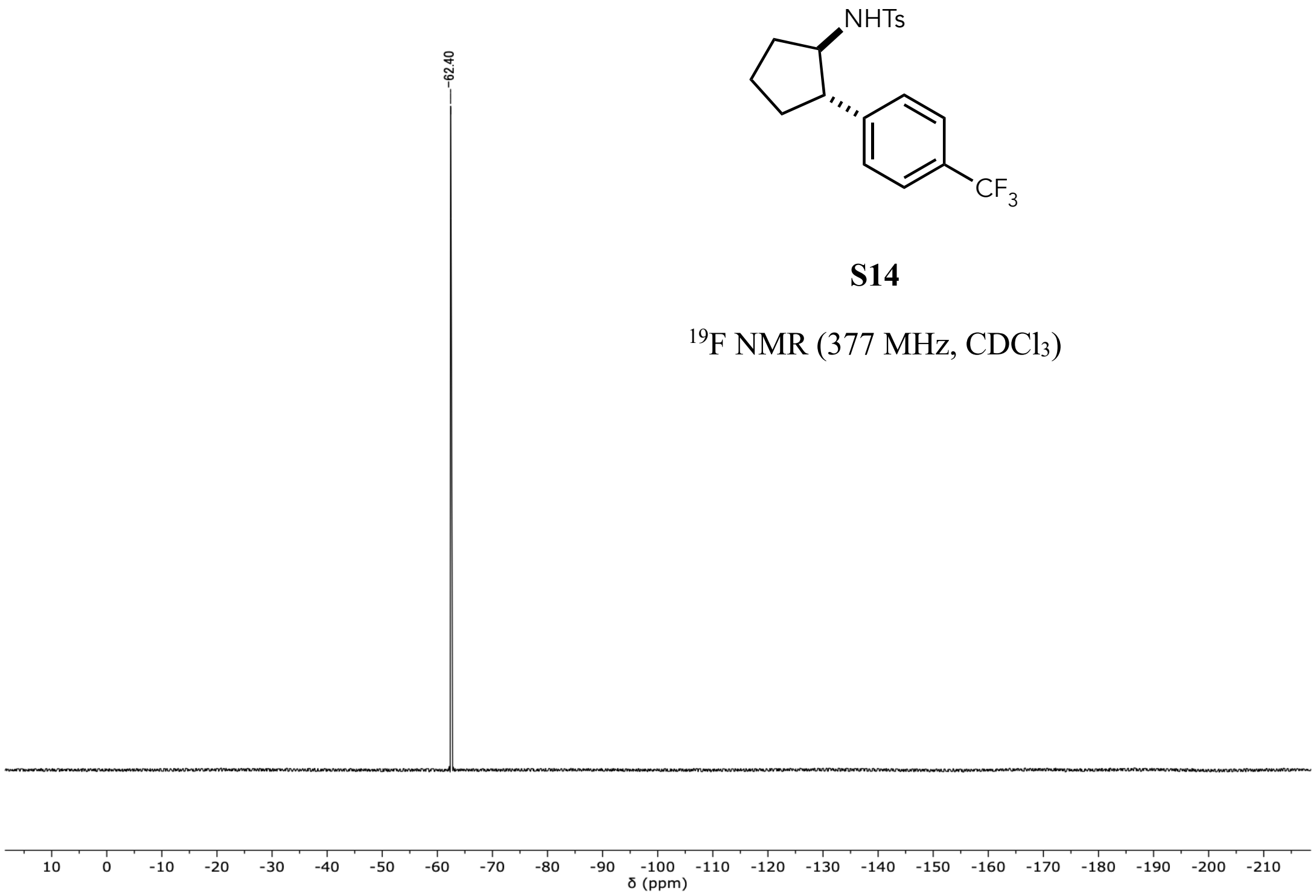

S14

^{19}F NMR (377 MHz, $CDCl_3$)

S16

^{1}H NMR (500 MHz, $CDCl_3$)

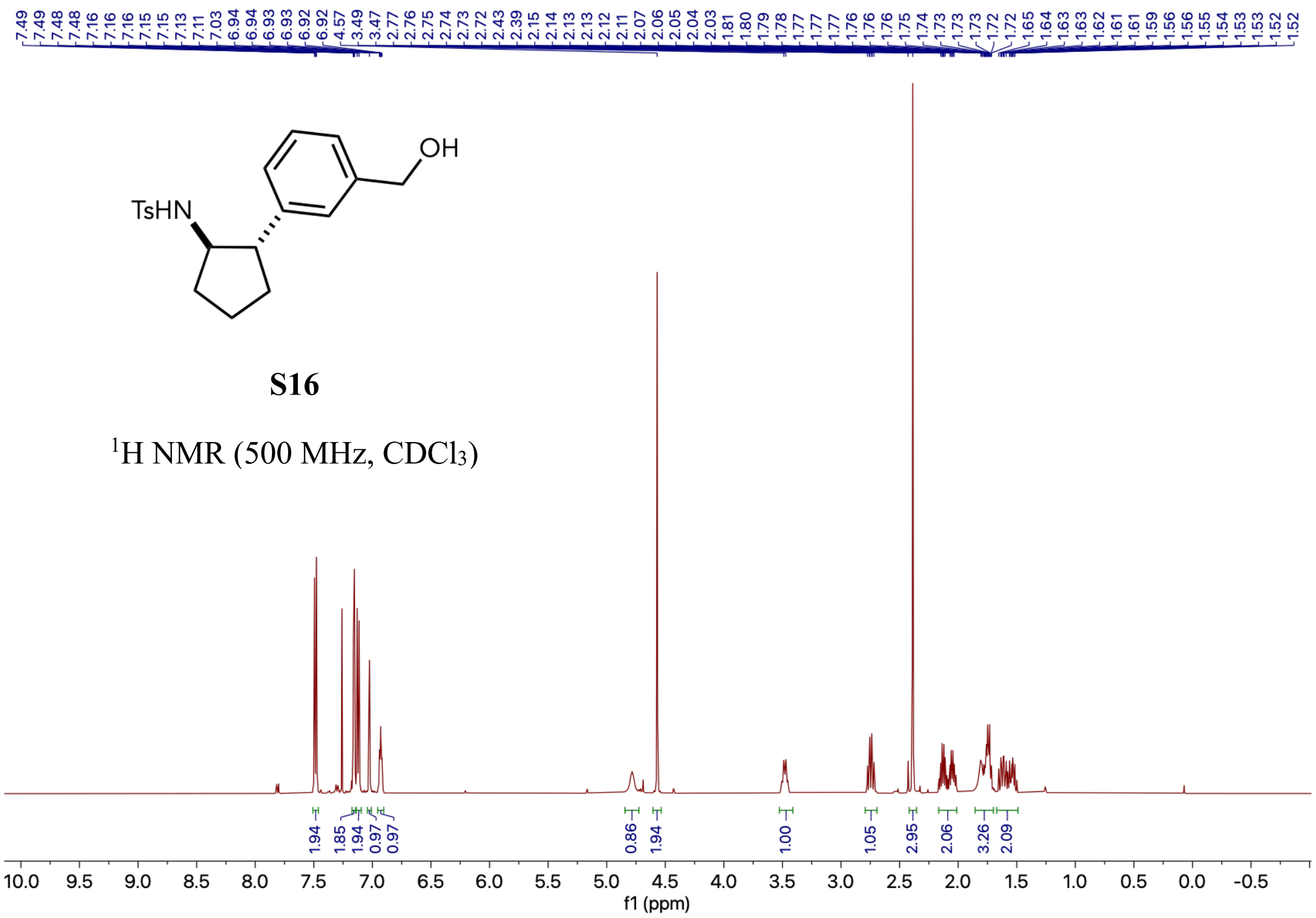

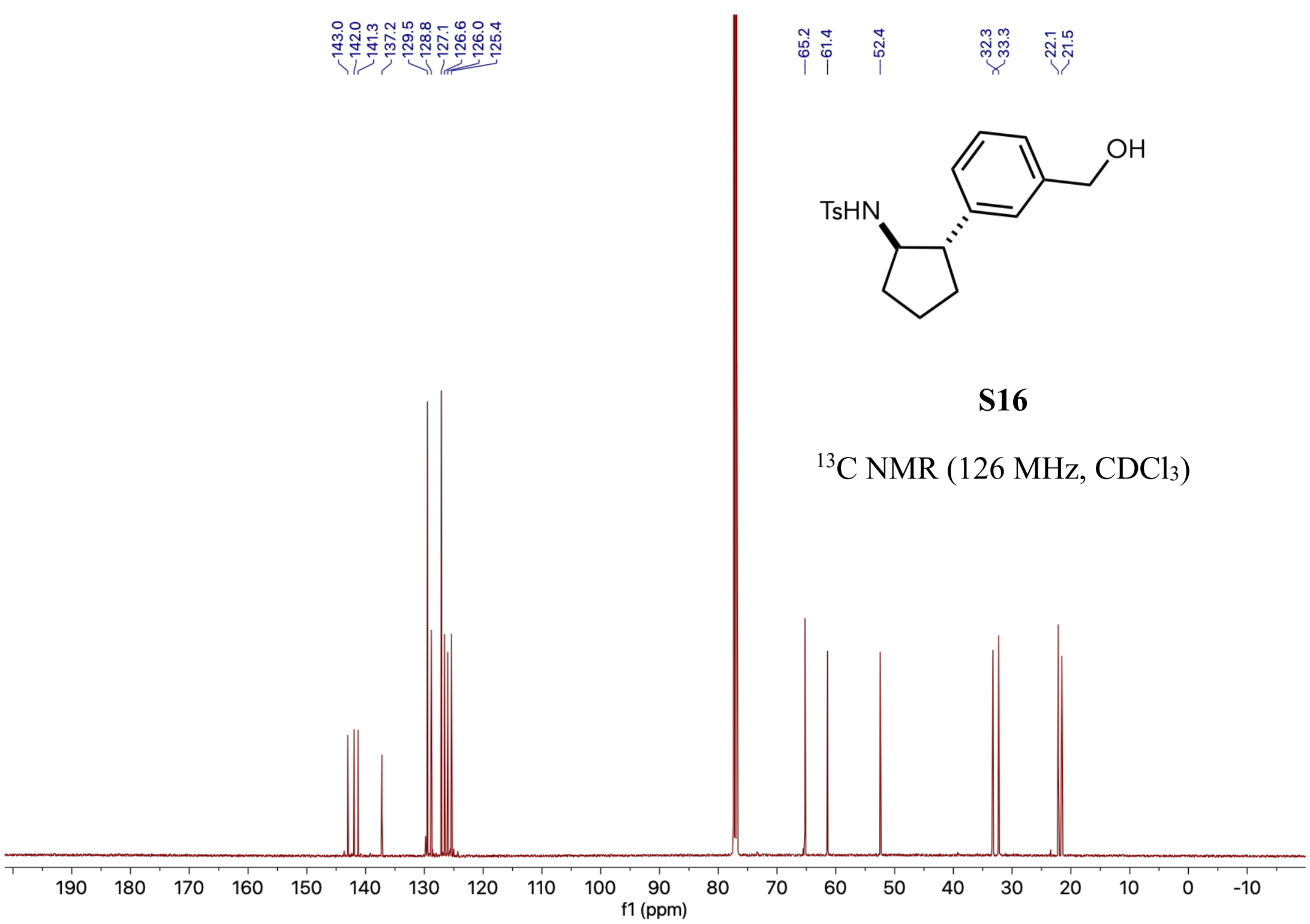

S16

^{13}C NMR (126 MHz, $CDCl_3$)

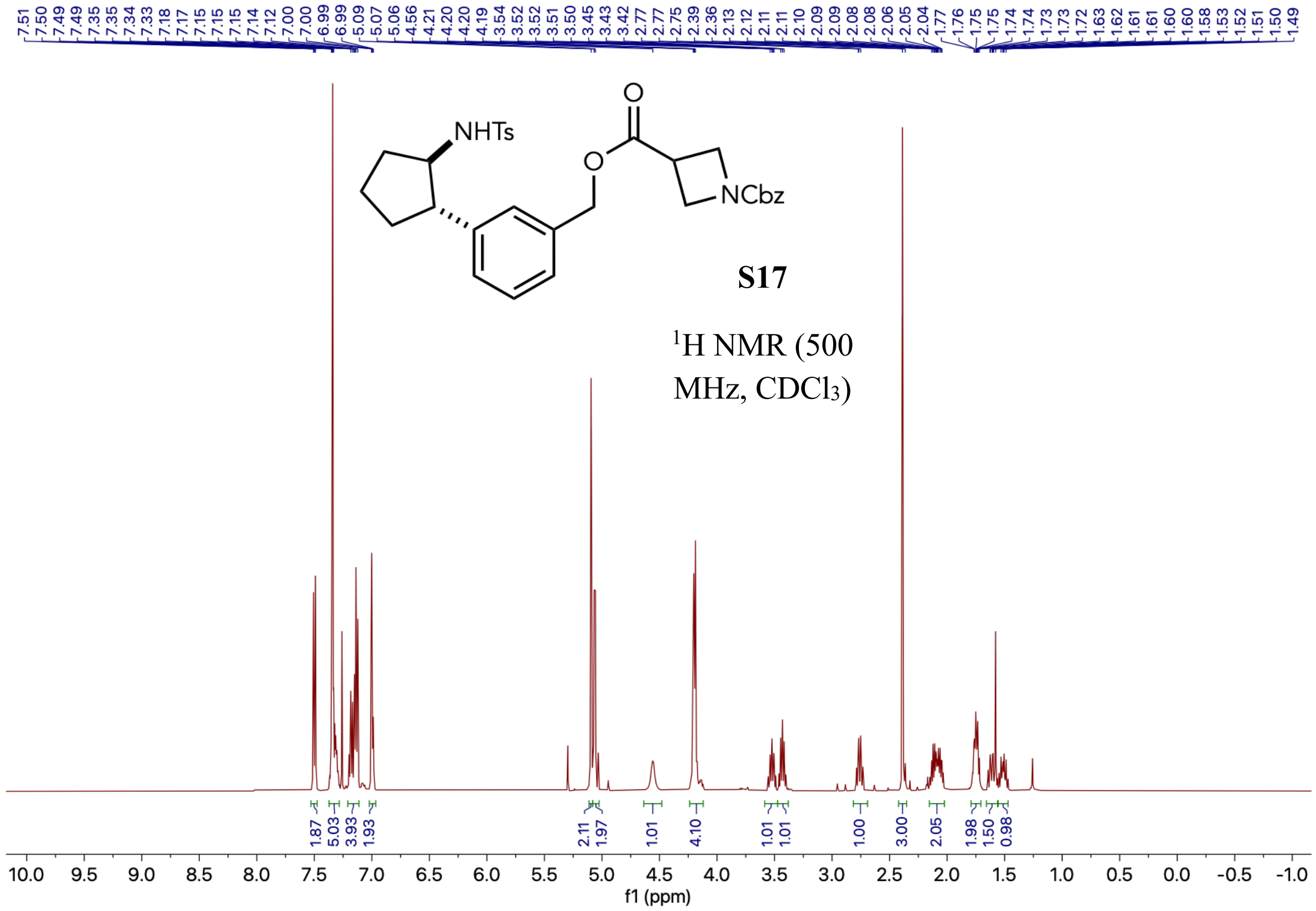
7.51 7.50 7.49 7.35 7.35 7.34 7.33 7.18 7.17 7.15 7.15 7.15 7.14 7.12 7.00 7.00 6.99 6.99 5.09 5.07 5.06 4.56 4.21 4.20 4.20 4.19 3.54 3.52 3.52 3.51 3.50 3.45 3.43 3.42 2.77 2.77 2.75 2.39 2.36 2.13 2.12 2.11 2.11 2.10 2.09 2.09 2.08 2.08 2.06 2.05 2.04 1.77 1.76 1.75 1.75 1.74 1.74 1.73 1.73 1.72 1.63 1.62 1.61 1.60 1.58 1.53 1.52 1.51 1.50 1.49
NHTs
O
O
NCbz
S17
^{1}H NMR (500 MHz, CDCl$_3$)
1.87 5.03 3.93 1.93 2.11 1.97 1.01 4.10 1.01 1.01 1.00 3.00 2.05 1.98 1.50 0.98
10.0 9.5 9.0 8.5 8.0 7.5 7.0 6.5 6.0 5.5 5.0 4.5 4.0 3.5 3.0 2.5 2.0 1.5 1.0 0.5 0.0 -0.5 -1.0
f1 (ppm)

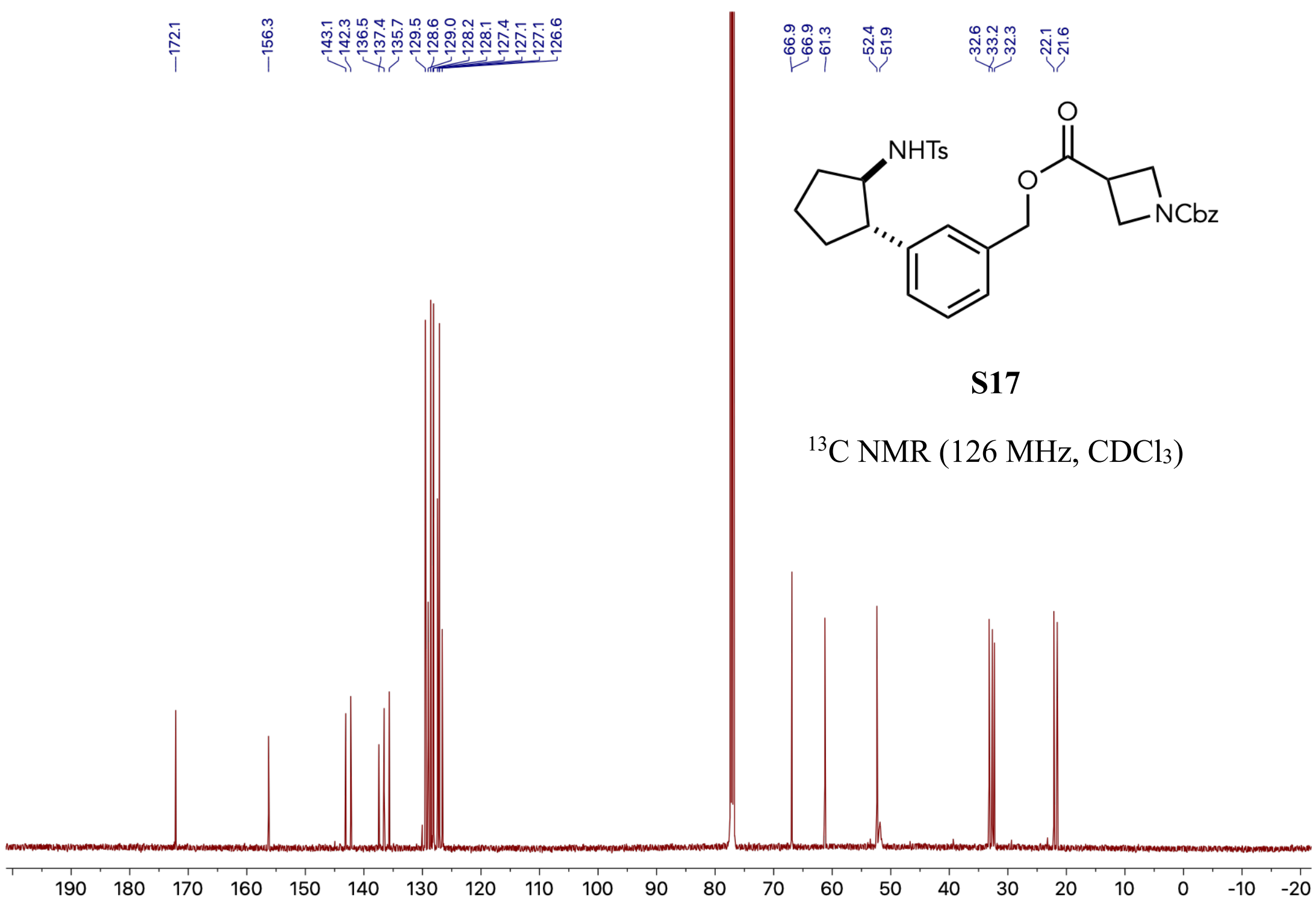
172.1
156.3
143.1 142.3 136.5 137.4 135.7 129.5 128.6 129.0 128.2 128.1 127.4 127.1 126.6
66.9 66.9 61.3
52.4 51.9
32.6 33.2 32.3
22.1 21.6
NHTs
O
O
NCbz
S17
^{13}C NMR (126 MHz, CDCl$_3$)
190 180 170 160 150 140 130 120 110 100 90 80 70 60 50 40 30 20 10 0 -10 -20
f1 (ppm)

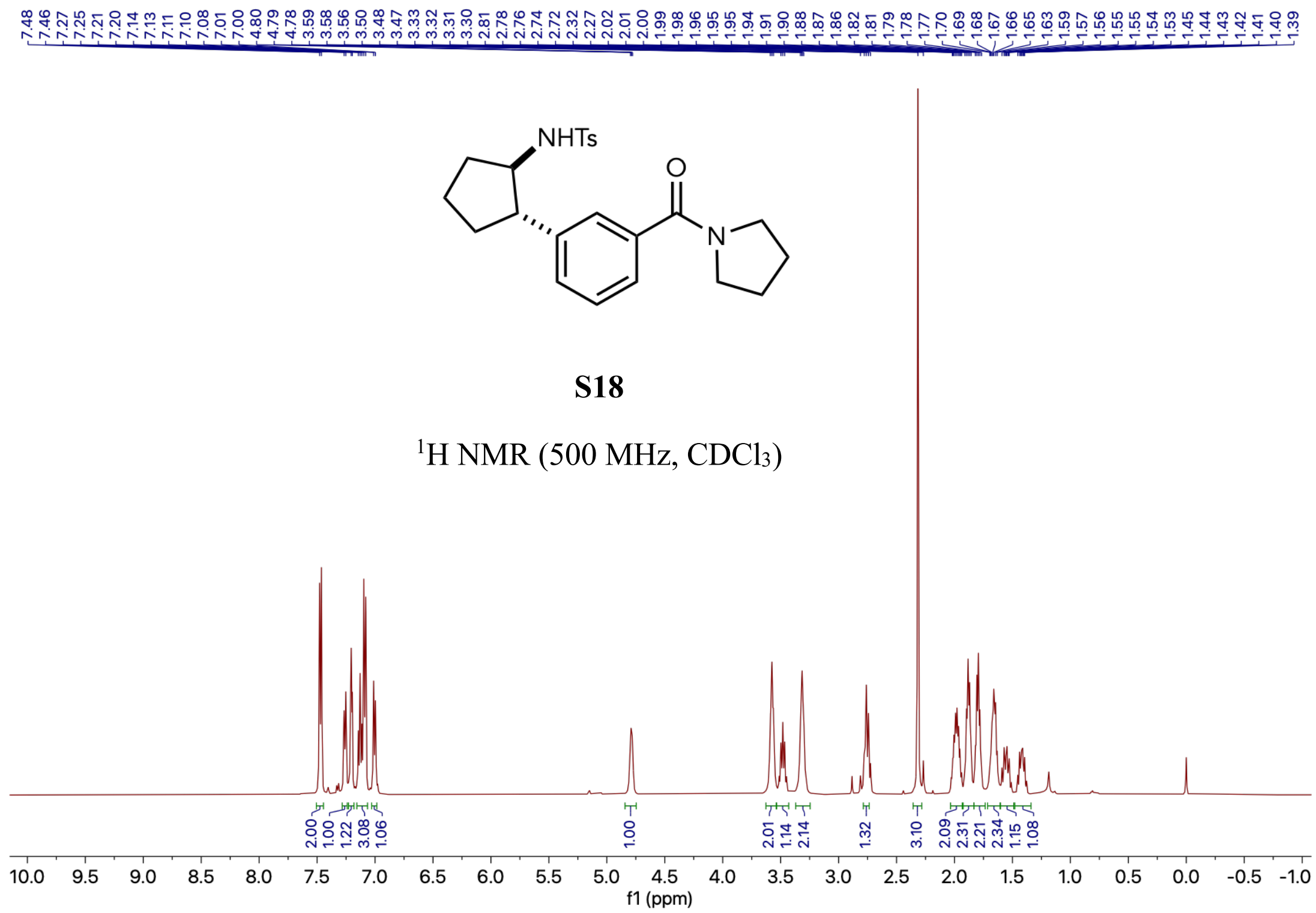

S18

^{1}H NMR (500 MHz, $CDCl_3$)

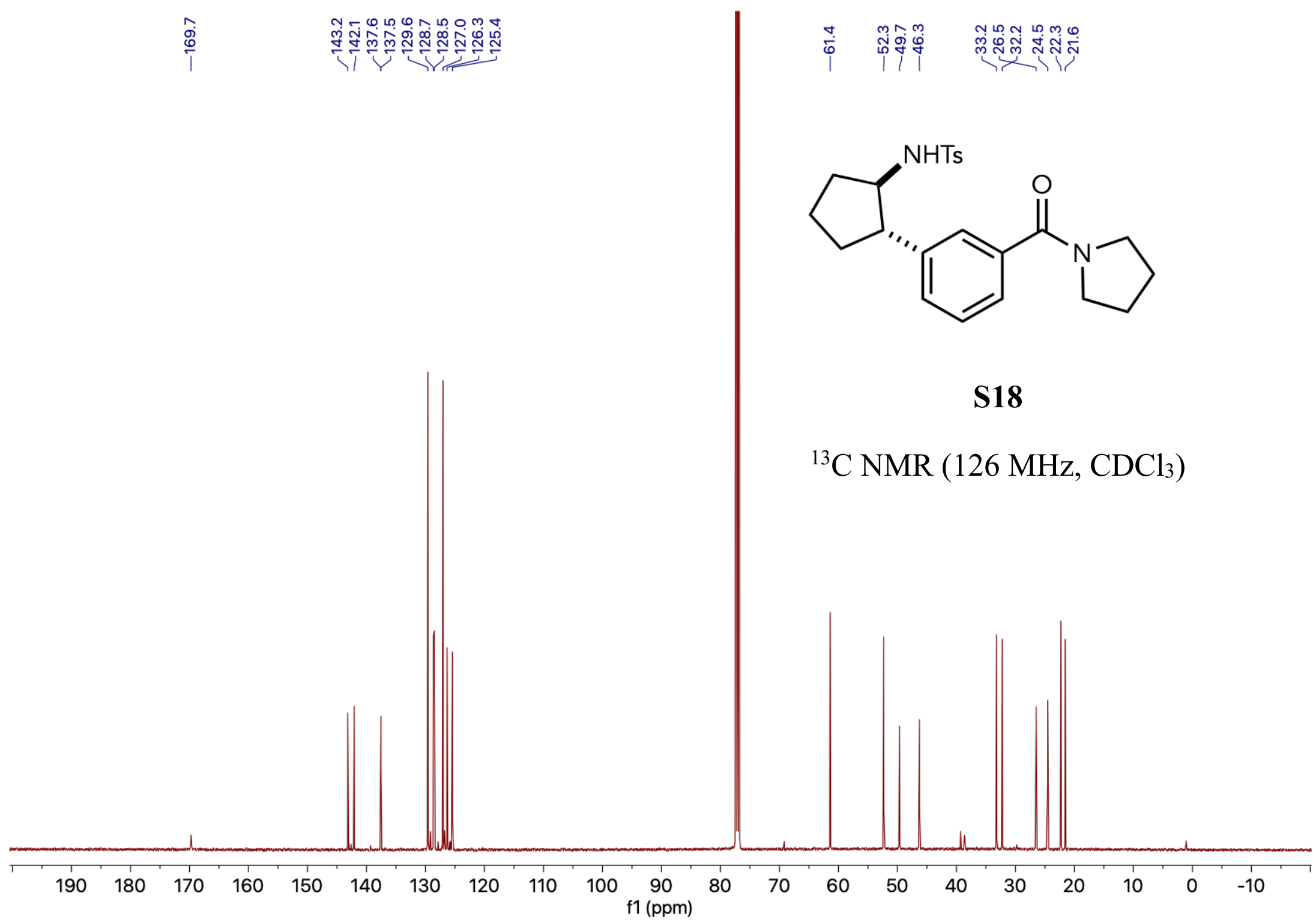

S18

^{13}C NMR (126 MHz, $CDCl_3$)

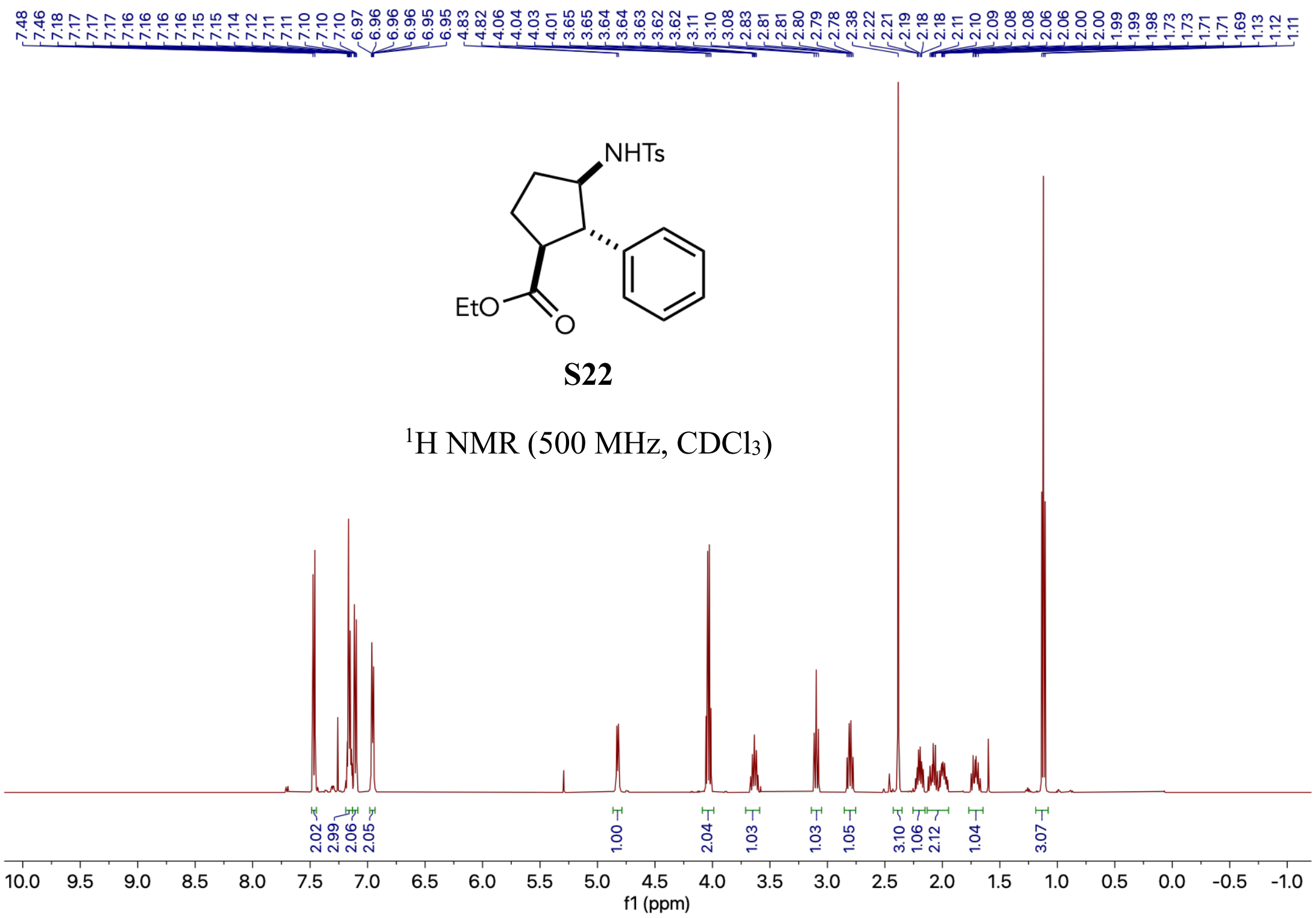

NHTs
EtO
O
S22
1H NMR (500 MHz, CDCl3)
f1 (ppm)

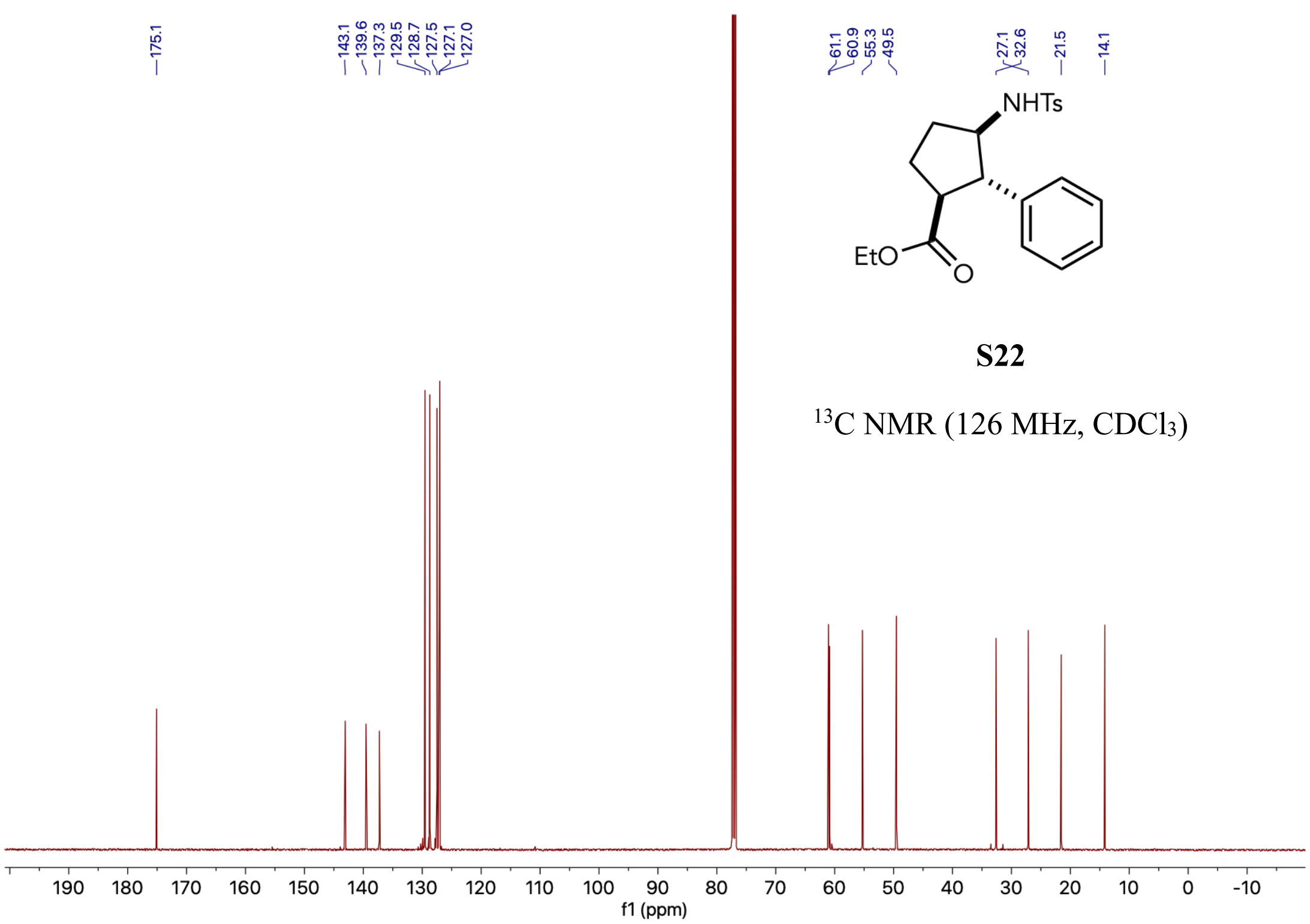

175.1
143.1
139.6
137.3
129.5
128.7
127.5
127.1
127.0
61.1
60.9
55.3
49.5
27.1
32.6
21.5
14.1
NHTs
EtO
O
S22
13C NMR (126 MHz, CDCl3)
f1 (ppm)

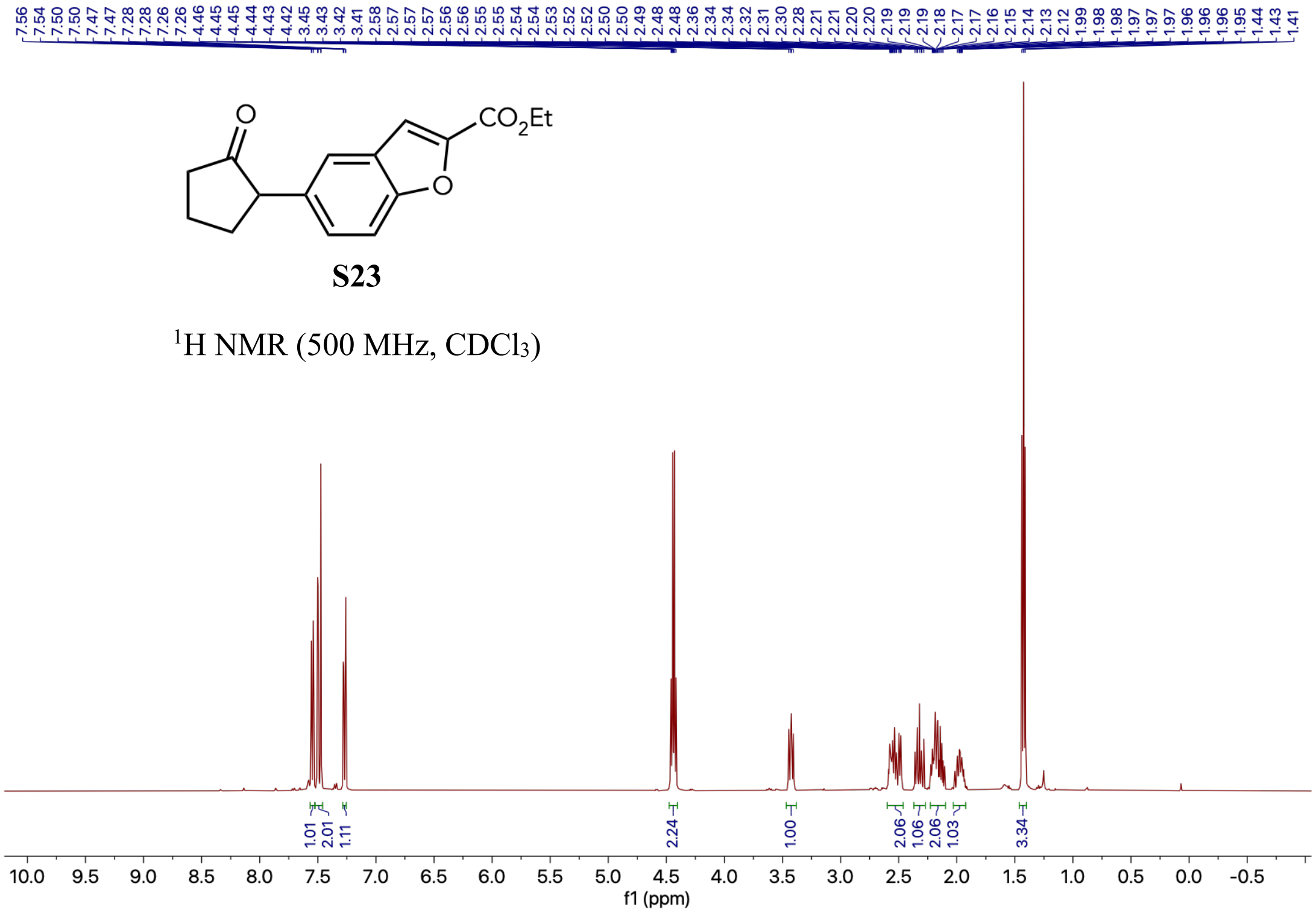

7.56 7.54 7.50 7.47 7.28 7.28 7.26 7.26 4.46 4.45 4.45 4.44 4.43 4.42 3.45 3.43 3.42 3.41 2.58 2.57 2.57 2.57 2.56 2.56 2.55 2.55 2.54 2.54 2.53 2.52 2.52 2.50 2.50 2.49 2.48 2.48 2.36 2.34 2.34 2.32 2.31 2.30 2.28 2.21 2.21 2.20 2.20 2.19 2.19 2.19 2.18 2.17 2.17 2.16 2.15 2.14 2.13 2.12 1.99 1.98 1.98 1.97 1.97 1.96 1.96 1.95 1.44 1.43 1.41
CO2Et
O
S23
1H NMR (500 MHz, CDCl3)
1.01 2.01 1.11 2.24 1.00 2.06 1.06 2.06 1.03 3.34
f1 (ppm)

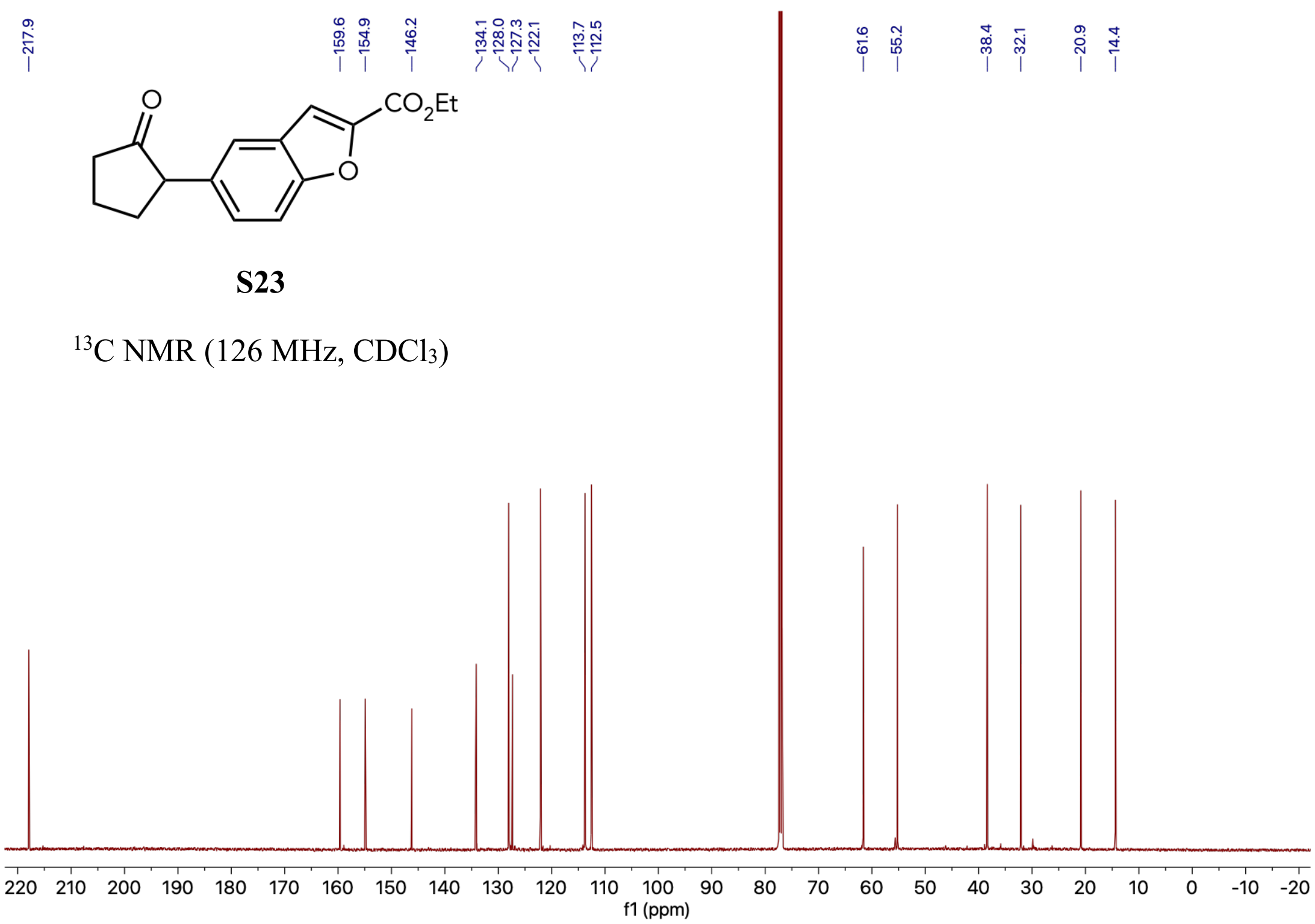

217.9 159.6 154.9 146.2 134.1 128.0 127.3 122.1 113.7 112.5 61.6 55.2 38.4 32.1 20.9 14.4
CO2Et
O
S23
13C NMR (126 MHz, CDCl3)
f1 (ppm)

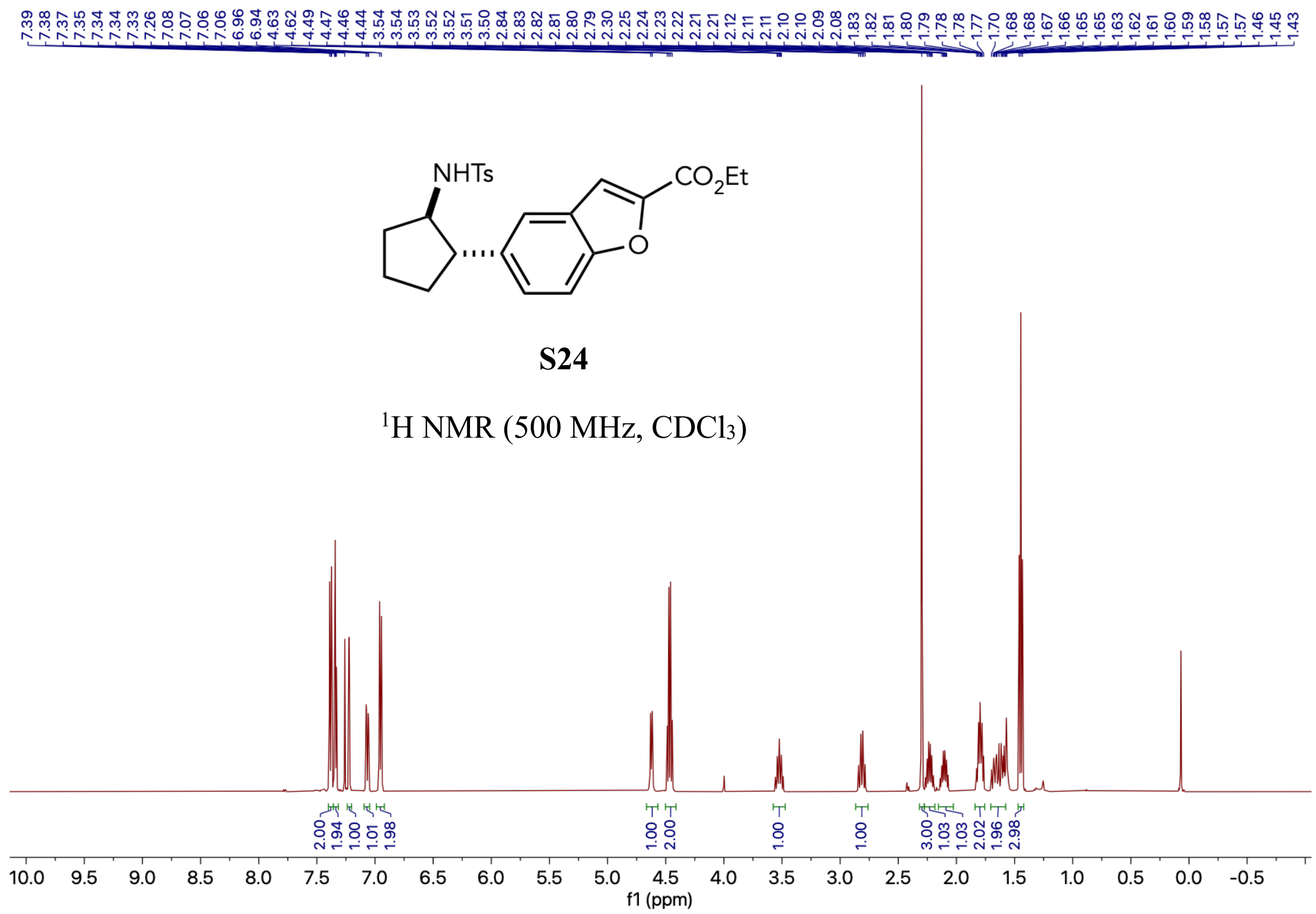
7.39 7.38 7.37 7.35 7.34 7.34 7.33 7.26 7.08 7.07 7.06 7.06 6.96 6.94 4.63 4.62 4.49 4.47 4.46 4.44 3.54 3.54 3.53 3.53 3.52 3.52 3.51 3.50 2.84 2.83 2.82 2.81 2.80 2.79 2.30 2.25 2.24 2.23 2.22 2.21 2.21 2.12 2.11 2.11 2.10 2.10 2.09 2.08 1.83 1.82 1.81 1.80 1.79 1.78 1.78 1.77 1.70 1.68 1.68 1.67 1.66 1.65 1.65 1.63 1.62 1.61 1.60 1.59 1.58 1.57 1.57 1.46 1.45 1.43
NHTs
CO2Et
O
S24
1H NMR (500 MHz, CDCl3)
2.00 1.94 1.00 1.01 1.98 1.00 2.00 1.00 1.00 3.00 1.03 1.03 2.02 1.96 2.98
10.0 9.5 9.0 8.5 8.0 7.5 7.0 6.5 6.0 5.5 5.0 4.5 4.0 3.5 3.0 2.5 2.0 1.5 1.0 0.5 0.0 -0.5
f1 (ppm)

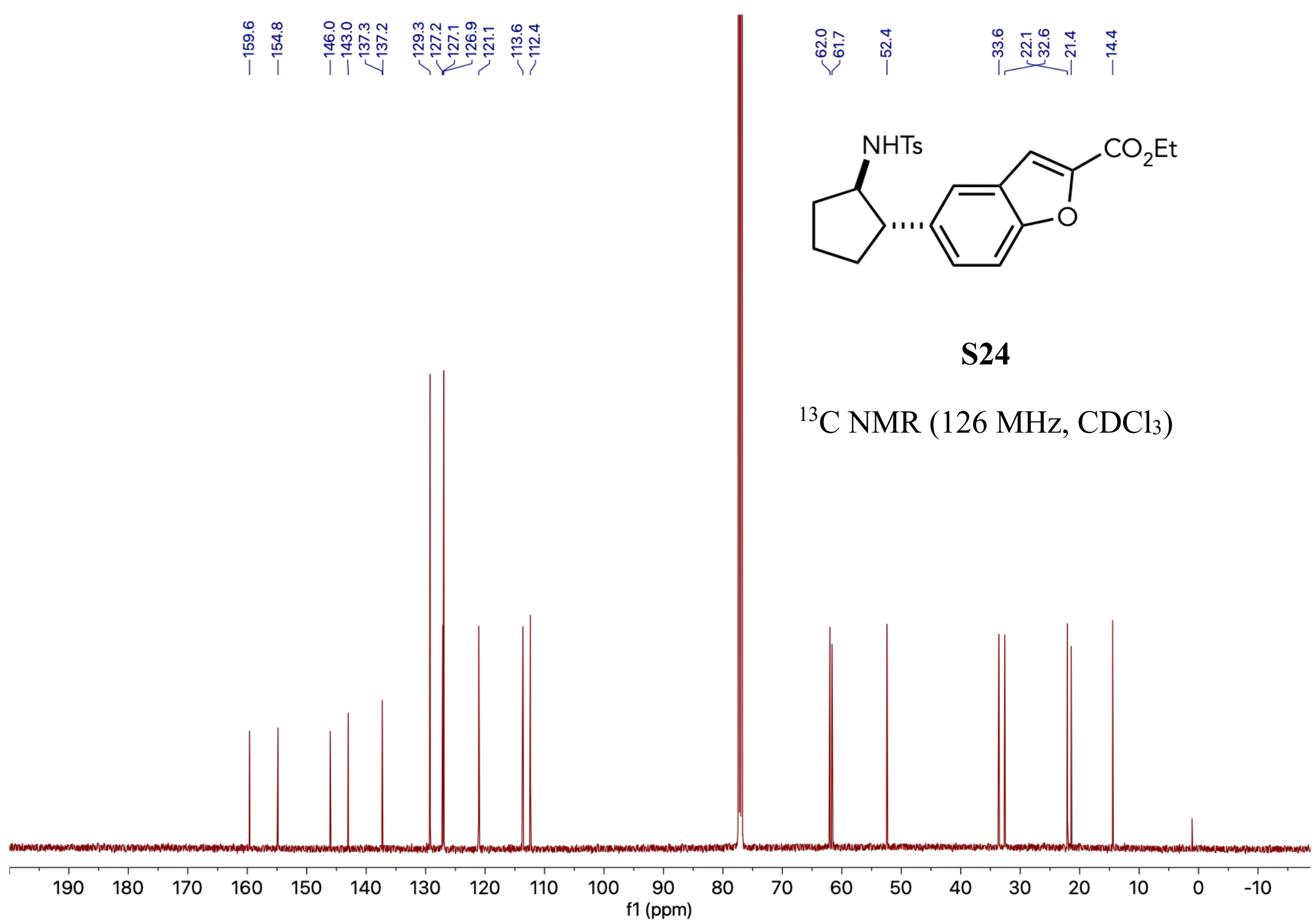
159.6 154.8 146.0 143.0 137.3 137.2 129.3 127.2 127.1 126.9 121.1 113.6 112.4 62.0 61.7 52.4 33.6 22.1 32.6 21.4 14.4
NHTs
CO2Et
O
S24
13C NMR (126 MHz, CDCl3)
190 180 170 160 150 140 130 120 110 100 90 80 70 60 50 40 30 20 10 0 -10
f1 (ppm)

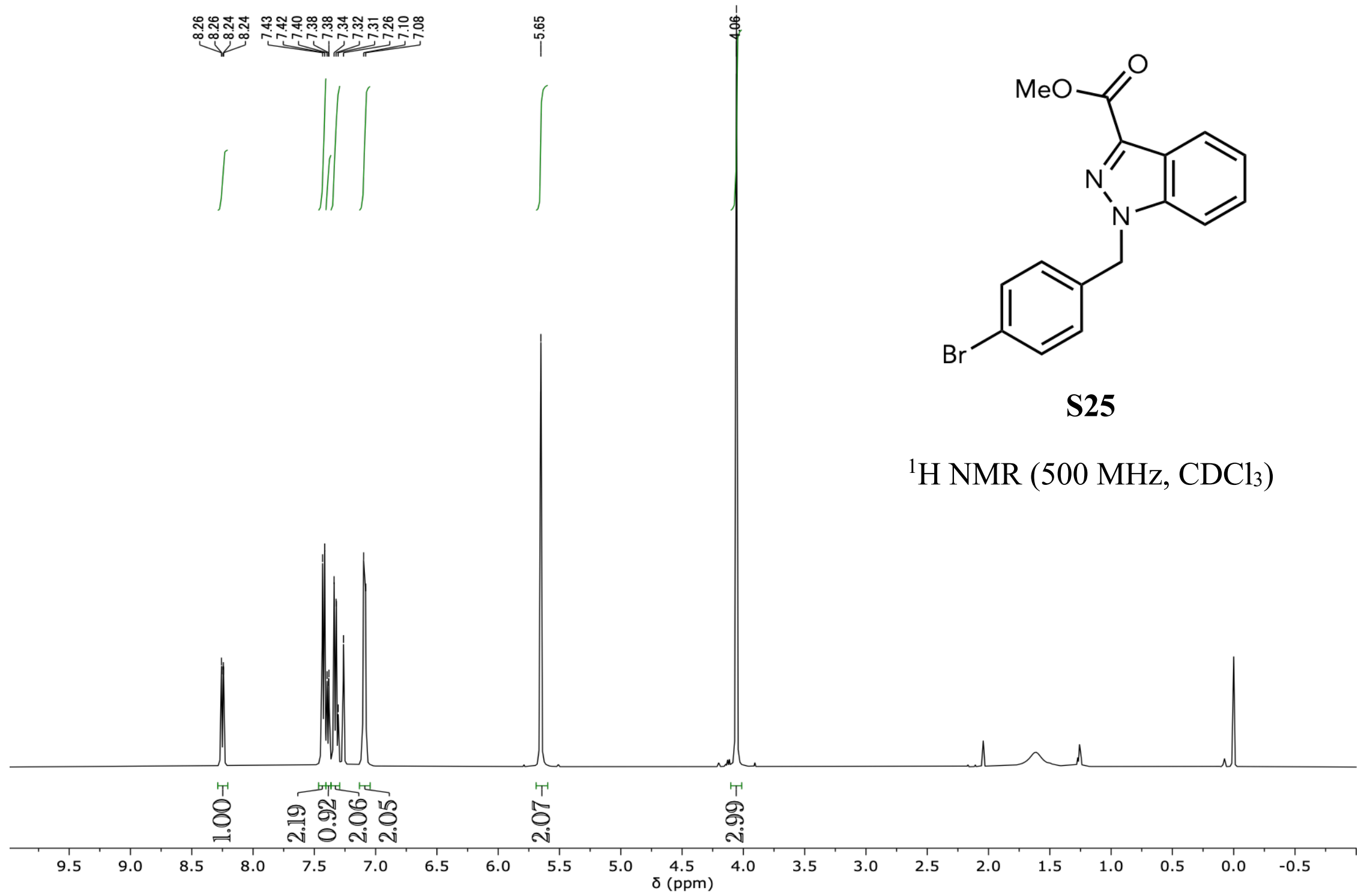

S25

^{1}H NMR (500 MHz, $CDCl_3$)

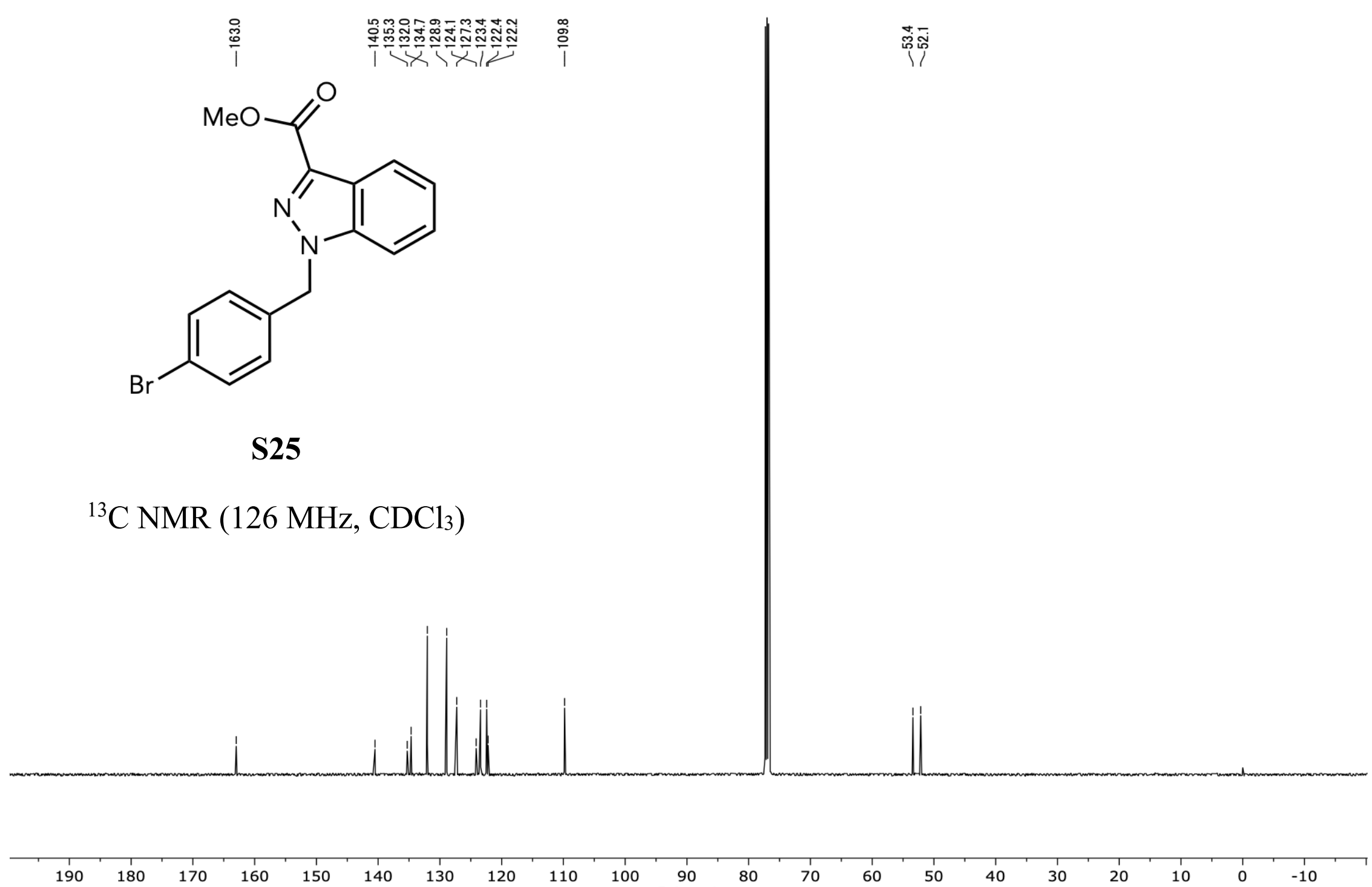

S25

^{13}C NMR (126 MHz, $CDCl_3$)

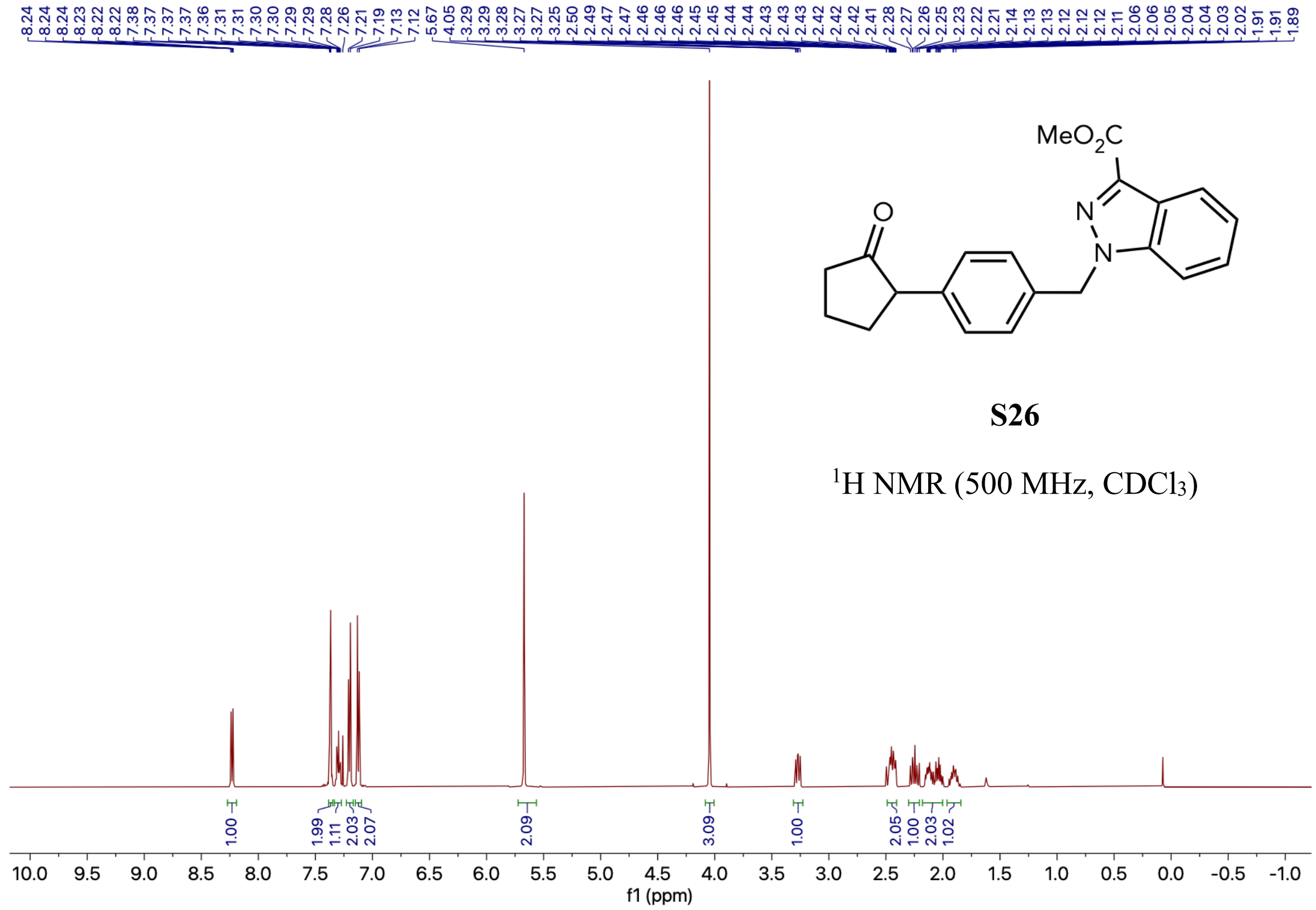

S26

^{1}H NMR (500 MHz, $CDCl_3$)

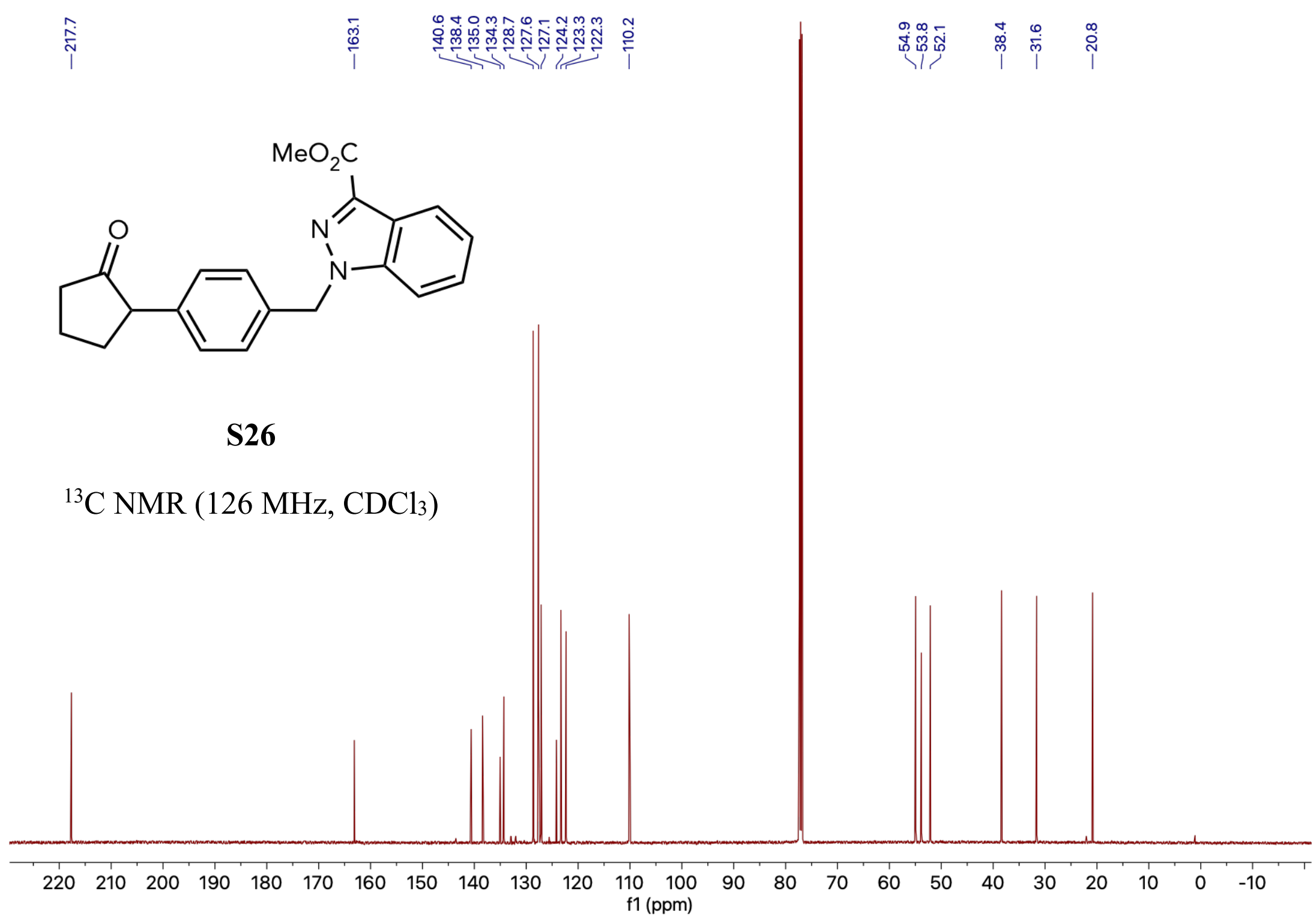

S26

^{13}C NMR (126 MHz, $CDCl_3$)

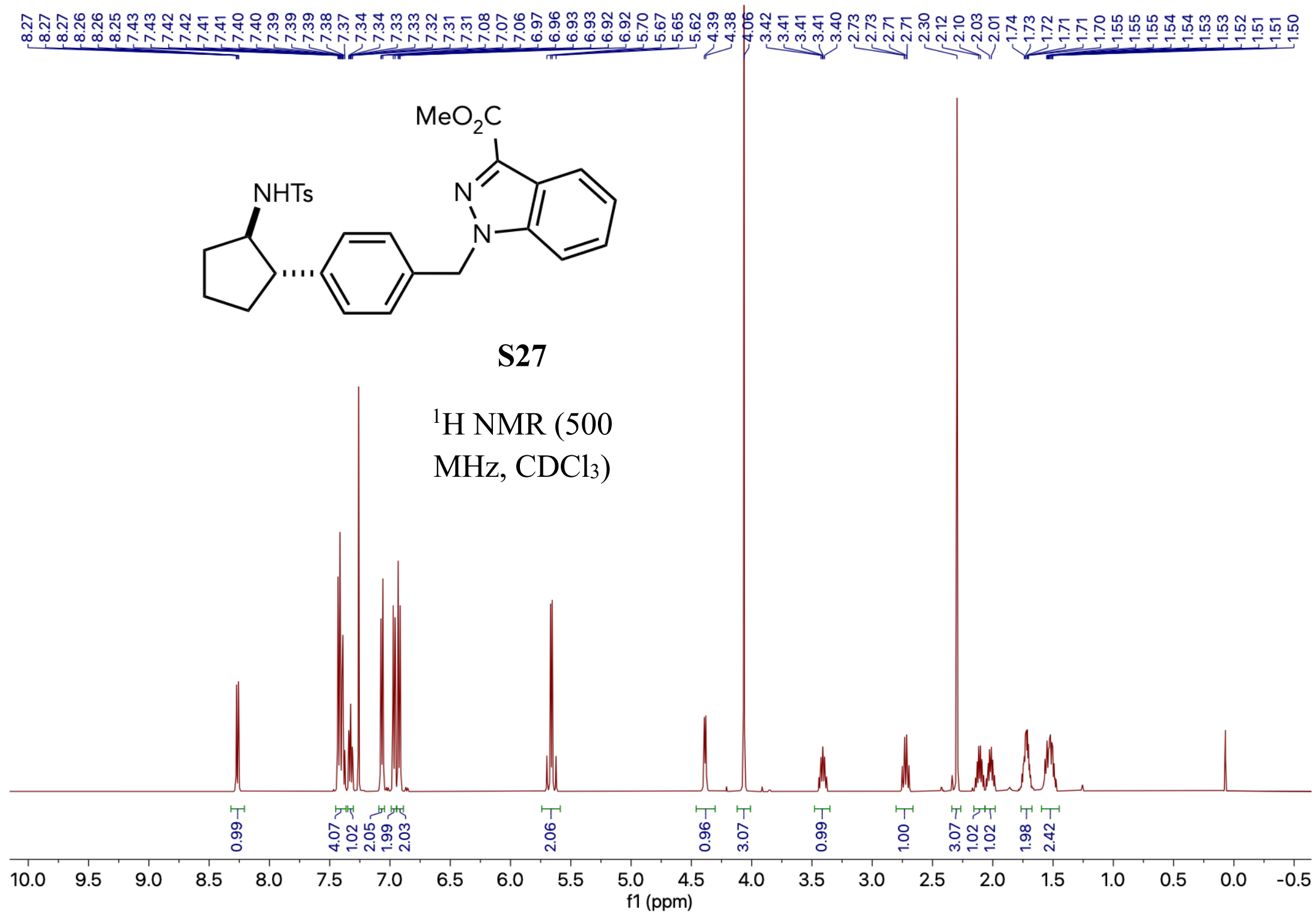
8.27
8.27
8.26
8.26
8.25
7.43
7.43
7.42
7.42
7.41
7.40
7.40
7.39
7.39
7.39
7.38
7.37
7.34
7.34
7.33
7.33
7.32
7.31
7.31
7.08
7.07
7.06
6.97
6.96
6.93
6.93
6.92
6.92
5.70
5.67
5.65
5.62
4.39
4.38
4.06
3.42
3.41
3.41
3.41
3.40
2.73
2.73
2.71
2.71
2.30
2.12
2.10
2.03
2.01
1.74
1.73
1.72
1.71
1.71
1.70
1.55
1.55
1.55
1.54
1.54
1.53
1.53
1.52
1.51
1.51
1.50
MeO2C
NHTs
N
N
S27
1H NMR (500 MHz, CDCl3)
0.99
4.07
1.02
2.05
1.99
2.03
2.06
0.96
3.07
0.99
1.00
3.07
1.02
1.02
1.98
2.42
f1 (ppm)

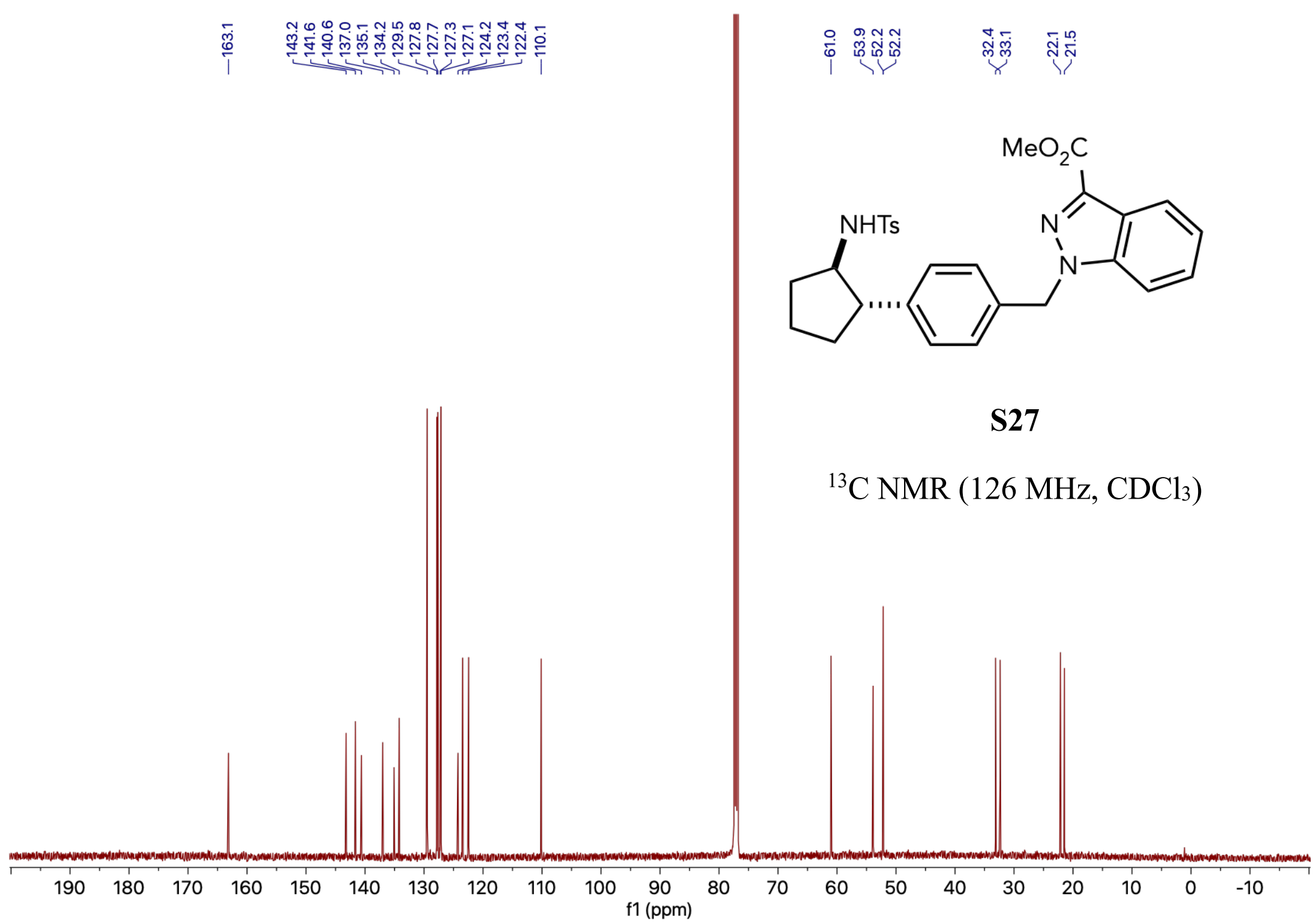
163.1
143.2
141.6
140.6
137.0
135.1
134.2
129.5
127.8
127.7
127.3
127.1
124.2
123.4
122.4
110.1
61.0
53.9
52.2
52.2
32.4
33.1
22.1
21.5
MeO2C
NHTs
N
N
S27
13C NMR (126 MHz, CDCl3)
f1 (ppm)

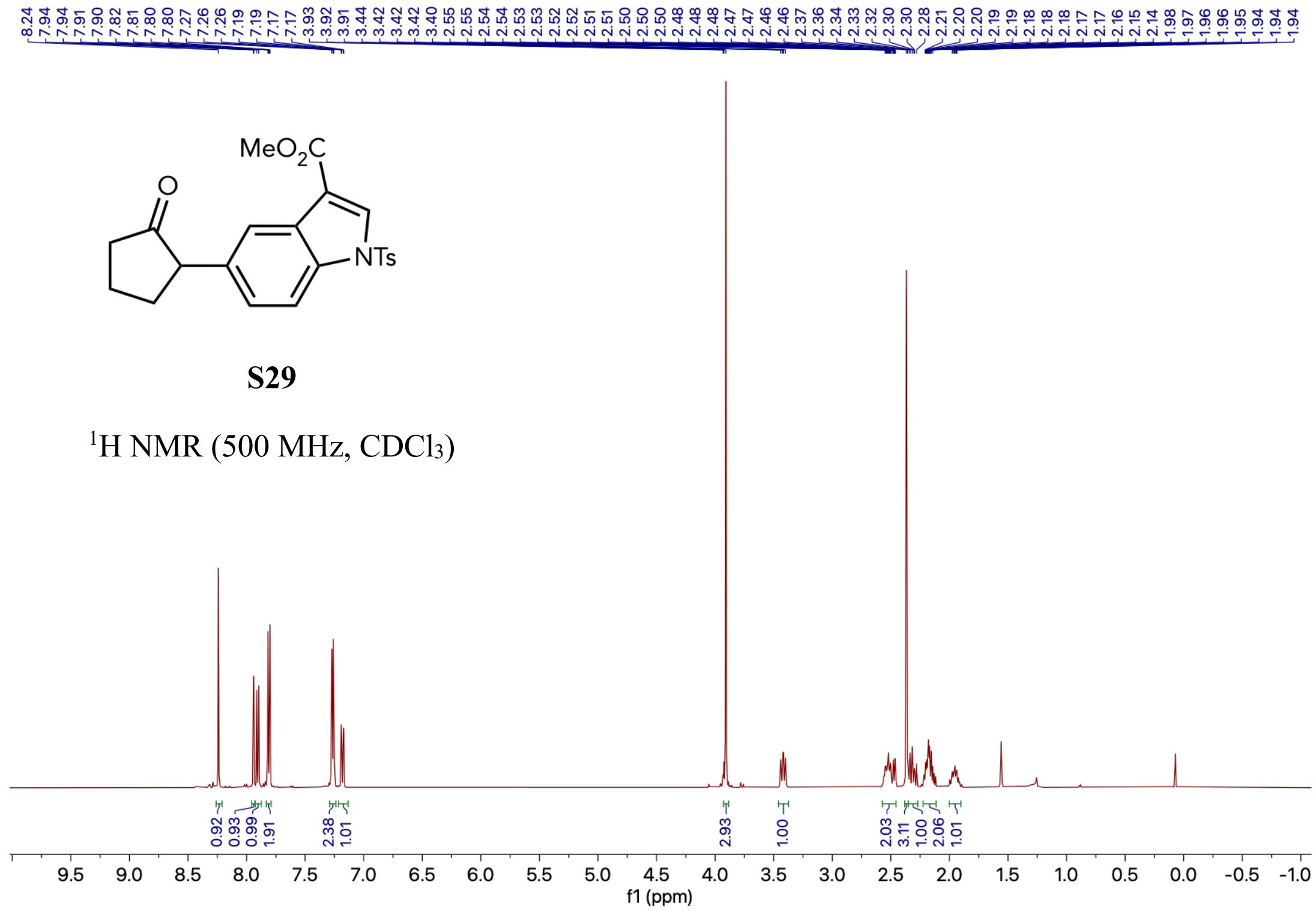
8.24 7.94 7.94 7.91 7.90 7.82 7.81 7.80 7.80 7.27 7.26 7.26 7.19 7.19 7.17 7.17 3.93 3.92 3.91 3.44 3.42 3.42 3.42 3.40 2.55 2.55 2.54 2.54 2.53 2.53 2.52 2.52 2.51 2.51 2.50 2.50 2.50 2.48 2.48 2.48 2.47 2.47 2.46 2.46 2.37 2.36 2.34 2.33 2.32 2.30 2.30 2.28 2.21 2.20 2.20 2.19 2.19 2.18 2.18 2.18 2.17 2.17 2.16 2.15 2.14 1.98 1.97 1.96 1.96 1.95 1.94 1.94 1.94
MeO2C
O
NTs
S29
1H NMR (500 MHz, CDCl3)
0.92 0.93 0.99 1.91 2.38 1.01 2.93 1.00 2.03 3.11 1.00 2.06 1.01
f1 (ppm)

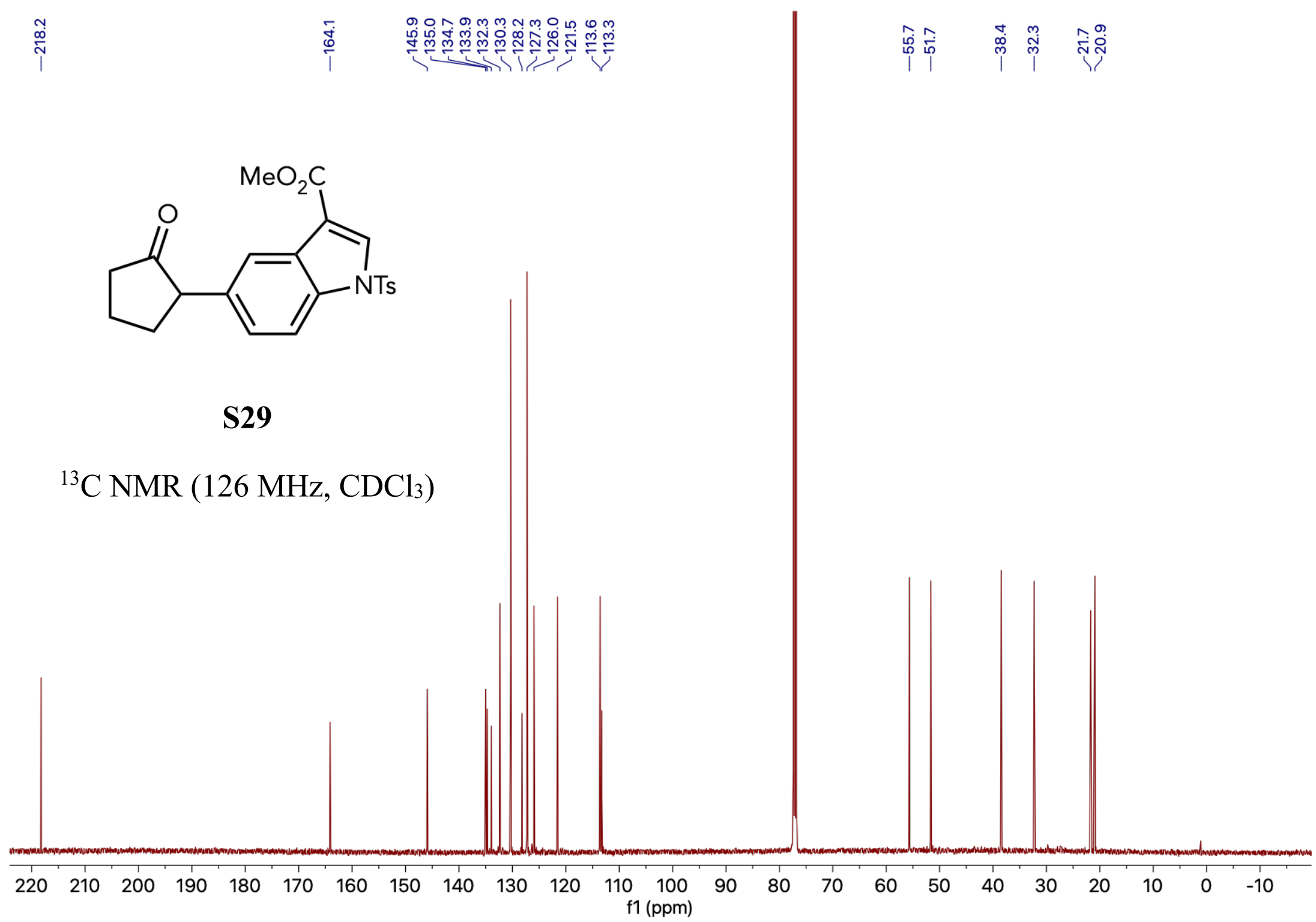
218.2 164.1 145.9 135.0 134.7 133.9 132.3 130.3 128.2 127.3 126.0 121.5 113.6 113.3 55.7 51.7 38.4 32.3 21.7 20.9
MeO2C
O
NTs
S29
13C NMR (126 MHz, CDCl3)
f1 (ppm)

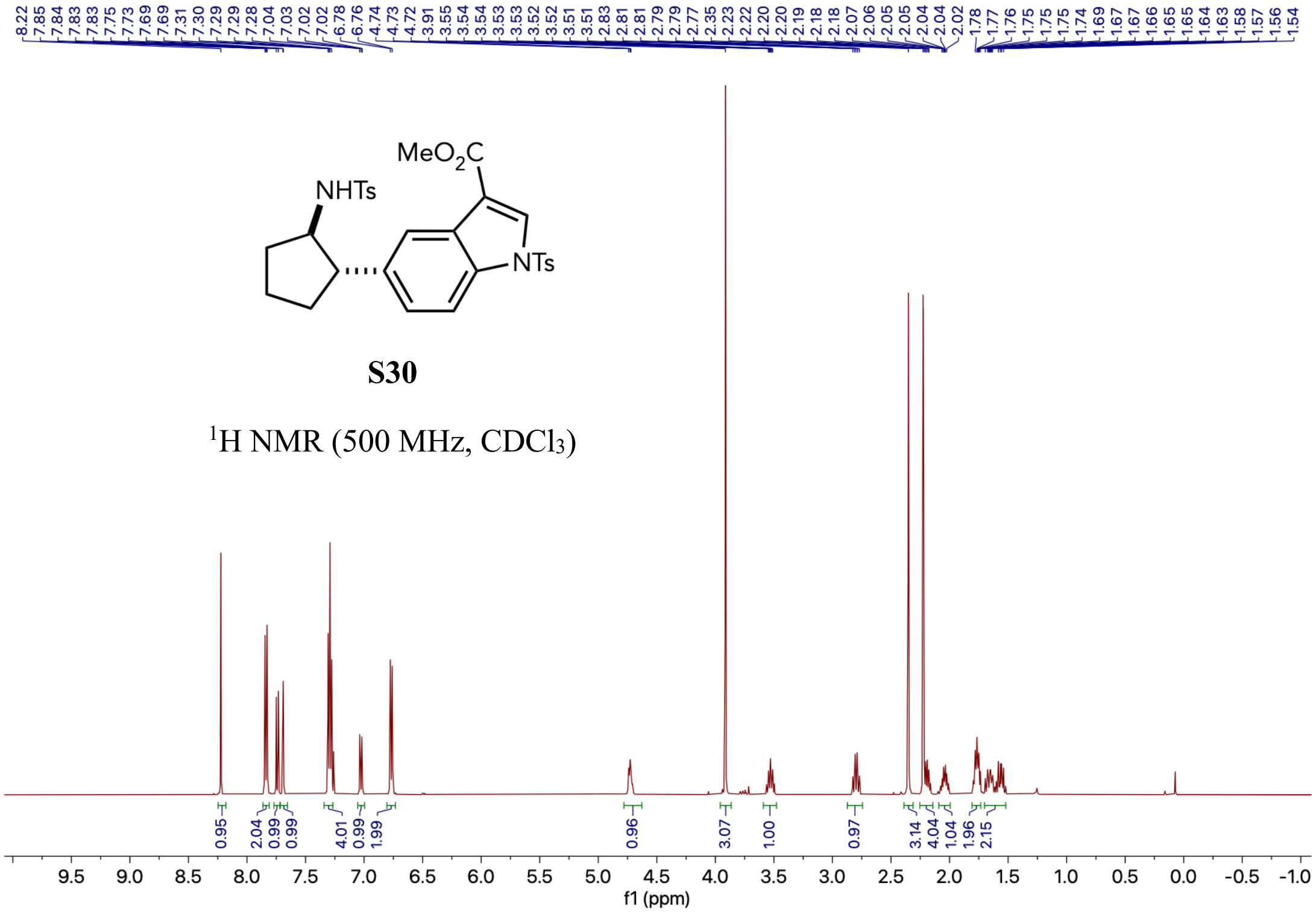

8.22 7.85 7.84 7.83 7.75 7.73 7.69 7.69 7.31 7.30 7.29 7.29 7.28 7.04 7.03 7.02 7.02 6.78 6.76 4.74 4.73 4.72 3.91 3.55 3.54 3.54 3.53 3.53 3.52 3.52 3.51 3.51 2.83 2.81 2.81 2.79 2.79 2.77 2.35 2.23 2.22 2.20 2.20 2.19 2.18 2.18 2.07 2.06 2.05 2.05 2.04 2.04 2.02 1.78 1.77 1.76 1.75 1.75 1.75 1.74 1.69 1.67 1.66 1.65 1.64 1.63 1.58 1.57 1.56 1.54
MeO2C
NHTs
NTs
S30
1H NMR (500 MHz, CDCl3)
0.95 2.04 0.99 0.99 4.01 0.99 1.99 0.96 3.07 1.00 0.97 3.14 4.04 1.04 1.96 2.15
f1 (ppm)

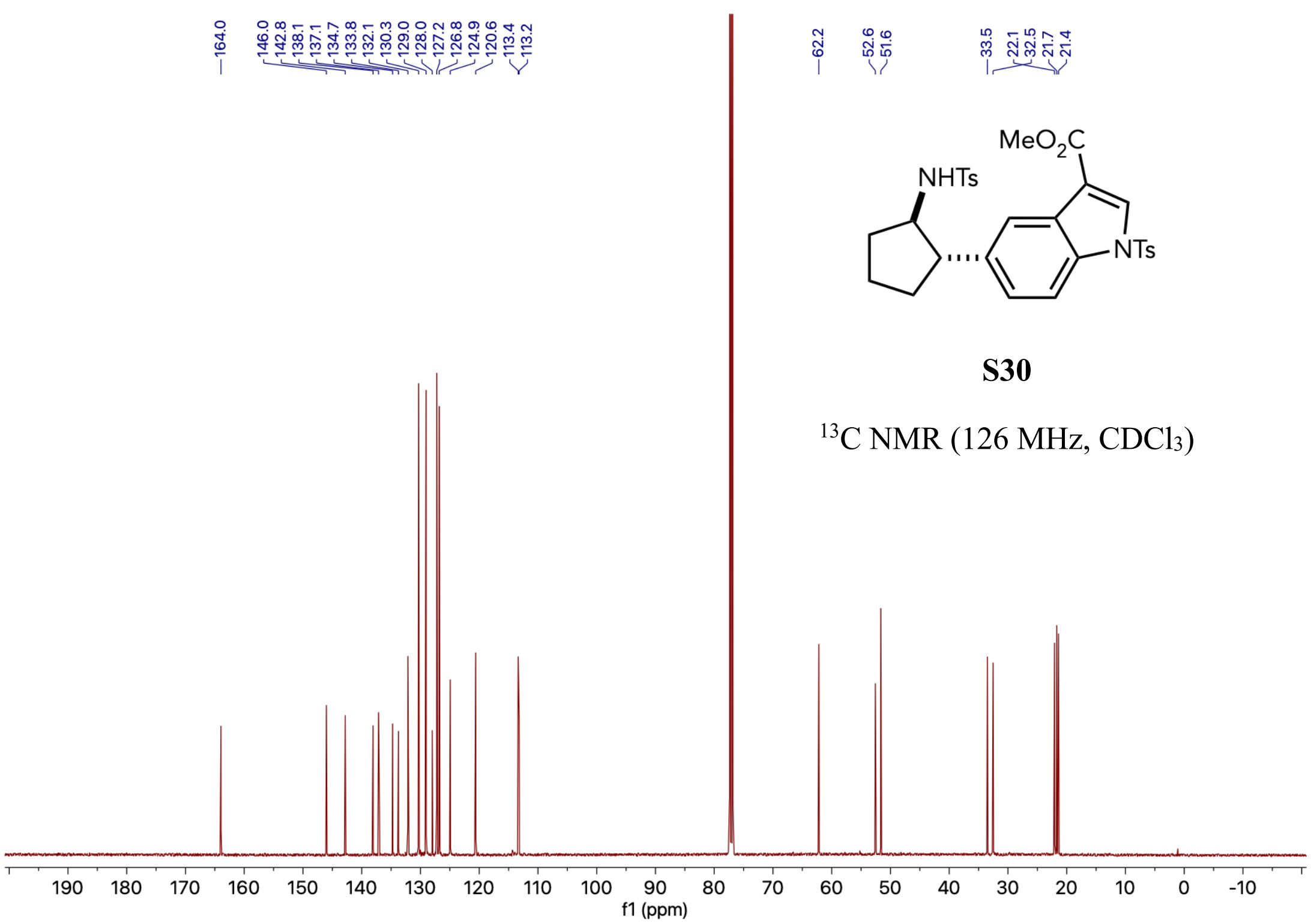

164.0 146.0 142.8 138.1 137.1 134.7 133.8 132.1 130.3 129.0 128.0 127.2 126.8 124.9 120.6 113.4 113.2 62.2 52.6 51.6 33.5 22.1 32.5 21.7 21.4
MeO2C
NHTs
NTs
S30
13C NMR (126 MHz, CDCl3)
f1 (ppm)

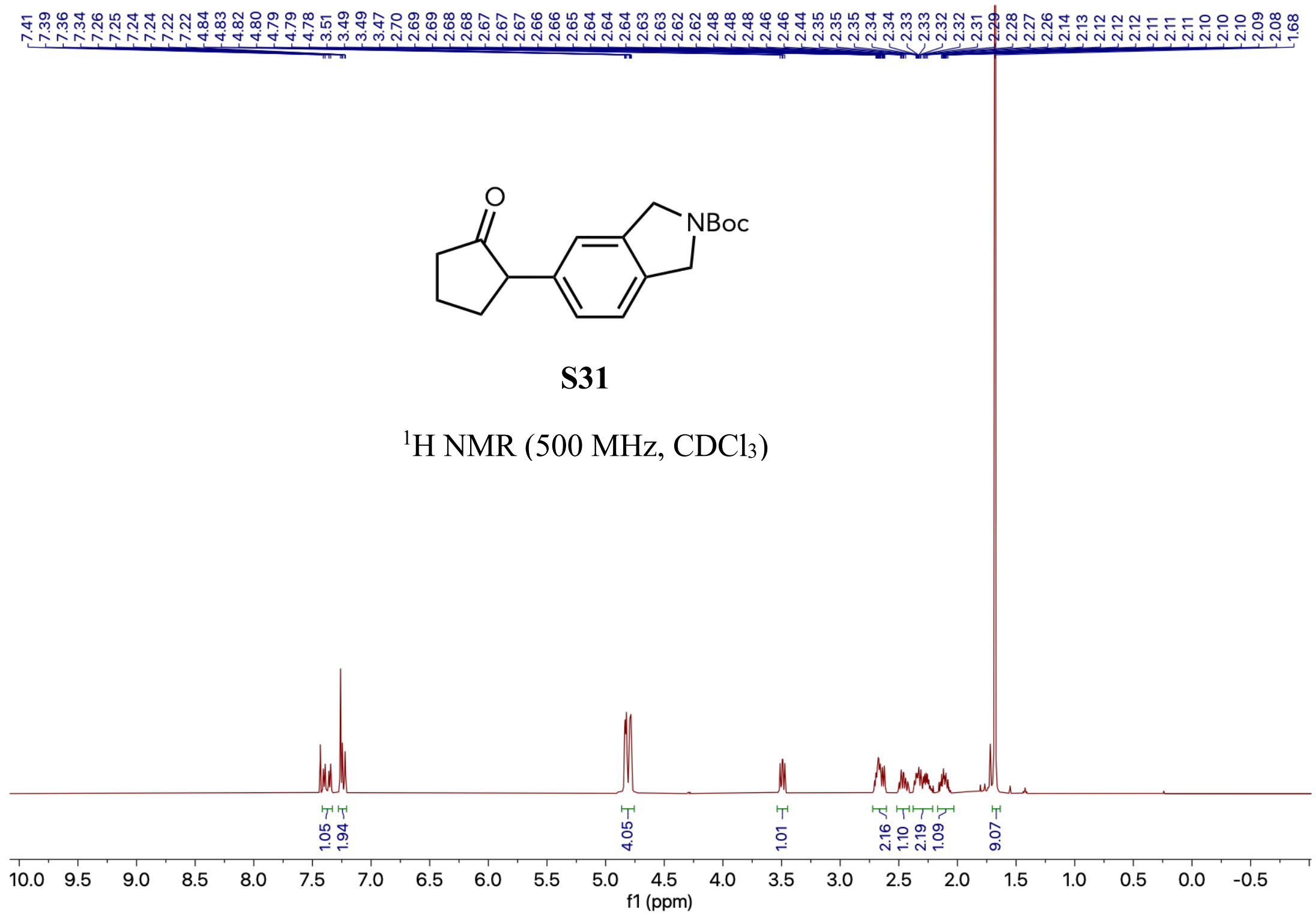

S31

^{1}H NMR (500 MHz, $CDCl_3$)

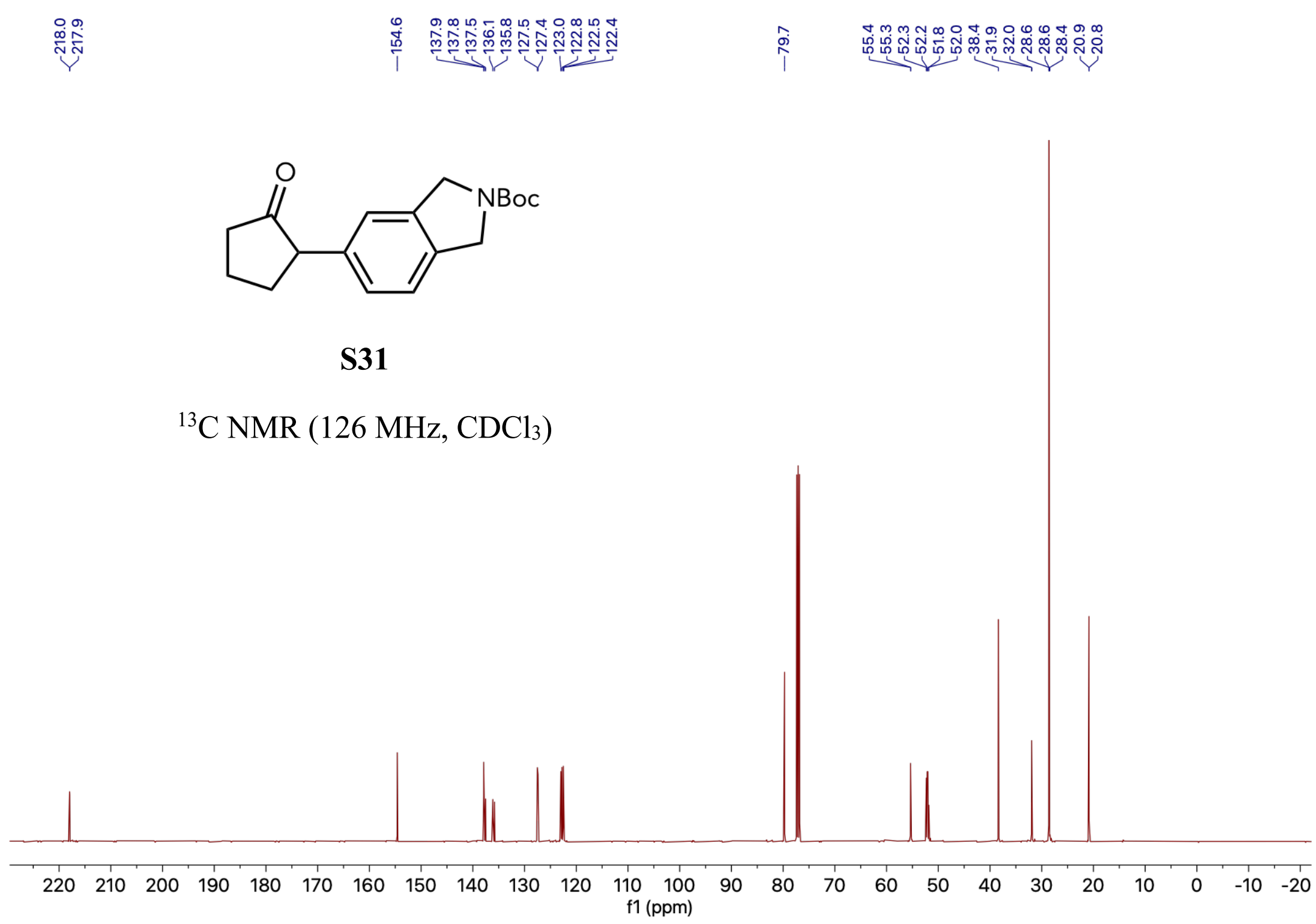

S31

^{13}C NMR (126 MHz, $CDCl_3$)

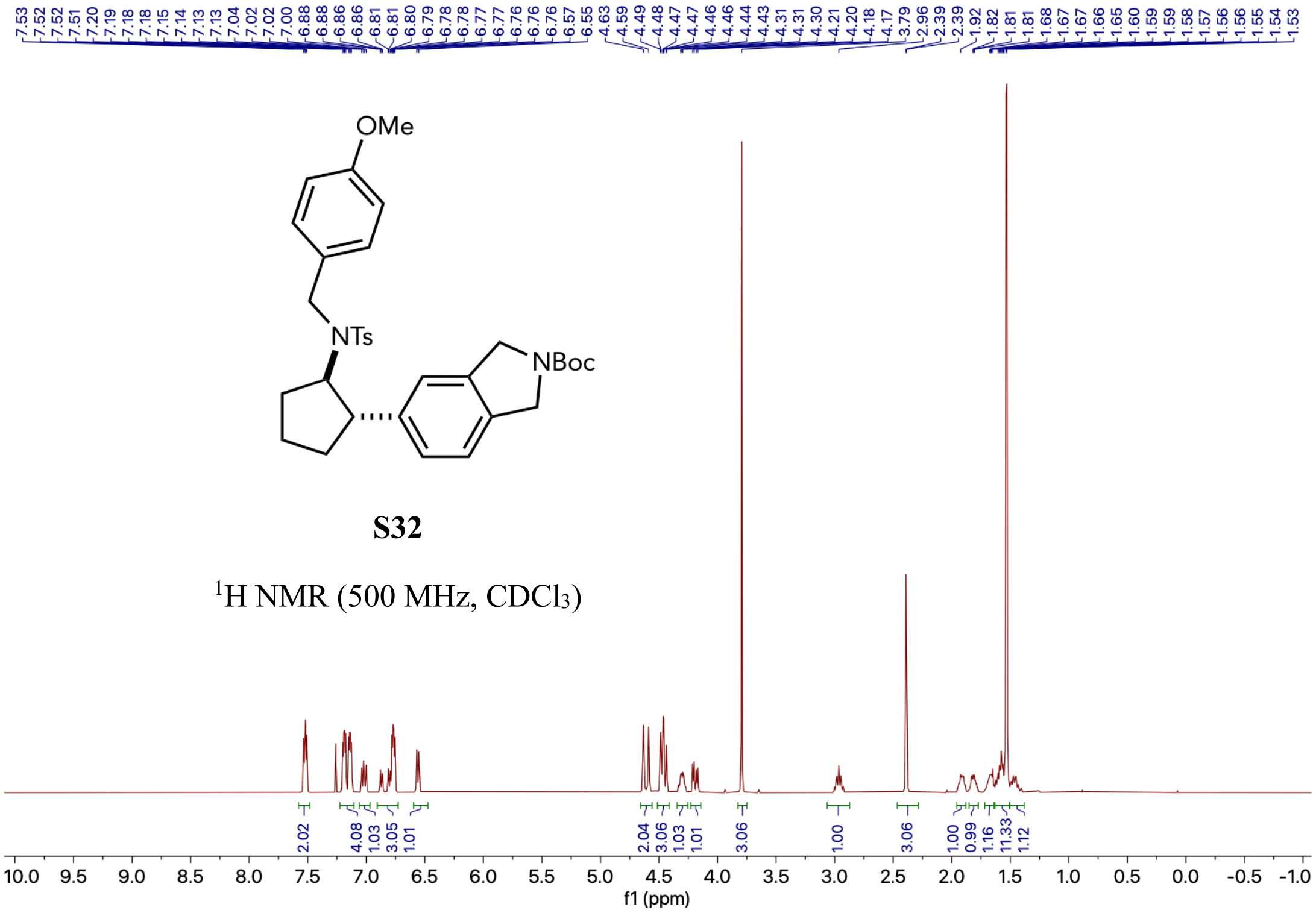

S32

^{1}H NMR (500 MHz, $CDCl_3$)

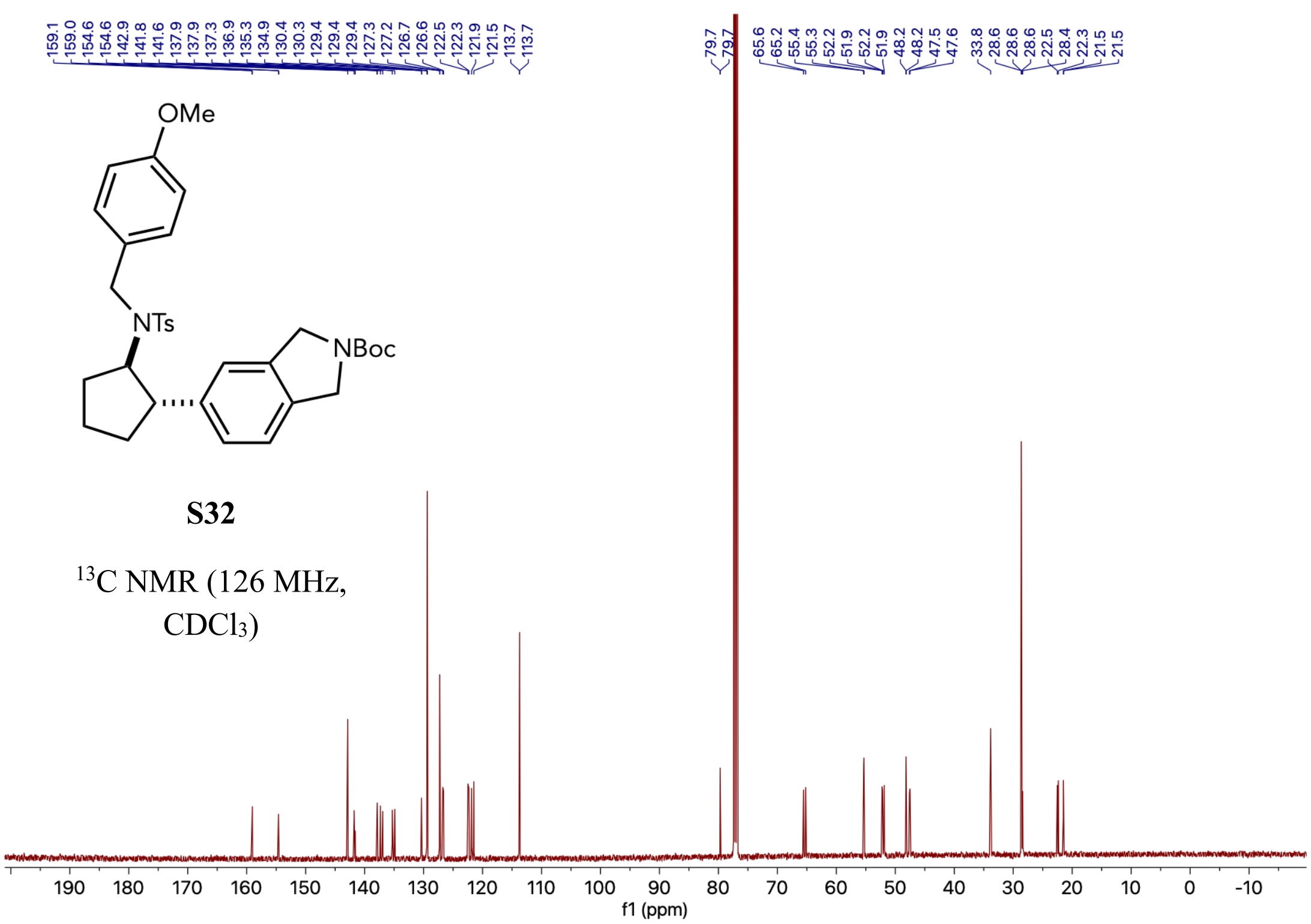

S32

^{13}C NMR (126 MHz, $CDCl_3$)

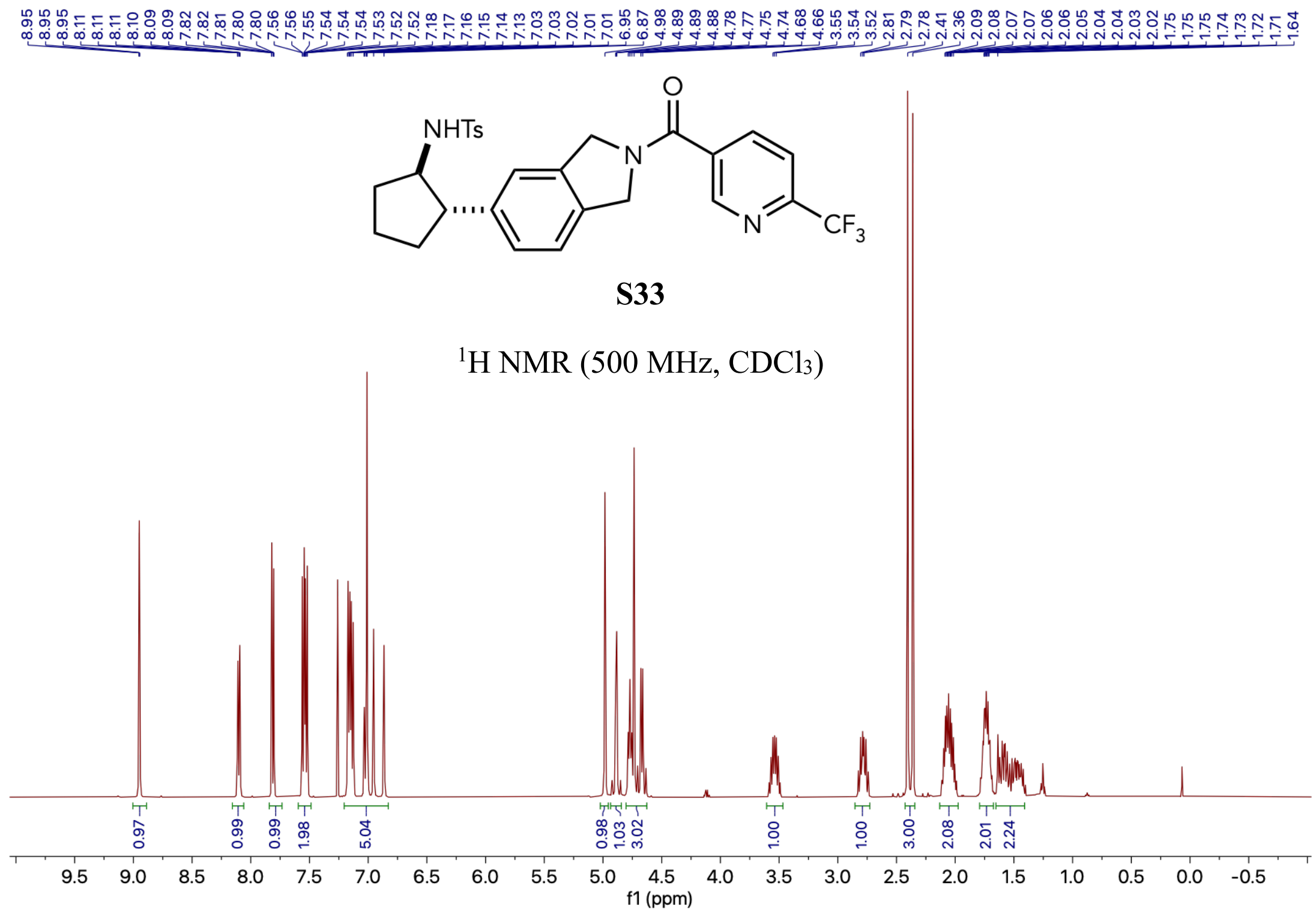

8.95 8.95 8.95 8.11 8.11 8.10 8.09 8.09 7.82 7.82 7.81 7.80 7.80 7.56 7.56 7.55 7.54 7.54 7.54 7.53 7.52 7.52 7.18 7.17 7.16 7.15 7.14 7.13 7.03 7.03 7.02 7.01 7.01 6.95 6.87 4.98 4.89 4.89 4.88 4.78 4.77 4.75 4.74 4.68 4.66 3.55 3.54 3.52 2.81 2.79 2.78 2.41 2.36 2.09 2.08 2.07 2.06 2.06 2.05 2.04 2.04 2.03 2.02 1.75 1.75 1.74 1.73 1.72 1.71 1.64
NHTs
O
N
N
CF3
S33
1H NMR (500 MHz, CDCl3)
0.97 0.99 0.99 1.98 5.04 0.98 1.03 3.02 1.00 1.00 3.00 2.08 2.01 2.24
9.5 9.0 8.5 8.0 7.5 7.0 6.5 6.0 5.5 5.0 4.5 4.0 3.5 3.0 2.5 2.0 1.5 1.0 0.5 0.0 -0.5
f1 (ppm)

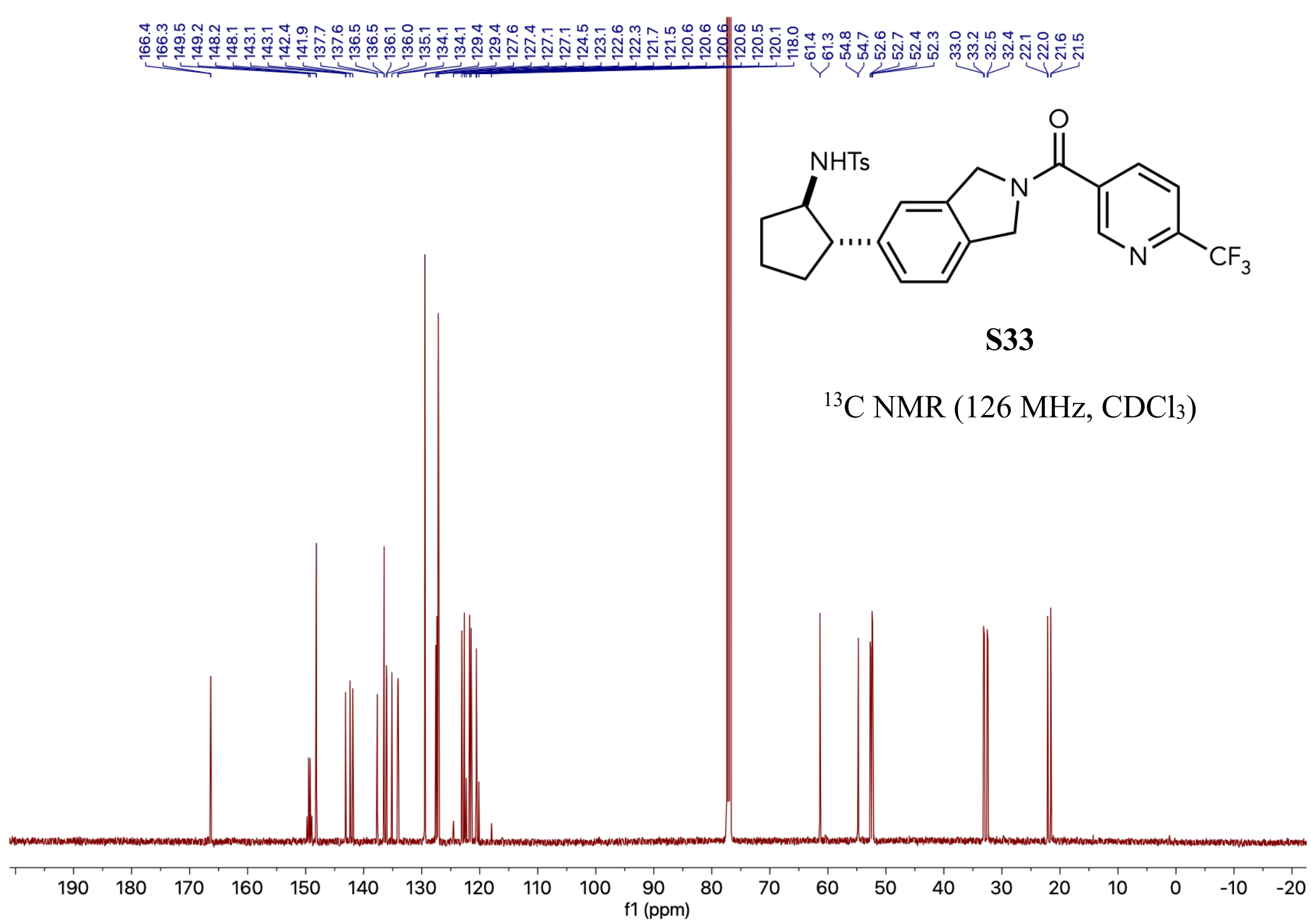

166.4 166.3 149.5 149.2 148.2 148.1 143.1 143.1 142.4 141.9 137.7 137.6 136.5 136.1 136.0 135.1 134.1 134.1 129.4 129.4 127.6 127.4 127.1 127.1 124.5 123.1 122.6 122.3 121.7 121.5 120.6 120.6 120.6 120.5 120.1 118.0 61.4 61.3 54.8 54.7 52.6 52.7 52.4 52.3 33.0 33.2 32.5 32.4 22.1 22.0 21.6 21.5
NHTs
O
N
N
CF3
S33
13C NMR (126 MHz, CDCl3)
190 180 170 160 150 140 130 120 110 100 90 80 70 60 50 40 30 20 10 0 -10 -20
f1 (ppm)

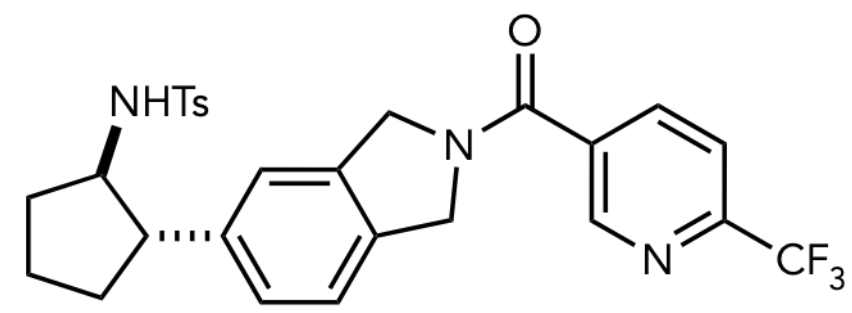

S33

^{19}F NMR (377 MHz, $CDCl_3$)

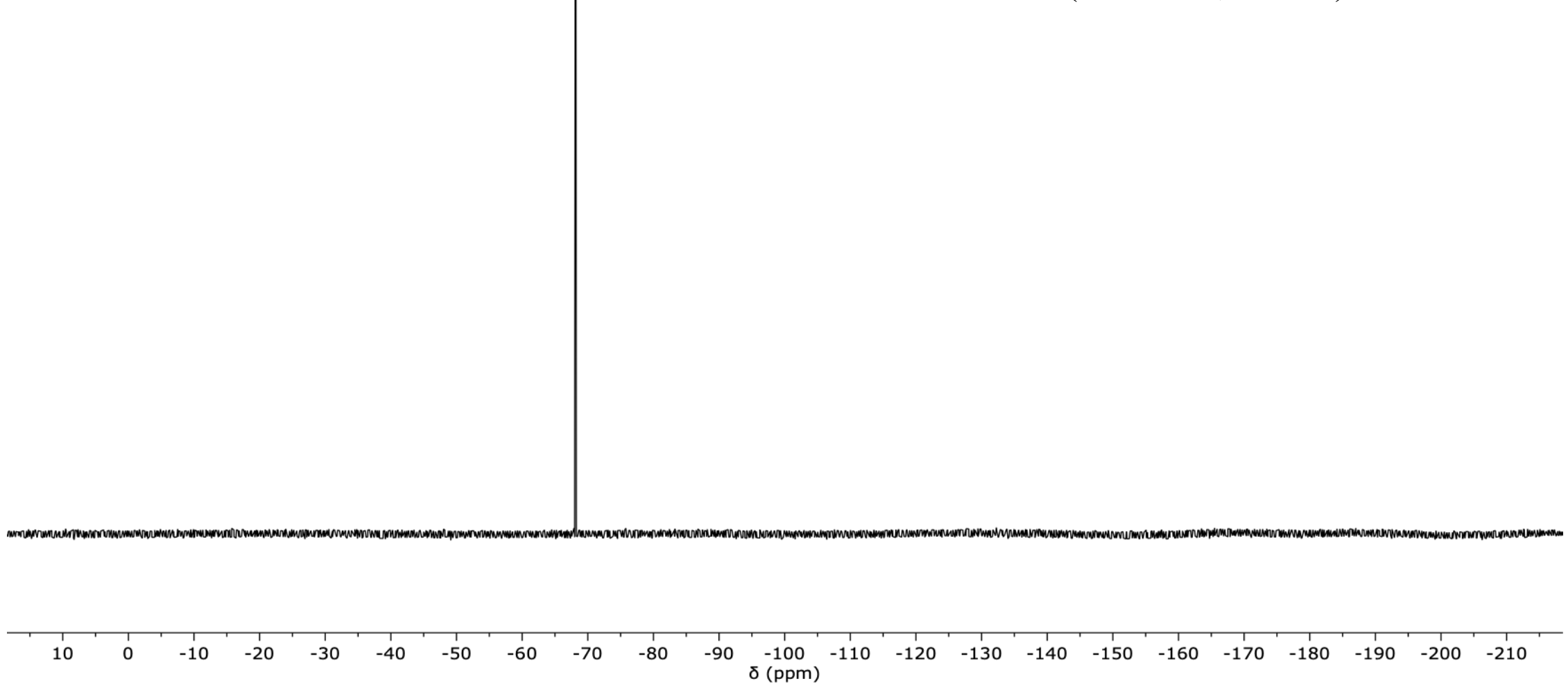

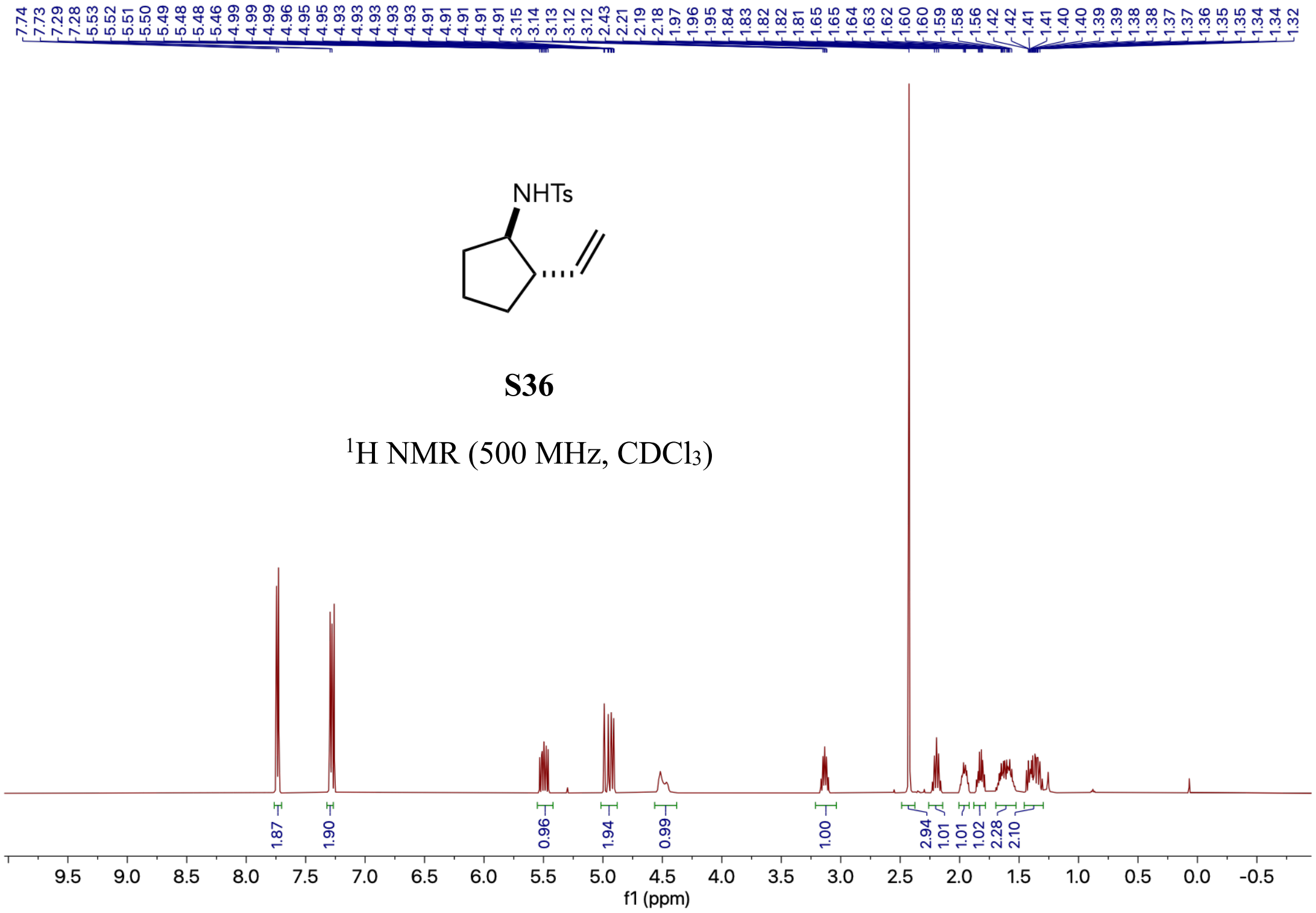
7.74
7.73
7.29
7.28
5.53
5.52
5.51
5.50
5.49
5.48
5.46
4.99
4.99
4.99
4.96
4.95
4.95
4.93
4.93
4.93
4.93
4.93
4.91
4.91
4.91
4.91
4.91
3.15
3.14
3.13
3.12
3.12
2.43
2.21
2.19
2.18
1.97
1.96
1.95
1.84
1.83
1.82
1.82
1.81
1.65
1.65
1.64
1.63
1.62
1.60
1.60
1.59
1.58
1.56
1.42
1.42
1.41
1.41
1.40
1.40
1.39
1.39
1.38
1.38
1.37
1.37
1.36
1.35
1.35
1.34
1.34
1.32
NHTs
S36
^{1}H NMR (500 MHz, CDCl$_3$)
1.87
1.90
0.96
1.94
0.99
1.00
2.94
1.01
1.01
1.02
2.28
2.10
9.5 9.0 8.5 8.0 7.5 7.0 6.5 6.0 5.5 5.0 4.5 4.0 3.5 3.0 2.5 2.0 1.5 1.0 0.5 0.0 -0.5
f1 (ppm)

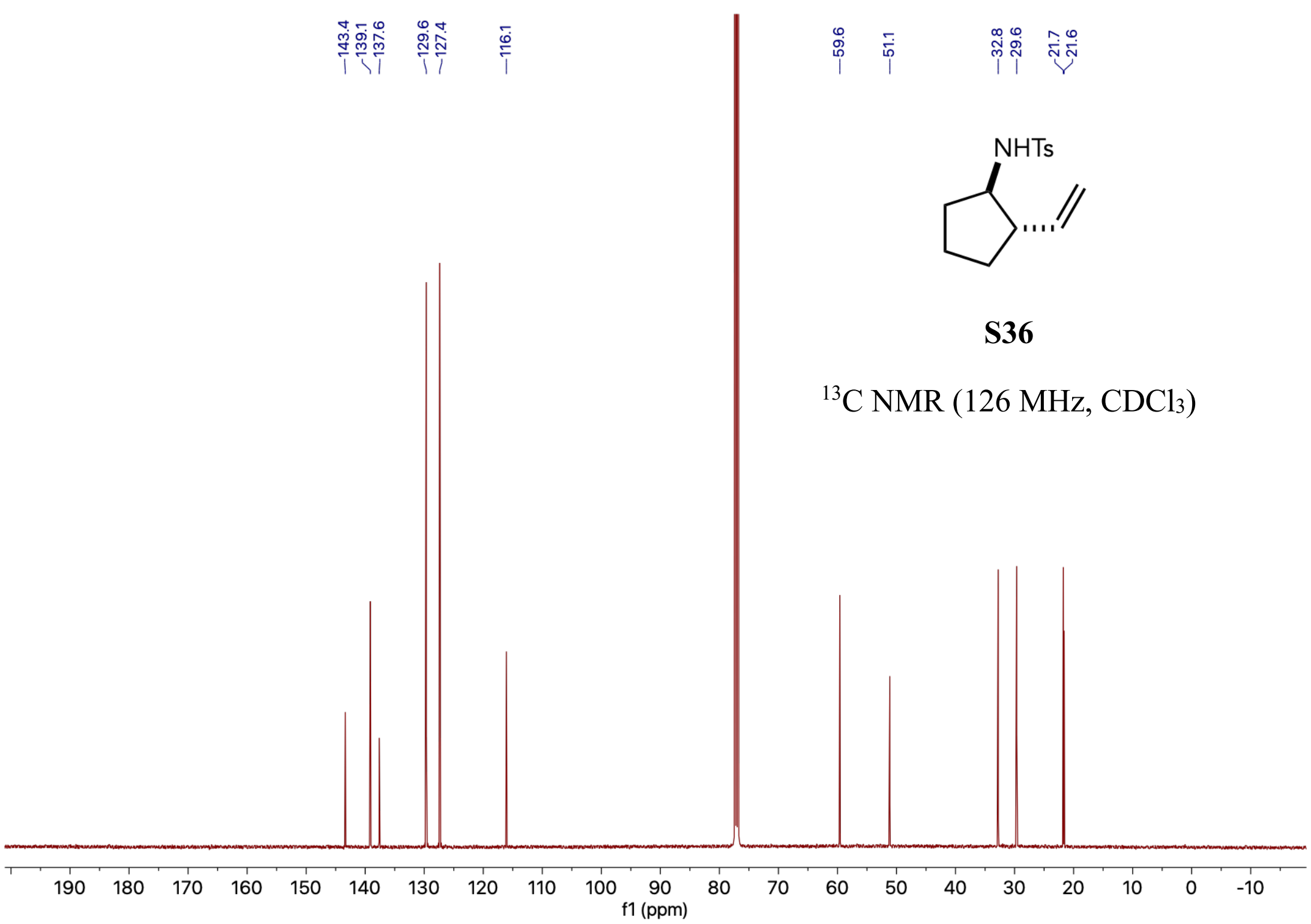
143.4
139.1
137.6
129.6
127.4
116.1
59.6
51.1
32.8
29.6
21.7
21.6
NHTs
S36
^{13}C NMR (126 MHz, CDCl$_3$)
190 180 170 160 150 140 130 120 110 100 90 80 70 60 50 40 30 20 10 0 -10
f1 (ppm)

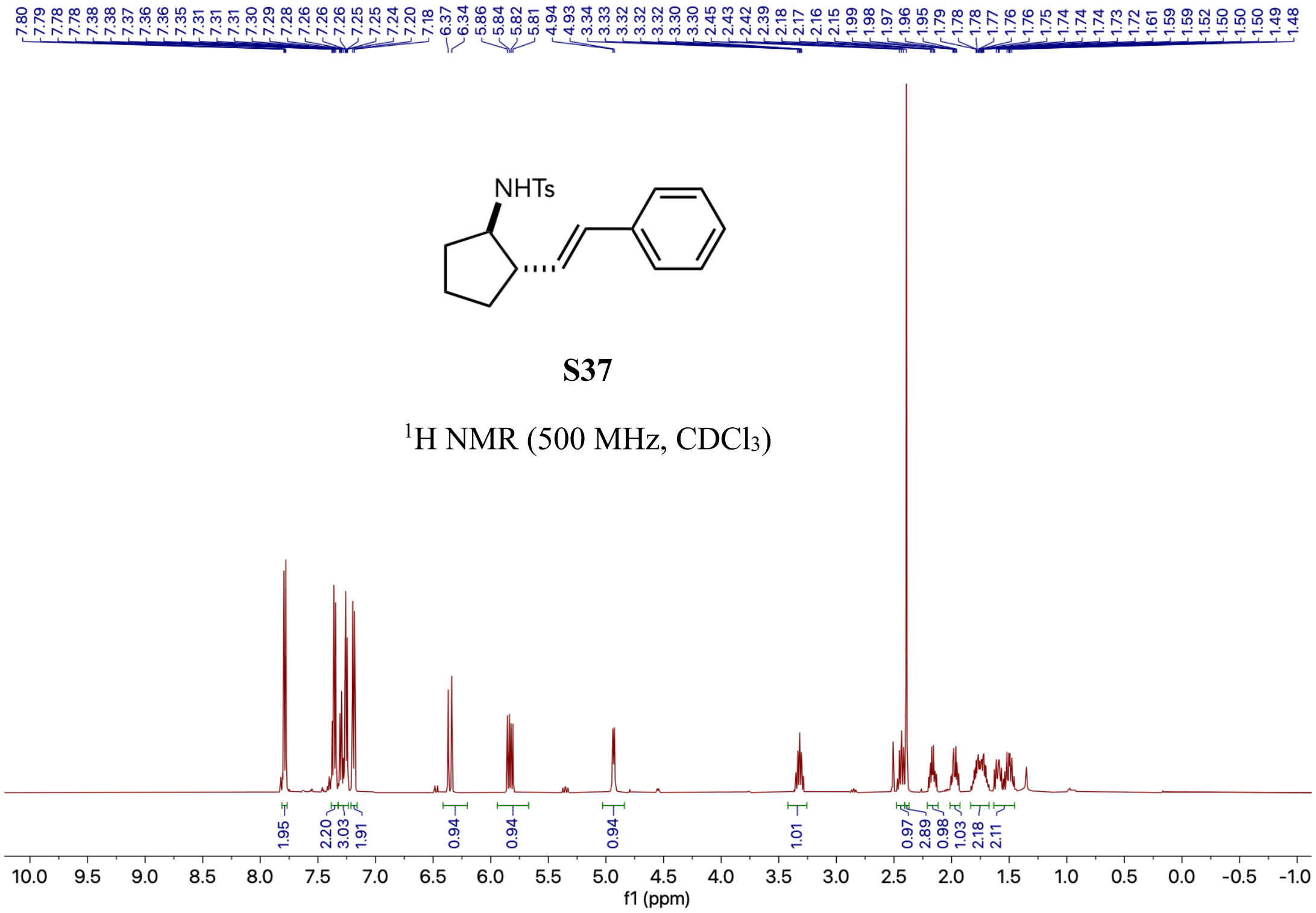

S37

^{1}H NMR (500 MHz, $CDCl_3$)

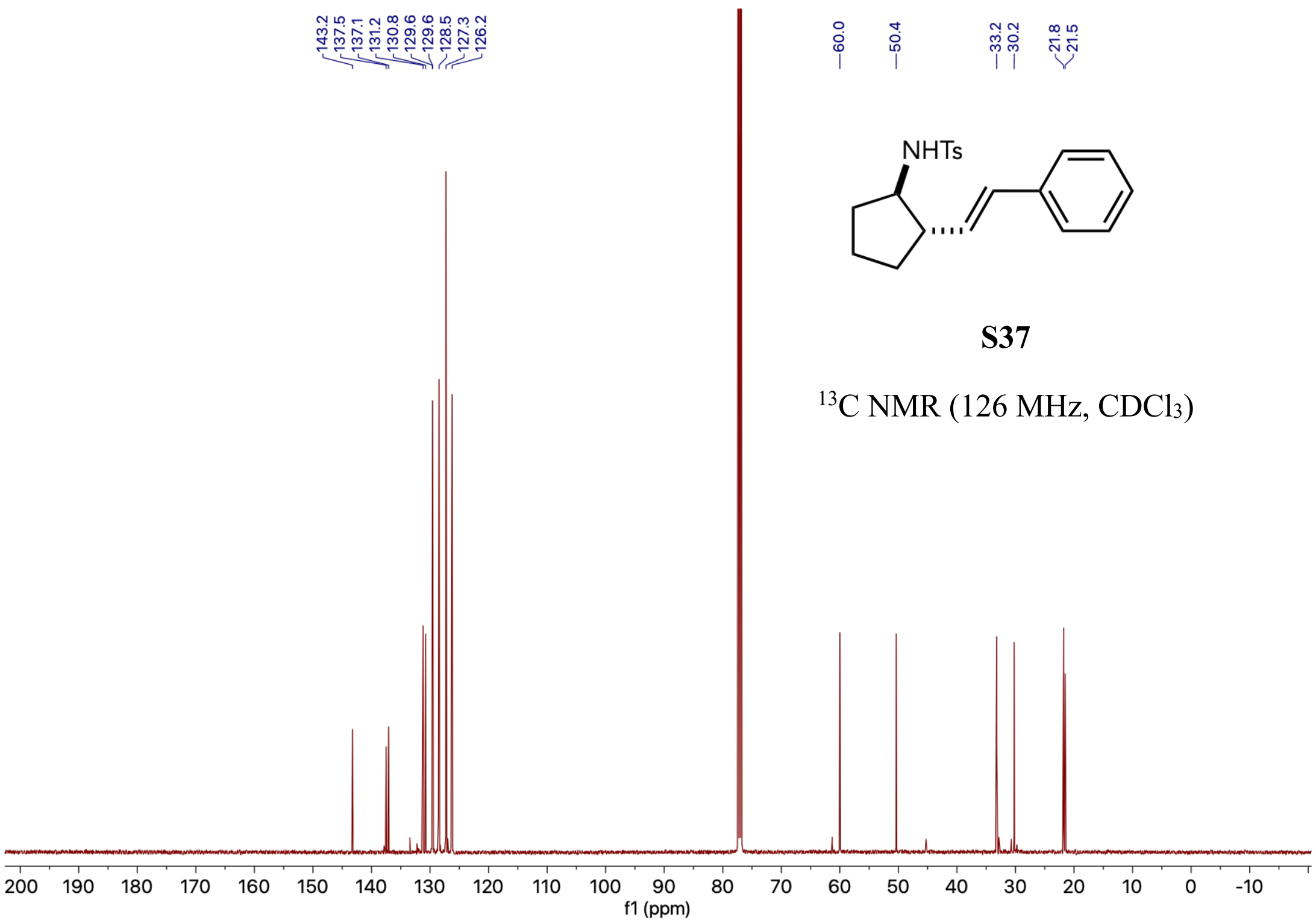

S37

^{13}C NMR (126 MHz, $CDCl_3$)

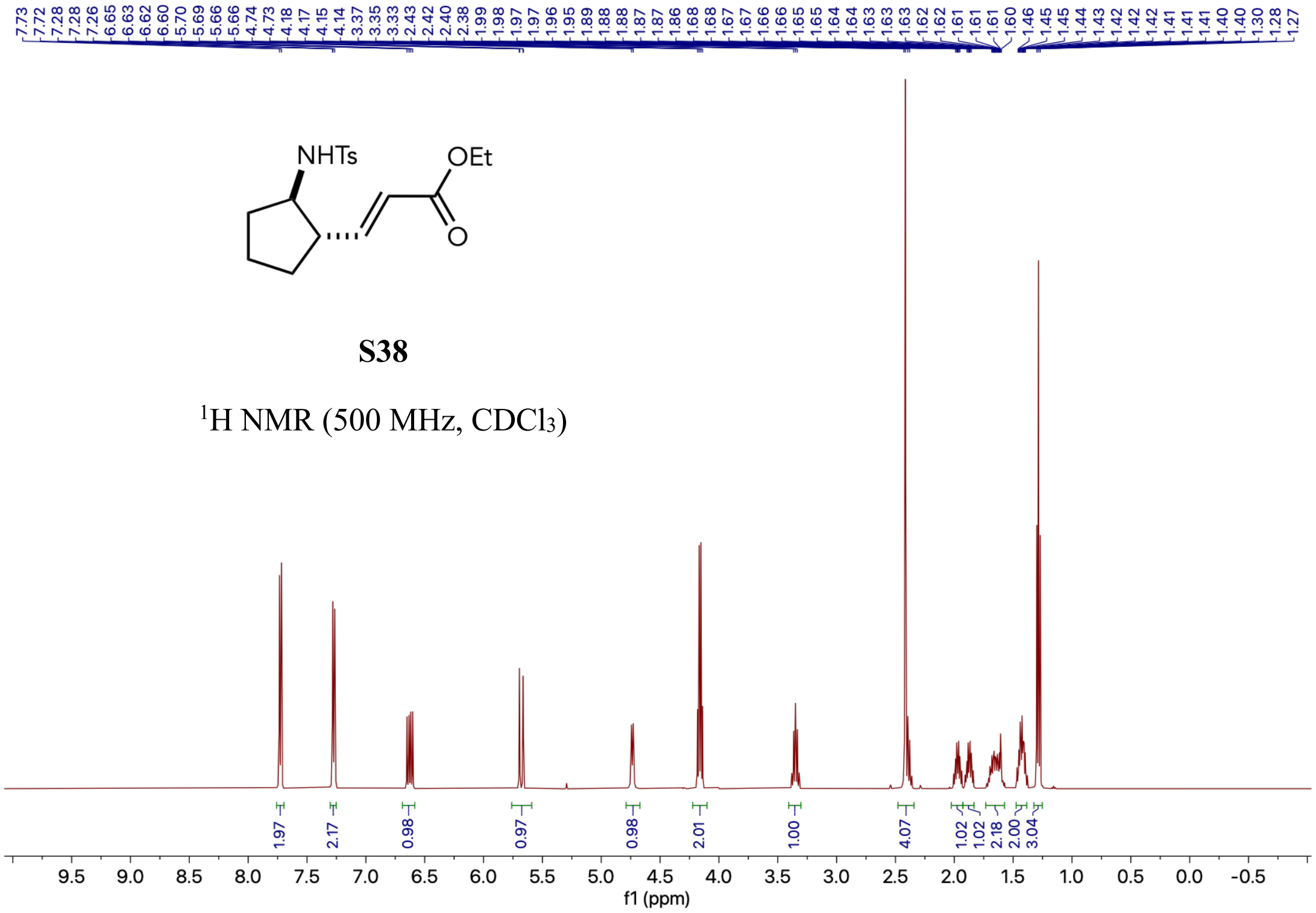
7.73 7.72 7.28 7.28 7.26 6.65 6.63 6.62 6.60 5.70 5.69 5.66 5.66 4.74 4.73 4.18 4.17 4.15 4.14 3.37 3.35 3.33 2.43 2.42 2.40 2.38 1.99 1.98 1.97 1.97 1.96 1.95 1.89 1.88 1.88 1.87 1.87 1.86 1.68 1.68 1.67 1.66 1.66 1.65 1.65 1.64 1.64 1.63 1.63 1.63 1.62 1.62 1.61 1.61 1.61 1.60 1.46 1.45 1.45 1.44 1.43 1.42 1.42 1.42 1.41 1.41 1.40 1.30 1.28 1.27
NHTs
OEt
O
S38
^{1}H NMR (500 MHz, CDCl$_3$)
1.97 2.17 0.98 0.97 0.98 2.01 1.00 4.07 1.02 1.02 2.18 2.00 3.04
9.5 9.0 8.5 8.0 7.5 7.0 6.5 6.0 5.5 5.0 4.5 4.0 3.5 3.0 2.5 2.0 1.5 1.0 0.5 0.0 -0.5
f1 (ppm)

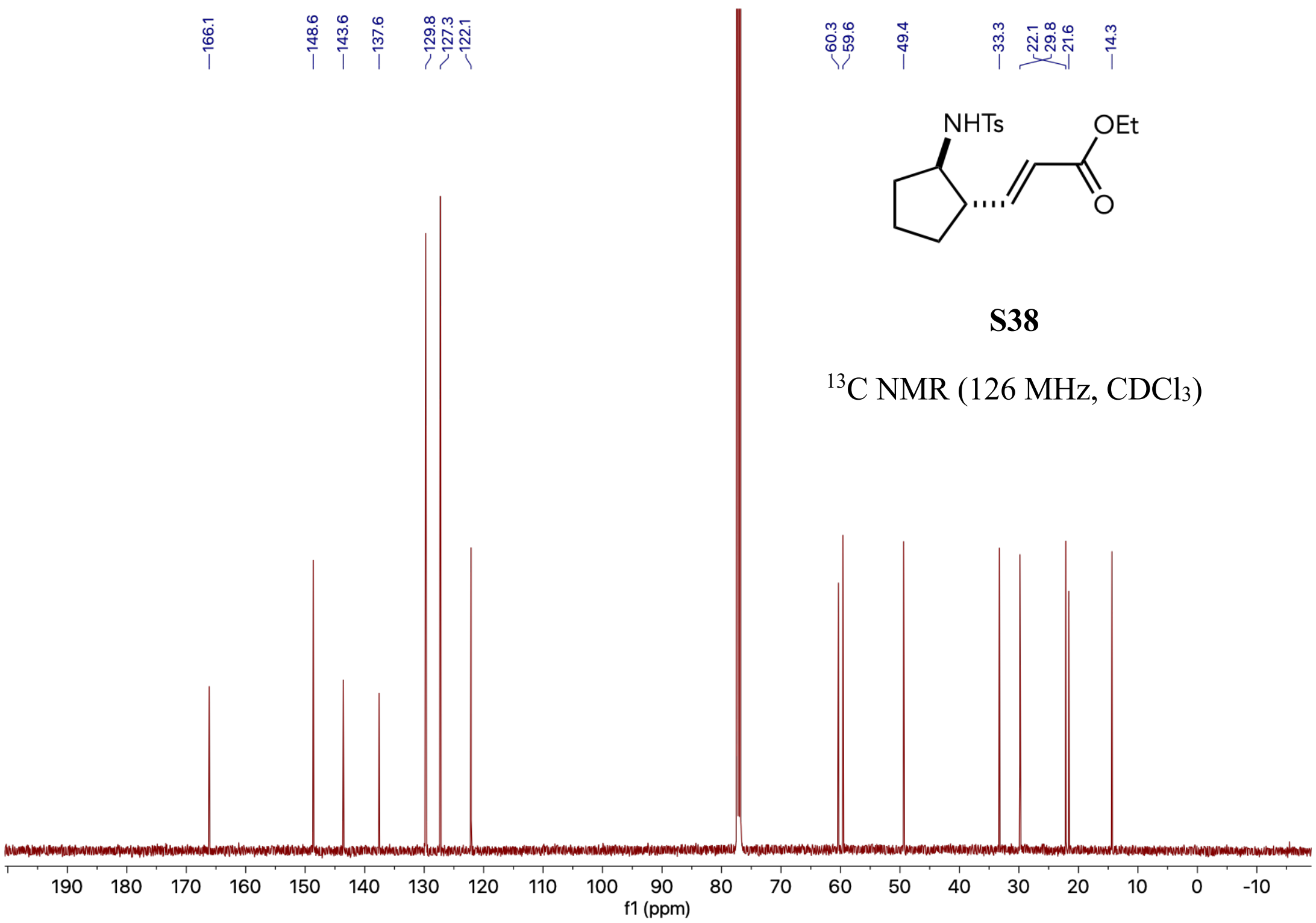
166.1 148.6 143.6 137.6 129.8 127.3 122.1 60.3 59.6 49.4 33.3 22.1 29.8 21.6 14.3
NHTs
OEt
O
S38
^{13}C NMR (126 MHz, CDCl$_3$)
190 180 170 160 150 140 130 120 110 100 90 80 70 60 50 40 30 20 10 0 -10
f1 (ppm)

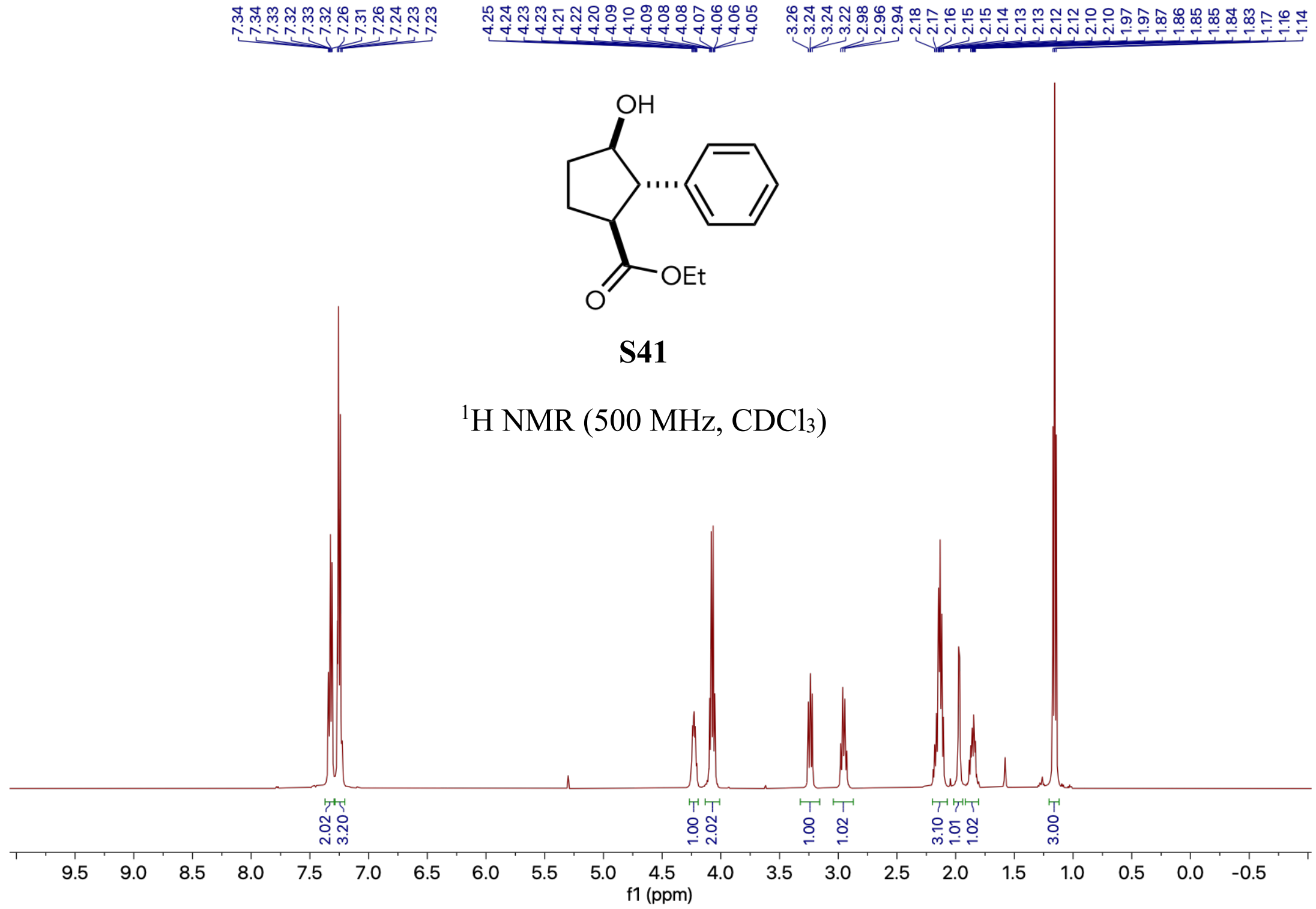

S41

^{1}H NMR (500 MHz, $CDCl_3$)

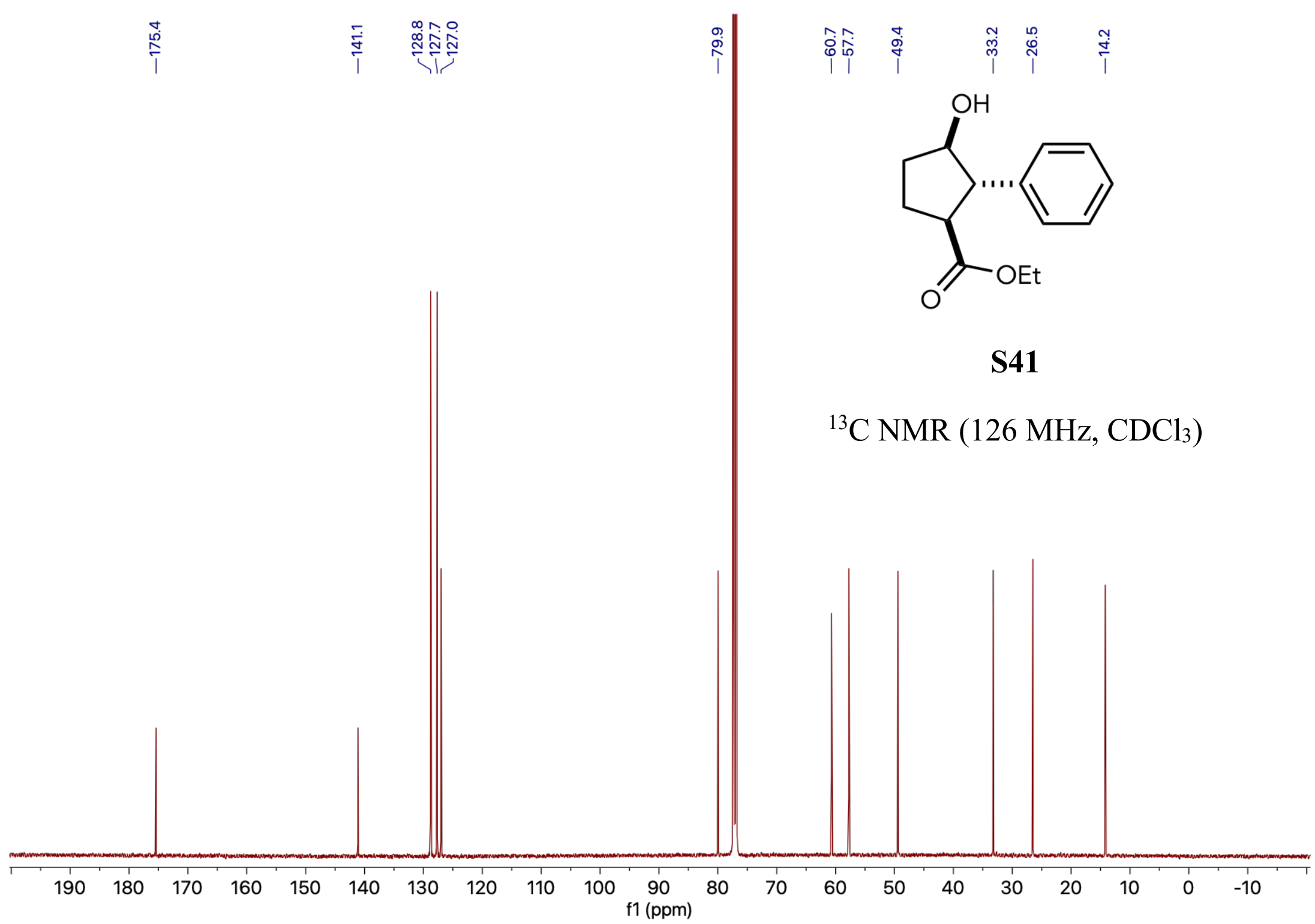

S41

^{13}C NMR (126 MHz, $CDCl_3$)

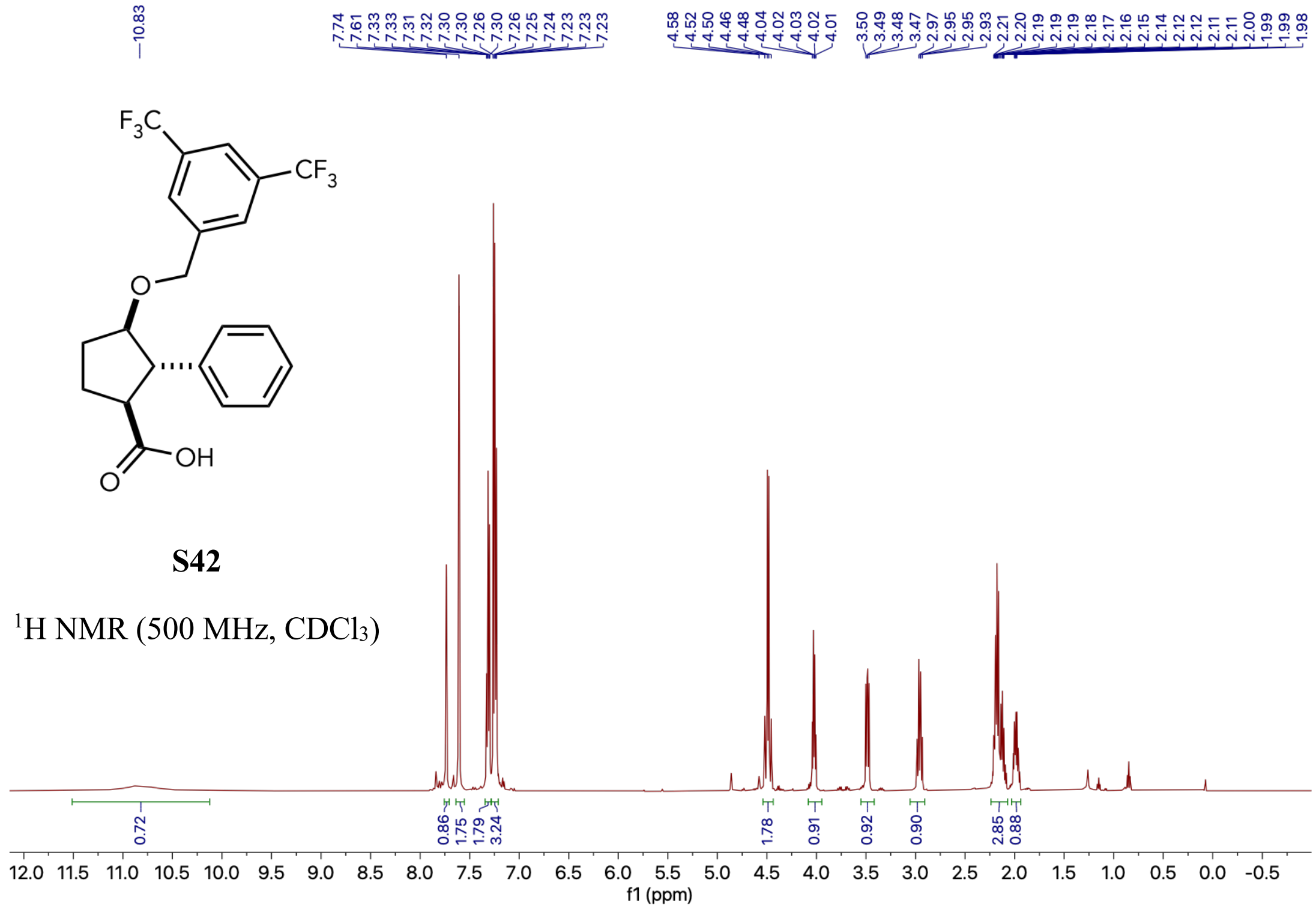

S42

^{1}H NMR (500 MHz, $CDCl_3$)

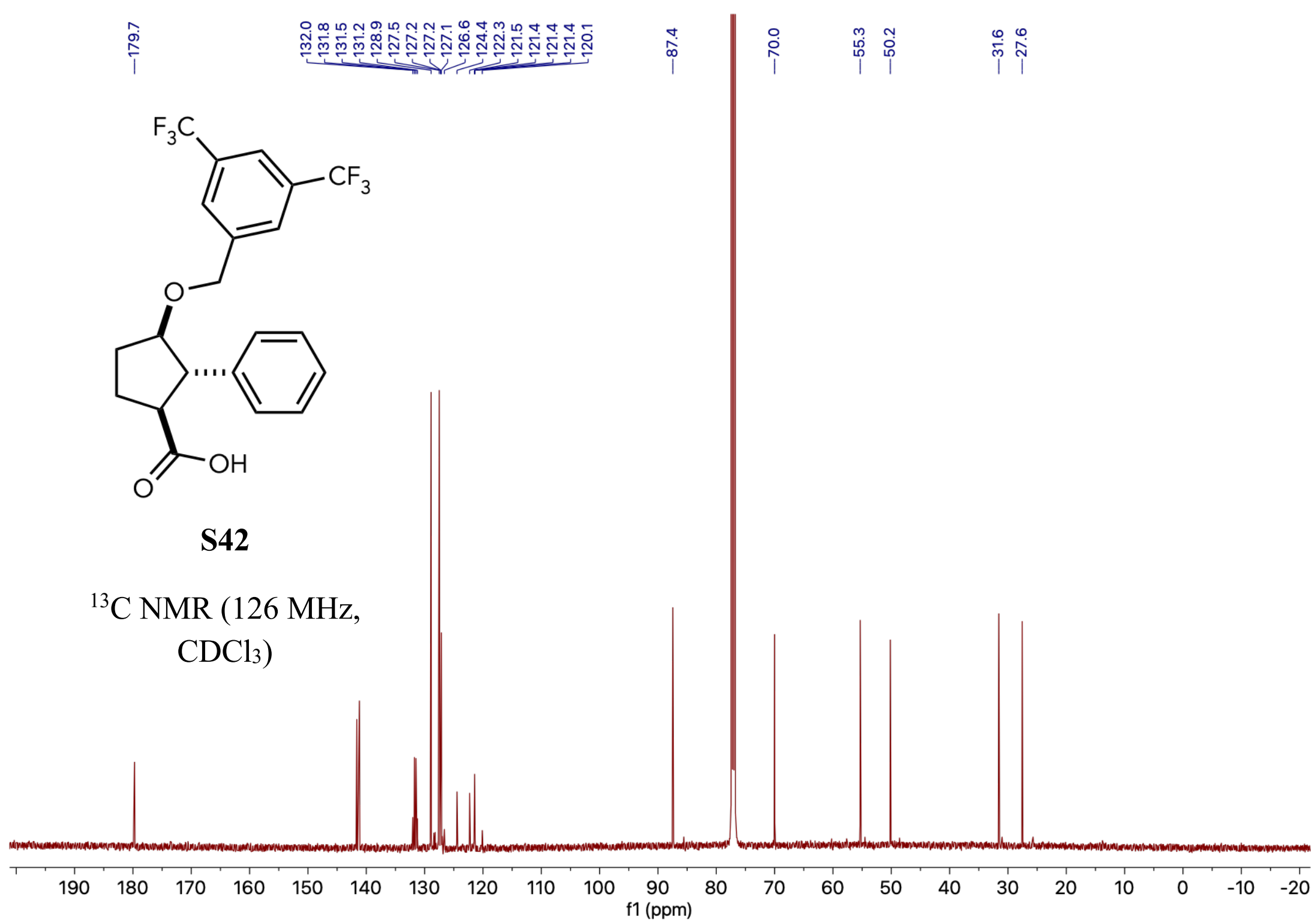

S42

^{13}C NMR (126 MHz, $CDCl_3$)

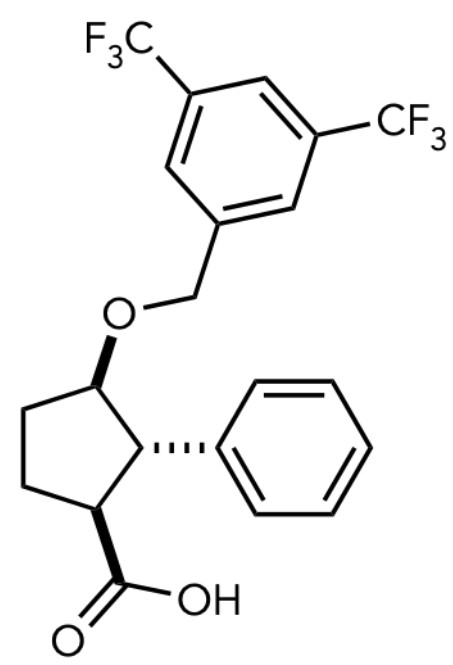

S42

^{19}F NMR (377 MHz, $CDCl_3$)

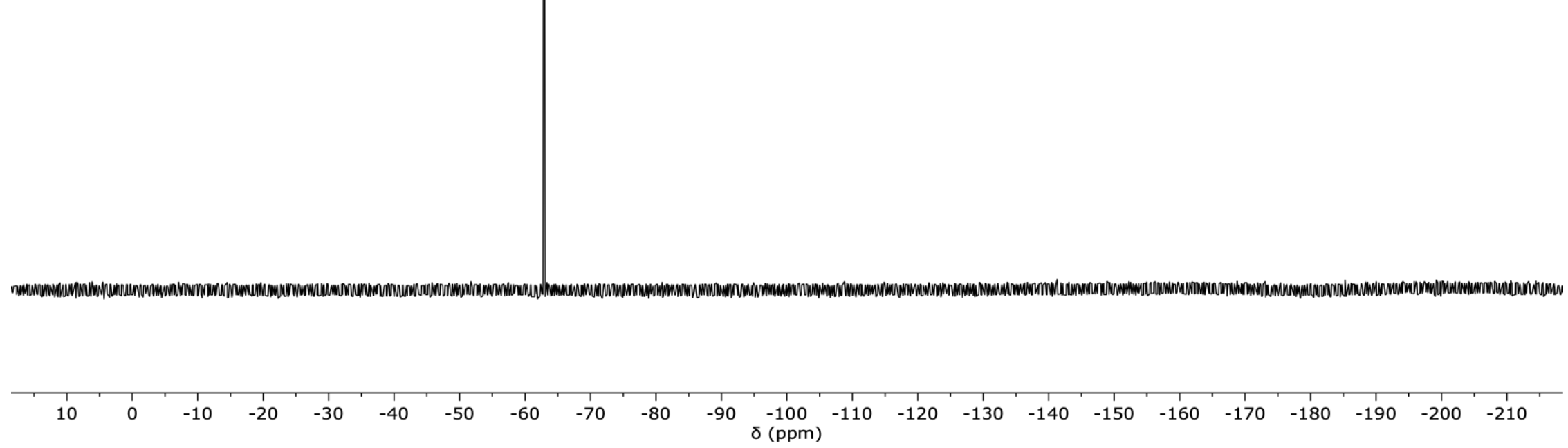

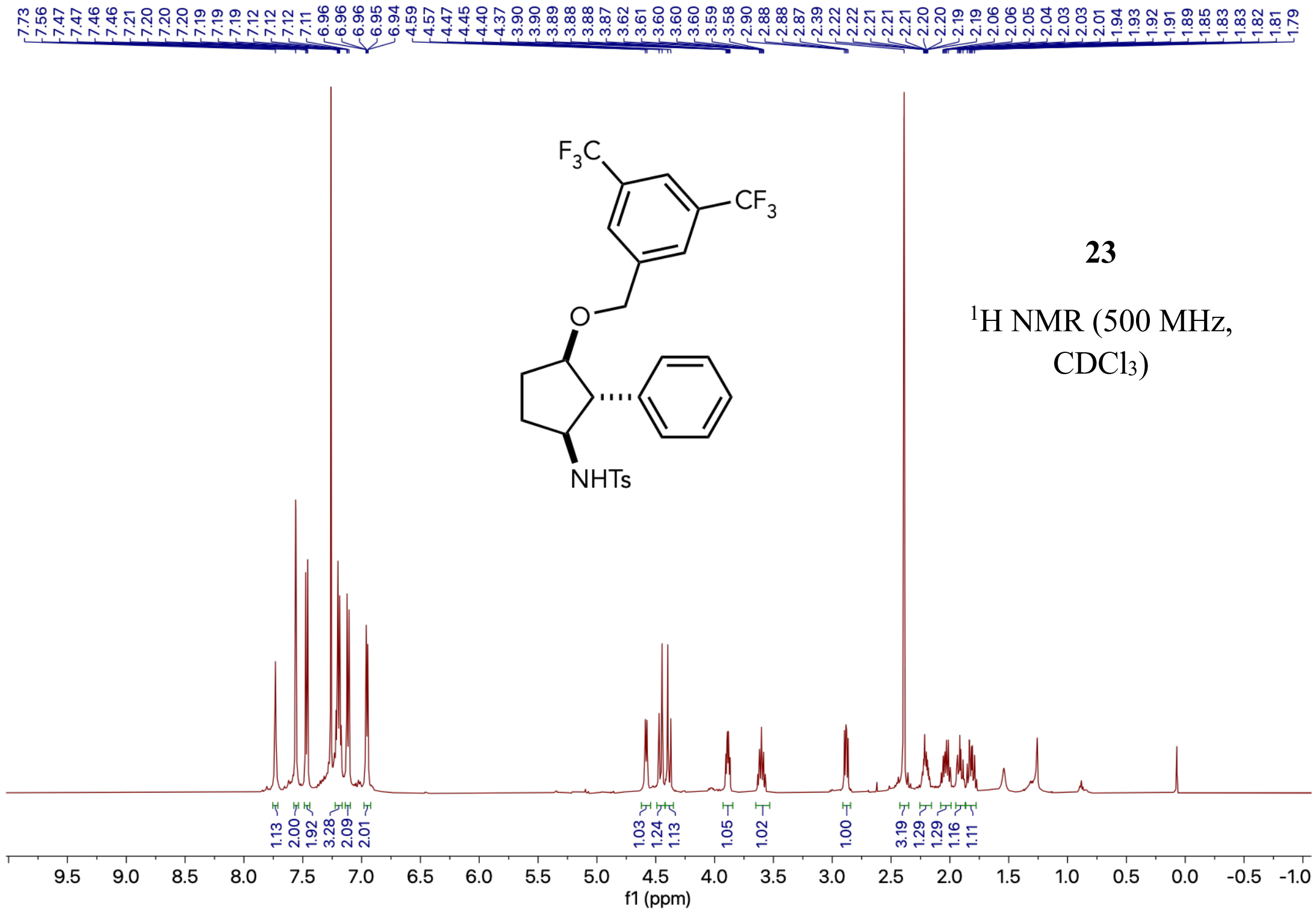
F3C
CF3
O
NHTs
23
1H NMR (500 MHz, CDCl3)
f1 (ppm)

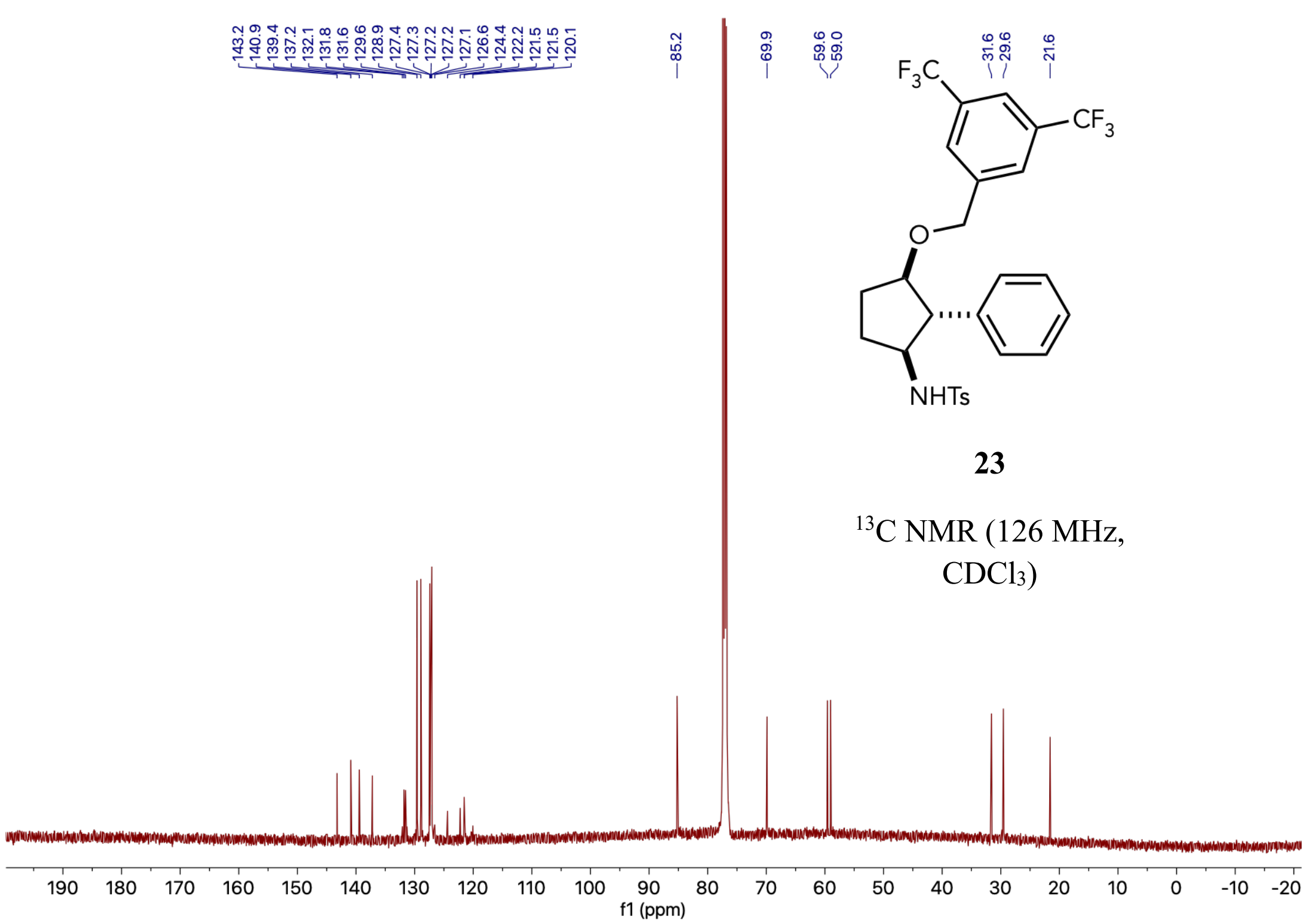
F3C
CF3
O
NHTs
23
13C NMR (126 MHz, CDCl3)
f1 (ppm)

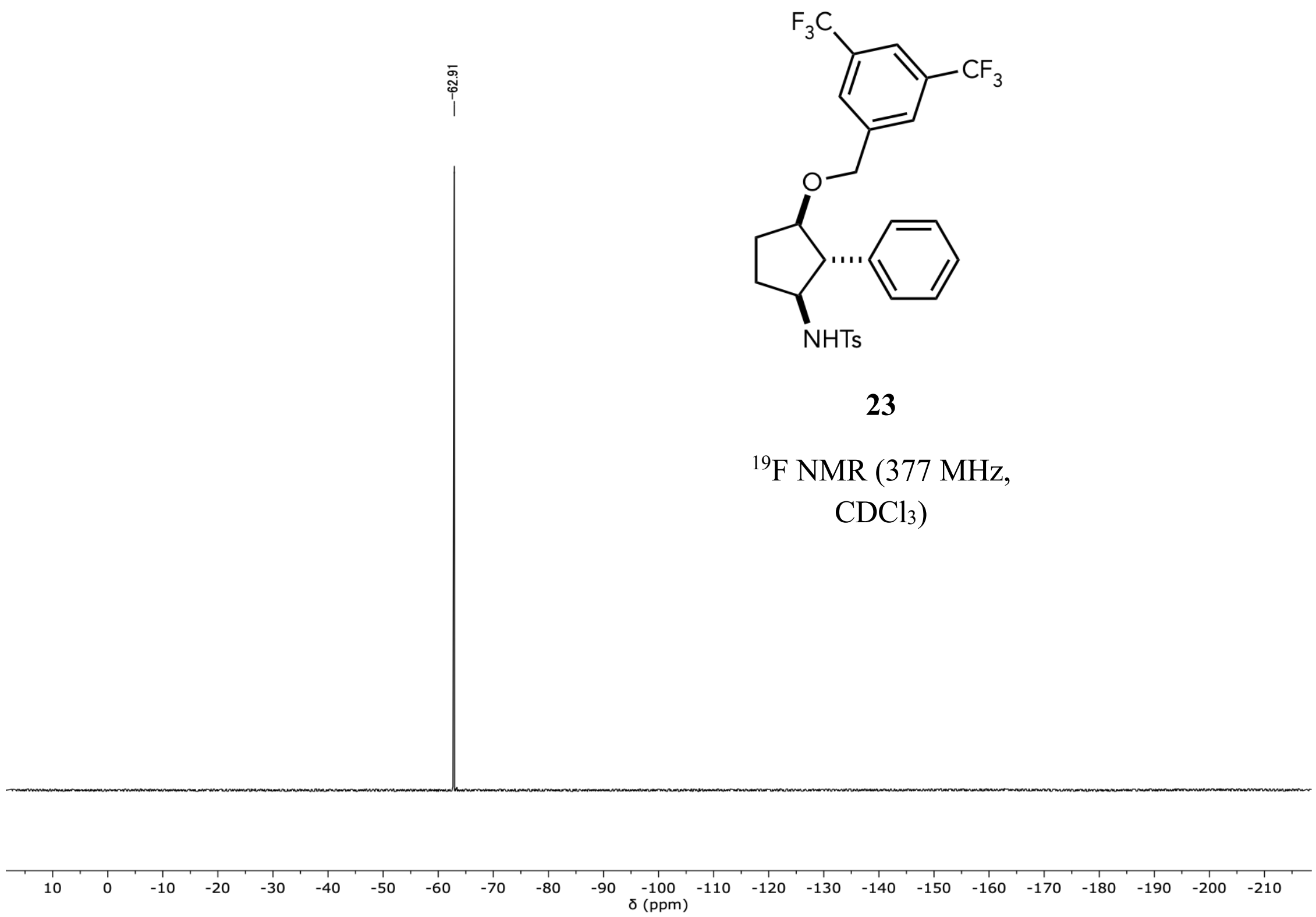

23

^{19}F NMR (377 MHz, $CDCl_3$)

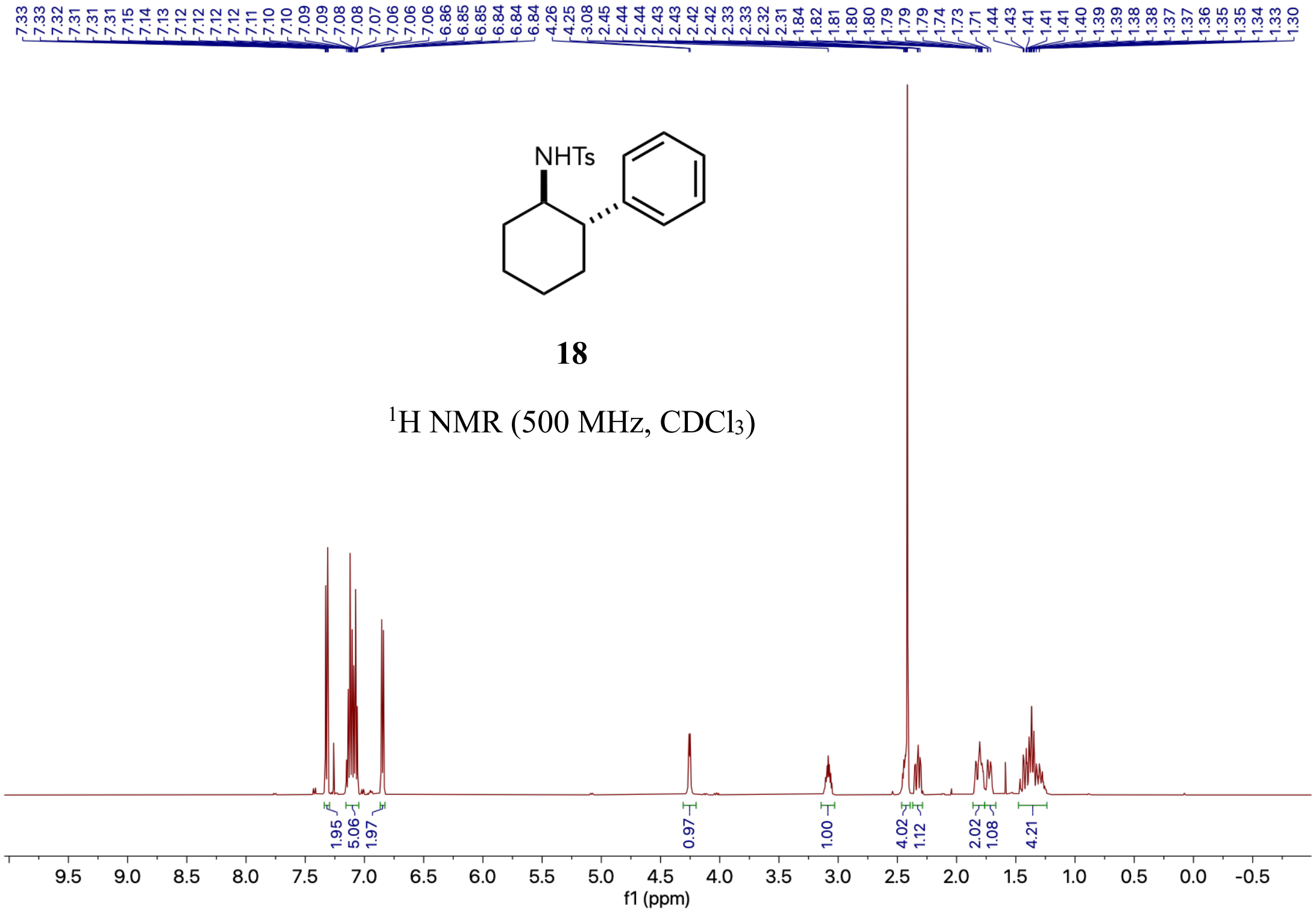
NHTs
18
1H NMR (500 MHz, CDCl3)
7.33 7.33 7.32 7.31 7.31 7.15 7.14 7.13 7.12 7.12 7.12 7.11 7.10 7.10 7.09 7.08 7.08 7.07 7.06 7.06 7.06 6.86 6.85 6.84 6.84 6.84 4.26 4.25 3.08 2.45 2.44 2.44 2.43 2.43 2.42 2.42 2.33 2.33 2.32 2.31 1.84 1.82 1.81 1.80 1.80 1.79 1.79 1.79 1.74 1.73 1.71 1.44 1.43 1.41 1.41 1.41 1.40 1.39 1.39 1.38 1.38 1.37 1.37 1.36 1.35 1.35 1.34 1.33 1.30
1.95 5.06 1.97 0.97 1.00 4.02 1.12 2.02 1.08 4.21
9.5 9.0 8.5 8.0 7.5 7.0 6.5 6.0 5.5 5.0 4.5 4.0 3.5 3.0 2.5 2.0 1.5 1.0 0.5 0.0 -0.5
f1 (ppm)

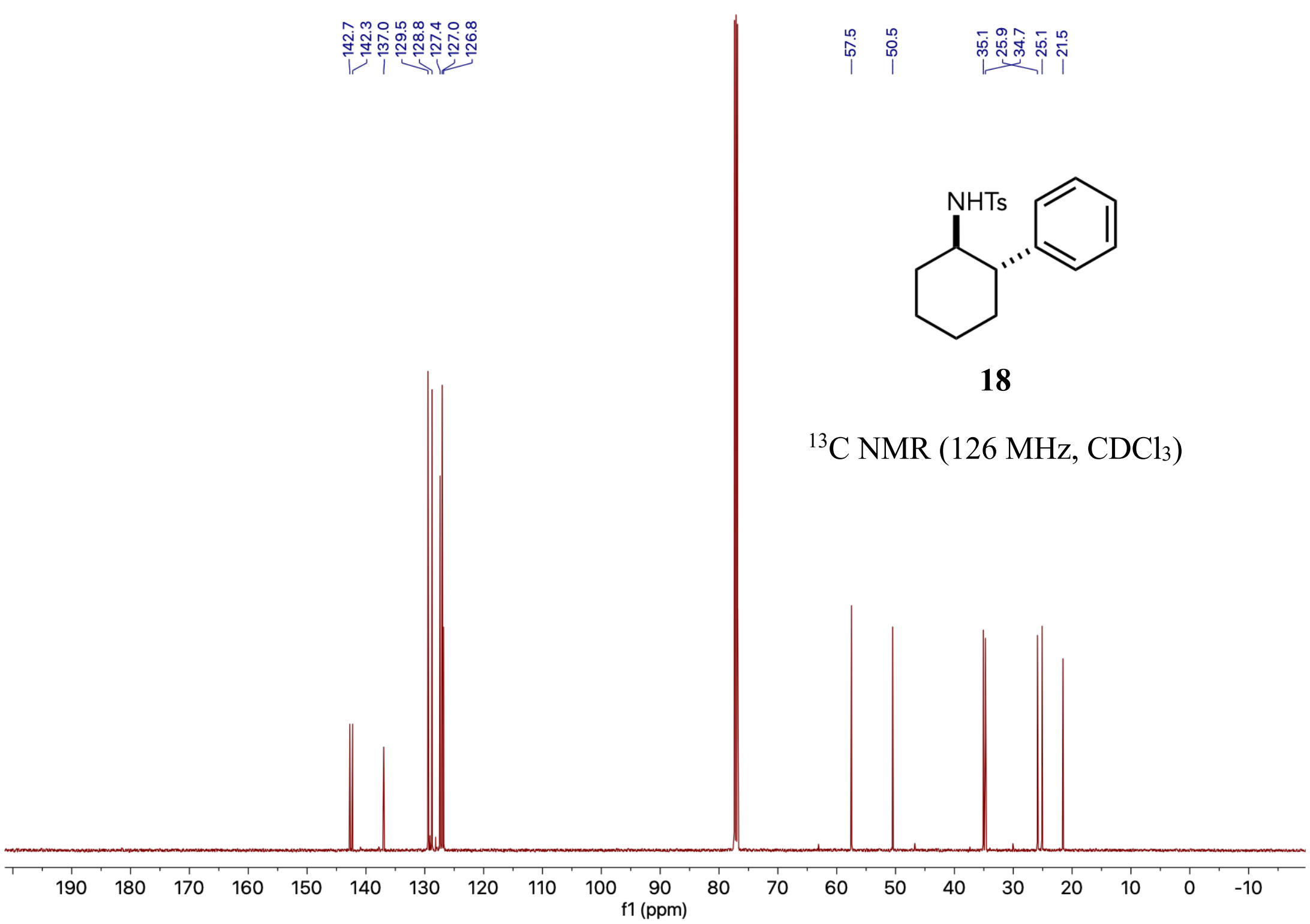
142.7 142.3 137.0 129.5 128.8 127.4 127.0 126.8
57.5
50.5
35.1 25.9 34.7 25.1 21.5
NHTs
18
13C NMR (126 MHz, CDCl3)
190 180 170 160 150 140 130 120 110 100 90 80 70 60 50 40 30 20 10 0 -10
f1 (ppm)

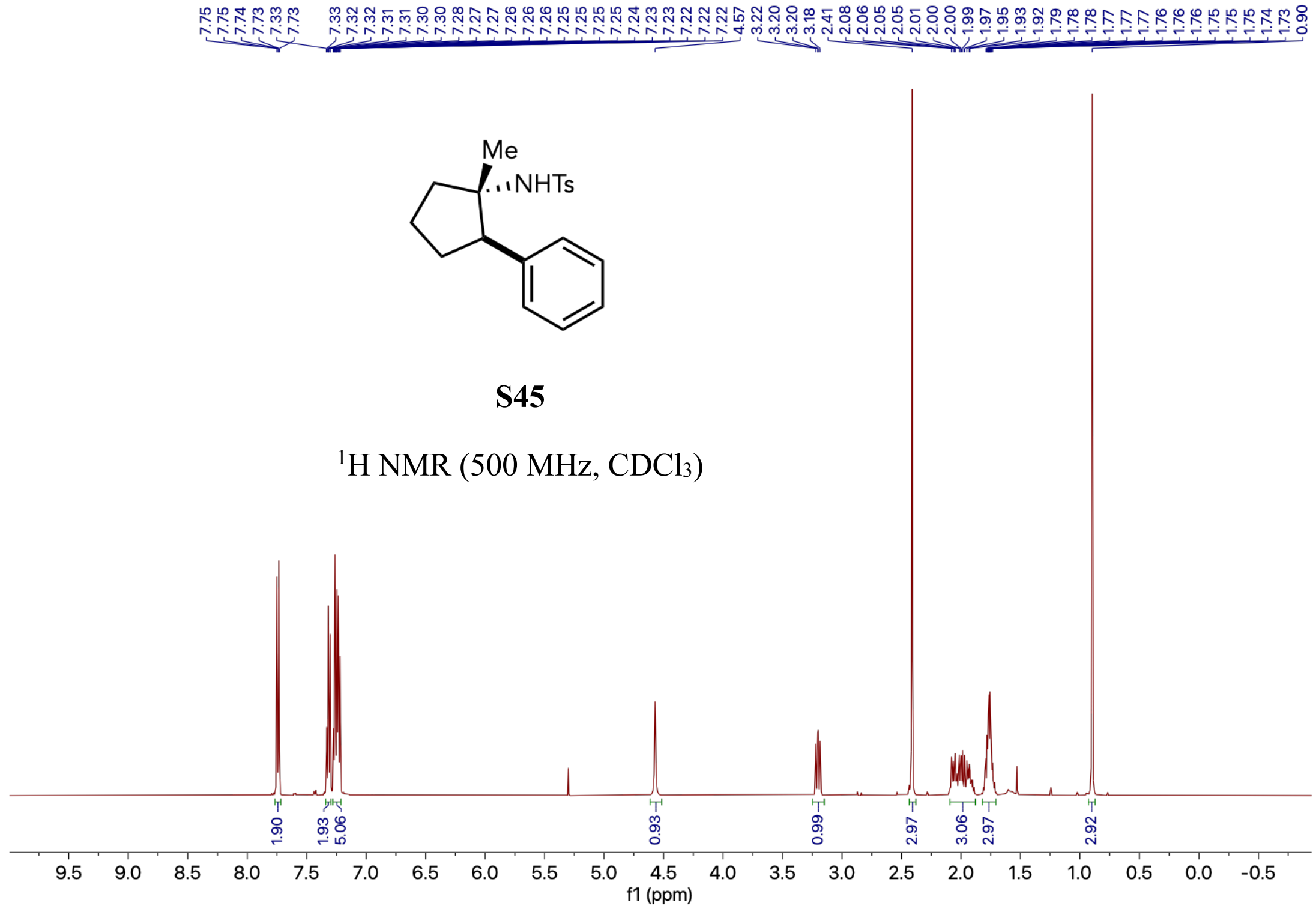
7.75 7.74 7.73 7.33 7.73 7.33 7.32 7.32 7.31 7.31 7.30 7.30 7.28 7.27 7.27 7.26 7.26 7.26 7.25 7.25 7.25 7.25 7.24 7.23 7.23 7.22 7.22 7.22 7.22 4.57 3.22 3.20 3.20 3.18 2.41 2.08 2.06 2.05 2.05 2.01 2.00 2.00 1.99 1.97 1.95 1.93 1.92 1.79 1.78 1.78 1.77 1.77 1.77 1.76 1.76 1.76 1.75 1.75 1.75 1.74 1.73 0.90
Me
NHTs
S45
1H NMR (500 MHz, CDCl3)
1.90
1.93
5.06
0.93
0.99
2.97
3.06
2.97
2.92
f1 (ppm)

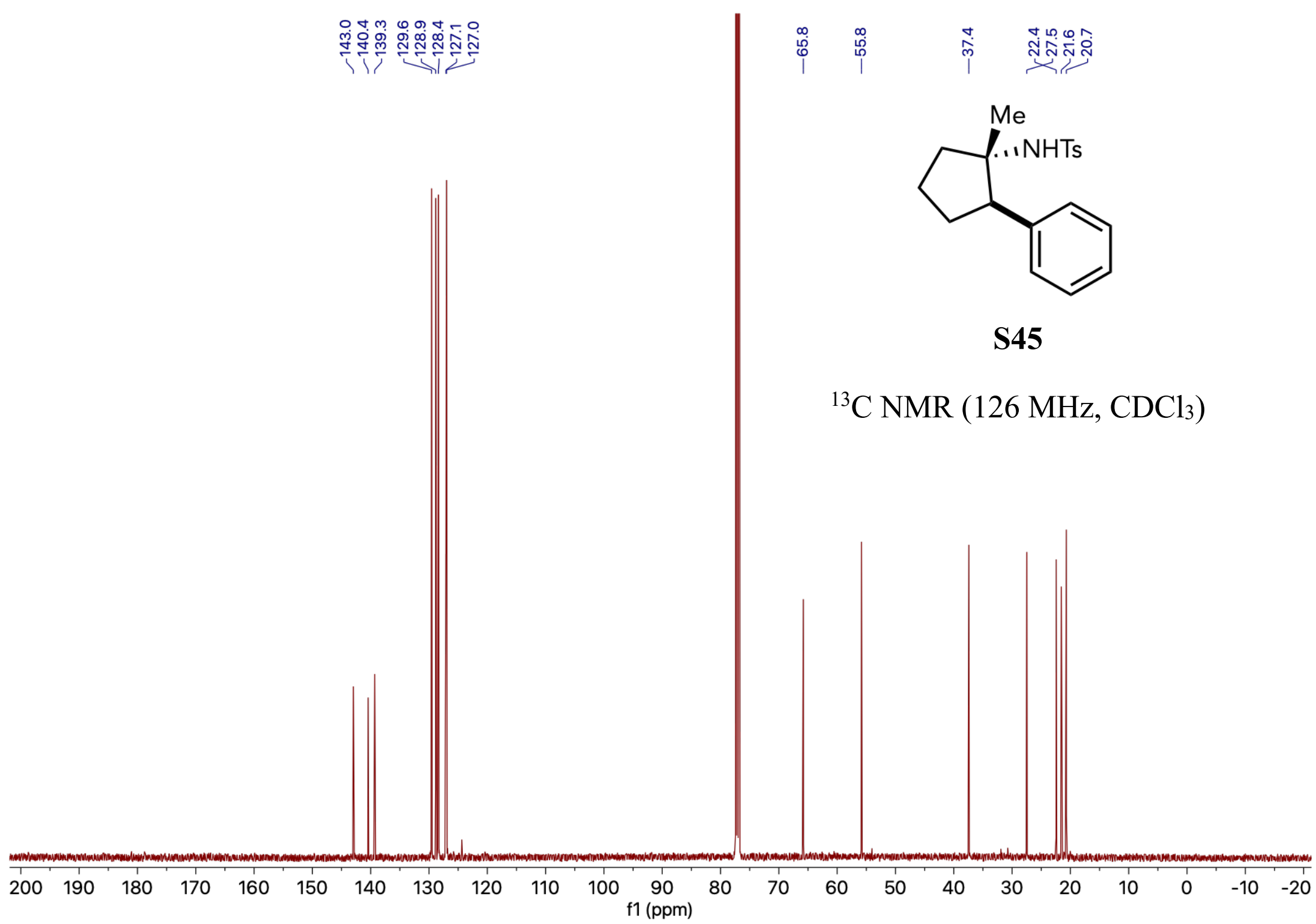
143.0 140.4 139.3 129.6 128.9 128.4 127.1 127.0
65.8
55.8
37.4
22.4 27.5 21.6 20.7
Me
NHTs
S45
13C NMR (126 MHz, CDCl3)
f1 (ppm)

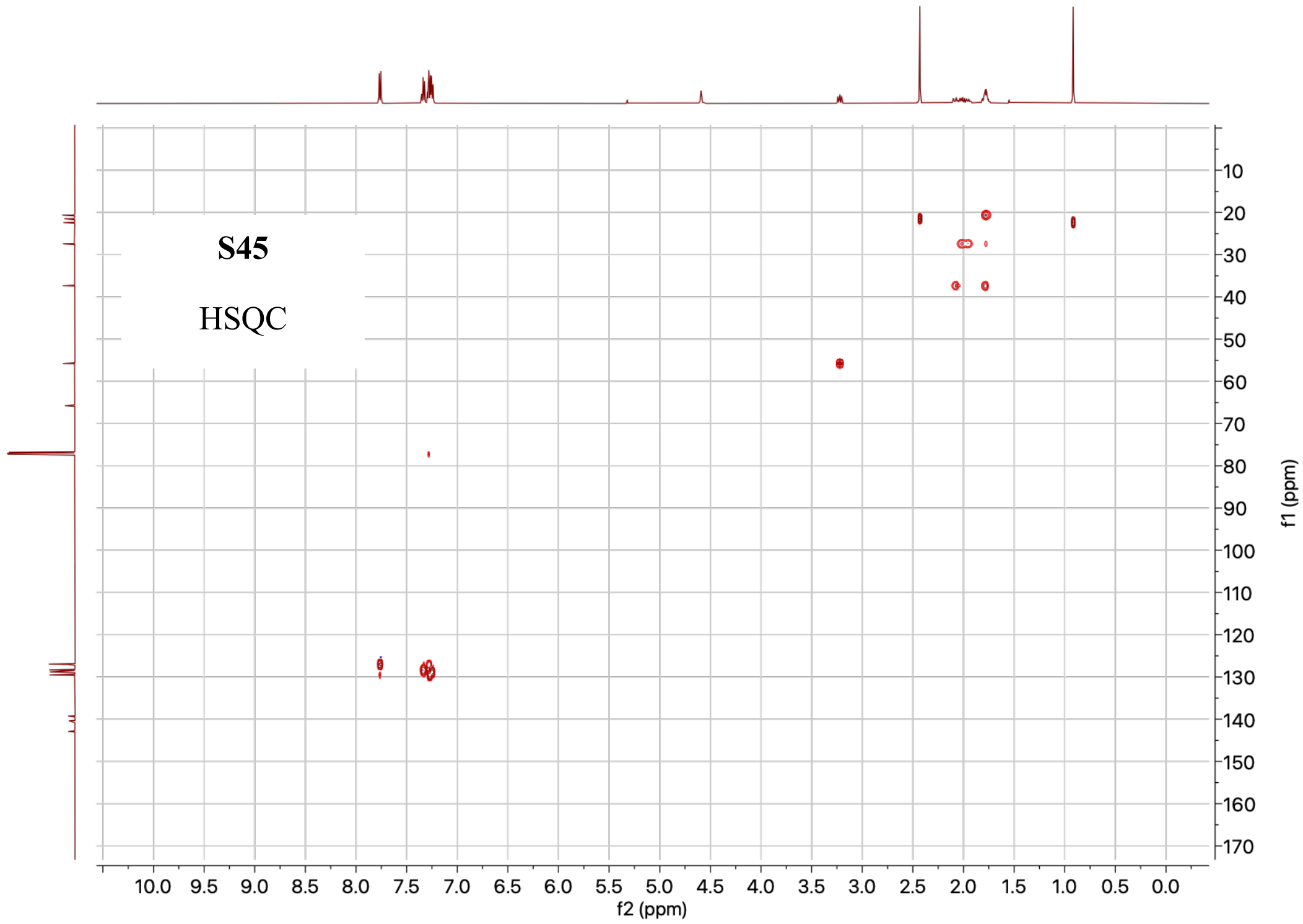
S45
HSQC
f1 (ppm)
f2 (ppm)

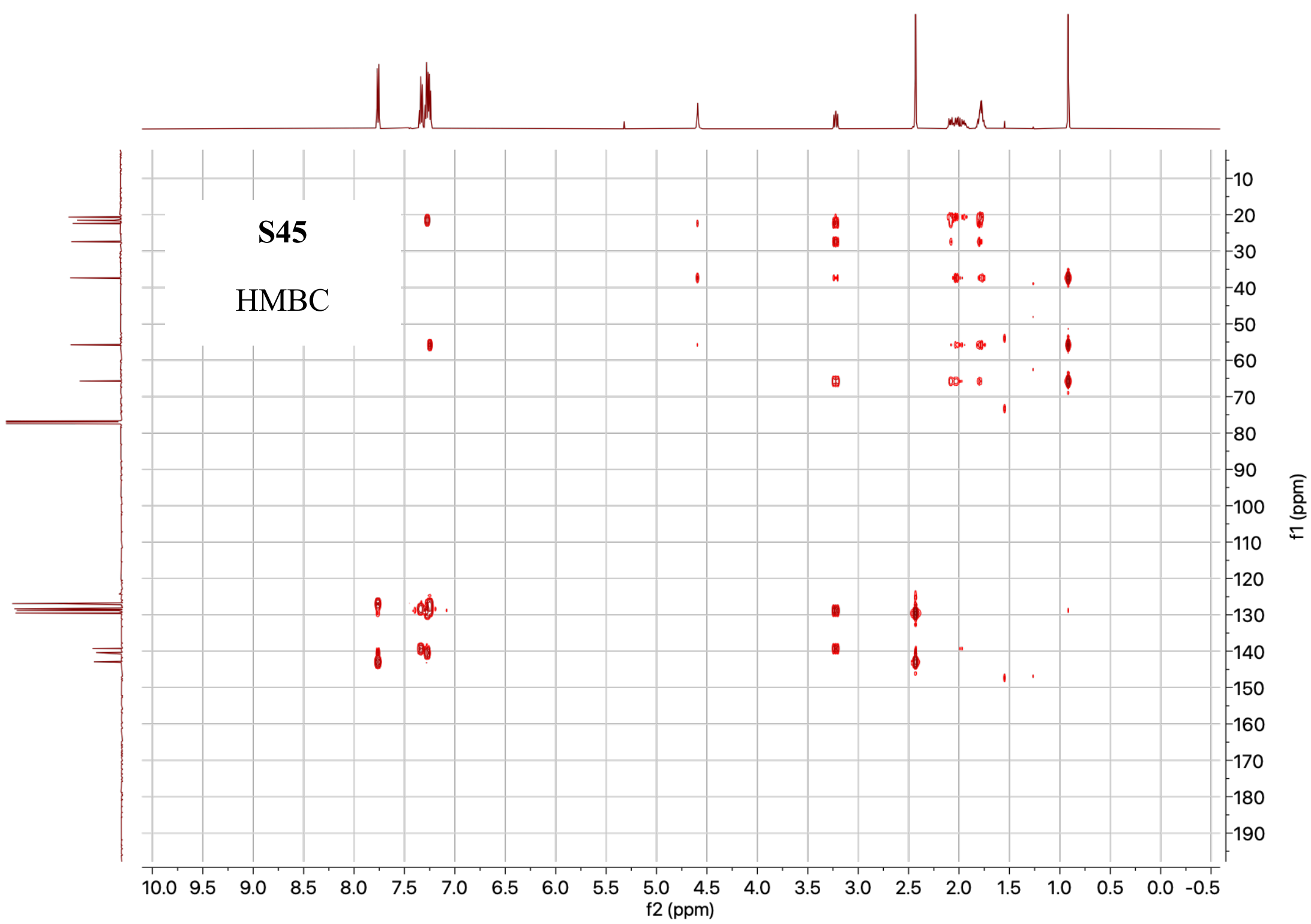
S45
HMBC
f1 (ppm)
f2 (ppm)

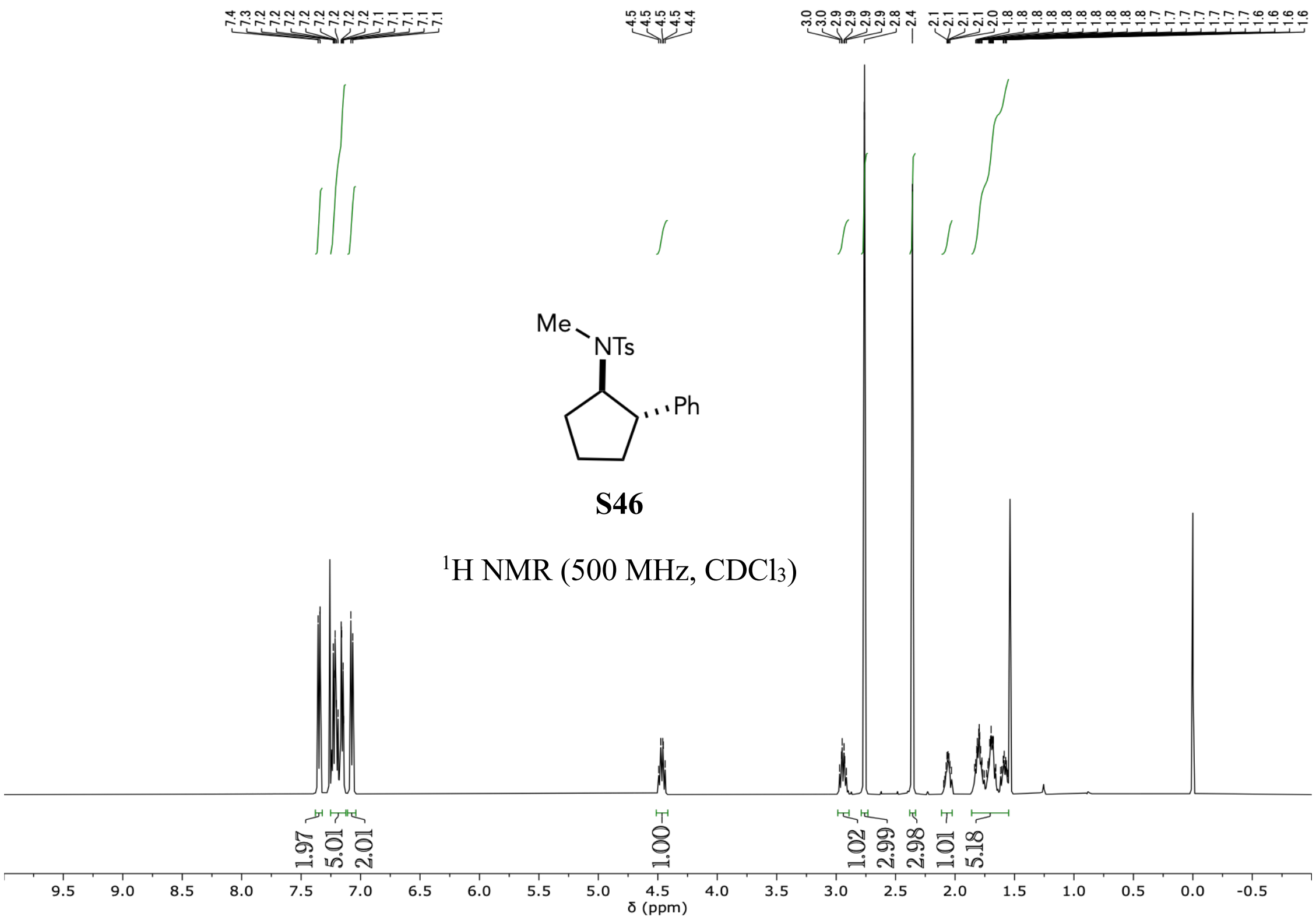

S46

^{1}H NMR (500 MHz, CDCl3)

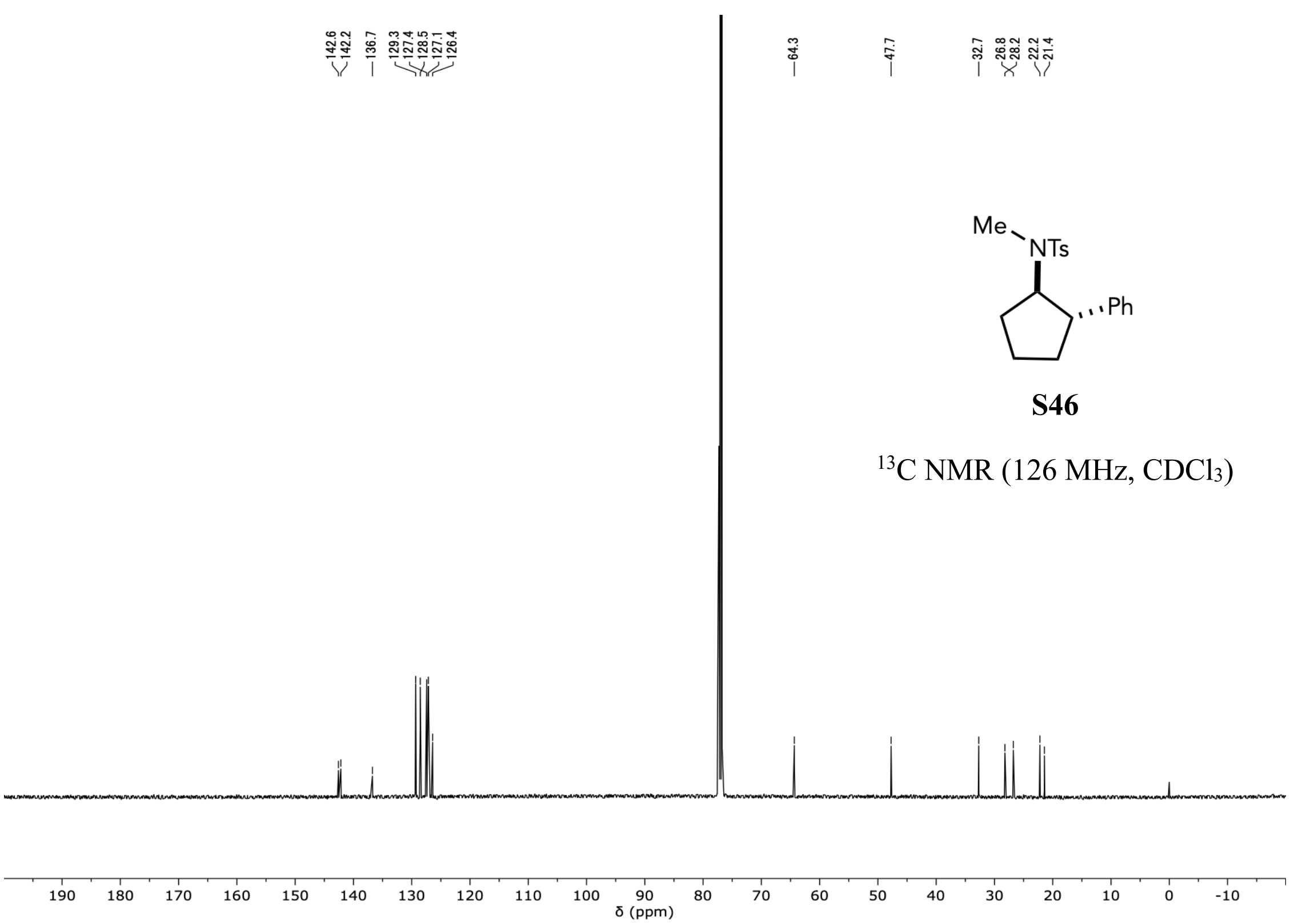

S46

^{13}C NMR (126 MHz, CDCl3)

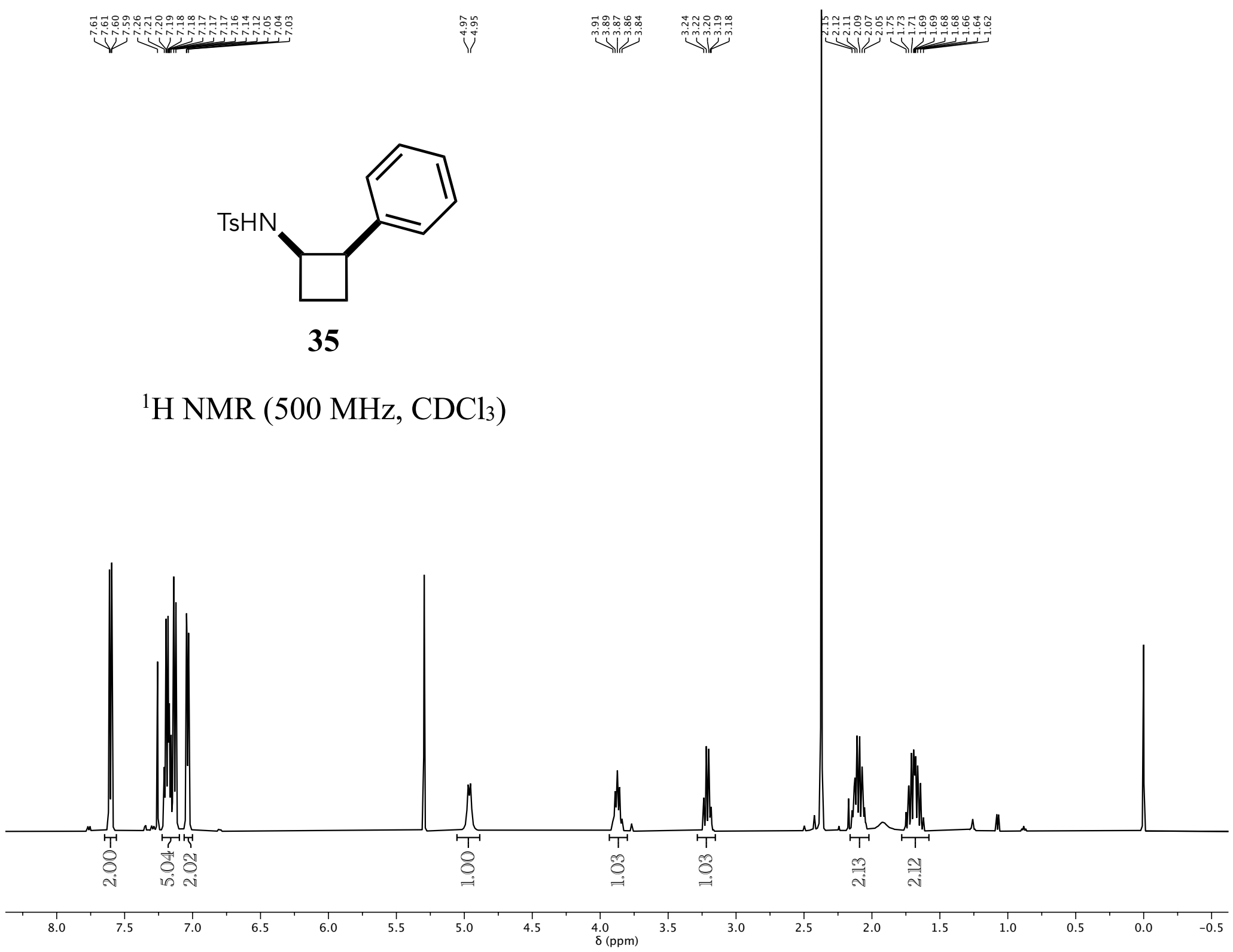

35

^{1}H NMR (500 MHz, $CDCl_3$)

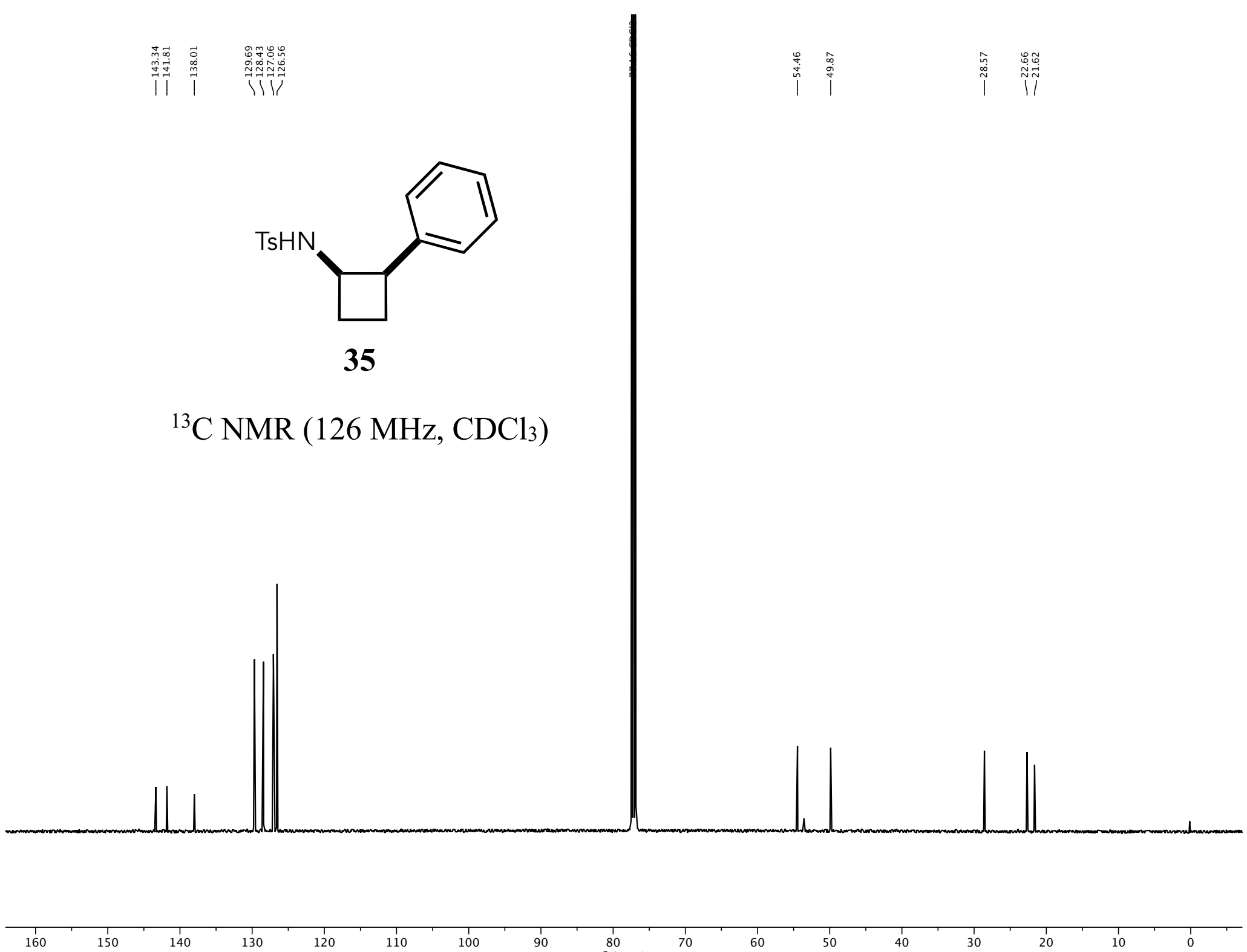

35

^{13}C NMR (126 MHz, $CDCl_3$)

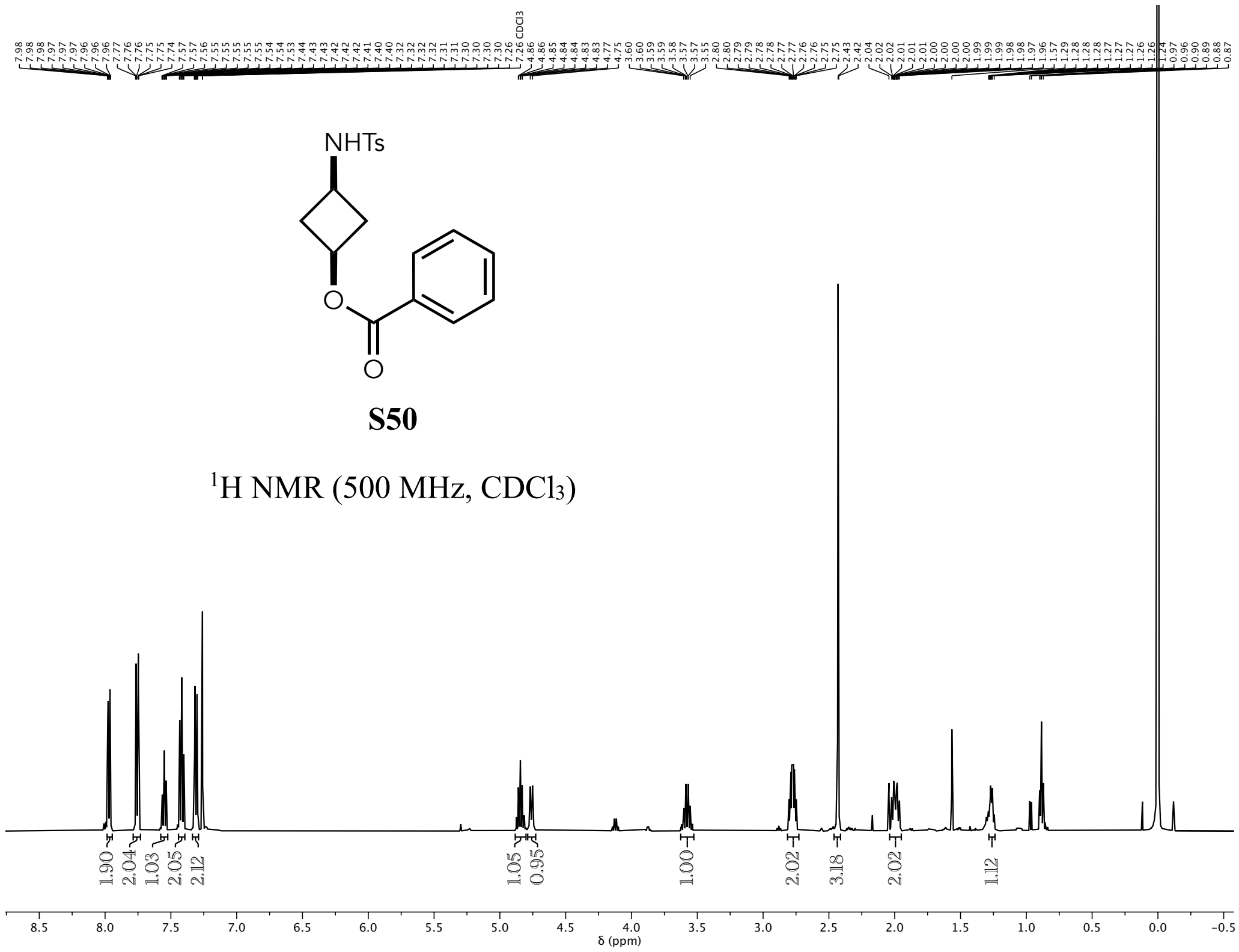
NHTs
O
O
S50
^{1}H NMR (500 MHz, CDCl$_3$)
1.90
2.04
1.03
2.05
2.12
1.05
0.95
1.00
2.02
3.18
2.02
1.12
δ (ppm)

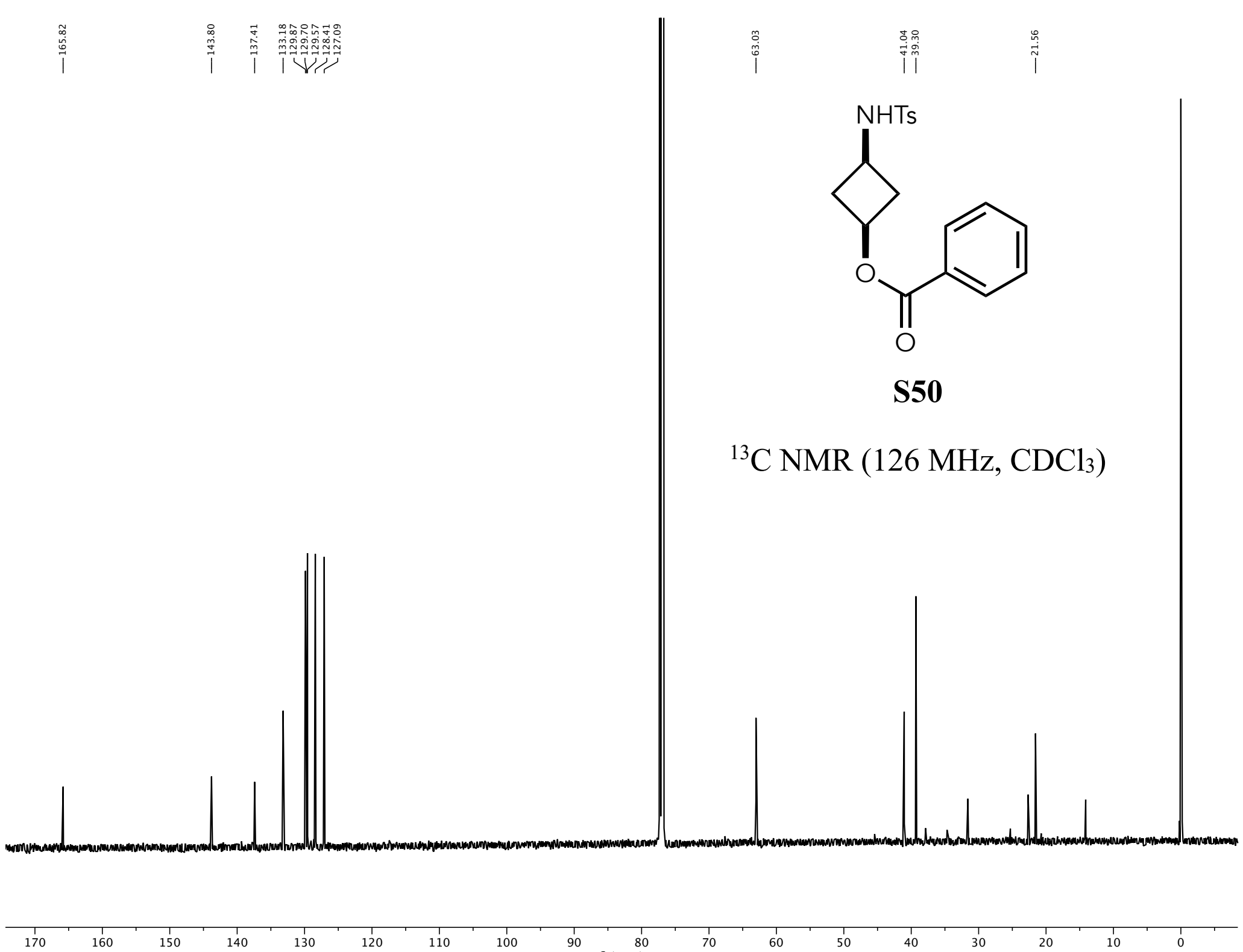
165.82
143.80
137.41
133.18
129.87
129.70
129.57
128.41
127.09
63.03
41.04
39.30
21.56
NHTs
O
O
S50
^{13}C NMR (126 MHz, CDCl$_3$)
δ (ppm)

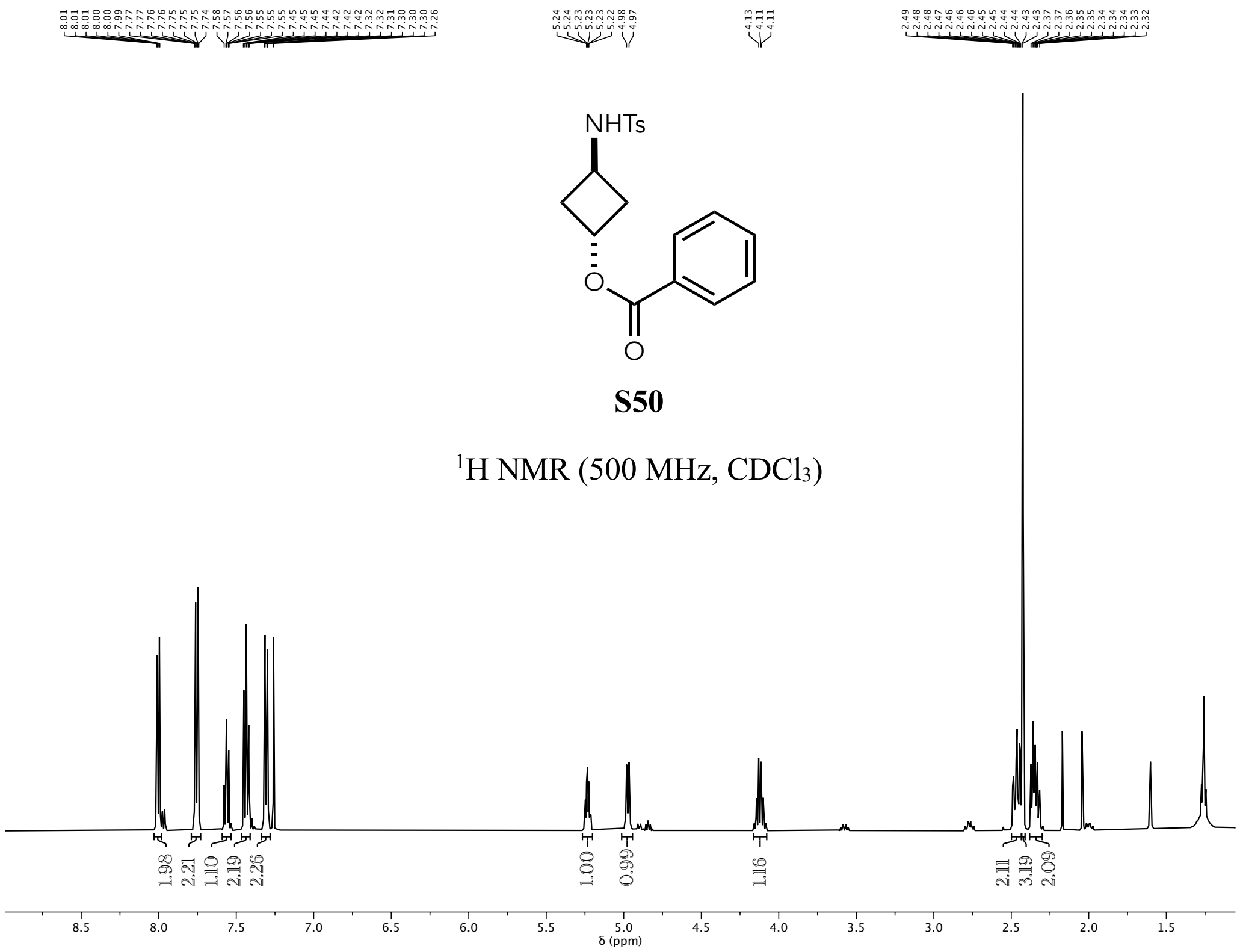

S50

^{1}H NMR (500 MHz, $CDCl_3$)

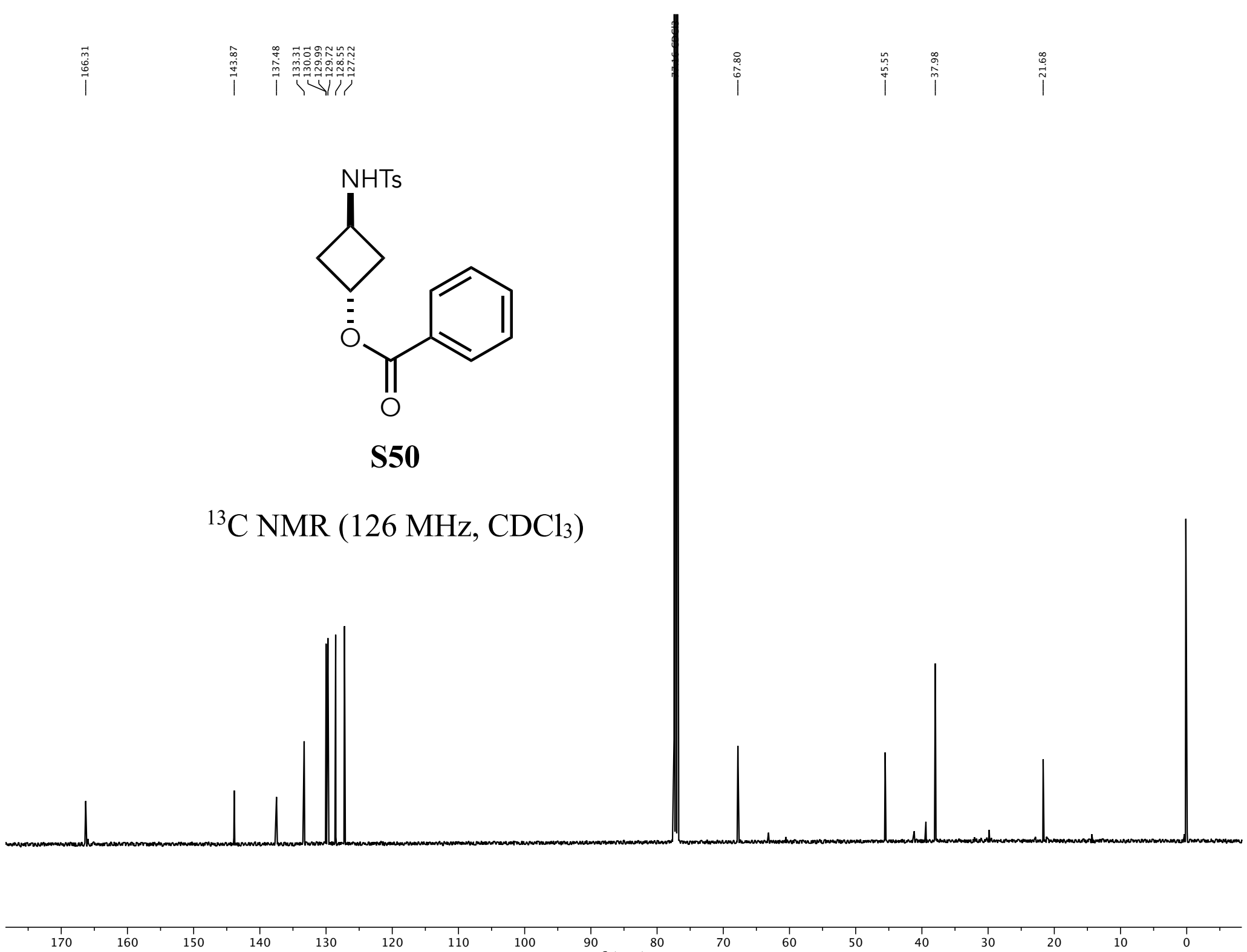

S50

^{13}C NMR (126 MHz, $CDCl_3$)

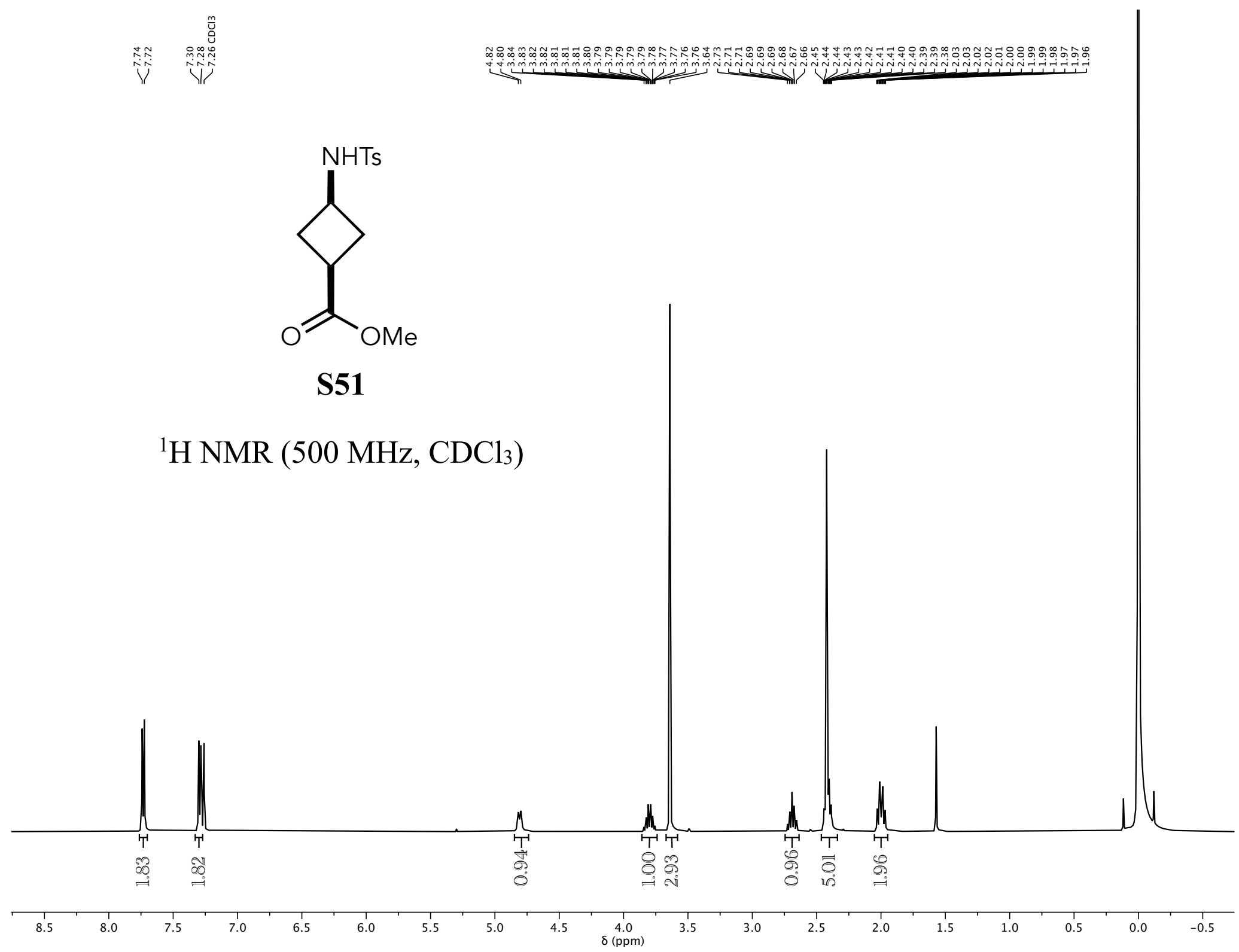
NHTs
OMe
O
S51
^{1}H NMR (500 MHz, CDCl$_3$)
1.83
1.82
0.94
1.00
2.93
0.96
5.01
1.96
δ (ppm)

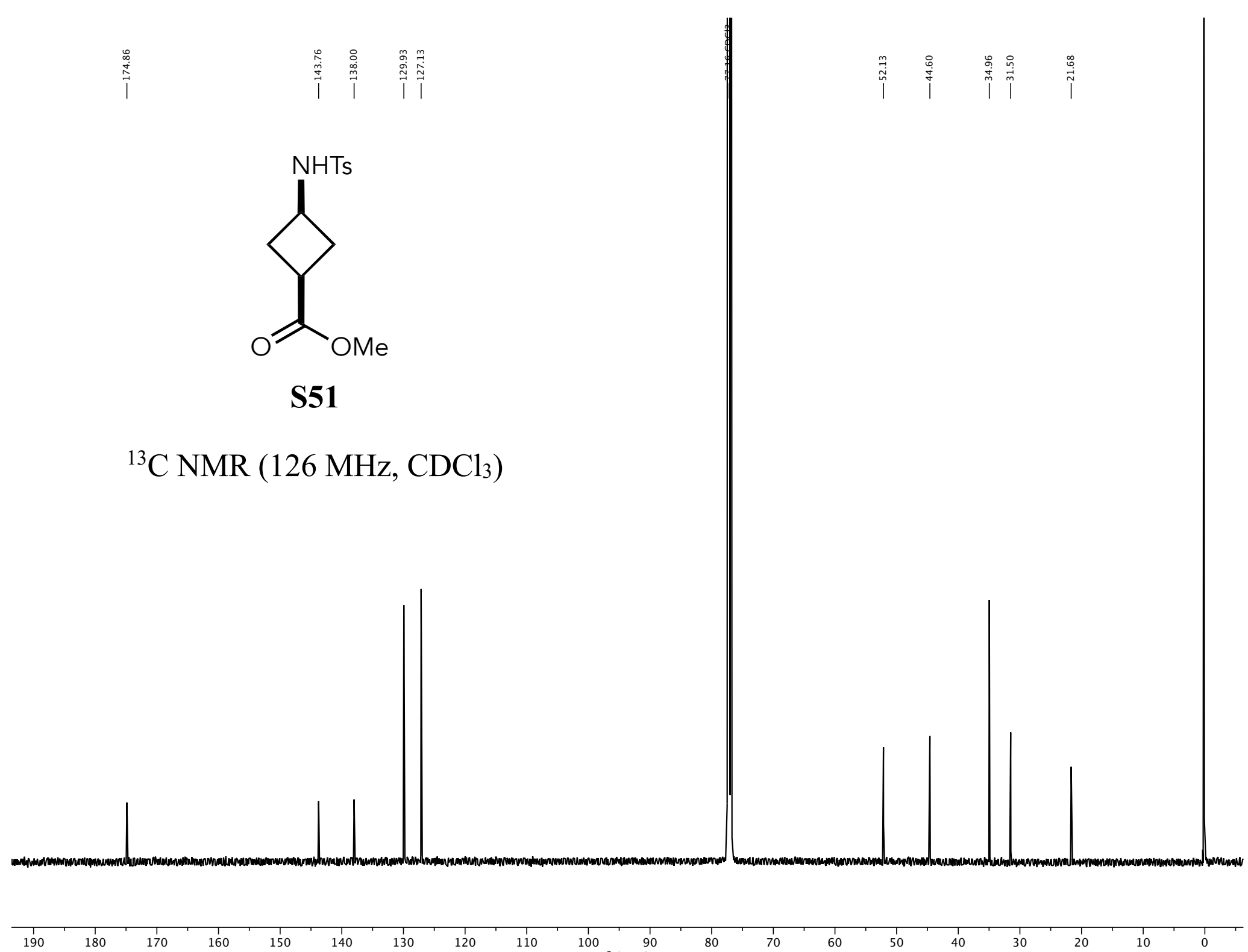
174.86
143.76
138.00
129.93
127.13
52.13
44.60
34.96
31.50
21.68
NHTs
OMe
O
S51
^{13}C NMR (126 MHz, CDCl$_3$)
δ (ppm)

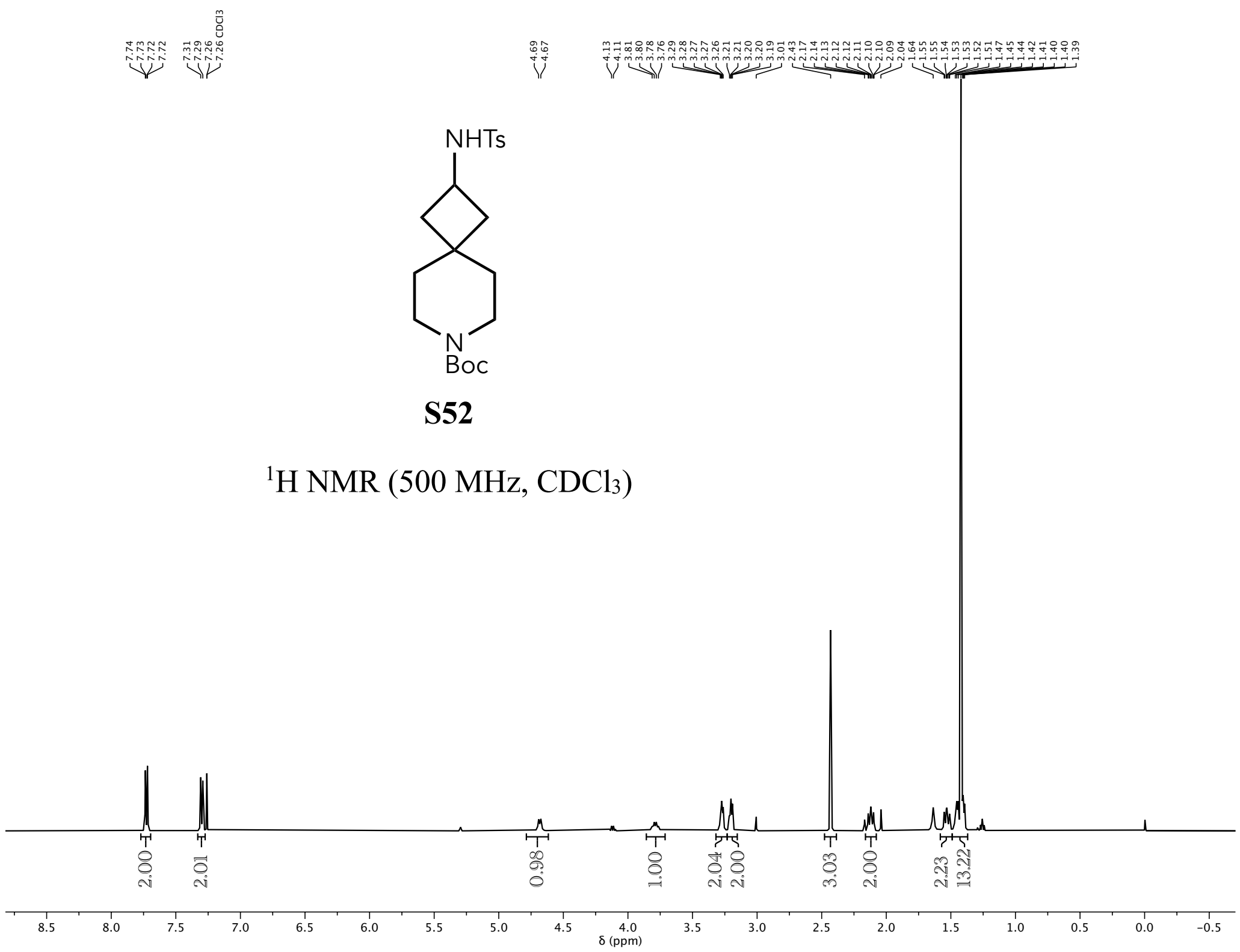

S52

^{1}H NMR (500 MHz, $CDCl_3$)

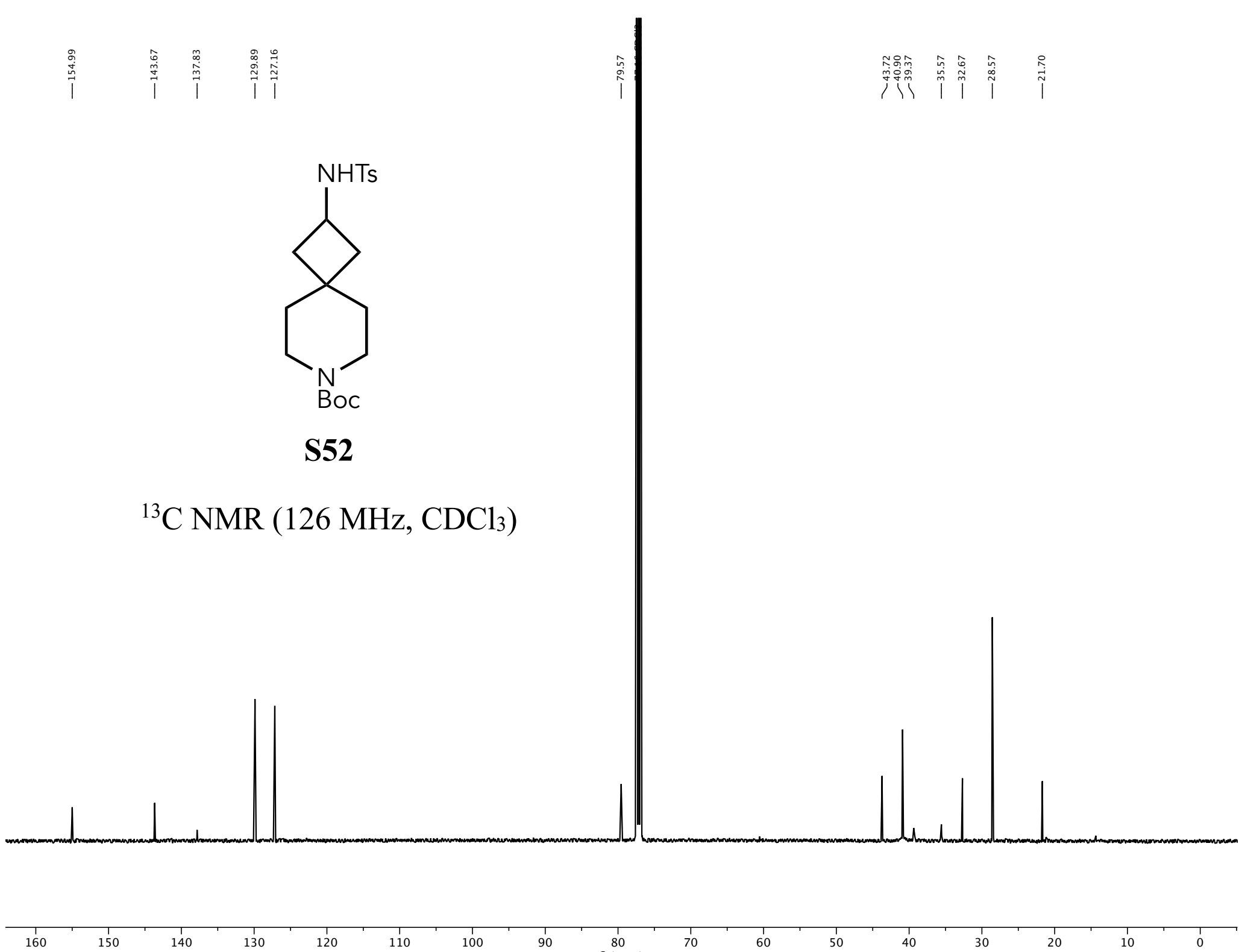

S52

^{13}C NMR (126 MHz, $CDCl_3$)

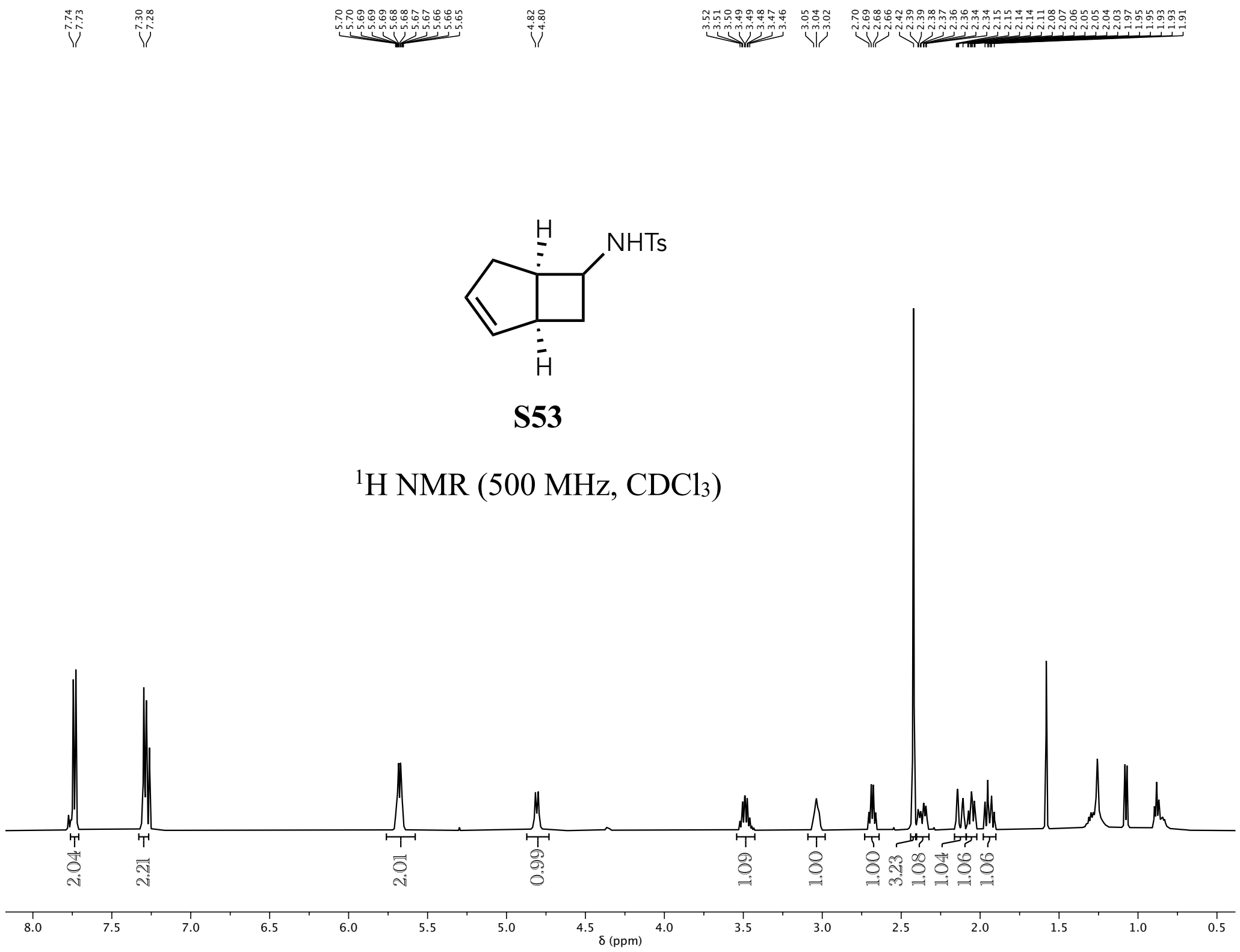

S53

^{1}H NMR (500 MHz, $CDCl_3$)

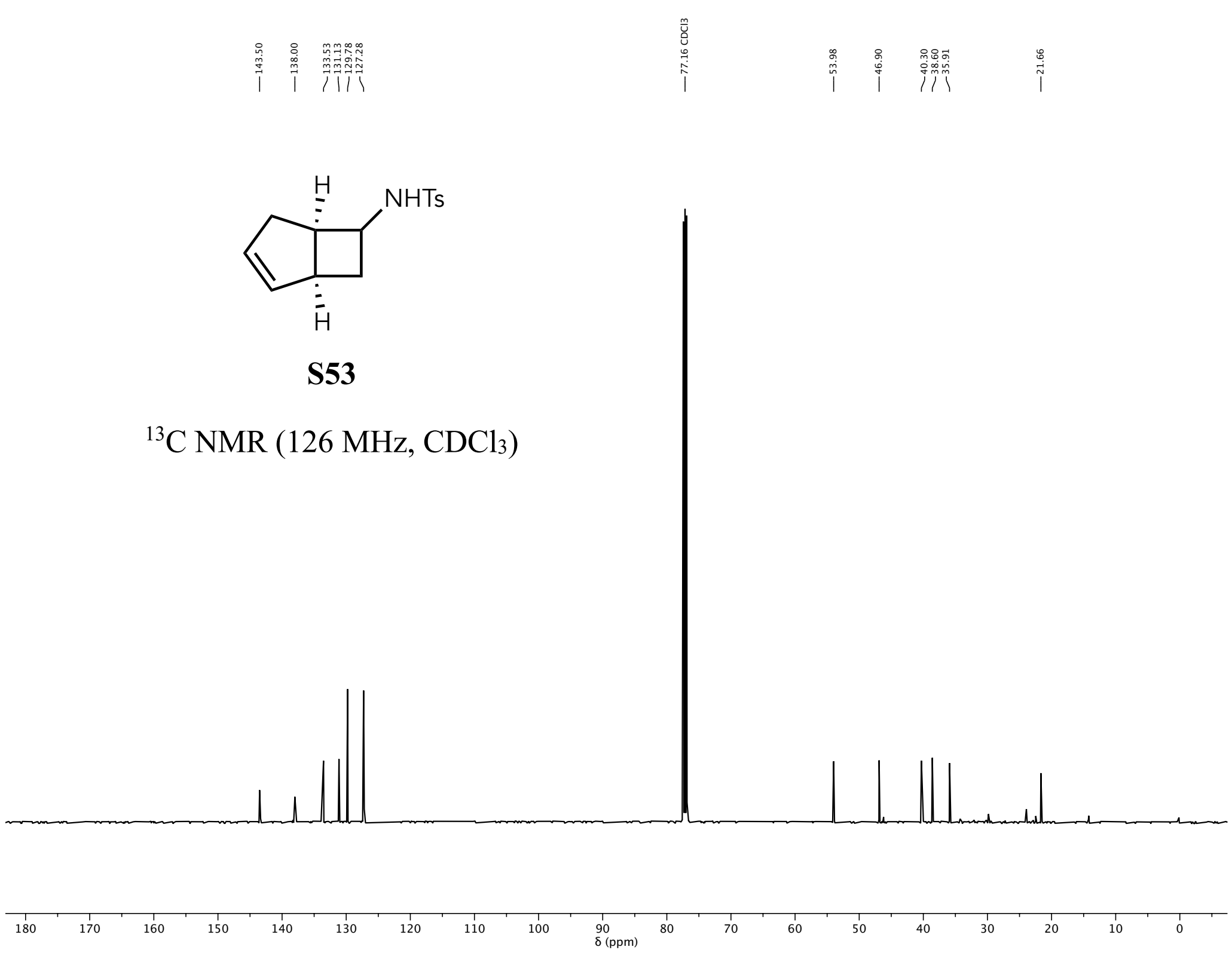

S53

^{13}C NMR (126 MHz, $CDCl_3$)

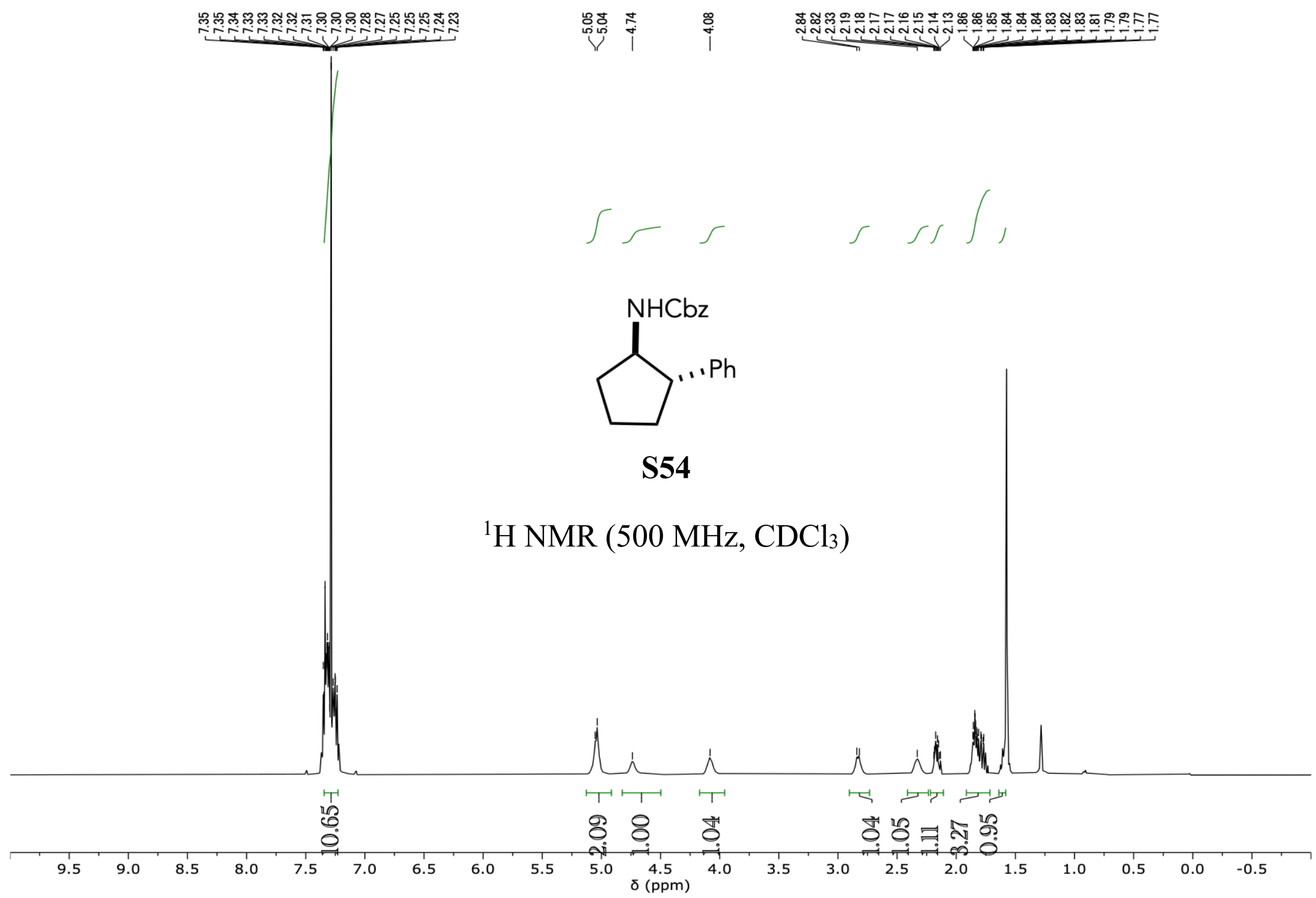

S54

^{1}H NMR (500 MHz, $CDCl_3$)

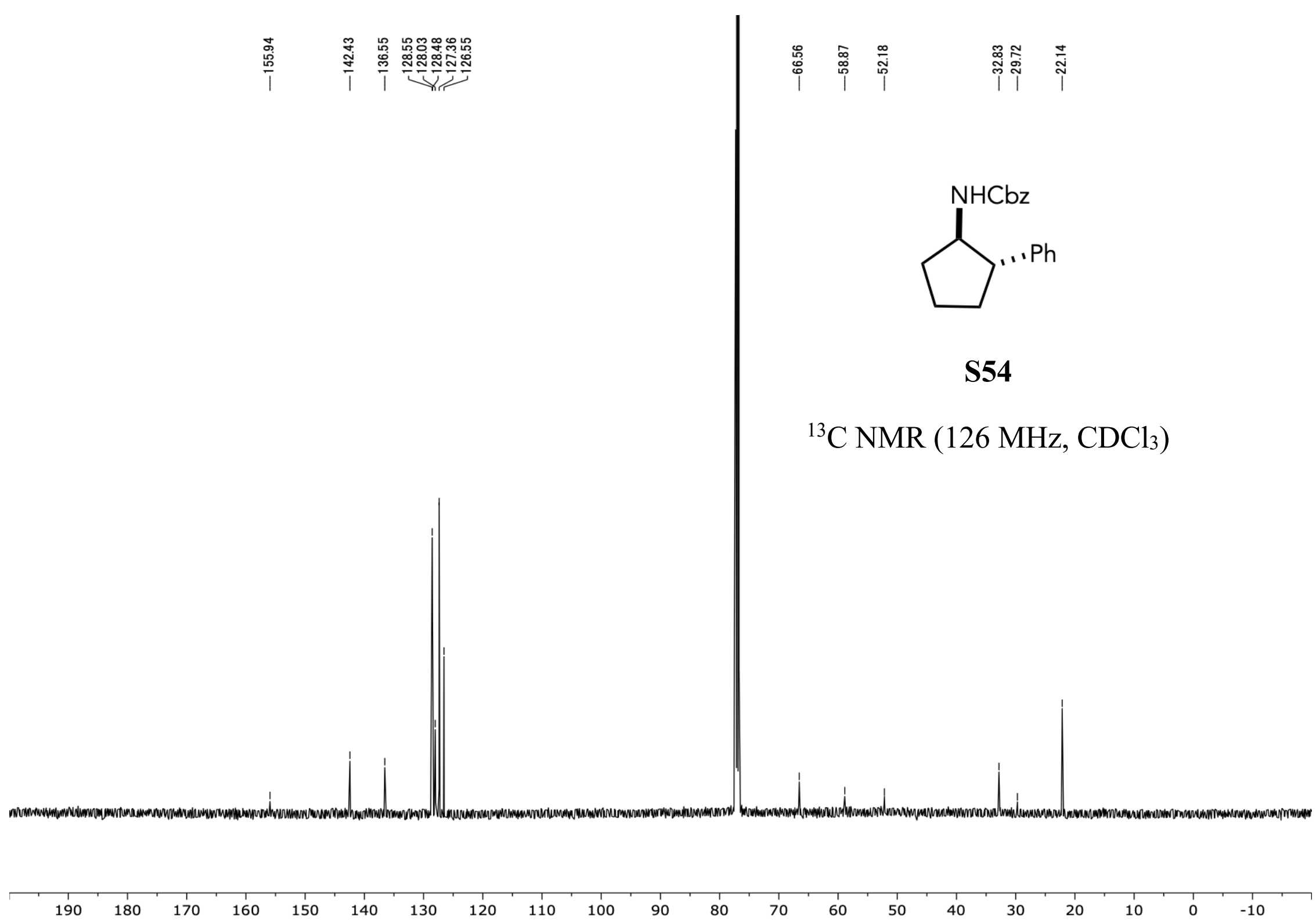

S54

^{13}C NMR (126 MHz, $CDCl_3$)

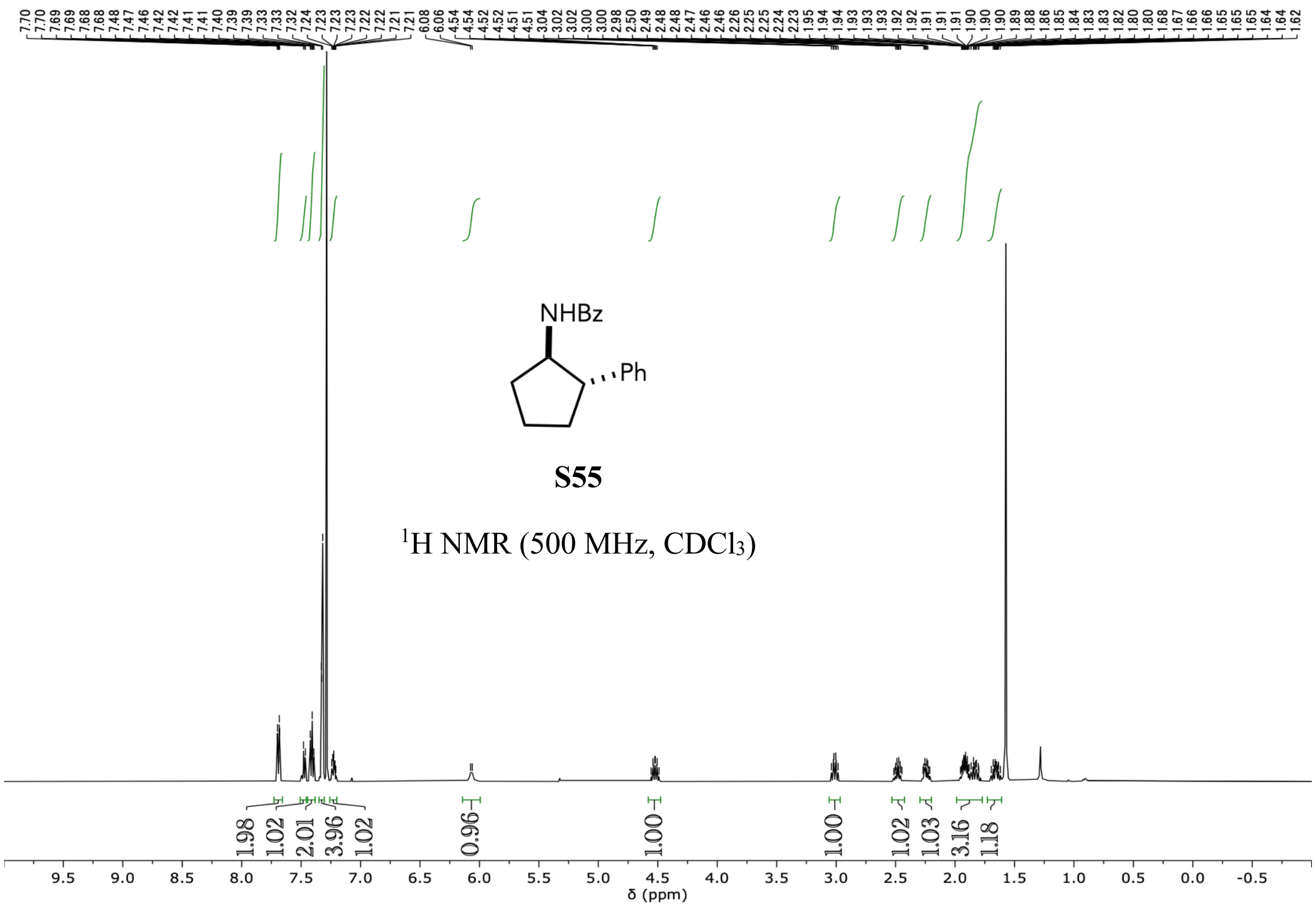
NHBz
Ph
S55
^{1}H NMR (500 MHz, CDCl$_3$)
1.98
1.02
2.01
3.96
1.02
0.96
1.00
1.00
1.02
1.03
3.16
1.18
δ (ppm)

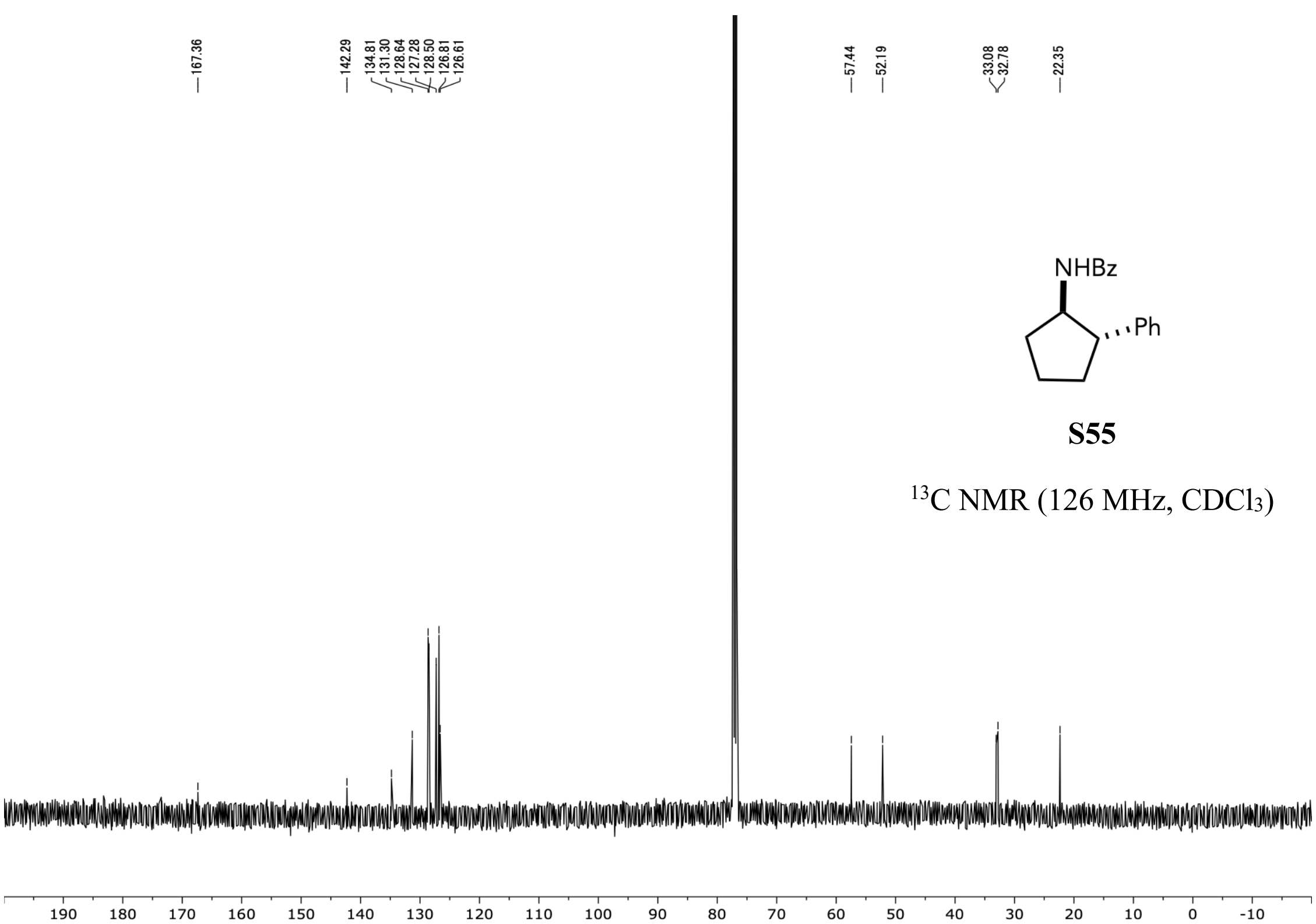
167.36
142.29
134.81
131.30
128.64
127.28
128.50
126.81
126.61
57.44
52.19
33.08
32.78
22.35
NHBz
Ph
S55
^{13}C NMR (126 MHz, CDCl$_3$)
δ (ppm)

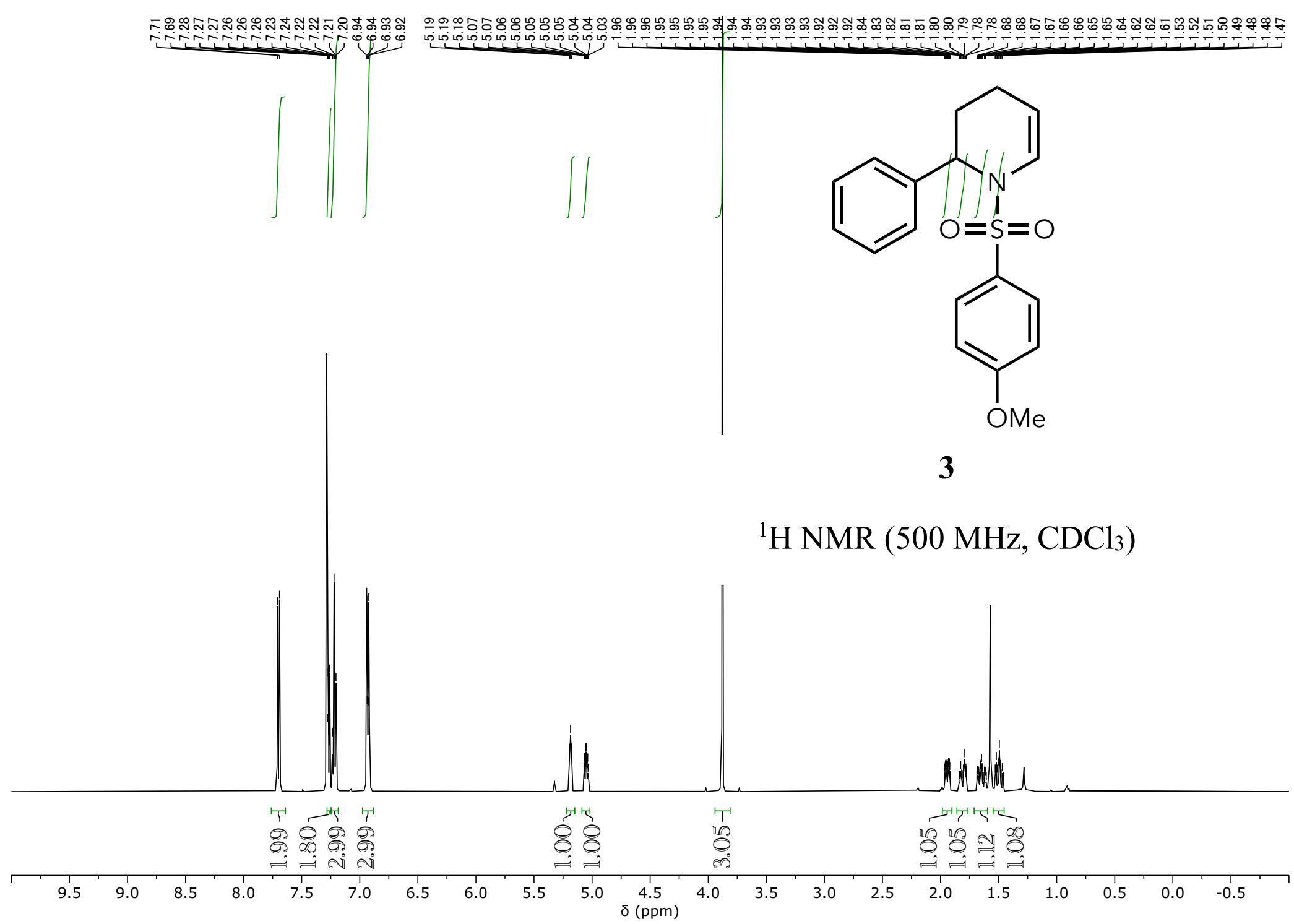

3

^{1}H NMR (500 MHz, $CDCl_3$)

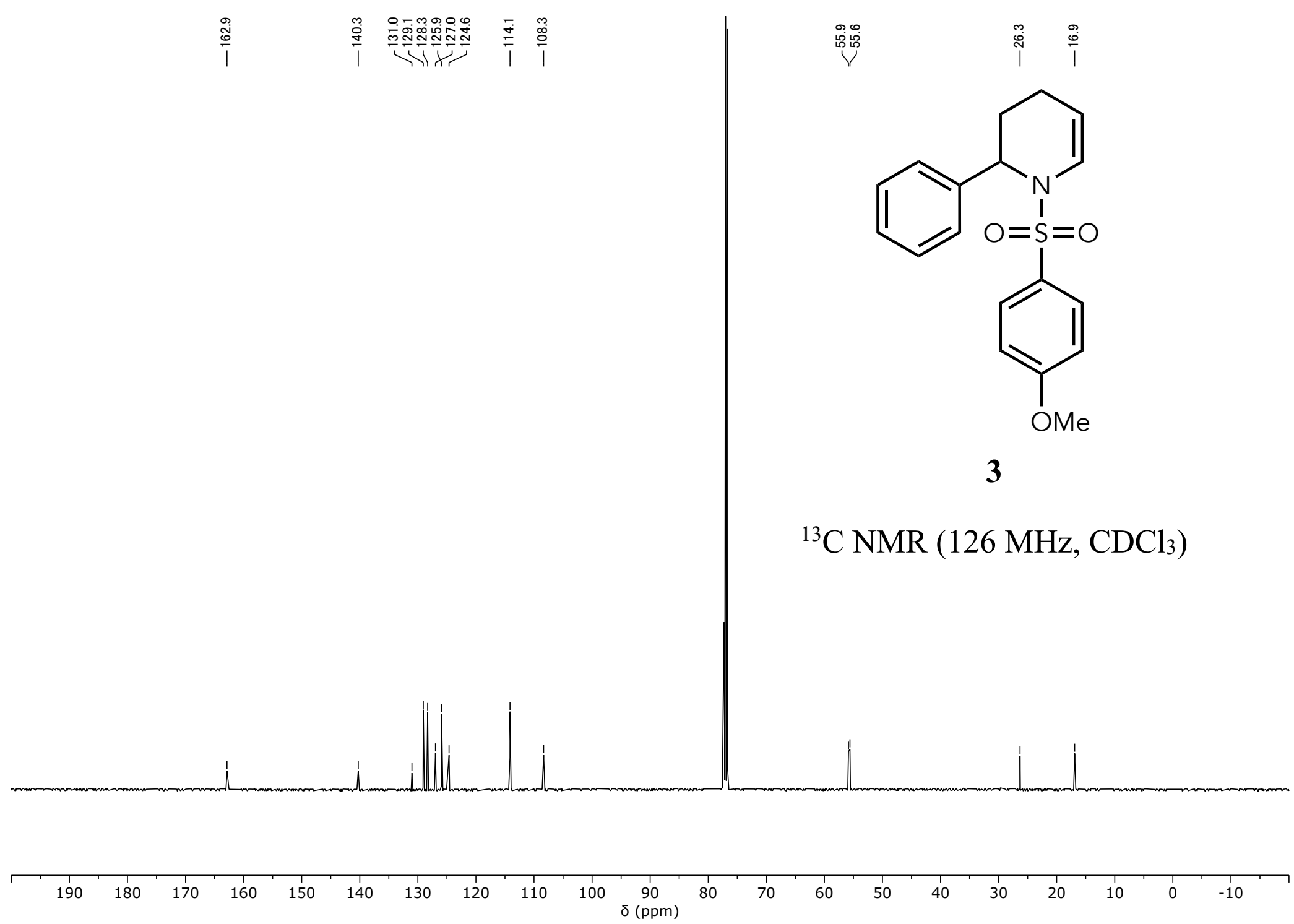

3

^{13}C NMR (126 MHz, $CDCl_3$)

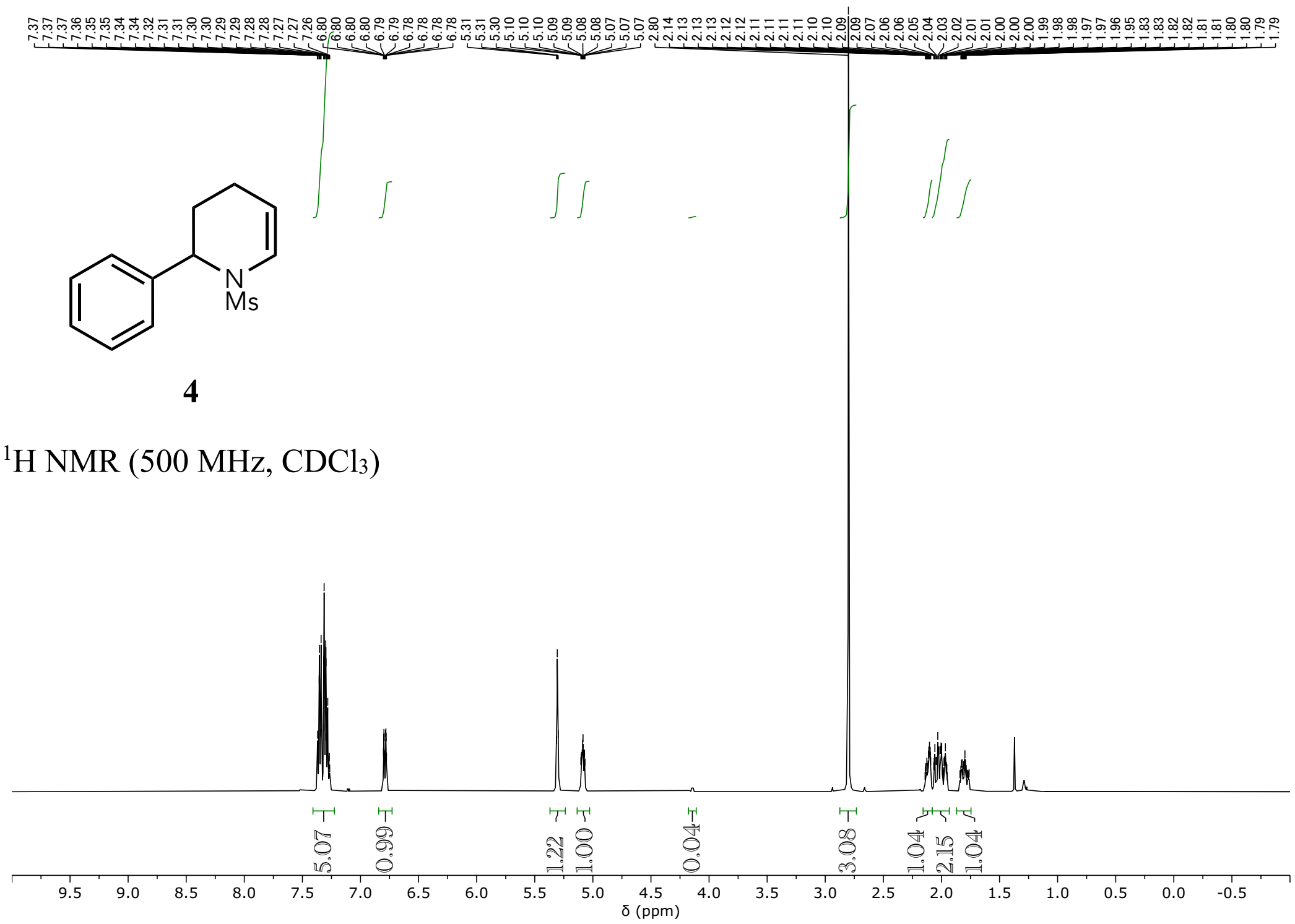

4

^{1}H NMR (500 MHz, $CDCl_3$)

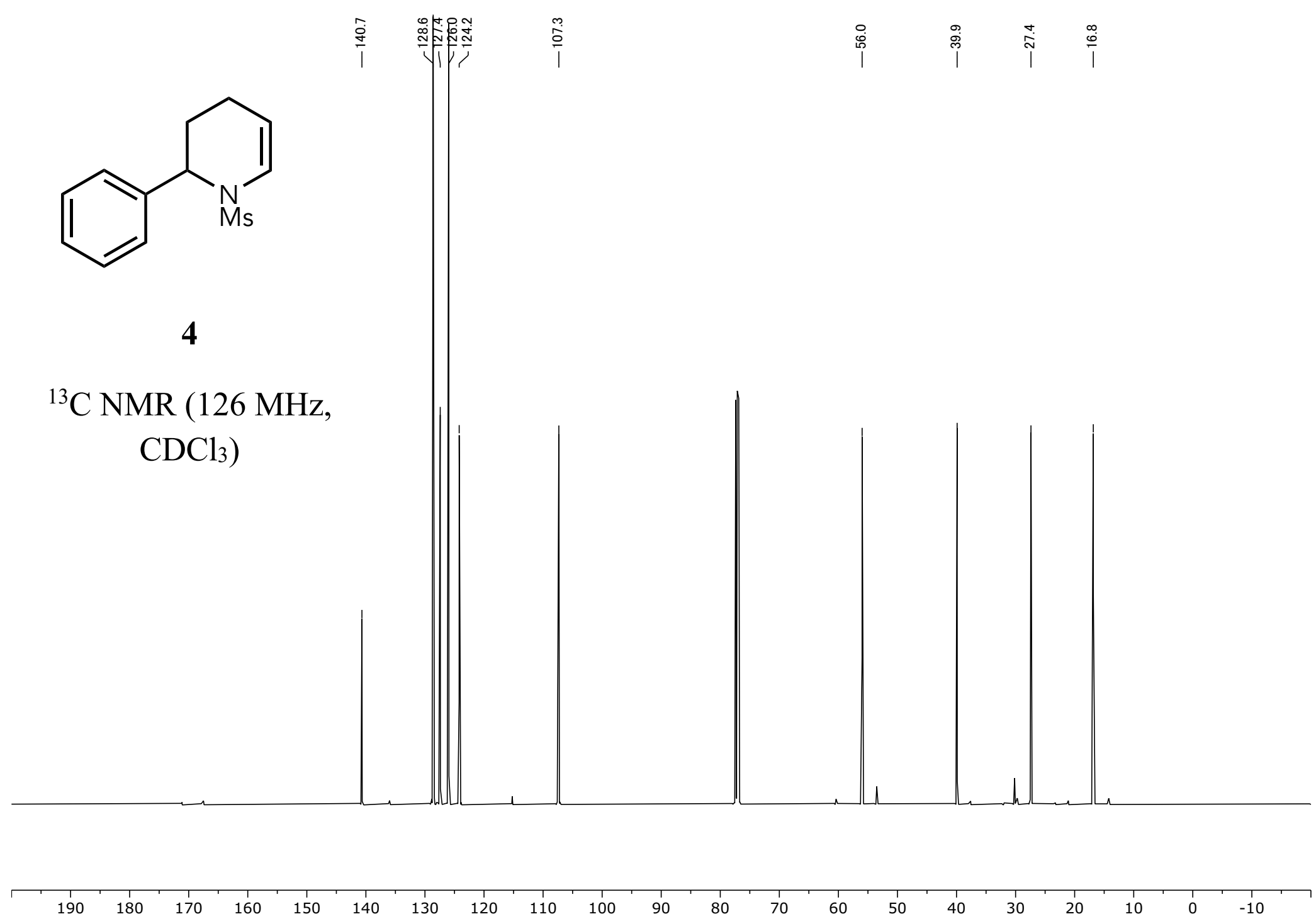

4

^{13}C NMR (126 MHz, $CDCl_3$)

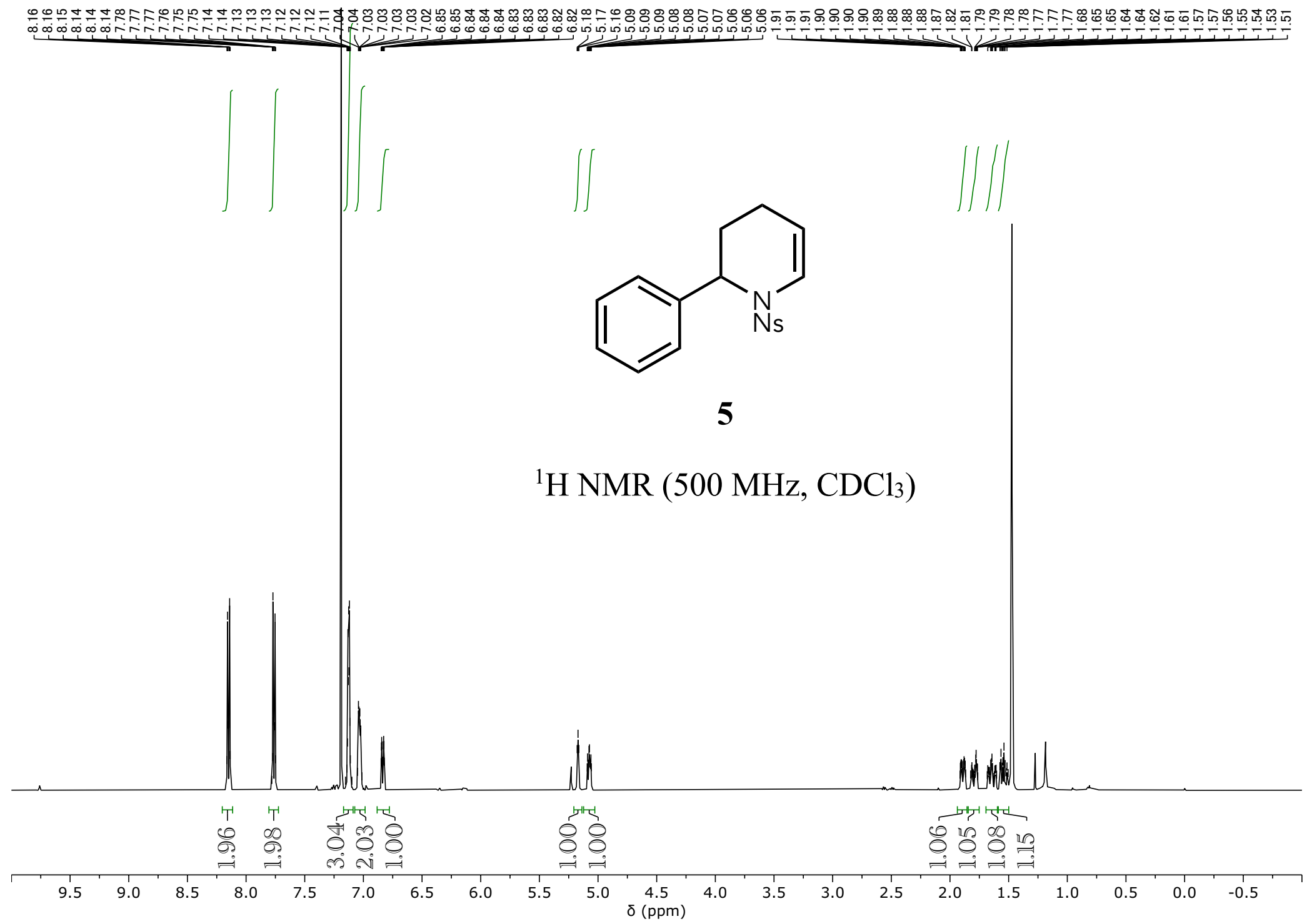
8.16 8.16 8.15 8.14 8.14 7.78 7.77 7.77 7.76 7.75 7.75 7.14 7.14 7.13 7.13 7.13 7.12 7.12 7.11 7.04 7.04 7.03 7.03 7.03 7.02 6.85 6.85 6.84 6.84 6.83 6.83 6.83 6.82 6.82 5.18 5.17 5.16 5.09 5.09 5.09 5.08 5.08 5.07 5.07 5.06 5.06 5.06 1.91 1.91 1.90 1.90 1.90 1.89 1.88 1.88 1.88 1.87 1.82 1.81 1.79 1.79 1.78 1.78 1.77 1.77 1.77 1.68 1.65 1.65 1.64 1.64 1.62 1.61 1.61 1.57 1.56 1.55 1.54 1.53 1.51
N
Ns
5
^{1}H NMR (500 MHz, $CDCl_3$)
1.96
1.98
3.04
2.03
1.00
1.00
1.00
1.06
1.05
1.08
1.15
9.5 9.0 8.5 8.0 7.5 7.0 6.5 6.0 5.5 5.0 4.5 4.0 3.5 3.0 2.5 2.0 1.5 1.0 0.5 0.0 -0.5
δ (ppm)

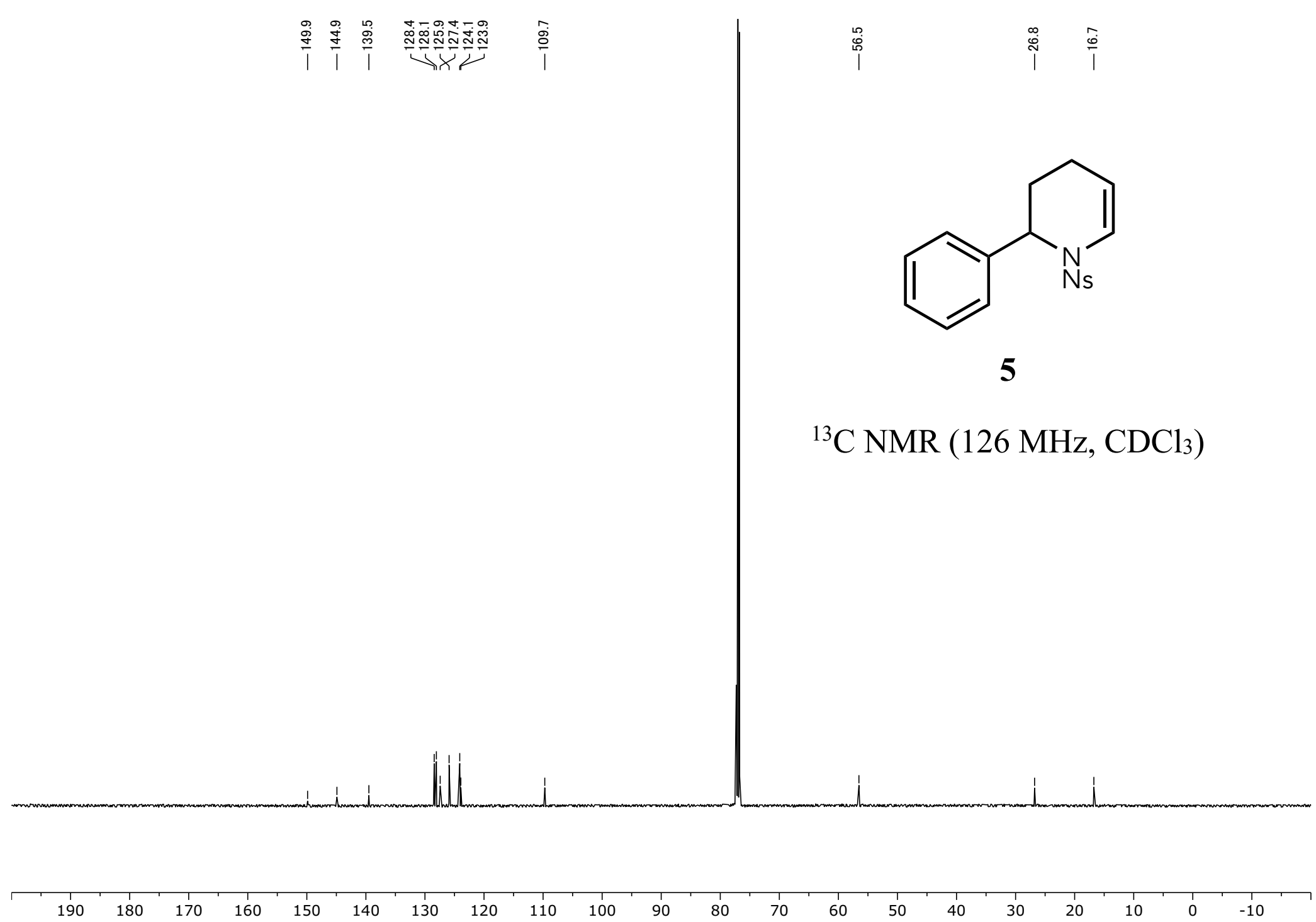
149.9
144.9
139.5
128.4
128.1
125.9
127.4
124.1
123.9
109.7
56.5
26.8
16.7
N
Ns
5
^{13}C NMR (126 MHz, $CDCl_3$)
190 180 170 160 150 140 130 120 110 100 90 80 70 60 50 40 30 20 10 0 -10
δ (ppm)

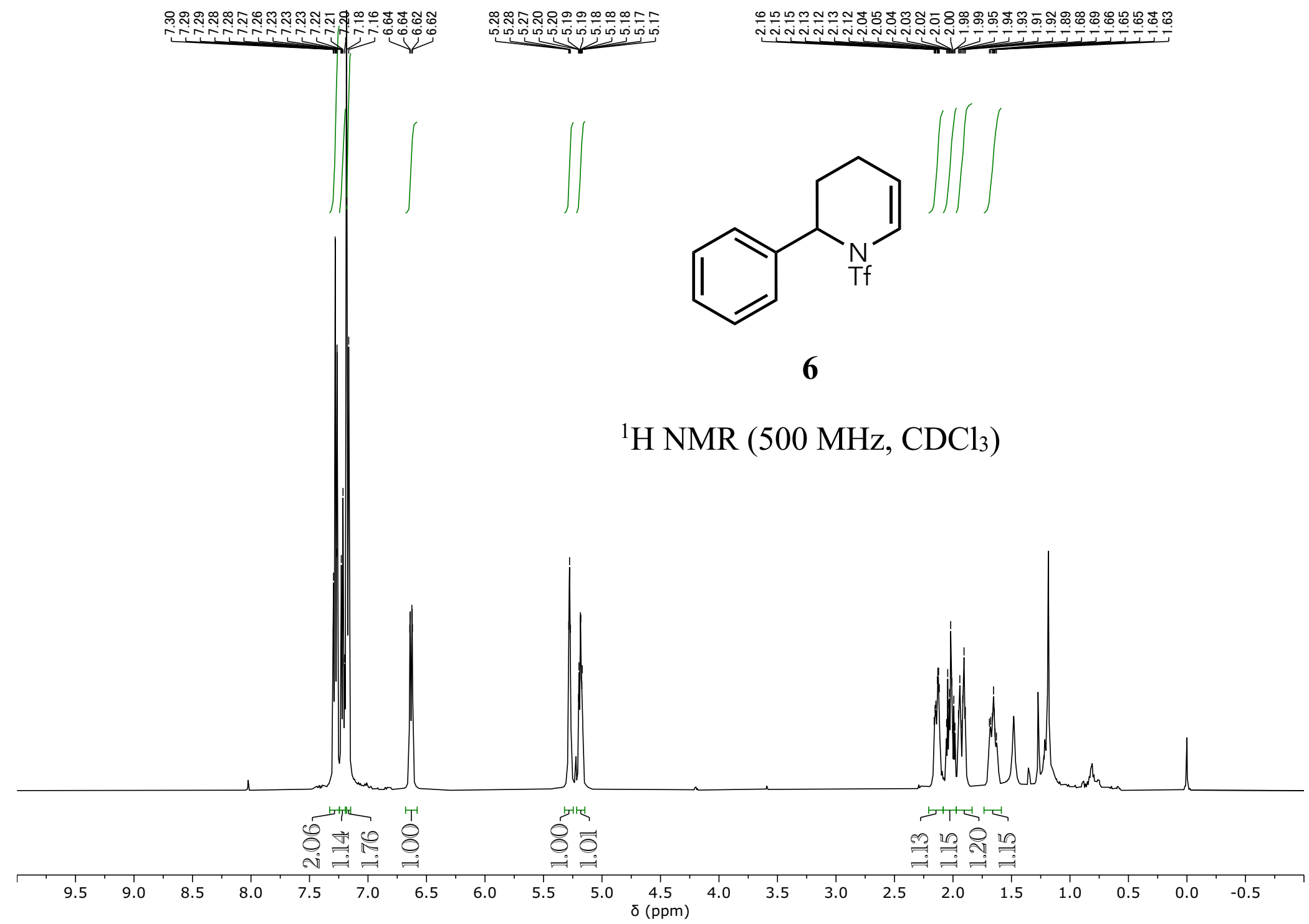

7.30 7.29 7.29 7.28 7.28 7.27 7.26 7.23 7.23 7.23 7.22 7.21 7.20 7.18 7.16 6.64 6.64 6.62 6.62
5.28 5.28 5.27 5.20 5.20 5.19 5.18 5.18 5.18 5.17 5.17
2.16 2.15 2.13 2.12 2.13 2.12 2.04 2.05 2.04 2.03 2.02 2.01 2.00 1.98 1.99 1.95 1.94 1.93 1.91 1.92 1.89 1.68 1.69 1.66 1.65 1.64 1.63
N
Tf
6
^{1}H NMR (500 MHz, $CDCl_3$)
2.06 1.14 1.76 1.00 1.00 1.01 1.13 1.15 1.20 1.15
δ (ppm)

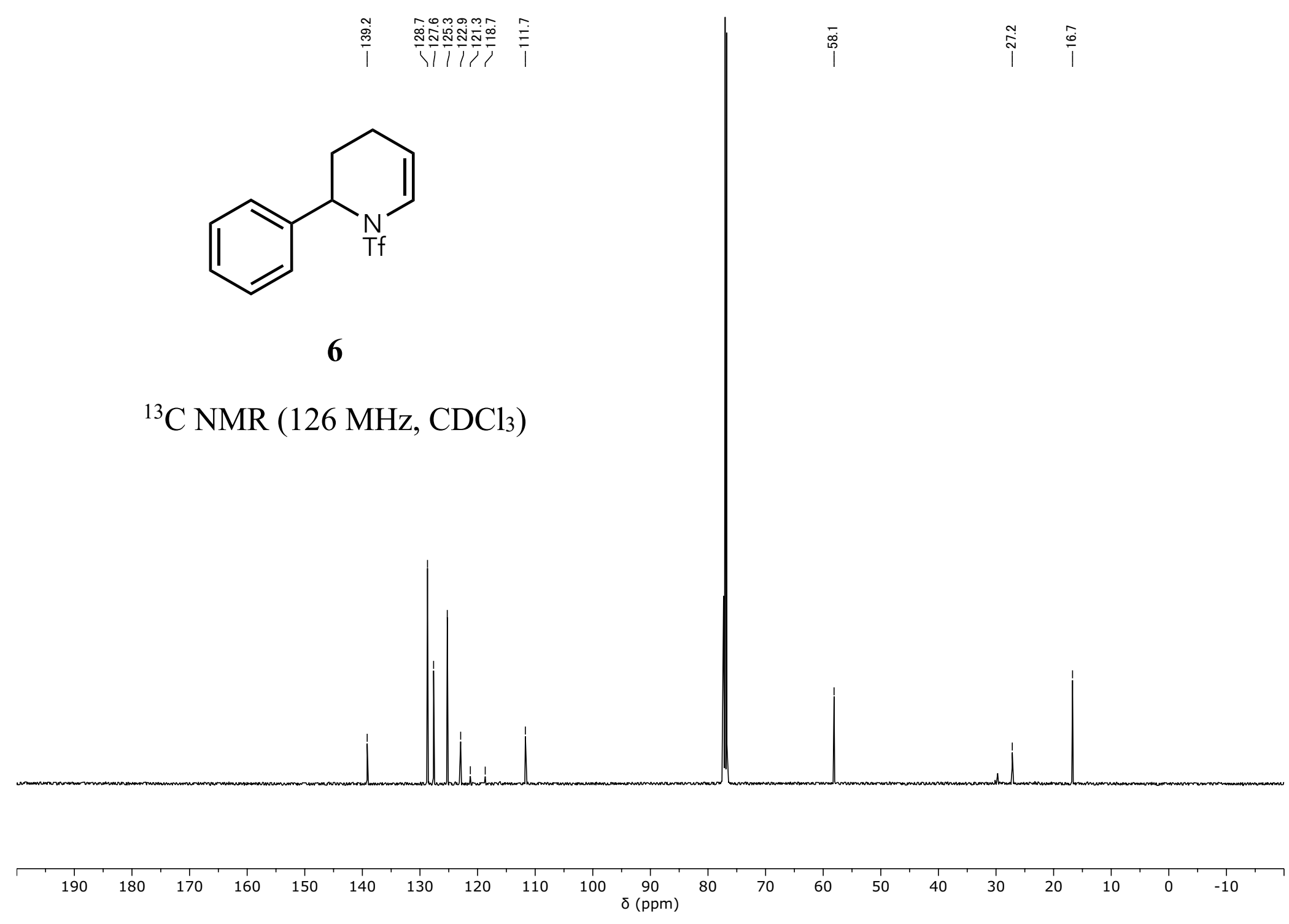

139.2
128.7 127.6 125.3 122.9 121.3 118.7
111.7
58.1
27.2
16.7
N
Tf
6
^{13}C NMR (126 MHz, $CDCl_3$)
δ (ppm)

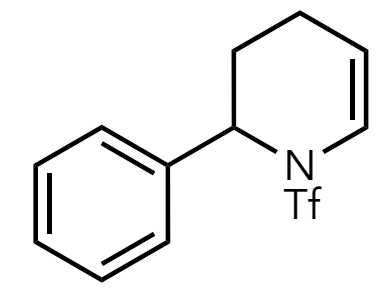

6

^{19}F NMR (377 MHz, $CDCl_3$)

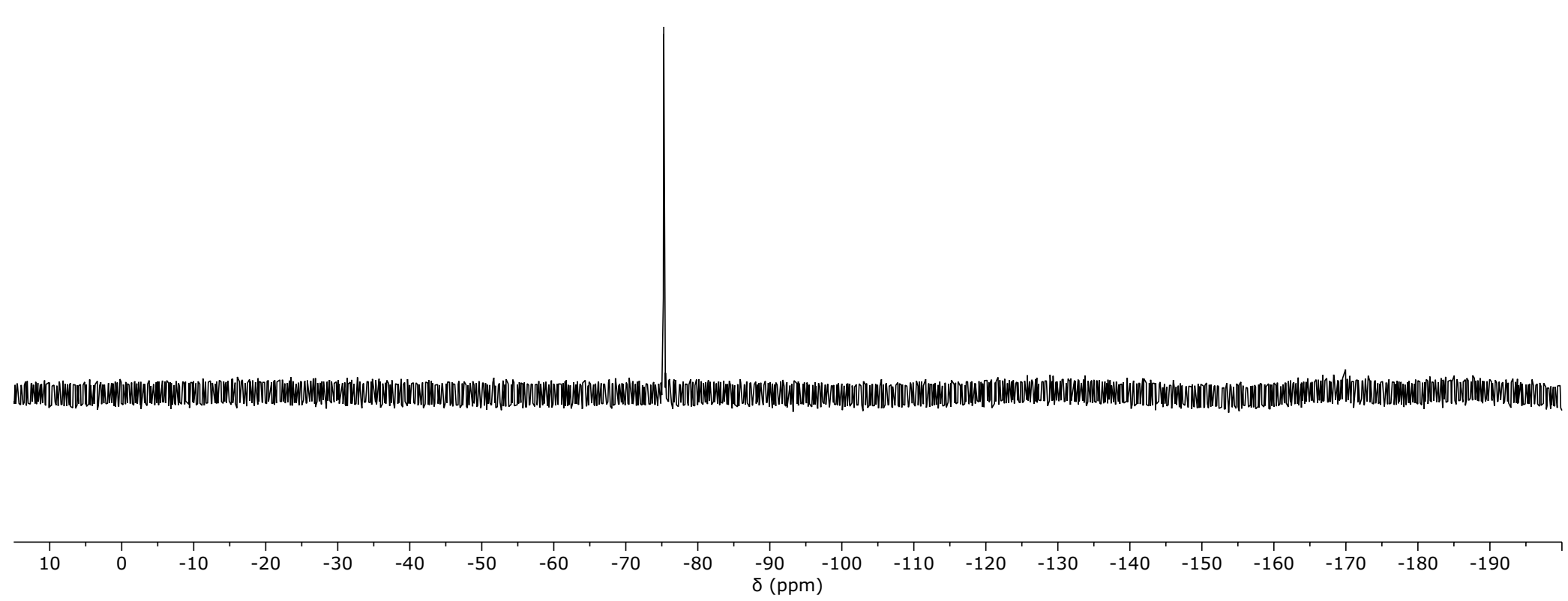

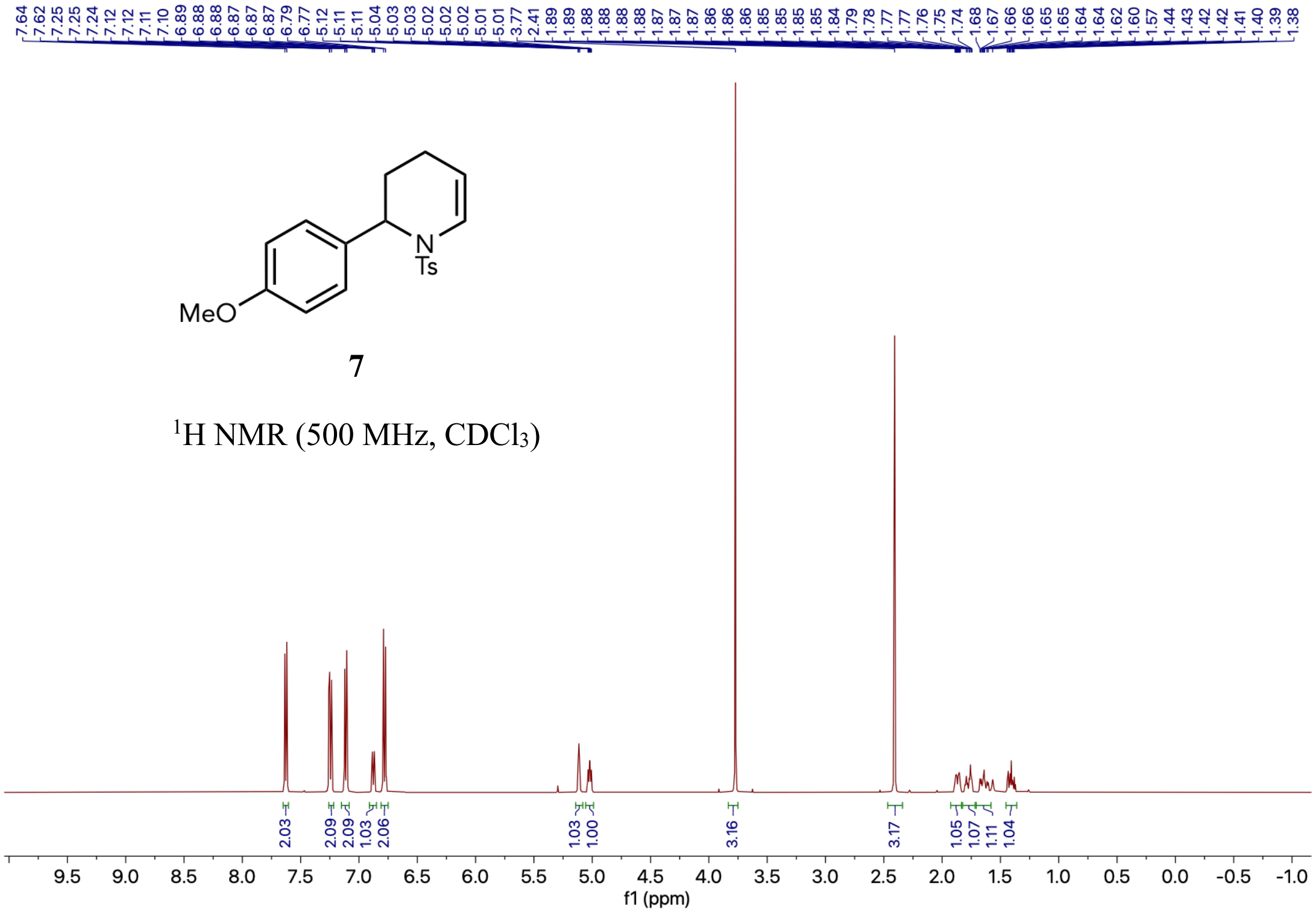

7

^{1}H NMR (500 MHz, $CDCl_3$)

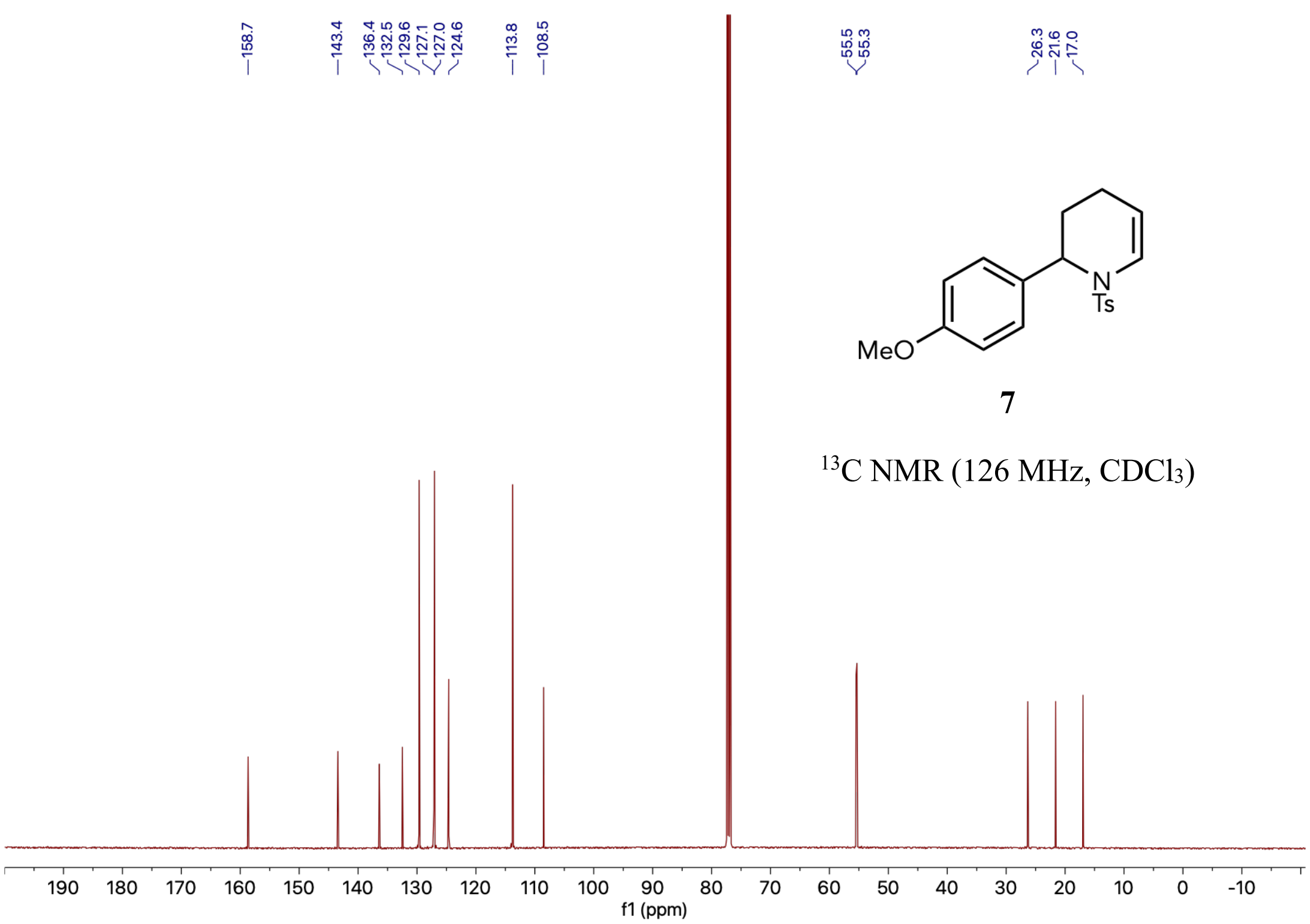

7

^{13}C NMR (126 MHz, $CDCl_3$)

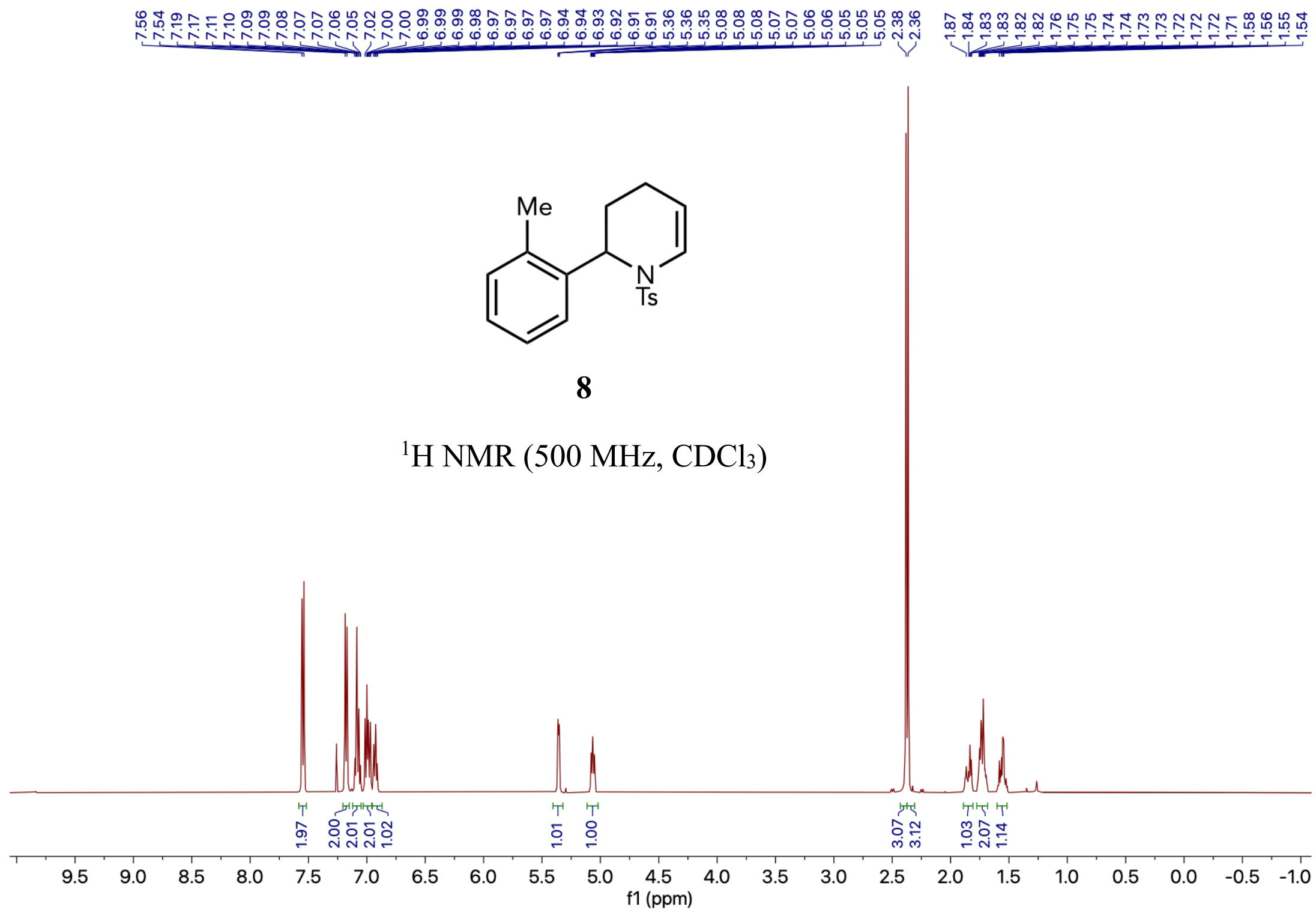
7.56 7.54 7.19 7.17 7.11 7.10 7.09 7.09 7.08 7.07 7.07 7.06 7.05 7.02 7.00 7.00 6.99 6.99 6.99 6.98 6.97 6.97 6.97 6.97 6.94 6.94 6.93 6.92 6.91 6.91 5.36 5.36 5.35 5.35 5.08 5.08 5.08 5.07 5.07 5.06 5.06 5.05 5.05 5.05 2.38 2.36 1.87 1.84 1.83 1.83 1.82 1.82 1.76 1.75 1.75 1.74 1.74 1.73 1.73 1.72 1.72 1.72 1.71 1.58 1.56 1.55 1.54
Me
N
Ts
8
^{1}H NMR (500 MHz, CDCl$_3$)
1.97 2.00 2.01 2.01 1.02 1.01 1.00 3.07 3.12 1.03 2.07 1.14
f1 (ppm)

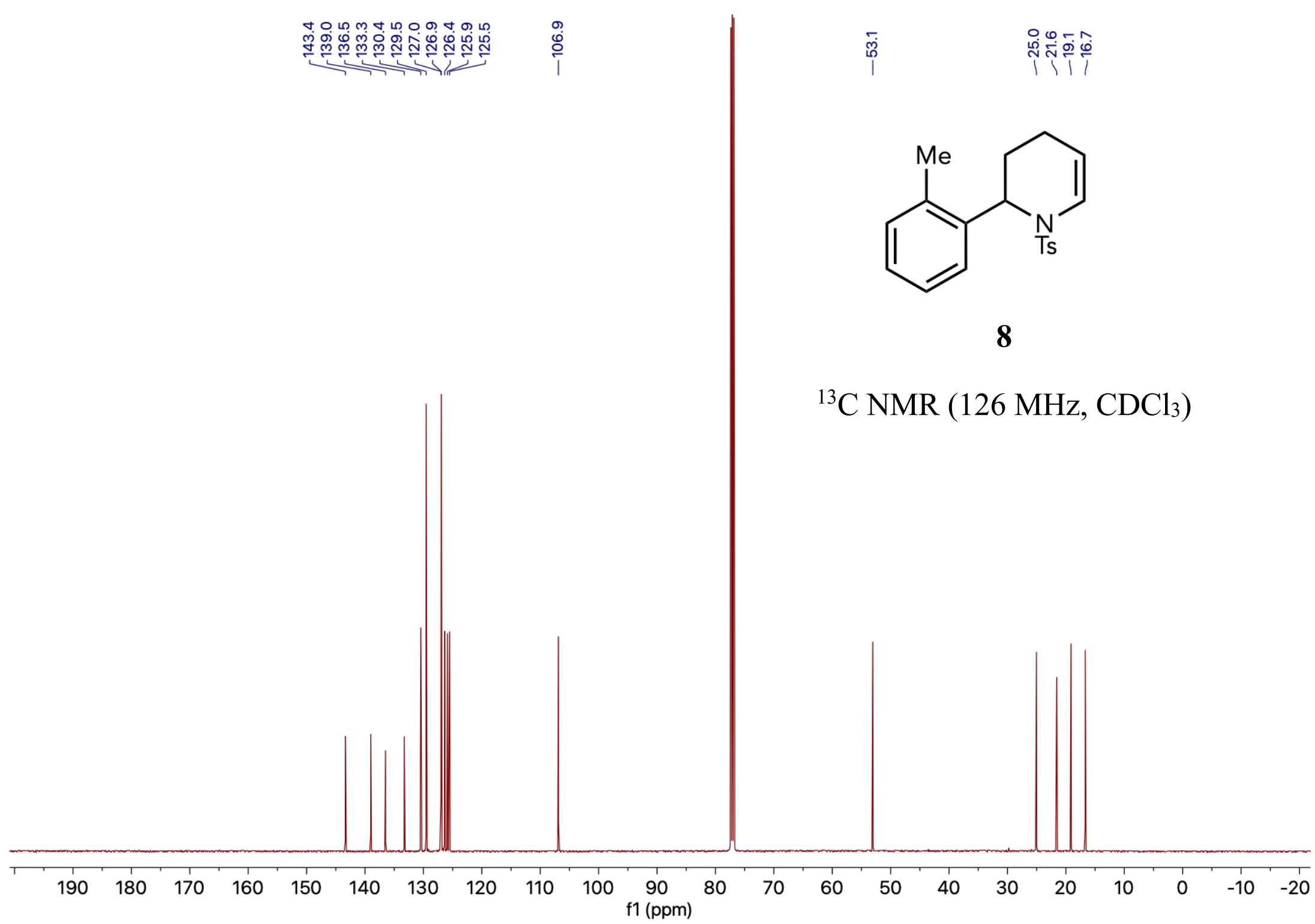
143.4 139.0 136.5 133.3 130.4 129.5 127.0 126.9 126.4 125.9 125.5 106.9 53.1 25.0 21.6 19.1 16.7
Me
N
Ts
8
^{13}C NMR (126 MHz, CDCl$_3$)
f1 (ppm)

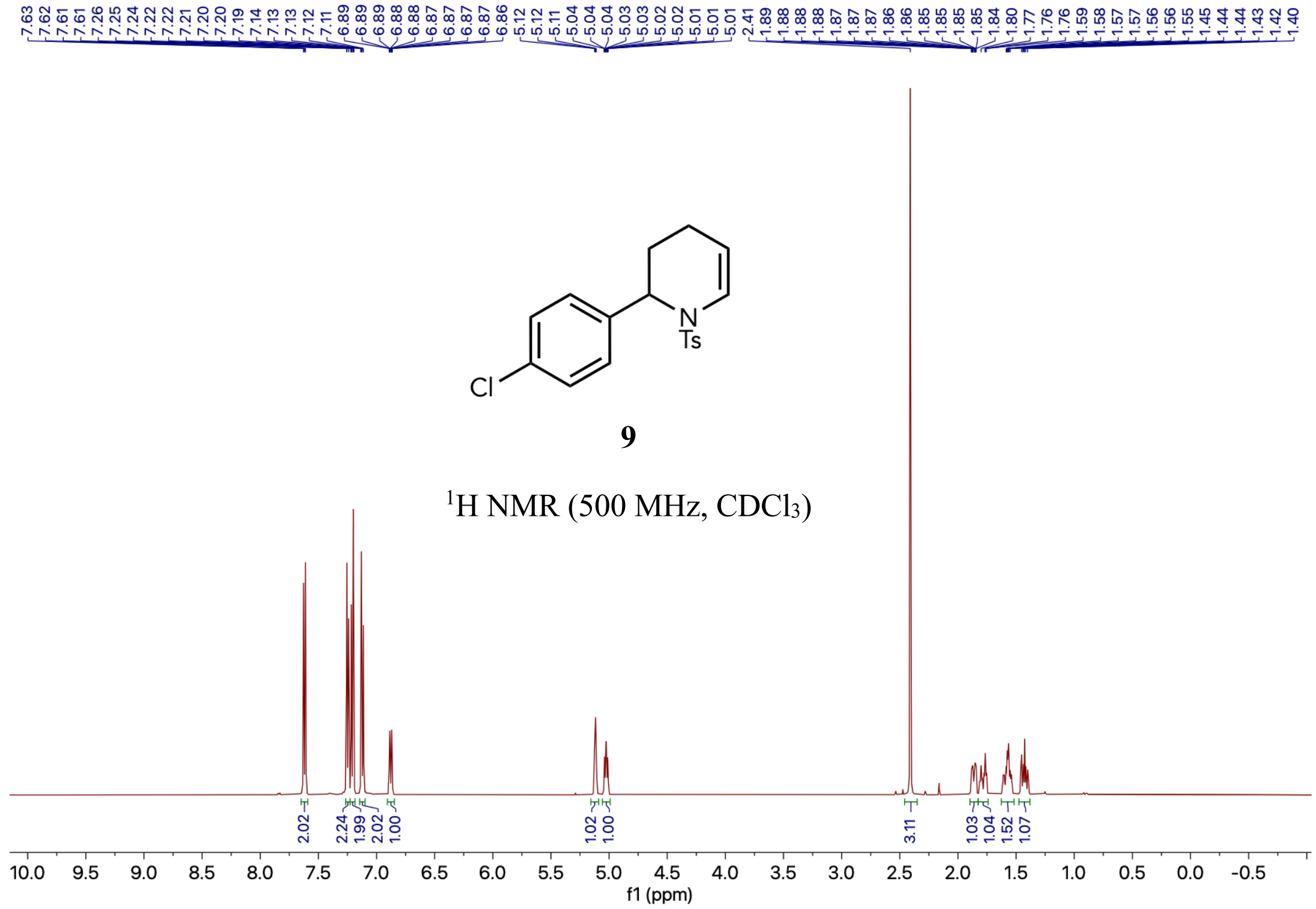
7.63 7.62 7.61 7.26 7.25 7.24 7.22 7.22 7.21 7.20 7.20 7.19 7.14 7.13 7.13 7.12 7.11 6.89 6.89 6.89 6.88 6.88 6.87 6.87 6.87 6.87 6.86
5.12 5.12 5.11 5.04 5.04 5.04 5.03 5.03 5.02 5.02 5.01 5.01 5.01
2.41 1.89 1.88 1.88 1.88 1.87 1.87 1.87 1.86 1.86 1.85 1.85 1.85 1.85 1.84 1.80 1.77 1.76 1.76 1.59 1.58 1.57 1.57 1.56 1.56 1.55 1.45 1.44 1.44 1.43 1.42 1.40
Cl
N
Ts
9
1H NMR (500 MHz, CDCl3)
2.02 2.24 1.99 2.02 1.00 1.02 1.00 3.11 1.03 1.04 1.52 1.07
f1 (ppm)

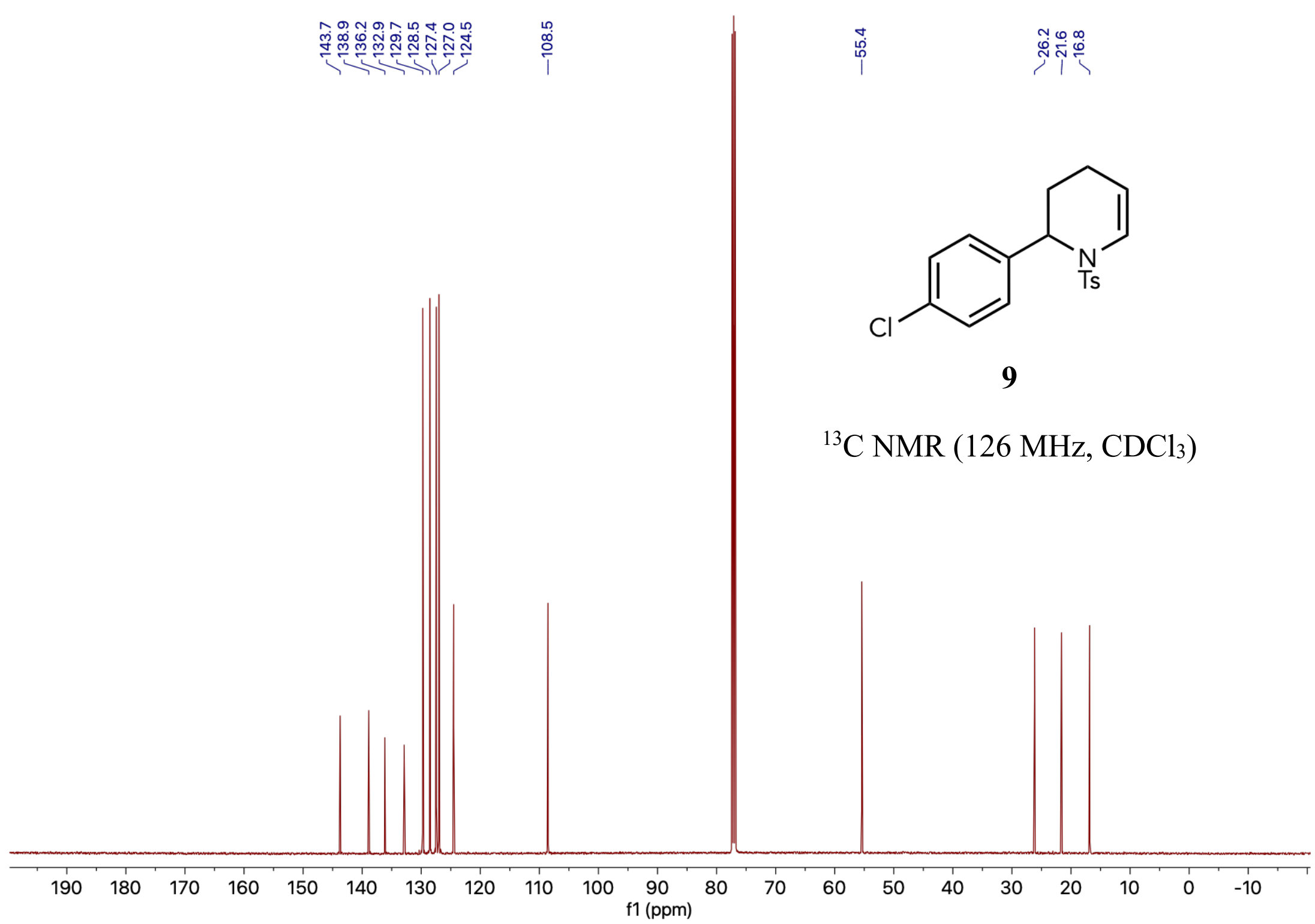
143.7 138.9 136.2 132.9 129.7 128.5 127.4 127.0 124.5
108.5
55.4
26.2 21.6 16.8
Cl
N
Ts
9
13C NMR (126 MHz, CDCl3)
f1 (ppm)

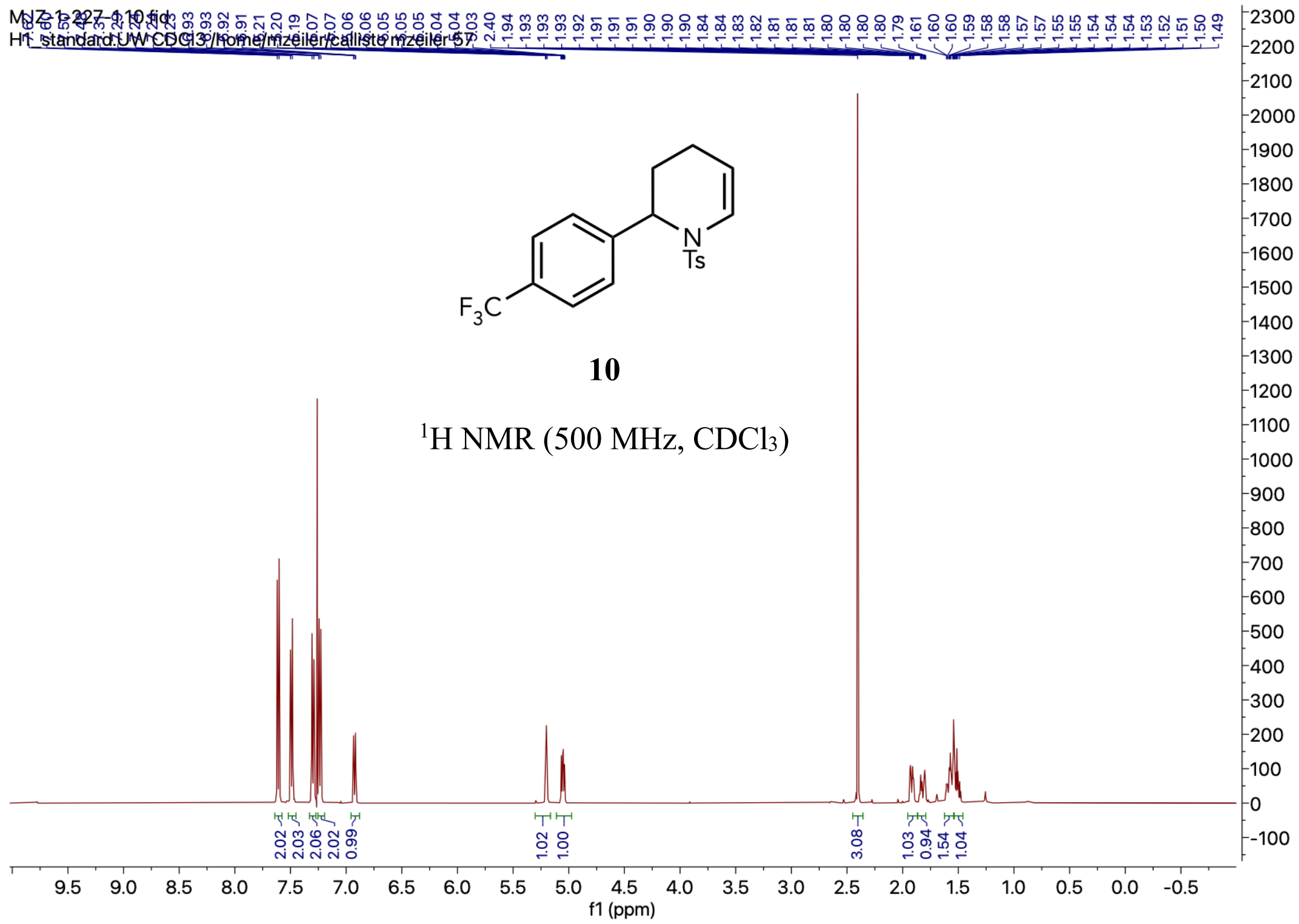

^{1}H NMR (500 MHz, $CDCl_3$)

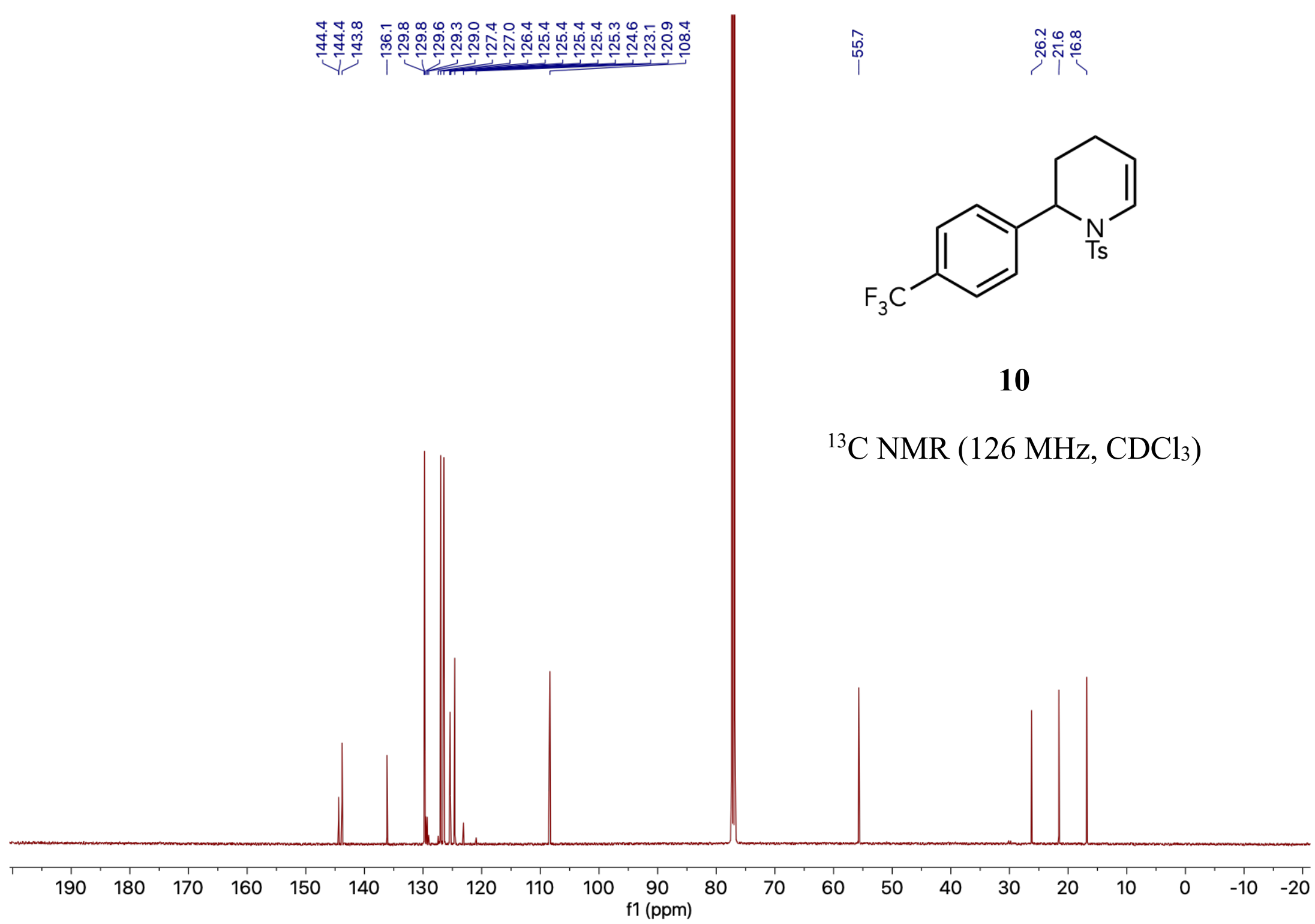

^{13}C NMR (126 MHz, $CDCl_3$)

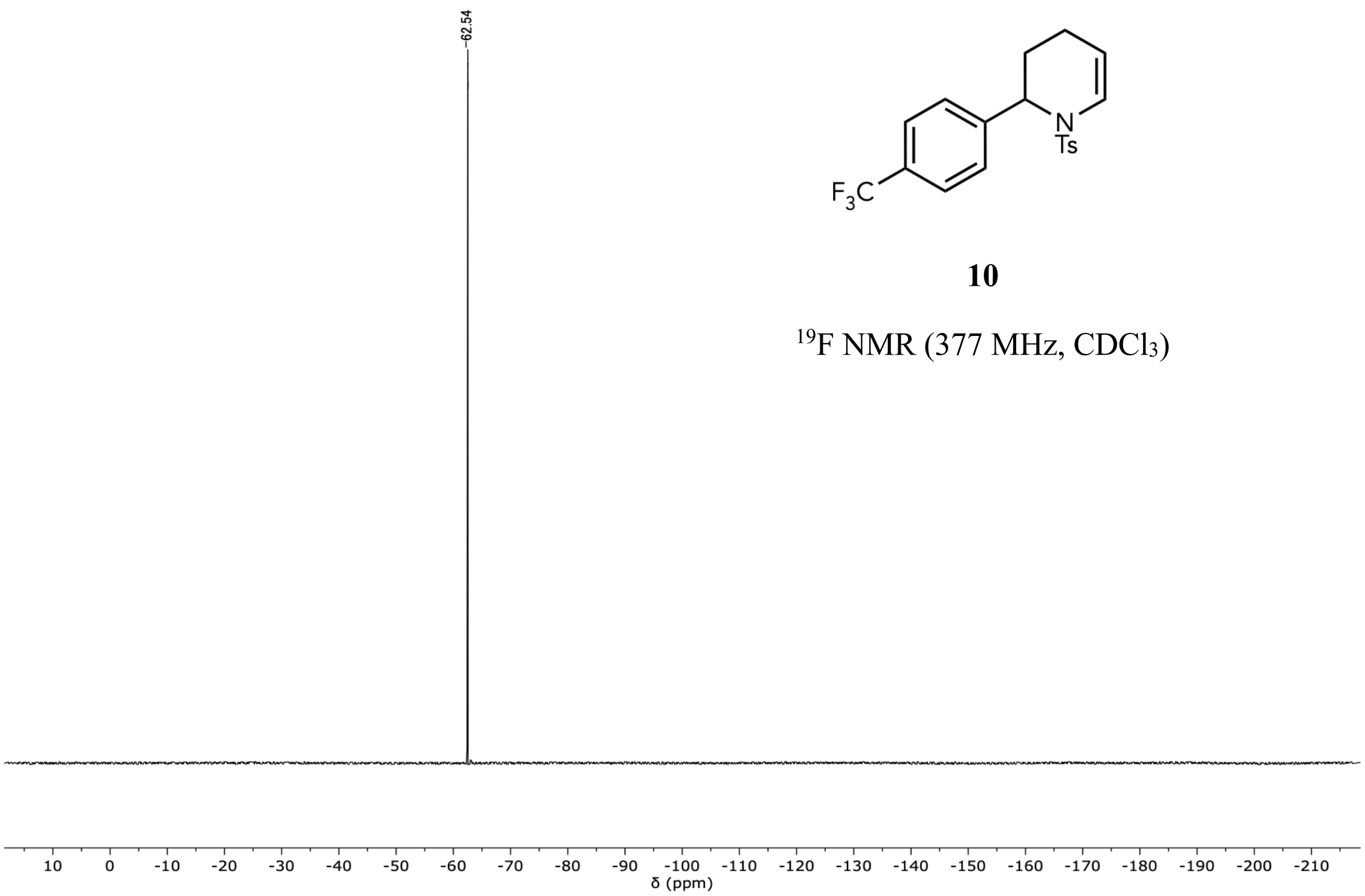

10

^{19}F NMR (377 MHz, $CDCl_3$)

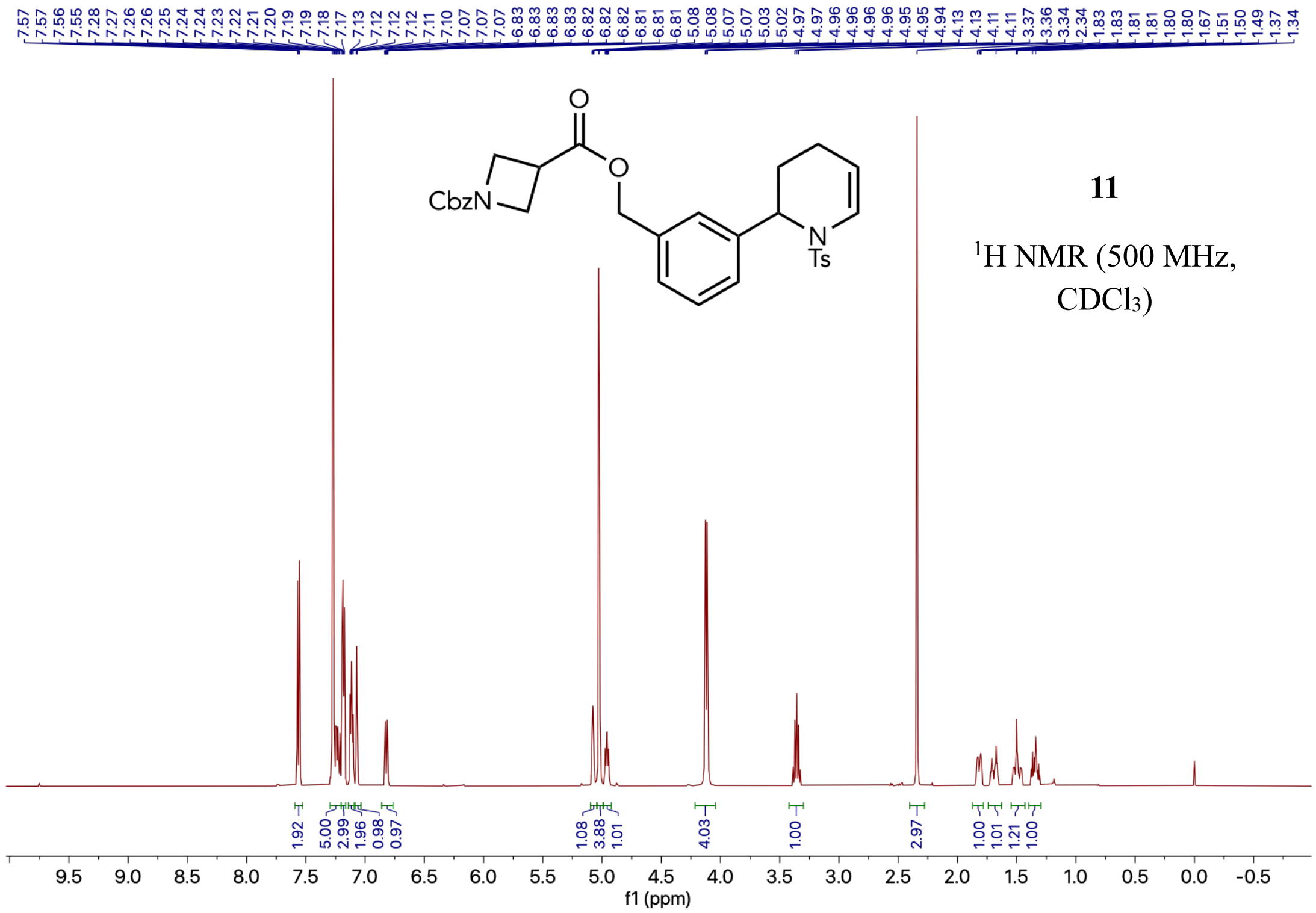
7.57 7.57 7.56 7.55 7.28 7.27 7.26 7.26 7.25 7.24 7.24 7.23 7.22 7.21 7.20 7.19 7.19 7.18 7.17 7.13 7.12 7.12 7.12 7.11 7.10 7.07 7.07 7.07 6.83 6.83 6.83 6.83 6.82 6.82 6.82 6.81 6.81 6.81 6.81 5.08 5.08 5.07 5.07 5.03 5.02 4.97 4.97 4.96 4.96 4.96 4.96 4.95 4.95 4.94 4.13 4.13 4.11 4.11 3.37 3.36 3.34 2.34 1.83 1.83 1.81 1.81 1.80 1.67 1.51 1.50 1.49 1.37 1.34
CbzN
O
O
N
Ts
11
1H NMR (500 MHz, CDCl3)
1.92 5.00 2.99 1.96 0.98 0.97 1.08 3.88 1.01 4.03 1.00 2.97 1.00 1.01 1.21 1.00
9.5 9.0 8.5 8.0 7.5 7.0 6.5 6.0 5.5 5.0 4.5 4.0 3.5 3.0 2.5 2.0 1.5 1.0 0.5 0.0 -0.5
f1 (ppm)

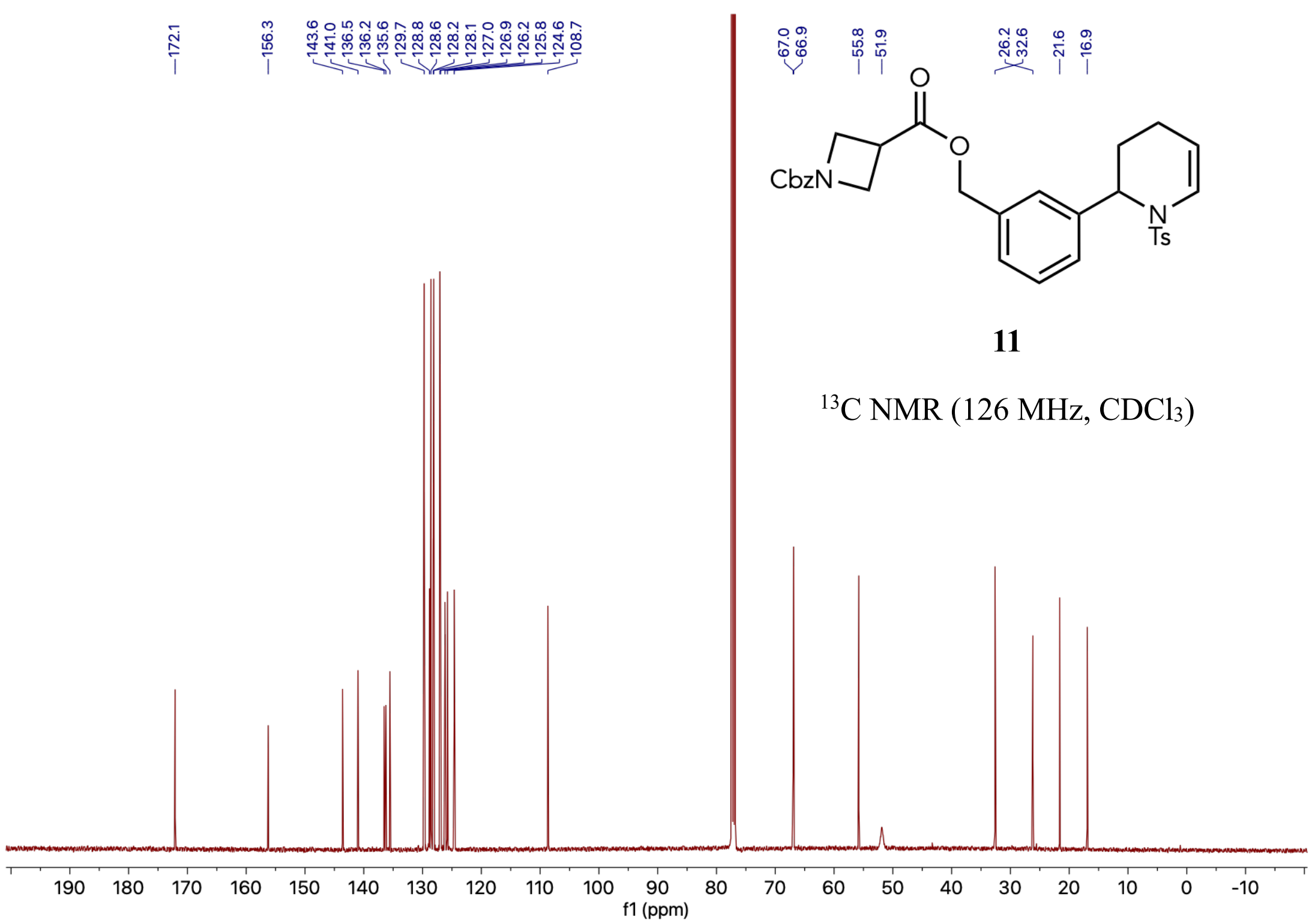
172.1 156.3 143.6 141.0 136.5 136.2 135.6 129.7 128.8 128.6 128.2 128.1 127.0 126.9 126.2 125.8 124.6 108.7 67.0 66.9 55.8 51.9 26.2 32.6 21.6 16.9
CbzN
O
O
N
Ts
11
13C NMR (126 MHz, CDCl3)
190 180 170 160 150 140 130 120 110 100 90 80 70 60 50 40 30 20 10 0 -10
f1 (ppm)

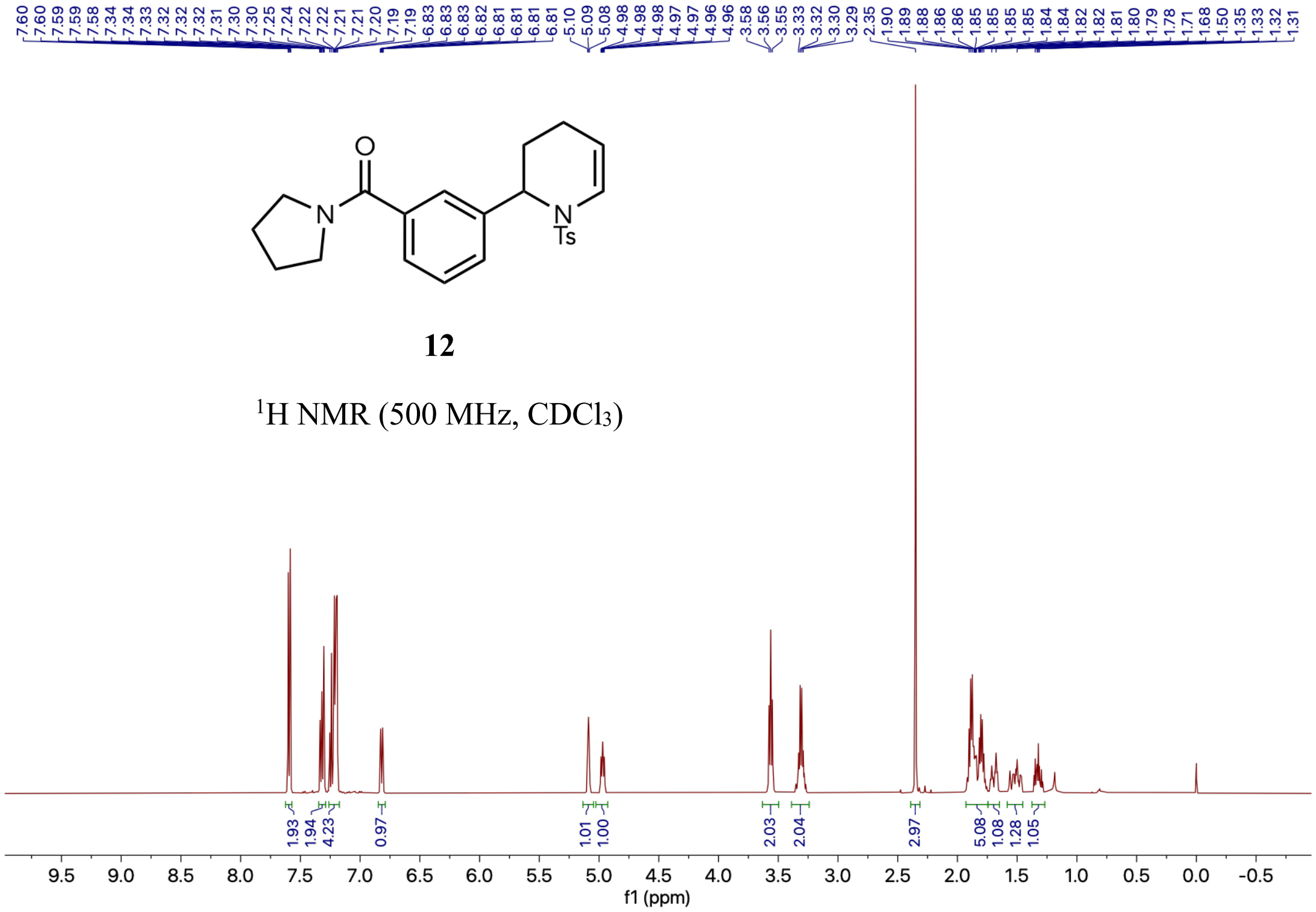

12

^{1}H NMR (500 MHz, $CDCl_3$)

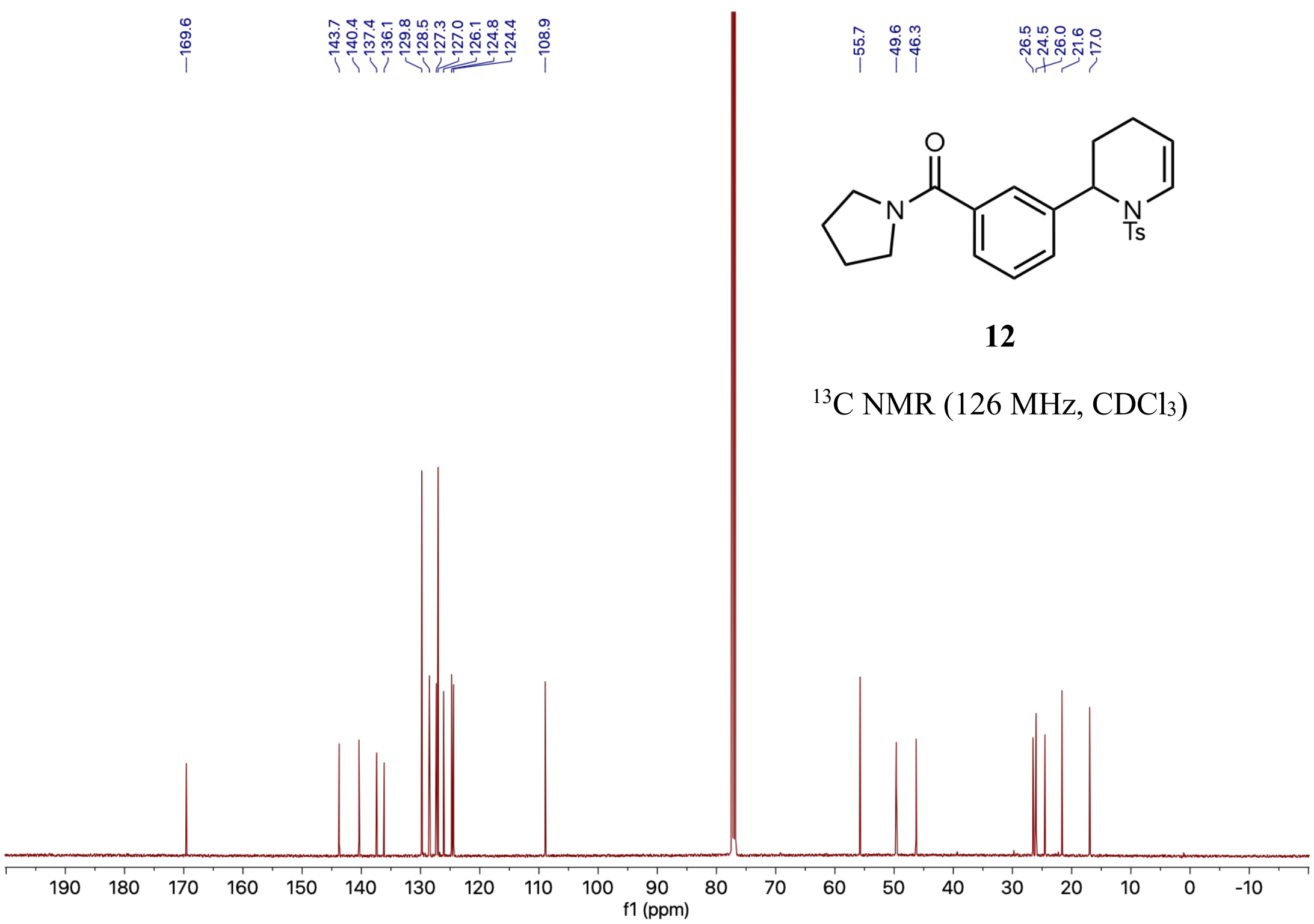

12

^{13}C NMR (126 MHz, $CDCl_3$)

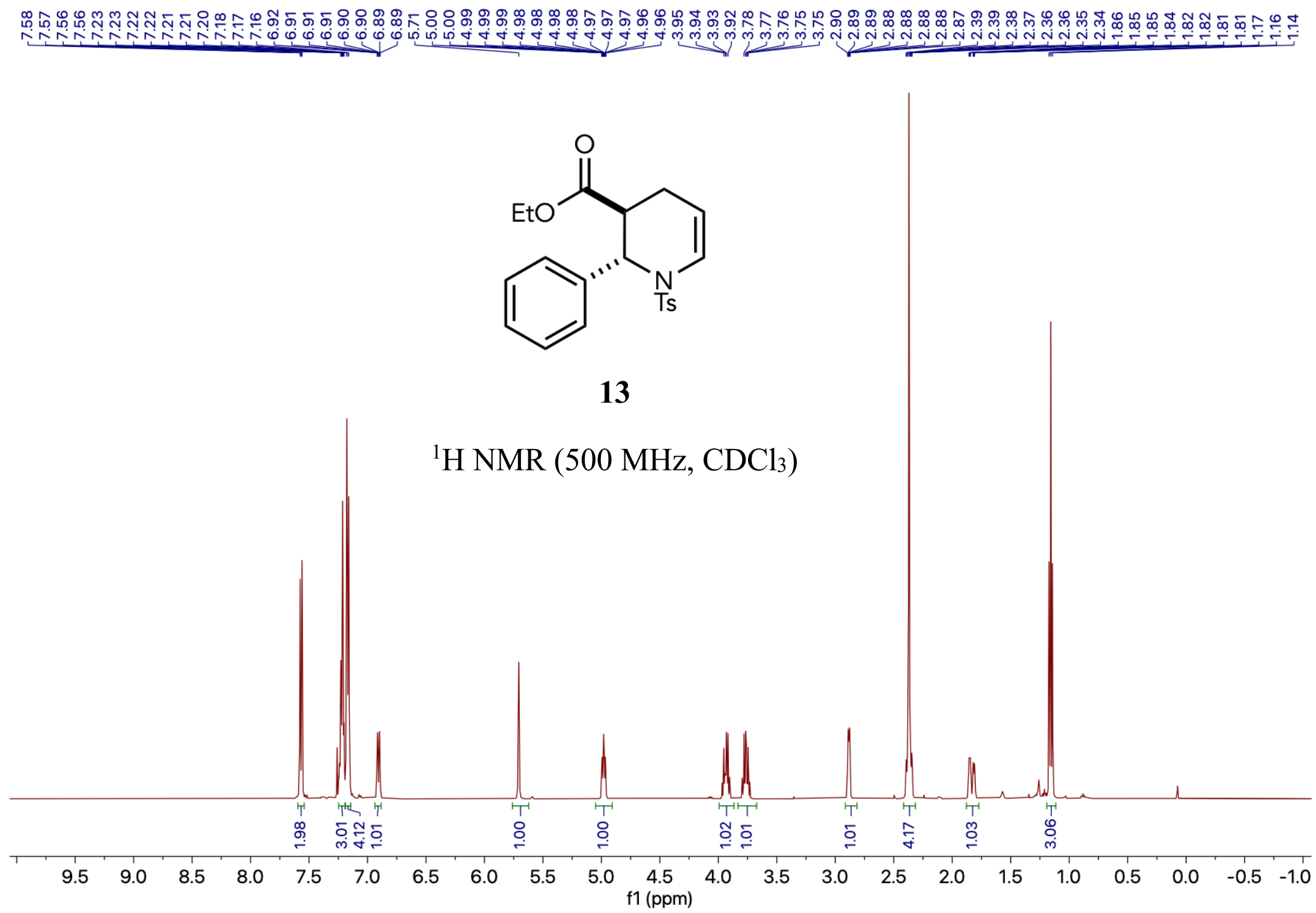

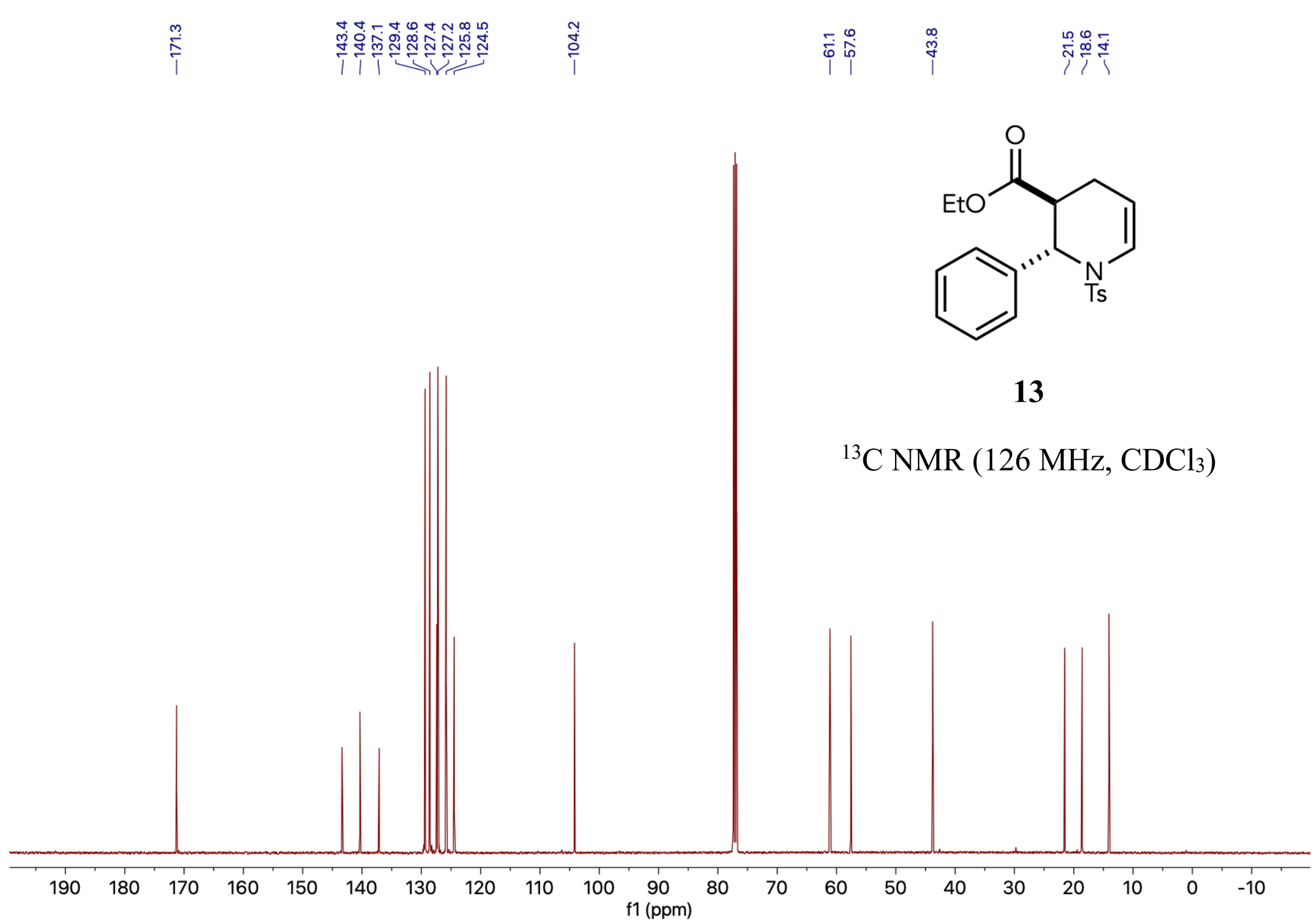

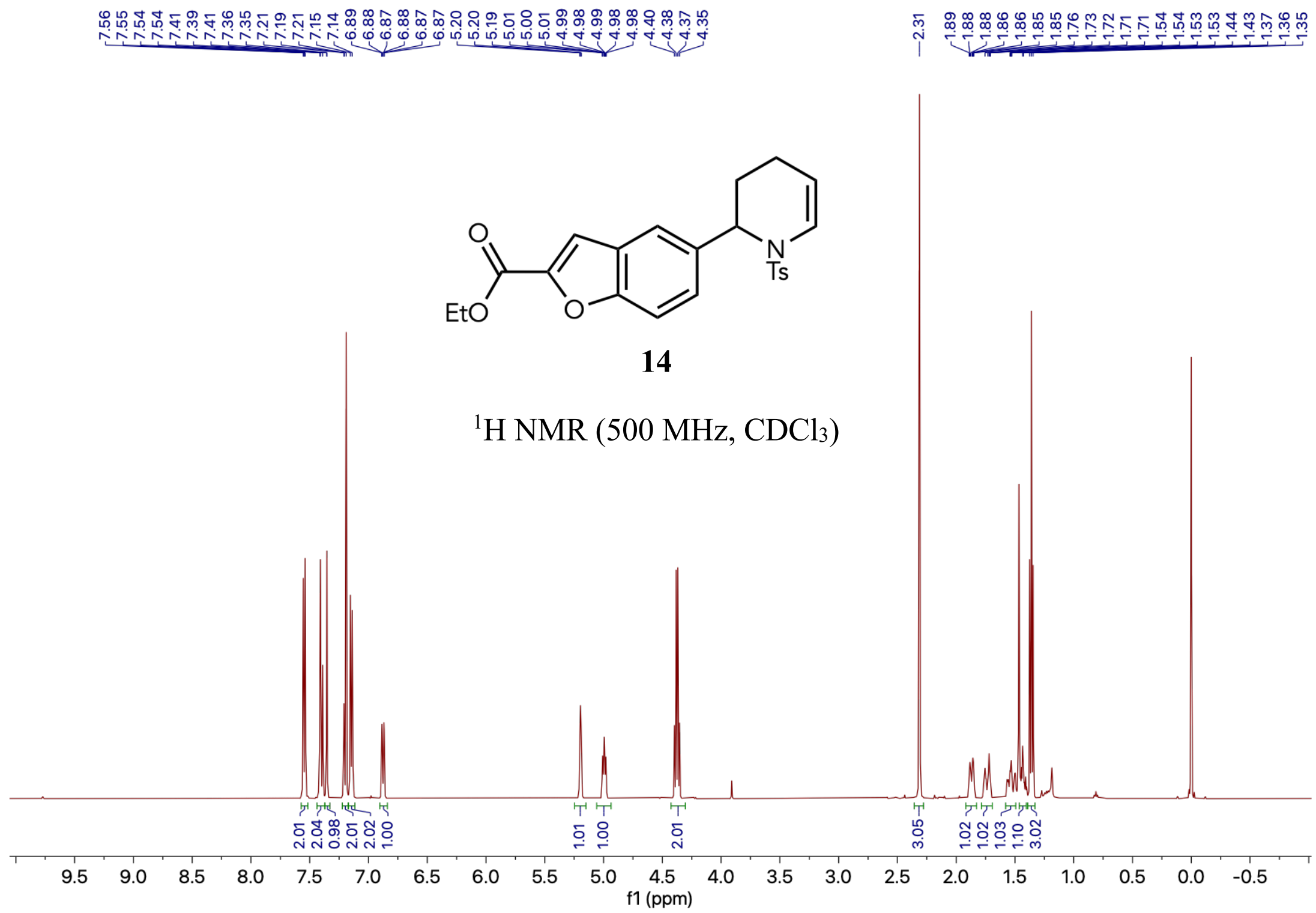

14

^{1}H NMR (500 MHz, $CDCl_3$)

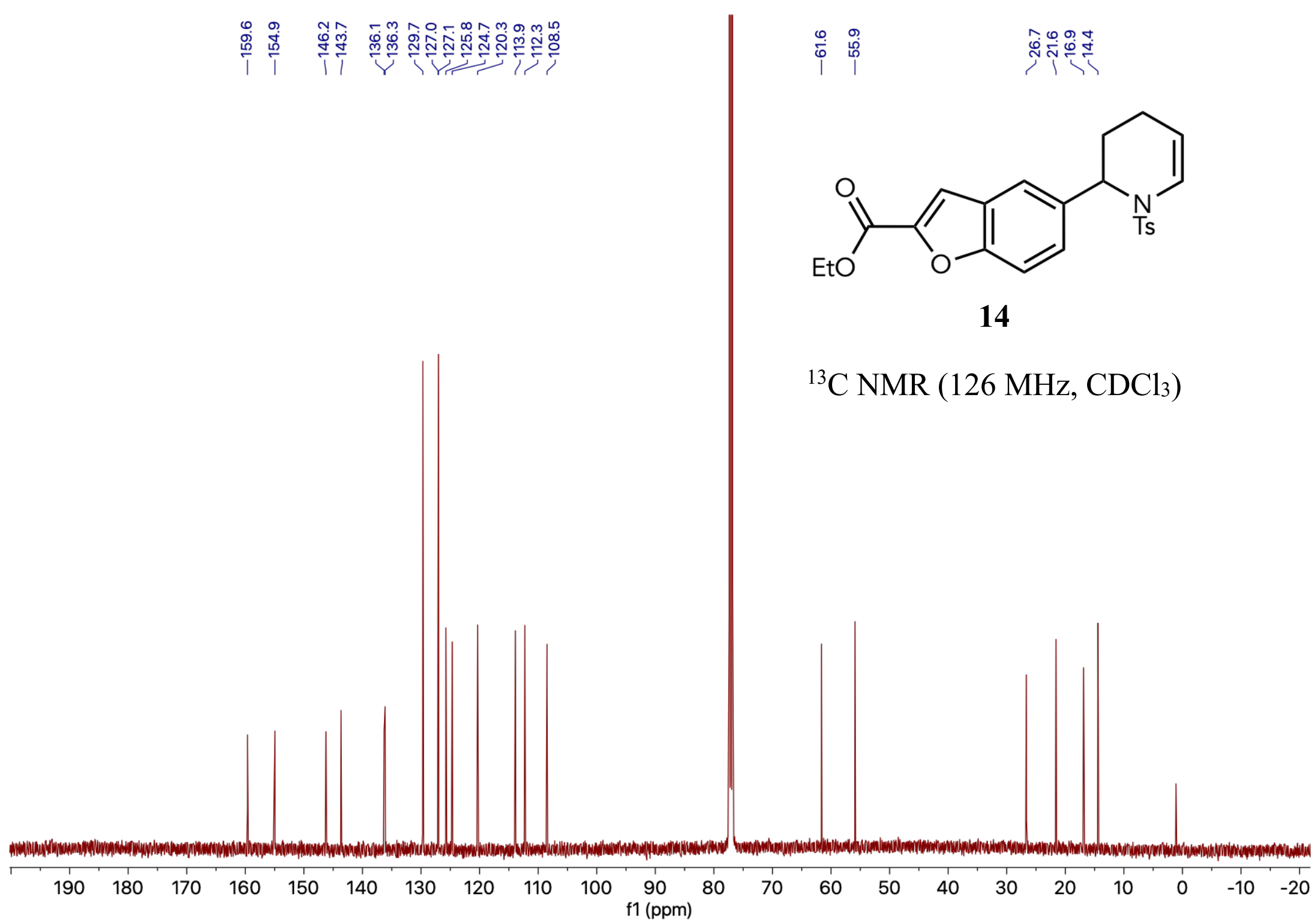

14

^{13}C NMR (126 MHz, $CDCl_3$)

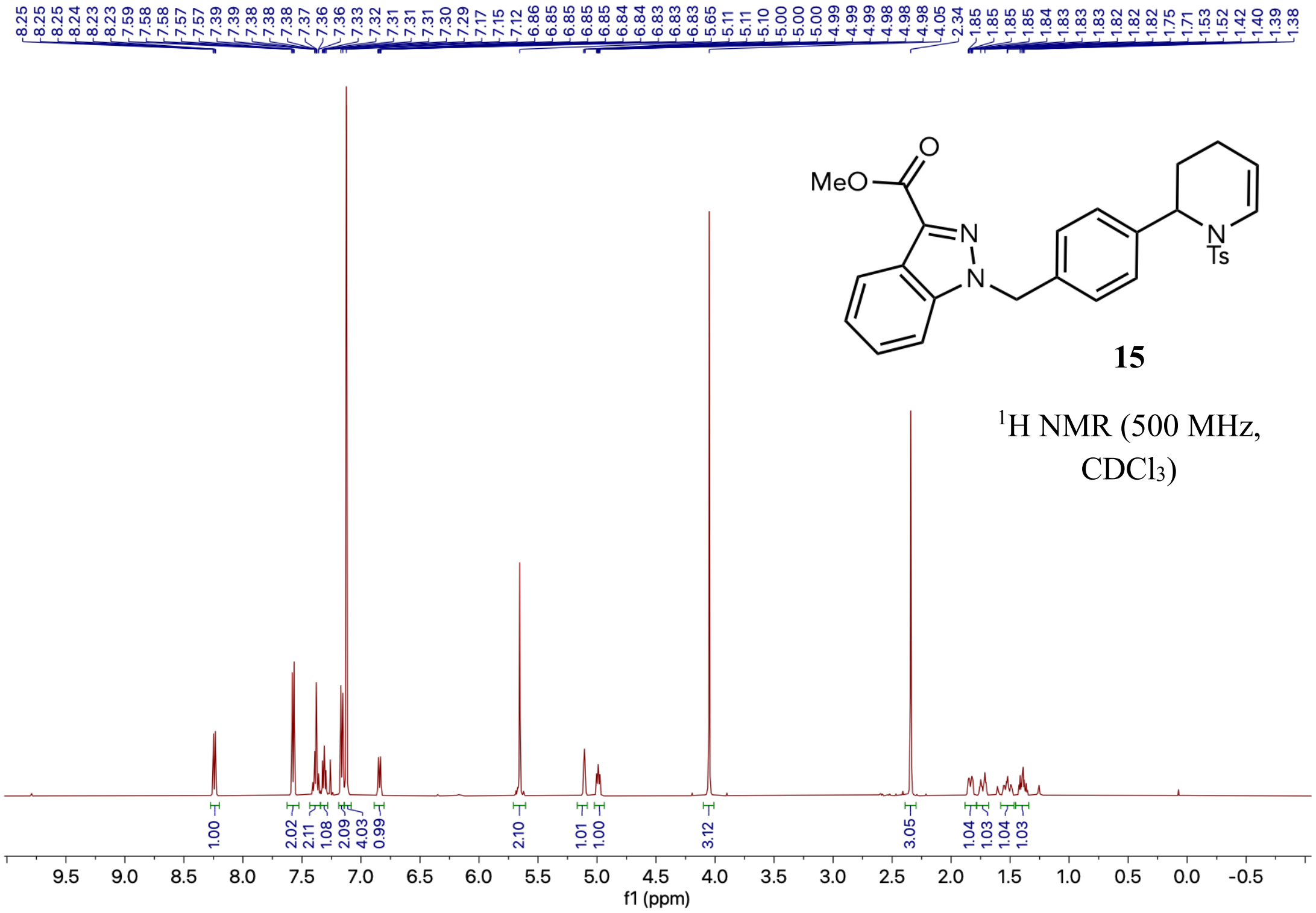

15

^{1}H NMR (500 MHz, $CDCl_3$)

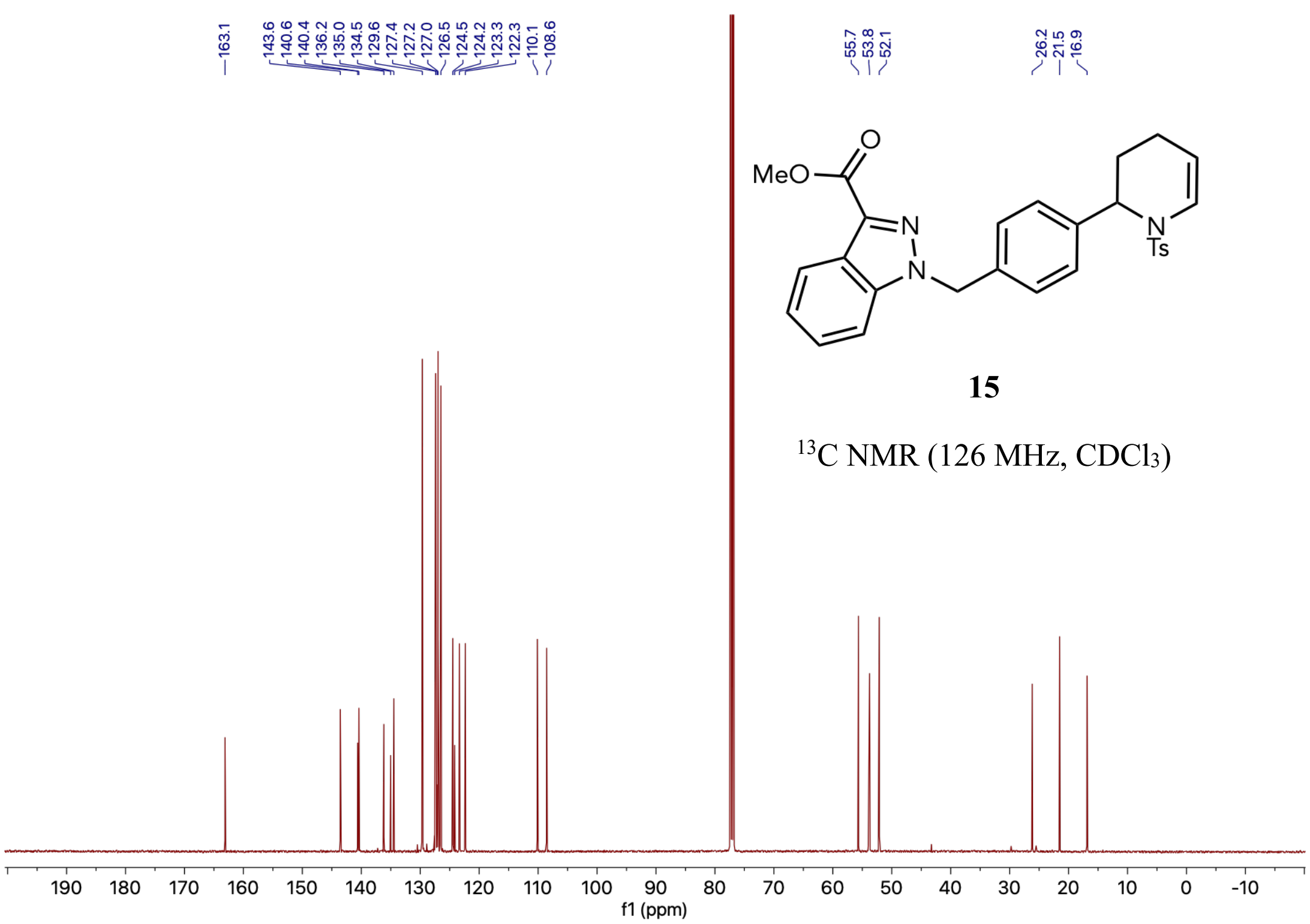

15

^{13}C NMR (126 MHz, $CDCl_3$)

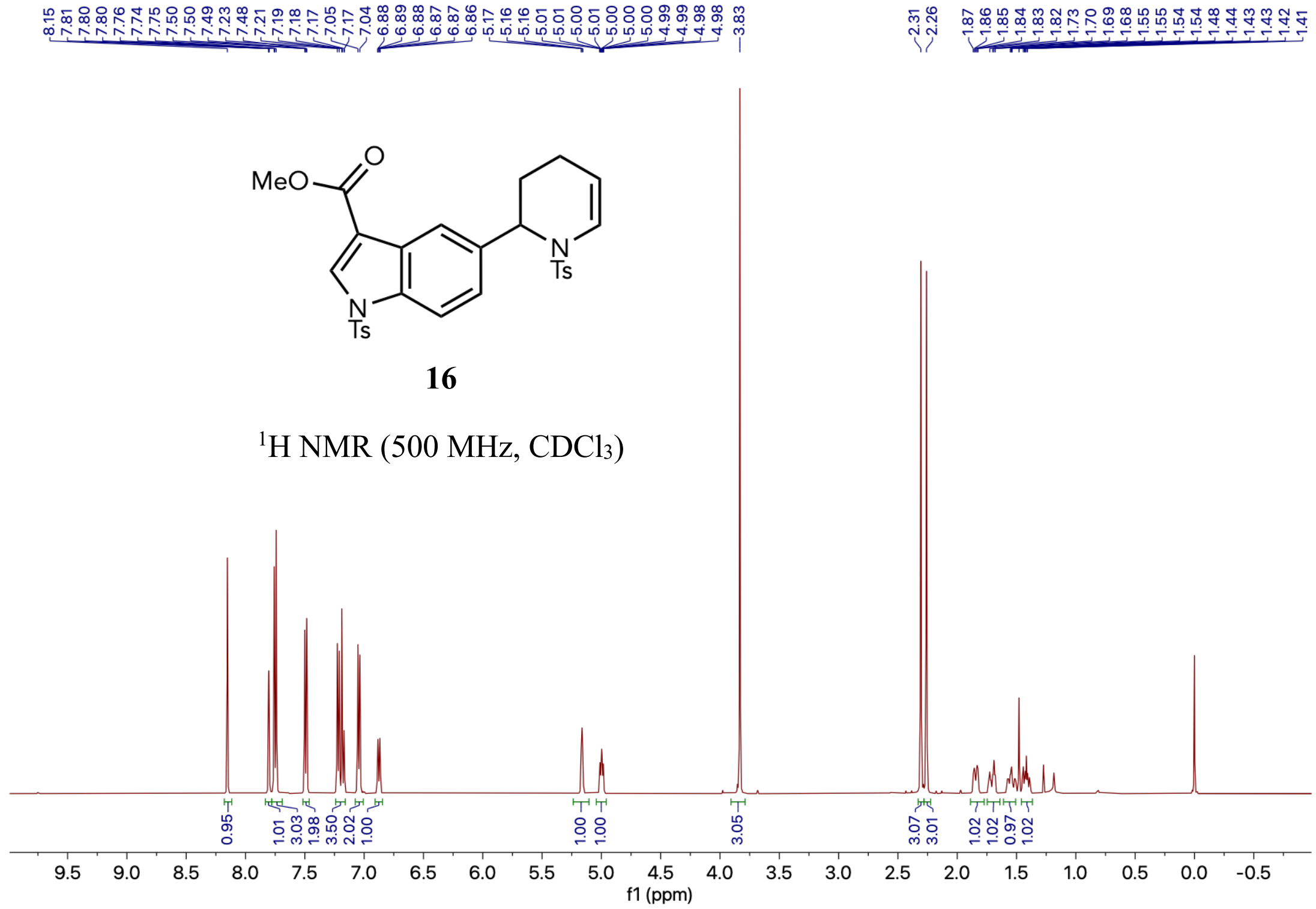

16

^{1}H NMR (500 MHz, $CDCl_3$)

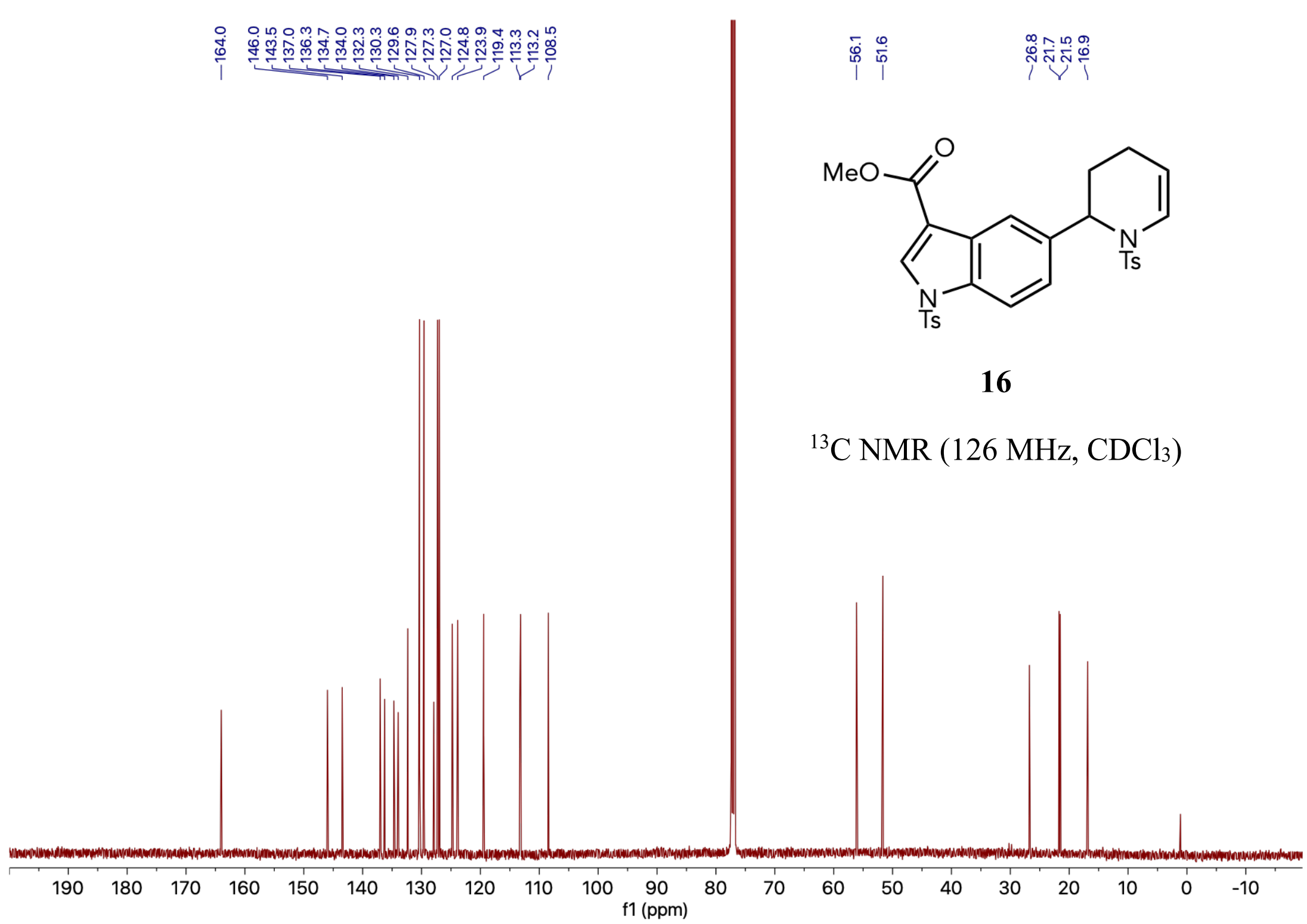

16

^{13}C NMR (126 MHz, $CDCl_3$)

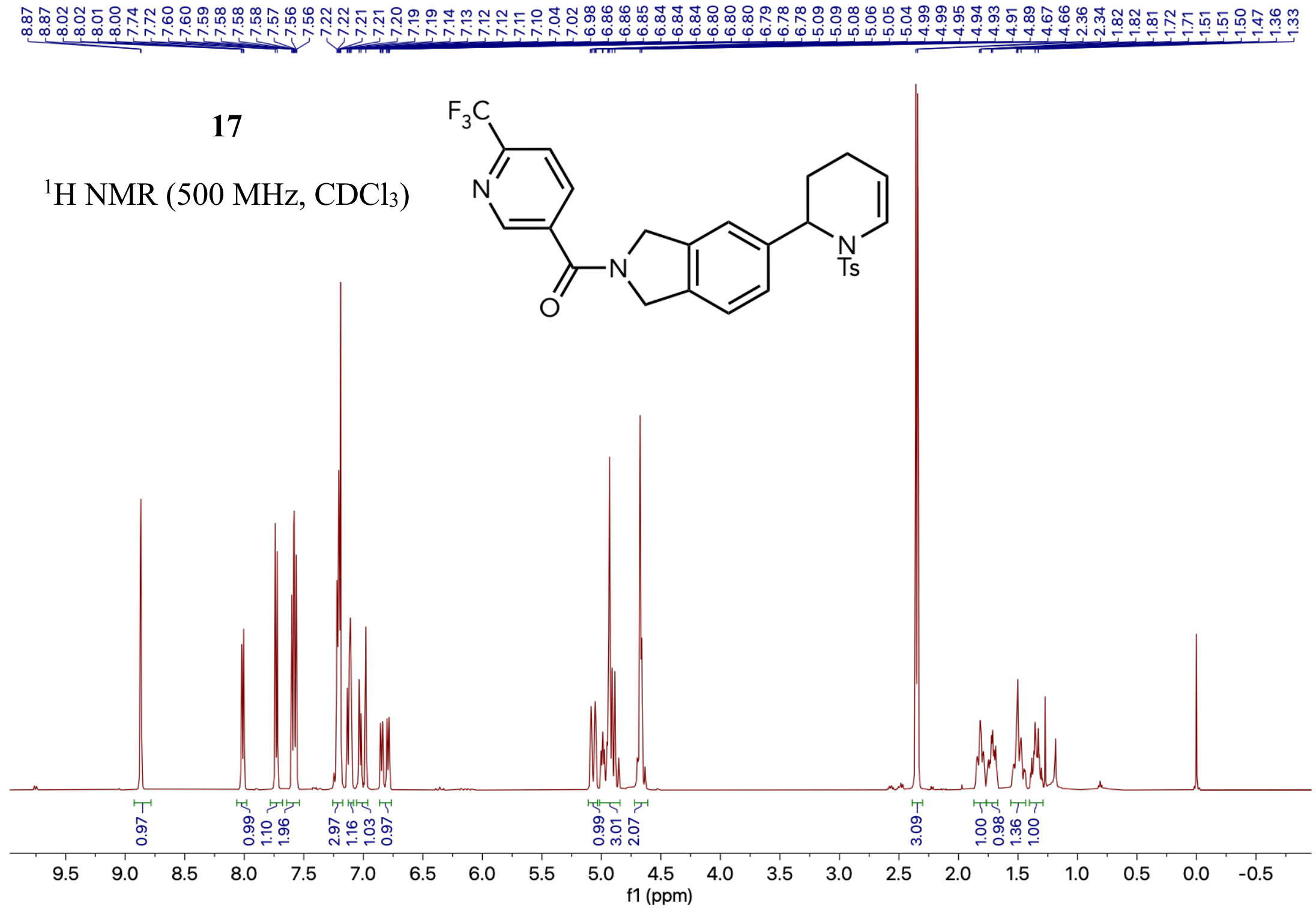
8.87 8.87 8.02 8.01 8.00 7.74 7.72 7.60 7.60 7.59 7.58 7.58 7.58 7.57 7.56 7.56 7.22 7.22 7.21 7.21 7.20 7.19 7.19 7.14 7.13 7.12 7.12 7.11 7.10 7.04 7.02 6.98 6.86 6.86 6.85 6.84 6.84 6.84 6.80 6.80 6.80 6.79 6.78 6.78 5.09 5.09 5.08 5.06 5.05 5.04 4.99 4.99 4.95 4.94 4.93 4.91 4.89 4.67 4.66 2.36 2.34 1.82 1.82 1.81 1.72 1.71 1.51 1.51 1.50 1.47 1.36 1.33
17
¹H NMR (500 MHz, CDCl₃)
F3C
N
O
N
N
Ts
0.97 0.99 1.10 1.96 2.97 1.16 1.03 0.97 0.99 3.01 2.07 3.09 1.00 0.98 1.36 1.00
9.5 9.0 8.5 8.0 7.5 7.0 6.5 6.0 5.5 5.0 4.5 4.0 3.5 3.0 2.5 2.0 1.5 1.0 0.5 0.0 -0.5
f1 (ppm)

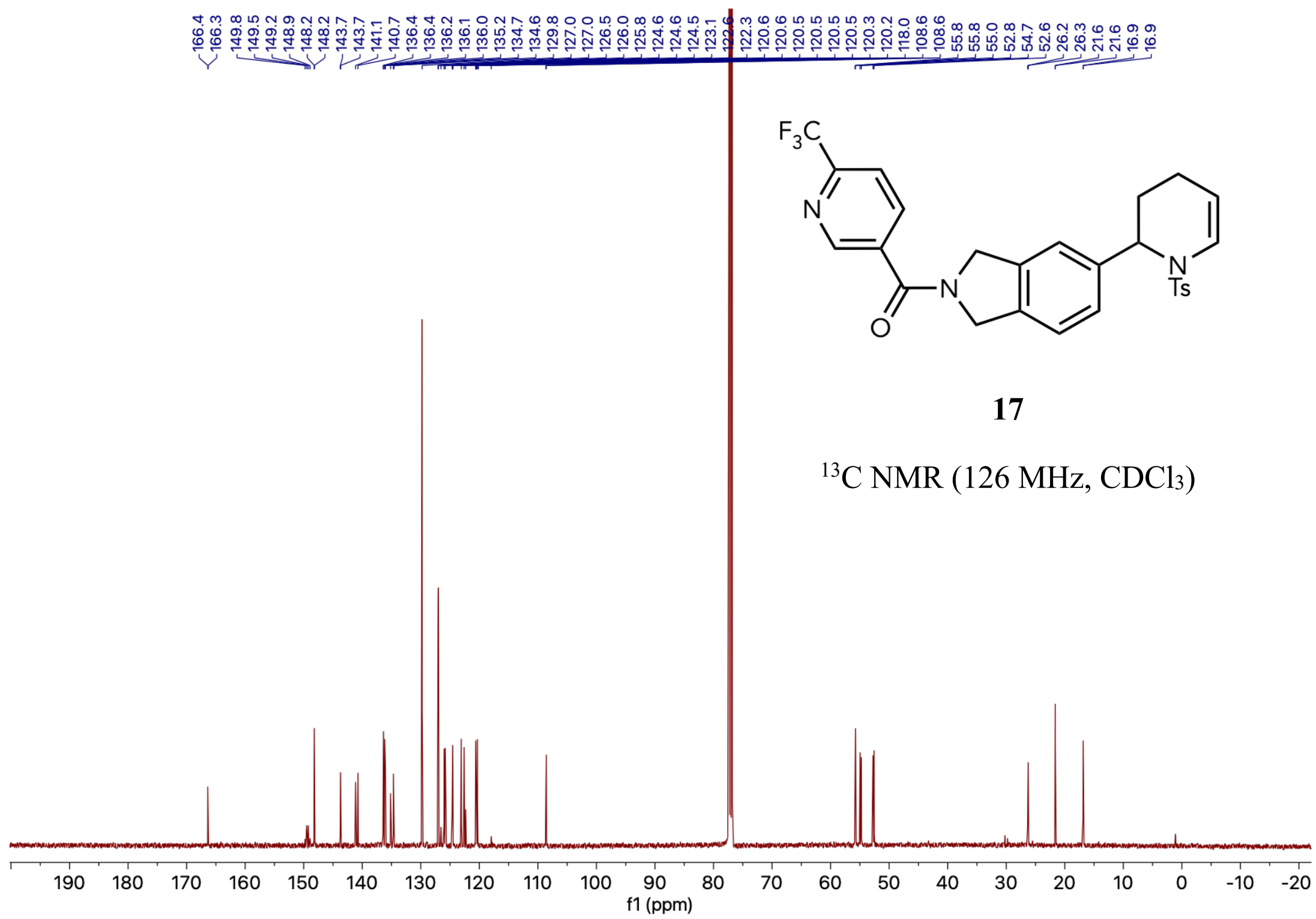
166.4 166.3 149.8 149.5 149.2 148.9 148.2 148.2 143.7 143.7 141.1 140.7 136.4 136.4 136.2 136.1 136.0 135.2 134.7 134.6 129.8 127.0 126.5 126.0 125.8 124.6 124.6 124.5 123.1 122.6 122.3 120.6 120.6 120.5 120.5 120.5 120.5 120.3 120.2 118.0 108.6 108.6 55.8 55.8 55.0 52.8 54.7 52.6 26.2 26.3 21.6 21.6 16.9 16.9
F3C
N
O
N
N
Ts
17
¹³C NMR (126 MHz, CDCl₃)
190 180 170 160 150 140 130 120 110 100 90 80 70 60 50 40 30 20 10 0 -10 -20
f1 (ppm)

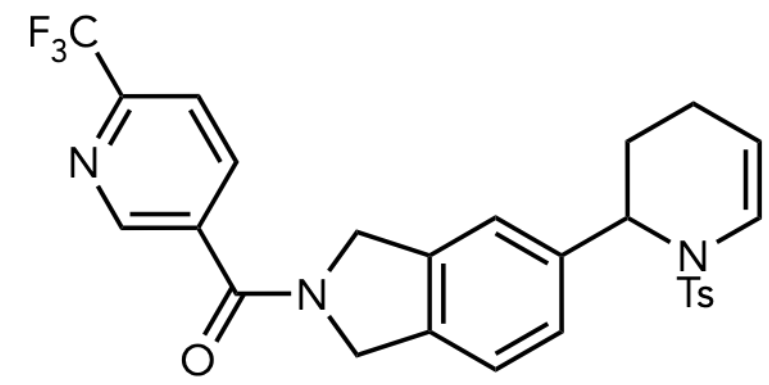

17

^{19}F NMR (377 MHz, $CDCl_3$)

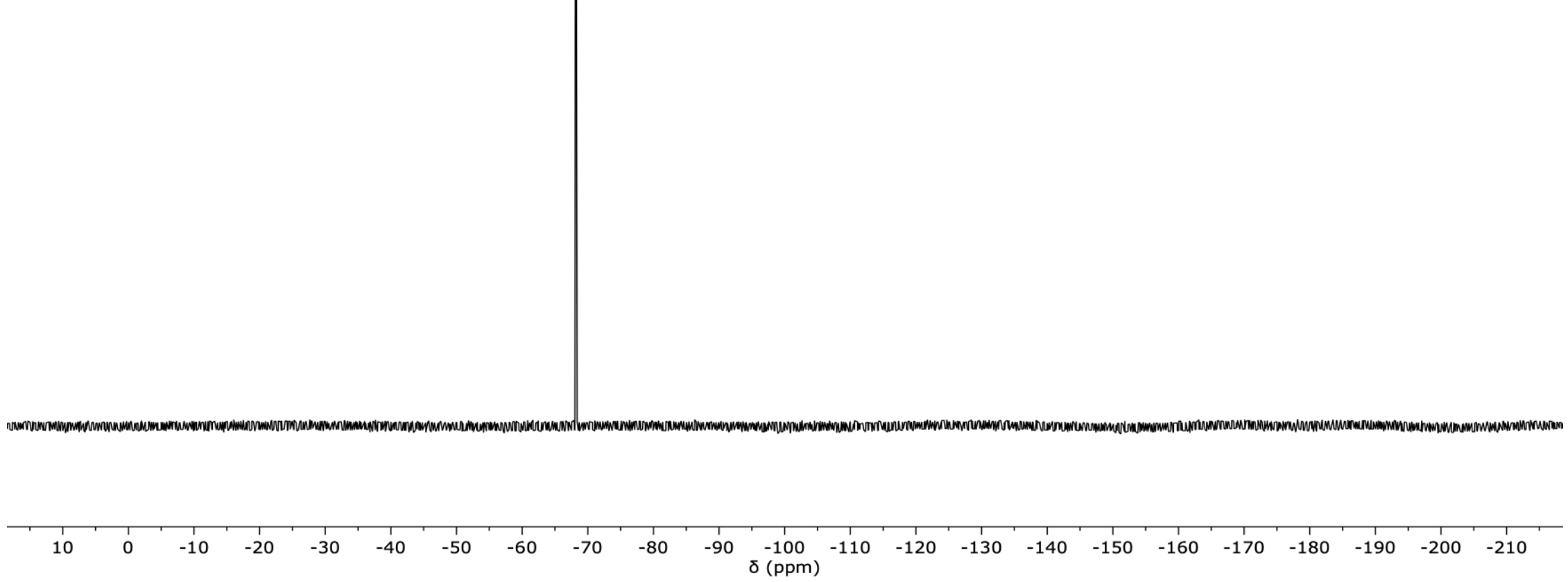

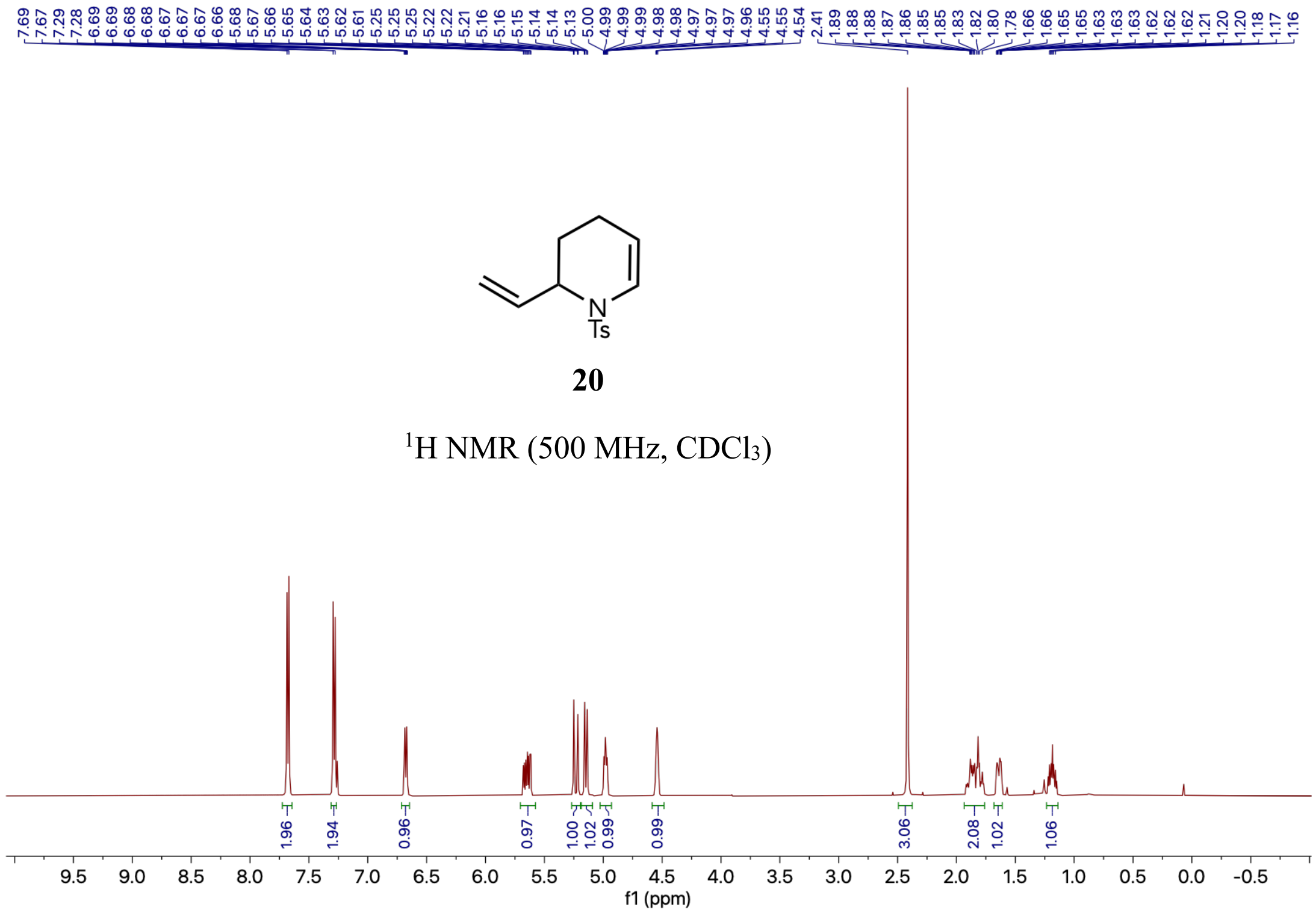
7.69 7.67 7.29 7.28 6.69 6.69 6.68 6.68 6.67 6.67 6.66 5.68 5.67 5.66 5.65 5.64 5.63 5.62 5.61 5.25 5.25 5.25 5.22 5.22 5.21 5.16 5.16 5.15 5.14 5.14 5.13 5.00 4.99 4.99 4.99 4.98 4.98 4.97 4.97 4.96 4.55 4.55 4.54 2.41 1.89 1.88 1.88 1.87 1.86 1.85 1.85 1.83 1.82 1.80 1.78 1.66 1.66 1.65 1.65 1.63 1.63 1.63 1.62 1.62 1.62 1.21 1.20 1.20 1.18 1.17 1.16
N
Ts
20
1H NMR (500 MHz, CDCl3)
1.96 1.94 0.96 0.97 1.00 1.02 0.99 0.99 3.06 2.08 1.02 1.06
9.5 9.0 8.5 8.0 7.5 7.0 6.5 6.0 5.5 5.0 4.5 4.0 3.5 3.0 2.5 2.0 1.5 1.0 0.5 0.0 -0.5
f1 (ppm)

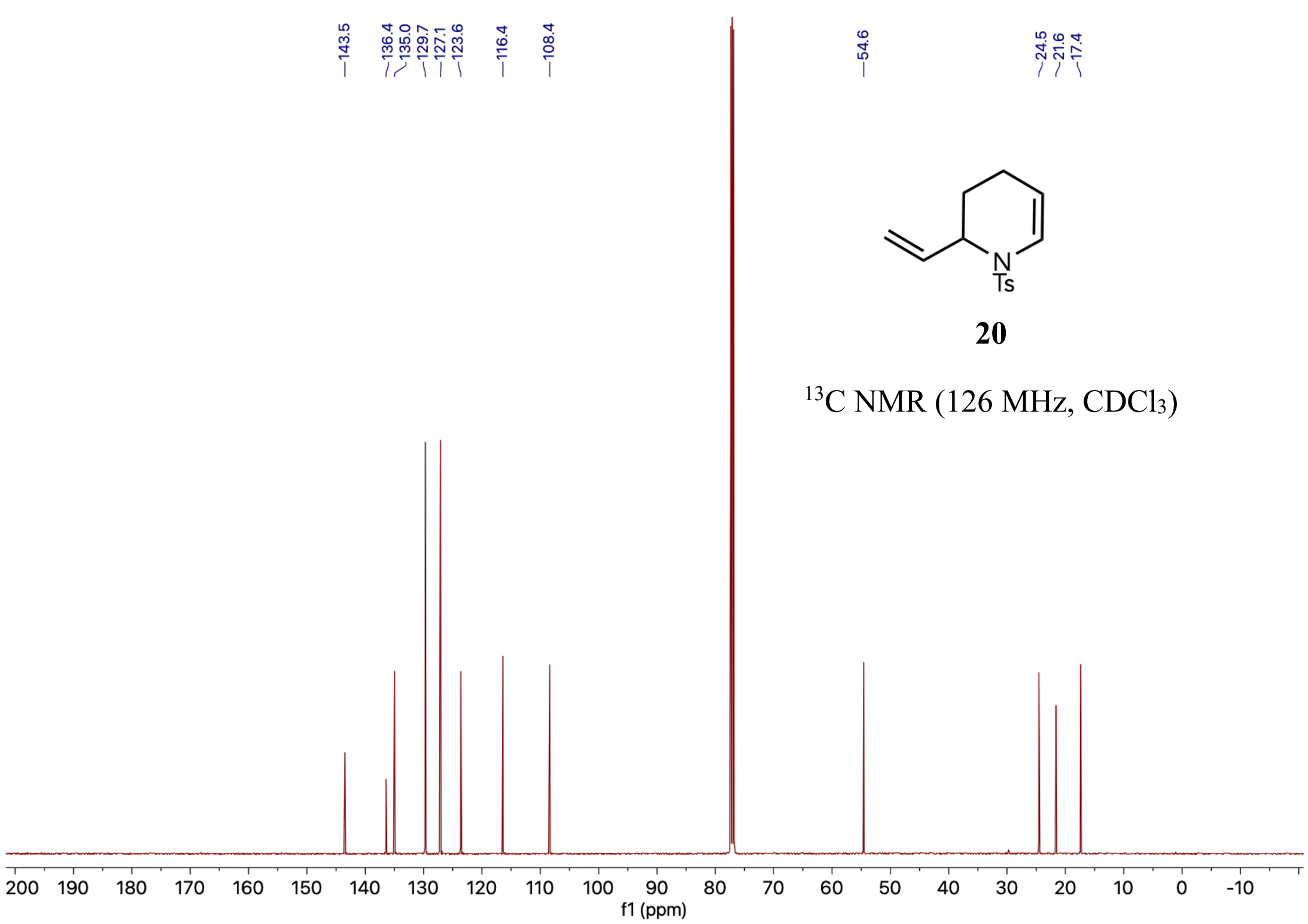
143.5 136.4 135.0 129.7 127.1 123.6 116.4 108.4 54.6 24.5 21.6 17.4
N
Ts
20
13C NMR (126 MHz, CDCl3)
200 190 180 170 160 150 140 130 120 110 100 90 80 70 60 50 40 30 20 10 0 -10
f1 (ppm)

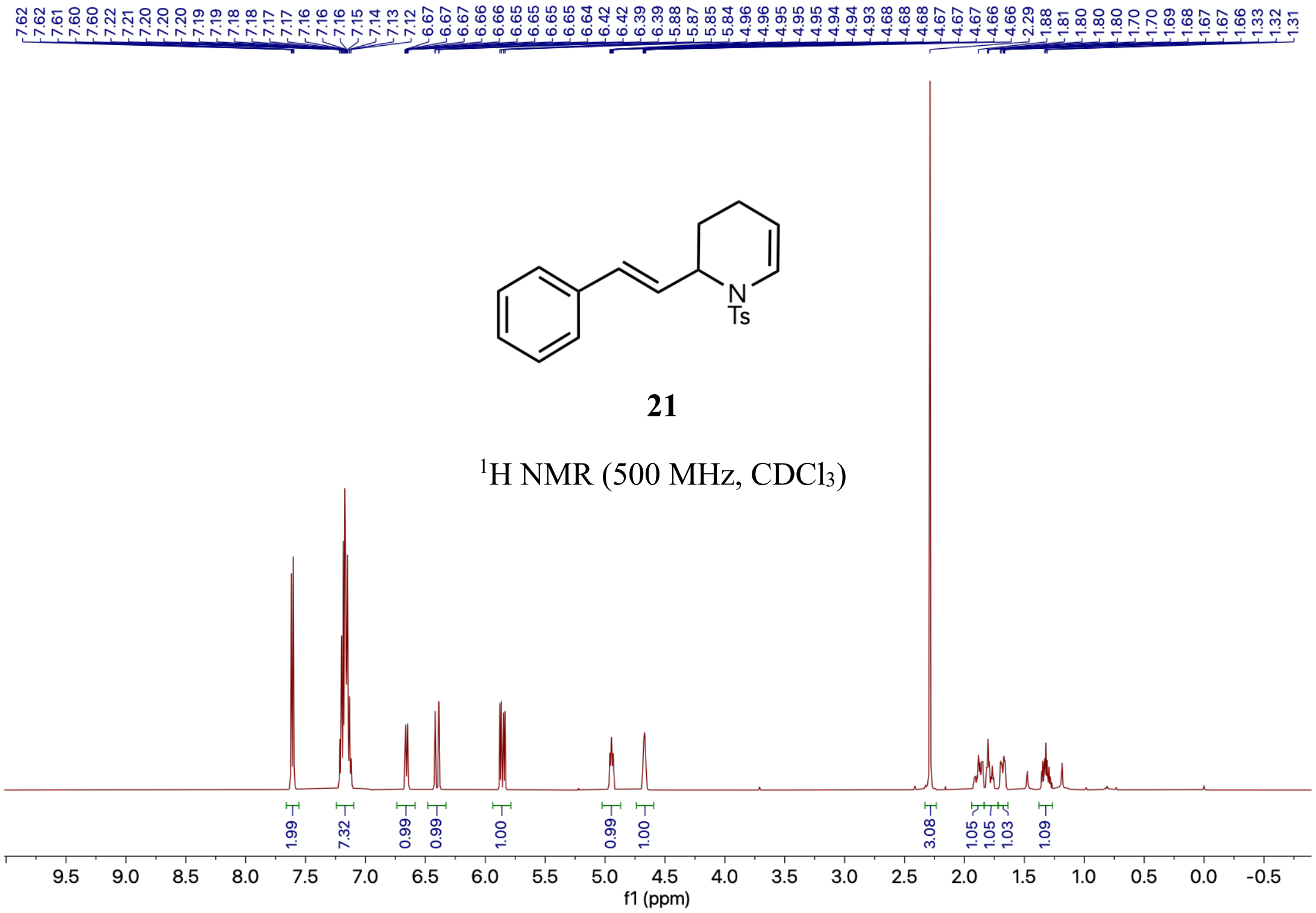
7.62
7.62
7.61
7.60
7.22
7.21
7.20
7.20
7.19
7.19
7.18
7.18
7.17
7.16
7.16
7.16
7.15
7.14
7.13
7.12
6.67
6.67
6.67
6.66
6.66
6.65
6.65
6.65
6.65
6.64
6.42
6.42
6.39
6.39
5.88
5.87
5.85
5.84
4.96
4.96
4.95
4.95
4.95
4.94
4.94
4.93
4.68
4.68
4.68
4.67
4.67
4.67
4.66
4.66
2.29
1.88
1.81
1.80
1.80
1.80
1.70
1.69
1.68
1.67
1.66
1.33
1.32
1.31
N
Ts
21
1H NMR (500 MHz, CDCl3)
1.99
7.32
0.99
0.99
1.00
0.99
1.00
3.08
1.05
1.05
1.03
1.09
9.5 9.0 8.5 8.0 7.5 7.0 6.5 6.0 5.5 5.0 4.5 4.0 3.5 3.0 2.5 2.0 1.5 1.0 0.5 0.0 -0.5
f1 (ppm)

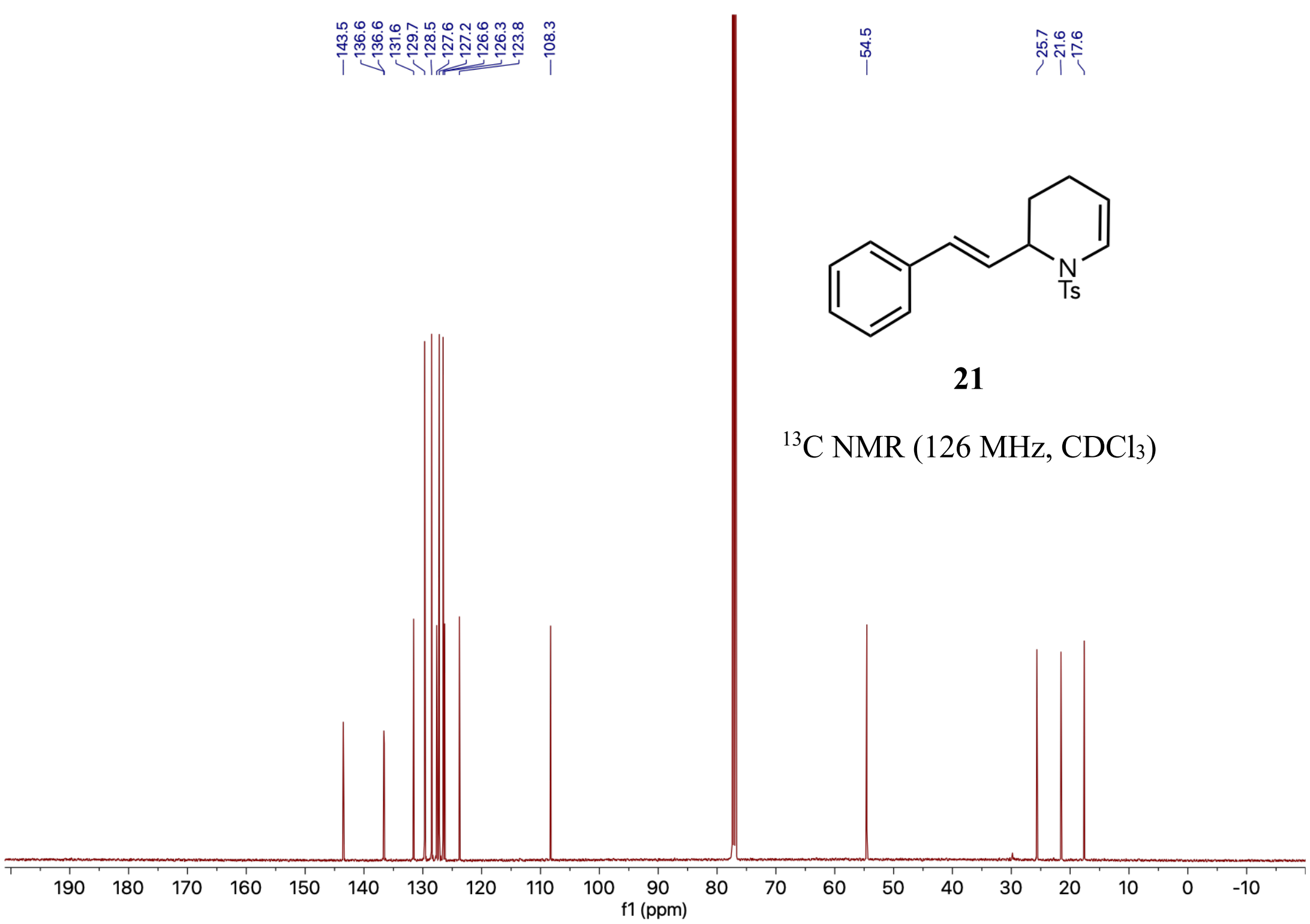
143.5
136.6
136.6
131.6
129.7
128.5
127.6
127.2
126.6
126.3
123.8
108.3
54.5
25.7
21.6
17.6
N
Ts
21
13C NMR (126 MHz, CDCl3)
190 180 170 160 150 140 130 120 110 100 90 80 70 60 50 40 30 20 10 0 -10
f1 (ppm)

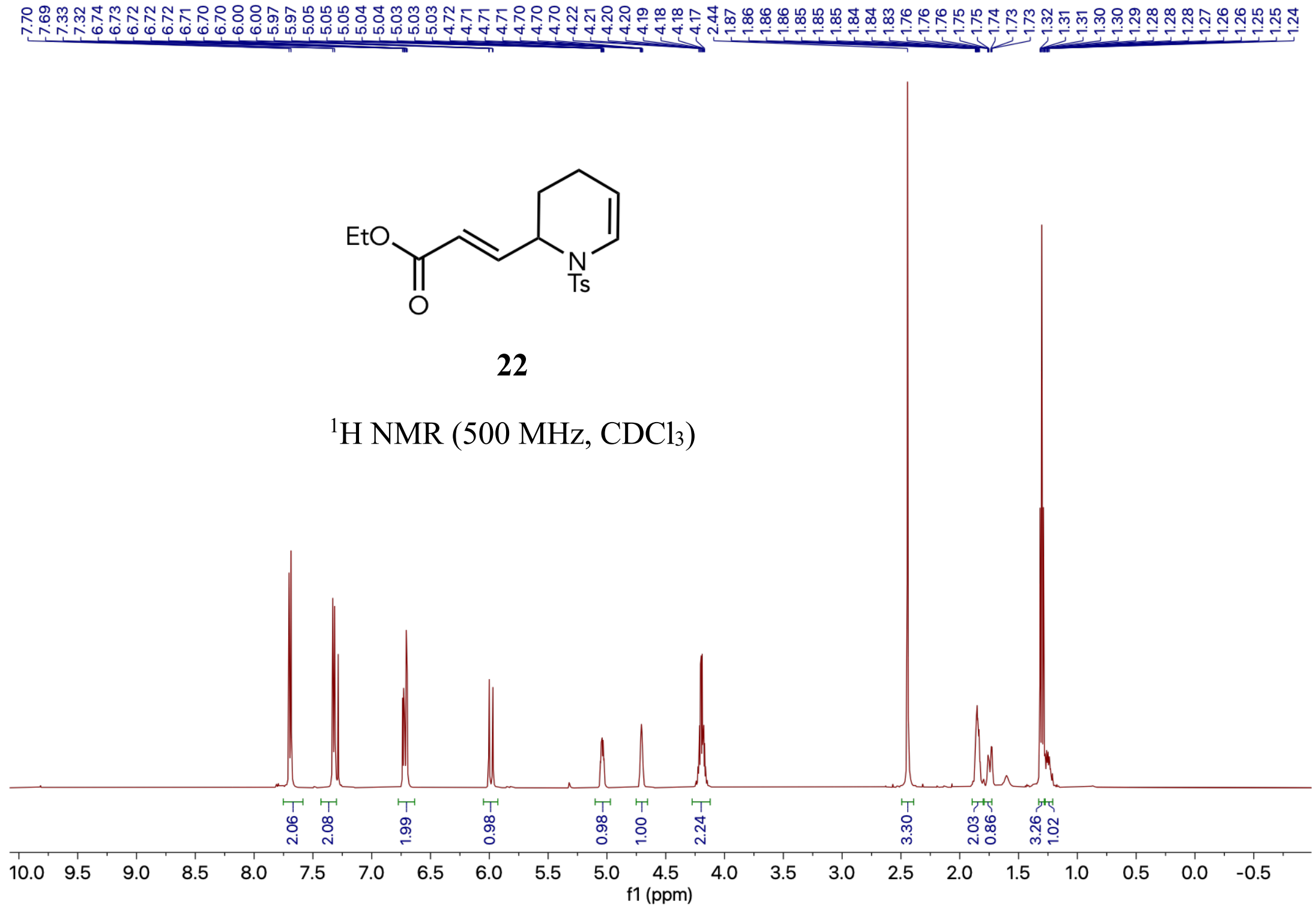

22

^{1}H NMR (500 MHz, $CDCl_3$)

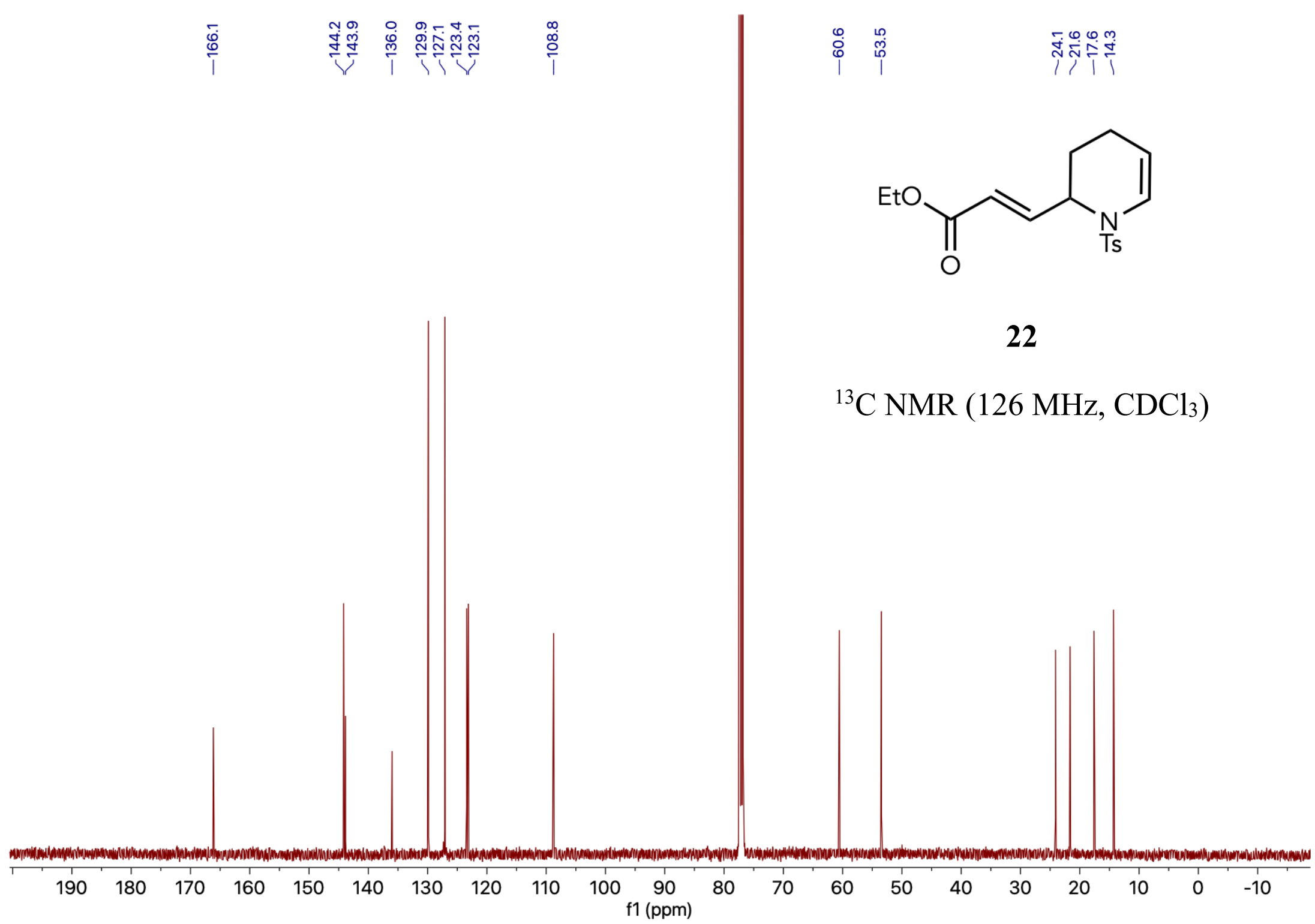

22

^{13}C NMR (126 MHz, $CDCl_3$)

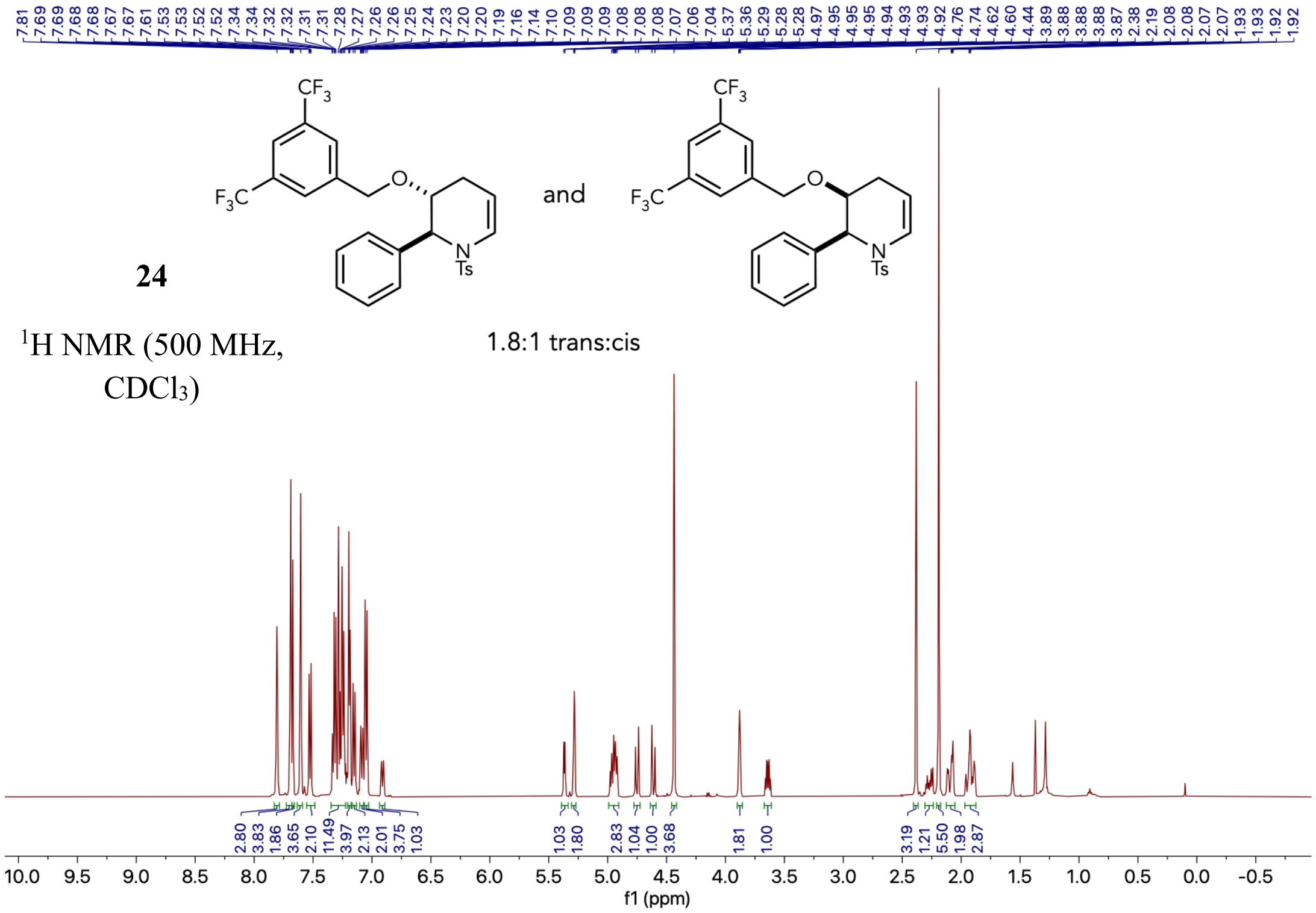
24
^{1}H NMR (500 MHz, CDCl$_3$)
1.8:1 trans:cis
f1 (ppm)

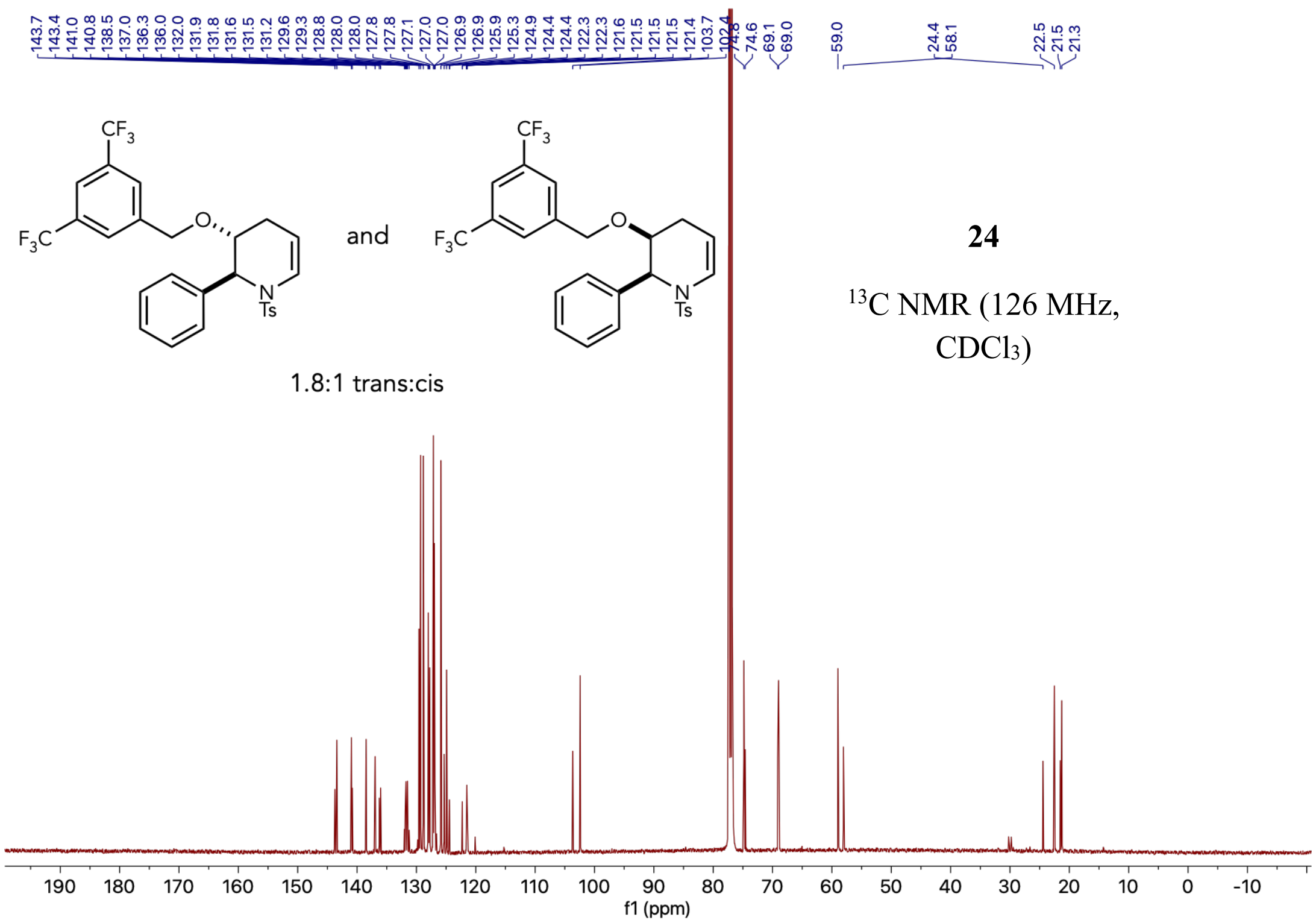
24
^{13}C NMR (126 MHz, CDCl$_3$)
1.8:1 trans:cis
f1 (ppm)

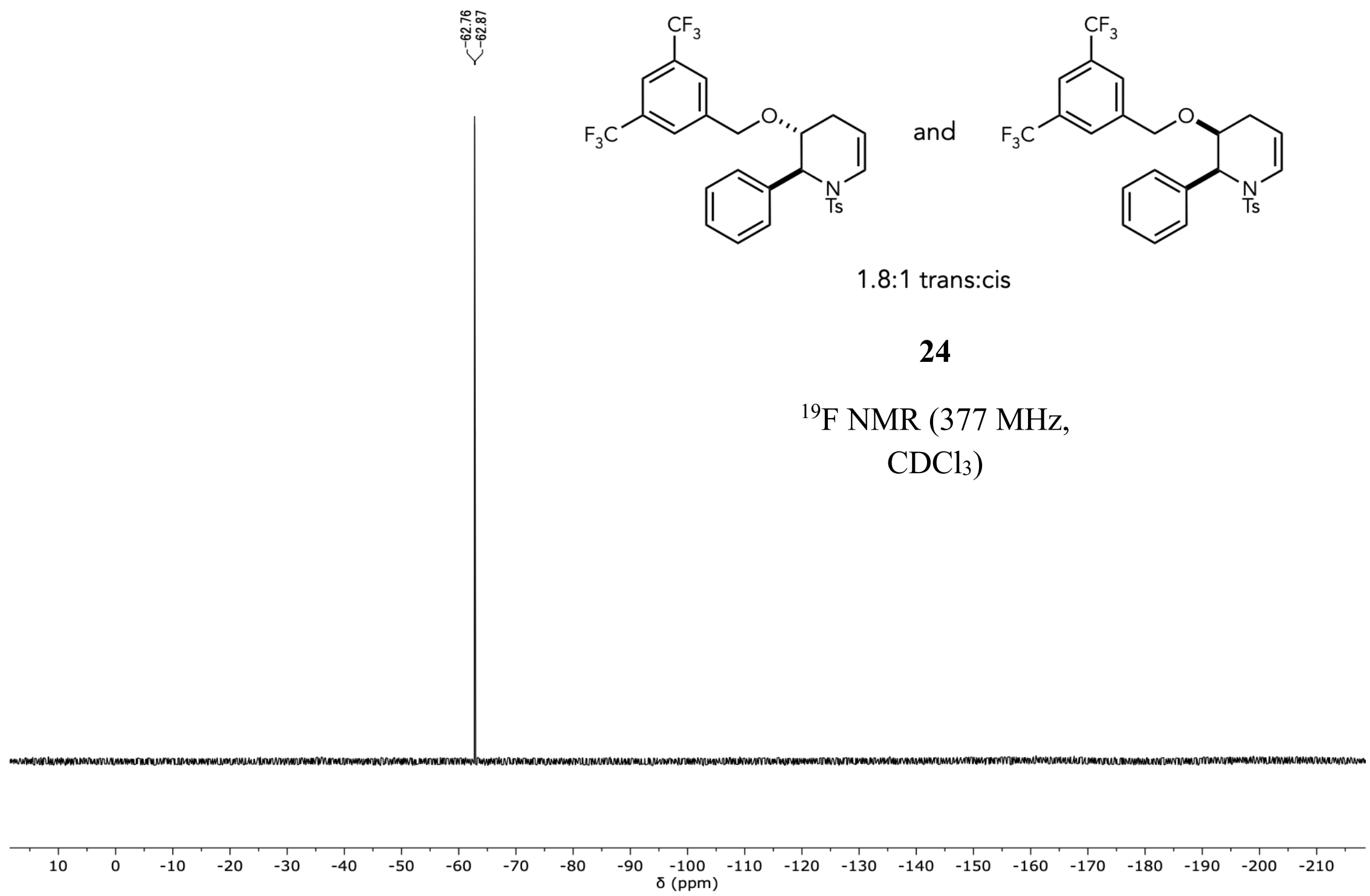

1.8:1 trans:cis

24

^{19}F NMR (377 MHz, $CDCl_3$)

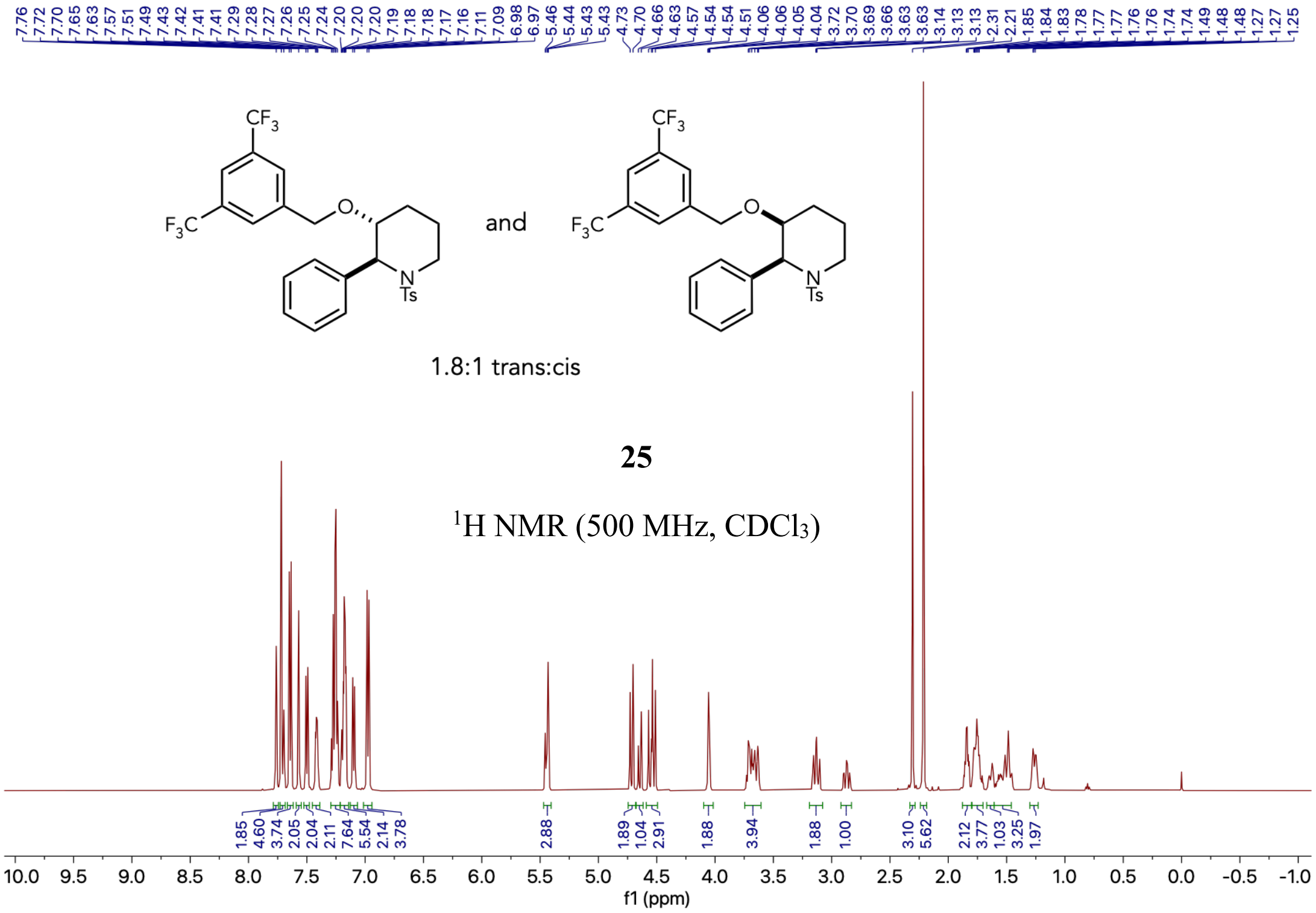
7.76 7.72 7.70 7.65 7.63 7.57 7.51 7.49 7.43 7.42 7.41 7.41 7.29 7.28 7.27 7.26 7.25 7.24 7.20 7.20 7.20 7.19 7.18 7.18 7.17 7.16 7.11 7.09 6.98 6.97 5.46 5.44 5.43 5.43 4.73 4.70 4.66 4.63 4.57 4.54 4.54 4.51 4.06 4.06 4.05 4.04 3.72 3.70 3.69 3.66 3.63 3.63 3.14 3.13 3.13 2.31 2.21 1.85 1.84 1.83 1.78 1.77 1.76 1.74 1.74 1.49 1.48 1.48 1.27 1.27 1.25
CF3
F3C
O
N
Ts
and
CF3
F3C
O
N
Ts
1.8:1 trans:cis
25
1H NMR (500 MHz, CDCl3)
1.85 4.60 3.74 2.05 2.04 2.11 7.64 5.54 2.14 3.78 2.88 1.89 1.04 2.91 1.88 3.94 1.88 1.00 3.10 5.62 2.12 3.77 1.03 3.25 1.97
10.0 9.5 9.0 8.5 8.0 7.5 7.0 6.5 6.0 5.5 5.0 4.5 4.0 3.5 3.0 2.5 2.0 1.5 1.0 0.5 0.0 -0.5 -1.0
f1 (ppm)

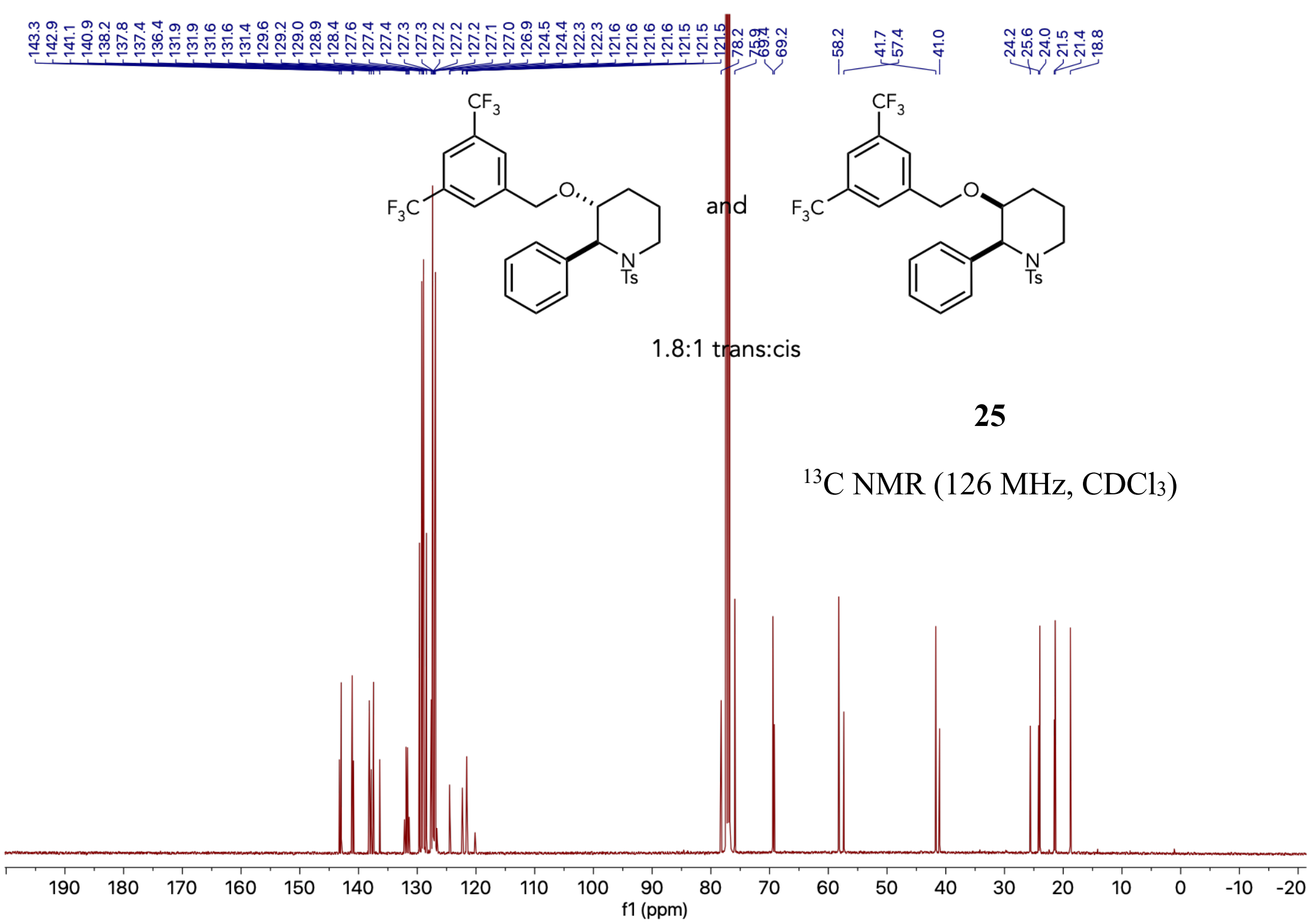
143.3 142.9 141.1 140.9 138.2 137.8 137.4 136.4 131.9 131.6 131.6 131.4 129.6 129.2 129.0 128.9 128.4 127.6 127.4 127.3 127.3 127.2 127.2 127.2 127.1 127.0 126.9 124.5 124.4 122.3 122.3 121.6 121.6 121.6 121.6 121.5 121.5 121.5 78.2 75.9 69.4 69.2 58.2 41.7 57.4 41.0 24.2 25.6 24.0 21.5 21.4 18.8
CF3
F3C
O
N
Ts
and
CF3
F3C
O
N
Ts
1.8:1 trans:cis
25
13C NMR (126 MHz, CDCl3)
190 180 170 160 150 140 130 120 110 100 90 80 70 60 50 40 30 20 10 0 -10 -20
f1 (ppm)

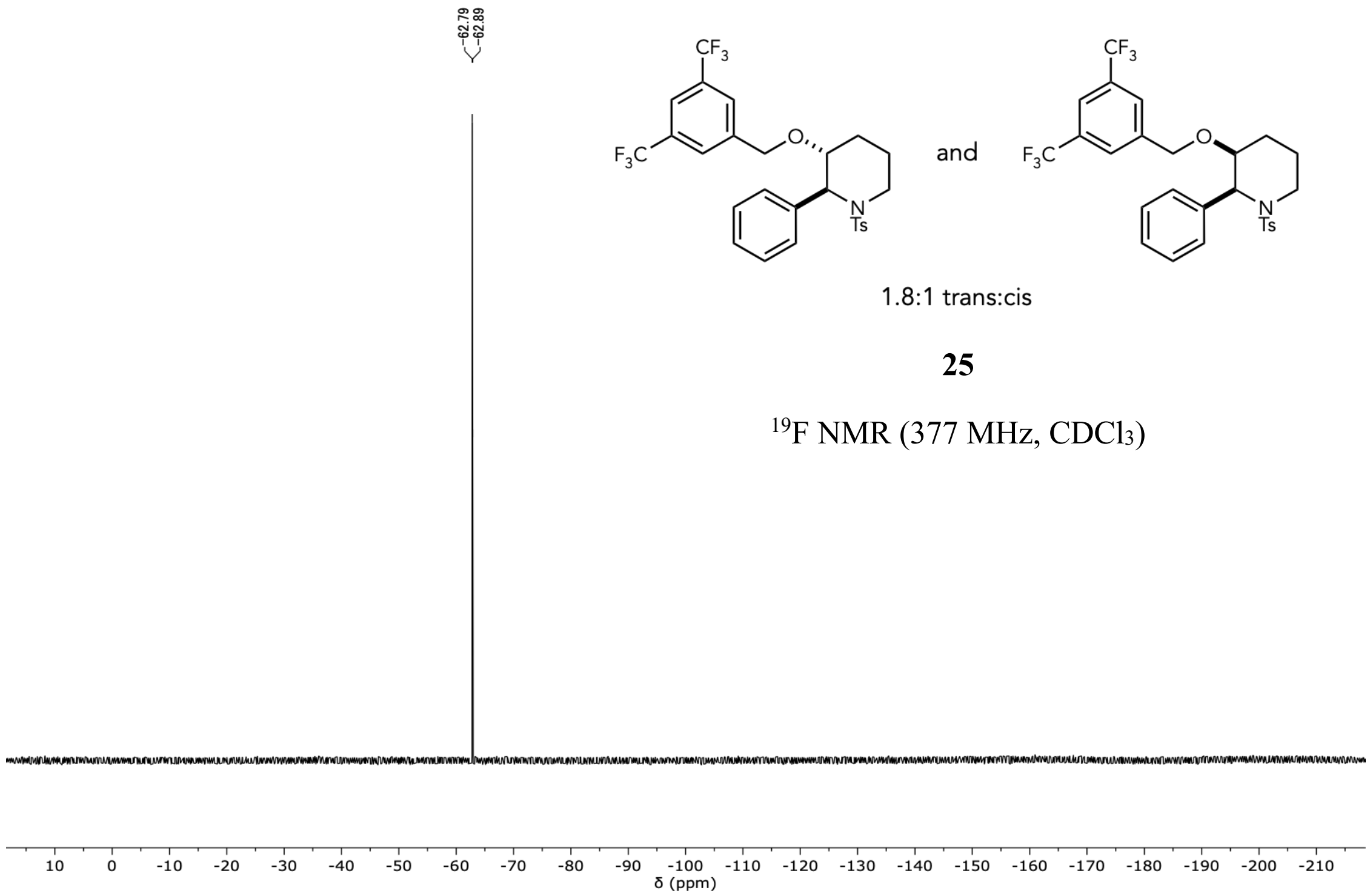

25

^{19}F NMR (377 MHz, $CDCl_3$)

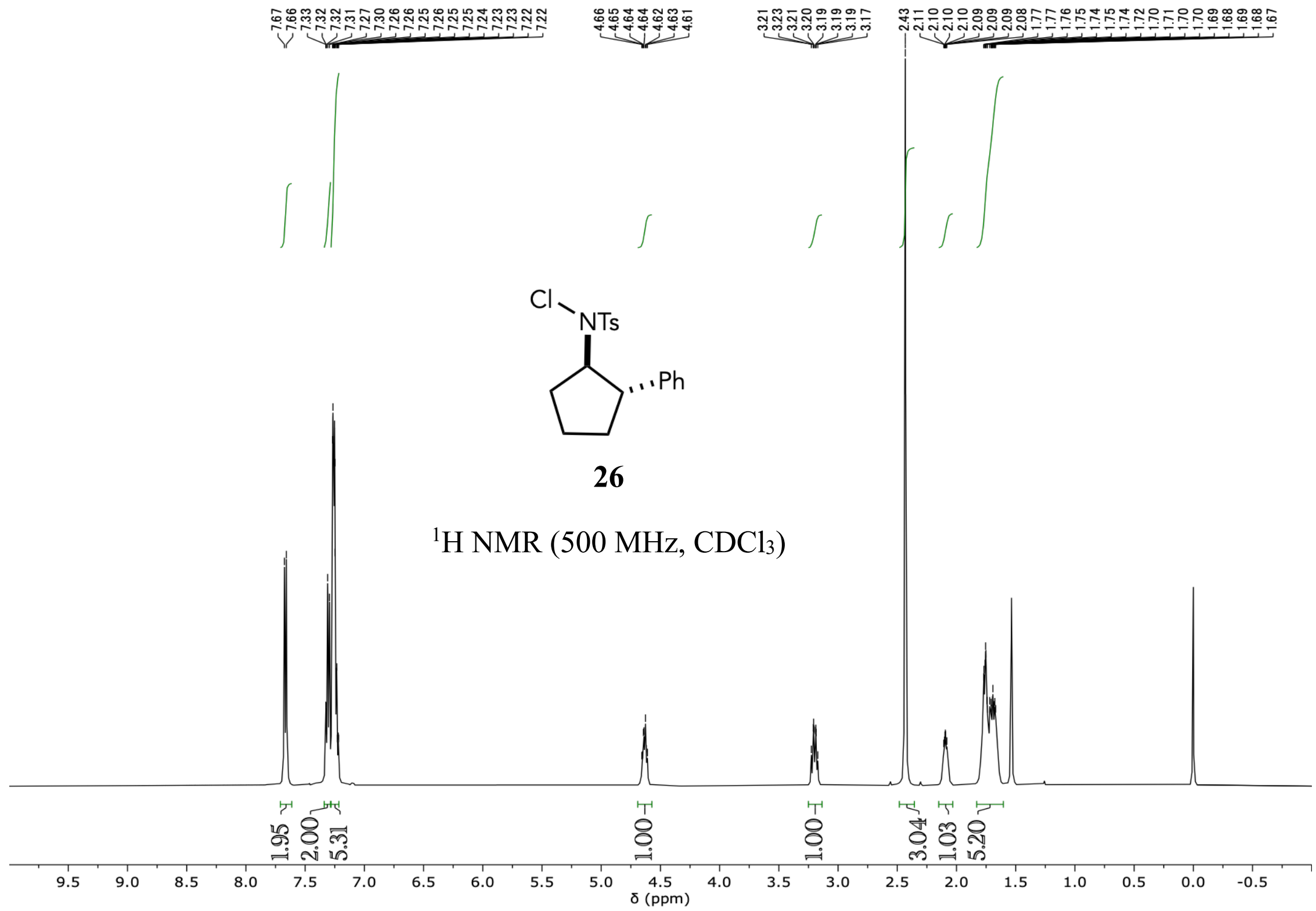
Cl
NTs
Ph
26
¹H NMR (500 MHz, CDCl₃)
1.95
2.00
5.31
1.00
1.00
3.04
1.03
5.20
δ (ppm)

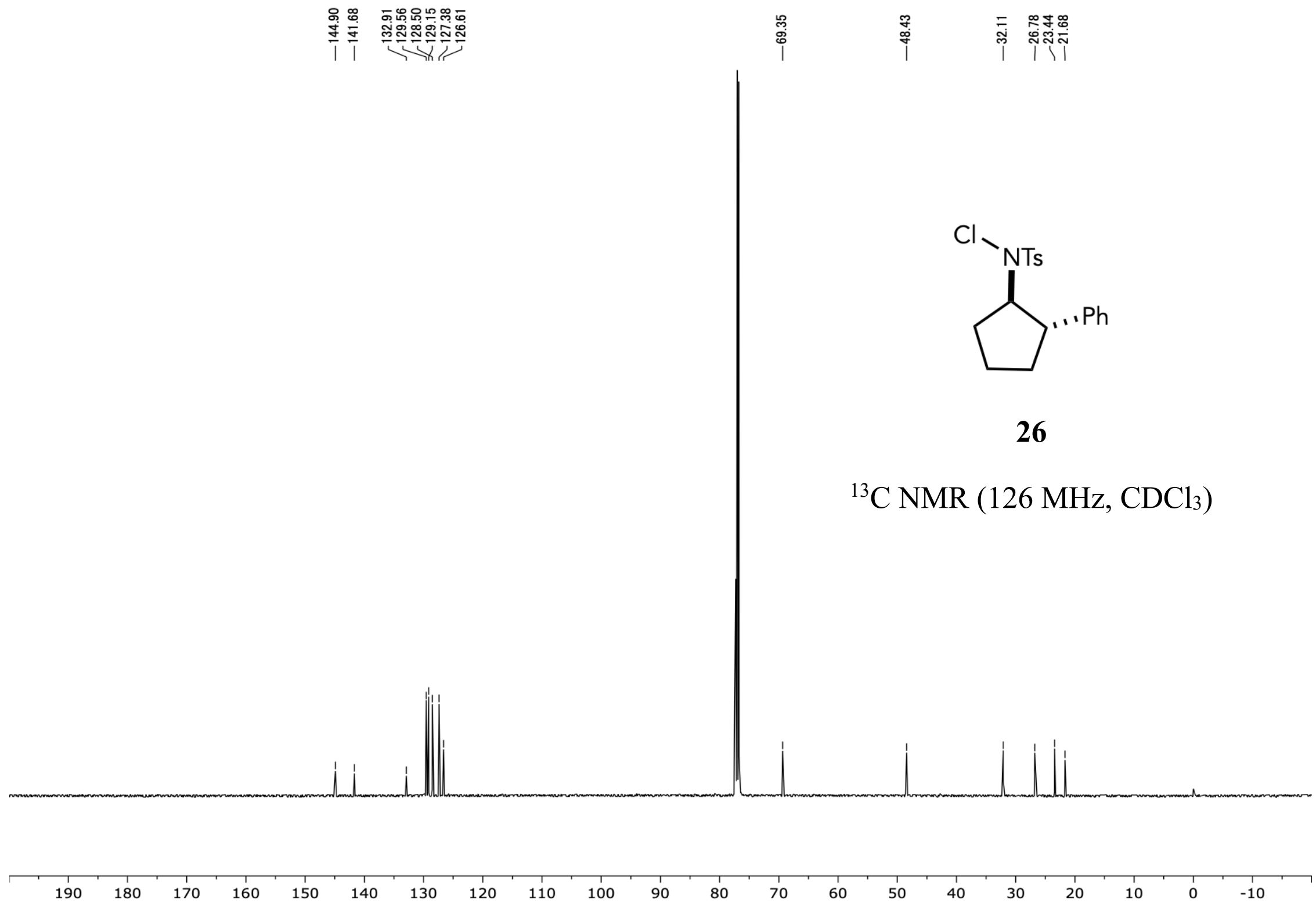
144.90
141.68
132.91
129.56
128.50
129.15
127.38
126.61
69.35
48.43
32.11
26.78
23.44
21.68
Cl
NTs
Ph
26
¹³C NMR (126 MHz, CDCl₃)
δ (ppm)

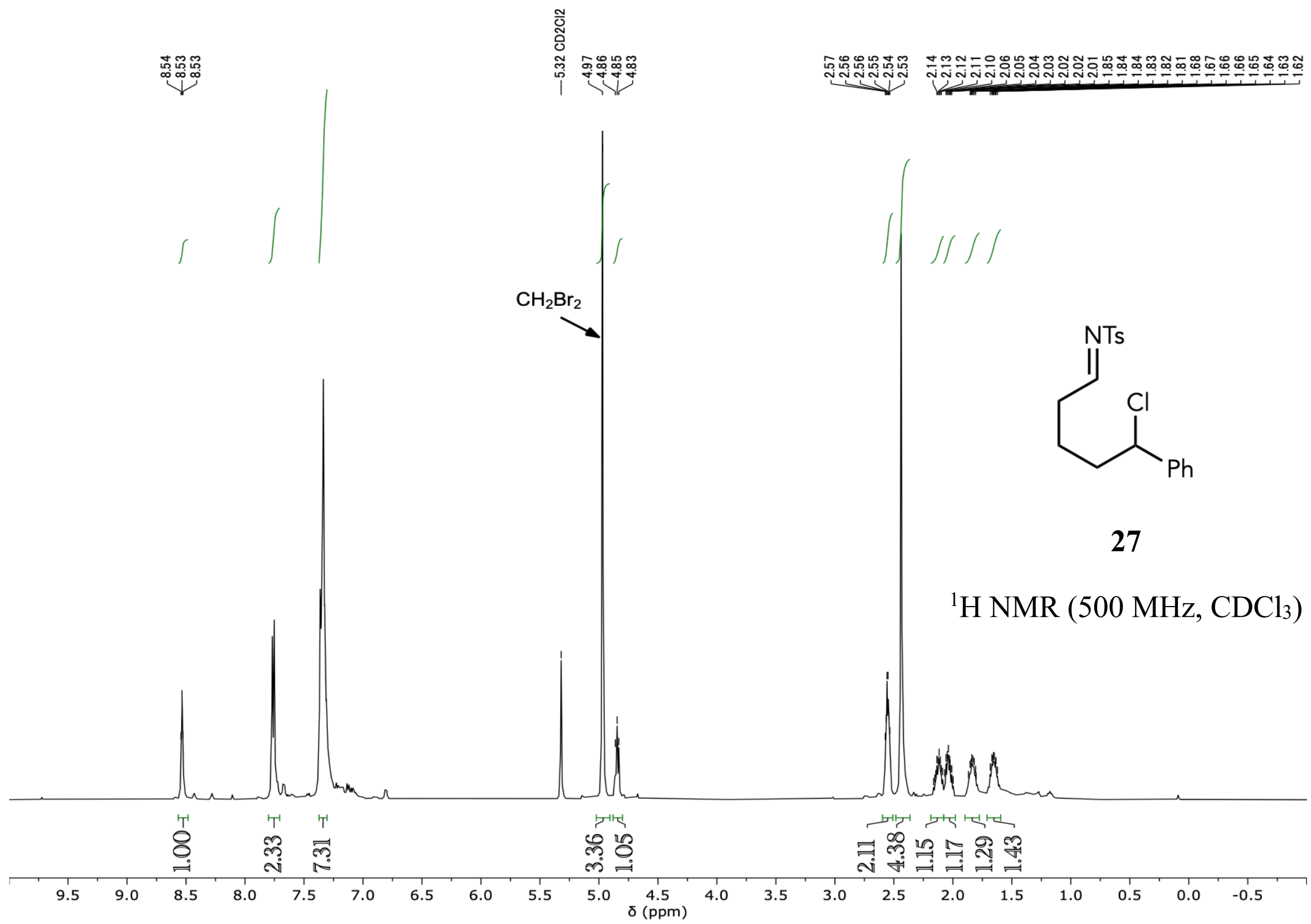

27

^{1}H NMR (500 MHz, CDCl$_3$)

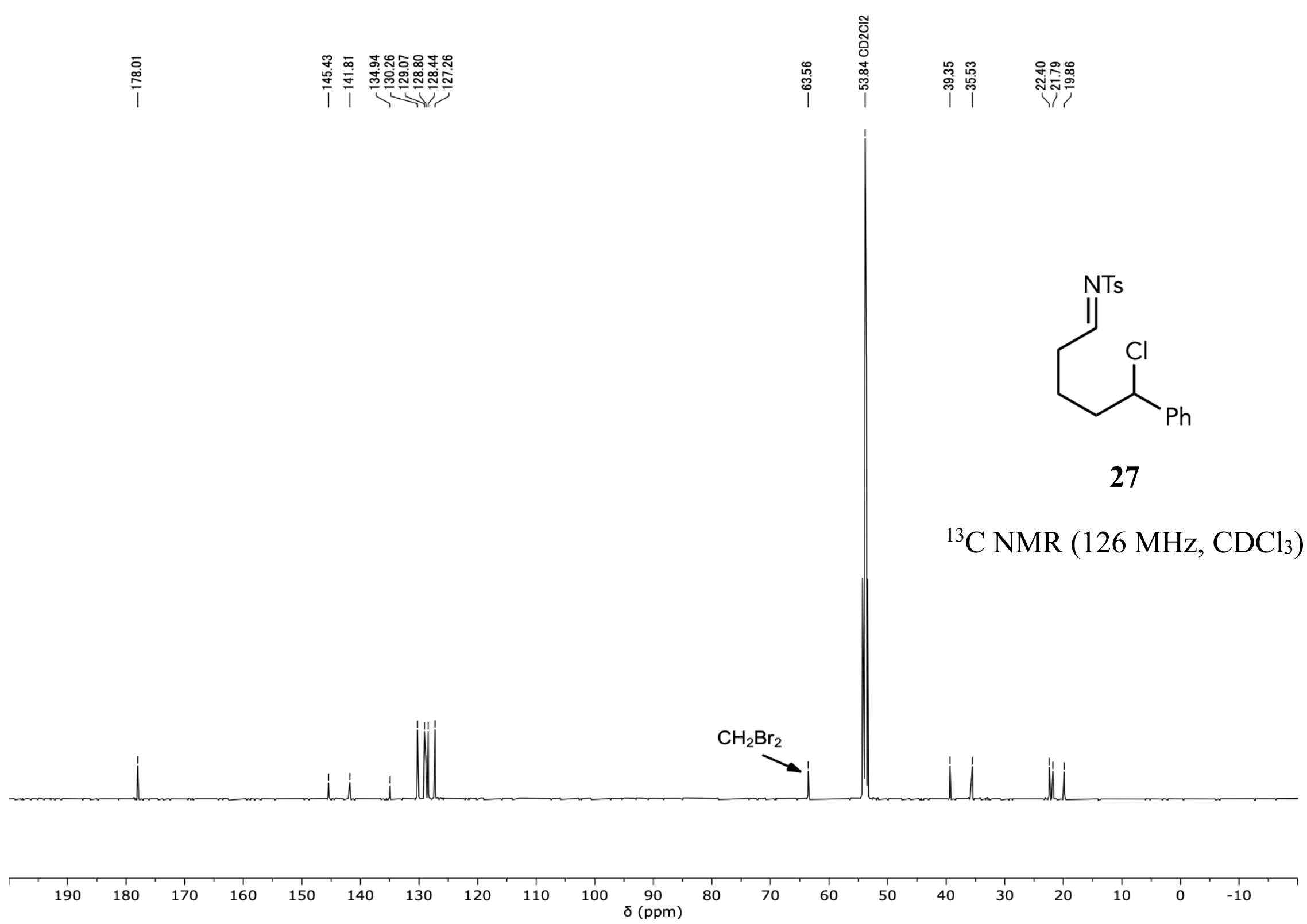

27

^{13}C NMR (126 MHz, CDCl$_3$)

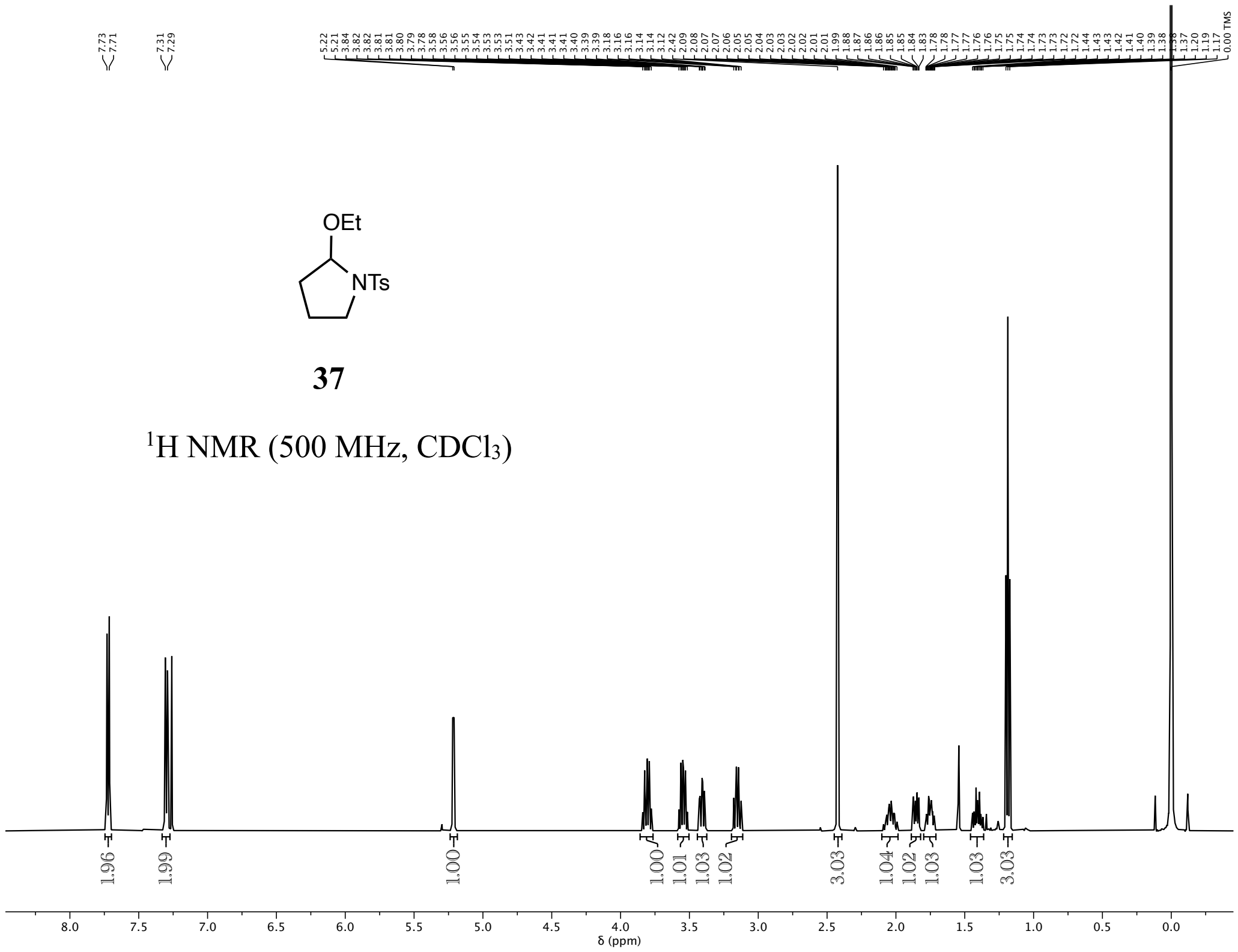

37

^{1}H NMR (500 MHz, $CDCl_3$)

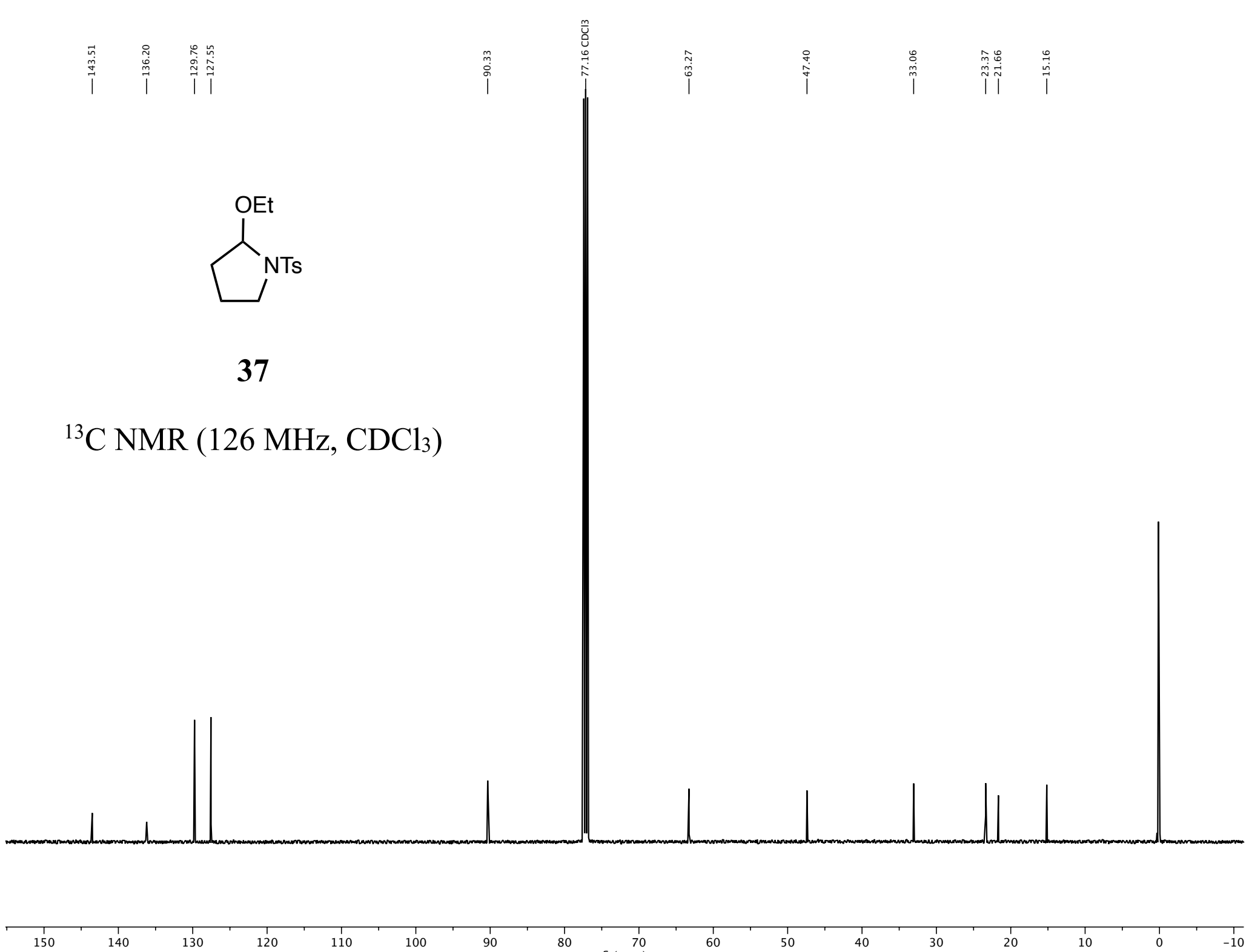

37

^{13}C NMR (126 MHz, $CDCl_3$)

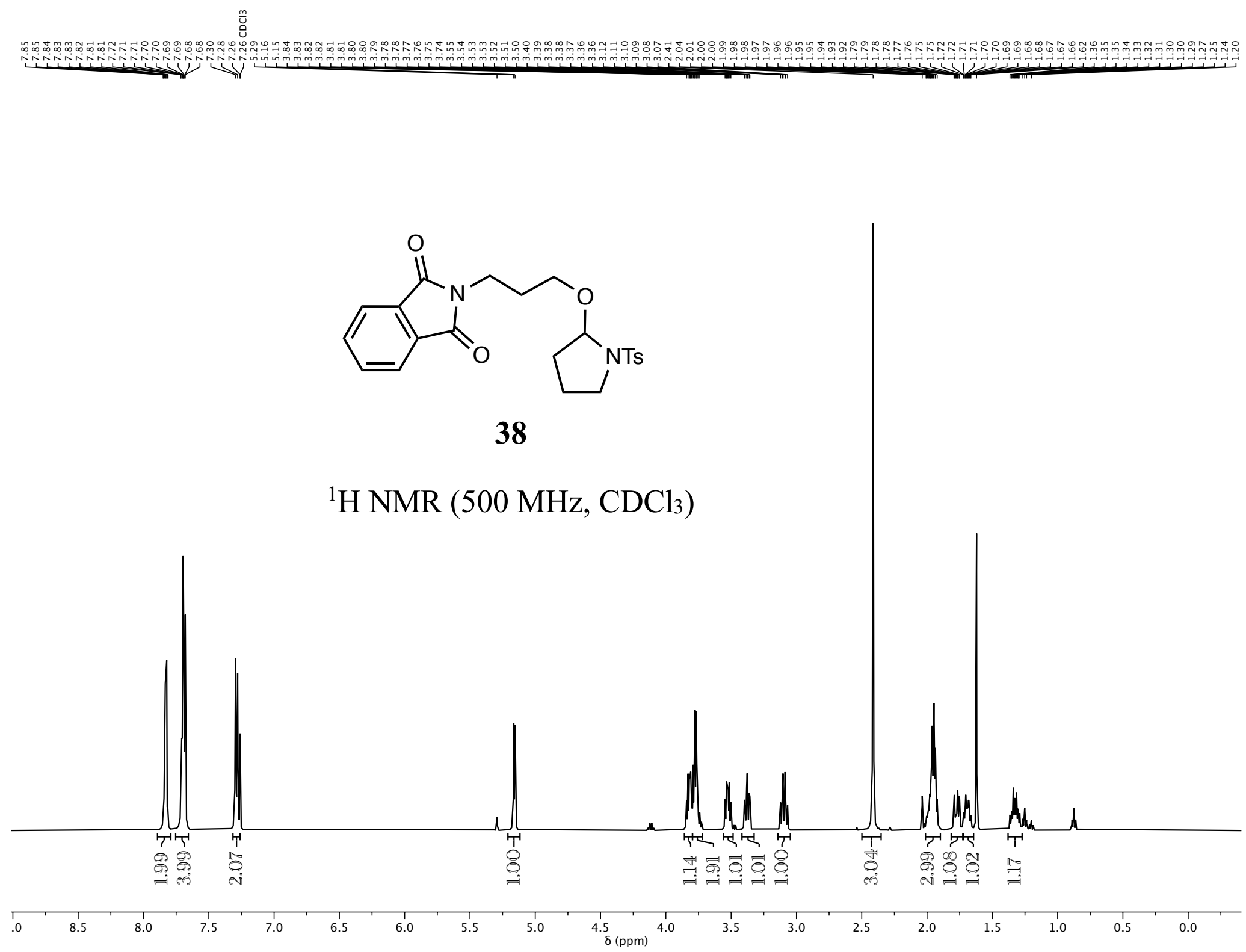
38
1H NMR (500 MHz, CDCl3)
1.99
3.99
2.07
1.00
1.14
1.91
1.01
1.01
1.00
3.04
2.99
1.08
1.02
1.17
δ (ppm)

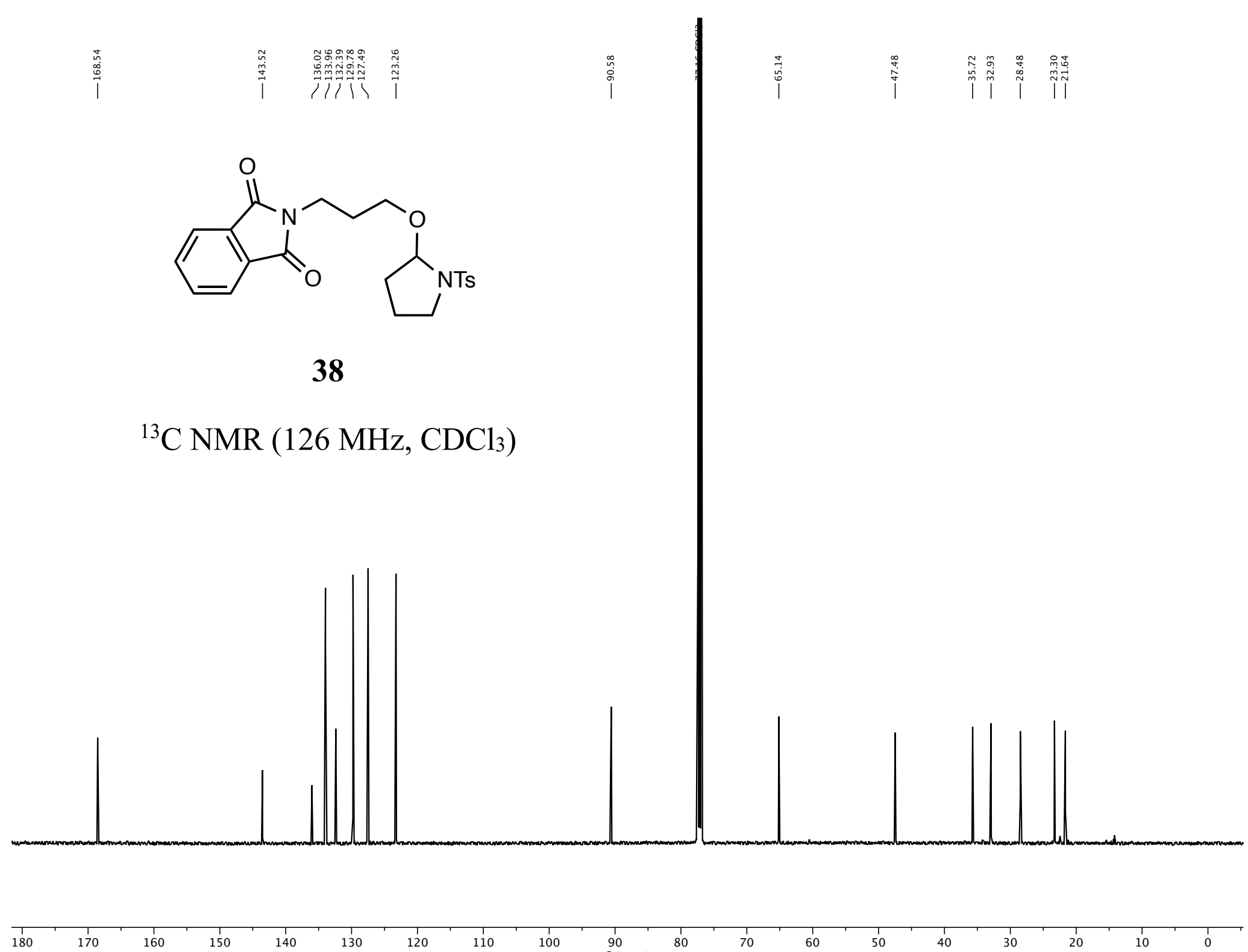
168.54
143.52
136.02
133.96
132.39
129.78
127.49
123.26
90.58
65.14
47.48
35.72
32.93
28.48
23.30
21.64
38
13C NMR (126 MHz, CDCl3)
δ (ppm)

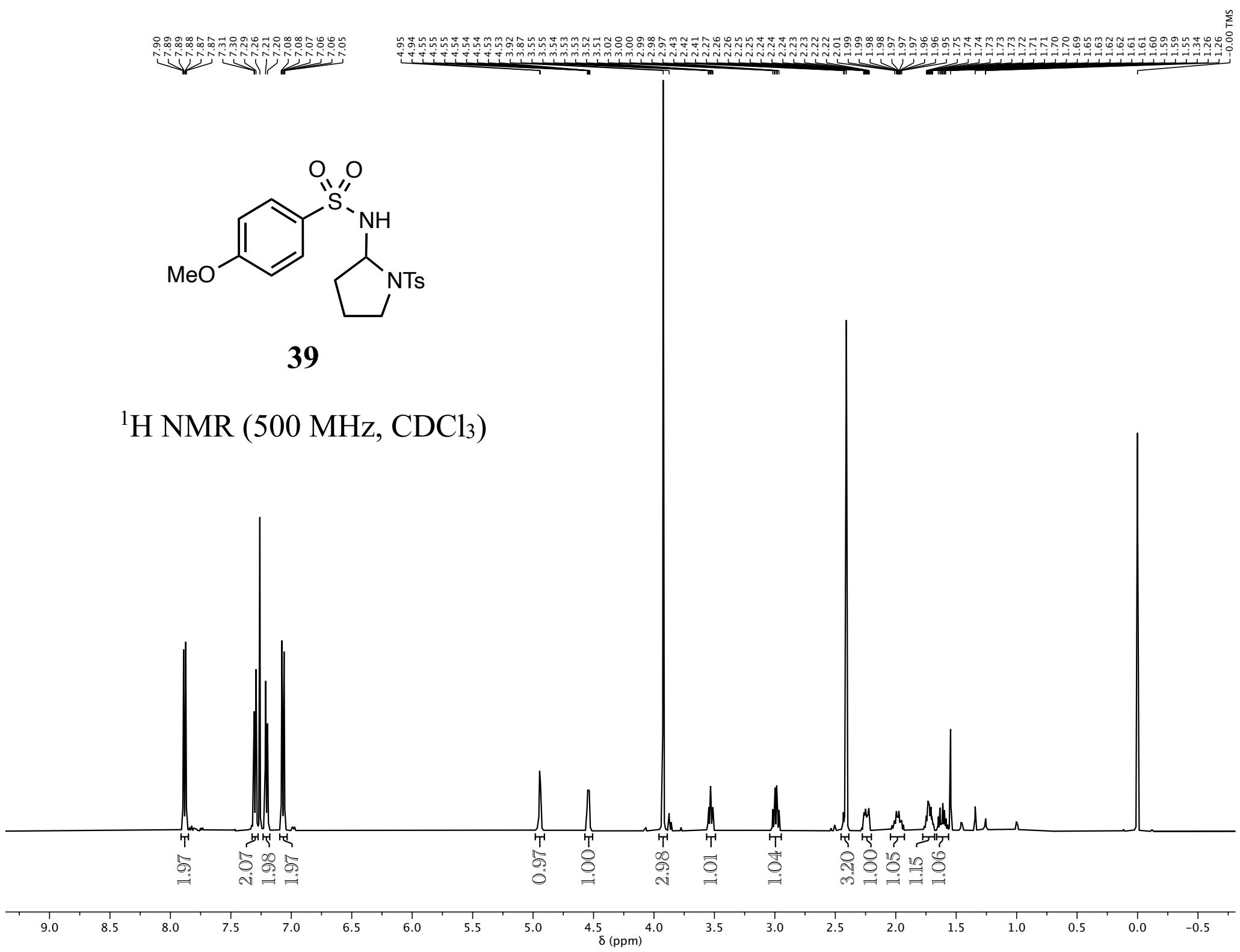

MeO
NH
NTs
39
¹H NMR (500 MHz, CDCl3)
1.97
2.07
1.98
1.97
0.97
1.00
2.98
1.01
1.04
3.20
1.00
1.05
1.15
1.06
δ (ppm)

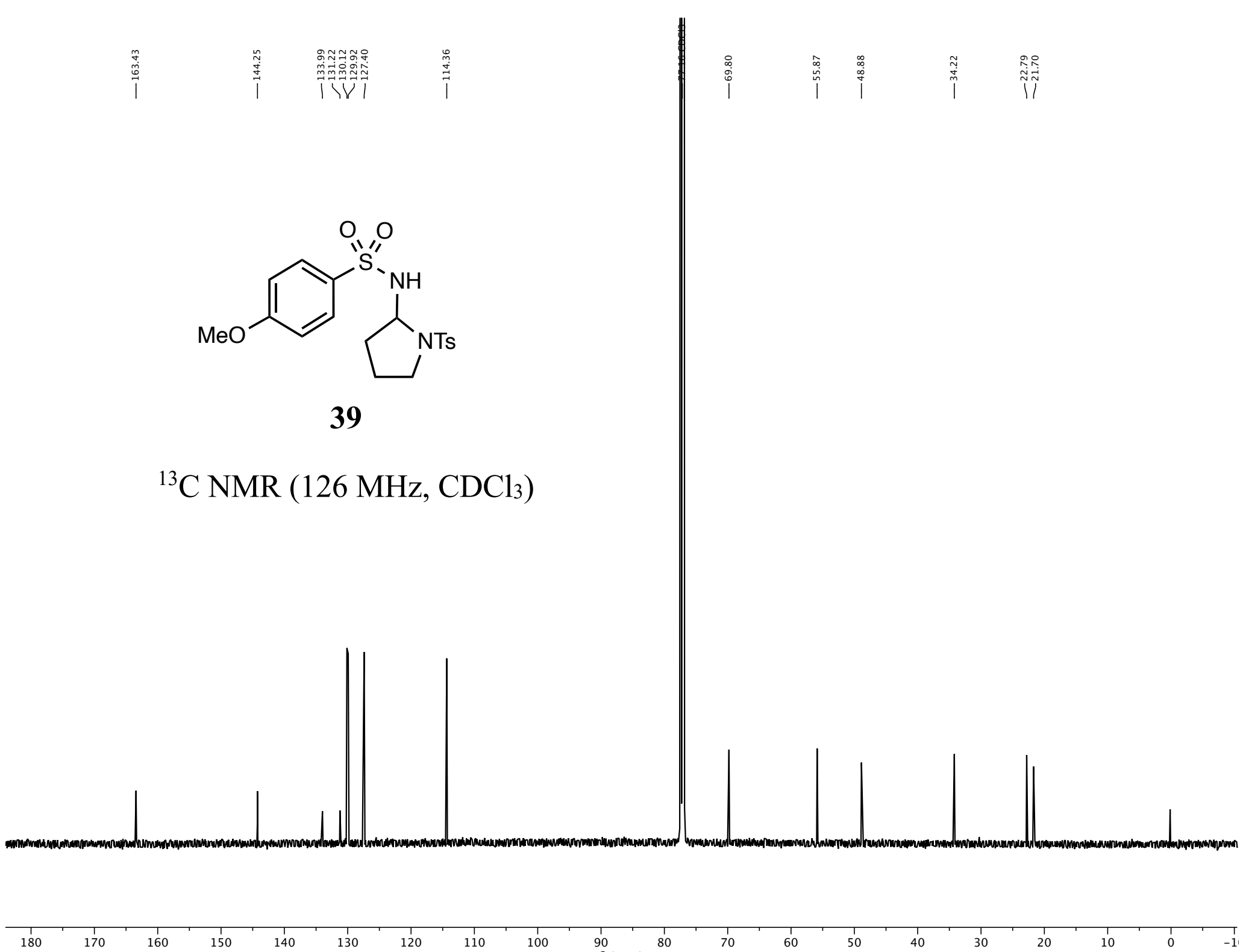

163.43
144.25
133.99
131.22
130.12
129.92
127.40
114.36
69.80
55.87
48.88
34.22
22.79
21.70
MeO
NH
NTs
39
¹³C NMR (126 MHz, CDCl3)
δ (ppm)

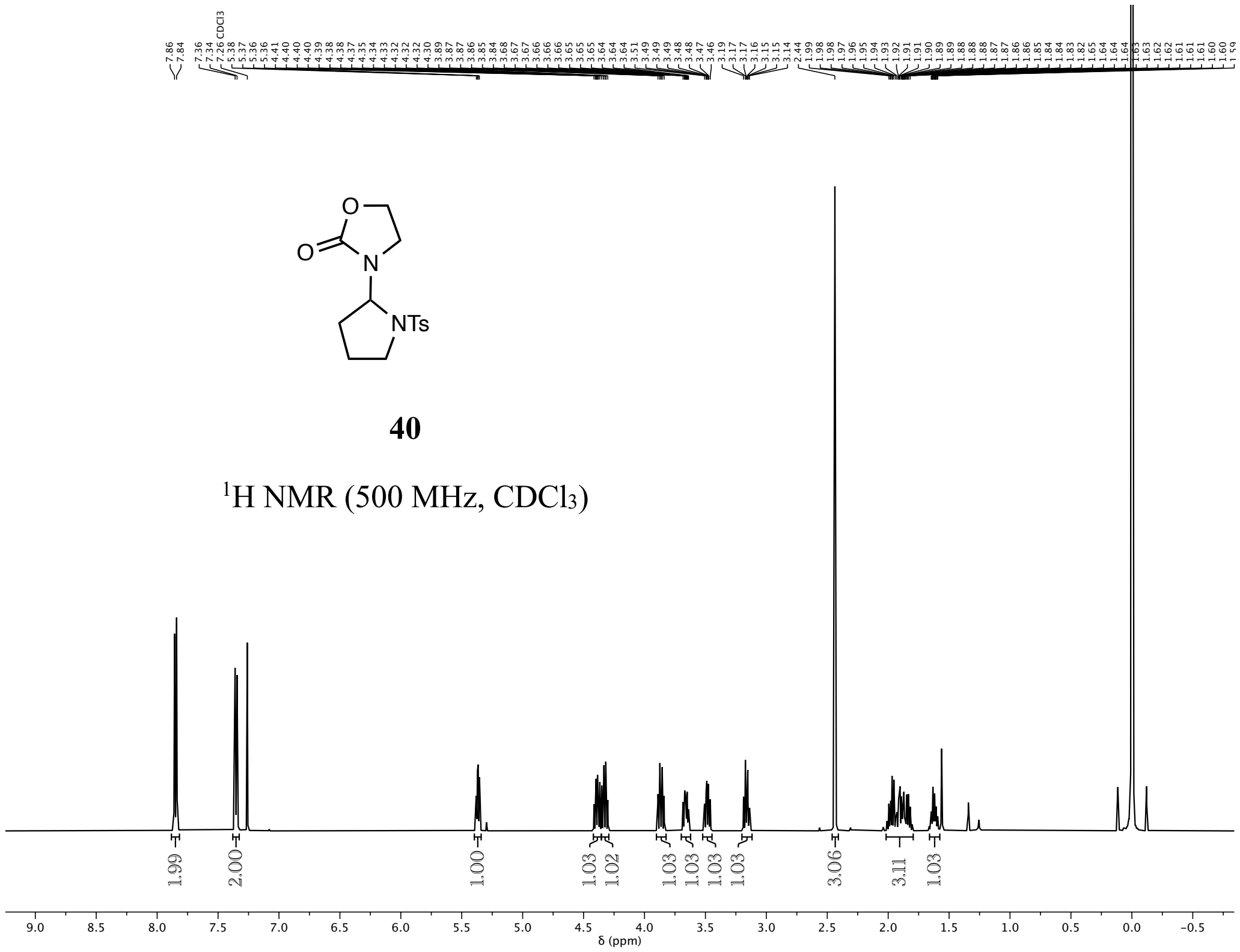

40

^{1}H NMR (500 MHz, $CDCl_3$)

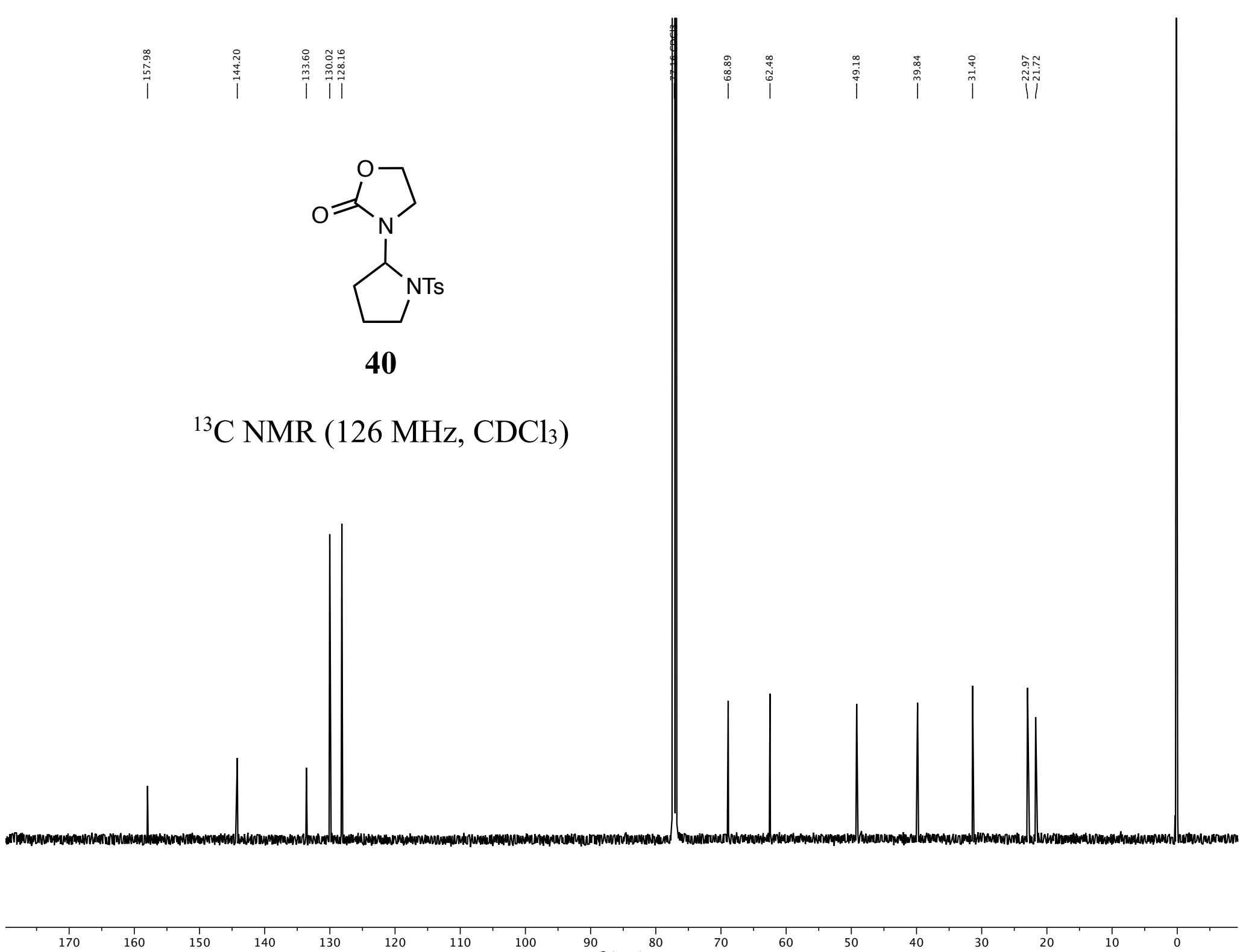

40

^{13}C NMR (126 MHz, $CDCl_3$)

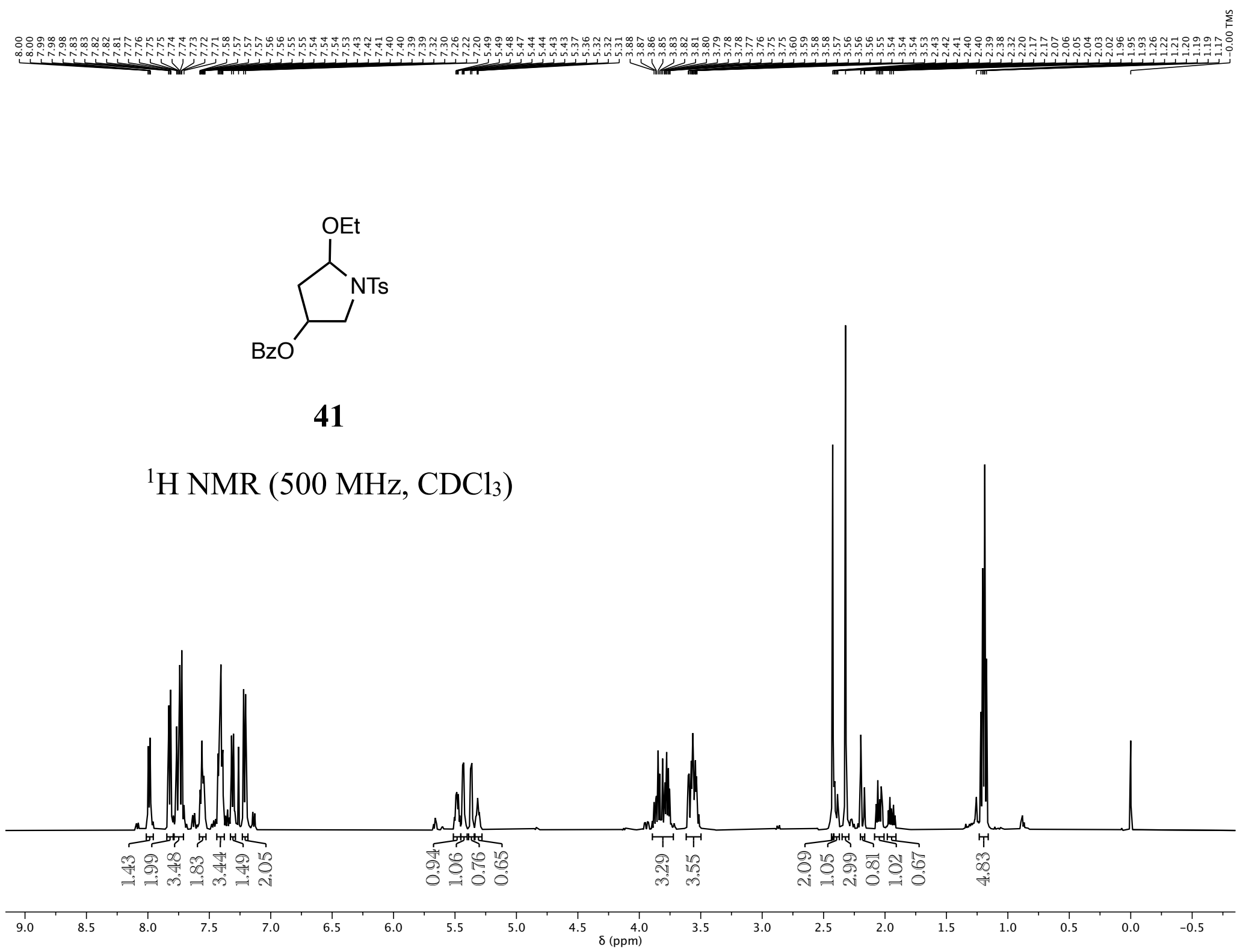
OEt
NTs
BzO
41
¹H NMR (500 MHz, CDCl3)
δ (ppm)

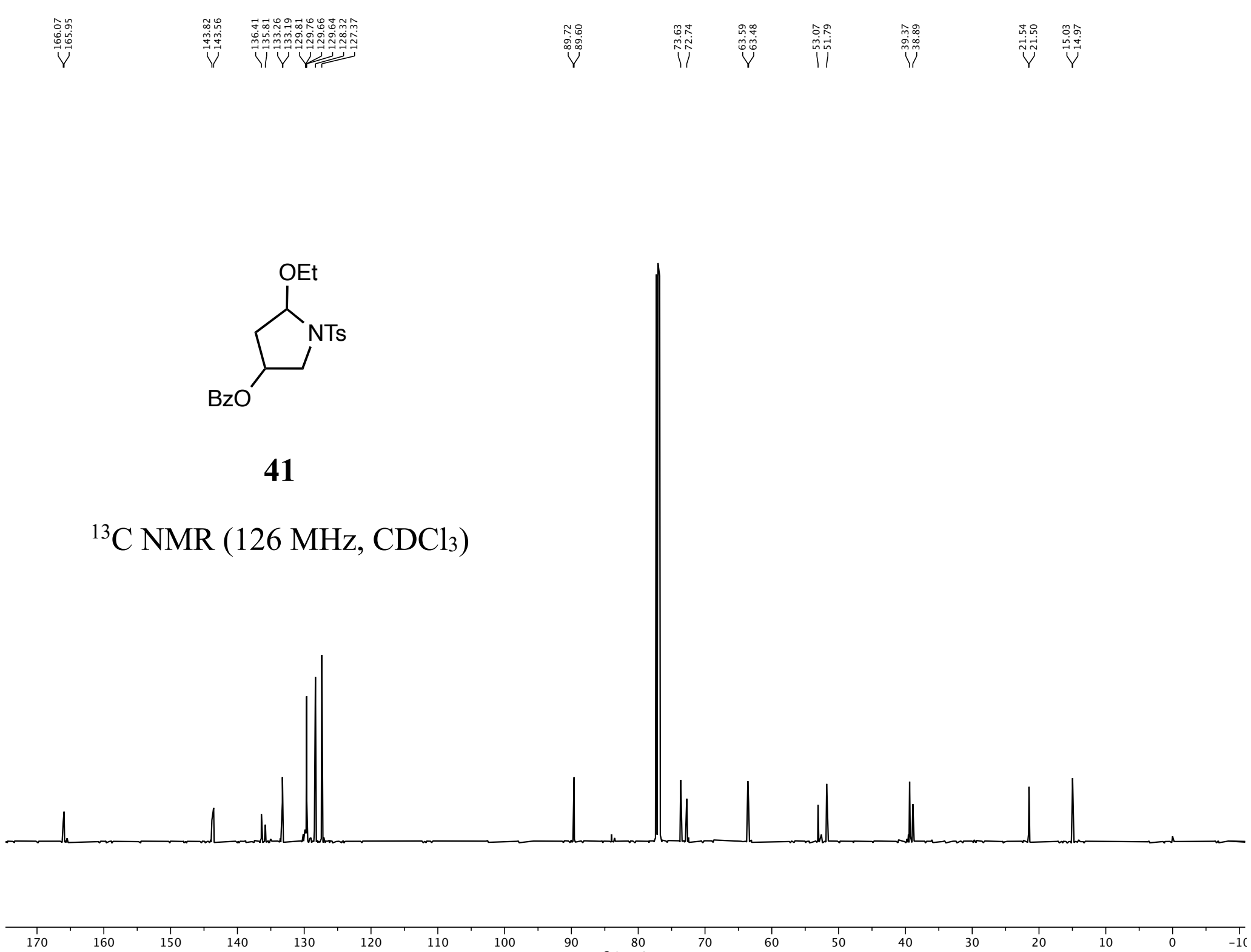
OEt
NTs
BzO
41
¹³C NMR (126 MHz, CDCl3)
δ (ppm)

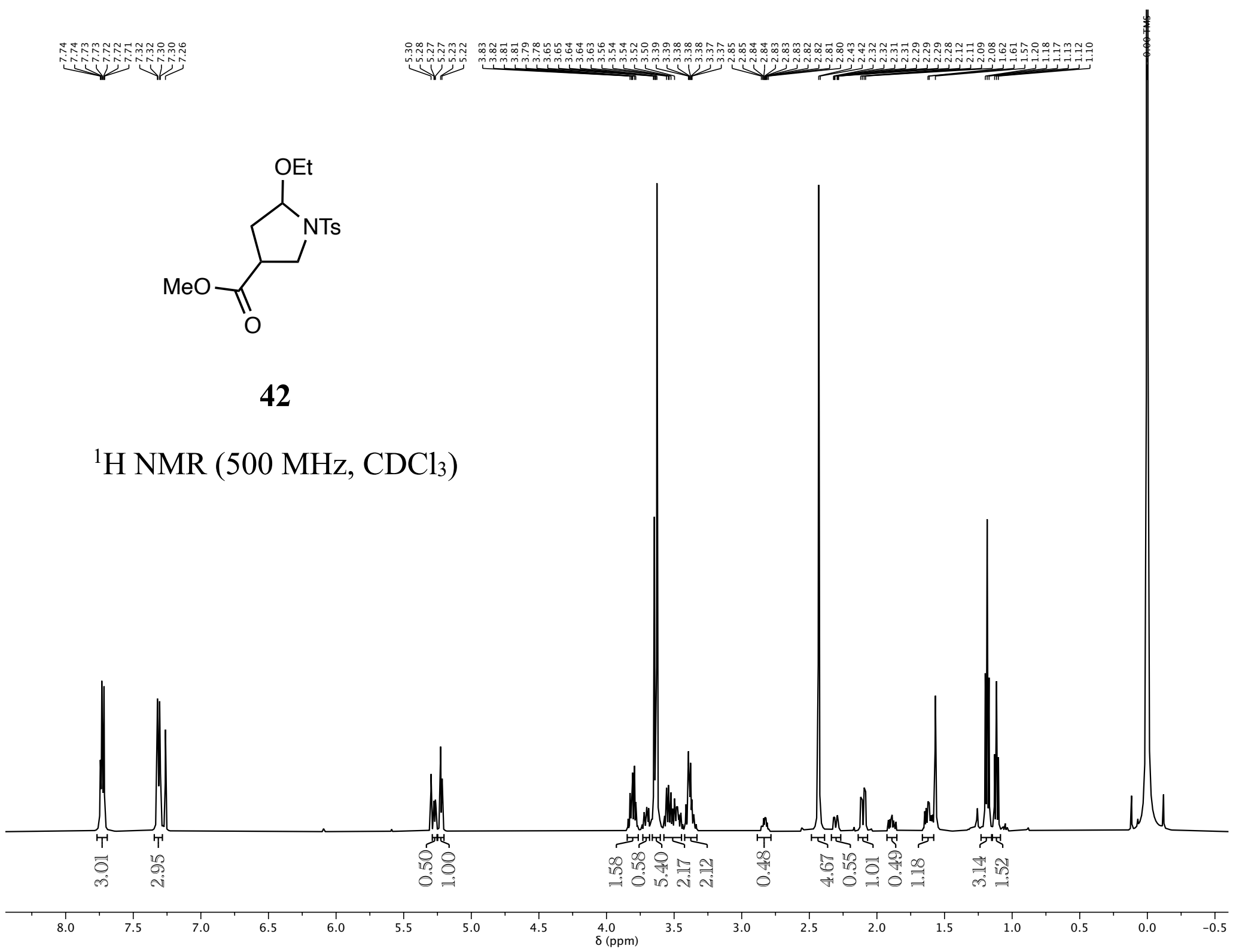
OEt
NTs
MeO
O
42
^{1}H NMR (500 MHz, $CDCl_3$)
δ (ppm)

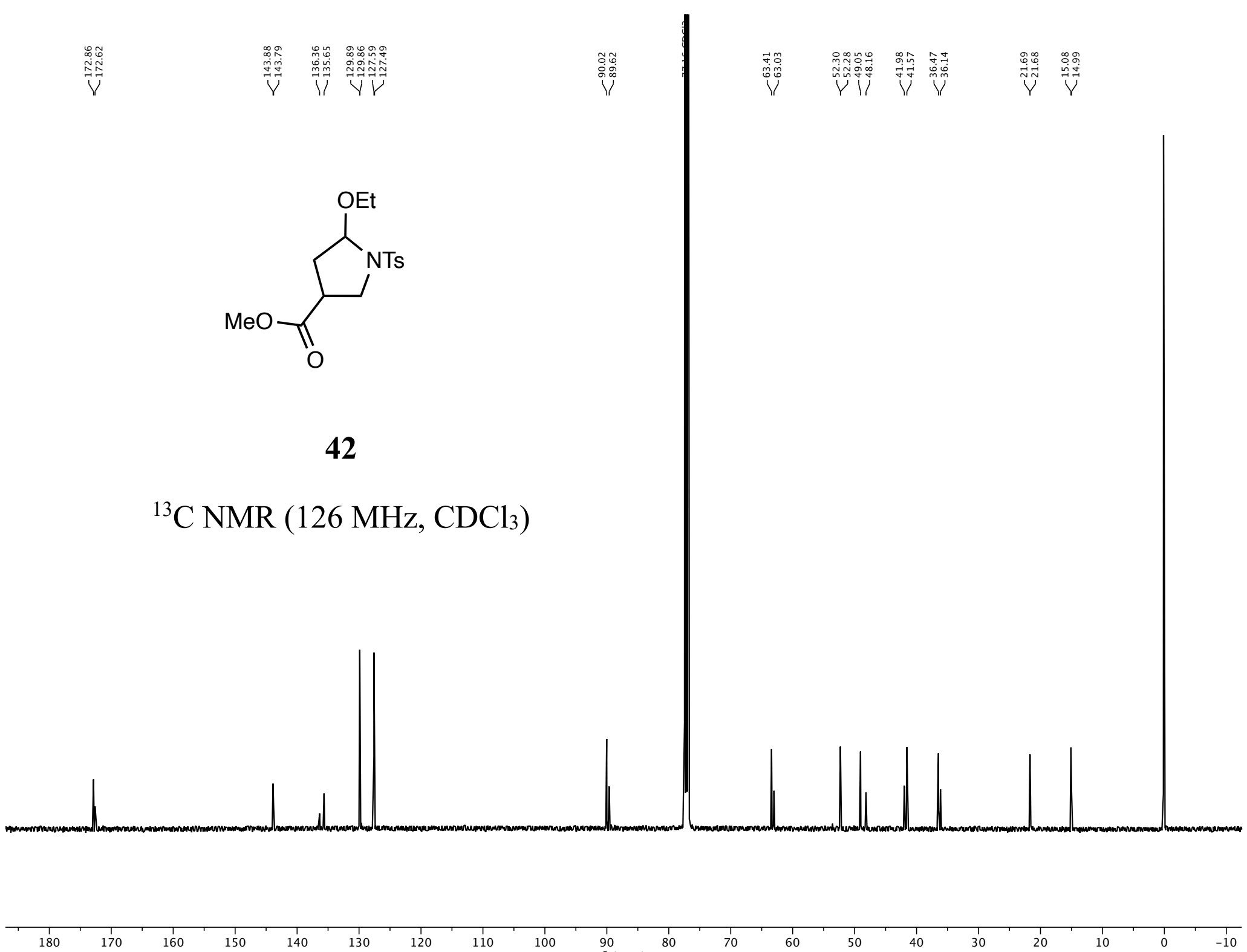
OEt
NTs
MeO
O
42
^{13}C NMR (126 MHz, $CDCl_3$)
δ (ppm)

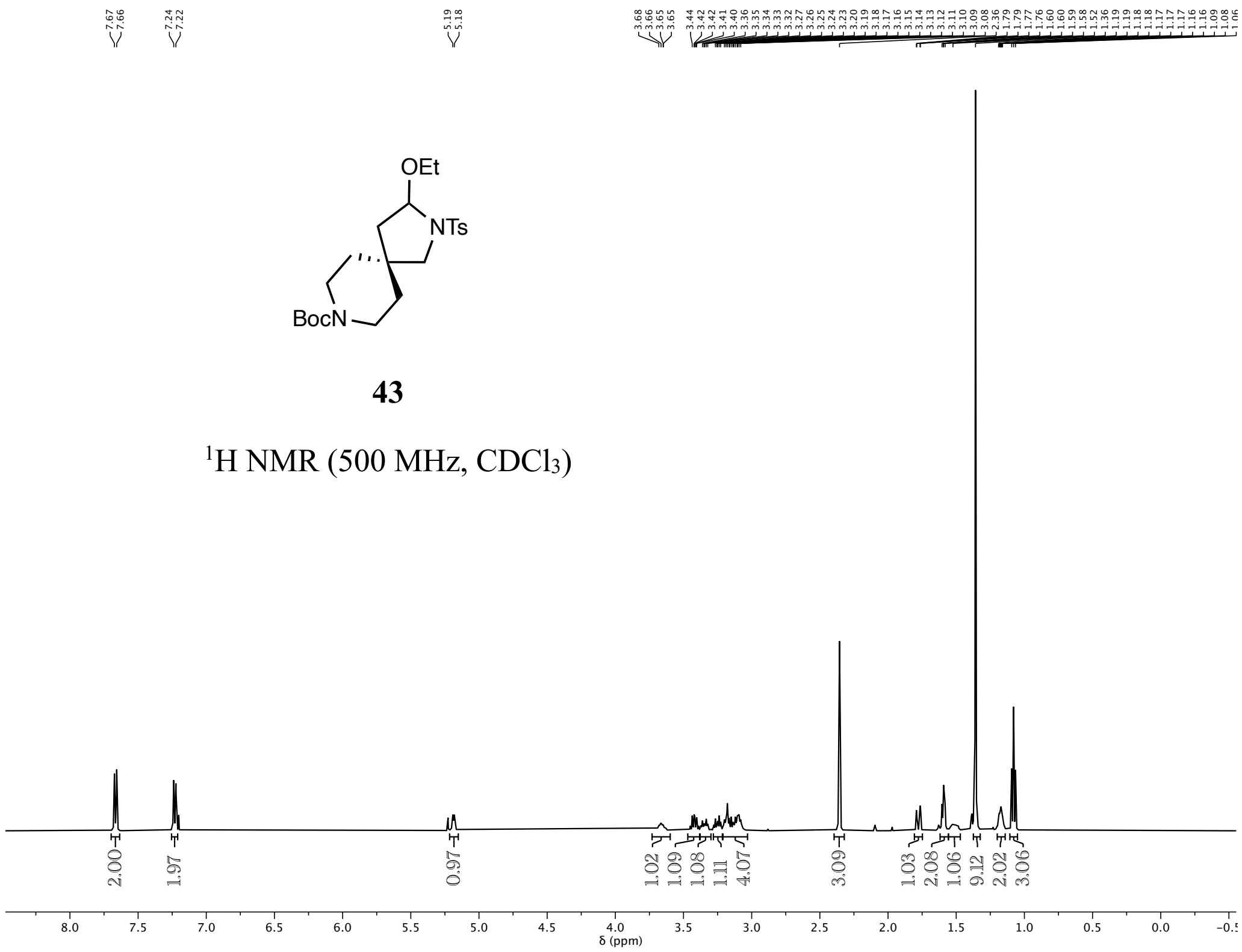

43

^{1}H NMR (500 MHz, $CDCl_3$)

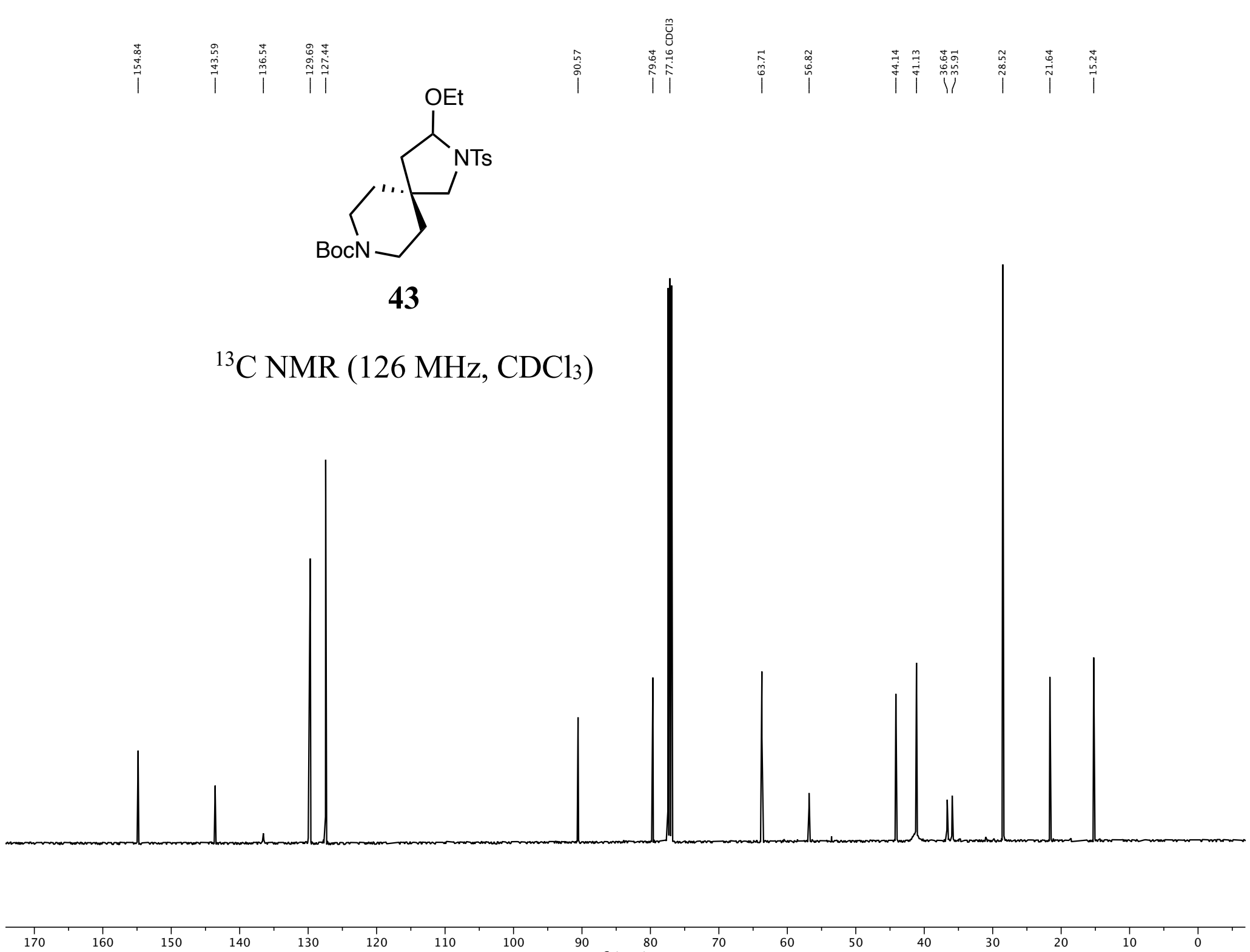

43

^{13}C NMR (126 MHz, $CDCl_3$)

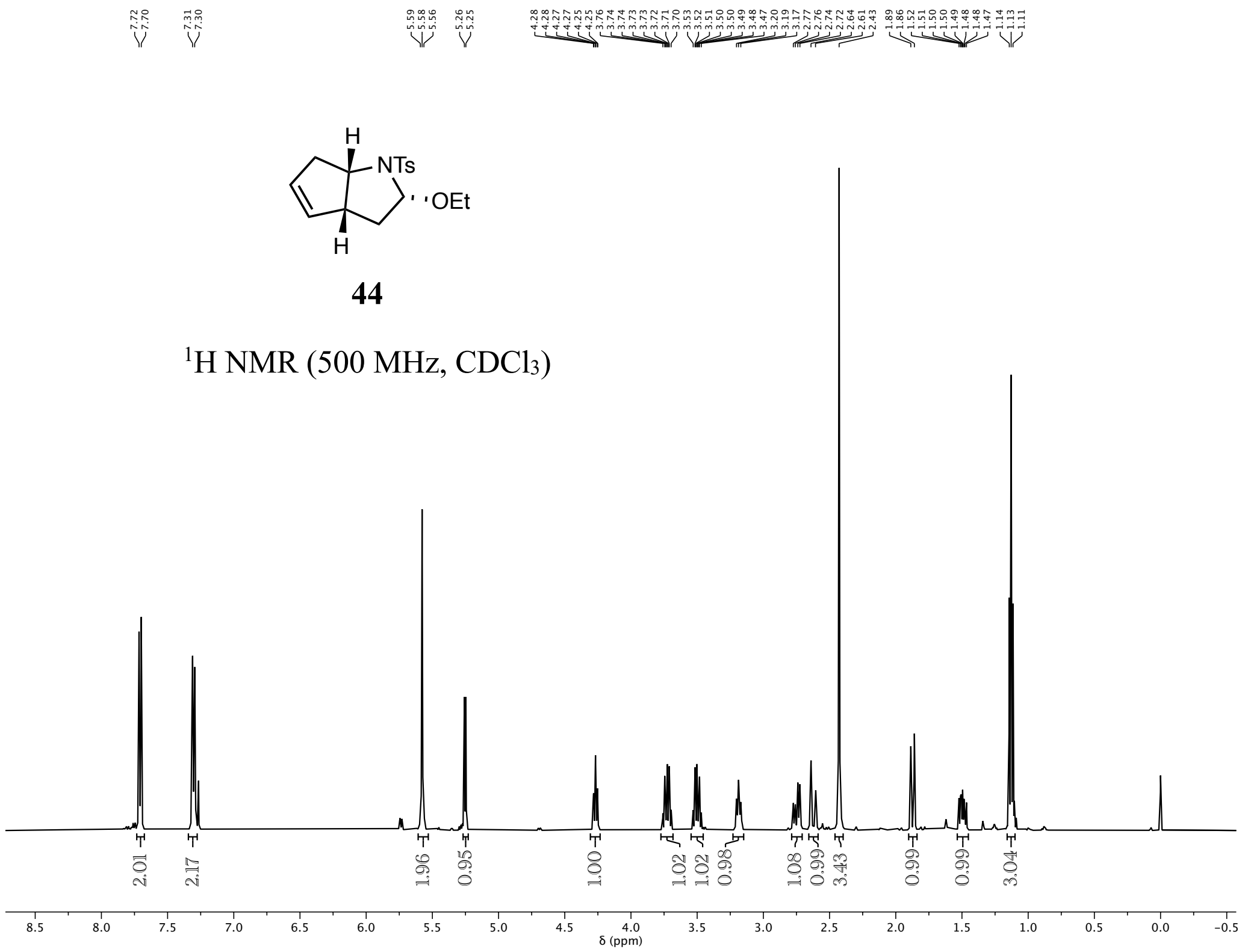
NTs
OEt
H
H
44
1H NMR (500 MHz, CDCl3)
δ (ppm)

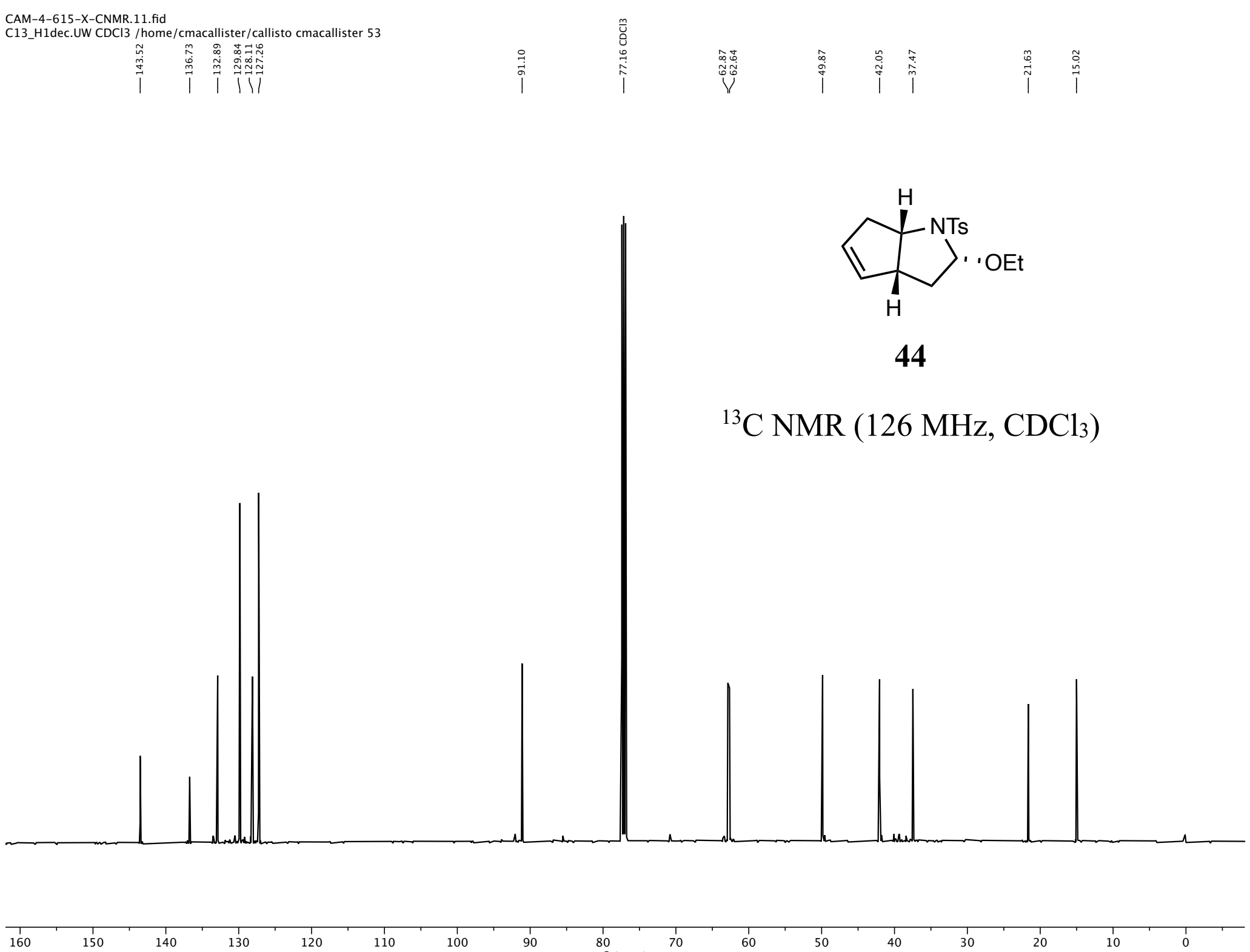
CAM-4-615-X-CNMR.11.fid
C13_H1dec.UW CDCl3 /home/cmacallister/callisto cmacallister 53
NTs
OEt
H
H
44
13C NMR (126 MHz, CDCl3)
δ (ppm)

P. References

[1] A. B. Pangborn, M. A. Giardello, R. H. Grubbs, R. K. Rosen, and F. J. Timmers, *Organometallics* 1996, **15**, 1518.

[2] Meyer, A. R.; Popescu, M. V.; Sau, A.; Damrauer, N. H.; Paton, R. S.; Yoon, T. P. Combined Synthetic, Spectroscopic, and Computational Insights Into a General Method for Photosensitized Alkene Aziridination. *ACS Catal.* **2024**, *14*, 12310–12317.

[3] Sun, B.; Zhang, Q.; Wang, J.; Xu, Y.; Wang, J.; Lv, C.; Zhuang, X.; Yu, C.; Jin, C. Light Empowered Aziridination of Olefins under Metal- and Photocatalyst-Free Conditions. *Green Chem.* **2025**, *27*, 8980–8987.

[4] Kumar, M.; Verma, S.; Mishra, V.; Reiser, O.; Verma, A. K. Visible-Light-Accelerated Copper-Catalyzed [3 + 2] Cycloaddition of N-Tosylcyclopropylamines with Alkynes/Alkenes. *J. Org. Chem.* **2022**, *87*, 6263–6272.

[5] Xu, Y.; Su, T.; Huang, Z.; Dong, G. Practical Direct α-Arylation of Cyclopentanones by Palladium/Enamine Cooperative Catalysis. *Angew. Chem. Int. Ed.* **2016**, *55*, 2559–2563.

[6] Wen, L.; Ding, J.; Duan, L.; Wang, S.; An, Q.; Wang, H.; Zuo, Z. Multiplicative Enhancement of Stereoenrichment by a Single Catalyst for Deracemization of Alcohols. *Science* **2023**, *382*, 458–464.

[7] Meyer, D.; Renaud, P. Enantioselective Hydroazidation of Trisubstituted Non-Activated Alkenes. *Angew. Chem. Int. Ed.* **2017**, *56*, 10858–10861.

[8] Richter, J. M.; Cheney, D. L.; Bates, J. A.; Wei, A.; Luettgen, J. M.; Rendina, A. R.; Harper, T. M.; Narayanan, R.; Wong, P. C.; Seiffert, D.; Wexler, R. R.; Priestley, E. S. Design and Synthesis of Novel Meta-Linked Phenylglycine Macrocyclic FVIIa Inhibitors. *ACS Med. Chem. Lett.* **2017**, *8*, 67–72.

[9] Zhu, Y.; Smith, M. J. S.; Tu, W.; Bower, J. F. A Stereospecific Alkene 1,2-Aminofunctionalization Platform for the Assembly of Complex Nitrogen-Containing Ring Systems. *Angew. Chem.* **2023**, *62*, e202301262.

[10] Xu, R.; Zhou, H.; Bae, H. Y.; Wakchaure, V. N.; Baldinelli, L.; Leach, I. F.; Bistoni, G.; Kraft, P.; List, B. The Catalytic Asymmetric Mukaiyama–Michael Reaction of Silyl Ketene Acetals with Cyclic Enones: Short Routes to Jasmonates. *J. Am. Chem. Soc.* **2026**, *148*, 6779–6785.

[11] Xiong, Y.; Lin, H.; Zhu, C.-L.; Chen, Y.-H.; Ye, R.; Hu, G.-W.; Xie, J.-H.; Zhou, Q.-L. Asymmetric Hydrogenation of Racemic α-Aryl-β-Ethoxycarbonyl Cyclopentanones via Dynamic Kinetic Resolution and Its Application to the Synthesis of (+)-Burmaniol A. *Org. Lett.* **2021**, *23*, 8883–8887.

[12] Hong, V. S.; Jeong, S.; Yun, Y.; Choo, H.; Won, J.; Lee, J. 1,3,4-Oxadiazole-2(3H)-Thione Analogs as PIM Kinase Inhibitors. *Bulletin of the Korean Chemical Society* **2020**, *41* (10), 994–1001.

[13] White, D. H.; Noble, A.; Booker-Milburn, K. I.; Aggarwal, V. K. Diastereoselective Photoredox-Catalyzed [3 + 2] Cycloadditions of N-Sulfonyl Cyclopropylamines with Electron-Deficient Olefins. *Org. Lett.* **2021**, *23*, 3038–3042.

[14] Huang, Z.; Wang, C.; Dong, G. A Hydrazone-Based Exo-Directing-Group Strategy for β C−H Oxidation of Aliphatic Amines. *Angew. Chem. Int. Ed.* **2016**, *55*, 5299–5303.

[15] López, M. M.; Jamey, N.; Pinet, A.; Figadère, B.; Ferrié, L. Oxidative Ring Expansion of Cyclobutanols: Access to Functionalized 1,2-Dioxanes. *Org. Lett.* **2021**, *23*, 1626–1631.

[16] Harwood, L. A.; Xiong, Z.; Christensen, K. E.; Wang, R.; Wong, L. L.; Robertson, J. Selective P450BM3 Hydroxylation of Cyclobutylamine and Bicyclo[1.1.1]Pentylamine Derivatives: Underpinning Synthetic Chemistry for Drug Discovery. *J. Am. Chem. Soc.* **2023**, *145*, 27767–27773.

[17] Sutton, A. E.; Seigal, B. A.; Finnegan, D. F.; Snapper, M. L. New Tandem Catalysis: Preparation of Cyclic Enol Ethers through a Ruthenium-Catalyzed Ring-Closing Metathesis−Olefin Isomerization Sequence. *J. Am. Chem. Soc.* **2002**, *124*, 13390–13391.

[18] Zhang, H.; Muñiz, K. Selective Piperidine Synthesis Exploiting Iodine-Catalyzed Csp^3–H Amination under Visible Light. *ACS Catal.* **2017**, *7*, 4122–4125.

[19] Wang, J.; Lu, H.; He, Y.; Jing, C.; Wei, H. Cobalt-Catalyzed Nitrogen Atom Insertion in Arylcycloalkenes. *J. Am. Chem. Soc.* **2022**, *144*, 22433–22439.

[20] Kamimura, A.; Yo, R.; Uno, H. Preparation of 2,3-*Trans*-Substituted Piperidines from Optically Active β-Amino-α-Methylene Esters: Synthesis of Optically Active (2*S*,3*R*)-(−)-*Epi*-CP-99,994. *Tetrahedron* **2017**, *73*, 4770–4778.

[21] Wang, W.; Cao, X.; Xiao, W.; Shi, X.; Zuo, X.; Liu, L.; Chang, W.; Li, J. Stereospecific Synthesis of Cis-2,5-Disubstituted Pyrrolidines via N,O-Acetals Formed by Hydroamination Cyclization–Hydroalkoxylation of Homopropargylic Sulfonamides in HFIP. *J. Org. Chem.* **2020**, *85*, 7045–7059.

[22] Liu, R.-Z.; Li, J.; Sun, J.; Liu, X.-G.; Qu, S.; Li, P.; Zhang, B. Generation and Reactivity of Amidyl Radicals: Manganese-Mediated Atom-Transfer Reaction. *Angew. Chem. Int. Ed.* **2020**, *59*, 4428–4433.

[23] Wappes, E. A.; Fosu, S. C.; Chopko, T. C.; Nagib, D. A. Triiodide-Mediated δ-Amination of Secondary C−H Bonds. *Angew. Chem. Int. Ed.* **2016**, *55*, 9974–9978.

[24] MacAllister, C. A.; Lacker, C. R.; Maciejewski, M. F.; Wessels, F.; Bates, D. M.; Bagley, S. W.; Yoon, T. P. Oxygen Migration into Carbon–Carbon Single Bonds by Photochemical Oxidation. *Nat. Synth.* **2026**, 5, 349–356.

[25] Frisch, M. J.; Trucks, G. W.; Schlegel, H. B.; Scuseria, G. E.; Robb, M. A.; Cheeseman, J. R.; Scalmani, G.; Barone, V.; Petersson, G. A.; Nakatsuji, H.; Li, X.; Caricato, M.; Marenich, A. V.; Bloino, J.; Janesko, B. G.; Gomperts, R.; Mennucci, B.; Hratchian, H. P.; Ortiz, J. V.; Izmaylov, A. F.; Sonnenberg, J. L.; Williams-Young, D.; Ding, F.; Lipparini, F.; Egidi, F.; Goings, J.; Peng, B.; Petrone, A.; Henderson, T.; Ranasinghe, D.; Zakrzewski, V. G.; Gao, J.; Rega, N.; Zheng, G.; Liang, W.; Hada, M.; Ehara, M.; Toyota, K.; Fukuda, R.; Hasegawa, J.; Ishida, M.; Nakajima, T.; Honda, Y.; Kitao, O.; Nakai, H.; Vreven, T.; Throssell, K.; Montgomery, J. A., Jr.; Peralta, J. E.; Ogliaro, F.; Bearpark, M. J.; Heyd, J. J.; Brothers, E. N.; Kudin, K. N.; Staroverov, V. N.; Keith, T. A.; Kobayashi, R.; Normand, J.; Raghavachari, K.; Rendell, A. P.; Burant, J. C.; Iyengar, S. S.; Tomasi, J.; Cossi, M.; Millam, J. M.; Klene, M.; Adamo, C.; Cammi, R.; Ochterski, J. W.; Martin, R. L.; Morokuma, K.; Farkas, O.; Foresman, J. B.; Fox, D. J. *Gaussian 16* , Revision C.01, **2016**.

[26] Cramer, C. J. *Essentials of Computational Chemistry: Theories and Models*, **2014**.
